# Spatially defined single-cell transcriptional profiling characterizes diverse chondrocyte subtypes and nucleus pulposus progenitors in human intervertebral discs

**DOI:** 10.1038/s41413-021-00163-z

**Published:** 2021-08-16

**Authors:** Yibo Gan, Jian He, Jun Zhu, Zhengyang Xu, Zhong Wang, Jing Yan, Ou Hu, Zhijie Bai, Lin Chen, Yangli Xie, Min Jin, Shuo Huang, Bing Liu, Peng Liu

**Affiliations:** 1 Department of Spine Surgery, Center of Orthopedics, Daping Hospital, Army Medical University (Third Military Medical University), Chongqing, China; 2 State Key Laboratory of Trauma, Burns and Combined Injury, Army Medical University (Third Military Medical University), Chongqing, China; 3 State Key Laboratory of Proteomics, Academy of Military Medical Sciences, Academy of Military Sciences, Beijing, China; 4 Center of Bone Metabolism and Repair, State Key Laboratory of Trauma, Burns and Combined Injury, Trauma Center, Research Institute of Surgery, Laboratory for the Prevention and Rehabilitation of Military Training Related Injuries, Daping Hospital, Army Medical University (Third Military Medical University), Chongqing, China; 5 State Key Laboratory of Experimental Hematology, Institute of Hematology, Fifth Medical Center of Chinese PLA General Hospital, Beijing, China; 6 Key Laboratory for Regenerative Medicine of Ministry of Education, Institute of Hematology, School of Medicine, Jinan University, Guangzhou, China

**Keywords:** Bone quality and biomechanics, Bone

## Abstract

A comprehensive understanding of the cellular heterogeneity and molecular mechanisms underlying the development, homeostasis, and disease of human intervertebral disks (IVDs) remains challenging. Here, the transcriptomic landscape of 108 108 IVD cells was mapped using single-cell RNA sequencing of three main compartments from young and adult healthy IVDs, including the nucleus pulposus (NP), annulus fibrosus, and cartilage endplate (CEP). The chondrocyte subclusters were classified based on their potential regulatory, homeostatic, and effector functions in extracellular matrix (ECM) homeostasis. Notably, in the NP, a PROCR^+^ resident progenitor population showed enriched colony-forming unit-fibroblast (CFU-F) activity and trilineage differentiation capacity. Finally, intercellular crosstalk based on signaling network analysis uncovered that the PDGF and TGF-β cascades are important cues in the NP microenvironment. In conclusion, a single-cell transcriptomic atlas that resolves spatially regulated cellular heterogeneity together with the critical signaling that underlies homeostasis will help to establish new therapeutic strategies for IVD degeneration in the clinic.

## Introduction

Degenerative disc disease (DDD) is regarded as the primary cause of low back pain, resulting in a global healthcare burden and significant socioeconomic costs.^1^ It may lead to a severe impact on the quality of life of patients.^2^ The current treatment of DDD, mainly including bed rest, rehabilitation, medication, interventional therapy, and surgery,^3^ provides only symptomatic relief but fails to reestablish the homeostasis of the intervertebral disc (IVD).^4^ Furthermore, the deterioration of the health of the compromised spine cannot be prevented.^5^ Thus, the unrelenting threat posed by DDD to human health has motivated the search for an increased understanding of human IVD physiology and pathology.

The IVD has a well-confined structure, including three components: the central hydrated nucleus pulposus (NP), the surrounding lamellar annulus fibrosus (AF), and the cartilage endplate (CEP) that is adjoining to the vertebra.^6^ The confined structure of the IVD plays a part in the mechanical function.^7^ Unfortunately, alterations in the cellular composition and microenvironment cause the IVD to undergo a slow but relentless program that causes the confined structure to be compromised during the degenerative process.^8–10^ The origin of the IVD is heterologous, where the NP is believed to be derived from the notochord,^11,12^ and the AF and CEP are derived from the sclerotome.^13,14^ Consequently, the cells in the IVD are also heterogeneous, composed of NP cells, and notochord cells in the NP, AF cells in the AF, and chondrocytes in the CEPs.^15^ However, classification based on spatial location cannot uncover the highly heterogeneous cell populations in regard to phenotype and function. Although previous studies have revealed phenotypes of IVD cells by bulk RNA sequencing,^16–18^ the search for molecular mechanisms underlying degeneration has been complicated by the large amount of heterogeneity in cellular compositions and the subsequently highly complex cellular microenvironment of the IVD. To further examine the cellular heterogeneity, some efforts were made to distinguish the critical cell types in IVD. The hypothesis regarding cellular heterogeneity in the IVD was initially supported by Hunter CJ et al., as evidenced by the existence of large vacuolar notochordal cells in the NP and small rounded chondrocytes.^12,19^ Notochordal cells are thought to disappear starting in adolescence in the human IVD,^20,21^ which has been questioned because *brachyury (TBXT)*, a notochord lineage marker, continued to be expressed in the IVD.^22^ Thus, notochord cells are thought to be the precursors of all NP cells regardless of variations in morphology and size at different stages.^23^ In addition, mesenchymal stem cells (MSCs) are thought to exist in the IVD due to the expression of the MSC markers *ENG* (CD105), *CD44*, *THY1* (CD90), *NT5E* (CD73), and *NGFR* (CD271).^24,25^ NP progenitor cells are characterized by clonogenicity, pluripotency, and NP reorganization properties.^26^ However, the different lineages remain largely unknown due to the lack of high-precision and unbiased resolution for distinguishing cell populations in the human IVD, although its importance is widely acknowledged.

Single-cell RNA sequencing (scRNA-seq) is considered as a powerful tool for resolving cellular heterogeneity and hierarchical factors forming a complicated cell niche.^27,28^ Here, we performed scRNA-seq to obtain an unbiased picture of IVD cell populations. Our findings provide a better understanding of the inherent heterogeneity and reshape the existing classifications of chondrocytes in the IVD. Notably, we also confirmed the existence of progenitor cells in the IVD marked by *PDGFRA* and *PROCR*. Thus, our study reveals the cellular landscape of the human IVD and provides insights that could help to identify therapeutic targets for human DDD.

## Results

### Comprehensive scRNA-seq analyses resolve the major cell types in the human IVD

**Fig. 1:**
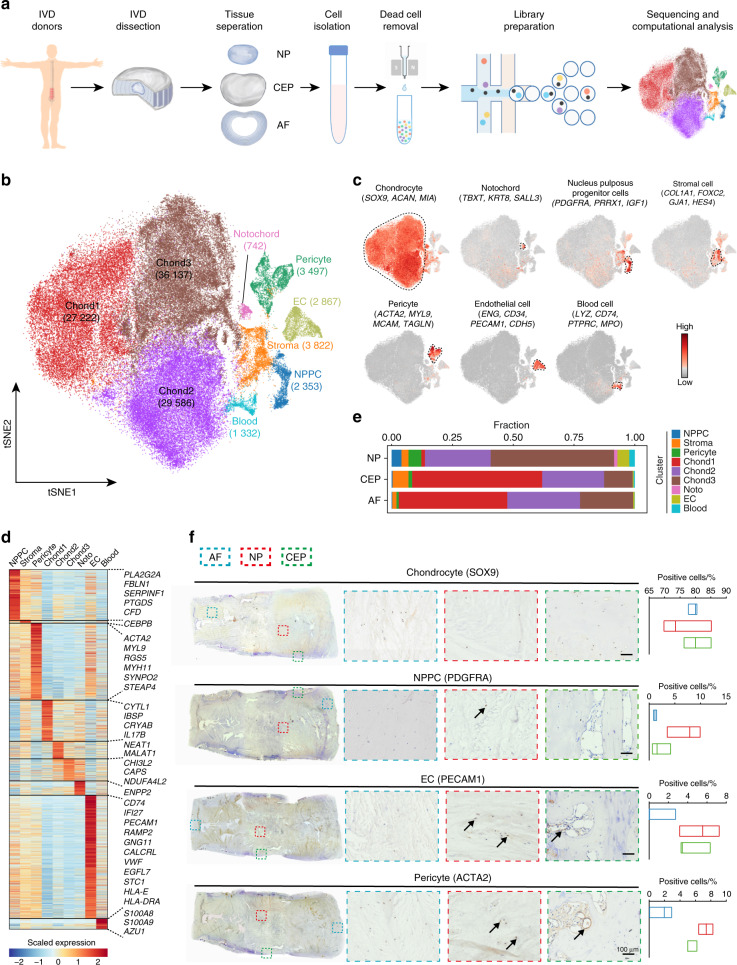
Single-cell transcriptomic landscape of human intervertebral disc (IVD) cells. **a** Schematic workflow of the experimental strategy. Cells isolated from the NP, AF, and CEP of the human IVD were subjected to droplet-based scRNA-seq. NP nucleus pulposus, CEP cartilage endplate, AF annulus fibrosus, IVD intervertebral disc, scRNA-seq single-cell RNA sequencing. **b** Distribution of 108 108 cells from human intervertebral disks. Eight cell clusters were visualized by a tSNE plot. Cell numbers for each cluster are indicated in brackets. NPPC nucleus pulposus progenitor cell, EC endothelial cell, Chond chondrocyte, tSNE t-distributed stochastic neighbor embedding. **c** The average expression of curated feature genes for cell clusters defined in **b** on the tSNE map. **d** Heatmap revealing the scaled expression of DEGs for each cell cluster. DEGs differentially expressed genes. **e** Fraction of cell clusters in the NP, CEP, and AF. **f** Representative immunohistochemistry staining of signature markers of the indicated cell clusters in the AF, NP, and CEP of healthy human IVD tissues and quantification of positive cells displayed with a box plot (*n* = 3). Scale bar, 100 μm

To determine the cellular composition of the human IVD, we employed droplet-based single-cell transcriptomic profiling (10X Genomics Chromium System) of cells from the NP, AF, and CEP from five healthy human IVDs (Pfirrmann I) (Fig. 1a and Supplementary Table 1), as evaluated by magnetic resonance imaging (MRI) according to the Pfirrmann grading system^29^ (Supplementary Fig. 1a). The integrity of the IVD was confirmed because the sagittal cross-section showed that it met the criteria of high hydration and ordered organization with increased deposition of chondroitin sulfate based on hematoxylin & eosin and safranin O/fast green staining (Supplementary Fig. 1a). Because it was difficult to distinguish the boundary between the NP and inner AF, we harvested gelatinous tissue from the central region as the NP. Thus, the tissue origins of harvested cells were identified clearly due to the strict criteria of sampling. A total of 128 833 individual human IVD cells were profiled, and 108 108 cells were retained for subsequent analysis after rigorous quality control and doublet exclusion (Supplementary Table 1). The resulting cells were sequenced to a median depth of 5 367 unique molecular identifiers (UMIs) per cell, with a median of 1 569 genes detected per cell (Supplementary Fig. 1b and Supplementary Table 1). Similarities between samples determined by Pearson’s correlations and the sequencing depth suggested that all samples were comparable (Supplementary Fig. 1c).

We performed fastMNN^30^ to correct batch effects among different data sets. Unbiased clustering based on t-distributed stochastic neighbor embedding (tSNE) identified nine putative root clusters in the healthy human IVD (Fig. 1b and Supplementary Fig. 1d), including (1–3) three clusters of *SOX9*^+^ chondrocytes (Chond1, Chond2, and Chond3); (4) notochord cells; (5) stromal cells; (6) pericytes; (7) endothelial cells (ECs); (8) nucleus pulposus progenitor cells (NPPCs); and (9) blood cells. The chondrogenic marker gene *SOX9*^+^ and chondrocyte-specific ECM genes (*COL2A1* and *ACAN*) were ubiquitously expressed in the three chondrocyte clusters (Fig. 1c, d). The notochord origin marker gene, *TBXT*,^31^ was dominantly expressed in the notochord cell cluster, along with notochord-derived cytokeratin genes, such as *KRT8*^32^ (Fig. 1c, d). *FOXC2*, *GJA1*, and *HES4*, which are essential for stromal cell differentiation,^33–35^ that were mainly expressed in the stromal cell cluster. Pericyte and EC clusters were identified by feature gene expression (*ACTA2*, *TAGLN*, and *MCAM* for pericytes^36–38^ and *PECAM1*, *CD34*, *CDH5*, *ERG*, and *VWF* for EC^39–42^) (Fig. 1c, d and Supplementary Table 2). We found that *PDGFRA* (the mesenchymal progenitor marker),^43^ *PRRX1* (which is restricted to the mesodermal origin and regulator of mesenchymal precursors)^44^ and *IGF1* (a growth factor that effectively differentiates MSCs into NP-like cells)^45^ were specifically expressed in the NPPC clusters (Fig. 1c, d). Thus, we speculated that *PDGFRA*^+^ NPPCs could be a mesoderm-derived progenitor cell cluster in the IVD. A total of 2 651 differentially expressed genes (DEGs) were identified that distinguished human IVD cell populations (Fig. 1d and Supplementary Table 2). Spatially, chondrocytes and stromal cells were abundant in the NP, CEP, and AF, while notochord cells were mainly found in the NP (Fig. 1e and Supplementary Fig. 1e). The expression of some widely reported marker genes of the IVD was also detected in these cell populations (Supplementary Fig. 1f). We then performed an immunohistochemistry assay to validate the spatial distribution of major cell types (Fig. 1f). We found that most SOX9^+^ chondrocytes were detected in the NP, AF, and CEP, as expected. PDGFRA^+^ NPPCs were mainly distributed in the NP and rarely found in the AF and CEP. ACTA2^+^ pericytes and PECAM1^+^ ECs were sporadically distributed in the NP and were present in the tube-like CEP, in line with previous findings on capillaries in the CEP.^46^ Moreover, immunofluorescence staining of the human IVD (Pfirrmann I and II) validated the presence of scattered PECAM1^+^CD34^+^ cells and ACTA2^+^ cells in the IVD (Supplementary Fig. 1g).

Pairwise correlation analysis clearly distinguished the chondrocyte and nonchondrocyte subsets (Supplementary Fig. 1h). Gene ontology (GO) analysis revealed distinct functional enrichment in these cell types (Supplementary Fig. 1i). For example, Chond1 was enriched for signaling regulation and stimulus-response, while Chond2 was enriched for ECM synthesis and organization. As expected, pericyte and ECs were enriched for genes involved in regulating vasculature development, cell adhesion, and junctions. Interestingly, the NPPC cluster was enriched for terms that regulated skeletal development and ossification.

To validate the conserved cell heterogeneity of the IVD across species, we compared the transcriptome of IVD cells between humans and rats by reanalyzing the scRNA-seq data from a recent rat study.^47^ As expected, most of the cell clusters identified in the human IVD were also found in the rat IVD and showed gene expression conservation across cell types, including NPPCs, ECs, and pericytes (Supplementary Fig. 2a, b). In particular, NPPCs in rats also highly expressed *PDGFRA*, *PRRX1*, and *IGF1* and shared distinct gene expression patterns with their counterparts in humans (Supplementary Fig. 2c, d).

Overall, these results revealed the cellular diversity in the human IVD, and we identified a set of markers that can potentially be used to recognize the cell clusters in the human IVD.

### The functional definition of chondrocyte subpopulations in the IVD

**Fig. 2:**
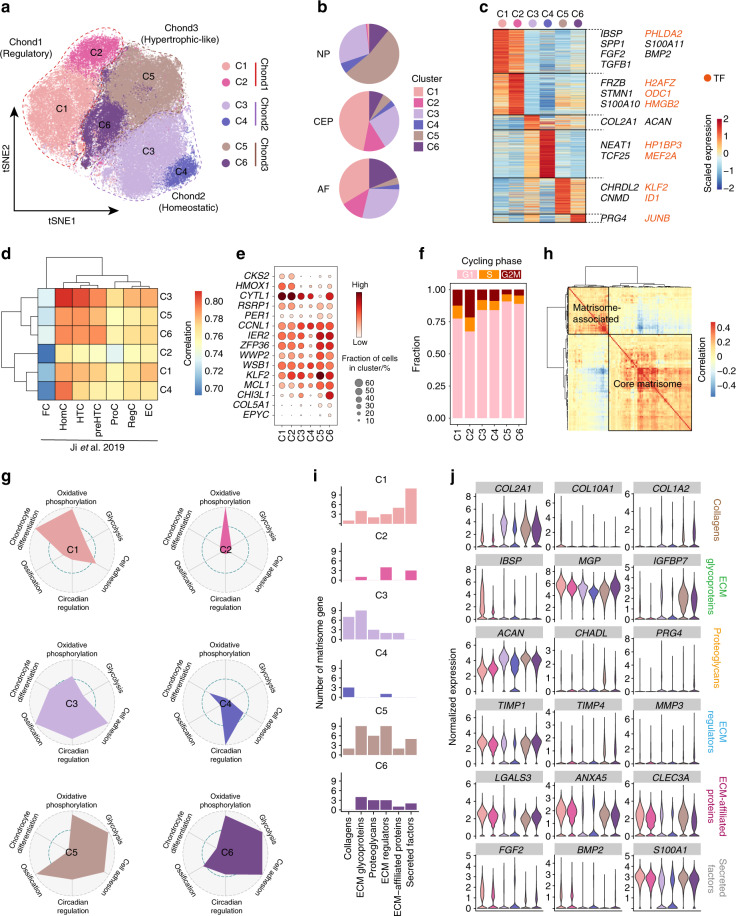
Characterization of chondrocytes in the human IVD. **a** tSNE plot of the six subclusters of 93 495 chondrocytes defined in the IVD. **b** Fraction of each chondrocyte subcluster in the NP, CEP, and AF. **c** Heatmap revealing the scaled expression of DEGs for each chondrocyte subcluster. **d** Heatmap showing pairwise Pearson correlations in the global transcriptome between IVD chondrocytes and articular chondrocytes (Ji et al.,^51^). FC fibrocartilage chondrocyte, HomC homeostatic chondrocyte, HTC hypertrophic chondrocyte, preHTC prehypertrophic chondrocyte, ProC proliferative chondrocyte, RegC regulatory chondrocyte, EC effector chondrocyte. **e** Dot plot showing the mean expression of selected chondrocyte function-associated genes among the six chondrocyte subclusters. Dot size indicates the percentage of cells in subclusters with detected expression. **f** The fraction of each chondrocyte subcluster arrested in the different cell-cycle phases. **g** Radar map showing the performance of six gene sets associated with the indicated function and metabolic pathway among each chondrocyte subcluster. **h** Heatmap showing pairwise Pearson correlations of expressed matrisome genes in chondrocytes. Two signature patterns (matrisome-associated and core matrisome) were identified by hierarchical clustering. **i** The number of expressed genes associated with six matrisome patterns in each chondrocyte subcluster. ECM extracellular matrix. **j** Violin plots showing the expression levels of representative genes associated with six matrisome patterns in each chondrocyte subcluster

As chondrocytes are known to play a pivotal role in ECM homeostasis and the degeneration of the IVD,^48^ we sought to determine their composition. Each of the three chondrocyte clusters was divided into two subclusters (Fig. 2a). The distribution of subclusters exhibited apparent distinctions in the three compartments of the IVD (Fig. 2b). The subclusters of C1 and C2 were mostly located in the AF and CEP, while C5 was mainly located in the NP. Subclusters of C3, C4, and C6 were relatively evenly distributed in the NP, AF, and CEP. A total of 912 DEGs were found among the six chondrocyte subclusters (Fig. 2c and Supplementary Table 3). We found that C1 preferentially expressed growth factor (GF) genes such as *BMP2, TGFB1*, and *FGF2*. Subclusters C3 and C4 preferentially expressed the genes of the main ECM components of the IVD, such as *ACAN* and *COL2A1*. Subclusters C5 and C6 preferentially expressed *PRG4* and *CNMD*, suggesting that they may play a protective role and stabilize the chondrocyte phenotype.^49,50^

To better understand the specific characteristics of IVD chondrocytes, we compared the transcriptomic differences between these chondrocyte subclusters and articular cartilage chondrocytes at different stages of osteoarthritis (stages 0-4) (Fig. 2d).^51^ There was no characteristic correspondence among subclusters C1, C2, and articular chondrocytes. We found that subclusters C1 and C2 shared some DEGs with articular regulatory chondrocytes (RegCs), including *CKS2* and *HMOX1* (Fig. 2e), and highly expressed *IBSP and CYTL1*, which are NP-negative biomarkers (Supplementary Table 3).^17,52^ Moreover, subclusters C1 and C2 showed a higher percentage of cells arrested in the G2/M phase than that in others, which was indicative of relatively higher proliferative activity (Fig. 2f). We also evaluated these subclusters using a gene set related to chondrocyte function (Fig. 2g). The results showed that oxidative phosphorylation played a role in the metabolic pattern of C1 and C2, which could be explained by the fact that C1 and C2 were mainly located in the vascularized AF and CEP. Notably, subcluster C1 was enriched for genes related to chondrogenic differentiation. Therefore, we hypothesized that subclusters C1 and C2 represent regulatory chondrocytes that stimulated surrounding cells by secreting GFs.

Pairwise correlation analysis revealed close relationships among C3, C4, and homeostatic chondrocytes (HomCs, Fig. 2d) with a similar pattern of gene expression as articular HomCs, such as *CCNL1* and *WSB1* (Fig. 2e).^51^ In contrast with regulatory chondrocytes C1 and C2, fewer cells in C3 and C4 were arrested in the G2/M phase (Fig. 2f). In particular, both C3 and C4 exhibited strong enrichment of circadian regulation genes and moderate enrichment of chondrogenic differentiation (Fig. 2g). C3 was also enriched for cellular adhesion genes, which are critical for forming chondrocyte clonal columns within an ordered, three-dimensional cell array.^53^ Considering that they preferentially expressed ECM-related genes, we chose to classify C3 and C4 as homeostatic chondrocytes, which function in maintaining ECM homeostasis and circadian rhythm.

The C6 subcluster was relatively similar to hypertrophic chondrocytes (HTCs) and prehypertrophic chondrocytes (preHTCs, Fig. 2d). *COL5A1* and *EPYC* were expressed in the subclusters of C5 and C6 (Fig. 2e), similar to articular HTCs and preHTCs.^51^ We also found that C5 and C6 highly expressed genes reflecting protective characteristics (*KLF2* and *CHI3L1*) (Fig. 2e)^54,55^ and existed in the dormant stage of proliferation (Fig. 2f). Interestingly, subclusters C5 and C6 preferentially performed metabolic processes, including oxidative phosphorylation and glycolysis, showing the traits of a high metabolism (Fig. 2g) and the characteristics of articular effector chondrocytes.^51^ Unlike the resident quiescent chondrocytes with low metabolism,^56^ these subclusters were possibly adapted to anaerobic metabolism because C5 was mainly located in the NP, which has an avascular and hypoxic microenvironment, consistent with previous study showing that the NP that is predominantly glycolytic due to vigorous *HIF1* activity.^57^ Collectively, we inferred that C5 and C6 were effector chondrocytes with high metabolic rates and protective/repair functions.

To reveal the core function of chondrocytes in modulating ECM homeostasis, we detected the expression of matrisome-related genes. Matrisome genes were categorized into the core matrisome (collagens, proteoglycans, and ECM glycoproteins) and matrisome-associated (ECM regulators, ECM affiliation, and secreted factors) according to a matrisome classification database (matrisomeproject.mit.edu).^58^ We first evaluated the average expression of six modules in eight clusters (Supplementary Fig. 3a) and compared the expression of matrisome genes distinctly expressed in the NP, CEP, and AF (Supplementary Fig. 3b and Supplementary Table 4). Correlation analysis of matrisome-related genes in chondrocytes revealed two patterns: the core matrisome and matrisome-associated (Fig. 2h). To clarify the primary function of matrisome-related gene subsets in six chondrocyte subclusters, we compared the expression abundance of these genes (Fig. 2i, j). We found that secreted factors were predominantly expressed in the regulatory C1 subset, while the homeostatic C3 subset preferentially expressed genes of the core matrisome (Fig. 2i, j). In contrast, the effector C5 subset exhibited high expression of ECM regulators, reflecting its regulatory role in ECM homeostasis (Fig. 2i, j).

Taken together, these data add to the knowledge on the functions of chondrocyte subclusters in human IVD.

### Delineating nucleus pulposus progenitor cells and their signature genes

**Fig. 3:**
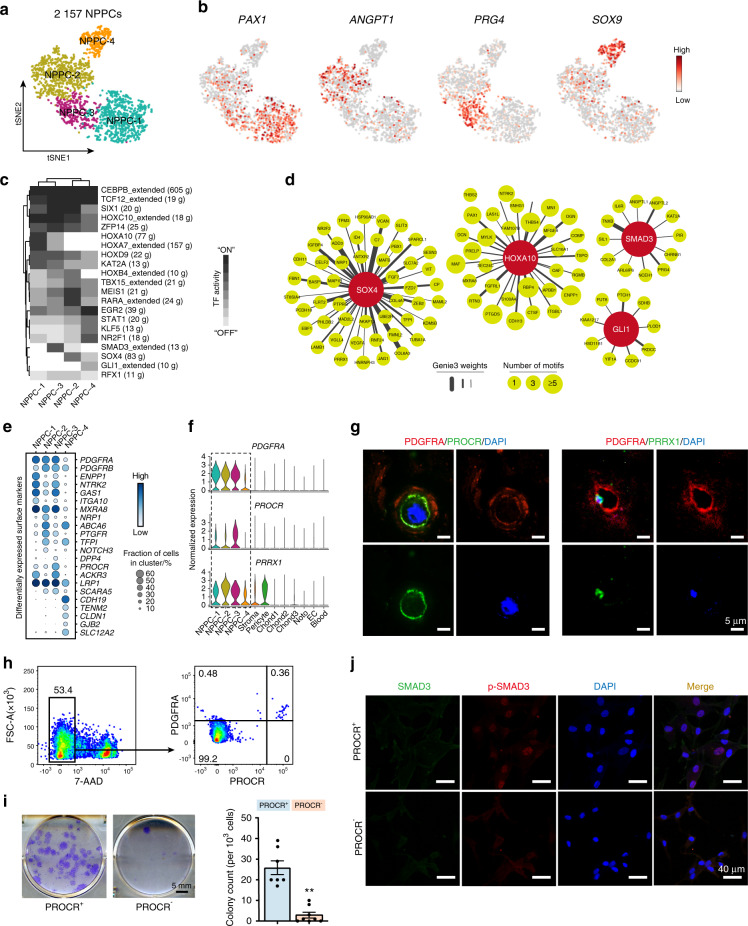
Characterization of NPPC in human IVD. **a** Four subclusters of 2 157 NPPCs were visualized by a tSNE plot. **b** tSNE plot of signature gene expression in NPPCs. **c** Heatmap revealing binary regulon activities analyzed with SCENIC in each subcluster of NPPCs. “ON” indicates active regulons; “OFF” indicates inactive regulons. SCENIC Single-Cell Regulatory Network Inference and Clustering. **d** The HOXA10, SOX4, SMAD3, and GLI1 regulon networks in NPPC subclusters. The TFs are in dark red, and the corresponding target genes are in light green. The line thickness indicates the level of GENIE3 weights. The dot size indicates the number of enriched TF motifs. TFs transcription factors, GENIE3 GEne Network Inference with Ensemble of trees. **e** Dot plot showing differentially expressed genes encoding surface markers in each subcluster of NPPCs. **f** Violin plots showing the expression levels of the signature genes of NPPC-3 in the IVD. **g** Immunofluorescence staining showing the coexpression of PDGFRA, PROCR, and PRRX1 in human IVD cells in situ (*n* = 3). Scale bar, 5 μm. **h** Flow cytometry gating strategies for sorting PDGFRA^+^PROCR^+^ in the human IVD. **i** Representative crystal violet staining of CFU-F colonies generated by sorted primary PROCR^+^ cells of the human IVD (left, *n* = 3). Scale bar, 5 mm. Quantification of the number of CFU-F colonies (right). The statistical significance of differences was determined using one-way ANOVA with multiple comparison tests (LSD). **P < 0.000 1. Error bars indicate the SEM. CFU-F colony-forming unit-fibroblast, ANOVA Analysis of Variance, LSD least significant difference, SEM Standard Error of the Mean. **j** Immunofluorescence staining of SMAD3 and p-SMAD3 in the PROCR^+^ and PROCR^−^ cells of the human IVD (*n* = 3). Scale bar, 40 μm

NP progenitor/stem cells are critical in the physiological and pathological processes of the IVD.^59,60^ We identified NPPC-enriched genes related to bone development, bone morphogenesis, connective tissue development, and endochondral bone growth (Supplementary Fig. 1i). To better understand the role of the NPPC cluster, we sought to determine their composition in the human IVD. We partitioned NPPCs into four subclusters (Fig. 3a). The localization of the discogenic marker *PAX1* confirmed the physical presence of the NPPC-1 subcluster. *PAX1* is expressed in the sclerotome, which is critical for the formation of vertebrae and IVDs,^61^ indicating the potential role of discogenic differentiation in NPPCs. Subcluster NPPC-2 specifically expressed *ANGPT1*, which is critical for the survival of nucleus pulposus cells.^26^ *PRG4*, the signature gene of NPPC-3, was also highly expressed in articular cartilage progenitor cells.^62^ *SOX9* expression indicated the chondrogenic priming of NPPC-4 (Fig. 3b). These NPPC subclusters were also distinguished by the indicated DEGs (Supplementary Fig. 4a and Supplementary Table 5). GO analysis of these DEGs showed that NPPC-1 and NPPC-3 were enriched for genes regulating ECM organization, while NPPC-4 was enriched for genes involved in mRNA catabolic metabolism (Supplementary Fig. 4b). Gene set enrichment analysis (GSEA) showed that NPPC-1 was enriched for the calcium signaling pathway, which played a vital role in modulating NP homeostasis by regulating *AQP2*.^63^ NPPC-2 was enriched for the MAPK signaling pathway, potentially playing a protective role in cell survival in the NP.^64^ NPPC-3 preferentially expressed the *SMAD2/3* pathway, and NPPC-4 was enriched for *NOTCH* signaling, which plays a role in cell growth (Supplementary Fig. 4c).^65^

To explore the regulatory networks that determine cell fate specification in the NPPC subclusters, we utilized single-cell regulatory network inference and clustering (SCENIC) to infer the regulatory activity (regulon) from the coexpression of transcription factors (TFs) and their downstream target genes.^66^ We filtered 21 core regulons out of 227 regulons that were used to discriminate the four NPPC clusters (Fig. 3c, Supplementary Fig. 4d, and Supplementary Table 6). The highly enriched regulons in NPPC-1 included *HOXA10* and *HOXA7*. The *SOX4*, *RARA*, and *MEIS1* regulons were specific to NPPC-2. NPPC-3 exhibited strong enrichment of *ZFP14* and *SMAD3*. NPPC-4 was enriched for regulons such as *GLI1*, *EGR2*, and *NR2F1* (Fig. 3c and Supplementary Table 6). Some important regulons, including *HOXA10*, *SOX4*, *SMAD3*, and *GLI1*, together with their downstream target genes, such as the abovementioned *PAX1*, *PRG4*, and *ANGPT*, had the potential to regulate the function of NPPCs (Fig. 3d and Supplementary Table 5). Specifically, *HOXA10* is a critical regulator of osteogenesis.^67^ *SOX4* is highly expressed in osteoblast progenitors, and its expression is increased during osteoblast differentiation.^68^ *GLI1* marks mesenchymal progenitors responsible for bone formation and fracture repair and regulates chondrocyte differentiation.^69^ *SMAD3*, the downstream target of *TGF-β*, plays a dominant role in chondrogenesis and maintaining the phenotype of chondrocytes.^70^

To immunophenotype these NPPC subclusters, we screened for cell surface marker genes that were differentially expressed among the four NPPC subclusters. Among them, *PDGFRA* showed higher expression in NPPC-1, NPPC-2, and NPPC-3 than in NPPC-4. Interestingly, we found that NPPC-3 preferentially expressed *PROCR* (Fig. 3e), a widely reported signature gene for progenitor cells in multiple organs, including the hematopoietic and vascular systems,^71–74^ pancreas,^75^ ovaries,^76^ etc. Thus, the specific expression of *PROCR* suggested the potential stemness capacity of NPPC-3. We then combined the expression of the membranous marker genes *PDGFRA* and *PROCR* and the transcription factor *PRRX1* as a signature for the identification of NPPC-3 (Fig. 3f and Supplementary Fig. 4e) and performed immunofluorescence staining to examine their coexpression (Fig. 3g). Immunostaining of a healthy IVD (Pfirrmann I, Supplementary Table 1) showed that PDGFRA^+^PROCR^+^ NPPCs were mainly located in the NP zone (Supplementary Fig. 4f). To assess the proportion of PDGFRA^+^PROCR^+^ NPPCs in the NP, primary PDGFRA^+^PROCR^+^ cells were flow cytometrically sorted from the human IVD (Pfirrmann II, Supplementary Table 1). The results showed that the frequency of PDGFRA^+^PROCR^+^ cells was 0.36% (Fig. 3h), and PDGFRA was enriched in almost all PROCR^+^ cells in the IVD. To test the clonogenicity of NPPC-3, primary PROCR^+^ cells were sorted by flow cytometry for a colony-forming unit-fibroblast (CFU-F) colony formation assay. The counts of typical colonies derived from primary PROCR^+^ cells were 25.9 ± 3.3 per 1 000 cells, which was comparable to that for PDGFRA^+^ MSCs^77^ and significantly higher than that for PROCR^−^ cells (2.9 ± 1.4 per 1 000 cells) (Fig. 3i), indicating that NPPCs exhibited enhanced colony-formation ability. To verify the in silico finding of the enriched regulatory activity of SMAD3 in PROCR^+^ NPPCs, we detected the expression level of p-SMAD3 in P2 PROCR^+^ and PROCR^−^ cells from the human IVD (Pfirrmann II, Supplementary Table 1). As expected, the expression of p-SMAD3 in the nucleus was higher in PROCR^+^ cells than in PROCR^−^ cells (Fig. 3j). These results indicated that SMAD3 was highly activated in PROCR^+^ cells, suggesting the potential role of PROCR^+^ cells in the chondrogenesis of IVD.

Taken together, these data elucidated the cellular heterogeneity in NPPCs, which was highly regulated and comprised the population with clonogenicity that could be enriched by PDGFRA and PROCR.

### Reconstruction of the bilineage trajectory of PDGFRA^+^PROCR^+^ NPPCs

Connective tissue comprised stromal cells with phenotypic and functional complexity,^78^ which provided support during NP development and repair.^79^ We collected 1 372 stromal cells from the NP that were divided into six subclusters (Supplementary Fig. 5a and Supplementary Table 7), including three subclusters of fibroblasts (Fib1, Fib2, and Fib3) that expressed high levels of fibroblast signature genes, such as *CEMIP*, *AKR1C1*, *MGP*, *COMP*, *DNER*, and *MELTF*,^80,81^ two subclusters of neurogenic cells (Neu1 and Neu2) with high expression of the neurogenic markers *SOX2*, *NGFR*, *NCMAP*, and *CLDN19*,^82–85^ and osteogenic cells that expressed high levels of the osteogenic regulators *RUNX2* and *DLX5* (Supplementary Fig. 5b).^86–88^ To further verify the existence of the cell clusters in the IVD, immunofluorescence staining of human IVDs (Pfirrmann I and II) showed a few RUNX^+^SP7^+^ cells and SOX2^+^ cells in the IVD (Supplementary Fig. 5c), consistent with the findings from the scRNA-seq analysis.

**Fig. 4:**
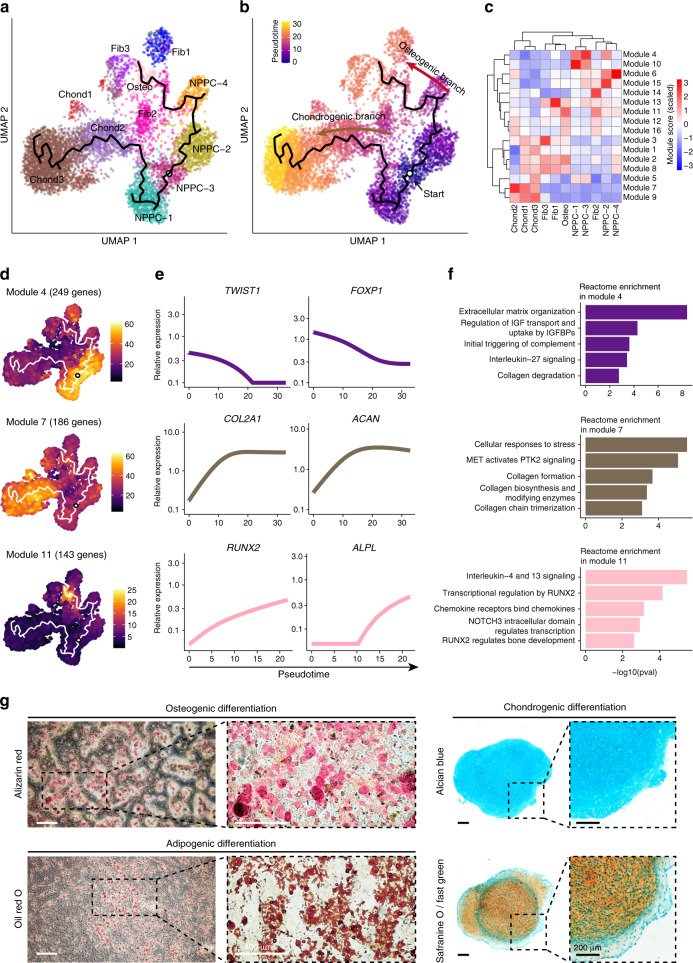
Reconstruction of bilineage trajectories in NP cells. **a** UMAP visualization of NPPC, chondrocyte, fibroblast, and osteogenic subclusters. The principal graph of trajectories reported by Monocle 3 was rooted in NPPC-3, as indicated by a cycle. UMAP, Uniform manifold approximation and projection. **b** Developmental pseudotime for cells present along the trajectory inferred by Monocle 3, with osteogenic and chondrogenic branches coming from NPPC subclusters. **c** Heatmap showing the scaled mean expression of modules of coregulated genes grouped by Louvain community analysis across the subclusters. **d** UMAP plots showing the relative expression level of representative gene modules in NP cell subclusters. **e** Pseudotime kinetics of the indicated genes in the modules in **d** from the root along the trajectories to chondrogenic and osteogenic differentiation. **f** Histogram showing the pathways enriched by ReactomePA for each module indicated in **d**. **g** Representative alizarin red (top left), oil red O (bottom left), alcian blue (top right), and safranine O/fast green (bottom right) staining after the osteogenic, adipogenic, and chondrogenic differentiation of PROCR^+^ cells (*n* = 3). Magnified images of the boxed areas are shown on the right. White scale bars, 400 μm. Black scale bars, 200 μm

We next sought to investigate the differentiation trajectories that determined the cellular hierarchy in NP cells. All NP cells, including four subclusters of NPPCs, three subclusters of fibroblasts, three subclusters of chondrocytes, and osteogenic cells, were involved in reconstructing the differentiation trajectories using Monocle 3 (Fig. 4a), an algorithm for the reconstruction of lineage programs based on similarity at the transcriptional level.^89^ We set NPPC-3 as the starting point of the differentiation trajectories due to its high expression of pluripotent genes and progenitor potential identified above, and then computed pseudotime for cells along the inferred developmental axis (Fig. 4a, b). More specifically, NPPC-3 was predicted to differentiate into two distinct cell lineages, including the chondrogenic branch, which includes NPPC-3, NPPC-1, Chond2, and Chond3, and the osteogenic branch, which includes NPPC-3, NPPC-2, NPPC-4, osteogenic cells, and Fib3 (Fig. 4a, b).

To explore gene expression dynamics along the trajectories, we grouped genes that varied between cell clusters into 16 modules using Louvain community analysis (Supplementary Table 8). A heatmap showed the aggregated expression in each module across cell clusters (Fig. 4c). We found that the expression of genes in module 4 was downregulated along both trajectories, such as the differential regulator genes *TWIST1* and *FOXP1*,^90,91^ which were enriched for genes related to ECM organization (Fig. 4d–f). In contrast, the expression of chondrogenic genes was gradually elevated along the chondrogenic trajectory, such as *COL2A1* and *ACAN* in module 7, and remained at high expression levels until terminal differentiation (Fig. 4d, e), which was also evidenced by the expression of module 9 (e.g., the chondrogenic *CNMD and FGFBP2*) (Supplementary Fig. 5d, e). The expression of the osteogenic gene set was elevated along the osteogenic trajectory, such as *RUNX2* and *ALPL* in module 11 (Fig. 4d, e) and the expression of *SP7*, *BGLAP*, and *MMP11* in module 16 (Supplementary Fig. 5d, e).

According to the prediction of the bifurcating differentiation trajectories of NPPC-3, we tested the trilineage differentiation of PROCR^+^ cells (cells that were expanded from CFU-F colonies) ex vivo and found that they efficiently underwent osteogenic, chondrogenic, and adipogenic differentiation (Fig. 4g).

Taken together, these data depicted the trajectories of NP cells, in which PROCR^+^ cells were enriched for multipotent NPPCs that generate three lineages, consequently revealing the successive activation of transcriptional programs in NP homeostasis.

### Putative signaling network for the intercellular crosstalk regulating the homeostasis of the NP

**Fig. 5:**
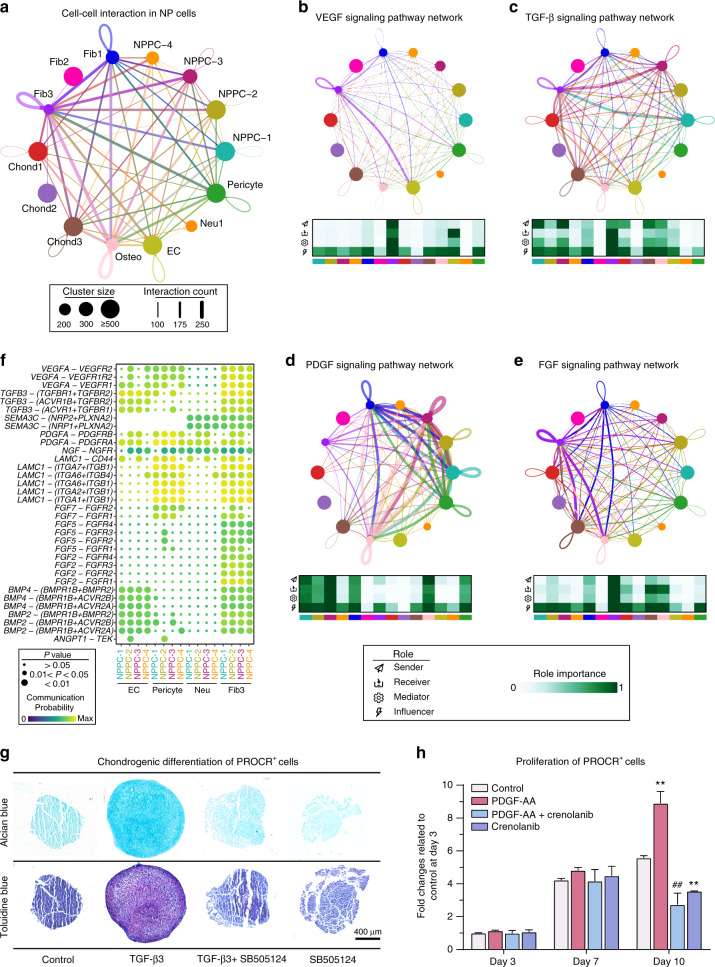
Overview of the crosstalk networks among the clusters in the NP. **a** Overview of the cellular network regulating the homeostasis of the NP. Dots indicate cell clusters. The dot size indicates the relative quantity of each cluster. The thickness of the directed line indicates the relative quantity of significant ligand-receptor pairs between any two pairs of cell clusters. **b-e** Circle plots showing the inferred VEGF (**b**), TGF-β (**c**), PDGF (**d**), and FGF (**e**) signaling networks. **f** Dot plot showing the communication probability of the indicated ligand-receptor pairs between EC, Pericyte, Neu, and Fib3 subclusters (sending signals) and four NPPC subclusters (accepting signals). **g** Representative alcian blue and toluidine blue staining for the chondrogenic effect of TGF-β3 supplementation (10 ng·mL^−1^) for 28 days on PROCR^+^ cells from the human IVD. Scale bars, 400 μm. **h** Histogram showing the proliferation of PDGF-AA (20 ng·mL^−1^) on PROCR^+^ cells from the human IVD detected by a CCK-8 kit (*n* = 3). The statistical significance of differences was determined using one-way ANOVA with multiple comparison tests (LSD). **P < 0.01 compared with the control group at day 10. ^##^*P* < 0.01 compared with the PDGF-AA group at day 10. Error bars indicate the SEM

To seek further insights into the critical factors involved in the NP cell niche of the human IVD, we investigated the signaling network among the main cell types in the NP. CellChat analysis of these 14 subclusters in the NP identified the signaling network for intercellular crosstalk. Relative active bidirectional signaling interactions among these cell subclusters revealed highly regulated cellular communications (Fig. 5a and Supplementary Table 9). ECs, pericytes, fibroblasts, and neurogenic cells identified as niche components in the NP played distinct roles in signaling interactions to regulate the differential process. To determine the important factors, we further analyzed the intercellular signaling networks of VEGF, TGFB, PDGF, and FGF (Fig. 5b–e).

Interestingly, Fib3 was involved in VEGF signaling, both autocrine and paracrine (Fig. 5b, f). ECs were the leading receiver of VEGF signals, as expected, and NPPC subclusters functioned as regulators of the communication (Fig. 5b). Moreover, the TGF-β pathway was involved in many signaling interactions among chondrocyte subclusters and NPPC clusters via *TGFB3-TGFBR* or *TGFB3-ACVR1* (Fig. 5c, f). As shown above, NPPC-3 was enriched for *SMAD3*, the key downstream target of TGF-β, which prompted us to further investigate the role of TGF-β3 in chondrogenesis in NPPCs. The results showed that 10 ng·mL^−1^ TGF-β3 effectively induced chondrogenesis and the formation of dense cartilage extracellular matrix (ECM) compared with that in the negative control group after 28 days of differentiation. However, supplementation with 10 μmol·L^−1^ SB505124, a TGF-β receptor inhibitor, blocked the chondrogenesis of PROCR^+^ cells both with and without TGF-β3 (Fig. 5g). The results demonstrated that the TGF-β family plays an important role in the chondrogenic regulation of PROCR^+^ cells.

In the PDGF signaling network, the NPPC clusters acted as critical contributors by secreting PDGFA ligand, leading to the paracrine activity of NPPCs to osteogenic cells, pericytes, and Fib1 and the autocrine activity of NPPCs to themselves. Specifically, NPPC-3 was the key population that dominated the PDGF signaling network (Fig. 5d, f). Previous studies have reported that PDGF-AA is involved in the regulation of cell proliferation.^92,93^ Therefore, we explored the effect of PDGF-AA on the proliferation of PROCR^+^ cells from the human IVD (Fig. 5h). The results showed that 20 ng·mL^−1^ PDGF-AA significantly promoted the proliferation of PROCR^+^ cells on the 10th day of expansion, and 100 nmol·L^−1^ crenolanib, a PDGFR α/β inhibitor, significantly inhibited the proliferation of PROCR^+^ cells in the presence or absence of PDGF-AA after treatment for 10 days. The FGF signaling network exhibited intensive exchanges among almost all the cell types with *FGF* ligands that were mainly secreted by Fib3 (Fig. 5e, f).

By comprehensively predicting signaling networks for intercellular crosstalk, large numbers of ligand-receptor pairs participated in ligand-receptor pairs of *VEGF*, *TGFB*, *SEMA3*, *PDGF*, *NGF*, *LAMC*, *FGF*, *BMP*, and *ANGPT* between NPPCs and other cell types (Fig. 5f). Interestingly, Fib3 was involved in almost all the above pathways, suggesting its significance in NP homeostasis. We further revealed that NPPC-1, NPPC-2, NPPC-3, and pericytes sent communications to other cells via *IGF* and *PDGF* (Supplementary Fig. 6a). As expected, Fib3 was exclusively dominated by *FN1* in regard to outgoing communication. In addition, Fib3 and Chond1 received incoming communication by *BMP*, *GDF*, and *ANGPT*, which reportedly played a prominent essential role in the IVD (Supplementary Fig. 6b).^26,94,95^ *EGF*, used by NPPC-2, NPPC-4, and Neu1 for incoming signaling, could be a protective factor in IVD regeneration (Supplementary Fig. 6b).^96^

CellChat analysis of NP, AF, and CEP cells also revealed a large number of signaling networks among cell subclusters from the three substructures of the IVD (Supplementary Fig. 6c). For example, NPPCs interacted with Chond1 from the AF and CEP. In particular, the GAS signaling pathways were intensively regulated between NPPCs and Chond3 from the AF (Supplementary Fig. 6d), possibly protecting the IVD from inflammatory factors.^97^ *SPP1*, an osteogenesis-related factor, was highly involved in the interaction among NPPCs and stromal cells in the CEP (Supplementary Fig. 6d). This was in line with our above hypothesis that osteogenic cells might play a role in IVD homeostasis and/or degenerative processes. However, these proposed signaling pathways should be considered as multiple biological cascades rather than a sole event because the three substructures always work as a whole.

Taken together, these results indicated that there is a complicated relationship among the distinct cell types and described a cellular crosstalk network with a hierarchical signaling pathway that regulates NP homeostasis in a coordinated manner.

## Discussion

The severe threat of DDD to human health prompted us to seek an innovative treatment that reestablishes IVD homeostasis. Inadequate knowledge of IVD physiology, and pathology poses a challenge to the development of novel treatment strategies. Due to the cellular heterogeneity and resulting complex microenvironment in the human IVD, an in-depth understanding of specific markers and their roles in IVD homeostasis is urgently needed. Here, we resolved the cellular diversity at a single-cell level using transcriptomic profiling and identified the cell types with a set of specific markers in the human IVD. We classified IVD chondrocytes into three subtypes based on their potential roles in ECM homeostasis. Notably, we identified new subtypes of progenitor cells with signature genes, spatial distribution in situ, and progenitor potential. Moreover, we analyzed the intercellular crosstalk based on the signaling network and uncovered key factors, such as the PDGF and TGF-β cascades, as important cues for regulating the NP microenvironment. Together with previous studies,^12,98,99^ a better understanding of the cellular heterogeneity of the human IVD is developing, with the aim of contributing to new therapeutic strategies for DDD.

The cellular heterogeneity of IVD cells has been a long-debated controversy due to the complexity of the IVD ontogeny, a tricomponent organization with distinct origins.^100^ Multiple developmental origins lead to the inhomogeneity of the cell composition. Although some scholars have attempted to examine the IVD at the single-cell level, a highly precise and unbiased description of cell populations in the human IVD remains to be elucidated.^52,98^ Previously, notochord cells and chondrocytes were recognized in the NP, which was regarded as the notochordal lineage, evidenced by the constant expression of *TBXT*.^101^ In line with previous findings, we found a minor cluster that expressed high levels of the markers *TBXT* and *KRT8*, which could be a rare but distinct notochord cell cluster. As expected, we found three major clusters of chondrocytes, which are always regarded as core players in ECM homeostasis in the human IVD. Although the expression of *TBXT* was not detected, another notochord marker, *NOG*, was expressed in the majority of chondrocytes (Supplementary Fig. 1f). This interesting finding coincides with a previous theory that distinctive cellular morphology in the NP is due to the various phases along the notochord lineage during aging and degeneration.^5,102–105^

Apart from the leading role of notochord lineage cells, the supporting role of minor cell clusters is more notable because of their unclear function, which has been infrequently reported. First, *SOX2*^*+*^*NGFR*^*+*^ neurogenic cells, one of the stromal subclusters, were also found in the NP (Supplementary Fig. 5a–c). Although the healthy disc was regarded as an aneural tissue,^106^ the pattern of nerve endings has been previously confirmed in healthy and degenerative IVDs,^107–110^ which were small in diameter and relatively sparse.^111^ Thus, sporadic SOX2^+^ neurogenic cells were probably related to neural ingrowth. Furthermore, *RUNX2* played a part in postnatal IVD development and regulated the notochordal transition into chondrocyte-like cells.^112^ Upregulated *RUNX2* expression was also found in the degenerated IVD, which led to IVD calcification.^113,114^ In addition, the stem cells in the IVD exhibited osteogenic potential during ex vivo culture.^25^ These studies may have indicated that the homeostasis of bone formation is important for the physiological and pathological processes of IVD. Our scRNA-seq analysis and immunofluorescence staining revealed the existence of a rare cell cluster that differentially expressed the osteogenic genes *RUNX2*, *DLX5*, and *SP7*,^86–88^ which were defined as osteogenic cells (Supplementary Fig. 5a–c). This finding suggested that osteogenic cells exist in healthy IVDs. We hypothesized that osteogenic cells likely contribute to the homeostasis of the IVD or are involved in the pathological process of early degeneration, which began as early as during the teenage years.^115,116^ Finally, the dynamics of vascularization, represented by ECs and pericytes, play a role in disc homeostasis. Previous studies showed that blood vessels penetrated the AF and CEP during the early postnatal years but regressed later, leaving an avascular microenvironment, which accounted for the poor ability for remodeling and repair in IVDs.^117–119^ However, blood vessels are present in the human IVD until even the third decade of life.^120^ During the slow process of vascular regression, it is reasonable that some remnants are left behind, such as ECs. A recent study reported that cross bridges after vascular regression are indeed present in both healthy and degenerated human disks. The cross-bridges of the IVD stained positively for PECAM1 in adult sheep, although the PECAM1^+^ cross-bridges declined with aging.^121^ In line with scRNA-seq analysis, ACTA2^+^MCAM^+^ pericytes and PECAM1^+^CD34^+^ ECs were scattered in the IVD (Fig. 1b–f and Supplementary Fig. 1g). Our data showed that ECs and pericytes communicated with NPPCs via the VEGF, PDGF, and TGF-β signaling pathways, suggesting that they played a role in NP homeostasis (Fig. 5). Notably, MCAM is regarded as a classical surface marker of pericytes/MSCs.^122^ Previously, periosteal and meniscal MCAM^+^ cells were shown to exhibit canonical features of skeletogenesis,^123,124^ and MCAM^+^ or ACTA2^+^ cells were also detected in the disc.^47,125–127^ Interestingly, MCAM was specifically expressed in the cell population with migration and repopulating potential in degenerative IVDs.^125^ The functional characteristics of these cell types should be investigated in future studies. The highly conserved cellular heterogeneity across cell clusters between human and rat IVDs (Supplementary Fig. 2) suggested that the rat is an ideal animal model to study the role of the above cell clusters in IVD homeostasis.

Cells in the IVD are generally referred to as “chondrocyte-like” cells or “IVD chondrocytes”. Traditionally, chondrocytes in the IVD are classified into NP, AF, and CEP chondrocytes based on their spatial distribution. However, the spatial-based classification of the cell population was insufficient because of the cellular heterogeneity and possible cell migration among the three sites of the IVD.^128^ Thus, the precise roles of IVD chondrocytes in ECM homeostasis are still largely unknown.^16–18,129^ Therefore, a deeper understanding of the roles of IVD chondrocytes in ECM homeostasis is necessary. Taking advantage of the high throughput nature of analysis at the single-cell level with scRNA-seq, we were able to identify six subclusters of IVD chondrocytes with three functional patterns (Fig. 2). First, we identified a new population of regulatory chondrocytes with active GF expression and chondrogenic pathway regulators, implying its regulatory role in chondroid ECM homeostasis. In contrast, homeostatic chondrocytes showed high similarity to classical chondrocytes, which were quiescent, fully differentiated, and responsible for ECM deposition.^130^ Interestingly, homeostatic chondrocytes were enriched in circadian regulation genes, which involved key pathways regulating the homeostasis of IVDs.^131^ This finding suggests that homeostatic chondrocytes could be a potential therapeutic target for circadian rhythm in the human IVD. It is noteworthy that the effector chondrocytes were metabolically active, which is important in maintaining the ECM biogenesis of the IVD.^132^ In addition, the high expression of *PRG4* (lubricin) also implies that they play a protective role in reducing shear stress and inflammation and keeping the joint healthy.^133^ In contrast, effector chondrocytes were characterized by ossification and shared expression patterns with articular HTCs.^51^ Thus, the definitive function of effector chondrocytes is certainly worth future investigation. Overall, the six transcriptomically defined populations of chondrocytes exhibited distinct roles in ECM homeostasis, providing new perspectives for exploring the mechanism of IVD chondrocytes.

The IVD possesses the capability of spontaneous regeneration, as evidenced by self-healing after disc degeneration,^134^ probably due to the presence of in situ progenitor cells. Progenitor cells expressing different marker gene sets existed in three compartments of the IVD.^59,60^ The progenitor cells exhibited certain plasticity and the ability to slow down disc degeneration.^135,136^ Thus, it is a promising strategy to activate endogenous progenitor cells or transplant exogenous progenitor cells for DDD therapy. However, a comprehensive understanding of their in vivo characteristics, including discriminable identity, lineage, spatial distribution, and functional role, is still lacking. We sought to help to increase the understanding of progenitor cells at a single-cell resolution. Surprisingly, we found a cluster of cells that exclusively expressed *PDGFRA*, a signature of MSCs,^77,137,138^ and was mainly distributed in the NP (Supplementary Fig. 4). Notably, the PDGFRA^+^PROCR^+^ NPPC subcluster was enriched for genes in the SMAD3 signaling pathway and exhibited higher activation of p-SMAD3 (Fig. 3), which determines the TGF-β-induced chondrogenesis^139^ and cell fate decisions of stem cells by participating in the cell-cycle process and binding of m^6^A methyltransferase.^140,141^ Moreover, *PROCR* was used to sort rare progenitor/stem cells with high efficacy. For example, *PROCR* (encoding CD201) was used as a sorting marker to harvest isolated 1% of islet cells, which robustly formed islet-like organoids.^75^ Applications in the hematopoietic system showed that *PROCR* enriched T1 prehematopoietic stem cells at a resolution of 68 parts per million and functional HSCs in the human fetal liver.^71,73^ In this study, we identified an NPPC cluster that highly expressed *PROCR*, which exhibited pluripotency with colony-formation capacity and osteochondrogenic potentials (Figs. 3 and 4), similar to the characteristics of multipotent mesenchymal stromal cells.^142^ Thus, we characterized these cells as resident progenitor cells in the human IVD. It is possible that the alternative cell fate in NPPCs determines the outcome of the IVD when a degenerative program is initiated. On the one hand, the chondrogenic fate could help rebalance IVD homeostasis via cell replenishment.^143^ On the other hand, the osteogenic fate could lead to DDD by inducing heterotopic ossification.^144^ Accordingly, these results have new implications for innovative therapeutic strategies targeting NPPCs.

The two branches of the cell fate of NPPCs motivated us to explore the key regulatory factors. Resident progenitor cells are exhausted or altered during degeneration,^26,145^ indicating that the microenvironment has a significant influence on cell fate. To identify the key factors regulating the fate of NPPCs, CellChat analysis was used to dissect the intercellular crosstalk based on the signaling network in the human IVD (Fig. 5). We found that GF-related signaling pathways were involved in the crosstalk network, mainly including the previously reported FGF family,^143,146^ TGF-β family,^147,148^ BMP family,^149,150^ and PDGF family.^151^ Among them, TGF-β was important due to the high activation of SMAD3 in NPPCs. TGF-β directs embryonic matrix development within the notochord and promotes the differentiation of the sclerotome into the AF,^61,152,153^ suggesting that it is an inherent regulator of the human IVD. Previous studies have shown that the TGF-β family plays an important role in the development and protection of the IVD, especially in maintaining the phenotype of chondrocytes.^154^ Moreover, the loss of TGF-β signaling in growth plate chondrocytes and inner AF cells led to the loss of matrix tissue and endplate cartilage cells and abnormal growth plate cartilage morphology in Tgfbr2 conditional knockout mice.^155^ The critical role of TGF-β was also evidenced by the observation that the knockout of SMAD3, the key downstream target of TGF-β, led to the spontaneous development of IVD degeneration in 30-day-old mice.^156^ In addition, TGF-β has been shown to have a beneficial effect on chondrogenic anabolism in MSCs.^157^ In this study, *TGFB* was involved in regulating NPPCs, as evidenced by TGF-β3 promoting the chondrogenesis of PROCR^+^ cells (Fig. 5). Meanwhile, the secretory role of chondrocyte clusters on TGF-β should not be neglected in the human IVD (Fig. 5). Furthermore, *PDGF* was found to engage in regulating NPPCs, probably due to the exclusive expression of its receptor gene *PDGFRA* in NPPCs. Previous studies have reported that PDGF-AA is involved in the regulation of cell proliferation.^92,93^ In line with the CellChat analysis, we found that PDGF-AA significantly promoted the proliferation of PROCR^+^ cells (Fig. 5). Interestingly, all the minor clusters in the NP are involved in interacting with NPPCs, suggesting their potential role in regulating NPPCs and subsequently maintaining IVD homeostasis. Moreover, further investigations need to elucidate their roles and establish an innovative strategy to optimize the microenvironment and benefit IVD stem/progenitor cells.

Although we validated the existence of identified cell populations by flow cytometry, immunofluorescence staining, and scRNA-seq evidence from the rat IVD, we surprisingly found that Sample 1 was from a 16-year-old donor who suffered from vertebral fracture exhibited obvious variability in the proportion of cell clusters (Supplementary Fig. 1e). Acute trauma has been shown to stimulate resident cells to regenerate in previous studies.^158–160^ Interestingly, a recent study reported that NP cells derived from trauma patients showed higher adipogenic and chondrogenic potential than those derived from degenerated IVDs.^161^ Thus, we are more inclined to hypothesize that ECs, pericytes, and NPPCs are rare in the IVD, and acute trauma may induce local regeneration, which accounts for the unwanted distribution variability across donors. Due to the scarcity of desirable samples of healthy disks from young patients with vertebral fractures, this needs to be explored in future studies.

In summary, our study described the cell atlas of the human IVD, providing a valuable resource for further investigation of IVD homeostasis at the mechanistic level. The cellular heterogeneity and signaling network we uncovered help to increase the understanding of the human IVD at a single-cell level and provide crucial clues for establishing new therapeutic strategies for DDD treatment.

## Materials and methods

For full methods, see the Supplementary Methods.

### Human IVD tissue specimens

This study was approved by the Institutional Ethics Review Board of Daping Hospital [Ethics Committee (2019-127)] and the Chinese Clinical Trial registry (ChiCTR1900028201). All procedures were performed in accordance with the ethical standards of the committee responsible for human experimentation and with the Declaration of Helsinki of 1975, as revised in 2000. Informed consent was obtained from all patients for inclusion in the study. Eleven human IVDs were carefully dissected from nine donors in this study (Supplementary Table 1). The gelatinous tissue from the central region was harvested as the NP. The peripheral lamellar structure of the outer IVD was harvested as AF. The superior and inferior homogeneous cartilage tissue was harvested as CEP. The sampling areas of NP, AF, and CEP are indicated (Supplementary Fig. 1a).

### Single-cell RNA sequencing

The cells were washed with PBS three times and concentrated to 700–1 200 cells per μL. The suspension was then loaded on a Chromium Controller (10X Genomics). For scRNA-seq library construction, a Chromium Single Cell 3′ Library and Gel Bead Kit V2 (10X Genomics, PN120237) was utilized to generate single-cell gel beads in emulsion (GEM) within barcoded, full-length cDNA from polyadenylated mRNA. The captured cells were lysed in GEM, and the released RNA was reverse-transcribed with primers containing poly-T, a barcode, UMIs, and the read 1 primer sequence, in that order. Barcoded, full-length cDNA was PCR amplified for library construction. After enzymatic fragmentation, an adapter ligation reaction was performed to add a sample index and read 2 primer sequences to the cDNA fragment. After quality control, the libraries were sequenced on an Illumina NovaSeq 6000 platform to generate 150-bp paired-end reads, according to the manufacturer’s instructions (Berry Genomics).

## Supplementary information

Supplementary Methods

Supplementary Table 1

Supplementary Table 2

Supplementary Table 3

Supplementary Table 4

Supplementary Table 5

Supplementary Table 6

Supplementary Table 7

Supplementary Table 8

Supplementary Table 9

Supplementary Figure 1

Supplementary Figure 2

Supplementary Figure 3

Supplementary Figure 4

Supplementary Figure 5

Supplementary Figure 6

## Data Availability

All data from the study are available in online supplementary files. The scRNA-seq data have been deposited in GEO (GSE160756). All other relevant data from this study are available from the corresponding authors upon reasonable request.
