# Supplementary material for: Spatially defined single-cell transcriptional profiling characterizes diverse chondrocyte subtypes and nucleus pulposus progenitors in human intervertebral discs
Source: Bone Res. 2021 Aug 16;9:37. doi: 10.1038/s41413-021-00163-z (PMC8368097; doi:10.1038/s41413-021-00163-z)
Supplement: Supplementary file 1 — Supplementary Methods [file 41413_2021_163_MOESM1_ESM.docx]

**Supplementary Methods**

7.0T micro-MRI imaging of IVD sample

The discs were transported in the MACS Tissue Storage Solution (Miltenyi, Germany) at 4⁰C for 1 hour before scanning. T2-weighted, T1-weighted sagittal, and axial MRI were acquired from the two healthy lumbar spines on a 7.0T micro-MRI machine ( BioSpec 70/20 USR, BRUKER, Germany) at Daping Hospital according to a previous study.^1^ The field of view (FOV) for the chemical exchange saturation transfer (CEST) imaging was adjusted to 60×60 mm2 to accommodate the size of human lumbar discs (matrix size = 384×384, slice thickness = 1 mm). Single-channel volume radiofrequency (RF) coils were 78 mm for human cadaveric discs. A blinded observer scored the grade of degeneration on T2 weighted scans according to the classification scale.^2^

Preparation of human IVD single-cell suspension

To avoid the obstructed dissociation caused by dense extracellular matrix (ECM) and capture scare cell populations, we performed the effective two-step isolation as follows: (1) Using a cocktail of less harmful and ECM-specific enzymes within a shorter time to improve the isolation efficiency and reduce the impairment to the embedded cells;^3^ (2) Using magnetic bead separation to remove the dead cells and tissue debris (Fig. 1a).^4^ The discs sample was stored in the MACS Tissue Storage Solution (Miltenyi, Germany) at 4⁰C within 4 hours before cell separation. Separated NP, AF, and CEP samples were minced as finely as possible and washed several times by phosphate-buffered saline (PBS) until no visible blood contamination. Single-cell pituitary suspensions were then prepared based on the enzyme cocktail.^5^ The samples were transferred to pre-warmed TrypLE Express (Hyclone, USA) at 37℃ for 30 min, followed by digestion with 0.2% pronase (Sigma-Aldrich, USA) for 60 min. After that, the tissues were incubated with 0.012% collagenase II (Sigma-Aldrich, USA) under slow rotation for 2~4h, depending on tissue residue. We terminated the enzymatic digestion when the tissue pieces have entirely disintegrated and gently pipette about 20 times. The digested cells were filtered through a 40-mm cell strainer (JETBIOFIL, China) to remove tissue chips. Subsequently, the cell suspension was treated with the 1× Red Blood Cell Lysis Solution (Miltenyi, Germany) to remove the residual blood cells in the samples. Dead cells were eliminated by labeling cells with Dead Cell Removal Kit (Miltenyi, Germany) and separated over an LS column in the magnetic field of a MidiMACS™ Separator (Miltenyi, Germany). Cell viability was tested by AO/PI staining using the Rigel S2 Cell Counter (Countstar, China).

scRNA-seq data processing

For each library, sequenced raw reads embedded in fastq files were processed using Cellranger (v4.0.0) count function. Human genome reference GRCh38 (Ensembl 93) was used to align the reads, and count matrixes were generated after low-quality reads were filtered with default parameters. For quality control, we applied Scrublet (v0.2.1)^6^ to remove doublets for each data set, and cells with more than 200 genes expressed and counts of the mitochondrial gene in all counts less than 10% were retained for downstream analysis.

Integration, dimensionality reduction, and clustering

We used Seurat (v3.2.2)^7^ workflow for data set integration and dimensionality reduction and clustering. Briefly, the fastMNN function implemented in the SeuratWrappers package was used to remove batch effects among the data set. A union of the top 3,000 genes with the highest dispersion for each data set to generate an integrated matrix. The top 50 components were used for tSNE (t-distributed stochastic neighbor embedding) dimensionality reduction. For first-round clustering, the shared nearest neighbor (SNN) graph was constructed with k.param set as 20, and the Findcluster function was performed using the Louvain algorithm with the resolution set as 0.2, resulting in nine major cell clusters for subsequent analyses. For second-round chondrocyte sub-clustering, we reconstructed the SNN graphs for Chond1, Chond2, and Chond3 clusters, respectively, and two subclusters were determined resolution set as 0.1 for each chondrocyte cluster. For second-round stroma sub-clustering, seven subclusters were found with the resolution set as 0.2.

Analysis of single-cell Transcriptomic data

Original fastq data were trimmed and transformed into digital expression matrix following the Droplet-based scRNA-seq data analysis pipeline described in detail above. A filter was applied to remove low-quality cells and doublets. Then R package "Seurat", "SCENIC", "Monocle 3" and "Cellchat" were applied to perform downstream analysis and visualization. Gene ontology was performed by R package "clusterProfiler" and "ReactomePA". Parameters for each function were described above.

Differential expression analyses

The FindAllMarkers function implemented in Seurat was used to calculate DEGs among different clusters. The Wilcoxon test was performed on each gene, and the adjusted P-value for statistical significance was computed. Genes met the criteria that 1) expressing in a minimum fraction of 20% in either of the two tested populations; 2) at least 0.25-fold difference (log-scale) between the two tested populations; 3) adjusted P values less than 0.01, were considered as signature genes. Cell clusters were annotated according to the expression of those signature genes reported in the literature.

Species comparative analysis

For comparative analysis between human and rat datasets, the expression data matrix of rat IVD from GSE154884 was collected.^8^ To ensure comparability, the rat datasets were processed by the same steps as human datasets, including dimension reduction and clustering. We calculated the Pearson correlation of average expression of homologous genes in cell clusters between human and rat cells. The overlap of DEGs from human and rat NPPC clusters were listed.

Gene regulatory network analyses

We applied Single Cell Regulatory Network Inference and Clustering (SCENIC) ^9^ to identify NPPC cluster-specific gene regulatory networks. The expression matrix was filtered for genes available in RcisTarget's human database (hg19-tss-centered-10kb). Spearman correlation matrix was conducted using the cor function implemented in the stats package. The Pyscenic grn function was used for building the initial co-expression gene regulatory networks (GRN). The regulon data was then analyzed using the RcisTarget package to create TF motifs using the hg19-tss-centered-10kb database. The regulon activity scores were calculated using Area Under the Curve (AUC) function.

Regulon activity-based tSNE visualization was performed. Active binary regulons were determined by being detected in at least 50% of cells in one of the NPPC clusters. Predicted target genes of regulon were ranked by Genie3Weight value and filtered by normalized enrichment score (NES) of binding motifs. The transcriptional network of TF and predicted target genes were visualized by Cytoscape (v3.6). Edges indicated the Genie3Weights and node size indicated the number of motifs.

Flow cytometry

The following antibodies were used: PDGFRA-FITC (1:50, MA5-28585, Invitrogen, USA) and PROCR-BV786 (1:50,743556, BD, USA). Cells were stained in sorting buffer (PBS +3% BSA) for 30 min at 4 °C, washed once, and resuspended in sorting buffer with 7-AAD (1:200, 00-6993-50, eBioscience, USA). Flow cytometry was performed on BD FACS Aria II. Pre-gating was first done for live cells based on 7-AAD staining. Gating strategies were based on Fluorescence Minus One (FMO) controls. FlowJo v10.6 software was used for analyzing the flow cytometry data.

CFU-F culture

For CFU-F cultures, sorted cells were seeded in 6-well plates (1 × 10^3^ cells/well) containing Mesenchymal Stem Cell Basal Medium (DAKEWE, China) supplemented with 5% EliteGro-Adv (EPA-050, Elitecell, USA), 1% Penicillin/Streptomycin solution, and incubated at 37 °C with 5% CO_2_. Half of the medium was changed every 3–4 days. On day 14, cells were fixed and stained with crystal violet staining solution. Adherent colonies with more than 50 cells were quantified.

Cell proliferation assay

The cell proliferation of PROCR^+^ cells from human IVD was determined by CCK-8 assay on day 3, 7, and 10. The PROCR+ cells were cytometrically sorted from human IVD and sorted cells were cultured for 14 days and replated at a density of 1000/well in 96-well plates (medium was changed every 3 days). Then the cells were cultured in αMEM(Gibco, USA) containing 10% fetal bovine serum (FBS, Gibco, USA) with supplementation of PDGF-AA (20 ng/ml, 100-13, PeproTech, USA) and/or Crenolanib (10 nM, S2730, Selleck, China), the PDGFR α/β inhibitor for 10 days. Aliquots of each 10μl CCK-8 solution (Dojindo, Japan) were added to each well and incubated at 37 °C for 2 h until the color turned to orange. The optical density (OD) value was measured at 450 nm with a microplate reader (Bio-Rad Laboratories, USA) (n=3).

Single-cell trajectory construction

Single-cell trajectory was performed on cell clusters (exclude neurogenic and hematopoietic cells) in NP using the Monocle3 package (v0.2.3).^10^ We identified highly variable genes (HVGs) for the new pooled dataset and re-calculated the PCs (principal components) with default parameters. We performed Uniform Manifold Approximation and Projection (UMAP) for dimensionality reduction, which was subjected to Monocle3 for trajectory construction using learn_graph function with NPPC-3 set as the root node and minimal_branch_len set as 5. We then utilized the find_gene_module function to group co-regulated genes into modules using Louvain community analysis. The module scores were calculated by aggregating all genes expressed in each module across all the clusters and visualized by heatmap.

Adipogenic, osteogenic, and chondrogenic differentiation assays

For adipogenic and osteogenic differentiation, sorted cells were cultured for 14 days and replated at a density of 2.0 × 10^4^/cm^2^. Adipogenic differentiation was performed in Adipogenic Differentiation Kit (STEMCELL, USA) for 2 weeks (medium was changed every 3 days), and quantified by oil red O staining (Sigma-Aldrich, USA). Osteogenic differentiation was performed in Osteogenic Differentiation Kit (STEMCELL, USA) for 2 weeks (medium was changed every 3 days) and quantified by alizarin red staining (Sigma-Aldrich). For chondrogenic differentiation, 2.5 × 10^5^ cultured cells were centrifugated at 300g in 15 mL polypropylene conical tubes to form pellets and cultured in a chondrogenic medium (Cyagen, China) for 4 weeks (medium was changed every 3 days). The chondrogenic medium contained 100 nM dexamethasone, 50 μg/mL ascorbic acid, 1mM sodium pyruvate, 40 μg/mL proline and 1× ITS cell culture, and 10 ng/mL TGFβ3 (Peprotech, USA). Chondrogenic differentiation was quantified by 4% polyoxymethylene of the cell pellets followed by safranin O/fast green (Solarbio, China) and alcian blue staining (Sigma-Aldrich, USA). For investigating the role of the TGF-β3 on the chondrogenesis in the PROCR^+^ cells, the PROCR^+^ cells were cytometrically sorted from human IVD and sorted cells were cultured for 14 days and replated at a density of 2.0 × 10^4^/cm^2^. Then the cell pellets were cultured in the chondrogenic medium as described above with supplementation of TGF-β3 (10 ng/ml) and/or SB505124 (10 uM, Selleck, China), the TGF-β receptor inhibitor, for 4 weeks. Chondrogenic differentiation was quantified by 4% polyoxymethylene of the cell pellets followed by toluidine blue (Sigma-Aldrich, USA) and alcian blue staining (Sigma-Aldrich, USA).

Matrisome and gene set signature analyses

A set of 713 matrisome-genes detected in chondrocytes was generated according to a previous study^11^, including 41, 157, and 30 genes encoding collagens, ECM glycoproteins, and proteoglycans, which categorized into core matrisome genes, and 163, 102, and 220 genes encoding ECM regulators, ECM-affiliated proteins and secreted factors, which categorized into matrisome-associated genes. Gene sets of oxidative phosphorylation, glycolysis, cell adhesion, circadian regulation, ossification, chondrocyte differentiation were selected from the Molecular Signatures Database (C2: curated gene sets) website. Module scores for each gene set were calculated using the AddModuleScore function implemented in Seurat. The ggradar package (v0.2) was used to visualize the relative abundance of given gene set in each chondrocyte subcluster.

Gene functional annotation analysis

GO enrichment analysis was performed on DEGs using the clusterProfiler package.^12^ P values of each enriched term were adjusted by the Benjamini-Hochberg method and only terms with P value less than 0.05 were retained. We also removed redundancy of the enriched term using simplify the function. Gene sets enrichment analysis ^13^ (GSEA, v4.0) was performed on C2 Gene sets to screen signaling pathways enriched in NPPC clusters. We applied the enrichPathway function in the ReactomePA^14^ package for signaling pathways enrichment of genes in given gene modules from Monocle3 analysis.

Surface marker genes, TFs and cell-cycle

For screening cell surface protein to FACS-sort NPPCs, we downloaded the surface marker gene list from the in silico human surfaceome database (http://wlab.ethz.ch/surfaceome/) and filtered the candidate genes from DEGs among NPPC cell clusters. TF list was downloaded HumanTFDB3.0 (http://bioinfo.life.hust.edu.cn/HumanTFDB/) website. We used the previously reported G1/S and G2/M phase-specific genes to compute scores of S phase and G2M phase, which categorized cells into three cell-cycle status (G1, S, and G2M)^15^.

Cell communications

Cellchat package (v0.0.2)^16^ was conducted to analyze the cell communications in NP clusters. A standard pipeline could be found in https://github.com/sqjin/CellChat/blob/master/vignettes. We firstly set ligand-receptor interaction list in human and selected "Secreted Signaling", "ECM-Receptor" and "Cell-Cell Contact" as communicating ways. We then projected the gene expression data onto the protein-protein interaction (PPI) network by identifying the over-expressed ligand-receptor interactions. To obtain the biologically significant cell-cell communications, probability values for each interaction were calculated by performing permutation tests. The inferred intercellular communication network of each ligand-receptor pair and each signaling pathway was summarized and visualized by circle plots. We also computed the importance of each cell cluster in the signaling pathway across all ligand-receptor interactions. To identify the cell clusters that coordinate to function, we grouped all signaling into distinct patterns according to expression levels of ligand-receptor interactions in signaling for each cell group. The top 5 "outgoing" patterns (means cells function as secreting roles) and "incoming" patterns (means cells function as target cells) were visualized by river plots.

Histological, immunohistochemical, and immunofluorescence analysis

After IVD sampling fixation, histological analysis was performed washing 4% paraformaldehyde for seven days and processed with decalcification by EDTA for 30 days. For the chondrogenesis experiment, the cell pellets were processed with twice rinse with PBS, and fixation using 4% paraformaldehyde for three days. Then, we embedded the fixed samples with paraffin and sectioned them into 6-mm-thick slices. The hematoxylin and eosin (H&E) and safranin O / fast green staining were performed on the IVD sections. The alcian blue, toluidine blue, and safranin O/fast green staining were performed on the cell pellets.

For visualization of cell clusters' distribution in the IVD in situ, the samples were then stained with anti-SOX9 antibody (1:200, ab185966, Abcam, UK), anti-PDGFRα antibody (1:200, ab203491, Abcam, UK), anti-PECAM-1 antibody (1:200, ab28364, Abcam, UK) and anti-ACTA2 antibody (1:2000, ab5694, Abcam, UK) following a standard staining protocol. For quantitative examination, the section results were scanned by a high-resolution digital slide scanner (VS-200, Olympus, Japan).

For detection of the co-expression of signature genes and their spatial distribution of cell clusters, immunofluorescence staining was performed using anti-PDGFRα(1:200, ab185966, Abcam, UK), anti-PROCR (1:1000, ab56689, Abcam, UK), anti-PRRX1 (1:200, ab211292, Abcam, UK), anti-PECAM1 (1:200, ab24590, Abcam, UK), anti-CD34 (1:200, ab81289, Abcam, UK), anti-ACTA2 (1:2000, ab5694, Abcam, UK), anti-SOX2 (1:200, ab79351, Abcam, UK), anti-RUNX2 (1:100, ab76956, Abcam, UK) and anti-SP7 (1:200, ab16669, Abcam, UK). In short, we incubated the sections with proteinase K (1:200, Solarbio, China) for 30 min, subsequently with Triton X-100 (0.1%, Beyotime, China) for 30 min, and with normal goat serum (ZSGB-BIO, China) for 30 min. Next, the primary antibodies were applied to treat the sections at 4℃ overnight, and then the secondary antibodies (1:1000, A11008, A21422, A11001, A21428, Invitrogen, USA) were applied for 1 h at room temperature. Finally, DAPI solution (0.1%, Beyotime, China) was applied to visualize the cell nucleus for 10 min at room temperature. The immunostaining image was captured by the confocal microscopy system (IXplore SpinSR10, Olympus, Japan; LSM780, ZEISS, German) and analyzed by the matching softwares (cellSens Dimension, Olympus, Japan; Zen 2.3, ZEISS, German).

The expression of the p-SMAD3 in PROCR^+^ and PROCR^-^ cells of P2 from the human IVD was evaluated by immunofluorescence staining. After 24 h seeding on the sterile slides in 24-well plates, cells were washed with PBS for 3 times and fixed with 4% paraformaldehyde for 20 min. Cells on the slides were permeabilized with 0.2% Triton X-100 for 5 min and blocked with 5% BSA for 1 h. Then slides were incubated with the primary antibody (1:100, ab52903, Abcam, UK; 1:100, sc-101154, Santa Cruz, USA) overnight at 4 °C. Followed by incubation with fluorescent secondary antibody (1:1000, A11001; 1:1000, A21428, Invitrogen, USA) for 1 h at room temperature without lighting, cell nuclei were stained with DAPI for 5 min. Slides were imaged by confocal microscopy (LSM780, ZEISS, German).

**References**

1 Tam, V. *et al.* *The spatial proteome of the human intervertebral disc reveals architectural changes in health, ageing and degeneration*. (2020).

2 Pfirrmann, C. W., Metzdorf, A., Zanetti, M., Hodler, J. & Boos, N. Magnetic resonance classification of lumbar intervertebral disc degeneration. *Spine* **26**, 1873-1878, doi:10.1097/00007632-200109010-00011 (2001).

3 Reichard, A. & Asosingh, K. Best Practices for Preparing a Single Cell Suspension from Solid Tissues for Flow Cytometry. *Cytometry. Part A : the journal of the International Society for Analytical Cytology* **95**, 219-226, doi:10.1002/cyto.a.23690 (2019).

4 van Galen, P. *et al.* Single-Cell RNA-Seq Reveals AML Hierarchies Relevant to Disease Progression and Immunity. *Cell* **176**, 1265-1281.e1224, doi:<https://doi.org/10.1016/j.cell.2019.01.031> (2019).

5 Sakai, D. *et al.* Successful fishing for nucleus pulposus progenitor cells of the intervertebral disc across species. *JOR spine* **1**, e1018, doi:10.1002/jsp2.1018 (2018).

6 Wolock, S. L., Lopez, R. & Klein, A. M. Scrublet: Computational Identification of Cell Doublets in Single-Cell Transcriptomic Data. *Cell systems* **8**, 281-291.e289, doi:10.1016/j.cels.2018.11.005 (2019).

7 Butler, A., Hoffman, P., Smibert, P., Papalexi, E. & Satija, R. Integrating single-cell transcriptomic data across different conditions, technologies, and species. *Nature biotechnology* **36**, 411-420, doi:10.1038/nbt.4096 (2018).

8 Wang, J. *et al.* Novel biomarkers of intervertebral disc cells and evidence of stem cells in the intervertebral disc. *Osteoarthritis and cartilage*, doi:10.1016/j.joca.2020.12.005 (2020).

9 Aibar, S. *et al.* SCENIC: single-cell regulatory network inference and clustering. *Nature methods* **14**, 1083-1086, doi:10.1038/nmeth.4463 (2017).

10 Cao, J. *et al.* The single-cell transcriptional landscape of mammalian organogenesis. *Nature* **566**, 496-502, doi:10.1038/s41586-019-0969-x (2019).

11 Naba, A. *et al.* The extracellular matrix: Tools and insights for the “omics” era. *Matrix Biology* **49**, 10-24, doi:<https://doi.org/10.1016/j.matbio.2015.06.003> (2016).

12 Yu, G., Wang, L. G., Han, Y. & He, Q. Y. clusterProfiler: an R package for comparing biological themes among gene clusters. *Omics : a journal of integrative biology* **16**, 284-287, doi:10.1089/omi.2011.0118 (2012).

13 Subramanian, A. *et al.* Gene set enrichment analysis: a knowledge-based approach for interpreting genome-wide expression profiles. *Proceedings of the National Academy of Sciences of the United States of America* **102**, 15545-15550, doi:10.1073/pnas.0506580102 (2005).

14 Yu, G. & He, Q. Y. ReactomePA: an R/Bioconductor package for reactome pathway analysis and visualization. *Molecular bioSystems* **12**, 477-479, doi:10.1039/c5mb00663e (2016).

15 Tirosh, I. *et al.* Dissecting the multicellular ecosystem of metastatic melanoma by single-cell RNA-seq. *Science (New York, N.Y.)* **352**, 189-196, doi:10.1126/science.aad0501 (2016).

16 Jin, S. *et al.* *Inference and analysis of cell-cell communication using CellChat*. (2020).
