# Supplementary material for: Spatially defined single-cell transcriptional profiling characterizes diverse chondrocyte subtypes and nucleus pulposus progenitors in human intervertebral discs
Source: Bone Res. 2021 Aug 16;9:37. doi: 10.1038/s41413-021-00163-z (PMC8368097; doi:10.1038/s41413-021-00163-z)
Supplement: Supplementary file 3 — Supplementary Table 2 [file 41413_2021_163_MOESM3_ESM.pdf]

Supplementary Table 2.
DEGs among the 9 clusters defined in human IVD

p_val	avg_logFC	pct.1	pct.2	p_val_adj	cluster	gene
0	3.25665578	0.654	0.219	0	NPPC	PLA2G2A
0	2.65922332	0.797	0.039	0	NPPC	FBLN1
0	2.63184202	0.861	0.033	0	NPPC	SERPINF1
0	2.59471685	0.659	0.144	0	NPPC	CFD
0	2.51145059	0.301	0.029	0	NPPC	PTGDS
0	2.4895023	0.502	0.014	0	NPPC	WISP2
0	2.35766279	0.738	0.256	0	NPPC	IGFBP6
0	2.21530418	0.933	0.592	0	NPPC	GSN
0	2.14623931	0.695	0.022	0	NPPC	MMP2
0	2.1346376	0.857	0.226	0	NPPC	C1R
0	2.11523787	0.659	0.313	0	NPPC	CFH
0	2.08361065	0.272	0.008	0	NPPC	ANGPTL7
0	2.07344274	0.351	0.028	0	NPPC	RARRES1
0	1.97459231	0.314	0.008	0	NPPC	C7
0	1.89560472	0.646	0.051	0	NPPC	COL12A1
0	1.73920142	0.392	0.035	0	NPPC	MYOC
0	1.7173005	0.645	0.076	0	NPPC	COL14A1
0	1.69465896	0.488	0.029	0	NPPC	THBS4
0	1.67420573	0.289	0.056	0	NPPC	APOE
0	1.63556922	0.662	0.094	0	NPPC	SPARCL1
0	1.59677979	0.697	0.081	0	NPPC	LTBP4
0	1.56510466	0.588	0.128	0	NPPC	ASPN
0	1.56243303	0.607	0.119	0	NPPC	THBS2
0	1.53625833	0.922	0.617	0	NPPC	SERPING1
0	1.52134645	0.377	0.006	0	NPPC	SFRP2
0	1.50107127	0.863	0.431	0	NPPC	C1S
0	1.47361181	0.484	0.119	0	NPPC	HBB
0	1.45837261	0.357	0.009	0	NPPC	C3
0	1.42056497	0.367	0.015	0	NPPC	EFEMP1
0	1.40073572	0.672	0.135	0	NPPC	PDGFRA
0	1.39648604	0.493	0.043	0	NPPC	PLPP3
0	1.37135812	0.655	0.128	0	NPPC	LHFPL6
0	1.36790871	0.459	0.033	0	NPPC	TNXB
0	1.35099735	0.831	0.389	0	NPPC	TCF4
0	1.35005136	0.531	0.024	0	NPPC	GPNMB
0	1.3483271	0.805	0.616	0	NPPC	GPX3
0	1.34765648	0.694	0.27	0	NPPC	COL6A3
0	1.34267727	0.562	0.05	0	NPPC	EBF1
0	1.33747593	0.423	0.009	0	NPPC	ABCA6
0	1.29907294	0.404	0.006	0	NPPC	IGF1
0	1.29186901	0.634	0.172	0	NPPC	PDGFRL
0	1.28887416	0.868	0.342	0	NPPC	COL1A2
0	1.28736143	0.509	0.125	0	NPPC	GAS1
0	1.2859849	0.677	0.111	0	NPPC	IGFBP5
0	1.25290567	0.725	0.081	0	NPPC	COL1A1
0	1.23202619	0.563	0.103	0	NPPC	LIMA1
0	1.22431235	0.702	0.323	0	NPPC	MXRA8

0	1.22344181	0.486	0.099	0	NPPC	NTRK2
0	1.21511565	0.646	0.181	0	NPPC	PRRX1
0	1.20749085	0.533	0.097	0	NPPC	METTL7A
0	1.19874571	0.45	0.064	0	NPPC	CYP1B1
0	1.1970285	0.544	0.067	0	NPPC	RBMS3
0	1.19465902	0.455	0.052	0	NPPC	MGST1
0	1.18797392	0.911	0.561	0	NPPC	IGFBP7
0	1.16215675	0.601	0.14	0	NPPC	NFIB
0	1.15955966	0.764	0.39	0	NPPC	TXNIP
0	1.15565775	0.42	0.032	0	NPPC	GGT5
0	1.15093485	0.658	0.222	0	NPPC	MARCKS
0	1.14124897	0.837	0.478	0	NPPC	LRP1
0	1.12599335	0.706	0.236	0	NPPC	COL3A1
0	1.11222432	0.47	0.127	0	NPPC	NR2F2
0	1.1121798	0.942	0.491	0	NPPC	TMSB4X
0	1.08033966	0.43	0.037	0	NPPC	COL15A1
0	1.07324326	0.592	0.229	0	NPPC	PIK3R1
0	1.06636149	0.627	0.235	0	NPPC	SPTBN1
0	1.06437411	0.496	0.09	0	NPPC	PDGFRB
0	1.0633187	0.419	0.068	0	NPPC	NBL1
0	1.0579395	0.704	0.382	0	NPPC	CYBRD1
0	1.05676471	0.769	0.36	0	NPPC	ZFP36L1
0	1.03953866	0.883	0.464	0	NPPC	CALD1
0	1.03428792	0.359	0.007	0	NPPC	LAMA2
0	1.02467216	0.666	0.286	0	NPPC	EPB41L2
0	1.01559408	0.33	0.04	0	NPPC	CXCL12
0	1.00948436	0.971	0.817	0	NPPC	MT-ND3
0	1.00708015	0.496	0.12	0	NPPC	COLEC12
0	1.00410688	0.622	0.229	0	NPPC	FOXP1
0	1.00160574	0.477	0.087	0	NPPC	ADD3
0	0.9974531	0.378	0.045	0	NPPC	FBLN2
0	0.97955543	0.374	0.047	0	NPPC	SNED1
0	0.97026223	0.415	0.058	0	NPPC	CDH11
0	0.96250214	0.289	0.007	0	NPPC	VIT
0	0.95468639	0.838	0.696	0	NPPC	CD9
0	0.95432565	0.338	0.067	0	NPPC	PTGFR
0	0.95084495	0.295	0.054	0	NPPC	TNC
0	0.95051417	0.34	0.009	0	NPPC	PODN
0	0.94987929	0.605	0.231	0	NPPC	IFI16
0	0.93970191	0.312	0.03	0	NPPC	SEMA3C
0	0.92437043	0.414	0.047	0	NPPC	BICC1
0	0.90301011	0.433	0.08	0	NPPC	LTBP1
0	0.89578444	0.229	0.006	0	NPPC	ABCA10
0	0.88942978	0.496	0.182	0	NPPC	DAB2
0	0.8677339	0.372	0.12	0	NPPC	LTBP2
0	0.8667589	0.346	0.034	0	NPPC	AKR1C1
0	0.86666391	0.465	0.153	0	NPPC	PLEKHH2
0	0.84609073	0.249	0.03	0	NPPC	SPRY1
0	0.84538026	0.467	0.153	0	NPPC	PLS3
0	0.83173097	0.453	0.135	0	NPPC	FTX
0	0.82760952	0.433	0.154	0	NPPC	FBN1

0	0.81900132	0.405	0.091	0	NPPC	IGFBP4
0	0.8094868	0.412	0.112	0	NPPC	AKAP12
0	0.80767528	0.434	0.14	0	NPPC	ZFHX4
0	0.80645467	0.397	0.104	0	NPPC	ANTXR1
0	0.79994602	0.964	0.858	0	NPPC	MT-CYB
0	0.79803595	0.67	0.389	0	NPPC	NFIA
0	0.79734535	0.951	0.824	0	NPPC	MT-ND2
0	0.79631532	0.413	0.119	0	NPPC	DLC1
0	0.79098437	0.351	0.06	0	NPPC	ZEB2
0	0.78944543	0.35	0.085	0	NPPC	ITGBL1
0	0.78720337	0.35	0.084	0	NPPC	EPHX1
0	0.78427914	0.378	0.075	0	NPPC	MAN1A1
0	0.78048902	0.312	0.021	0	NPPC	FGL2
0	0.77509038	0.397	0.101	0	NPPC	SASH1
0	0.77367056	0.289	0.071	0	NPPC	FLRT2
0	0.77243842	0.285	0.014	0	NPPC	CELF2
0	0.77165485	0.28	0.036	0	NPPC	TSHZ2
0	0.76675624	0.286	0.021	0	NPPC	PCDH18
0	0.76277786	0.789	0.466	0	NPPC	IFITM2
0	0.7610229	0.764	0.501	0	NPPC	AHNAK
0	0.75622672	0.856	0.664	0	NPPC	LGALS1
0	0.75582913	0.425	0.131	0	NPPC	PLSCR4
0	0.75041913	0.861	0.604	0	NPPC	COL6A2
0	0.74841708	0.323	0.089	0	NPPC	TFPI
0	0.74709664	0.277	0.032	0	NPPC	PLTP
0	0.74685151	0.926	0.669	0	NPPC	IFITM3
0	0.74160256	0.229	0.013	0	NPPC	SFRP4
0	0.74023821	0.866	0.705	0	NPPC	TIMP2
0	0.73514408	0.365	0.088	0	NPPC	RUNX1T1
0	0.72030663	0.209	0.005	0	NPPC	SCARA5
0	0.71014071	0.35	0.102	0	NPPC	PROS1
0	0.70423105	0.266	0.042	0	NPPC	SRPX
0	0.69732657	0.961	0.869	0	NPPC	MT-CO2
0	0.69605185	0.338	0.053	0	NPPC	COL4A1
0	0.69498308	0.916	0.695	0	NPPC	TMSB10
0	0.6932765	0.486	0.203	0	NPPC	DPYSL2
0	0.68664866	0.249	0.013	0	NPPC	EPHA3
0	0.68347872	0.206	0.017	0	NPPC	ADAMTS5
0	0.67921157	0.261	0.036	0	NPPC	EGFR
0	0.67698568	0.376	0.13	0	NPPC	11-Sep
0	0.669245	0.302	0.06	0	NPPC	LAMA4
0	0.66876654	0.251	0.025	0	NPPC	BNC2
0	0.66724504	0.253	0.053	0	NPPC	NRP1
0	0.66623959	0.226	0.031	0	NPPC	LAMB1
0	0.66614261	0.978	0.896	0	NPPC	MT-ATP6
0	0.66334396	0.334	0.093	0	NPPC	LAMC1
0	0.66067123	0.262	0.035	0	NPPC	COL8A1
0	0.66018443	0.25	0.018	0	NPPC	MEOX2
0	0.65076655	0.22	0.031	0	NPPC	CYGB
0	0.64741343	0.96	0.862	0	NPPC	MT-CO3
0	0.64536689	0.27	0.028	0	NPPC	ABLIM1

0	0.64382656	0.302	0.07	0	NPPC	FAP
0	0.64273587	0.28	0.024	0	NPPC	SLC9A9
0	0.63998301	0.221	0.006	0	NPPC	SVEP1
0	0.6360803	0.3	0.041	0	NPPC	HSD17B11
0	0.63358748	0.272	0.049	0	NPPC	STEAP2
0	0.6317215	0.952	0.86	0	NPPC	ITM2B
0	0.62537835	0.258	0.036	0	NPPC	PLA2R1
0	0.62380297	0.254	0.061	0	NPPC	MFAP4
0	0.6209808	0.284	0.054	0	NPPC	AR
0	0.61911962	0.224	0.042	0	NPPC	MXRA5
0	0.61056105	0.304	0.052	0	NPPC	COL4A2
0	0.6098608	0.285	0.029	0	NPPC	TWIST1
0	0.59082248	0.218	0.021	0	NPPC	PLXDC1
0	0.58334411	0.267	0.049	0	NPPC	MEDAG
0	0.583187	0.224	0.016	0	NPPC	LGALS3BP
0	0.57461486	0.23	0.044	0	NPPC	ANTXR2
0	0.57126748	0.283	0.075	0	NPPC	PTPRG
0	0.54881177	0.252	0.037	0	NPPC	SYNE3
0	0.5452239	0.237	0.054	0	NPPC	GHR
0	0.52400302	0.203	0.016	0	NPPC	MITF
0	0.51968209	0.227	0.029	0	NPPC	CPED1
0	0.51902577	0.218	0.046	0	NPPC	CLEC2B
0	0.51808081	0.221	0.044	0	NPPC	FAT4
0	0.5172142	0.215	0.02	0	NPPC	OLFML3
0	0.51335684	0.244	0.056	0	NPPC	SLCO3A1
0	0.50536425	0.988	0.964	0	NPPC	VIM
0	0.50432628	0.227	0.036	0	NPPC	FAXDC2
0	0.49209895	0.204	0.029	0	NPPC	ADAMTSL3
0	0.49060426	0.243	0.058	0	NPPC	TNFSF10
0	0.49041848	0.223	0.047	0	NPPC	TBC1D2B
0	0.47600597	0.203	0.022	0	NPPC	XAF1
0	0.42941784	0.218	0.043	0	NPPC	CASP4
0	0.31476978	0.439	0.134	0	NPPC	TAGLN
1.34E-298	0.56081932	0.329	0.105	3.38E-294	NPPC	EPS15
4.62E-298	0.66492718	0.76	0.51	1.16E-293	NPPC	DDX17
1.09E-291	0.48801214	0.205	0.046	2.76E-287	NPPC	PTPN13
1.11E-287	0.71559616	0.548	0.275	2.81E-283	NPPC	LTBP3
8.83E-282	0.67216903	0.418	0.168	2.23E-277	NPPC	PLAGL1
7.72E-270	0.67220142	0.402	0.161	1.95E-265	NPPC	CEP126
1.65E-267	0.66980059	0.457	0.208	4.16E-263	NPPC	YPEL3
2.28E-259	0.66539856	0.288	0.093	5.74E-255	NPPC	PROCR
1.70E-258	0.41158391	0.204	0.049	4.29E-254	NPPC	DIO3OS
1.30E-255	0.48977568	0.246	0.07	3.29E-251	NPPC	EZH1
2.66E-247	0.77481309	0.499	0.264	6.72E-243	NPPC	CTSK
3.53E-247	0.6488477	0.68	0.454	8.91E-243	NPPC	HP1BP3
2.00E-246	0.70316858	0.469	0.223	5.04E-242	NPPC	ECM2
1.32E-245	0.73710039	0.515	0.271	3.33E-241	NPPC	FSTL1
1.24E-240	0.52410465	0.295	0.1	3.14E-236	NPPC	CALCOCO1
2.14E-240	0.82876251	0.345	0.132	5.39E-236	NPPC	ACKR3
2.94E-240	0.54175641	0.991	0.976	7.42E-236	NPPC	DCN
7.99E-239	0.68949773	0.711	0.526	2.02E-234	NPPC	ARL6IP5

1.70E-236	0.51233821	0.241	0.07	4.29E-232	NPPC	CBLB
6.50E-231	0.63147617	0.368	0.15	1.64E-226	NPPC	MATN2
3.45E-226	0.55880685	0.238	0.072	8.71E-222	NPPC	NEGR1
8.33E-225	0.49682409	0.227	0.065	2.10E-220	NPPC	AKR1C2
5.47E-224	0.64316699	0.334	0.13	1.38E-219	NPPC	FZD8
1.17E-222	0.59685944	0.665	0.428	2.95E-218	NPPC	ZBTB20
5.11E-221	0.52260652	0.225	0.065	1.29E-216	NPPC	CMYA5
1.56E-217	0.55683977	0.354	0.144	3.93E-213	NPPC	OGT
7.16E-216	0.46493821	0.239	0.073	1.81E-211	NPPC	SLC16A7
2.84E-210	0.58309786	0.363	0.157	7.15E-206	NPPC	MPZL1
5.56E-207	0.61705127	0.366	0.158	1.40E-202	NPPC	SCARA3
2.96E-205	0.42987005	0.228	0.07	7.48E-201	NPPC	C1RL
2.95E-202	0.64013096	0.239	0.078	7.44E-198	NPPC	LRMDA
6.60E-197	0.60151361	0.445	0.225	1.66E-192	NPPC	ZIC1
1.02E-179	0.51322416	0.356	0.161	2.58E-175	NPPC	KIDINS220
6.00E-177	0.62941622	0.406	0.204	1.51E-172	NPPC	BOC
1.65E-174	0.51267454	0.384	0.185	4.17E-170	NPPC	MIR99AHG
3.32E-174	0.44606036	0.249	0.09	8.39E-170	NPPC	NT5E
8.46E-174	0.62232669	0.405	0.209	2.13E-169	NPPC	CD302
1.16E-173	0.49246289	0.215	0.069	2.92E-169	NPPC	GALNT15
8.68E-171	0.42893204	0.23	0.08	2.19E-166	NPPC	SLC43A3
3.54E-161	0.54306023	0.55	0.333	8.94E-157	NPPC	PCOLCE
3.32E-160	0.44183359	0.765	0.627	8.39E-156	NPPC	N4BP2L2
1.04E-159	0.50482836	0.277	0.114	2.61E-155	NPPC	ECHDC2
2.20E-156	0.44023581	0.235	0.086	5.55E-152	NPPC	DYNC2H1
4.77E-151	0.57104205	0.326	0.153	1.20E-146	NPPC	DSE
1.35E-149	0.34771093	0.204	0.069	3.40E-145	NPPC	ANK2
4.52E-147	0.50932765	0.329	0.156	1.14E-142	NPPC	ZNF704
7.56E-146	0.51117917	0.351	0.177	1.91E-141	NPPC	SNX9
1.41E-141	0.54306447	0.516	0.338	3.55E-137	NPPC	ASPH
4.93E-136	0.50217484	0.306	0.144	1.25E-131	NPPC	CAMK2D
8.83E-135	0.57120989	0.53	0.358	2.23E-130	NPPC	SSPN
4.31E-128	0.54840951	0.392	0.23	1.09E-123	NPPC	VEGFB
6.61E-120	0.51338554	0.372	0.211	1.67E-115	NPPC	ZNF106
8.86E-120	0.45163742	0.267	0.122	2.24E-115	NPPC	CDON
8.98E-120	0.41971001	0.227	0.094	2.27E-115	NPPC	WIPF1
8.94E-116	0.44317706	0.31	0.157	2.26E-111	NPPC	NOTCH2
8.11E-114	0.42096722	0.311	0.159	2.05E-109	NPPC	PKD2
2.07E-113	0.40128479	0.255	0.116	5.21E-109	NPPC	MUM1
2.28E-113	0.39888212	0.226	0.096	5.74E-109	NPPC	LRRN4CL
1.54E-111	0.42185678	0.248	0.112	3.87E-107	NPPC	SMAD9
3.72E-111	0.44966787	0.371	0.215	9.40E-107	NPPC	STAG2
8.41E-111	0.45116655	0.439	0.278	2.12E-106	NPPC	TBL1XR1
1.08E-109	0.37343507	0.247	0.113	2.73E-105	NPPC	SH3D19
3.69E-109	0.43186752	0.285	0.142	9.30E-105	NPPC	OIP5-AS1
8.66E-108	0.44915872	0.55	0.401	2.19E-103	NPPC	MPHOSPH8
1.22E-106	0.40785418	0.278	0.139	3.09E-102	NPPC	GLT8D2
2.17E-104	0.45452961	0.569	0.439	5.47E-100	NPPC	SERINC1
3.28E-101	0.33973804	0.205	0.087	8.28E-97	NPPC	HCG11
5.49E-101	0.35304137	0.212	0.092	1.39E-96	NPPC	DYNC2LI1
3.61E-100	0.46047355	0.444	0.296	9.11E-96	NPPC	PHF3

9.05E-100	0.42935027	0.459	0.306	2.28E-95	NPPC	CREBRF
5.10E-99	0.42075262	0.597	0.462	1.29E-94	NPPC	LUC7L3
1.76E-98	0.36160735	0.244	0.115	4.44E-94	NPPC	NPEPPS
7.82E-98	0.45130037	0.705	0.585	1.97E-93	NPPC	CEBPD
3.00E-97	0.44119664	0.283	0.148	7.58E-93	NPPC	KIF22
4.07E-97	0.42969344	0.334	0.189	1.03E-92	NPPC	SLC44A1
4.00E-95	0.37180548	0.221	0.1	1.01E-90	NPPC	ZNF83
8.25E-91	0.37428143	0.262	0.132	2.08E-86	NPPC	NF1
8.90E-91	0.47858349	0.205	0.093	2.25E-86	NPPC	HTRA3
7.12E-90	0.43128842	0.359	0.222	1.80E-85	NPPC	SMIM14
1.29E-89	0.37480077	0.221	0.103	3.24E-85	NPPC	MLH3
4.52E-89	0.45359612	0.402	0.268	1.14E-84	NPPC	ANXA4
3.43E-88	0.45474176	0.421	0.289	8.65E-84	NPPC	CPNE3
8.53E-87	0.43393351	0.527	0.386	2.15E-82	NPPC	TENT5A
2.03E-86	0.40659172	0.579	0.453	5.12E-82	NPPC	NFIC
9.85E-86	0.36686384	0.208	0.096	2.49E-81	NPPC	NHLRC3
1.29E-85	0.49746609	0.307	0.178	3.26E-81	NPPC	RETREG1
4.99E-81	0.34550808	0.243	0.125	1.26E-76	NPPC	PIAS1
5.27E-81	0.38246045	0.261	0.141	1.33E-76	NPPC	TFDP2
3.36E-80	0.36667208	0.243	0.125	8.49E-76	NPPC	CCNG2
7.15E-80	0.38808769	0.284	0.159	1.81E-75	NPPC	LNPEP
7.90E-80	0.3264763	0.21	0.1	1.99E-75	NPPC	LRP6
1.10E-76	0.37551466	0.4	0.27	2.77E-72	NPPC	PPFIBP1
3.56E-75	0.32029239	0.215	0.107	8.98E-71	NPPC	CLK4
1.44E-74	0.39839822	0.539	0.423	3.64E-70	NPPC	PPP3CA
3.92E-73	0.38623251	0.408	0.285	9.88E-69	NPPC	SCARB2
1.94E-72	0.38104185	0.252	0.138	4.91E-68	NPPC	SLC4A7
1.15E-71	0.36456422	0.538	0.419	2.90E-67	NPPC	GOLIM4
9.56E-71	0.3916403	0.451	0.335	2.41E-66	NPPC	LMAN1
2.06E-70	0.2855043	0.751	0.679	5.20E-66	NPPC	SON
4.00E-70	0.35008015	0.239	0.127	1.01E-65	NPPC	AFF3
1.86E-69	0.35787942	0.452	0.331	4.69E-65	NPPC	GLG1
2.23E-69	0.33771943	0.291	0.171	5.64E-65	NPPC	ATM
2.83E-68	0.41662211	0.377	0.26	7.14E-64	NPPC	TSPAN4
5.89E-68	0.39083083	0.343	0.228	1.49E-63	NPPC	BDH2
1.00E-67	0.30156315	0.713	0.617	2.53E-63	NPPC	SRRM2
1.09E-67	0.30227944	0.227	0.119	2.75E-63	NPPC	PRKAG2
1.10E-65	0.32914456	0.279	0.164	2.77E-61	NPPC	KANSL1
2.30E-65	0.34618137	0.258	0.147	5.80E-61	NPPC	MKLN1
7.46E-65	0.37445345	0.314	0.202	1.88E-60	NPPC	RTN3
1.43E-64	0.43787392	0.275	0.16	3.61E-60	NPPC	LOX
7.61E-64	0.35797553	0.211	0.113	1.92E-59	NPPC	RNF146
9.32E-64	0.55002368	0.435	0.324	2.35E-59	NPPC	CDO1
3.57E-61	0.39503473	0.417	0.309	9.00E-57	NPPC	NFIX
1.73E-58	0.3869629	0.329	0.223	4.37E-54	NPPC	SGCB
6.47E-58	0.31506177	0.23	0.132	1.63E-53	NPPC	SNX2
7.42E-58	0.31927328	0.243	0.14	1.87E-53	NPPC	RNPC3
8.11E-58	0.3101851	0.245	0.145	2.05E-53	NPPC	TMBIM1
1.74E-57	0.28252256	0.207	0.111	4.39E-53	NPPC	PHC3
5.48E-57	0.35055588	0.289	0.183	1.38E-52	NPPC	TTC14
8.10E-57	0.32039562	0.26	0.158	2.04E-52	NPPC	ESYT2

1.92E-56	0.35112551	0.296	0.19	4.84E-52	NPPC	GATAD1
3.33E-54	0.30322719	0.238	0.14	8.39E-50	NPPC	FBXL3
3.90E-54	0.31460919	0.21	0.119	9.83E-50	NPPC	CCNDBP1
1.12E-52	0.37473261	0.309	0.209	2.83E-48	NPPC	FOXN3
4.46E-52	0.30170543	0.227	0.133	1.13E-47	NPPC	RNF115
1.34E-51	0.3631544	0.442	0.358	3.37E-47	NPPC	NDFIP1
2.58E-51	0.3136561	0.524	0.435	6.50E-47	NPPC	ANKRD12
5.04E-51	0.32115986	0.242	0.145	1.27E-46	NPPC	SIPA1L1
6.12E-50	0.31249549	0.215	0.123	1.54E-45	NPPC	ECM1
1.45E-49	0.26949748	0.217	0.125	3.65E-45	NPPC	CREBZF
2.29E-49	0.32868306	0.22	0.129	5.77E-45	NPPC	ZC3H6
2.78E-49	0.36109827	0.417	0.327	7.00E-45	NPPC	MAGI2-AS3
3.69E-49	0.30087447	0.208	0.121	9.32E-45	NPPC	PARVA
1.97E-48	0.29650417	0.235	0.143	4.98E-44	NPPC	MAPK10
4.24E-47	0.27179391	0.214	0.126	1.07E-42	NPPC	STX16
3.03E-46	0.27422949	0.51	0.418	7.64E-42	NPPC	IDS
3.89E-46	0.32622733	0.38	0.291	9.82E-42	NPPC	CD47
6.39E-46	0.29356395	0.28	0.186	1.61E-41	NPPC	SESTD1
1.34E-45	0.33663673	0.323	0.232	3.37E-41	NPPC	DHRS7
6.58E-45	0.29570682	0.21	0.126	1.66E-40	NPPC	LNPK
7.07E-45	0.51594902	0.241	0.144	1.78E-40	NPPC	PRG4
1.23E-43	0.3267508	0.33	0.24	3.10E-39	NPPC	NSD3
6.13E-43	0.3132322	0.315	0.221	1.55E-38	NPPC	IL1R1
6.68E-43	0.2655402	0.481	0.388	1.69E-38	NPPC	RSRP1
5.43E-42	0.26509079	0.203	0.121	1.37E-37	NPPC	LRRC58
1.88E-41	0.25938898	0.767	0.681	4.75E-37	NPPC	PLAC9
2.49E-41	0.34397928	0.234	0.152	6.29E-37	NPPC	DHRS3
3.80E-41	0.30572995	0.228	0.146	9.58E-37	NPPC	SETD7
7.34E-41	0.28979292	0.204	0.124	1.85E-36	NPPC	ALDH6A1
1.80E-40	0.31702769	0.254	0.17	4.53E-36	NPPC	LMBRD1
4.23E-40	0.27949265	0.201	0.123	1.07E-35	NPPC	ALDH2
1.04E-39	0.29976738	0.438	0.351	2.61E-35	NPPC	CPQ
1.26E-39	0.3150738	0.283	0.198	3.17E-35	NPPC	TIAL1
5.91E-38	0.3330822	0.348	0.27	1.49E-33	NPPC	CTTN
9.76E-37	0.39015167	0.31	0.234	2.46E-32	NPPC	OAF
2.33E-36	0.28970332	0.332	0.25	5.89E-32	NPPC	SNRNP70
2.91E-36	0.35929791	0.255	0.177	7.35E-32	NPPC	RNF24
2.03E-34	0.26149293	0.219	0.143	5.12E-30	NPPC	OSBPL9
2.12E-34	0.28150548	0.248	0.171	5.36E-30	NPPC	VPS4B
6.64E-34	0.26694378	0.21	0.137	1.68E-29	NPPC	CDC40
4.34E-33	0.28394491	0.244	0.17	1.10E-28	NPPC	CTDSP2
6.20E-32	0.30164706	0.374	0.303	1.56E-27	NPPC	GCC2
7.75E-32	0.29277855	0.38	0.317	1.96E-27	NPPC	GINM1
1.30E-31	0.25066849	0.208	0.136	3.27E-27	NPPC	POGZ
1.48E-31	0.30838056	0.256	0.183	3.73E-27	NPPC	ZKSCAN1
1.52E-31	0.2700445	0.311	0.233	3.84E-27	NPPC	ZNF292
5.64E-31	0.26407159	0.364	0.291	1.42E-26	NPPC	CTSF
3.05E-30	0.29700054	0.32	0.25	7.69E-26	NPPC	SFT2D2
4.49E-30	0.25265999	0.215	0.145	1.13E-25	NPPC	MYO9A
6.35E-30	0.27110145	0.339	0.272	1.60E-25	NPPC	LRP10
2.45E-29	0.2976429	0.244	0.175	6.18E-25	NPPC	GPATCH8

2.78E-29	0.27227081	0.283	0.213	7.03E-25	NPPC	MBTPS1
6.04E-29	0.25705864	0.246	0.174	1.52E-24	NPPC	KLHL24
6.09E-29	0.28848734	0.336	0.268	1.54E-24	NPPC	FOXO3
1.25E-28	0.29311446	0.328	0.26	3.16E-24	NPPC	TCF7L2
2.40E-28	0.25083695	0.246	0.176	6.06E-24	NPPC	AGO2
3.53E-28	0.25022312	0.255	0.186	8.91E-24	NPPC	ATG12
7.98E-28	0.27518009	0.261	0.193	2.01E-23	NPPC	YIPF4
1.76E-27	0.25071673	0.534	0.493	4.44E-23	NPPC	UBE2B
1.82E-27	0.25605414	0.283	0.214	4.58E-23	NPPC	TRIP11
6.12E-26	0.25179274	0.326	0.265	1.54E-21	NPPC	TMEM87A
2.38E-25	0.25102774	0.419	0.365	6.01E-21	NPPC	PJA2
1.68E-24	0.26454853	0.258	0.196	4.24E-20	NPPC	CTSA
3.73E-23	0.27575319	0.27	0.214	9.42E-19	NPPC	MPG
4.08E-21	0.2587853	0.243	0.187	1.03E-16	NPPC	TMEM106B
2.14E-20	0.25434965	0.311	0.258	5.40E-16	NPPC	CFAP97
3.29E-20	0.41838244	0.349	0.307	8.31E-16	NPPC	MAF
3.99E-19	0.25707244	0.267	0.216	1.01E-14	NPPC	ADI1
9.61E-19	0.25044	0.291	0.242	2.42E-14	NPPC	PRMT2
2.62E-18	0.25712414	0.242	0.192	6.61E-14	NPPC	GPX8
5.58E-18	0.25595002	0.265	0.217	1.41E-13	NPPC	ANG
4.97E-12	0.3132838	0.618	0.627	1.25E-07	NPPC	MFGE8
0	0.99490961	0.259	0.086	0	Stroma	PRSS23
0	0.91504814	0.239	0.047	0	Stroma	RUNX2
0	0.86091691	0.282	0.102	0	Stroma	GJA1
0	0.85177291	0.314	0.116	0	Stroma	MAP1B
0	0.78276647	0.212	0.041	0	Stroma	HES4
0	0.56974129	0.948	0.936	0	Stroma	RPL39
8.56E-285	0.79052993	0.268	0.099	2.16E-280	Stroma	FRMD6
1.49E-277	1.0186792	0.229	0.078	3.76E-273	Stroma	PLEKHA5
1.76E-188	0.8016451	0.28	0.133	4.44E-184	Stroma	MMP14
4.34E-131	0.47311264	0.201	0.09	1.09E-126	Stroma	CLMP
1.45E-111	0.50900798	0.24	0.127	3.67E-107	Stroma	B4GALT1
2.24E-71	0.48066299	0.22	0.132	5.66E-67	Stroma	COL5A1
1.60E-70	0.7136795	0.573	0.555	4.04E-66	Stroma	CTNNB1
1.11E-24	0.50149858	0.365	0.354	2.81E-20	Stroma	ATP5MF
7.94E-21	0.52797926	0.31	0.274	2.00E-16	Stroma	RND3
9.73E-21	0.44758174	0.205	0.162	2.46E-16	Stroma	LSP1
7.95E-18	0.63652365	0.546	0.613	2.01E-13	Stroma	CEBPB
1.07E-17	0.53093182	0.246	0.21	2.70E-13	Stroma	FOXC2
5.63E-14	0.28297805	0.306	0.414	1.42E-09	Stroma	MEF2A
3.78E-08	0.32960709	0.599	0.69	0.0009527	Stroma	HNRNPH1
9.82E-08	0.29859015	0.21	0.189	0.0024774	Stroma	EMILIN1
5.90E-07	0.37202015	0.313	0.407	0.0149005	Stroma	ANKH
0	2.80127112	0.739	0.022	0	Pericyte	ACTA2
0	2.76946851	0.911	0.135	0	Pericyte	MYL9
0	2.67365453	0.603	0.016	0	Pericyte	RGS5
0	2.10980329	0.58	0.017	0	Pericyte	MYH11
0	1.772602	0.637	0.016	0	Pericyte	SYNPO2
0	1.75707466	0.474	0.027	0	Pericyte	STEAP4
0	1.7444572	0.816	0.015	0	Pericyte	NOTCH3
0	1.73146826	0.75	0.02	0	Pericyte	MCAM

0	1.72569823	0.972	0.608	0	Pericyte	ADIRF
0	1.68036886	0.906	0.499	0	Pericyte	TPM2
0	1.63088376	0.592	0.005	0	Pericyte	LMOD1
0	1.60775096	0.851	0.353	0	Pericyte	TPM1
0	1.46989398	0.627	0.008	0	Pericyte	PPP1R14A
0	1.4322946	0.821	0.125	0	Pericyte	EPAS1
0	1.42958581	0.695	0.019	0	Pericyte	TINAGL1
0	1.38779879	0.449	0.004	0	Pericyte	PLN
0	1.36812875	0.619	0.05	0	Pericyte	FILIP1L
0	1.32157608	0.686	0.015	0	Pericyte	EDNRA
0	1.3120977	0.751	0.113	0	Pericyte	JAG1
0	1.30660532	0.368	0.061	0	Pericyte	CCL2
0	1.27164118	0.559	0.073	0	Pericyte	SORBS2
0	1.26799802	0.651	0.062	0	Pericyte	COL18A1
0	1.24980854	0.836	0.338	0	Pericyte	LPP
0	1.23725476	0.676	0.144	0	Pericyte	FLNA
0	1.23313729	0.826	0.498	0	Pericyte	TIMP3
0	1.21608193	0.872	0.431	0	Pericyte	CAV1
0	1.18103055	0.408	0.017	0	Pericyte	FABP4
0	1.16029191	0.578	0.155	0	Pericyte	ADAMTS1
0	1.14197922	0.747	0.27	0	Pericyte	TPM4
0	1.13947672	0.924	0.568	0	Pericyte	7-Sep
0	1.13658145	0.768	0.227	0	Pericyte	EPS8
0	1.13084189	0.598	0.124	0	Pericyte	CRIP1
0	1.12951711	0.722	0.189	0	Pericyte	PPP1R12A
0	1.1212576	0.624	0.153	0	Pericyte	MYLK
0	1.09731181	0.721	0.139	0	Pericyte	MYH9
0	1.09519272	0.584	0.127	0	Pericyte	IFITM1
0	1.08369019	0.887	0.468	0	Pericyte	ITGB1
0	1.07334096	0.425	0.005	0	Pericyte	CARMN
0	1.05708264	0.779	0.419	0	Pericyte	ID3
0	1.03395935	0.591	0.041	0	Pericyte	C1QTNF1
0	1.03085396	0.958	0.752	0	Pericyte	MYL6
0	1.02281974	0.486	0.012	0	Pericyte	RCAN2
0	1.01157328	0.633	0.095	0	Pericyte	MAP3K20
0	1.01122932	0.636	0.091	0	Pericyte	TNS1
0	1.00011691	0.497	0.06	0	Pericyte	LGI4
0	0.99586213	0.422	0.01	0	Pericyte	ABCC9
0	0.9938538	0.557	0.071	0	Pericyte	PDGFA
0	0.9923157	0.623	0.12	0	Pericyte	PALLD
0	0.98283225	0.427	0.033	0	Pericyte	SNCG
0	0.98231209	0.711	0.194	0	Pericyte	STOM
0	0.97133706	0.731	0.315	0	Pericyte	ARID5B
0	0.96801085	0.412	0.114	0	Pericyte	PDK4
0	0.96639593	0.443	0.005	0	Pericyte	TBX2
0	0.96002128	0.63	0.087	0	Pericyte	KANK2
0	0.938273	0.361	0.013	0	Pericyte	PLAU
0	0.93810332	0.594	0.248	0	Pericyte	CSRP1
0	0.93058357	0.481	0.011	0	Pericyte	PDE5A
0	0.92016894	0.492	0.017	0	Pericyte	SLIT3
0	0.91715449	0.963	0.853	0	Pericyte	ACTB

0	0.9005269	0.477	0.026	0	Pericyte	MYO1B
0	0.89910153	0.42	0.024	0	Pericyte	ANGPT2
0	0.88901342	0.476	0.122	0	Pericyte	CSRP2
0	0.88299322	0.72	0.278	0	Pericyte	TLN1
0	0.88142774	0.326	0.014	0	Pericyte	ADAMTS4
0	0.87938213	0.377	0.021	0	Pericyte	KCNE4
0	0.87507969	0.767	0.399	0	Pericyte	CAVIN1
0	0.85336875	0.528	0.034	0	Pericyte	RBPMS
0	0.8529209	0.676	0.247	0	Pericyte	CHCHD10
0	0.84268897	0.399	0.019	0	Pericyte	ADGRF5
0	0.83819846	0.548	0.049	0	Pericyte	PDLIM1
0	0.82876082	0.334	0.003	0	Pericyte	CNN1
0	0.82613784	0.766	0.528	0	Pericyte	ID4
0	0.81895774	0.401	0.017	0	Pericyte	FAM13C
0	0.81022622	0.461	0.008	0	Pericyte	GUCY1B1
0	0.80997953	0.486	0.025	0	Pericyte	PHLDB2
0	0.80948177	0.378	0.038	0	Pericyte	SSTR2
0	0.80875233	0.383	0.006	0	Pericyte	GJA4
0	0.80695931	0.619	0.171	0	Pericyte	VCL
0	0.80680717	0.396	0.051	0	Pericyte	ANGPTL1
0	0.79764155	0.45	0.067	0	Pericyte	SLC7A2
0	0.79746276	0.392	0.004	0	Pericyte	ITGA7
0	0.79267829	0.681	0.225	0	Pericyte	JAK1
0	0.7871673	0.272	0.002	0	Pericyte	ACTG2
0	0.78482007	0.645	0.277	0	Pericyte	CD44
0	0.78469651	0.824	0.507	0	Pericyte	MGST3
0	0.78108389	0.371	0.023	0	Pericyte	NEXN
0	0.77505778	0.728	0.317	0	Pericyte	YWHAB
0	0.77181345	0.506	0.077	0	Pericyte	INAFM1
0	0.76826707	0.296	0.035	0	Pericyte	CLSTN2
0	0.76705717	0.673	0.36	0	Pericyte	CAVIN3
0	0.76366529	0.524	0.079	0	Pericyte	EHD2
0	0.76037948	0.999	0.958	0	Pericyte	PTMA
0	0.75853715	0.466	0.062	0	Pericyte	ITGA1
0	0.75692504	0.57	0.149	0	Pericyte	UBA2
0	0.75114658	0.64	0.218	0	Pericyte	PSME1
0	0.74967287	0.799	0.482	0	Pericyte	PFN1
0	0.74892986	0.38	0.038	0	Pericyte	PGF
0	0.74850513	0.427	0.014	0	Pericyte	PRKG1
0	0.74483254	0.505	0.077	0	Pericyte	TGFB1I1
0	0.74122242	0.422	0.04	0	Pericyte	MRGPRF
0	0.73636455	0.412	0.124	0	Pericyte	CCDC102B
0	0.73279269	0.42	0.029	0	Pericyte	ANGPT1
0	0.72865722	0.343	0.006	0	Pericyte	ADRA2A
0	0.72856974	0.367	0.015	0	Pericyte	GJC1
0	0.72791094	0.386	0.005	0	Pericyte	MRVI1
0	0.72037819	0.673	0.28	0	Pericyte	ARPC5
0	0.70710581	0.64	0.254	0	Pericyte	ROCK1
0	0.70664628	0.407	0.01	0	Pericyte	PDE1A
0	0.70064451	0.415	0.007	0	Pericyte	EBF2
0	0.69993382	0.471	0.093	0	Pericyte	TGFBI

0	0.69644351	0.625	0.247	0	Pericyte	ACTN4
0	0.69501796	0.365	0.036	0	Pericyte	BCAM
0	0.69480615	0.479	0.097	0	Pericyte	MSRB3
0	0.69068465	0.45	0.11	0	Pericyte	CRISPLD2
0	0.68474477	0.567	0.146	0	Pericyte	ROCK2
0	0.68440448	0.572	0.178	0	Pericyte	UTRN
0	0.68107643	0.409	0.138	0	Pericyte	CYTOR
0	0.68087585	0.692	0.362	0	Pericyte	SH3BGRL
0	0.68026462	0.51	0.135	0	Pericyte	MAP7D3
0	0.67921621	0.556	0.154	0	Pericyte	CAP1
0	0.6791197	0.904	0.643	0	Pericyte	PTMS
0	0.67879768	0.42	0.088	0	Pericyte	SLC2A3
0	0.67726638	0.549	0.172	0	Pericyte	SORBS3
0	0.6679418	0.576	0.206	0	Pericyte	ENAH
0	0.66625537	0.422	0.161	0	Pericyte	LBH
0	0.66055953	0.419	0.047	0	Pericyte	ARHGEF17
0	0.65599626	0.317	0.018	0	Pericyte	PTP4A3
0	0.64642367	0.678	0.343	0	Pericyte	CD151
0	0.64598075	0.308	0.004	0	Pericyte	SEMA5A
0	0.64590448	0.62	0.31	0	Pericyte	ZFHX3
0	0.64346366	0.372	0.025	0	Pericyte	AOC3
0	0.64260207	0.369	0.049	0	Pericyte	PPP1R12B
0	0.64206345	0.444	0.065	0	Pericyte	PAWR
0	0.63932382	0.805	0.525	0	Pericyte	SELENOW
0	0.63929533	0.384	0.062	0	Pericyte	PLEKHO1
0	0.63419082	0.44	0.103	0	Pericyte	SYNE2
0	0.63326693	0.367	0.016	0	Pericyte	SGIP1
0	0.63243947	0.449	0.075	0	Pericyte	MGLL
0	0.62720033	0.338	0.025	0	Pericyte	CCDC3
0	0.62714527	0.482	0.175	0	Pericyte	CAMK2N1
0	0.62487934	0.361	0.02	0	Pericyte	SYTL2
0	0.6234811	0.375	0.015	0	Pericyte	RERG
0	0.62333076	0.399	0.023	0	Pericyte	ESAM
0	0.62035439	0.622	0.282	0	Pericyte	RRAS
0	0.61762423	0.597	0.275	0	Pericyte	MEF2C
0	0.61579434	0.359	0.025	0	Pericyte	INPP4B
0	0.61513491	0.681	0.355	0	Pericyte	RAB13
0	0.61434659	0.292	0.093	0	Pericyte	C12orf75
0	0.61247167	0.642	0.282	0	Pericyte	TMEM50A
0	0.60576743	0.336	0.033	0	Pericyte	TPPP3
0	0.60362374	0.277	0.003	0	Pericyte	HRH2
0	0.5980742	0.468	0.115	0	Pericyte	AXL
0	0.59586834	0.916	0.785	0	Pericyte	MT-ND1
0	0.59198261	0.457	0.133	0	Pericyte	OAZ2
0	0.59011314	0.368	0.043	0	Pericyte	AHR
0	0.58810057	0.385	0.108	0	Pericyte	ARHGDIB
0	0.58414745	0.48	0.155	0	Pericyte	ZNF503
0	0.58216311	0.324	0.006	0	Pericyte	LINC01197
0	0.58174303	0.385	0.151	0	Pericyte	PHLDA1
0	0.57796283	0.587	0.27	0	Pericyte	ACTN1
0	0.57751925	0.316	0.034	0	Pericyte	OLFML2B

0	0.56980906	0.447	0.153	0	Pericyte	HSPB6
0	0.56867479	0.401	0.051	0	Pericyte	ADCY3
0	0.56755594	0.523	0.2	0	Pericyte	ARHGAP29
0	0.56346613	0.335	0.005	0	Pericyte	PARM1
0	0.56239633	0.992	0.922	0	Pericyte	B2M
0	0.56190562	0.324	0.012	0	Pericyte	LRRC32
0	0.56003691	0.526	0.216	0	Pericyte	VMP1
0	0.55615977	0.219	0.002	0	Pericyte	ITGA8
0	0.55536751	0.767	0.5	0	Pericyte	MYL12A
0	0.55398237	0.477	0.129	0	Pericyte	MPRIP
0	0.55380082	0.569	0.221	0	Pericyte	CAPZB
0	0.5534214	0.265	0.001	0	Pericyte	AVPR1A
0	0.55321455	0.582	0.258	0	Pericyte	CAV2
0	0.5511266	0.301	0.002	0	Pericyte	CDH6
0	0.54851683	0.799	0.55	0	Pericyte	COX6C
0	0.54577767	0.27	0.022	0	Pericyte	KCNAB1
0	0.54475496	0.564	0.247	0	Pericyte	NFE2L2
0	0.54238317	0.462	0.113	0	Pericyte	EPN2
0	0.5381193	0.963	0.862	0	Pericyte	MT-CO1
0	0.53726525	0.519	0.181	0	Pericyte	PTEN
0	0.53702947	0.342	0.098	0	Pericyte	ANGPTL4
0	0.53570418	0.392	0.06	0	Pericyte	SH3PXD2A
0	0.53443389	0.495	0.186	0	Pericyte	GLUL
0	0.53096924	0.297	0.009	0	Pericyte	NEURL1B
0	0.52815033	0.821	0.565	0	Pericyte	SLC25A6
0	0.52642992	0.234	0.001	0	Pericyte	CASQ2
0	0.52589982	0.542	0.188	0	Pericyte	TACC1
0	0.52576458	0.448	0.116	0	Pericyte	RARRES2
0	0.52190083	0.793	0.533	0	Pericyte	COL6A1
0	0.52054806	0.338	0.069	0	Pericyte	FILIP1
0	0.51877115	0.334	0.053	0	Pericyte	SORBS1
0	0.51537544	0.33	0.061	0	Pericyte	RABGAP1
0	0.51240666	0.436	0.122	0	Pericyte	ILK
0	0.51175415	0.327	0.019	0	Pericyte	PDE3A
0	0.51060521	0.411	0.109	0	Pericyte	REV3L
0	0.50757191	0.313	0.009	0	Pericyte	ARHGAP6
0	0.50373094	0.355	0.089	0	Pericyte	B3GNT2
0	0.50324513	0.322	0.043	0	Pericyte	PEAK1
0	0.5022779	0.326	0.075	0	Pericyte	ATP1B2
0	0.50201805	0.668	0.386	0	Pericyte	COX7A1
0	0.50188304	0.635	0.314	0	Pericyte	ESD
0	0.50014154	0.399	0.162	0	Pericyte	ITIH5
0	0.50000028	0.282	0.02	0	Pericyte	CLMN
0	0.49995766	0.253	0.063	0	Pericyte	HBA2
0	0.49962148	0.289	0.05	0	Pericyte	WTIP
0	0.49945873	0.351	0.064	0	Pericyte	MAP1A
0	0.49895648	0.669	0.359	0	Pericyte	WASF2
0	0.49834596	0.624	0.347	0	Pericyte	SLC25A5
0	0.49783565	0.508	0.192	0	Pericyte	ZBTB38
0	0.49542211	0.303	0.023	0	Pericyte	SMARCD3
0	0.49438464	0.835	0.59	0	Pericyte	RAC1

0	0.49371854	0.453	0.153	0	Pericyte	RAB31
0	0.49318974	0.435	0.126	0	Pericyte	COBLL1
0	0.49298816	0.47	0.149	0	Pericyte	CBX6
0	0.49288575	0.84	0.615	0	Pericyte	CFL1
0	0.49257283	0.393	0.071	0	Pericyte	RSU1
0	0.49183779	0.32	0.057	0	Pericyte	ISYNA1
0	0.49168693	0.518	0.234	0	Pericyte	FKBP1A
0	0.49079106	0.472	0.153	0	Pericyte	ARHGAP10
0	0.48954829	0.47	0.202	0	Pericyte	SERPINH1
0	0.48860923	0.647	0.363	0	Pericyte	PA2G4
0	0.48668055	0.333	0.072	0	Pericyte	4-Sep
0	0.48461721	0.327	0.026	0	Pericyte	C1orf54
0	0.48444002	0.253	0.014	0	Pericyte	ANKRD29
0	0.48056119	0.355	0.053	0	Pericyte	ECE1
0	0.47886467	0.424	0.124	0	Pericyte	ATP2B4
0	0.47635205	0.759	0.513	0	Pericyte	CLIC1
0	0.47586652	0.506	0.171	0	Pericyte	DYNC1LI2
0	0.47378794	0.554	0.291	0	Pericyte	RHOQ
0	0.47313712	0.325	0.061	0	Pericyte	HEY2
0	0.47310058	0.256	0.014	0	Pericyte	ADGRA2
0	0.47150466	0.26	0.004	0	Pericyte	NR2F2-AS1
0	0.47006372	0.889	0.661	0	Pericyte	NUCKS1
0	0.46691711	0.418	0.129	0	Pericyte	PRRX2
0	0.4667338	0.218	0.01	0	Pericyte	NRGN
0	0.46580229	0.252	0.006	0	Pericyte	DMD
0	0.46516261	0.31	0.024	0	Pericyte	ARHGEF25
0	0.46498184	0.605	0.311	0	Pericyte	ATP5F1B
0	0.46434389	0.427	0.143	0	Pericyte	PTK2
0	0.46415711	0.335	0.095	0	Pericyte	FBXO32
0	0.46366604	0.296	0.03	0	Pericyte	APOL6
0	0.4591133	0.316	0.038	0	Pericyte	MKL2
0	0.45732532	0.277	0.029	0	Pericyte	NID1
0	0.45267442	0.277	0.009	0	Pericyte	ANO1
0	0.45010461	0.576	0.265	0	Pericyte	2-Sep
0	0.44719441	0.728	0.367	0	Pericyte	A2M
0	0.44537671	0.277	0.06	0	Pericyte	KLHL23
0	0.44202663	0.371	0.093	0	Pericyte	PDLIM7
0	0.44162698	0.378	0.091	0	Pericyte	ARHGAP1
0	0.44047076	0.306	0.076	0	Pericyte	C20orf27
0	0.43853008	0.491	0.224	0	Pericyte	NUDT4
0	0.43701792	0.936	0.807	0	Pericyte	RPL27
0	0.43502859	0.283	0.046	0	Pericyte	AKAP1
0	0.43424146	0.302	0.061	0	Pericyte	FAM129A
0	0.43405674	0.298	0.046	0	Pericyte	ARHGEF7
0	0.43381671	0.458	0.189	0	Pericyte	GLRX5
0	0.43109162	0.386	0.144	0	Pericyte	SLC40A1
0	0.43053499	0.474	0.177	0	Pericyte	C5orf24
0	0.42998374	0.339	0.102	0	Pericyte	NT5DC2
0	0.42953618	0.437	0.157	0	Pericyte	WDR1
0	0.42943587	0.254	0.055	0	Pericyte	MIR4435-2HG
0	0.42924543	0.314	0.041	0	Pericyte	C16orf45

0	0.42843627	0.513	0.243	0	Pericyte	BBX
0	0.4283019	0.492	0.21	0	Pericyte	RBMS1
0	0.42749823	0.476	0.201	0	Pericyte	EFHD1
0	0.42699025	0.637	0.363	0	Pericyte	MINOS1
0	0.42579001	0.302	0.054	0	Pericyte	ARHGAP42
0	0.42460488	0.548	0.264	0	Pericyte	KMT2A
0	0.42155815	0.56	0.297	0	Pericyte	AP2M1
0	0.42138431	0.467	0.197	0	Pericyte	HIGD2A
0	0.42128215	0.541	0.252	0	Pericyte	DYNC1I2
0	0.42048435	0.415	0.146	0	Pericyte	PDLIM3
0	0.42015681	0.295	0.093	0	Pericyte	FAM213A
0	0.41555318	0.449	0.188	0	Pericyte	OPTN
0	0.41538641	0.244	0.002	0	Pericyte	FAM162B
0	0.41336605	0.55	0.281	0	Pericyte	KIF5B
0	0.41270555	0.43	0.163	0	Pericyte	SP100
0	0.41212577	0.307	0.064	0	Pericyte	RASL11A
0	0.40514172	0.236	0.005	0	Pericyte	VGLL3
0	0.40427022	0.255	0.006	0	Pericyte	FBLIM1
0	0.40312601	0.47	0.186	0	Pericyte	BLOC1S1
0	0.40243308	0.408	0.149	0	Pericyte	APEX1
0	0.39771708	0.313	0.07	0	Pericyte	TTLL7
0	0.39694996	0.46	0.178	0	Pericyte	ANXA11
0	0.39575783	0.315	0.064	0	Pericyte	NAB1
0	0.39446796	0.456	0.195	0	Pericyte	TNFRSF1A
0	0.3941211	0.378	0.137	0	Pericyte	NBEAL1
0	0.39132999	0.39	0.125	0	Pericyte	ZYX
0	0.39108097	0.942	0.798	0	Pericyte	HMGB1
0	0.39105107	0.218	0.011	0	Pericyte	SCN4B
0	0.38940916	0.474	0.214	0	Pericyte	PKIG
0	0.38905598	0.205	0.005	0	Pericyte	AC093908.1
0	0.38890291	0.317	0.069	0	Pericyte	PLEKHA4
0	0.38418749	0.501	0.238	0	Pericyte	NDUFA12
0	0.38371609	0.287	0.048	0	Pericyte	CSF1
0	0.38360198	0.3	0.099	0	Pericyte	FHL2
0	0.38221482	0.271	0.055	0	Pericyte	SORT1
0	0.38194656	0.309	0.094	0	Pericyte	SELENBP1
0	0.37926197	0.337	0.105	0	Pericyte	ASAP1
0	0.37789922	0.339	0.1	0	Pericyte	ANXA6
0	0.37534349	0.35	0.103	0	Pericyte	TNFSF12
0	0.37343073	0.37	0.135	0	Pericyte	PPP2R5E
0	0.37315991	0.303	0.064	0	Pericyte	SNX18
0	0.37305742	0.377	0.138	0	Pericyte	EEA1
0	0.37160581	0.219	0.012	0	Pericyte	SUSD2
0	0.36665511	0.287	0.084	0	Pericyte	TMEM159
0	0.36531862	0.263	0.013	0	Pericyte	GLIPR2
0	0.36531755	0.335	0.091	0	Pericyte	PLXND1
0	0.3648297	0.372	0.12	0	Pericyte	TMOD3
0	0.36443022	0.207	0.007	0	Pericyte	KCNJ8
0	0.36235848	0.265	0.048	0	Pericyte	DIAPH2
0	0.36228037	0.342	0.101	0	Pericyte	IFNAR1
0	0.35941086	0.293	0.066	0	Pericyte	ANO6

0	0.35875339	0.242	0.022	0	Pericyte	NFATC4
0	0.35819019	0.346	0.12	0	Pericyte	CDKN1B
0	0.35756514	0.256	0.028	0	Pericyte	KIRREL1
0	0.3573556	0.957	0.866	0	Pericyte	RPL27A
0	0.35552983	0.234	0.033	0	Pericyte	SPEG
0	0.35415853	0.309	0.069	0	Pericyte	FADS3
0	0.35393125	0.43	0.182	0	Pericyte	ADH5
0	0.35357752	0.227	0.022	0	Pericyte	RASL12
0	0.35233634	0.307	0.071	0	Pericyte	PCSK7
0	0.35144155	0.222	0.008	0	Pericyte	PTGIR
0	0.34807847	0.311	0.085	0	Pericyte	ARID1A
0	0.34521344	0.32	0.1	0	Pericyte	PBX1
0	0.3439523	0.211	0.051	0	Pericyte	ISG15
0	0.3422041	0.353	0.133	0	Pericyte	CSNK1E
0	0.34144308	0.217	0.019	0	Pericyte	AP000892.3
0	0.34029014	0.256	0.057	0	Pericyte	TYMP
0	0.33961385	0.408	0.167	0	Pericyte	HOOK3
0	0.337829	0.997	0.959	0	Pericyte	RPS8
0	0.33772036	0.506	0.232	0	Pericyte	MACF1
0	0.33546395	0.218	0.016	0	Pericyte	FRMD4A
0	0.3343651	0.278	0.077	0	Pericyte	FAT1
0	0.33424083	0.335	0.114	0	Pericyte	9-Sep
0	0.33410188	0.217	0.013	0	Pericyte	ITGA3
0	0.33408972	0.356	0.118	0	Pericyte	HADHB
0	0.32826014	0.369	0.136	0	Pericyte	DCTN2
0	0.32650497	0.325	0.035	0	Pericyte	AQP1
0	0.3258884	0.219	0.033	0	Pericyte	NTN4
0	0.32505244	0.361	0.126	0	Pericyte	ARHGEF12
0	0.32500064	0.248	0.037	0	Pericyte	GNB4
0	0.3244216	0.282	0.066	0	Pericyte	CABIN1
0	0.31873148	0.203	0.011	0	Pericyte	MAOA
0	0.31606818	0.235	0.046	0	Pericyte	SNRK
0	0.30720469	0.295	0.078	0	Pericyte	ENDOD1
0	0.30690547	0.434	0.183	0	Pericyte	DYNC1H1
0	0.30615509	0.338	0.12	0	Pericyte	MYO1C
0	0.30594737	0.281	0.061	0	Pericyte	STAT6
0	0.30391471	0.269	0.069	0	Pericyte	DAAM2
0	0.30361541	0.329	0.108	0	Pericyte	HDGFL3
0	0.30154634	0.301	0.079	0	Pericyte	NEK7
0	0.30137506	0.298	0.086	0	Pericyte	IRF2
0	0.30104623	0.269	0.073	0	Pericyte	KLF11
0	0.29994616	0.327	0.114	0	Pericyte	NDUFS2
0	0.29959759	0.282	0.083	0	Pericyte	SMTN
0	0.29736437	0.301	0.077	0	Pericyte	PSMB8
0	0.29380965	0.226	0.021	0	Pericyte	CALHM2
0	0.29364502	0.228	0.055	0	Pericyte	BAG2
0	0.29351117	0.271	0.081	0	Pericyte	TNRC6C
0	0.29294738	0.236	0.061	0	Pericyte	PLEKHH3
0	0.29280304	0.287	0.079	0	Pericyte	CREB1
0	0.29242362	0.232	0.051	0	Pericyte	LINC02381
0	0.29037221	0.209	0.056	0	Pericyte	PRKAR2B

0	0.29006826	0.293	0.099	0	Pericyte	SMIM12
0	0.28856351	0.293	0.087	0	Pericyte	ST5
0	0.28488229	0.25	0.07	0	Pericyte	SEPT7P2
0	0.28485078	0.214	0.042	0	Pericyte	CAMK2G
0	0.27747278	0.284	0.072	0	Pericyte	ENG
0	0.27619241	0.229	0.048	0	Pericyte	ZNF667-AS1
0	0.27150916	0.209	0.048	0	Pericyte	RAP2A
0	0.2680189	0.203	0.021	0	Pericyte	CPT1A
0	0.26797819	0.218	0.048	0	Pericyte	SMAP2
0	0.26773773	0.245	0.066	0	Pericyte	CAT
0	0.26770802	0.32	0.111	0	Pericyte	PHLDB1
0	0.26568378	0.255	0.076	0	Pericyte	SMIM30
0	0.26558448	0.204	0.037	0	Pericyte	GNAI1
0	0.26544665	0.23	0.056	0	Pericyte	PCMTD2
0	0.26435845	0.254	0.064	0	Pericyte	ZCCHC24
0	0.26393338	0.279	0.089	0	Pericyte	BCL9L
0	0.26376437	0.201	0.048	0	Pericyte	ARHGAP17
0	0.26158492	0.221	0.055	0	Pericyte	GRK5
0	0.26136342	0.246	0.07	0	Pericyte	SETBP1
0	0.26114648	0.238	0.06	0	Pericyte	ABTB1
0	0.25759527	0.24	0.049	0	Pericyte	A4GALT
0	0.25462818	0.227	0.056	0	Pericyte	FHL3
0	0.2518507	0.209	0.043	0	Pericyte	CBL
5.18E-306	0.67046306	0.865	0.689	1.31E-301	Pericyte	S100A4
7.37E-305	0.49112263	0.831	0.602	1.86E-300	Pericyte	PABPC1
6.65E-304	0.3147272	0.359	0.138	1.68E-299	Pericyte	YAP1
4.16E-302	0.29920339	0.323	0.116	1.05E-297	Pericyte	SGCE
2.40E-300	0.32511084	0.405	0.165	6.05E-296	Pericyte	MAML2
7.05E-300	0.32053518	0.345	0.131	1.78E-295	Pericyte	TRAPPC1
6.88E-299	0.28889291	0.297	0.101	1.74E-294	Pericyte	IRF2BPL
2.32E-298	0.41213677	0.644	0.377	5.84E-294	Pericyte	NDUFB10
2.07E-297	0.43562121	0.771	0.543	5.22E-293	Pericyte	COX5B
3.76E-296	0.33724662	0.363	0.143	9.49E-292	Pericyte	NCK2
4.75E-294	0.40071354	0.578	0.314	1.20E-289	Pericyte	XRCC5
1.74E-291	0.27402016	0.263	0.084	4.39E-287	Pericyte	TAOK3
2.66E-290	0.32537973	0.978	0.912	6.71E-286	Pericyte	RPL29
5.04E-289	0.38987993	0.492	0.236	1.27E-284	Pericyte	RALBP1
9.57E-289	0.4218432	0.779	0.553	2.41E-284	Pericyte	SLC25A3
1.16E-287	0.35441595	0.528	0.266	2.92E-283	Pericyte	ATP6AP2
4.86E-287	0.72201771	0.963	0.85	1.23E-282	Pericyte	DSTN
1.75E-285	0.32269318	0.373	0.152	4.42E-281	Pericyte	NDUFS8
1.80E-285	0.43136594	0.795	0.562	4.53E-281	Pericyte	COX6A1
6.03E-285	0.28038828	0.301	0.106	1.52E-280	Pericyte	MRPS5
4.83E-284	0.7235217	0.341	0.139	1.22E-279	Pericyte	FGF7
7.52E-284	0.38517328	0.618	0.342	1.90E-279	Pericyte	MTATP6P1
1.44E-283	0.31781224	0.41	0.177	3.62E-279	Pericyte	GNG12
6.00E-283	0.39951749	0.483	0.234	1.51E-278	Pericyte	ZC3H13
3.20E-282	0.43245497	0.563	0.295	8.07E-278	Pericyte	IER5L
4.34E-278	0.35556453	0.523	0.268	1.10E-273	Pericyte	MAP4
4.50E-276	0.41737255	0.696	0.441	1.13E-271	Pericyte	ATP5MD
2.01E-275	0.40589556	0.761	0.524	5.07E-271	Pericyte	ATP5ME

2.00E-272	0.33662435	0.474	0.227	5.04E-268	Pericyte	BRD4
3.89E-272	0.47132916	0.485	0.242	9.82E-268	Pericyte	HCFC1R1
4.26E-270	0.44713707	0.77	0.55	1.07E-265	Pericyte	PSMA7
1.02E-269	0.33671387	0.519	0.26	2.57E-265	Pericyte	BOD1L1
2.00E-269	0.3675729	0.466	0.221	5.06E-265	Pericyte	RSF1
1.29E-268	0.36322543	0.539	0.275	3.25E-264	Pericyte	IRF2BP2
4.67E-268	0.40128361	0.643	0.394	1.18E-263	Pericyte	NDUFB7
1.12E-267	0.2846001	0.346	0.137	2.82E-263	Pericyte	TRIM8
1.35E-266	0.2662882	0.315	0.118	3.40E-262	Pericyte	KDM5A
1.46E-266	0.2680839	0.239	0.077	3.67E-262	Pericyte	SLMAP
9.74E-266	0.31330869	0.359	0.148	2.46E-261	Pericyte	SSBP2
3.02E-264	0.4679921	0.604	0.359	7.61E-260	Pericyte	EIF5A
4.98E-264	0.33866701	0.289	0.105	1.26E-259	Pericyte	DKK3
4.48E-262	0.38047223	0.912	0.751	1.13E-257	Pericyte	PPIA
6.32E-260	0.30624665	0.448	0.209	1.59E-255	Pericyte	NCOR1
1.50E-258	0.33785332	0.436	0.203	3.78E-254	Pericyte	PTPRA
6.30E-257	0.38198176	0.558	0.306	1.59E-252	Pericyte	IK
7.59E-256	0.2549988	0.251	0.084	1.92E-251	Pericyte	RUFY3
1.81E-255	0.28547182	0.351	0.144	4.57E-251	Pericyte	BAZ1B
4.19E-253	0.32550958	0.444	0.211	1.06E-248	Pericyte	RBPJ
3.05E-251	0.39523928	0.875	0.713	7.68E-247	Pericyte	YBX1
1.12E-250	0.28761678	0.319	0.126	2.83E-246	Pericyte	SH3KBP1
1.64E-249	0.39896848	0.649	0.406	4.14E-245	Pericyte	UQCRQ
2.02E-249	0.3291168	0.415	0.194	5.11E-245	Pericyte	MRPL32
3.94E-248	0.27773282	0.986	0.939	9.94E-244	Pericyte	RPL18A
6.56E-248	0.33805446	0.512	0.265	1.65E-243	Pericyte	CHD4
1.11E-247	0.40346856	0.546	0.295	2.80E-243	Pericyte	SELENOP
2.27E-247	0.32785175	0.395	0.182	5.72E-243	Pericyte	ARPC1A
2.32E-247	0.28658431	0.375	0.161	5.85E-243	Pericyte	ZBTB7A
2.96E-246	0.30584363	0.447	0.218	7.47E-242	Pericyte	ITGB1BP1
1.57E-245	0.32882065	0.504	0.267	3.96E-241	Pericyte	ATP5F1A
3.82E-244	0.33776599	0.341	0.143	9.63E-240	Pericyte	CHSY1
7.20E-244	0.27919293	0.278	0.102	1.82E-239	Pericyte	SHPRH
1.13E-243	0.32123586	0.441	0.214	2.85E-239	Pericyte	NDUFS7
2.05E-238	0.25207626	0.289	0.11	5.18E-234	Pericyte	ZNF22
5.43E-237	0.37874251	0.821	0.614	1.37E-232	Pericyte	SET
3.94E-236	0.37472314	0.7	0.472	9.94E-232	Pericyte	UQCR11
2.65E-235	0.60504771	0.391	0.182	6.69E-231	Pericyte	THBS1
6.95E-234	0.35419333	0.641	0.394	1.75E-229	Pericyte	NDUFB1
1.32E-233	0.29271761	0.347	0.149	3.33E-229	Pericyte	SMIM10
1.84E-233	0.34538563	0.622	0.375	4.64E-229	Pericyte	UBXN1
1.36E-231	0.35459803	0.602	0.361	3.44E-227	Pericyte	EIF3G
2.25E-229	0.26108078	0.334	0.141	5.68E-225	Pericyte	NARS
3.76E-229	0.37211932	0.625	0.38	9.50E-225	Pericyte	VAMP2
5.00E-229	0.33788723	0.554	0.313	1.26E-224	Pericyte	EIF3A
1.92E-227	0.2527819	0.347	0.148	4.84E-223	Pericyte	CMTM6
5.25E-227	0.29331398	0.324	0.136	1.33E-222	Pericyte	HSPB2
9.18E-227	0.29096815	0.367	0.165	2.32E-222	Pericyte	NUTF2
1.76E-226	0.31516827	0.516	0.286	4.44E-222	Pericyte	ATP5F1C
5.77E-226	0.28531011	0.331	0.14	1.45E-221	Pericyte	VASP
1.07E-224	0.31429116	0.386	0.182	2.69E-220	Pericyte	CCT5

2.06E-222	0.27585846	0.344	0.148	5.19E-218	Pericyte	RSBN1L
2.54E-222	0.2600746	0.311	0.128	6.42E-218	Pericyte	PBRM1
3.19E-222	0.28132366	0.367	0.166	8.05E-218	Pericyte	DTWD1
2.10E-221	0.27226886	0.326	0.14	5.31E-217	Pericyte	RTL8A
9.79E-221	0.25331269	0.337	0.145	2.47E-216	Pericyte	PIN1
8.91E-218	0.32010612	0.413	0.202	2.25E-213	Pericyte	DDAH2
1.55E-216	0.26924248	0.971	0.89	3.90E-212	Pericyte	RPL35
1.29E-215	0.26576619	0.341	0.149	3.25E-211	Pericyte	RBFOX2
3.88E-215	0.32098802	0.365	0.169	9.78E-211	Pericyte	OSBPL1A
3.96E-215	0.28676623	0.337	0.142	9.98E-211	Pericyte	MAFB
5.37E-214	0.26189232	0.975	0.885	1.35E-209	Pericyte	RPL38
6.58E-211	0.41787425	0.595	0.363	1.66E-206	Pericyte	PRKAR1A
1.41E-209	0.29411985	0.424	0.211	3.55E-205	Pericyte	KPNB1
1.53E-209	0.31233854	0.927	0.778	3.87E-205	Pericyte	BTF3
6.01E-209	0.31294849	0.688	0.454	1.52E-204	Pericyte	CSDE1
9.91E-209	0.35646232	0.657	0.434	2.50E-204	Pericyte	ATP5MC3
2.12E-207	0.40641979	0.78	0.573	5.34E-203	Pericyte	TUBA1B
2.33E-206	0.26311877	0.347	0.158	5.87E-202	Pericyte	CBR1
4.64E-204	0.26819163	0.523	0.287	1.17E-199	Pericyte	ASH1L
5.38E-201	0.30044748	0.417	0.21	1.36E-196	Pericyte	ITPRIPL2
2.15E-197	0.33737339	0.468	0.252	5.42E-193	Pericyte	SPATS2L
3.25E-197	0.33537537	0.788	0.593	8.19E-193	Pericyte	ELOB
3.88E-196	0.26656697	0.409	0.207	9.79E-192	Pericyte	MRPL43
7.48E-196	0.26820377	0.415	0.209	1.89E-191	Pericyte	AHCYL1
7.30E-195	0.29388107	0.389	0.191	1.84E-190	Pericyte	TMEM47
4.88E-194	0.30921613	0.519	0.295	1.23E-189	Pericyte	PSIP1
6.12E-194	0.27780813	0.415	0.209	1.54E-189	Pericyte	MTHFD2
3.83E-193	0.29193487	0.402	0.202	9.66E-189	Pericyte	PPM1G
6.96E-193	0.31588117	0.866	0.692	1.76E-188	Pericyte	SUMO2
1.70E-191	0.32362463	0.632	0.416	4.30E-187	Pericyte	ATP5PD
1.94E-191	0.29947585	0.398	0.199	4.89E-187	Pericyte	MAP1LC3A
3.64E-188	0.27458546	0.438	0.233	9.18E-184	Pericyte	PHB2
4.56E-186	0.31459183	0.599	0.369	1.15E-181	Pericyte	KMT2E
7.20E-185	0.27325635	0.244	0.096	1.82E-180	Pericyte	SMOC2
4.42E-184	0.25143901	0.389	0.195	1.11E-179	Pericyte	RTL8C
1.14E-183	0.28516115	0.608	0.375	2.88E-179	Pericyte	NR3C1
1.54E-183	0.25909847	0.423	0.223	3.88E-179	Pericyte	MRPL34
5.61E-183	0.31849731	0.726	0.514	1.42E-178	Pericyte	RPS17
4.09E-178	0.32763784	0.702	0.472	1.03E-173	Pericyte	TUBA1A
1.77E-175	0.27312373	0.519	0.304	4.47E-171	Pericyte	ISCU
2.40E-175	0.29751807	0.624	0.406	6.06E-171	Pericyte	EIF3F
1.26E-174	0.54765034	0.671	0.465	3.18E-170	Pericyte	SOCS3
7.22E-174	0.30042637	0.568	0.348	1.82E-169	Pericyte	PURA
7.72E-173	0.25000188	0.99	0.923	1.95E-168	Pericyte	FAU
8.92E-172	0.26043575	0.26	0.109	2.25E-167	Pericyte	HIPK2
1.50E-171	0.25225146	0.422	0.227	3.80E-167	Pericyte	SDHD
2.35E-169	0.2737397	0.321	0.149	5.93E-165	Pericyte	UGCG
5.40E-169	0.30097762	0.542	0.32	1.36E-164	Pericyte	KLF9
1.18E-166	0.30068681	0.68	0.478	2.99E-162	Pericyte	NDUFB4
8.15E-166	0.32439346	0.657	0.442	2.06E-161	Pericyte	SYF2
1.96E-165	0.29271927	0.543	0.331	4.95E-161	Pericyte	YY1

4.83E-165	0.25960998	0.406	0.213	1.22E-160	Pericyte	OSBPL8
5.03E-165	0.29640977	0.654	0.449	1.27E-160	Pericyte	LEPROT
3.13E-163	0.26997059	0.525	0.32	7.89E-159	Pericyte	HADHA
6.10E-163	0.27534539	0.467	0.27	1.54E-158	Pericyte	CHMP3
2.55E-162	0.26211626	0.486	0.283	6.43E-158	Pericyte	CHMP5
3.08E-161	0.28816074	0.663	0.465	7.78E-157	Pericyte	NEDD8
7.86E-160	0.29744927	0.864	0.698	1.98E-155	Pericyte	NAP1L1
4.45E-159	0.56494723	0.758	0.594	1.12E-154	Pericyte	ZFP36
2.02E-158	0.30127583	0.468	0.274	5.11E-154	Pericyte	TMEM256
3.15E-156	0.26928541	0.476	0.276	7.94E-152	Pericyte	SARS
7.24E-156	0.28042346	0.673	0.48	1.83E-151	Pericyte	BRK1
1.72E-155	0.25999766	0.242	0.102	4.34E-151	Pericyte	SEPHS2
7.75E-154	0.27573744	0.385	0.208	1.96E-149	Pericyte	PCYOX1
1.01E-153	0.43268641	0.871	0.762	2.54E-149	Pericyte	YBX3
7.38E-153	0.26221052	0.549	0.348	1.86E-148	Pericyte	HNRNPR
2.66E-148	0.26357904	0.495	0.298	6.71E-144	Pericyte	COX14
9.58E-148	0.26495813	0.419	0.236	2.42E-143	Pericyte	REEP3
2.66E-147	0.25522357	0.51	0.317	6.72E-143	Pericyte	C19orf70
3.38E-147	0.27510978	0.509	0.316	8.54E-143	Pericyte	PSMB3
1.35E-146	0.26702745	0.556	0.355	3.42E-142	Pericyte	NDUFB9
3.90E-145	0.28428351	0.399	0.224	9.84E-141	Pericyte	MXI1
4.37E-144	0.25249338	0.559	0.363	1.10E-139	Pericyte	VPS28
3.05E-141	0.28314484	0.741	0.555	7.69E-137	Pericyte	DEK
4.32E-141	0.28082904	0.229	0.098	1.09E-136	Pericyte	RNF152
5.55E-141	0.25055077	0.481	0.293	1.40E-136	Pericyte	PDAP1
4.65E-140	0.30918397	0.363	0.192	1.17E-135	Pericyte	RRAD
1.12E-138	0.26451812	0.701	0.502	2.84E-134	Pericyte	ANP32B
5.28E-137	0.26737838	0.7	0.517	1.33E-132	Pericyte	ATP5F1D
3.93E-133	0.25700433	0.643	0.447	9.91E-129	Pericyte	RWDD1
8.06E-133	0.30272958	0.366	0.204	2.03E-128	Pericyte	CCDC107
1.15E-131	0.32584039	0.427	0.249	2.90E-127	Pericyte	DNAJB4
6.16E-128	0.39655263	0.689	0.531	1.55E-123	Pericyte	MIF
2.27E-124	0.25682251	0.509	0.333	5.73E-120	Pericyte	RAD23A
8.21E-122	0.26664179	0.781	0.618	2.07E-117	Pericyte	COX7A2
6.32E-114	0.27082023	0.397	0.235	1.59E-109	Pericyte	FOSL2
3.48E-113	0.28621411	0.839	0.859	8.78E-109	Pericyte	TIMP1
4.28E-110	0.25513649	0.641	0.468	1.08E-105	Pericyte	NDUFB2
8.20E-109	0.34581006	0.386	0.234	2.07E-104	Pericyte	MYC
2.60E-106	0.44456691	0.255	0.131	6.57E-102	Pericyte	CREM
1.57E-103	0.25762457	0.266	0.137	3.96E-99	Pericyte	GLRX
1.72E-82	0.50870747	0.461	0.327	4.35E-78	Pericyte	RGS16
1.51E-77	0.45462411	0.511	0.385	3.80E-73	Pericyte	NR4A1
2.08E-72	0.2543639	0.669	0.522	5.25E-68	Pericyte	PMEPA1
7.94E-69	0.25399425	0.538	0.393	2.00E-64	Pericyte	BNIP3L
1.05E-57	0.26607693	0.448	0.334	2.65E-53	Pericyte	NFIL3
1.53E-54	0.25833376	0.518	0.392	3.86E-50	Pericyte	HES1
1.74E-40	0.37333475	0.428	0.331	4.40E-36	Pericyte	VEGFA
1.14E-09	0.25079064	0.617	0.58	2.88E-05	Pericyte	ATF3
0	2.40239831	0.962	0.353	0	Chond1	CYTL1
0	1.79638976	0.68	0.139	0	Chond1	IBSP
0	1.69060801	0.956	0.695	0	Chond1	CRYAB

0	1.68673682	0.982	0.575	0	Chond1	APOD
0	1.663744	0.646	0.029	0	Chond1	IL17B
0	1.65056591	0.984	0.627	0	Chond1	RBP4
0	1.59580123	0.999	0.896	0	Chond1	C2orf40
0	1.38559841	0.94	0.524	0	Chond1	CHAD
0	1.35695627	0.351	0.067	0	Chond1	SPP1
0	1.31966172	0.533	0.094	0	Chond1	HSPA6
0	1.31952243	0.937	0.634	0	Chond1	HSPA1A
0	1.30109065	0.324	0.022	0	Chond1	COL10A1
0	1.29490107	0.973	0.561	0	Chond1	SOD3
0	1.20475946	0.816	0.29	0	Chond1	PDPN
0	1.18637009	0.992	0.789	0	Chond1	FGFBP2
0	1.18596068	0.521	0.159	0	Chond1	HMOX1
0	1.14064408	0.893	0.528	0	Chond1	HSPA1B
0	1.04492328	0.618	0.087	0	Chond1	STMN1
0	0.96467487	0.908	0.584	0	Chond1	H2AFZ
0	0.9279976	0.991	0.856	0	Chond1	CST3
0	0.89819358	0.449	0.176	0	Chond1	DDIT4
0	0.86903275	0.697	0.262	0	Chond1	PHLDA2
0	0.86285109	0.903	0.552	0	Chond1	MAP1LC3B
0	0.80416379	1	0.985	0	Chond1	MGP
0	0.79516733	0.66	0.269	0	Chond1	ROCR
0	0.79456703	0.735	0.369	0	Chond1	DDIT3
0	0.78502966	0.752	0.489	0	Chond1	KCNMA1
0	0.78455614	0.708	0.353	0	Chond1	SLC3A2
0	0.78145609	0.984	0.736	0	Chond1	SERPINA1
0	0.77787743	0.819	0.489	0	Chond1	HSPE1
0	0.77496605	0.911	0.635	0	Chond1	DYNLL1
0	0.76969508	0.844	0.54	0	Chond1	DNAJB1
0	0.76092415	0.969	0.674	0	Chond1	MIA
0	0.74791162	0.723	0.303	0	Chond1	AL118516.1
0	0.74568411	0.628	0.257	0	Chond1	LARP6
0	0.73715355	0.627	0.279	0	Chond1	FGF2
0	0.72864959	0.474	0.1	0	Chond1	BMP2
0	0.72554367	0.748	0.335	0	Chond1	SPINT2
0	0.72431604	0.489	0.141	0	Chond1	NMB
0	0.71952253	0.547	0.149	0	Chond1	MARCKSL1
0	0.71675495	0.98	0.821	0	Chond1	S100A10
0	0.70652324	0.814	0.444	0	Chond1	CDKN1A
0	0.70240914	0.855	0.522	0	Chond1	DNAJA1
0	0.70040113	0.986	0.88	0	Chond1	HSP90AB1
0	0.68486664	0.657	0.292	0	Chond1	TM4SF1
0	0.67144996	0.677	0.294	0	Chond1	BTG3
0	0.66783353	0.999	0.942	0	Chond1	S100A6
0	0.66755253	0.854	0.534	0	Chond1	SELENOK
0	0.65718879	0.652	0.278	0	Chond1	UBE2S
0	0.6317965	0.976	0.856	0	Chond1	HSP90AA1
0	0.63118712	0.226	0.013	0	Chond1	AZGP1
0	0.62837619	0.567	0.211	0	Chond1	BEX2
0	0.61088295	0.958	0.722	0	Chond1	CALM1
0	0.61087258	0.608	0.283	0	Chond1	TUBB2B

0	0.61003915	0.879	0.591	0	Chond1	EMP3
0	0.60248176	0.804	0.496	0	Chond1	EIF1B
0	0.59765093	0.76	0.405	0	Chond1	CYCS
0	0.5929743	0.759	0.405	0	Chond1	TUBB2A
0	0.59214364	0.721	0.341	0	Chond1	GEM
0	0.57967848	0.711	0.345	0	Chond1	ODC1
0	0.57422706	0.91	0.623	0	Chond1	SAT1
0	0.57304522	0.677	0.428	0	Chond1	PMP22
0	0.56985685	0.434	0.163	0	Chond1	SERPINI1
0	0.56855834	0.955	0.705	0	Chond1	S100A13
0	0.561863	0.502	0.193	0	Chond1	SNHG12
0	0.56121296	0.808	0.507	0	Chond1	BZW1
0	0.54998614	0.892	0.671	0	Chond1	HSPB1
0	0.54242332	0.933	0.716	0	Chond1	LGALS3
0	0.54089932	0.719	0.394	0	Chond1	WTAP
0	0.54055432	0.391	0.094	0	Chond1	HMGA1
0	0.53953102	0.727	0.417	0	Chond1	TNFRSF12A
0	0.53575989	0.835	0.587	0	Chond1	SQSTM1
0	0.53568545	0.798	0.502	0	Chond1	H2AFJ
0	0.53432632	0.98	0.905	0	Chond1	UBC
0	0.53331353	0.865	0.557	0	Chond1	PPP1R15A
0	0.532915	0.522	0.199	0	Chond1	CRNDE
0	0.53065767	0.656	0.312	0	Chond1	CD55
0	0.52443333	0.786	0.506	0	Chond1	RAN
0	0.52212106	0.722	0.267	0	Chond1	HSPH1
0	0.5203715	0.932	0.72	0	Chond1	UBB
0	0.52029777	0.952	0.732	0	Chond1	CALM2
0	0.51970124	0.306	0.07	0	Chond1	WFDC2
0	0.5190586	0.919	0.69	0	Chond1	S100A11
0	0.51348027	0.388	0.119	0	Chond1	ZFAND2A
0	0.51333882	0.735	0.427	0	Chond1	GNG5
0	0.51119525	0.214	0.007	0	Chond1	ELF3
0	0.51092576	0.648	0.483	0	Chond1	NFKBIA
0	0.50696819	0.376	0.116	0	Chond1	ISG20
0	0.50651037	0.967	0.749	0	Chond1	RGCC
0	0.50383778	0.412	0.118	0	Chond1	N4BP2L1
0	0.50277368	0.376	0.101	0	Chond1	KRT18
0	0.49741732	0.675	0.379	0	Chond1	DANCR
0	0.49583762	0.592	0.268	0	Chond1	BAG3
0	0.49274756	0.894	0.6	0	Chond1	SNHG8
0	0.49146329	0.458	0.177	0	Chond1	PCSK1N
0	0.4908492	0.731	0.447	0	Chond1	IER5
0	0.49011864	0.454	0.189	0	Chond1	MLF1
0	0.48912712	0.354	0.084	0	Chond1	CKS2
0	0.48796253	0.36	0.113	0	Chond1	GGTA1P
0	0.48747116	0.453	0.173	0	Chond1	CDK2AP2
0	0.48737251	0.723	0.436	0	Chond1	RBM8A
0	0.48696304	0.697	0.347	0	Chond1	THUMPD3-AS1
0	0.48415997	0.482	0.16	0	Chond1	FTH1P10
0	0.48380048	0.863	0.716	0	Chond1	C11orf96
0	0.4828701	0.627	0.335	0	Chond1	HMGB2

0	0.4820229	0.439	0.176	0	Chond1	SNHG19
0	0.47151161	0.665	0.372	0	Chond1	RPL22L1
0	0.47012507	0.989	0.837	0	Chond1	SNORC
0	0.46998808	0.834	0.559	0	Chond1	SBDS
0	0.46919969	0.462	0.268	0	Chond1	FABP5
0	0.46300656	0.937	0.805	0	Chond1	GADD45B
0	0.46176086	0.619	0.333	0	Chond1	PNRC2
0	0.45866969	0.653	0.367	0	Chond1	DNAJB6
0	0.45452561	0.369	0.119	0	Chond1	UBE2D1
0	0.45267704	0.495	0.219	0	Chond1	GTF2B
0	0.45211108	0.998	0.953	0	Chond1	EIF1
0	0.44985235	0.26	0.03	0	Chond1	LCN2
0	0.44860501	0.869	0.64	0	Chond1	TSC22D1
0	0.44162389	0.57	0.297	0	Chond1	TOP1
0	0.44153548	0.544	0.271	0	Chond1	DCXR
0	0.44059183	0.517	0.254	0	Chond1	DNAJB9
0	0.43748118	0.485	0.24	0	Chond1	CITED4
0	0.43147282	0.246	0.02	0	Chond1	MATN3
0	0.43112452	0.213	0.017	0	Chond1	TACC3
0	0.43052054	0.396	0.142	0	Chond1	ATF7IP2
0	0.42911829	0.644	0.374	0	Chond1	ARL6IP1
0	0.42823341	0.847	0.58	0	Chond1	SEC61G
0	0.42800444	0.857	0.612	0	Chond1	SEC61B
0	0.42726009	0.373	0.132	0	Chond1	BEX5
0	0.42430048	0.962	0.805	0	Chond1	MT-RNR1
0	0.4236146	0.988	0.817	0	Chond1	RPS26
0	0.42225803	0.811	0.598	0	Chond1	NUPR1
0	0.42153204	0.663	0.389	0	Chond1	POLR2K
0	0.42143455	0.612	0.34	0	Chond1	CAMTA1
0	0.42004719	0.469	0.204	0	Chond1	CDKN1C
0	0.41667504	0.331	0.077	0	Chond1	NUPR2
0	0.41503811	0.597	0.469	0	Chond1	FRZB
0	0.41458674	0.812	0.548	0	Chond1	GPX4
0	0.41405882	0.218	0.025	0	Chond1	CHAC1
0	0.4133737	0.859	0.586	0	Chond1	NDUFA4
0	0.4130176	0.226	0.063	0	Chond1	SFN
0	0.41170076	0.665	0.407	0	Chond1	TCEAL9
0	0.40904655	0.914	0.678	0	Chond1	ZFAS1
0	0.4060828	0.499	0.235	0	Chond1	DMKN
0	0.40412055	0.98	0.751	0	Chond1	S100A1
0	0.40351668	0.34	0.122	0	Chond1	TSPAN13
0	0.39956931	0.894	0.652	0	Chond1	TSPO
0	0.39930825	0.611	0.299	0	Chond1	SNHG9
0	0.39703609	0.662	0.408	0	Chond1	TUBA1C
0	0.39670355	0.517	0.267	0	Chond1	ILF2
0	0.39583511	0.363	0.128	0	Chond1	AKIRIN2
0	0.39532595	0.797	0.577	0	Chond1	TXN
0	0.39405528	0.872	0.643	0	Chond1	ANXA5
0	0.3937557	0.259	0.051	0	Chond1	AC015912.3
0	0.39292986	0.649	0.358	0	Chond1	LRRFIP1
0	0.39203821	0.597	0.312	0	Chond1	CNN3

0	0.39011175	0.926	0.714	0	Chond1	GSTP1
0	0.38968378	0.291	0.099	0	Chond1	TIFA
0	0.38858156	0.743	0.471	0	Chond1	SEM1
0	0.38560272	0.549	0.299	0	Chond1	EZR
0	0.38489141	0.848	0.621	0	Chond1	EIF5
0	0.38302721	0.625	0.367	0	Chond1	CCDC59
0	0.38263167	0.535	0.298	0	Chond1	BUD31
0	0.38251995	0.332	0.112	0	Chond1	HOTAIRM1
0	0.38137076	0.621	0.362	0	Chond1	SNRPF
0	0.38098997	0.543	0.309	0	Chond1	CYSTM1
0	0.37987778	0.818	0.563	0	Chond1	LMNA
0	0.37868873	0.367	0.154	0	Chond1	TCEAL6
0	0.37370482	0.451	0.218	0	Chond1	UGDH
0	0.37236229	0.35	0.122	0	Chond1	NKX3-1
0	0.37004503	0.665	0.423	0	Chond1	RHEB
0	0.36851826	0.439	0.205	0	Chond1	MIR100HG
0	0.3683736	0.479	0.203	0	Chond1	PNP
0	0.36495003	0.344	0.136	0	Chond1	DDIT4L
0	0.36482245	0.783	0.55	0	Chond1	LINC01578
0	0.36453256	0.295	0.074	0	Chond1	CLCF1
0	0.36424672	0.947	0.769	0	Chond1	PNRC1
0	0.36412217	0.524	0.289	0	Chond1	YWHAH
0	0.36327409	0.695	0.446	0	Chond1	RSRC2
0	0.36068513	0.375	0.165	0	Chond1	RHOD
0	0.36013161	0.823	0.591	0	Chond1	SAP18
0	0.35867796	0.446	0.193	0	Chond1	MIR22HG
0	0.35733449	0.65	0.39	0	Chond1	FGFR1
0	0.35689764	0.449	0.228	0	Chond1	BCAS2
0	0.35501543	0.505	0.264	0	Chond1	SVIP
0	0.35429722	0.889	0.644	0	Chond1	SH3BGRL3
0	0.35409812	0.479	0.25	0	Chond1	PITHD1
0	0.35402573	0.473	0.203	0	Chond1	ETNK1
0	0.35219275	0.919	0.696	0	Chond1	CCNI
0	0.3510443	0.274	0.063	0	Chond1	PTTG1
0	0.34707305	0.349	0.162	0	Chond1	ANKRD37
0	0.34524913	0.213	0.095	0	Chond1	G0S2
0	0.34475942	0.992	0.947	0	Chond1	MT-RNR2
0	0.34427821	0.648	0.395	0	Chond1	COX7B
0	0.34323309	0.417	0.233	0	Chond1	IRF1
0	0.34259941	0.841	0.594	0	Chond1	CLEC3A
0	0.34252659	0.437	0.22	0	Chond1	EFNA1
0	0.3348005	0.782	0.544	0	Chond1	SUB1
0	0.33374822	0.256	0.071	0	Chond1	ARG2
0	0.33324511	0.498	0.28	0	Chond1	NLRP1
0	0.33317077	0.399	0.181	0	Chond1	SERTAD2
0	0.33053284	0.272	0.081	0	Chond1	RAMP1
0	0.32950737	0.469	0.258	0	Chond1	CHMP4B
0	0.32942317	0.909	0.702	0	Chond1	CHCHD2
0	0.32830566	0.609	0.338	0	Chond1	MAFF
0	0.32692248	0.226	0.083	0	Chond1	PDE4B
0	0.32651805	0.592	0.358	0	Chond1	SNRPG

0	0.32600572	0.382	0.146	0	Chond1	FAM107B
0	0.32590477	0.55	0.331	0	Chond1	SDCBP
0	0.32528137	0.545	0.324	0	Chond1	LITAF
0	0.32256263	0.379	0.164	0	Chond1	UPP1
0	0.3223343	0.593	0.366	0	Chond1	CDV3
0	0.32073976	0.323	0.13	0	Chond1	PLIN2
0	0.32065537	0.902	0.692	0	Chond1	SOD1
0	0.31900452	0.714	0.502	0	Chond1	HSPD1
0	0.31782064	0.61	0.318	0	Chond1	F13A1
0	0.31759261	0.665	0.413	0	Chond1	IER3
0	0.31721825	0.526	0.299	0	Chond1	EIF3J
0	0.31715329	0.896	0.693	0	Chond1	PRDX1
0	0.31686861	0.557	0.341	0	Chond1	HNRNPAB
0	0.31598881	0.556	0.338	0	Chond1	MPC2
0	0.3151709	0.536	0.31	0	Chond1	LMO4
0	0.31514265	0.277	0.09	0	Chond1	C1QTNF4
0	0.31294438	0.7	0.478	0	Chond1	SERP1
0	0.31288726	0.709	0.489	0	Chond1	DBI
0	0.31227967	0.347	0.16	0	Chond1	HIGD1A
0	0.31159416	0.372	0.193	0	Chond1	KIF9
0	0.30934025	0.909	0.715	0	Chond1	BTG1
0	0.30800073	0.358	0.162	0	Chond1	SMIM4
0	0.3077824	0.703	0.465	0	Chond1	ARF4
0	0.30736408	0.287	0.098	0	Chond1	CDKN2D
0	0.30490025	0.414	0.221	0	Chond1	DPH3
0	0.30447192	0.349	0.148	0	Chond1	SLC25A33
0	0.30434381	0.332	0.139	0	Chond1	CDC37L1
0	0.30338515	0.309	0.125	0	Chond1	TPD52L1
0	0.30257487	0.995	0.944	0	Chond1	FTH1
0	0.30160959	0.339	0.151	0	Chond1	PLAUR
0	0.30157764	0.4	0.209	0	Chond1	SOX9-AS1
0	0.30046258	0.402	0.209	0	Chond1	AHSA1
0	0.29886764	0.747	0.541	0	Chond1	PTGES3
0	0.2986036	0.555	0.348	0	Chond1	SNRPB
0	0.29848562	0.332	0.132	0	Chond1	CMC2
0	0.2979202	0.524	0.314	0	Chond1	CCT4
0	0.29666188	0.522	0.339	0	Chond1	GSTO1
0	0.29558184	0.424	0.233	0	Chond1	KIAA0040
0	0.29536497	0.76	0.493	0	Chond1	ITM2C
0	0.29459069	0.355	0.17	0	Chond1	ARPC5L
0	0.29426382	0.41	0.215	0	Chond1	ENHO
0	0.29254173	0.917	0.725	0	Chond1	SKP1
0	0.29173553	0.727	0.509	0	Chond1	RPL9P9
0	0.2916091	0.204	0.073	0	Chond1	CD83
0	0.29039115	0.698	0.475	0	Chond1	MORF4L2
0	0.29036972	0.282	0.093	0	Chond1	SPHK1
0	0.28926885	0.211	0.04	0	Chond1	ALDH1A2
0	0.28912898	0.672	0.426	0	Chond1	TCEAL2
0	0.2886048	0.525	0.328	0	Chond1	UBE2D2
0	0.28749774	0.225	0.08	0	Chond1	ZNF487
0	0.28431696	0.366	0.152	0	Chond1	KRT8

0	0.28428567	0.266	0.098	0	Chond1	AL392172.1
0	0.28331293	0.996	0.938	0	Chond1	RPL24
0	0.28303958	0.71	0.49	0	Chond1	SF3B6
0	0.28166557	0.774	0.557	0	Chond1	YWHAZ
0	0.28040155	0.388	0.211	0	Chond1	SNHG3
0	0.28007649	0.45	0.26	0	Chond1	NDUFA6
0	0.27722324	0.445	0.251	0	Chond1	BIRC2
0	0.2770306	0.906	0.714	0	Chond1	OAZ1
0	0.27575976	0.988	0.87	0	Chond1	SERF2
0	0.27560415	0.896	0.696	0	Chond1	RPL36AL
0	0.2755257	0.869	0.627	0	Chond1	TUBB4B
0	0.27490779	0.215	0.064	0	Chond1	SNHG10
0	0.27483073	0.888	0.668	0	Chond1	OST4
0	0.27330308	0.76	0.545	0	Chond1	HMGN2
0	0.27205914	0.342	0.129	0	Chond1	MXD1
0	0.26863683	0.368	0.205	0	Chond1	AC044849.1
0	0.26672303	0.81	0.611	0	Chond1	HNRNPC
0	0.26628952	0.39	0.166	0	Chond1	SNHG25
0	0.2657458	0.386	0.204	0	Chond1	ASXL1
0	0.26511715	0.826	0.594	0	Chond1	YWHAE
0	0.26469784	0.425	0.218	0	Chond1	NEU1
0	0.26453876	0.343	0.173	0	Chond1	TPBG
0	0.26450754	0.351	0.185	0	Chond1	MED19
0	0.26315993	0.606	0.344	0	Chond1	CILP
0	0.260816	0.574	0.378	0	Chond1	ELOC
0	0.26057965	0.362	0.189	0	Chond1	UBE2A
0	0.26031691	0.289	0.121	0	Chond1	TSPYL4
0	0.26025358	0.385	0.218	0	Chond1	MPLKIP
0	0.26014152	0.653	0.446	0	Chond1	XBP1
0	0.25989358	0.414	0.253	0	Chond1	IFT20
0	0.25935056	0.583	0.374	0	Chond1	BEX4
0	0.25926445	0.289	0.117	0	Chond1	ERN1
0	0.25895748	0.333	0.174	0	Chond1	MORN2
0	0.25774588	0.378	0.204	0	Chond1	ISCA1
0	0.25757492	0.902	0.679	0	Chond1	C12orf57
0	0.25696783	0.309	0.154	0	Chond1	LGALSL
0	0.25542598	0.551	0.341	0	Chond1	SDC4
0	0.25414102	0.818	0.595	0	Chond1	TMEM258
0	0.25353239	0.231	0.08	0	Chond1	TGFBR3L
0	0.25238304	0.707	0.519	0	Chond1	HERPUD1
0	0.25161706	0.284	0.139	0	Chond1	FNDC4
0	0.25138697	0.618	0.413	0	Chond1	TAF7
0	0.25130331	0.593	0.371	0	Chond1	GADD45A
0	0.25123577	0.485	0.29	0	Chond1	UAP1
0	0.25011362	0.877	0.659	0	Chond1	TMA7
0	0.25004125	0.291	0.148	0	Chond1	TMEM106C
0	1.04125013	0.909	0.943	0	Chond2	NEAT1
0	0.85530923	0.998	0.999	0	Chond2	MALAT1
0	0.81742933	0.535	0.626	0	Chond2	DST
0	0.68213497	0.538	0.604	0	Chond2	COL11A1
0	0.67070679	0.734	0.699	0	Chond2	COL2A1

0	0.62596003	0.801	0.812	0	Chond2	FN1
0	0.62355376	0.945	0.906	0	Chond2	ACAN
0	0.46663716	0.685	0.801	0	Chond2	FMOD
0	0.45558591	0.878	0.915	0	Chond2	COMP
0	0.39615585	0.205	0.403	0	Chond2	SLC5A3
0	0.37459242	0.734	0.786	0	Chond2	EGR1
0	0.37008652	0.186	0.368	0	Chond2	ABI2
0	0.31570351	0.918	0.932	0	Chond2	LUM
0	0.31479769	0.224	0.452	0	Chond2	NUFIP2
0	0.31452107	0.706	0.609	0	Chond2	OGN
0	0.30936112	0.221	0.42	0	Chond2	ANKRD28
0	0.30670043	0.198	0.409	0	Chond2	CHD2
0	0.30209961	0.246	0.473	0	Chond2	FNDC3B
0	0.28712064	0.119	0.267	0	Chond2	CHORDC1
0	0.28655434	0.221	0.428	0	Chond2	CDR1
0	0.2838621	0.262	0.513	0	Chond2	SF1
0	0.28256616	0.147	0.303	0	Chond2	EWSR1
0	0.27817275	0.194	0.377	0	Chond2	BCAT1
0	0.27604672	0.167	0.336	0	Chond2	ELL2
0	0.27495247	0.294	0.569	0	Chond2	DDX3Y
0	0.27474093	0.231	0.476	0	Chond2	IFRD1
0	0.27089763	0.151	0.312	0	Chond2	CREB5
0	0.26556996	0.282	0.551	0	Chond2	SLC38A2
0	0.26479553	0.143	0.313	0	Chond2	ANKRD36BP1
0	0.26417482	0.153	0.329	0	Chond2	GFPT1
0	0.26322006	0.243	0.484	0	Chond2	SEC31A
0	0.25925693	0.285	0.569	0	Chond2	DDX3X
0	0.25797079	0.252	0.545	0	Chond2	SPAG9
0	0.25769454	0.136	0.289	0	Chond2	CHD1
0	0.25701272	0.288	0.575	0	Chond2	SRSF11
0	0.25371419	0.156	0.317	0	Chond2	COLGALT2
0	0.25338168	0.133	0.287	0	Chond2	PELI1
2.80E-306	0.31360513	0.28	0.53	7.07E-302	Chond2	SKIL
5.90E-306	0.33764088	0.218	0.421	1.49E-301	Chond2	SLC25A36
1.31E-304	0.31923276	0.275	0.517	3.31E-300	Chond2	UGP2
1.22E-303	0.33098458	0.19	0.371	3.08E-299	Chond2	AFF4
5.03E-301	0.27429936	0.137	0.278	1.27E-296	Chond2	SPEN
1.02E-300	0.25677337	0.177	0.345	2.57E-296	Chond2	CHST3
8.92E-292	0.33196657	0.291	0.549	2.25E-287	Chond2	JMJD1C
4.31E-289	0.2519561	0.131	0.266	1.09E-284	Chond2	TUT4
2.14E-282	0.30867706	0.154	0.302	5.39E-278	Chond2	BDP1
4.12E-276	0.34880376	0.155	0.304	1.04E-271	Chond2	RORA
3.78E-273	0.28611041	0.12	0.242	9.54E-269	Chond2	NFATC2
2.71E-271	0.28689622	0.218	0.406	6.85E-267	Chond2	SLC39A14
3.46E-269	0.32468369	0.177	0.337	8.73E-265	Chond2	LDLR
8.08E-266	0.25905928	0.117	0.238	2.04E-261	Chond2	MSI2
2.30E-265	0.27567809	0.32	0.585	5.80E-261	Chond2	MT-ND4L
8.44E-264	0.27116313	0.123	0.247	2.13E-259	Chond2	NFATC1
4.57E-263	0.2568383	0.373	0.692	1.15E-258	Chond2	PNISR
8.75E-261	0.26117891	0.278	0.499	2.21E-256	Chond2	SLC14A1
1.21E-259	0.25888494	0.146	0.282	3.04E-255	Chond2	AUTS2

5.90E-258	0.30403568	0.149	0.286	1.49E-253	Chond2	GPRC5A
3.82E-255	0.34180088	0.272	0.501	9.65E-251	Chond2	CHD9
1.85E-250	0.28659021	0.345	0.633	4.66E-246	Chond2	MBNL1
1.99E-250	0.2778874	0.107	0.217	5.01E-246	Chond2	GPSM2
4.95E-245	0.30392807	0.272	0.495	1.25E-240	Chond2	IVNS1ABP
2.38E-244	0.27535435	0.134	0.26	6.01E-240	Chond2	LINC00632
5.15E-242	0.35911932	0.212	0.389	1.30E-237	Chond2	PRPF4B
1.44E-240	0.38488836	0.245	0.45	3.65E-236	Chond2	GOLGA4
9.16E-239	0.25875848	0.124	0.241	2.31E-234	Chond2	ADPRHL1
5.29E-236	0.34929889	0.299	0.548	1.34E-231	Chond2	RBM25
3.11E-235	0.26995418	0.117	0.23	7.85E-231	Chond2	PRKCA
4.38E-235	0.25996785	0.316	0.567	1.11E-230	Chond2	TTC3
9.01E-235	0.36071202	0.159	0.296	2.27E-230	Chond2	HMGCS1
3.31E-234	0.39047313	0.265	0.49	8.35E-230	Chond2	SRSF10
1.30E-231	0.26892236	0.147	0.276	3.27E-227	Chond2	MDM4
1.61E-231	0.35730834	0.18	0.332	4.05E-227	Chond2	WEE1
2.16E-231	0.26401552	0.227	0.408	5.45E-227	Chond2	MRC2
4.78E-230	0.34265314	0.252	0.458	1.21E-225	Chond2	MELTF
1.24E-225	0.3443357	0.15	0.279	3.13E-221	Chond2	INTS6
2.91E-225	0.30646692	0.212	0.383	7.33E-221	Chond2	DDR2
2.11E-222	0.28629633	0.118	0.229	5.33E-218	Chond2	SEC24D
2.22E-217	0.29278133	0.149	0.275	5.59E-213	Chond2	C1orf56
1.52E-215	0.32224941	0.127	0.241	3.84E-211	Chond2	GLIS3
1.59E-214	0.3346808	0.135	0.254	4.00E-210	Chond2	GABPB1-AS1
1.19E-206	0.37914735	0.287	0.511	3.00E-202	Chond2	SLC25A37
7.53E-205	0.37726335	0.215	0.381	1.90E-200	Chond2	TLE4
9.52E-205	0.33270486	0.345	0.619	2.40E-200	Chond2	TRA2B
1.05E-199	0.30377666	0.194	0.345	2.65E-195	Chond2	LAMB2
9.84E-198	0.82642721	0.469	0.533	2.48E-193	Chond2	MEG3
3.21E-197	0.2586786	0.185	0.322	8.10E-193	Chond2	NFKBIZ
2.23E-189	0.30775003	0.123	0.228	5.63E-185	Chond2	CDK13
1.75E-187	0.27237031	0.151	0.271	4.41E-183	Chond2	GPC6
1.66E-186	0.32529078	0.293	0.508	4.18E-182	Chond2	PAPSS2
3.84E-184	0.25038785	0.332	0.571	9.69E-180	Chond2	ENPP1
1.12E-180	0.42042757	0.248	0.438	2.84E-176	Chond2	BCLAF1
1.61E-179	0.28625226	0.108	0.201	4.05E-175	Chond2	RBM5
3.80E-176	0.36348716	0.212	0.364	9.59E-172	Chond2	AC020916.1
5.46E-164	0.39102067	0.179	0.311	1.38E-159	Chond2	SMC5
2.19E-161	0.38282827	0.657	0.783	5.52E-157	Chond2	CTGF
2.80E-161	0.26765012	0.371	0.635	7.08E-157	Chond2	HIF1A
1.09E-159	0.39643292	0.328	0.579	2.75E-155	Chond2	ZRANB2
8.01E-159	0.3453295	0.173	0.299	2.02E-154	Chond2	SOX5
3.04E-158	0.29815099	0.965	0.976	7.68E-154	Chond2	CLU
9.43E-155	0.28699027	0.124	0.218	2.38E-150	Chond2	TNRC6A
5.00E-153	0.39051164	0.164	0.282	1.26E-148	Chond2	SLC20A1
3.71E-150	0.67870647	0.509	0.662	9.36E-146	Chond2	CCNL1
1.40E-147	0.2914076	0.122	0.214	3.53E-143	Chond2	CSPG4
6.45E-147	0.29948933	0.12	0.211	1.63E-142	Chond2	CCDC14
8.81E-145	0.42392306	0.242	0.41	2.22E-140	Chond2	BCL6
1.48E-138	0.40501962	0.283	0.478	3.73E-134	Chond2	CCDC88A
3.28E-134	0.28743689	0.121	0.207	8.28E-130	Chond2	CDH19

6.79E-134	0.37159144	0.161	0.271	1.71E-129	Chond2	PER1
1.09E-119	0.39720696	0.237	0.391	2.75E-115	Chond2	ITGA10
2.48E-116	0.36083907	0.215	0.35	6.25E-112	Chond2	XYLT1
6.92E-113	0.48464047	0.248	0.407	1.75E-108	Chond2	SYNE1
1.42E-107	0.41716837	0.28	0.46	3.57E-103	Chond2	AKAP9
5.19E-104	0.40937003	0.377	0.646	1.31E-99	Chond2	SFPQ
3.81E-99	0.27973254	0.446	0.764	9.61E-95	Chond2	RBM39
1.46E-97	0.50036014	0.224	0.362	3.68E-93	Chond2	ZNF207
1.30E-83	0.4096217	0.643	0.864	3.29E-79	Chond2	MT-ND5
8.37E-81	0.4135085	0.291	0.464	2.11E-76	Chond2	TRPS1
1.30E-75	0.60638809	0.458	0.586	3.29E-71	Chond2	COL11A2
1.65E-75	0.51475128	0.256	0.406	4.15E-71	Chond2	LINC-PINT
7.34E-69	0.45901474	0.355	0.572	1.85E-64	Chond2	MCL1
6.10E-64	0.52975899	0.312	0.499	1.54E-59	Chond2	RNMT
1.09E-62	0.46969899	0.348	0.559	2.75E-58	Chond2	GOLGB1
1.03E-48	0.53139466	0.339	0.542	2.61E-44	Chond2	GLS
8.88E-45	0.53378355	0.256	0.387	2.24E-40	Chond2	SREK1
1.30E-43	0.55232428	0.237	0.353	3.28E-39	Chond2	COL27A1
7.13E-22	0.34157393	0.446	0.705	1.80E-17	Chond2	RRBP1
1.62E-20	0.42989595	0.177	0.236	4.08E-16	Chond2	CILP2
5.12E-20	0.55279648	0.29	0.415	1.29E-15	Chond2	NOVA1
3.98E-10	0.57794749	0.338	0.498	1.01E-05	Chond2	AHI1
2.45E-07	0.51196851	0.16	0.202	0.0061838	Chond2	AC058791.1
0	0.95715016	0.654	0.325	0	Chond3	CHRDL2
0	0.8974478	0.858	0.423	0	Chond3	CAPS
0	0.88968943	0.959	0.637	0	Chond3	NDUFA4L2
0	0.78910086	0.865	0.337	0	Chond3	ABI3BP
0	0.75565813	0.938	0.617	0	Chond3	CNMD
0	0.74259315	0.537	0.218	0	Chond3	CHI3L2
0	0.73307433	0.977	0.847	0	Chond3	MT1E
0	0.6859339	0.997	0.919	0	Chond3	MT1X
0	0.67910928	0.804	0.263	0	Chond3	VCAN
0	0.65244609	0.442	0.11	0	Chond3	CRISPLD1
0	0.651794	0.521	0.161	0	Chond3	NRN1
0	0.62962588	0.806	0.295	0	Chond3	SERPINA5
0	0.62865385	0.852	0.42	0	Chond3	CP
0	0.59749399	0.895	0.532	0	Chond3	NNMT
0	0.59288041	0.893	0.498	0	Chond3	CYR61
0	0.56635846	0.888	0.41	0	Chond3	PLXDC2
0	0.55939191	0.991	0.965	0	Chond3	MT2A
0	0.55144066	0.855	0.524	0	Chond3	KLF2
0	0.54092602	0.987	0.879	0	Chond3	RPL13A
0	0.53903926	0.401	0.209	0	Chond3	CHI3L1
0	0.52928409	0.975	0.693	0	Chond3	BGN
0	0.52168482	0.917	0.541	0	Chond3	HTRA1
0	0.51349354	0.644	0.259	0	Chond3	ADM
0	0.50913643	0.468	0.11	0	Chond3	SPON2
0	0.50406106	0.213	0.049	0	Chond3	ORM1
0	0.50093718	0.979	0.808	0	Chond3	RPL7
0	0.49745662	0.642	0.167	0	Chond3	ANGPTL5
0	0.46939831	0.856	0.466	0	Chond3	VKORC1

0	0.46488531	0.565	0.231	0	Chond3	SLC30A1
0	0.46230606	0.925	0.571	0	Chond3	EID1
0	0.46145739	0.815	0.396	0	Chond3	CTSD
0	0.45928096	0.685	0.294	0	Chond3	BNIP3
0	0.45764215	0.919	0.538	0	Chond3	PCOLCE2
0	0.45532997	0.992	0.851	0	Chond3	PRELP
0	0.44527579	0.746	0.347	0	Chond3	ID1
0	0.44124895	0.983	0.849	0	Chond3	FOS
0	0.43802786	0.757	0.338	0	Chond3	P4HA1
0	0.4369712	0.928	0.594	0	Chond3	EIF3E
0	0.43172242	1	0.979	0	Chond3	EEF1A1
0	0.41650534	0.835	0.486	0	Chond3	SERPINE2
0	0.41584589	0.611	0.178	0	Chond3	PAX1
0	0.41436938	0.982	0.832	0	Chond3	RPS20
0	0.41432465	0.989	0.85	0	Chond3	RPLP0
0	0.40916356	0.843	0.446	0	Chond3	ENO1
0	0.40814308	0.991	0.875	0	Chond3	RPL3
0	0.40387258	0.882	0.511	0	Chond3	AEBP1
0	0.40359601	0.992	0.88	0	Chond3	RPS2
0	0.39732262	0.729	0.299	0	Chond3	ISLR
0	0.39621188	0.496	0.173	0	Chond3	TIMP4
0	0.39307223	0.843	0.464	0	Chond3	PRDX4
0	0.39253643	1	0.972	0	Chond3	FTL
0	0.39004764	0.625	0.348	0	Chond3	MT1F
0	0.38322215	0.715	0.318	0	Chond3	PGK1
0	0.38322006	0.543	0.139	0	Chond3	P4HA2
0	0.38068749	0.82	0.43	0	Chond3	PDIA6
0	0.37540748	0.811	0.407	0	Chond3	P4HB
0	0.37496738	1	0.973	0	Chond3	TPT1
0	0.37215249	0.884	0.478	0	Chond3	FBXO2
0	0.37053954	0.993	0.859	0	Chond3	SCRG1
0	0.36917666	0.833	0.429	0	Chond3	FXYD6
0	0.36453709	0.934	0.592	0	Chond3	COL9A3
0	0.36153154	0.474	0.19	0	Chond3	OTUD1
0	0.3610069	0.872	0.524	0	Chond3	KLF4
0	0.35902146	0.675	0.304	0	Chond3	TIPARP
0	0.35814497	0.998	0.922	0	Chond3	RPL15
0	0.35764875	0.511	0.165	0	Chond3	METRN
0	0.35759775	0.864	0.558	0	Chond3	DUSP1
0	0.35718211	0.959	0.715	0	Chond3	EEF1D
0	0.35678129	0.622	0.192	0	Chond3	COL5A2
0	0.35515664	0.698	0.34	0	Chond3	ID2
0	0.35432183	0.53	0.158	0	Chond3	SNAI2
0	0.35400514	0.824	0.539	0	Chond3	IER2
0	0.35377244	0.79	0.395	0	Chond3	ATRAID
0	0.35093141	0.806	0.412	0	Chond3	SSR2
0	0.34978718	0.938	0.721	0	Chond3	JUNB
0	0.34852341	0.941	0.711	0	Chond3	FOSB
0	0.34531752	0.948	0.638	0	Chond3	S100B
0	0.34506649	0.964	0.743	0	Chond3	RPSA
0	0.34346261	0.994	0.889	0	Chond3	RPL5

0	0.34314256	0.631	0.249	0	Chond3	CLEC11A
0	0.33951067	0.839	0.486	0	Chond3	RHOB
0	0.33813069	0.632	0.271	0	Chond3	BAMBI
0	0.33568505	0.998	0.94	0	Chond3	RPL19
0	0.3349544	0.69	0.263	0	Chond3	DPP7
0	0.33492293	0.894	0.55	0	Chond3	NPC2
0	0.33075461	0.701	0.387	0	Chond3	BTG2
0	0.33050507	0.404	0.17	0	Chond3	MATN4
0	0.32688519	0.949	0.668	0	Chond3	EIF3H
0	0.32686234	0.906	0.758	0	Chond3	JUN
0	0.32598844	0.748	0.331	0	Chond3	SDC2
0	0.32308446	0.853	0.482	0	Chond3	ITM2A
0	0.32186436	0.585	0.216	0	Chond3	INHBA
0	0.32124369	0.932	0.639	0	Chond3	RPS4Y1
0	0.32012277	0.762	0.436	0	Chond3	CCDC80
0	0.31921967	0.998	0.92	0	Chond3	RPL8
0	0.31817025	0.678	0.327	0	Chond3	FHL1
0	0.31752751	0.921	0.605	0	Chond3	CD99
0	0.31320428	0.993	0.889	0	Chond3	RPS16
0	0.31143006	0.598	0.209	0	Chond3	BARX1
0	0.31122906	0.44	0.101	0	Chond3	CD82
0	0.31053297	0.995	0.904	0	Chond3	RPS3
0	0.30903237	0.994	0.894	0	Chond3	RPS11
0	0.30168332	0.788	0.388	0	Chond3	CRTAP
0	0.3004555	0.448	0.092	0	Chond3	THY1
0	0.30013102	0.921	0.611	0	Chond3	EEF2
0	0.3000316	0.995	0.899	0	Chond3	RPL7A
0	0.29976663	0.429	0.095	0	Chond3	AL035258.1
0	0.29804968	0.998	0.949	0	Chond3	RPS3A
0	0.29739985	0.865	0.502	0	Chond3	BEX3
0	0.29688622	0.907	0.602	0	Chond3	GAS5
0	0.29598471	0.707	0.31	0	Chond3	TMEM167A
0	0.29526905	0.985	0.832	0	Chond3	RPS5
0	0.29520003	0.265	0.037	0	Chond3	WIF1
0	0.29499559	0.759	0.361	0	Chond3	SDF4
0	0.29497157	1	0.97	0	Chond3	RPL10
0	0.29275121	0.98	0.788	0	Chond3	EEF1B2
0	0.29227605	0.861	0.501	0	Chond3	TOMM20
0	0.29153306	0.999	0.944	0	Chond3	RPS6
0	0.2912061	0.916	0.639	0	Chond3	AL078639.1
0	0.29059904	0.996	0.903	0	Chond3	RPL23A
0	0.28973973	0.382	0.075	0	Chond3	AGT
0	0.28785517	0.581	0.203	0	Chond3	GALNT1
0	0.28702369	0.814	0.438	0	Chond3	PKM
0	0.28698042	0.393	0.068	0	Chond3	ATRNL1
0	0.2853637	0.759	0.383	0	Chond3	EIF4B
0	0.28484222	0.563	0.22	0	Chond3	PPIC
0	0.27805889	0.838	0.472	0	Chond3	TMED10
0	0.27714186	0.84	0.491	0	Chond3	SSR3
0	0.27645504	0.737	0.346	0	Chond3	WWP2
0	0.27636387	0.888	0.685	0	Chond3	SPARC

0	0.27592946	0.843	0.48	0	Chond3	RPL17
0	0.27532885	0.942	0.646	0	Chond3	TMEM59
0	0.27456517	0.456	0.119	0	Chond3	ATP1B1
0	0.27385192	0.742	0.329	0	Chond3	OS9
0	0.27288977	0.883	0.548	0	Chond3	ST13
0	0.2709246	0.968	0.749	0	Chond3	GAPDH
0	0.26886427	0.975	0.764	0	Chond3	NPM1
0	0.2684548	0.937	0.692	0	Chond3	RPL31
0	0.26820399	0.722	0.337	0	Chond3	RCN2
0	0.26691439	0.645	0.251	0	Chond3	TGOLN2
0	0.26691251	0.762	0.386	0	Chond3	TMED9
0	0.26530991	0.891	0.561	0	Chond3	PPDPF
0	0.2647291	0.759	0.391	0	Chond3	CPE
0	0.26420525	0.994	0.891	0	Chond3	RPS9
0	0.26403869	0.7	0.32	0	Chond3	EDIL3
0	0.26256429	0.997	0.916	0	Chond3	RPS4X
0	0.26246054	0.234	0.026	0	Chond3	CA9
0	0.26155912	0.361	0.067	0	Chond3	BAALC
0	0.26125208	0.949	0.71	0	Chond3	CD81
0	0.26014736	0.625	0.246	0	Chond3	RNF130
0	0.25907559	0.669	0.295	0	Chond3	TMED4
0	0.25888633	0.959	0.71	0	Chond3	LRRC75A-AS1
0	0.25809265	0.987	0.848	0	Chond3	RPL10A
0	0.25764373	0.817	0.509	0	Chond3	H1FX
0	0.25701335	0.325	0.051	0	Chond3	NRTN
0	0.2541376	0.937	0.646	0	Chond3	HLA-A
0	0.2534852	0.809	0.49	0	Chond3	HAPLN1
0	0.25341801	0.263	0.047	0	Chond3	DPT
0	0.25300466	0.939	0.652	0	Chond3	PSAP
0	0.25098241	0.939	0.659	0	Chond3	RPL4
0	0.25029096	0.758	0.394	0	Chond3	PRDX2
1.91E-298	0.4805268	0.848	0.641	4.82E-294	Chond3	SLPI
2.25E-297	0.38494564	0.855	0.728	5.68E-293	Chond3	MT1G
2.95E-278	0.45583648	0.643	0.491	7.45E-274	Chond3	MT1M
5.97E-275	0.26075786	0.33	0.214	1.51E-270	Chond3	MT1A
0	1.50026039	0.24	0.014	0	Noto	KRT19
0	1.49795615	0.26	0.004	0	Noto	CA3
0	1.29541032	0.461	0.051	0	Noto	EPYC
0	0.83607511	0.589	0.052	0	Noto	ENPP2
0	0.75828293	0.226	0.017	0	Noto	CD24
0	0.73678347	0.446	0.04	0	Noto	CTHRC1
0	0.72676225	0.342	0.021	0	Noto	ACTC1
0	0.69234084	0.315	0.024	0	Noto	PCSK2
0	0.69099417	0.478	0.048	0	Noto	RAPGEF5
0	0.6333415	0.319	0.002	0	Noto	RAB3B
0	0.63161627	0.283	0.012	0	Noto	HOPX
0	0.62715023	0.567	0.119	0	Noto	SUSD5
0	0.61134274	0.45	0.001	0	Noto	LINC01896
0	0.6025523	0.458	0.023	0	Noto	BASP1
0	0.60247994	0.408	0.048	0	Noto	PCDH9
0	0.58846828	0.358	0.002	0	Noto	TBXT

0	0.51633473	0.419	0.059	0	Noto	ANK3
0	0.4374727	0.478	0.068	0	Noto	VAMP8
0	0.43535827	0.206	0.017	0	Noto	C1QTNF3
0	0.43262098	0.315	0.022	0	Noto	EHD3
0	0.42283795	0.294	0.001	0	Noto	SALL3
0	0.41768681	0.321	0.01	0	Noto	GALNT3
0	0.34713334	0.294	0.025	0	Noto	ALPK2
0	0.34295265	0.29	0.024	0	Noto	SPOCK1
0	0.335168	0.252	0.009	0	Noto	CLVS2
0	0.33389017	0.228	0.018	0	Noto	TNFSF13B
0	0.32818536	0.268	0.024	0	Noto	SAMD11
0	0.32558303	0.268	0.02	0	Noto	NDNF
0	0.26975155	0.233	0.008	0	Noto	BARX2
1.24E-295	0.62592009	0.62	0.144	3.13E-291	Noto	SESN3
2.56E-287	0.34391653	0.205	0.019	6.47E-283	Noto	S100P
3.20E-271	0.26086617	0.251	0.028	8.06E-267	Noto	ST8SIA1
8.15E-264	0.53433423	0.547	0.122	2.06E-259	Noto	LIMCH1
3.19E-259	0.42558048	0.228	0.025	8.06E-255	Noto	ALCAM
5.07E-241	0.35179511	0.279	0.038	1.28E-236	Noto	TMEM56
1.27E-236	0.29321081	0.264	0.035	3.21E-232	Noto	SBSPON
5.13E-227	0.2790858	0.29	0.043	1.29E-222	Noto	ODF3B
7.27E-226	0.8840594	0.832	0.331	1.83E-221	Noto	CD109
1.57E-216	0.46490032	0.283	0.044	3.96E-212	Noto	FNDC1
1.80E-178	0.30921325	0.319	0.062	4.53E-174	Noto	TSPAN5
3.90E-174	0.51316493	0.496	0.138	9.83E-170	Noto	HSPB8
4.63E-158	0.28860993	0.237	0.04	1.17E-153	Noto	NAV2
7.98E-154	0.46154471	0.995	0.941	2.01E-149	Noto	RPL12
1.51E-151	0.36293108	0.468	0.133	3.80E-147	Noto	MIR497HG
1.12E-150	0.32774222	0.296	0.061	2.84E-146	Noto	CLDN11
7.03E-147	0.40423103	0.411	0.111	1.77E-142	Noto	C3orf58
4.54E-142	0.79000735	0.84	0.444	1.15E-137	Noto	TNFRSF11B
1.58E-138	0.60945968	0.778	0.358	3.98E-134	Noto	SLC26A2
2.07E-138	0.42850455	0.635	0.238	5.23E-134	Noto	C1QBP
6.12E-133	0.57501982	0.854	0.451	1.54E-128	Noto	RHOBTB3
1.17E-128	0.26138703	0.404	0.114	2.95E-124	Noto	DSG2
1.45E-127	0.350635	0.447	0.136	3.66E-123	Noto	JDP2
1.97E-119	0.46857719	0.562	0.213	4.98E-115	Noto	DUSP14
3.38E-114	0.36752454	0.482	0.166	8.53E-110	Noto	NEDD9
3.71E-114	0.31436533	0.427	0.131	9.35E-110	Noto	LAG3
7.70E-106	0.58364621	0.683	0.311	1.94E-101	Noto	SERPINE1
5.06E-105	0.26870513	0.431	0.141	1.28E-100	Noto	TMEM43
2.30E-104	0.2918393	0.392	0.122	5.80E-100	Noto	XPC
1.01E-103	0.55552181	0.833	0.458	2.56E-99	Noto	CTSL
1.19E-103	0.28676652	0.454	0.152	3.00E-99	Noto	ZNF385B
4.64E-102	0.29615235	0.465	0.162	1.17E-97	Noto	FAM89A
7.89E-98	0.29297193	0.303	0.084	1.99E-93	Noto	NABP1
5.94E-96	0.32107069	0.606	0.252	1.50E-91	Noto	ZNF385D
4.23E-94	0.45584653	0.923	0.662	1.07E-89	Noto	PEBP1
3.51E-90	0.35687887	0.999	0.981	8.85E-86	Noto	RPS12
1.05E-88	0.42072606	0.654	0.303	2.64E-84	Noto	LRRC75A
1.78E-87	0.32586969	0.588	0.256	4.50E-83	Noto	RAPH1

2.51E-85	0.28961954	0.547	0.229	6.32E-81	Noto	YIF1A
1.32E-82	0.26048953	0.369	0.126	3.34E-78	Noto	GLDN
3.63E-82	0.28466223	0.457	0.176	9.16E-78	Noto	KMT5A
4.70E-80	0.26690118	0.54	0.225	1.19E-75	Noto	STK38L
3.49E-76	0.3167952	0.997	0.95	8.81E-72	Noto	RPS13
6.86E-76	0.28341832	0.66	0.317	1.73E-71	Noto	MLEC
2.16E-75	0.32249826	0.491	0.207	5.44E-71	Noto	ING2
2.83E-74	0.34750335	0.698	0.361	7.13E-70	Noto	MDFI
1.01E-73	0.33194411	1	0.95	2.55E-69	Noto	H3F3B
8.18E-72	0.36094382	0.763	0.435	2.06E-67	Noto	UQCR10
1.52E-71	0.38555541	0.811	0.516	3.83E-67	Noto	TMEM14C
9.55E-71	0.26376104	0.539	0.24	2.41E-66	Noto	LRRC59
5.30E-68	0.26997303	0.702	0.366	1.34E-63	Noto	STMP1
7.12E-67	0.27530209	0.997	0.955	1.80E-62	Noto	RPL21
8.35E-66	0.30387966	0.744	0.413	2.11E-61	Noto	HSBP1
1.11E-65	0.25879259	0.429	0.177	2.81E-61	Noto	FOXD1
1.26E-65	0.44704601	0.695	0.383	3.17E-61	Noto	CD164
3.08E-64	0.29230787	0.586	0.289	7.77E-60	Noto	RIOK3
2.34E-63	0.67161898	0.978	0.814	5.92E-59	Noto	ANXA1
1.91E-60	0.35744247	0.72	0.405	4.81E-56	Noto	MIDN
2.67E-59	0.39407053	0.829	0.555	6.74E-55	Noto	RPS27L
1.14E-58	0.25225346	0.473	0.212	2.88E-54	Noto	TRIM47
2.48E-58	0.29157186	0.278	0.099	6.26E-54	Noto	ATP6V1B2
1.58E-56	0.31828603	0.792	0.497	3.98E-52	Noto	YWHAQ
7.16E-54	0.27840751	0.813	0.508	1.81E-49	Noto	RBM3
4.46E-53	0.32492593	0.77	0.495	1.13E-48	Noto	C6orf48
1.81E-52	0.27167356	0.993	0.945	4.56E-48	Noto	RPL18
2.42E-52	0.312974	0.352	0.147	6.10E-48	Noto	HIST1H2AC
2.02E-51	0.28809758	0.964	0.768	5.11E-47	Noto	CIRBP
4.17E-51	0.26717999	0.856	0.57	1.05E-46	Noto	PARK7
2.72E-48	0.62820714	0.856	0.607	6.87E-44	Noto	EMP1
8.29E-48	0.33574744	0.309	0.132	2.09E-43	Noto	FKBP5
6.49E-47	0.27283811	0.795	0.498	1.64E-42	Noto	SERTAD1
2.06E-44	0.33999603	0.732	0.441	5.20E-40	Noto	TSC22D3
1.68E-41	0.25890556	0.977	0.849	4.24E-37	Noto	RPS10
2.64E-40	0.35521484	0.519	0.291	6.65E-36	Noto	CKB
1.61E-38	0.39665715	0.786	0.531	4.05E-34	Noto	MYADM
0	2.76123237	0.789	0.053	0	EC	CD74
0	2.72542122	0.916	0.02	0	EC	IFI27
0	2.35519161	0.899	0.008	0	EC	PECAM1
0	2.18876872	0.876	0.081	0	EC	RAMP2
0	2.14940032	0.932	0.253	0	EC	GNG11
0	2.04356492	0.79	0.015	0	EC	CALCRL
0	1.97747671	0.694	0.005	0	EC	VWF
0	1.90570528	0.804	0.008	0	EC	EGFL7
0	1.80745191	0.377	0.005	0	EC	STC1
0	1.7816071	0.952	0.445	0	EC	HLA-E
0	1.7573988	0.614	0.018	0	EC	HLA-DRA
0	1.6649769	0.795	0.132	0	EC	HSPG2
0	1.66013943	0.682	0.003	0	EC	SOX18
0	1.62652952	0.701	0.191	0	EC	SLC9A3R2

0	1.60400977	0.657	0.062	0	EC	HLA-DRB1
0	1.60016395	0.745	0.05	0	EC	ITGA6
0	1.57438983	0.747	0.003	0	EC	EMCN
0	1.55937917	0.827	0.158	0	EC	NPDC1
0	1.51666399	0.543	0.009	0	EC	PLAT
0	1.51092839	0.686	0.068	0	EC	FLT1
0	1.50449552	0.478	0.009	0	EC	CLDN5
0	1.49567086	0.785	0.104	0	EC	ELK3
0	1.48090167	0.616	0.005	0	EC	PODXL
0	1.47451568	0.699	0.003	0	EC	ECSCR
0	1.41341335	0.833	0.283	0	EC	TGFBR2
0	1.39496504	0.569	0.021	0	EC	RAMP3
0	1.38264585	0.976	0.718	0	EC	HLA-B
0	1.36911353	0.709	0.113	0	EC	PALMD
0	1.36789433	0.858	0.351	0	EC	CRIP2
0	1.36100084	0.644	0.01	0	EC	CD34
0	1.28358897	0.537	0.08	0	EC	KCTD12
0	1.26912212	0.463	0.014	0	EC	IL32
0	1.25194551	0.548	0.04	0	EC	HLA-DPA1
0	1.243763	0.618	0.005	0	EC	MMRN2
0	1.23894107	0.624	0.003	0	EC	PCAT19
0	1.23710812	0.679	0.073	0	EC	MGST2
0	1.22468321	0.452	0.003	0	EC	APLNR
0	1.21424059	0.619	0.158	0	EC	PRCP
0	1.17264329	0.574	0.076	0	EC	HYAL2
0	1.16845551	0.591	0.006	0	EC	LDB2
0	1.16097191	0.797	0.372	0	EC	TPM3
0	1.15087258	0.548	0.023	0	EC	HEG1
0	1.14401871	0.618	0.061	0	EC	ADAM15
0	1.13504757	0.566	0.002	0	EC	CYYR1
0	1.12196802	0.953	0.729	0	EC	HLA-C
0	1.11749271	0.217	0.001	0	EC	SELE
0	1.11594518	0.519	0.01	0	EC	TSPAN7
0	1.11230545	0.698	0.193	0	EC	VAMP5
0	1.09948679	0.553	0.008	0	EC	LPAR6
0	1.09787191	0.546	0.002	0	EC	CDH5
0	1.09013228	0.541	0.016	0	EC	JAM2
0	1.08797231	0.487	0.026	0	EC	THBD
0	1.07796139	0.416	0.009	0	EC	RBP7
0	1.07126212	0.554	0.002	0	EC	CXorf36
0	1.06987932	0.516	0.027	0	EC	ICAM2
0	1.06062134	0.518	0.002	0	EC	GIMAP1
0	1.05069011	0.541	0.003	0	EC	TIE1
0	1.04925981	0.565	0.015	0	EC	LMO2
0	1.0378173	0.537	0.039	0	EC	SLFN5
0	1.02879032	0.805	0.399	0	EC	TAGLN2
0	1.02808092	0.419	0.01	0	EC	CAVIN2
0	1.01564275	0.681	0.221	0	EC	ETS2
0	1.0156356	0.471	0.028	0	EC	PCDH17
0	1.00541283	0.608	0.11	0	EC	BMPR2
0	1.0030733	0.502	0.004	0	EC	PTPRB

0	0.99103709	0.474	0.002	0	EC	KDR
0	0.98978212	0.422	0.019	0	EC	EFNB2
0	0.98317732	0.507	0.001	0	EC	SHANK3
0	0.96917195	0.475	0.008	0	EC	FAM198B
0	0.96803587	0.512	0.001	0	EC	MYCT1
0	0.96638224	0.768	0.377	0	EC	IL6ST
0	0.965914	0.257	0.003	0	EC	DNASE1L3
0	0.96526852	0.658	0.172	0	EC	RAB11A
0	0.96125053	0.619	0.152	0	EC	SH3BP5
0	0.94829327	0.808	0.438	0	EC	GNAI2
0	0.93018512	0.478	0.013	0	EC	PREX2
0	0.91928315	0.513	0.138	0	EC	DUSP23
0	0.91010353	0.659	0.251	0	EC	MSN
0	0.90935436	0.286	0.005	0	EC	IFI44L
0	0.89816265	0.787	0.402	0	EC	APP
0	0.89254221	0.623	0.176	0	EC	ARPC1B
0	0.88824516	0.432	0.036	0	EC	NOTCH4
0	0.88195501	0.4	0.004	0	EC	MCTP1
0	0.87516514	0.562	0.121	0	EC	GNAQ
0	0.87492974	0.496	0.038	0	EC	PSMB9
0	0.86835547	0.382	0.005	0	EC	THSD7A
0	0.86396761	0.627	0.215	0	EC	ETS1
0	0.86388284	0.39	0.005	0	EC	SEMA6A
0	0.85772407	0.58	0.196	0	EC	MTUS1
0	0.8564932	0.321	0.003	0	EC	SLCO2A1
0	0.84743434	0.724	0.391	0	EC	CCDC85B
0	0.84359997	0.555	0.123	0	EC	PIK3C2A
0	0.83419328	0.447	0.014	0	EC	S1PR1
0	0.8239655	0.35	0.065	0	EC	LIFR
0	0.81770698	0.376	0.02	0	EC	ITGB4
0	0.81204073	0.431	0.017	0	EC	CDH13
0	0.80704464	0.646	0.261	0	EC	LIMS1
0	0.80594014	0.589	0.143	0	EC	TJP1
0	0.79777371	0.433	0.025	0	EC	GMFG
0	0.797734	0.569	0.16	0	EC	CFLAR
0	0.79194124	0.396	0.006	0	EC	ENTPD1
0	0.79004975	0.537	0.119	0	EC	PHACTR2
0	0.7899589	0.397	0.01	0	EC	LIMS2
0	0.78855258	0.538	0.23	0	EC	SOX4
0	0.78718794	0.679	0.332	0	EC	RDX
0	0.7857444	0.435	0.073	0	EC	CRYBG3
0	0.78382169	0.529	0.118	0	EC	SSFA2
0	0.78303152	0.419	0.078	0	EC	LYST
0	0.78219965	0.488	0.097	0	EC	LAP3
0	0.78079477	0.446	0.004	0	EC	IL3RA
0	0.77926745	0.735	0.447	0	EC	RHOC
0	0.77691197	0.464	0.049	0	EC	PTPRM
0	0.77666691	0.346	0.004	0	EC	GBP4
0	0.76889207	0.482	0.102	0	EC	NCOA7
0	0.76737518	0.428	0.025	0	EC	DOCK9
0	0.76728799	0.552	0.171	0	EC	S100A16

0	0.76684203	0.621	0.236	0	EC	GNB1
0	0.76560587	0.428	0.173	0	EC	INSR
0	0.76542028	0.519	0.23	0	EC	RF00100.4
0	0.76210776	0.374	0.007	0	EC	FAM167B
0	0.76182471	0.533	0.099	0	EC	SULF2
0	0.76147936	0.492	0.074	0	EC	KIAA0355
0	0.75952147	0.917	0.718	0	EC	ATP5F1E
0	0.75766768	0.51	0.104	0	EC	HLA-F
0	0.75710148	0.445	0.042	0	EC	RAI14
0	0.75470091	0.407	0.004	0	EC	DOCK4
0	0.75376969	0.805	0.58	0	EC	MYL12B
0	0.75311692	0.373	0.001	0	EC	TEK
0	0.75104636	0.357	0.009	0	EC	NES
0	0.74815772	0.777	0.418	0	EC	CAST
0	0.74793051	0.43	0.057	0	EC	LMCD1
0	0.7474535	0.462	0.029	0	EC	HDAC7
0	0.74618887	0.388	0.018	0	EC	ADAMTS9
0	0.74192537	0.391	0.002	0	EC	ROBO4
0	0.73928573	0.435	0.044	0	EC	SMAD1
0	0.73883148	0.473	0.126	0	EC	DUSP6
0	0.73837176	0.357	0.001	0	EC	TM4SF18
0	0.73730663	0.773	0.434	0	EC	KTN1
0	0.73700221	0.414	0.021	0	EC	FLI1
0	0.73494548	0.427	0.134	0	EC	VWA1
0	0.7340568	0.942	0.813	0	EC	SRP14
0	0.73355325	0.404	0.021	0	EC	PLCB1
0	0.73004113	0.382	0.137	0	EC	TGM2
0	0.72373081	0.451	0.042	0	EC	ERG
0	0.72320663	0.552	0.168	0	EC	ZEB1
0	0.72078771	0.653	0.318	0	EC	CLIC4
0	0.71674684	0.512	0.102	0	EC	AP1S2
0	0.71594469	0.453	0.06	0	EC	FLNB
0	0.71415878	0.375	0.003	0	EC	ADCY4
0	0.7139036	0.56	0.16	0	EC	PTPN12
0	0.71308887	0.445	0.066	0	EC	SYNPO
0	0.71251059	0.499	0.164	0	EC	ARL4A
0	0.71211761	0.435	0.114	0	EC	HLA-DPB1
0	0.71101066	0.393	0.004	0	EC	KANK3
0	0.70951764	0.417	0.061	0	EC	PARP14
0	0.70663226	0.642	0.283	0	EC	CDC37
0	0.70532963	0.455	0.075	0	EC	FRYL
0	0.70506682	0.415	0.025	0	EC	GRASP
0	0.70471832	0.401	0.026	0	EC	JCAD
0	0.69315452	0.388	0.02	0	EC	ADGRL2
0	0.69003158	0.556	0.177	0	EC	CCDC50
0	0.68646498	0.369	0.014	0	EC	FAM110D
0	0.68630902	0.303	0.003	0	EC	NOSTRIN
0	0.68572523	0.371	0.046	0	EC	MALL
0	0.6822064	0.399	0.026	0	EC	NOTCH1
0	0.68151671	0.335	0.007	0	EC	KCNN3
0	0.68136649	0.469	0.105	0	EC	BAZ2B

0	0.68019978	0.341	0.002	0	EC	SOX7
0	0.67949587	0.554	0.177	0	EC	PLEC
0	0.67934607	0.716	0.354	0	EC	ZFP36L2
0	0.67744812	0.8	0.533	0	EC	RHOA
0	0.67586566	0.337	0.002	0	EC	SHE
0	0.66755125	0.361	0.02	0	EC	AKR1C3
0	0.66251224	0.503	0.125	0	EC	CMIP
0	0.66166509	0.403	0.077	0	EC	NUAK1
0	0.66164697	0.35	0.085	0	EC	PXDN
0	0.66002599	0.624	0.27	0	EC	CAPNS1
0	0.65729864	0.385	0.012	0	EC	RASIP1
0	0.65534142	0.784	0.485	0	EC	FXYD5
0	0.65295007	0.809	0.57	0	EC	GUK1
0	0.65208701	0.484	0.124	0	EC	MAP4K4
0	0.64857308	0.278	0.078	0	EC	LTC4S
0	0.64753482	0.474	0.124	0	EC	SHC1
0	0.64338504	0.679	0.402	0	EC	TUBB
0	0.64224504	0.335	0.005	0	EC	CLIC2
0	0.64032593	0.407	0.052	0	EC	PXN
0	0.63642038	0.29	0.032	0	EC	HLA-DMA
0	0.6352068	0.448	0.165	0	EC	FSCN1
0	0.63476752	0.354	0.045	0	EC	CTSH
0	0.63414864	0.272	0.003	0	EC	PDGFB
0	0.63272598	0.559	0.273	0	EC	PPP1R14B
0	0.63145348	0.438	0.086	0	EC	ITGA5
0	0.62814862	0.552	0.199	0	EC	MBNL2
0	0.62807581	0.35	0.022	0	EC	RALGAPA2
0	0.62606051	0.598	0.254	0	EC	ACTR2
0	0.62448986	0.218	0.004	0	EC	C2CD4B
0	0.6237589	0.359	0.04	0	EC	PKP4
0	0.62365586	0.386	0.027	0	EC	CARD8
0	0.6180747	0.411	0.071	0	EC	EHD4
0	0.61118917	0.349	0.008	0	EC	FGD5
0	0.61111053	0.445	0.083	0	EC	CTNND1
0	0.61047503	0.28	0.026	0	EC	TMTC1
0	0.60717999	0.42	0.084	0	EC	ELK4
0	0.60528142	0.311	0.01	0	EC	RAPGEF3
0	0.60115834	0.631	0.314	0	EC	WWTR1
0	0.60106905	0.432	0.068	0	EC	ADAM10
0	0.59946744	0.335	0.004	0	EC	HHEX
0	0.59873943	0.358	0.025	0	EC	PPM1F
0	0.59808062	0.491	0.171	0	EC	WNK1
0	0.59649745	0.323	0.001	0	EC	BCL6B
0	0.59578194	0.37	0.027	0	EC	TSPAN14
0	0.59550052	0.463	0.181	0	EC	CALCOCO2
0	0.5954118	0.456	0.178	0	EC	GYPC
0	0.59489451	0.293	0.003	0	EC	MECOM
0	0.5945412	0.478	0.156	0	EC	SKAP2
0	0.59448694	0.287	0.005	0	EC	AP002004.1
0	0.59257117	0.651	0.317	0	EC	ATRX
0	0.59256486	0.262	0.013	0	EC	RASA4

0	0.59142356	0.512	0.17	0	EC	LUZP1
0	0.59108299	0.379	0.057	0	EC	CEP68
0	0.58726689	0.571	0.264	0	EC	CRIM1
0	0.5826646	0.387	0.039	0	EC	MAP3K11
0	0.58158528	0.36	0.03	0	EC	TMEM204
0	0.58120031	0.318	0.011	0	EC	NHSL2
0	0.57977929	0.375	0.062	0	EC	KIAA1551
0	0.57912055	0.781	0.526	0	EC	CDC42
0	0.57818879	0.474	0.16	0	EC	SLK
0	0.57590319	0.533	0.218	0	EC	FLOT1
0	0.57530667	0.258	0.024	0	EC	ARHGAP18
0	0.5746655	0.441	0.133	0	EC	VPS13C
0	0.57395959	0.325	0.012	0	EC	ATP8B1
0	0.57387585	0.428	0.091	0	EC	RNF213
0	0.57353392	0.424	0.097	0	EC	TRIOBP
0	0.57306164	0.379	0.05	0	EC	CTTNBP2NL
0	0.57260357	0.589	0.28	0	EC	SERPINB6
0	0.5714421	0.276	0.003	0	EC	TMEM273
0	0.57105658	0.742	0.469	0	EC	ARGLU1
0	0.57105631	0.299	0.024	0	EC	BTN3A2
0	0.57077762	0.529	0.189	0	EC	TMEM123
0	0.56884922	0.321	0.01	0	EC	MAGI1
0	0.56548741	0.257	0.001	0	EC	SOX17
0	0.56487911	0.317	0.033	0	EC	MDK
0	0.56439386	0.324	0.017	0	EC	ARHGEF3
0	0.56355896	0.478	0.176	0	EC	SWAP70
0	0.56072803	0.525	0.191	0	EC	AFDN
0	0.56009156	0.563	0.252	0	EC	SPCS3
0	0.55963382	0.352	0.031	0	EC	EPHB4
0	0.55904489	0.337	0.102	0	EC	CCND1
0	0.55858344	0.323	0.009	0	EC	RASGRP3
0	0.55770552	0.29	0.028	0	EC	MAP3K1
0	0.55579978	0.348	0.03	0	EC	PRKCH
0	0.55474606	0.751	0.492	0	EC	SERBP1
0	0.55335765	0.235	0.01	0	EC	ARL15
0	0.5532848	0.517	0.225	0	EC	ACTR3
0	0.5526062	0.263	0.01	0	EC	IGFBP2
0	0.55218829	0.492	0.202	0	EC	PRMT1
0	0.55197137	0.338	0.034	0	EC	TSPAN18
0	0.54911642	0.486	0.183	0	EC	PLSCR1
0	0.5477477	0.341	0.073	0	EC	SLC12A2
0	0.54636423	0.452	0.131	0	EC	TAOK1
0	0.54630561	0.293	0.006	0	EC	MCF2L
0	0.54571771	0.295	0.049	0	EC	LXN
0	0.5456965	0.285	0.005	0	EC	CCDC69
0	0.54380866	0.338	0.034	0	EC	SELENON
0	0.54156472	0.252	0.021	0	EC	LEPR
0	0.54138263	0.394	0.097	0	EC	BACE2
0	0.54113573	0.42	0.147	0	EC	PLK2
0	0.54078595	0.526	0.229	0	EC	BNIP2
0	0.54074158	0.498	0.195	0	EC	RBM17

0	0.5401999	0.43	0.102	0	EC	TMEM173
0	0.5395694	0.296	0.02	0	EC	FZD4
0	0.53955232	0.381	0.065	0	EC	RHOJ
0	0.53898524	0.588	0.3	0	EC	CAPZA2
0	0.53869858	0.351	0.03	0	EC	TMEM255B
0	0.5382449	0.277	0.001	0	EC	PDE2A
0	0.53796598	0.254	0.007	0	EC	PGM5
0	0.53667679	0.995	0.956	0	EC	RPS28
0	0.53569492	0.311	0.008	0	EC	PLEKHG1
0	0.53421621	0.55	0.254	0	EC	TNRC6B
0	0.53316723	0.537	0.214	0	EC	CTNNA1
0	0.53159167	0.459	0.169	0	EC	MTIF3
0	0.53112064	0.35	0.072	0	EC	FAM241A
0	0.53067878	0.454	0.157	0	EC	PEA15
0	0.53007908	0.228	0.006	0	EC	ADM5
0	0.5294045	0.345	0.044	0	EC	FNBP1L
0	0.52670496	0.587	0.289	0	EC	PAK2
0	0.52301467	0.252	0.013	0	EC	HLA-DQB1
0	0.52082025	0.485	0.179	0	EC	RAB5C
0	0.51620406	0.428	0.143	0	EC	NAA10
0	0.51492229	0.347	0.072	0	EC	MAST4
0	0.51097148	0.348	0.064	0	EC	SOCS2
0	0.50981343	0.301	0.044	0	EC	MYOF
0	0.5091939	0.226	0.023	0	EC	APOLD1
0	0.5070274	0.258	0.005	0	EC	SPNS2
0	0.50348398	0.442	0.172	0	EC	EFCAB14
0	0.50193614	0.279	0.02	0	EC	LRRC8C
0	0.50165057	0.302	0.017	0	EC	GRB10
0	0.50125198	0.349	0.08	0	EC	RASAL2
0	0.49985398	0.266	0.04	0	EC	PLCB4
0	0.49745459	0.392	0.079	0	EC	F2R
0	0.49707915	0.591	0.29	0	EC	AKAP13
0	0.49488509	0.398	0.103	0	EC	RPS6KA3
0	0.4946832	0.42	0.125	0	EC	ADAR
0	0.49427224	0.285	0.003	0	EC	SCARF1
0	0.49324719	0.526	0.249	0	EC	GNB2
0	0.49237409	0.431	0.14	0	EC	SETX
0	0.49196536	0.96	0.894	0	EC	MT-ND4
0	0.49085071	0.279	0.042	0	EC	WARS
0	0.48986787	0.317	0.028	0	EC	ARHGAP31
0	0.48914104	0.254	0.007	0	EC	TNFRSF1B
0	0.48878275	0.268	0.006	0	EC	TNFAIP8L1
0	0.48560361	0.466	0.178	0	EC	ACAP2
0	0.48501951	0.292	0.017	0	EC	JUP
0	0.48485482	0.378	0.106	0	EC	TAX1BP3
0	0.48418816	0.47	0.198	0	EC	CAPN2
0	0.48297827	0.3	0.02	0	EC	SHROOM4
0	0.48074611	0.213	0.002	0	EC	RASSF9
0	0.48046045	0.323	0.08	0	EC	SYNGR2
0	0.479905	0.38	0.117	0	EC	CNOT6L
0	0.47988775	0.315	0.027	0	EC	TSPAN9

0	0.47953122	0.211	0.002	0	EC	ACE
0	0.47897582	0.285	0.023	0	EC	TNFRSF14
0	0.47513403	0.211	0.009	0	EC	CNTNAP3B
0	0.47510768	0.265	0.001	0	EC	CPLX1
0	0.47474387	0.382	0.105	0	EC	YES1
0	0.47451489	0.305	0.023	0	EC	LIPA
0	0.47392828	0.27	0.02	0	EC	CASKIN2
0	0.47283971	0.218	0.009	0	EC	SH3BGRL2
0	0.47017121	0.411	0.13	0	EC	SNTB2
0	0.47007585	0.278	0.014	0	EC	TSPAN12
0	0.46952322	0.288	0.013	0	EC	DOCK6
0	0.46719117	0.264	0.002	0	EC	AFAP1L1
0	0.46694405	0.255	0.002	0	EC	NOS3
0	0.46674716	0.219	0.002	0	EC	JAG2
0	0.46616134	0.366	0.081	0	EC	LYPLA1
0	0.46602108	0.353	0.092	0	EC	CPD
0	0.46502104	0.39	0.135	0	EC	SEC14L1
0	0.46491832	0.326	0.089	0	EC	GBP2
0	0.46273551	0.251	0.003	0	EC	STAB1
0	0.46248133	0.421	0.13	0	EC	CRK
0	0.46196012	0.207	0.014	0	EC	TCN2
0	0.46067791	0.236	0.008	0	EC	C1orf115
0	0.4578562	0.457	0.171	0	EC	OGA
0	0.45758488	0.372	0.118	0	EC	PPP2R5A
0	0.45714343	0.45	0.182	0	EC	FRMD4B
0	0.45626004	0.305	0.066	0	EC	SP110
0	0.45496658	0.25	0.001	0	EC	HSPA12B
0	0.45462895	0.234	0.01	0	EC	APOL1
0	0.45393766	0.286	0.027	0	EC	FZD6
0	0.45312783	0.219	0.003	0	EC	INHBB
0	0.45054417	0.223	0.018	0	EC	TP53I11
0	0.44945591	0.391	0.119	0	EC	DICER1
0	0.4491454	0.293	0.027	0	EC	TNFAIP1
0	0.44859553	0.204	0.03	0	EC	MX1
0	0.44734035	0.216	0.006	0	EC	CARD16
0	0.44694947	0.366	0.121	0	EC	COL4A3BP
0	0.44631084	0.298	0.039	0	EC	STIM2
0	0.44469076	0.326	0.079	0	EC	TIA1
0	0.44379204	0.371	0.103	0	EC	NECTIN2
0	0.4423692	0.272	0.027	0	EC	RPS6KA2
0	0.44143794	0.325	0.07	0	EC	AIDA
0	0.43877616	0.248	0.019	0	EC	SAMHD1
0	0.43746502	0.408	0.143	0	EC	ATL3
0	0.43727105	0.368	0.095	0	EC	CDC42BPB
0	0.43708124	0.325	0.101	0	EC	ZNF521
0	0.43707354	0.293	0.026	0	EC	SIGIRR
0	0.43634416	0.33	0.08	0	EC	TCF12
0	0.43632886	0.235	0.009	0	EC	APOL3
0	0.43529523	0.255	0.025	0	EC	ERAP2
0	0.43526363	0.355	0.107	0	EC	SMAD2
0	0.43506124	0.412	0.15	0	EC	ADD1

0	0.43487565	0.235	0.002	0	EC	DLL4
0	0.43319303	0.316	0.061	0	EC	RALB
0	0.42702782	0.254	0.007	0	EC	SH2D3C
0	0.42661525	0.322	0.086	0	EC	ATXN3
0	0.42642468	0.279	0.017	0	EC	ELMO1
0	0.42607461	0.223	0.005	0	EC	RNF125
0	0.42587031	0.237	0.007	0	EC	IFI44
0	0.42558365	0.408	0.147	0	EC	CYB5A
0	0.42546272	0.276	0.047	0	EC	LY96
0	0.42533244	0.277	0.03	0	EC	RAPGEF1
0	0.42398402	0.391	0.129	0	EC	METTL9
0	0.42352972	0.336	0.099	0	EC	MIS18BP1
0	0.42346819	0.338	0.096	0	EC	PTPN14
0	0.42321975	0.202	0.021	0	EC	GBP1
0	0.42255388	0.309	0.067	0	EC	DGKH
0	0.42077793	0.235	0.012	0	EC	EBF3
0	0.41927068	0.26	0.002	0	EC	SEMA6B
0	0.41913462	0.302	0.052	0	EC	FYN
0	0.41883267	0.386	0.129	0	EC	PICALM
0	0.41791972	0.319	0.075	0	EC	RB1
0	0.41759131	0.329	0.089	0	EC	RALA
0	0.4164661	0.235	0.003	0	EC	PCDH12
0	0.41623688	0.216	0.001	0	EC	NPR1
0	0.41366737	0.423	0.158	0	EC	TNS2
0	0.41355497	0.237	0	0	EC	NOVA2
0	0.41288081	0.244	0.005	0	EC	ICA1
0	0.41242069	0.291	0.037	0	EC	DBN1
0	0.4112546	0.246	0.029	0	EC	STARD9
0	0.41046494	0.226	0.028	0	EC	IKBKB
0	0.41002539	0.226	0.011	0	EC	LPCAT2
0	0.40813284	0.303	0.061	0	EC	RIPOR1
0	0.40620354	0.279	0.08	0	EC	SOS1
0	0.40571437	0.232	0.007	0	EC	CADPS2
0	0.40458021	0.3	0.054	0	EC	ELOVL1
0	0.40334146	0.288	0.064	0	EC	CPNE8
0	0.40320078	0.318	0.074	0	EC	MPDZ
0	0.40250231	0.307	0.073	0	EC	SPTLC2
0	0.39928879	0.273	0.026	0	EC	FMNL3
0	0.39556473	0.325	0.089	0	EC	GNS
0	0.39492536	0.374	0.132	0	EC	HECTD1
0	0.3947628	0.288	0.016	0	EC	LAPTM5
0	0.39280744	0.212	0.01	0	EC	LAMA5
0	0.392265	0.248	0.028	0	EC	MYO5C
0	0.39183971	0.303	0.073	0	EC	LBR
0	0.39105306	0.367	0.124	0	EC	SHISA5
0	0.39020499	0.243	0.027	0	EC	TAOK2
0	0.39016899	0.356	0.101	0	EC	NCOA4
0	0.38947633	0.369	0.123	0	EC	SNAP23
0	0.38923043	0.273	0.04	0	EC	ARHGAP23
0	0.38686445	0.244	0.012	0	EC	LYL1
0	0.38583838	0.305	0.067	0	EC	GRAMD1A

0	0.3857712	0.301	0.065	0	EC	CYFIP1
0	0.38495276	0.292	0.075	0	EC	ATP11C
0	0.38355565	0.202	0.003	0	EC	F11R
0	0.38355495	0.24	0.006	0	EC	VASH1
0	0.38315325	0.276	0.054	0	EC	WWP1
0	0.38314096	0.326	0.085	0	EC	PRKAA1
0	0.382698	0.234	0.005	0	EC	ARAP3
0	0.38205706	0.261	0.041	0	EC	RBMS2
0	0.38181086	0.306	0.076	0	EC	APBB2
0	0.37934919	0.264	0.06	0	EC	VPS13A
0	0.37932112	0.277	0.072	0	EC	ZDHHC17
0	0.37922378	0.311	0.085	0	EC	CD2AP
0	0.37875446	0.275	0.075	0	EC	PHKB
0	0.37783834	0.28	0.07	0	EC	MGAT4A
0	0.37749798	0.201	0.014	0	EC	TRIM22
0	0.37736397	0.318	0.08	0	EC	PPP1R18
0	0.37556995	0.334	0.1	0	EC	NUMB
0	0.37555535	0.401	0.15	0	EC	PHF14
0	0.3749089	0.236	0.06	0	EC	C21orf91
0	0.3741807	0.317	0.079	0	EC	PTPRK
0	0.37202438	0.256	0.043	0	EC	HOXB4
0	0.37200448	0.31	0.078	0	EC	RIN2
0	0.37124666	0.26	0.058	0	EC	MVP
0	0.37124144	0.262	0.062	0	EC	TMEM245
0	0.36978347	0.317	0.1	0	EC	FEZ2
0	0.36799967	0.318	0.098	0	EC	KBTBD2
0	0.36766324	0.229	0.047	0	EC	GFOD1
0	0.36616424	0.331	0.095	0	EC	PON2
0	0.366164	0.23	0.005	0	EC	DYSF
0	0.3634186	0.336	0.104	0	EC	ERBIN
0	0.36276047	0.32	0.088	0	EC	CDK17
0	0.36263201	0.202	0.005	0	EC	PIK3C2B
0	0.36177026	0.22	0.01	0	EC	ADGRG1
0	0.36126258	0.219	0.03	0	EC	TAP1
0	0.36092061	0.206	0.035	0	EC	PARP9
0	0.35915778	0.28	0.076	0	EC	BCL3
0	0.3580188	0.23	0.024	0	EC	RASSF3
0	0.35726734	0.206	0.005	0	EC	RASGRF2
0	0.35715229	0.268	0.044	0	EC	GIT1
0	0.35611031	0.255	0.038	0	EC	KLHL5
0	0.35262186	0.274	0.068	0	EC	SYNJ2BP
0	0.35248926	0.986	0.927	0	EC	RPLP2
0	0.3522184	0.261	0.058	0	EC	DPP8
0	0.35216492	0.269	0.056	0	EC	GPRC5B
0	0.35196151	0.312	0.092	0	EC	MYO6
0	0.34901146	0.242	0.061	0	EC	MANSC1
0	0.34869854	0.992	0.956	0	EC	RPS24
0	0.34423474	0.226	0.051	0	EC	PIK3R3
0	0.34369104	0.201	0.042	0	EC	FAM111A
0	0.34368674	0.216	0.035	0	EC	TPST2
0	0.34341673	0.273	0.061	0	EC	RGL2

0	0.34263338	0.27	0.071	0	EC	DOCK1
0	0.34140937	0.263	0.063	0	EC	HMBOX1
0	0.3406416	0.302	0.092	0	EC	FAR1
0	0.34018429	0.304	0.084	0	EC	GSDMD
0	0.33969314	0.213	0.022	0	EC	KIAA1671
0	0.33937542	0.267	0.07	0	EC	EXOC5
0	0.33897556	0.232	0.043	0	EC	PML
0	0.33804595	0.226	0.016	0	EC	SH2B3
0	0.33782116	0.264	0.044	0	EC	CPNE2
0	0.33717691	0.212	0.032	0	EC	TLR4
0	0.33635899	0.255	0.046	0	EC	PKN1
0	0.33507543	0.253	0.051	0	EC	ABCD4
0	0.33410278	0.274	0.067	0	EC	WDFY1
0	0.33318306	0.286	0.07	0	EC	CHD7
0	0.33255425	0.26	0.037	0	EC	PARVB
0	0.33166425	0.207	0.003	0	EC	ABI3
0	0.32925172	0.274	0.076	0	EC	PCGF2
0	0.32851467	0.264	0.054	0	EC	MSL3
0	0.32326854	0.294	0.083	0	EC	FBXW5
0	0.32305447	0.208	0.016	0	EC	ITPKB
0	0.32110789	0.254	0.067	0	EC	GABPA
0	0.32056639	0.205	0.015	0	EC	BCR
0	0.31846492	0.214	0.015	0	EC	PCDH1
0	0.31793364	0.226	0.016	0	EC	IPO11
0	0.31605746	0.235	0.035	0	EC	MYO5A
0	0.31579811	0.237	0.058	0	EC	HERC2P2
0	0.31518978	0.277	0.074	0	EC	MAPK1
0	0.31479288	0.281	0.079	0	EC	SRGAP2
0	0.31410566	0.255	0.062	0	EC	TBC1D5
0	0.3128451	0.222	0.027	0	EC	ATP11A
0	0.31060907	0.283	0.077	0	EC	CEP170
0	0.30930734	0.241	0.056	0	EC	TRIO
0	0.30755347	0.227	0.051	0	EC	KITLG
0	0.30679669	0.243	0.06	0	EC	AP2A2
0	0.30419847	0.254	0.064	0	EC	DNMT1
0	0.30406242	0.273	0.075	0	EC	TANC1
0	0.30255459	0.208	0.028	0	EC	GFOD2
0	0.30118791	0.201	0.023	0	EC	PRKD2
0	0.29961868	0.221	0.039	0	EC	GIT2
0	0.29841137	0.219	0.04	0	EC	DGKZ
0	0.29815814	0.205	0.03	0	EC	NUDCD1
0	0.29694863	0.226	0.043	0	EC	SMAGP
0	0.29629306	0.253	0.062	0	EC	TLNRD1
0	0.29442445	0.208	0.044	0	EC	2-Mar
0	0.2941978	0.218	0.05	0	EC	CYTH1
0	0.29417381	0.218	0.037	0	EC	NOL4L
0	0.29055911	0.257	0.065	0	EC	KIF1C
0	0.29036963	0.21	0.048	0	EC	ITPR1
0	0.28864921	0.25	0.067	0	EC	KIFC3
0	0.28120091	0.239	0.045	0	EC	ACTR1A
0	0.27771523	0.21	0.044	0	EC	TMEM184B

0	0.27511426	0.201	0.04	0	EC	RIMKLB
0	0.27304419	0.236	0.05	0	EC	PPP1R13B
0	0.27249996	0.206	0.038	0	EC	TRAF7
0	0.27122504	0.216	0.052	0	EC	ZNF608
0	0.26861727	0.215	0.047	0	EC	MCM3
0	0.2670359	0.233	0.056	0	EC	LGALS8
0	0.2669206	0.21	0.04	0	EC	LYSMD2
0	0.26522451	0.21	0.046	0	EC	PCGF3
0	0.25936829	0.217	0.042	0	EC	LYN
0	0.25792778	0.216	0.052	0	EC	FAM160B1
0	0.25754391	0.226	0.05	0	EC	SINHCAF
1.11E-307	0.38925103	0.409	0.156	2.81E-303	EC	LARS
3.67E-307	0.41559992	0.408	0.158	9.26E-303	EC	RAB10
3.32E-306	0.30012516	0.223	0.056	8.39E-302	EC	HPCAL1
1.18E-305	0.4352997	0.438	0.175	2.97E-301	EC	MYCBP2
2.98E-305	0.46912385	0.493	0.218	7.53E-301	EC	ANKRD11
3.71E-303	0.32493126	0.316	0.1	9.36E-299	EC	ABL2
6.17E-302	0.30004176	0.299	0.091	1.56E-297	EC	NAV1
3.71E-301	0.25489919	0.248	0.067	9.36E-297	EC	GFM1
8.74E-300	0.46698819	0.482	0.211	2.21E-295	EC	SNX6
9.68E-300	0.42144562	0.451	0.185	2.44E-295	EC	PTBP3
4.05E-298	0.31635509	0.255	0.071	1.02E-293	EC	IGF1R
2.86E-297	0.31871247	0.307	0.098	7.21E-293	EC	CDC27
4.47E-297	0.35821582	0.352	0.122	1.13E-292	EC	BIRC6
5.71E-297	0.2958628	0.232	0.061	1.44E-292	EC	ORAI1
5.76E-297	0.4401818	0.934	0.804	1.45E-292	EC	RACK1
4.85E-296	0.55215863	0.939	0.802	1.22E-291	EC	ACTG1
1.05E-293	0.27479189	0.215	0.053	2.65E-289	EC	RNF38
1.09E-293	0.34115271	0.225	0.058	2.75E-289	EC	HOXB3
1.84E-293	0.2618135	0.235	0.062	4.65E-289	EC	NUP50
6.37E-293	0.5082069	0.526	0.249	1.61E-288	EC	NDRG1
8.84E-293	0.42973818	0.471	0.203	2.23E-288	EC	PDCD6IP
6.37E-291	0.32699401	0.247	0.069	1.61E-286	EC	ST6GAL1
7.85E-291	0.61608173	0.792	0.564	1.98E-286	EC	CD59
3.31E-290	0.32637699	0.357	0.125	8.35E-286	EC	HIP1
1.14E-289	0.45505037	0.488	0.217	2.87E-285	EC	HIPK3
3.00E-288	0.57738271	0.69	0.436	7.57E-284	EC	APLP2
6.78E-288	0.35575514	0.339	0.119	1.71E-283	EC	PPP1CA
2.91E-287	0.33915628	0.3	0.096	7.35E-283	EC	UHMK1
3.71E-286	0.54259687	0.58	0.317	9.37E-282	EC	NOP10
6.06E-285	0.44382703	0.509	0.235	1.53E-280	EC	RAD21
3.24E-284	0.38169582	0.307	0.099	8.18E-280	EC	KNOP1
4.99E-284	0.29743362	0.234	0.063	1.26E-279	EC	C19orf66
1.67E-282	0.27062332	0.237	0.065	4.22E-278	EC	ZNF281
1.02E-280	0.34347979	0.309	0.102	2.58E-276	EC	FCHO2
1.82E-280	0.26672552	0.234	0.064	4.59E-276	EC	DCAF7
2.95E-280	0.34233157	0.306	0.1	7.44E-276	EC	BAZ2A
8.74E-279	0.4029289	0.393	0.157	2.21E-274	EC	FDPS
6.66E-278	0.30355152	0.28	0.086	1.68E-273	EC	PTPRE
4.11E-274	0.32661173	0.316	0.107	1.04E-269	EC	POLR2J3.1
9.02E-274	0.44867149	0.559	0.289	2.28E-269	EC	SYNCRIP

4.20E-273	0.31911599	0.316	0.107	1.06E-268	EC	ANKRD17
2.84E-272	0.29890303	0.269	0.082	7.15E-268	EC	LRRC8A
5.24E-271	0.35823847	0.342	0.124	1.32E-266	EC	VAT1
3.00E-269	0.3730828	0.421	0.174	7.57E-265	EC	RAB6A
7.55E-269	0.28660104	0.226	0.062	1.90E-264	EC	HOXD8
1.39E-268	0.71114311	0.563	0.298	3.51E-264	EC	CD320
1.14E-267	0.4170072	0.41	0.168	2.89E-263	EC	10-Sep
2.05E-266	0.36244519	0.376	0.146	5.18E-262	EC	AKT3
1.24E-265	0.37716806	0.4	0.16	3.12E-261	EC	ODF2L
3.13E-265	0.39420739	0.357	0.135	7.91E-261	EC	CRBN
5.85E-265	0.45501069	0.634	0.361	1.48E-260	EC	QKI
2.97E-264	0.34397905	0.317	0.11	7.49E-260	EC	HOXB7
3.38E-264	0.26274428	0.248	0.073	8.53E-260	EC	CORO1B
8.10E-264	0.50514903	0.659	0.398	2.04E-259	EC	PTTG1IP
1.20E-263	0.39014284	0.397	0.161	3.02E-259	EC	FAM120A
1.81E-263	0.30423447	0.283	0.091	4.56E-259	EC	ACSL4
2.83E-263	0.39831621	0.449	0.198	7.14E-259	EC	MOB1A
2.67E-261	0.29685342	0.272	0.086	6.74E-257	EC	MOB2
4.67E-261	0.29926651	0.308	0.104	1.18E-256	EC	BRD9
5.64E-261	0.27803096	0.23	0.065	1.42E-256	EC	MAP3K3
8.80E-261	0.4244871	0.531	0.259	2.22E-256	EC	GRN
1.70E-260	0.34826339	0.376	0.147	4.30E-256	EC	CSNK2A1
1.83E-260	0.31700671	0.346	0.126	4.63E-256	EC	CCNL2
2.90E-260	0.36399023	0.325	0.116	7.31E-256	EC	SLC39A10
8.49E-259	0.35789546	0.396	0.163	2.14E-254	EC	LAMTOR2
4.86E-258	0.437881	0.452	0.205	1.23E-253	EC	CARHSP1
3.36E-257	0.35682299	0.423	0.178	8.47E-253	EC	SMARCA2
1.16E-256	0.32592894	0.99	0.945	2.93E-252	EC	RPL37
1.41E-256	0.34884247	0.379	0.149	3.57E-252	EC	TRIP12
2.71E-256	0.34489285	0.37	0.145	6.84E-252	EC	PAFAH1B2
5.99E-256	0.40480249	0.443	0.196	1.51E-251	EC	GSTK1
1.33E-255	0.30832623	0.286	0.095	3.36E-251	EC	UBN1
9.90E-255	0.26717643	0.258	0.079	2.50E-250	EC	KPNA3
2.18E-254	0.28618753	0.251	0.076	5.51E-250	EC	NFATC2IP
4.69E-254	0.30340125	0.264	0.084	1.18E-249	EC	CLCN3
8.36E-254	0.31739467	0.234	0.069	2.11E-249	EC	MINDY2
3.94E-253	0.26666384	0.221	0.062	9.94E-249	EC	UBP1
6.93E-253	0.43886353	0.52	0.266	1.75E-248	EC	PSMA4
1.62E-252	0.32604794	0.361	0.14	4.09E-248	EC	HDAC2
2.17E-252	0.40902233	0.423	0.183	5.48E-248	EC	PSME2
3.29E-252	0.3108936	0.279	0.091	8.30E-248	EC	RALGDS
5.30E-251	0.2754991	0.239	0.071	1.34E-246	EC	HERC1
6.85E-251	0.43345301	0.537	0.27	1.73E-246	EC	BPTF
1.44E-250	0.25100198	0.225	0.064	3.62E-246	EC	CDC42SE2
3.18E-250	0.32989552	0.321	0.116	8.01E-246	EC	KAT6A
5.93E-250	0.37617802	0.358	0.138	1.50E-245	EC	EIF2AK2
3.40E-248	0.59182672	0.635	0.377	8.59E-244	EC	SNHG7
4.21E-247	0.47168776	0.577	0.321	1.06E-242	EC	SH3GLB1
6.29E-247	0.34870596	0.31	0.111	1.59E-242	EC	TMPO
7.79E-246	0.49388338	0.721	0.487	1.96E-241	EC	POLR2L
3.59E-243	0.27053854	0.269	0.087	9.05E-239	EC	CDK11B

8.07E-243	0.26467818	0.272	0.088	2.04E-238	EC	UBA6
5.78E-242	0.26623547	0.256	0.08	1.46E-237	EC	UHRF2
6.98E-242	0.31877841	0.301	0.106	1.76E-237	EC	BTBD7
2.20E-241	0.27656388	0.234	0.07	5.56E-237	EC	STXBP1
3.27E-241	0.31618199	0.324	0.119	8.24E-237	EC	WDR82
6.37E-241	0.3437046	0.379	0.155	1.61E-236	EC	SVIL
1.34E-240	0.33449794	0.267	0.087	3.38E-236	EC	EHBP1L1
9.55E-240	0.31717897	0.328	0.122	2.41E-235	EC	SMARCE1
1.27E-238	0.33701333	0.34	0.13	3.20E-234	EC	IFNGR1
4.05E-237	0.2656595	0.251	0.079	1.02E-232	EC	GSK3B
4.87E-237	0.42192266	0.542	0.293	1.23E-232	EC	GDI2
1.87E-236	0.30058385	0.356	0.139	4.71E-232	EC	RERE
6.79E-235	0.36412464	0.398	0.174	1.71E-230	EC	ABHD17A
1.33E-234	0.27982434	0.286	0.099	3.35E-230	EC	PDS5A
5.04E-234	0.41831441	0.543	0.289	1.27E-229	EC	G3BP1
7.39E-234	0.31317561	0.33	0.124	1.87E-229	EC	TNPO1
3.47E-233	0.27141951	0.206	0.058	8.76E-229	EC	MARF1
6.23E-233	0.28232328	0.271	0.091	1.57E-228	EC	WASHC4
1.05E-232	0.45336657	0.473	0.241	2.66E-228	EC	AP2S1
1.13E-231	0.30427085	0.213	0.062	2.86E-227	EC	DGKE
1.86E-231	0.37515454	0.398	0.17	4.69E-227	EC	AP2B1
4.97E-231	0.32549304	0.35	0.138	1.25E-226	EC	RECQL
5.77E-230	0.3547763	0.327	0.125	1.46E-225	EC	KIAA1109
1.30E-229	0.44635224	0.542	0.295	3.29E-225	EC	C9orf16
1.59E-229	0.315807	0.337	0.13	4.00E-225	EC	RBM6
7.30E-229	0.40073709	0.523	0.266	1.84E-224	EC	UACA
9.29E-229	0.41311059	0.529	0.275	2.34E-224	EC	TPR
3.42E-228	0.2656314	0.249	0.079	8.64E-224	EC	FAM126A
3.94E-228	0.36841138	0.491	0.237	9.95E-224	EC	ARID1B
7.07E-228	0.28935861	0.29	0.102	1.79E-223	EC	PRKD3
9.71E-226	0.28096451	0.294	0.105	2.45E-221	EC	SH3BP2
1.86E-225	0.25971464	0.265	0.089	4.69E-221	EC	IARS2
2.65E-225	0.42648094	0.405	0.18	6.70E-221	EC	CEMIP2
8.68E-225	0.26555236	0.27	0.092	2.19E-220	EC	TSNAX
4.75E-224	0.33247744	0.396	0.171	1.20E-219	EC	H2AFY
3.69E-223	0.43829847	0.597	0.35	9.30E-219	EC	ARL2
1.31E-222	0.44605822	0.695	0.481	3.30E-218	EC	EIF4G2
2.75E-222	0.3052237	0.361	0.149	6.93E-218	EC	EIF4G1
5.96E-222	0.39487377	0.482	0.249	1.50E-217	EC	ARPC4
1.26E-221	0.33150341	0.374	0.157	3.19E-217	EC	ERICH1
2.14E-220	0.42964395	0.607	0.362	5.40E-216	EC	RAP1B
3.19E-220	0.37854214	0.498	0.258	8.04E-216	EC	NONO
8.54E-220	0.40324841	0.486	0.253	2.15E-215	EC	CCT6A
1.52E-219	0.29250389	0.305	0.114	3.84E-215	EC	EI24
3.17E-219	0.26893718	0.267	0.091	7.99E-215	EC	GON4L
6.48E-219	0.31273368	0.279	0.099	1.64E-214	EC	POLK
9.75E-218	0.40055869	0.593	0.325	2.46E-213	EC	NKTR
1.13E-217	0.29713092	0.335	0.132	2.85E-213	EC	SMG1
1.59E-217	0.39795122	0.396	0.174	4.01E-213	EC	SLC44A2
4.12E-217	0.25390295	0.283	0.101	1.04E-212	EC	SSRP1
7.80E-217	0.28031372	0.288	0.104	1.97E-212	EC	FAM208A

8.05E-217	0.32120766	0.363	0.15	2.03E-212	EC	SETD2
1.56E-216	0.25526407	0.288	0.104	3.93E-212	EC	EPN1
2.34E-216	0.33768278	0.343	0.14	5.91E-212	EC	MAPK3
2.66E-216	0.27334135	0.246	0.081	6.72E-212	EC	ZDHHC20
3.67E-216	0.37253908	0.501	0.259	9.25E-212	EC	SRSF4
6.37E-216	0.27528819	0.249	0.083	1.61E-211	EC	NOL8
6.66E-216	0.41887402	0.56	0.314	1.68E-211	EC	SYPL1
1.77E-215	0.3311306	0.343	0.138	4.47E-211	EC	ATF7IP
1.24E-214	0.36150193	0.471	0.235	3.12E-210	EC	XRN2
2.04E-214	0.2696938	0.274	0.097	5.15E-210	EC	STK25
9.44E-214	0.29757756	0.257	0.088	2.38E-209	EC	NUDT3
4.40E-213	0.35327397	0.372	0.159	1.11E-208	EC	AFF1
1.17E-212	0.28506192	0.309	0.119	2.94E-208	EC	PPP4C
3.25E-212	0.29788522	0.335	0.134	8.20E-208	EC	RAB1B
5.85E-212	0.27581068	0.321	0.123	1.48E-207	EC	CELF1
1.30E-211	0.2777614	0.275	0.098	3.27E-207	EC	CTCF
2.19E-211	0.31281882	0.373	0.158	5.52E-207	EC	THOC2
1.88E-210	0.26006244	0.22	0.069	4.73E-206	EC	WDR11
4.82E-210	0.28974589	0.337	0.135	1.22E-205	EC	FOXO1
7.80E-209	0.29168315	0.984	0.929	1.97E-204	EC	RPS15
1.23E-208	0.40683557	0.731	0.51	3.11E-204	EC	PRRC2C
2.93E-208	0.62590657	0.351	0.153	7.40E-204	EC	IFI6
1.30E-205	0.36209392	0.403	0.187	3.27E-201	EC	TAF15
3.59E-205	0.289123	0.337	0.138	9.05E-201	EC	ACIN1
3.69E-205	0.33393924	0.396	0.176	9.31E-201	EC	PPP2R2A
8.34E-205	0.5487148	0.452	0.231	2.10E-200	EC	TGFBR3
2.28E-203	0.39601761	0.37	0.162	5.75E-199	EC	ITPR2
2.88E-203	0.2663025	0.248	0.085	7.27E-199	EC	INPP1
6.43E-203	0.41736491	0.539	0.297	1.62E-198	EC	CD46
1.25E-202	0.41438681	0.576	0.346	3.16E-198	EC	CBX3
9.83E-202	0.30508477	0.385	0.171	2.48E-197	EC	RIF1
1.42E-201	0.420749	0.506	0.266	3.59E-197	EC	PCMTD1
6.14E-201	0.42319219	0.568	0.339	1.55E-196	EC	AES
6.40E-201	0.38896682	0.449	0.223	1.61E-196	EC	APPL1
9.41E-201	0.28164566	0.279	0.104	2.37E-196	EC	GPBP1L1
2.57E-200	0.34978753	0.481	0.247	6.48E-196	EC	NCKAP1
4.38E-200	0.35490074	0.437	0.214	1.10E-195	EC	DNAJC15
4.77E-199	0.33232759	0.325	0.133	1.20E-194	EC	UBTF
5.42E-199	0.35428584	0.458	0.229	1.37E-194	EC	ENSA
1.56E-198	0.32118563	0.343	0.148	3.93E-194	EC	LAPTM4B
3.21E-198	0.32317267	0.454	0.219	8.10E-194	EC	UPF2
9.10E-198	0.28982357	0.359	0.155	2.30E-193	EC	PPP2R1A
2.39E-197	0.26173155	0.288	0.108	6.03E-193	EC	LTBR
1.75E-196	0.25843907	0.275	0.101	4.41E-192	EC	EXOC1
2.49E-196	0.26935586	0.268	0.098	6.28E-192	EC	LTN1
3.40E-196	0.29482124	0.316	0.127	8.57E-192	EC	MLLT6
5.20E-196	0.37514062	0.442	0.22	1.31E-191	EC	RAP1A
6.37E-195	0.37220095	0.456	0.231	1.61E-190	EC	BCAP29
8.41E-195	0.32372119	0.382	0.172	2.12E-190	EC	XPO1
1.16E-194	0.25650135	0.266	0.097	2.93E-190	EC	GGCT
1.73E-194	0.33555151	0.306	0.123	4.37E-190	EC	OCIAD2

6.10E-193	0.28210277	0.293	0.113	1.54E-188	EC	ERC1
2.54E-192	0.31174841	0.363	0.159	6.41E-188	EC	CDC42BPA
2.56E-192	0.29262551	0.299	0.118	6.46E-188	EC	YPEL2
7.49E-191	0.3971138	0.573	0.341	1.89E-186	EC	ZNHIT1
1.21E-190	0.36262907	0.472	0.239	3.05E-186	EC	SPTAN1
1.53E-189	0.29176087	0.287	0.112	3.86E-185	EC	NBR1
1.91E-188	0.33808566	0.39	0.179	4.82E-184	EC	PHACTR4
2.48E-188	0.27852055	0.337	0.144	6.27E-184	EC	SSNA1
1.54E-187	0.2672445	0.282	0.108	3.88E-183	EC	EFHD2
2.93E-187	0.28963942	0.306	0.123	7.40E-183	EC	ZMYND11
5.65E-187	0.35777172	0.471	0.247	1.43E-182	EC	SCP2
1.99E-186	0.35795521	0.26	0.098	5.03E-182	EC	STAT1
3.21E-186	0.29038649	0.306	0.124	8.10E-182	EC	CCNY
6.45E-186	0.25516313	0.235	0.082	1.63E-181	EC	STAT2
8.61E-186	0.2733118	0.226	0.078	2.17E-181	EC	HOMER3
2.07E-185	0.31496441	0.375	0.171	5.23E-181	EC	SMCHD1
7.69E-183	0.3474486	0.418	0.206	1.94E-178	EC	PRKDC
2.03E-182	0.31582304	0.354	0.157	5.13E-178	EC	UBE2G2
5.24E-181	0.2839797	0.281	0.11	1.32E-176	EC	TTC37
1.43E-180	0.3230536	0.403	0.196	3.61E-176	EC	VAMP3
5.93E-180	0.3593461	0.337	0.149	1.50E-175	EC	MDFIC
1.05E-178	0.29431577	0.309	0.129	2.66E-174	EC	ZNF280D
3.73E-178	0.34408625	0.425	0.211	9.42E-174	EC	NFE2L1
1.28E-176	0.2922926	0.383	0.182	3.22E-172	EC	TIMM13
1.30E-176	0.31043847	0.423	0.209	3.27E-172	EC	NIPBL
3.50E-176	0.34767609	0.444	0.225	8.82E-172	EC	SMAD5
4.28E-176	0.28311151	0.315	0.133	1.08E-171	EC	SUZ12
3.90E-175	0.37505116	0.516	0.292	9.84E-171	EC	DDX46
6.52E-175	0.36057074	0.496	0.278	1.64E-170	EC	RAB14
6.89E-175	0.34902677	0.766	0.584	1.74E-170	EC	HNRNPA3
1.02E-173	0.26954437	0.382	0.18	2.57E-169	EC	TRIM44
1.58E-173	0.32327127	0.476	0.25	4.00E-169	EC	CSGALNACT1
1.78E-173	0.29720571	0.326	0.142	4.48E-169	EC	REST
2.36E-172	0.35305709	0.52	0.297	5.95E-168	EC	DAZAP2
2.88E-172	1.22358151	0.412	0.226	7.26E-168	EC	H19
3.29E-172	0.315106	0.444	0.23	8.30E-168	EC	CLTC
4.73E-172	0.41738192	0.663	0.484	1.19E-167	EC	ARPC2
5.20E-171	0.30865841	0.366	0.17	1.31E-166	EC	KMT2C
1.20E-170	0.25436987	0.253	0.096	3.04E-166	EC	USP33
3.77E-170	0.35357541	0.404	0.202	9.50E-166	EC	HSD17B12
1.08E-169	0.3114305	0.403	0.201	2.72E-165	EC	ANP32A
1.17E-169	0.25882538	0.98	0.927	2.96E-165	EC	RPL36
2.49E-169	0.28511394	0.293	0.122	6.27E-165	EC	STX12
5.75E-169	0.28965211	0.343	0.154	1.45E-164	EC	PITPNB
1.24E-168	0.28195442	0.277	0.112	3.13E-164	EC	RBL2
6.16E-168	0.25166532	0.258	0.1	1.56E-163	EC	APC
5.11E-167	0.29489607	0.433	0.217	1.29E-162	EC	NRP2
7.31E-165	0.26231327	0.262	0.103	1.85E-160	EC	RICTOR
7.58E-165	0.29508592	0.355	0.166	1.91E-160	EC	POLR2G
1.34E-164	0.28421049	0.337	0.151	3.37E-160	EC	AAK1
1.40E-164	0.41547385	0.695	0.517	3.54E-160	EC	HSPA8

1.43E-164	0.27844416	0.334	0.15	3.60E-160	EC	TTC1
5.64E-164	0.36770906	0.392	0.189	1.42E-159	EC	CTSC
5.78E-164	0.27423817	0.291	0.121	1.46E-159	EC	CCAR1
1.78E-162	0.30977571	0.356	0.167	4.49E-158	EC	ZNF148
5.89E-162	0.26985473	0.352	0.164	1.49E-157	EC	MBD2
3.21E-161	0.34777775	0.852	0.714	8.10E-157	EC	H3F3A
3.10E-160	0.28074163	0.357	0.167	7.82E-156	EC	RBM26
3.17E-160	0.33894167	0.38	0.186	7.99E-156	EC	ZMIZ1
9.40E-160	0.26397596	0.327	0.147	2.37E-155	EC	BAX
9.42E-160	0.34730058	0.856	0.705	2.38E-155	EC	ANXA2
3.00E-159	0.27324229	0.384	0.187	7.57E-155	EC	GNAI3
5.27E-159	0.36346326	0.511	0.305	1.33E-154	EC	BCAP31
8.25E-159	0.27633674	0.265	0.108	2.08E-154	EC	STX7
2.84E-158	0.31510276	0.366	0.177	7.16E-154	EC	FKBP9
3.56E-158	0.2657401	0.345	0.16	8.98E-154	EC	PHF20L1
1.18E-157	0.28029338	0.33	0.15	2.98E-153	EC	FNDC3A
1.08E-156	0.26690392	0.397	0.198	2.73E-152	EC	PLEKHA1
3.43E-156	0.33822305	0.541	0.32	8.67E-152	EC	SERINC3
3.79E-156	0.35056029	0.449	0.244	9.57E-152	EC	TAPBP
2.76E-155	0.28289067	0.365	0.176	6.98E-151	EC	BRD7
4.74E-155	0.28665118	0.379	0.188	1.19E-150	EC	CAPRIN1
3.74E-154	0.34071136	0.431	0.23	9.45E-150	EC	KLF3
9.03E-154	0.56479088	0.362	0.182	2.28E-149	EC	DEPP1
2.00E-153	0.27865813	0.33	0.153	5.04E-149	EC	TIMM8B
4.53E-153	0.33634795	0.731	0.541	1.14E-148	EC	PCBP2
1.57E-152	0.25657597	0.983	0.934	3.96E-148	EC	RPS15A
6.68E-152	0.29100227	0.356	0.171	1.68E-147	EC	PDCD10
4.06E-151	0.35360955	0.442	0.247	1.02E-146	EC	MRPL52
1.85E-150	0.27244927	0.334	0.155	4.67E-146	EC	RALY
1.95E-149	0.3271762	0.438	0.239	4.93E-145	EC	TROVE2
4.22E-149	0.26029244	0.379	0.187	1.06E-144	EC	PUM1
8.48E-149	0.27056423	0.283	0.123	2.14E-144	EC	NSD1
3.54E-148	0.34562517	0.5	0.303	8.92E-144	EC	NDUFS6
3.93E-148	0.26893764	0.349	0.167	9.91E-144	EC	NEMF
4.25E-148	0.25536984	0.331	0.154	1.07E-143	EC	DNAJC10
1.47E-147	0.27160707	0.303	0.136	3.71E-143	EC	CYB5B
1.81E-147	0.263719	0.293	0.129	4.56E-143	EC	RBM22
4.42E-147	0.30569954	0.409	0.22	1.12E-142	EC	CCT2
7.58E-147	0.29652031	0.378	0.191	1.91E-142	EC	SMC3
8.99E-147	0.25562508	0.267	0.113	2.27E-142	EC	MAT2B
2.79E-146	0.28441246	0.324	0.151	7.03E-142	EC	NCK1
3.21E-146	0.26991847	0.406	0.21	8.09E-142	EC	ZNF638
7.04E-146	0.27977022	0.463	0.258	1.78E-141	EC	NAMPT
1.28E-145	0.30997724	0.46	0.261	3.24E-141	EC	SELENOF
1.50E-145	0.25945726	0.311	0.142	3.79E-141	EC	FAM210B
4.03E-145	0.26445761	0.311	0.142	1.02E-140	EC	XRN1
2.84E-144	0.32580079	0.453	0.26	7.17E-140	EC	ARHGDIA
7.02E-144	0.26599458	0.414	0.22	1.77E-139	EC	CAPZA1
1.60E-143	0.25437494	0.327	0.152	4.04E-139	EC	CCDC25
1.87E-143	0.27086686	0.287	0.127	4.72E-139	EC	CD40
5.92E-143	0.25608985	0.306	0.139	1.49E-138	EC	GOLGA7

6.72E-143	0.26088499	0.401	0.208	1.69E-138	EC	IPO7
7.92E-143	0.27364903	0.366	0.182	2.00E-138	EC	SHOC2
1.79E-142	0.30550226	0.346	0.169	4.51E-138	EC	CDC42EP3
1.88E-142	0.28319618	0.348	0.171	4.74E-138	EC	NUMA1
3.28E-141	0.29056943	0.416	0.221	8.27E-137	EC	UPF3A
2.72E-140	0.27935139	0.757	0.581	6.87E-136	EC	HNRNPU
9.06E-140	0.30344776	0.446	0.253	2.29E-135	EC	NDUFB3
4.16E-139	0.3512137	0.373	0.2	1.05E-134	EC	UBE2J1
4.42E-139	0.37025762	0.655	0.491	1.12E-134	EC	ARPC3
1.01E-137	0.33802236	0.624	0.434	2.55E-133	EC	EIF2S2
2.42E-137	0.25521182	0.319	0.15	6.12E-133	EC	PLA2G16
3.32E-137	0.30566969	0.7	0.481	8.39E-133	EC	KLF6
5.59E-137	0.28105884	0.309	0.145	1.41E-132	EC	PAXX
1.33E-136	0.26000131	0.606	0.389	3.35E-132	EC	STAT3
3.27E-136	0.30631569	0.401	0.215	8.25E-132	EC	TMEM109
3.83E-135	0.33314331	0.485	0.296	9.67E-131	EC	COX5A
4.34E-134	0.38626889	0.633	0.464	1.10E-129	EC	GPX1
1.32E-133	0.29780378	0.388	0.207	3.34E-129	EC	TBC1D15
1.05E-132	0.32481004	0.625	0.442	2.66E-128	EC	HNRNPD
1.26E-132	0.33014814	0.501	0.315	3.17E-128	EC	GADD45GIP1
1.69E-131	0.25550958	0.314	0.149	4.28E-127	EC	XIAP
2.93E-130	0.32229329	0.596	0.396	7.39E-126	EC	SCAF11
4.54E-130	0.27958427	0.365	0.19	1.15E-125	EC	VPS35
7.73E-130	0.29043963	0.348	0.179	1.95E-125	EC	NDUFA8
1.07E-129	0.34243408	0.651	0.462	2.71E-125	EC	CANX
4.66E-129	0.30853838	0.613	0.414	1.18E-124	EC	EIF5B
1.22E-128	0.30307009	0.396	0.221	3.08E-124	EC	TXNDC17
1.27E-128	0.29954282	0.404	0.22	3.20E-124	EC	CGGBP1
5.46E-128	0.28483038	0.768	0.601	1.38E-123	EC	HNRNPK
7.21E-127	0.33140143	0.709	0.54	1.82E-122	EC	ATP5PF
1.83E-126	0.48118242	0.62	0.482	4.63E-122	EC	POMP
5.52E-126	0.29873151	0.55	0.354	1.39E-121	EC	DDX21
1.51E-125	0.2999311	0.454	0.27	3.81E-121	EC	UBE2L3
3.70E-125	0.30678302	0.467	0.273	9.33E-121	EC	SUN1
7.32E-123	0.25601537	0.325	0.161	1.85E-118	EC	FAM89B
1.22E-122	0.25314256	0.302	0.145	3.09E-118	EC	MSL1
1.87E-121	0.30291602	0.813	0.668	4.71E-117	EC	RPL36A
7.37E-120	0.26866676	0.412	0.231	1.86E-115	EC	MED13L
9.69E-120	0.26841268	0.447	0.262	2.45E-115	EC	PRPF40A
3.26E-119	0.26314056	0.366	0.194	8.23E-115	EC	TUG1
6.10E-118	0.26270875	0.472	0.285	1.54E-113	EC	NDUFA3
5.76E-117	0.2712559	0.39	0.218	1.45E-112	EC	PPP1CC
5.53E-116	0.25348007	0.349	0.187	1.40E-111	EC	PHB
3.16E-115	0.28563139	0.326	0.172	7.98E-111	EC	MRPL17
3.76E-115	0.26786508	0.629	0.432	9.50E-111	EC	SF3B1
6.38E-115	0.51403742	0.306	0.158	1.61E-110	EC	PLPP1
8.48E-115	0.29387645	0.495	0.313	2.14E-110	EC	CHMP2A
5.48E-114	0.26948833	0.363	0.198	1.38E-109	EC	CTSZ
2.70E-112	0.26971612	0.42	0.252	6.81E-108	EC	CCT3
4.17E-111	0.28229244	0.569	0.387	1.05E-106	EC	KHDRBS1
1.94E-110	0.26528996	0.343	0.181	4.90E-106	EC	PHIP

1.05E-109	0.26083927	0.382	0.214	2.65E-105	EC	SMIM37
9.47E-108	0.29065665	0.379	0.211	2.39E-103	EC	DAAM1
1.95E-107	0.26976006	0.584	0.409	4.91E-103	EC	RBX1
2.41E-106	0.35666301	0.585	0.428	6.08E-102	EC	NAA38
4.78E-104	0.25675748	0.433	0.258	1.21E-99	EC	SLTM
4.26E-103	0.28195053	0.457	0.295	1.07E-98	EC	APRT
1.09E-102	0.27536475	0.628	0.456	2.74E-98	EC	UBXN4
8.24E-102	0.26148938	0.369	0.208	2.08E-97	EC	RFC1
2.16E-101	0.25504207	0.368	0.211	5.44E-97	EC	TUBB6
4.06E-101	0.25005886	0.345	0.187	1.03E-96	EC	ESF1
1.60E-99	0.25907988	0.411	0.25	4.05E-95	EC	VPS29
2.61E-98	0.26572071	0.522	0.339	6.59E-94	EC	DDX18
3.89E-98	0.254123	0.403	0.246	9.81E-94	EC	CCT8
6.12E-98	0.25673797	0.537	0.359	1.54E-93	EC	TM9SF3
2.09E-97	0.33976989	0.493	0.339	5.28E-93	EC	ATP1B3
9.78E-97	0.33765989	0.334	0.188	2.47E-92	EC	NET1
1.35E-95	0.26202223	0.238	0.115	3.42E-91	EC	TRIM56
1.89E-95	0.27318046	0.515	0.359	4.76E-91	EC	MTPN
9.67E-94	0.28054019	0.471	0.3	2.44E-89	EC	NSRP1
1.04E-93	0.26165901	0.38	0.224	2.63E-89	EC	POLE4
8.09E-93	0.27614266	0.789	0.661	2.04E-88	EC	COX4I1
7.51E-89	0.25716783	0.442	0.277	1.90E-84	EC	ELF1
8.79E-88	0.26288083	0.514	0.365	2.22E-83	EC	SNRPE
1.17E-85	0.26915312	0.546	0.395	2.96E-81	EC	ATOX1
7.74E-85	0.25210938	0.577	0.404	1.95E-80	EC	EMP2
1.41E-82	0.25114008	0.593	0.424	3.56E-78	EC	TRAM1
1.54E-73	0.30241854	0.233	0.124	3.88E-69	EC	CRTAC1
3.97E-73	0.26347831	0.494	0.35	1.00E-68	EC	PIM3
1.66E-62	0.29483148	0.484	0.344	4.18E-58	EC	LY6E
0	4.39985924	0.736	0.031	0	Blood	LYZ
0	3.9312757	0.657	0.029	0	Blood	S100A9
0	3.80780323	0.619	0.021	0	Blood	S100A8
0	2.92091558	0.434	0.014	0	Blood	CXCL8
0	2.63834352	0.768	0.196	0	Blood	SRGN
0	2.31445917	0.441	0.009	0	Blood	AC020656.1
0	2.28109799	0.272	0.007	0	Blood	MPO
0	2.12814476	0.271	0.004	0	Blood	CCL3
0	1.85605887	0.396	0.004	0	Blood	TYROBP
0	1.82866758	0.219	0.007	0	Blood	AZU1
0	1.80849757	0.281	0.003	0	Blood	CCL3L1
0	1.80124964	0.387	0.004	0	Blood	FCER1G
0	1.4553119	0.217	0.028	0	Blood	CXCL2
0	1.42900014	0.327	0.003	0	Blood	AIF1
0	1.371126	0.34	0.023	0	Blood	CTSS
0	1.36311722	0.378	0.003	0	Blood	LCP1
0	1.25962149	0.287	0.007	0	Blood	CXCR4
0	1.23348875	0.333	0.012	0	Blood	HCST
0	1.23092149	0.218	0.002	0	Blood	FCN1
0	1.22014473	0.318	0.002	0	Blood	PTPRC
0	1.21724296	0.27	0.002	0	Blood	ALOX5AP
0	1.20546536	0.303	0.005	0	Blood	CD37

0	1.17098036	0.287	0.002	0	Blood	LST1
0	1.1086977	0.242	0.002	0	Blood	PLEK
0	1.0955896	0.252	0.003	0	Blood	CD52
0	1.08437595	0.305	0.004	0	Blood	CORO1A
0	0.95983646	0.285	0.001	0	Blood	CD48
0	0.90175096	0.241	0.003	0	Blood	ITGB2
0	0.79371342	0.242	0.001	0	Blood	CD53
0	0.78366795	0.209	0.025	0	Blood	PHACTR1
0	0.76792689	0.211	0.003	0	Blood	EVI2B
1.18E-291	1.55525251	0.64	0.332	2.97E-287	Blood	CYBA
5.13E-285	1.10069376	0.269	0.051	1.29E-280	Blood	TNFAIP3
2.37E-265	0.80517357	0.955	0.927	5.98E-261	Blood	RPS29
2.14E-231	0.57798106	0.967	0.951	5.39E-227	Blood	RPL28
5.69E-219	1.078835	0.32	0.089	1.44E-214	Blood	COTL1
7.41E-204	1.41337305	0.215	0.045	1.87E-199	Blood	GPR183
2.22E-163	0.38611539	0.994	0.99	5.61E-159	Blood	RPLP1
7.82E-152	0.67348275	0.248	0.07	1.97E-147	Blood	STK17B
1.34E-138	0.45237601	0.983	0.976	3.39E-134	Blood	RPS27
2.38E-133	0.74184022	0.269	0.087	6.00E-129	Blood	RASGEF1B
3.43E-103	0.56798803	0.241	0.084	8.66E-99	Blood	FAM49B
5.81E-94	0.665283	0.215	0.074	1.47E-89	Blood	PYCARD
1.72E-92	0.35319398	0.98	0.975	4.35E-88	Blood	RPS18
7.15E-83	0.39746438	0.934	0.946	1.81E-78	Blood	RPS19
3.35E-81	0.41663902	0.851	0.872	8.44E-77	Blood	RPS21
1.14E-80	0.35666648	0.911	0.914	2.89E-76	Blood	RPS7
2.64E-72	0.30938031	0.975	0.97	6.65E-68	Blood	RPL32
5.84E-72	0.51757226	0.21	0.082	1.47E-67	Blood	GLIPR1
6.55E-70	0.30779166	0.952	0.953	1.65E-65	Blood	RPS14
2.43E-67	0.28854342	0.935	0.948	6.13E-63	Blood	RPL26
1.39E-61	0.32157048	0.862	0.891	3.50E-57	Blood	UBA52
1.77E-61	1.02664114	0.375	0.247	4.46E-57	Blood	TKT
2.44E-45	0.50315119	0.213	0.104	6.15E-41	Blood	RHOG
1.48E-43	0.8918661	0.371	0.266	3.73E-39	Blood	SERPINB1
1.15E-38	0.50356021	0.206	0.105	2.90E-34	Blood	GPCPD1
1.84E-38	0.62728587	0.215	0.111	4.63E-34	Blood	DUSP2
4.72E-38	0.46812733	0.203	0.103	1.19E-33	Blood	NFKB1
1.54E-34	0.27787274	0.82	0.871	3.90E-30	Blood	RPL22
4.07E-34	0.63223169	0.22	0.125	1.03E-29	Blood	PPIF
1.75E-32	0.26177293	0.342	0.597	4.42E-28	Blood	CSTB
8.35E-26	0.51825437	0.208	0.124	2.11E-21	Blood	RAB11FIP1
1.71E-22	0.54136725	0.296	0.21	4.31E-18	Blood	KDM6B
3.02E-20	0.50643393	0.562	0.635	7.62E-16	Blood	ATP5MG
9.68E-19	0.41537449	0.202	0.13	2.44E-14	Blood	STK4
3.49E-17	0.4076884	0.236	0.166	8.82E-13	Blood	GRB2
5.04E-16	0.6391567	0.255	0.197	1.27E-11	Blood	CAPG
5.34E-15	0.42995886	0.208	0.144	1.35E-10	Blood	REL
5.58E-12	0.80002455	0.297	0.253	1.41E-07	Blood	SGK1
5.08E-10	0.38805707	0.216	0.168	1.28E-05	Blood	MAP2K3
5.31E-10	0.42765531	0.252	0.206	1.34E-05	Blood	MAP3K2
1.55E-09	0.53340738	0.279	0.246	3.92E-05	Blood	RILPL2
6.89E-09	0.5106201	0.204	0.162	0.0001739	Blood	ATP2B1-AS1

8.96E-09	0.47267504	0.474	0.52	0.0002262	Blood	SNHG5
2.00E-08	0.40017912	0.49	0.504	0.0005057	Blood	SOD2
4.59E-07	0.49778715	0.392	0.411	0.0115943	Blood	HMGN1
