# Supplementary material for: Spatially defined single-cell transcriptional profiling characterizes diverse chondrocyte subtypes and nucleus pulposus progenitors in human intervertebral discs
Source: Bone Res. 2021 Aug 16;9:37. doi: 10.1038/s41413-021-00163-z (PMC8368097; doi:10.1038/s41413-021-00163-z)
Supplement: Supplementary file 4 — Supplementary Table 3 [file 41413_2021_163_MOESM4_ESM.pdf]

Supplementary Table 3.<br>DEGs among the 6 chondrocyte subclusters							
p_val	avg_logFC	pct.1	pct.2	p_val_adj	cluster	gene	is.TF
0	2.385437553	0.762	0.16	0	C1	IBSP	FALSE
0	2.367277083	0.975	0.421	0	C1	CYTL1	FALSE
0	2.068852476	0.41	0.067	0	C1	SPP1	FALSE
0	1.571884401	0.401	0.026	0	C1	COL10A1	FALSE
0	1.529548188	0.69	0.075	0	C1	IL17B	FALSE
0	1.498271281	1	0.954	0	C1	C2orf40	FALSE
0	1.468010097	0.984	0.645	0	C1	APOD	FALSE
0	1.44649722	0.986	0.735	0	C1	RBP4	FALSE
0	1.4289038	0.974	0.619	0	C1	CHAD	FALSE
0	1.303300194	0.949	0.748	0	C1	CRYAB	FALSE
0	1.225038696	0.983	0.616	0	C1	SOD3	FALSE
0	1.112935926	0.529	0.201	0	C1	HMOX1	FALSE
0	1.095461972	0.995	0.883	0	C1	FGFBP2	FALSE
0	1.092199186	0.929	0.647	0	C1	HSPA1A	FALSE
0	0.981979725	0.525	0.134	0	C1	HSPA6	FALSE
0	0.95299221	0.883	0.547	0	C1	HSPA1B	FALSE
0	0.926547113	0.46	0.193	0	C1	DDIT4	FALSE
0	0.915316134	0.814	0.381	0	C1	PDPN	FALSE
0	0.87085415	0.737	0.292	0	C1	PHLDA2	TRUE
0	0.798153593	0.67	0.289	0	C1	TM4SF1	FALSE
0	0.721017311	1	0.993	0	C1	MGP	FALSE
0	0.711894843	0.512	0.173	0	C1	NMB	FALSE
0	0.710520677	0.662	0.33	0	C1	FGF2	FALSE
0	0.689211412	0.644	0.304	0	C1	LARP6	FALSE
0	0.687296482	0.895	0.597	0	C1	MAP1LC3	FALSE
0	0.681340261	0.976	0.78	0	C1	MIA	FALSE
0	0.675813645	0.488	0.126	0	C1	BMP2	FALSE
0	0.671019427	0.701	0.393	0	C1	SLC3A2	FALSE
0	0.641184225	0.999	0.945	0	C1	S100A6	FALSE
0	0.620083582	0.954	0.73	0	C1	CALM1	FALSE
0	0.612835493	0.842	0.551	0	C1	DNAJA1	FALSE
0	0.609710549	0.689	0.436	0	C1	PMP22	FALSE
0	0.607456673	0.92	0.693	0	C1	S100A11	FALSE
0	0.600091394	0.474	0.197	0	C1	IGFBP6	FALSE
0	0.594087498	0.751	0.424	0	C1	SPINT2	FALSE
0	0.593821493	0.829	0.532	0	C1	BZW1	FALSE
0	0.59237006	0.651	0.357	0	C1	ROCR	FALSE
0	0.586657379	0.938	0.74	0	C1	LGALS3	FALSE
0	0.579508818	0.754	0.578	0	C1	KCNMA1	FALSE
0	0.572008737	0.672	0.338	0	C1	BTG3	FALSE
0	0.571902324	0.983	0.894	0	C1	HSP90AB1	FALSE
0	0.567372979	0.739	0.393	0	C1	GEM	FALSE
0	0.562113451	0.851	0.577	0	C1	SELENOK	FALSE
0	0.553291435	0.883	0.633	0	C1	EMP3	FALSE
0	0.551648165	0.742	0.435	0	C1	GNG5	FALSE
0	0.548076065	0.913	0.672	0	C1	SAT1	FALSE
0	0.52630503	0.727	0.422	0	C1	WTAP	FALSE
0	0.517110201	0.541	0.235	0	C1	CRNDE	FALSE
0	0.51471451	0.68	0.347	0	C1	CD55	FALSE
0	0.496519956	0.956	0.757	0	C1	S100A13	FALSE
0	0.495956389	0.95	0.745	0	C1	CALM2	FALSE
0	0.494432001	0.397	0.135	0	C1	ZFAND2A	FALSE
0	0.489171762	0.392	0.119	0	C1	HMGA1	TRUE
0	0.47647229	0.779	0.534	0	C1	RAN	TRUE

0	0.473658741	0.391	0.134	0	C1	TSPAN13	FALSE
0	0.471783105	0.83	0.615	0	C1	SQSTM1	FALSE
0	0.463129019	0.754	0.475	0	C1	TUBB2A	FALSE
0	0.459691907	0.801	0.585	0	C1	TXN	FALSE
0	0.457348843	0.691	0.433	0	C1	IER3	FALSE
0	0.455948244	0.596	0.298	0	C1	CNN3	FALSE
0	0.455325493	0.583	0.305	0	C1	TOP1	FALSE
0	0.454227774	0.721	0.462	0	C1	RBM8A	TRUE
0	0.452223368	0.373	0.141	0	C1	GGTA1P	FALSE
0	0.451030359	0.213	0.027	0	C1	ELF3	TRUE
0	0.450863106	0.37	0.11	0	C1	HOTAIRM	FALSE
0	0.448953058	0.871	0.751	0	C1	C11orf96	FALSE
0	0.448941229	0.628	0.351	0	C1	PNRC2	FALSE
0	0.445431925	0.379	0.129	0	C1	UBE2D1	FALSE
0	0.443325245	0.707	0.421	0	C1	FGFR1	FALSE
0	0.443019411	0.664	0.385	0	C1	DNAJB6	FALSE
0	0.442908816	0.375	0.123	0	C1	KRT18	FALSE
0	0.439871564	0.411	0.143	0	C1	N4BP2L1	FALSE
0	0.439565968	0.732	0.431	0	C1	TIMP3	FALSE
0	0.438903591	0.955	0.801	0	C1	MT-RNR1	FALSE
0	0.437658721	0.858	0.605	0	C1	SEC61G	FALSE
0	0.43190635	0.488	0.219	0	C1	PCSK1N	FALSE
0	0.431719661	0.626	0.355	0	C1	CAMTA1	TRUE
0	0.430844726	0.79	0.546	0	C1	H2AFJ	FALSE
0	0.427941624	0.374	0.146	0	C1	PLAUR	FALSE
0	0.427287512	0.37	0.123	0	C1	CAMK2N1	FALSE
0	0.425119549	0.855	0.603	0	C1	NDUFA4	FALSE
0	0.424826065	0.976	0.919	0	C1	UBC	FALSE
0	0.422758082	0.749	0.476	0	C1	SEM1	FALSE
0	0.421505118	0.457	0.222	0	C1	MLF1	FALSE
0	0.416437663	0.655	0.4	0	C1	RPL22L1	FALSE
0	0.415264263	0.424	0.232	0	C1	IRF1	TRUE
0	0.413874051	0.675	0.423	0	C1	DANCR	FALSE
0	0.41269042	0.66	0.393	0	C1	COX7B	FALSE
0	0.409274646	0.484	0.234	0	C1	UGDH	FALSE
0	0.402212709	0.381	0.141	0	C1	AKIRIN2	FALSE
0	0.402058777	0.835	0.598	0	C1	SBDS	FALSE
0	0.39646116	0.83	0.592	0	C1	PTMS	FALSE
0	0.39308897	0.944	0.754	0	C1	HMGB1	TRUE
0	0.391806533	0.331	0.098	0	C1	RAB31	FALSE
0	0.389858784	0.23	0.043	0	C1	CHAC1	FALSE
0	0.386681199	0.671	0.419	0	C1	POLR2K	FALSE
0	0.384988211	0.859	0.643	0	C1	SEC61B	FALSE
0	0.381205682	0.881	0.649	0	C1	CD9	FALSE
0	0.380545147	0.811	0.572	0	C1	GPX4	FALSE
0	0.379589897	0.552	0.324	0	C1	SDCBP	FALSE
0	0.378973587	0.525	0.294	0	C1	DNAJB9	FALSE
0	0.37823491	0.966	0.844	0	C1	RGCC	FALSE
0	0.374578298	0.548	0.323	0	C1	CYSTM1	FALSE
0	0.373551357	0.99	0.945	0	C1	MT-RNR2	FALSE
0	0.370969923	0.33	0.13	0	C1	PLIN2	FALSE
0	0.369849529	0.614	0.332	0	C1	SNHG9	FALSE
0	0.369297132	0.4	0.169	0	C1	UPP1	FALSE
0	0.366813119	0.873	0.672	0	C1	ANXA5	FALSE
0	0.364357856	0.492	0.262	0	C1	CHMP4B	FALSE
0	0.364135084	0.423	0.192	0	C1	SERTAD2	FALSE
0	0.363789978	0.226	0.077	0	C1	CD83	FALSE
0	0.36323348	0.824	0.594	0	C1	LGALS1	FALSE

0	0.36305403	0.52	0.289	0	C1	ILF2	TRUE
0	0.362881047	0.988	0.93	0	C1	SNORC	FALSE
0	0.358636246	0.298	0.119	0	C1	TIFA	FALSE
0	0.358246042	0.889	0.667	0	C1	SH3BGRL3	FALSE
0	0.354126464	0.295	0.091	0	C1	RAMP1	FALSE
0	0.354099517	0.302	0.087	0	C1	SPHK1	FALSE
0	0.353564981	0.672	0.378	0	C1	F13A1	FALSE
0	0.352752765	0.227	0.095	0	C1	CLIC3	FALSE
0	0.349610384	0.538	0.303	0	C1	EIF3J	FALSE
0	0.348480869	0.536	0.316	0	C1	BUD31	FALSE
0	0.347594446	0.462	0.215	0	C1	MIR22HG	FALSE
0	0.34402161	0.282	0.08	0	C1	PTTG1	FALSE
0	0.343675894	0.698	0.465	0	C1	RSRC2	FALSE
0	0.343321931	0.399	0.179	0	C1	ATF7IP2	FALSE
0	0.34153455	0.898	0.691	0	C1	TSPO	FALSE
0	0.341043859	0.484	0.291	0	C1	CITED4	FALSE
0	0.340252551	0.547	0.316	0	C1	LMO4	FALSE
0	0.337130328	0.48	0.225	0	C1	PNP	FALSE
0	0.335593383	0.368	0.172	0	C1	BEX5	FALSE
0	0.335375429	0.21	0.025	0	C1	IRX3	TRUE
0	0.335186193	0.241	0.05	0	C1	ARHGDIB	FALSE
0	0.334699447	0.207	0.058	0	C1	TACSTD2	FALSE
0	0.334433708	0.532	0.302	0	C1	EIF5A	FALSE
0	0.332635766	0.55	0.335	0	C1	EZR	TRUE
0	0.332147327	0.785	0.568	0	C1	SUB1	FALSE
0	0.332140159	0.567	0.35	0	C1	LITAF	FALSE
0	0.329443934	0.232	0.089	0	C1	PDE4B	FALSE
0	0.329373065	0.375	0.172	0	C1	ARPC5L	FALSE
0	0.328769028	0.911	0.737	0	C1	BTG1	FALSE
0	0.327528491	0.332	0.114	0	C1	NUPR2	FALSE
0	0.326067617	0.301	0.107	0	C1	C1QTNF4	FALSE
0	0.325921132	0.532	0.344	0	C1	GSTO1	FALSE
0	0.325127924	0.922	0.742	0	C1	GSTP1	FALSE
0	0.324238114	0.455	0.266	0	C1	ARID5B	TRUE
0	0.321998833	0.783	0.576	0	C1	LINC0157	FALSE
0	0.320829282	0.921	0.718	0	C1	CCNI	FALSE
0	0.319387748	0.659	0.448	0	C1	TUBA1C	FALSE
0	0.319339689	0.565	0.351	0	C1	HNRNPAB	FALSE
0	0.319012104	0.364	0.17	0	C1	HIGD1A	FALSE
0	0.316417685	0.205	0.068	0	C1	S100A2	FALSE
0	0.316120754	0.389	0.201	0	C1	RHOD	FALSE
0	0.314362277	0.87	0.672	0	C1	YBX1	TRUE
0	0.314172517	0.382	0.197	0	C1	TCEAL6	TRUE
0	0.313726493	0.297	0.098	0	C1	CLCF1	FALSE
0	0.31186629	0.372	0.175	0	C1	SMIM4	FALSE
0	0.311667466	0.308	0.172	0	C1	RRAD	FALSE
0	0.310154745	0.987	0.875	0	C1	SERF2	FALSE
0	0.308889301	0.467	0.263	0	C1	NDUFA6	FALSE
0	0.308409028	0.357	0.156	0	C1	NKX3-1	TRUE
0	0.306586394	0.557	0.357	0	C1	SNRPB	FALSE
0	0.306006192	0.588	0.373	0	C1	SNRPG	FALSE
0	0.301283832	0.386	0.188	0	C1	TGFB1	FALSE
0	0.300090773	0.776	0.565	0	C1	YWHAZ	TRUE
0	0.299639168	0.468	0.266	0	C1	BIRC2	FALSE
0	0.299620516	0.897	0.717	0	C1	PRDX1	FALSE
0	0.29864845	0.651	0.439	0	C1	POLR2L	FALSE
0	0.298181571	0.44	0.249	0	C1	KIAA0040	FALSE
0	0.297937139	0.907	0.723	0	C1	CHCHD2	FALSE

0	0.290381025	0.33	0.157	0	C1	SRGN	FALSE
0	0.289370267	0.657	0.455	0	C1	CYB5R3	FALSE
0	0.288659322	0.979	0.86	0	C1	S100A1	FALSE
0	0.288043158	0.287	0.112	0	C1	CDKN2D	FALSE
0	0.287939739	0.267	0.087	0	C1	PRRX2	TRUE
0	0.287630535	0.802	0.58	0	C1	UBL5	FALSE
0	0.287146444	0.894	0.705	0	C1	MYL6	FALSE
0	0.286432537	0.888	0.685	0	C1	OST4	FALSE
0	0.284244587	0.713	0.499	0	C1	ARF4	FALSE
0	0.284135582	0.827	0.608	0	C1	YWHAE	TRUE
0	0.283609684	0.339	0.147	0	C1	CMC2	FALSE
0	0.283164562	0.342	0.155	0	C1	CDC37L1	FALSE
0	0.283089928	0.595	0.379	0	C1	ROMO1	FALSE
0	0.282192695	0.445	0.26	0	C1	ENHO	FALSE
0	0.280802449	0.529	0.341	0	C1	UBE2D2	FALSE
0	0.280431449	0.365	0.176	0	C1	FOXC2	TRUE
0	0.280381639	0.608	0.375	0	C1	MAFF	TRUE
0	0.277093764	0.878	0.666	0	C1	TMA7	FALSE
0	0.275835787	0.699	0.504	0	C1	SERP1	FALSE
0	0.274237948	0.731	0.517	0	C1	COX6A1	FALSE
0	0.274118988	0.428	0.231	0	C1	YWHAG	FALSE
0	0.274110454	0.733	0.524	0	C1	NDUFA1	FALSE
0	0.273367615	0.214	0.063	0	C1	MISP3	FALSE
0	0.272150766	0.267	0.111	0	C1	PHLDA1	FALSE
0	0.269646676	0.84	0.712	0	C1	CLEC3A	FALSE
0	0.269603119	0.359	0.147	0	C1	MXD1	TRUE
0	0.268100363	0.431	0.258	0	C1	C9orf16	FALSE
0	0.267696732	0.358	0.187	0	C1	TPBG	FALSE
0	0.265439696	0.638	0.446	0	C1	MYL12A	FALSE
0	0.262045221	0.264	0.13	0	C1	SPRY2	FALSE
0	0.260300365	0.35	0.195	0	C1	ANKRD37	FALSE
0	0.256810444	0.665	0.477	0	C1	TPM2	FALSE
0	0.255947631	0.365	0.183	0	C1	KRAS	FALSE
0	0.255632428	0.367	0.196	0	C1	UBE2A	FALSE
0	0.255508792	0.641	0.453	0	C1	UBE2B	FALSE
0	0.253112122	0.283	0.136	0	C1	LOX	FALSE
0	0.252606026	0.897	0.721	0	C1	SOD1	TRUE
0	0.251866267	0.204	0.064	0	C1	FBXO32	FALSE
0	0.251477314	0.246	0.099	0	C1	NTAN1	FALSE
0	1.422229383	0.966	0.521	0	C2	FRZB	FALSE
0	1.285579247	0.994	0.892	0	C2	CST3	FALSE
0	1.11858528	0.442	0.05	0	C2	AZGP1	FALSE
0	1.009942174	0.698	0.193	0	C2	STMN1	FALSE
0	0.976976605	0.614	0.288	0	C2	FABP5	FALSE
0	0.939465133	0.508	0.084	0	C2	RARRES2	FALSE
0	0.919007817	0.955	0.693	0	C2	DYNLL1	FALSE
0	0.839376522	0.956	0.666	0	C2	H2AFZ	TRUE
0	0.744423726	0.915	0.582	0	C2	ADIRF	FALSE
0	0.708365354	0.73	0.369	0	C2	UBE2S	FALSE
0	0.69841051	0.937	0.712	0	C2	HSPB1	FALSE
0	0.695704878	0.594	0.232	0	C2	MARCKSL	FALSE
0	0.6847188	0.303	0.108	0	C2	CSRP2	FALSE
0	0.667375165	0.855	0.565	0	C2	HSPE1	FALSE
0	0.658768634	0.207	0.013	0	C2	NPY	FALSE
0	0.656941569	0.979	0.755	0	C2	SLPI	FALSE
0	0.645616964	0.851	0.545	0	C2	CDKN1A	FALSE
0	0.640681755	0.876	0.619	0	C2	DNAJB1	FALSE
0	0.640174847	0.991	0.87	0	C2	S100A10	FALSE

0	0.626030031	0.419	0.122	0	C2	WFDC2	FALSE
0	0.61252305	0.55	0.231	0	C2	CDK2AP2	FALSE
0	0.611686564	0.78	0.475	0	C2	DDIT3	TRUE
0	0.598403219	0.987	0.887	0	C2	HSP90AA1	FALSE
0	0.590405105	0.994	0.878	0	C2	SERPINA1	FALSE
0	0.58769714	0.944	0.695	0	C2	SNHG8	FALSE
0	0.585432266	0.513	0.234	0	C2	SERPINI1	FALSE
0	0.584599658	0.966	0.865	0	C2	GADD45B	FALSE
0	0.574484361	0.751	0.426	0	C2	AL118516	FALSE
0	0.573785996	0.809	0.491	0	C2	CYCS	TRUE
0	0.566864511	0.784	0.45	0	C2	ODC1	TRUE
0	0.565013655	0.964	0.742	0	C2	ZFAS1	FALSE
0	0.554000617	0.272	0.106	0	C2	SFN	FALSE
0	0.549112874	0.429	0.179	0	C2	ISG20	FALSE
0	0.54067204	0.363	0.067	0	C2	MATN3	FALSE
0	0.532073351	0.339	0.092	0	C2	AC007906	FALSE
0	0.531455263	0.849	0.572	0	C2	EIF1B	FALSE
0	0.528353238	0.689	0.408	0	C2	HMGB2	TRUE
0	0.510003962	0.904	0.628	0	C2	PPP1R15A	FALSE
0	0.506614845	0.536	0.274	0	C2	SNHG12	FALSE
0	0.503243662	0.606	0.324	0	C2	BEX2	FALSE
0	0.491377558	0.596	0.327	0	C2	YWHAH	FALSE
0	0.490143383	0.264	0.06	0	C2	TACC3	FALSE
0	0.475741826	0.909	0.657	0	C2	SNHG6	FALSE
0	0.466713341	0.391	0.143	0	C2	CKS2	FALSE
0	0.457004929	0.956	0.764	0	C2	UBB	TRUE
0	0.45592624	0.763	0.509	0	C2	EPB41L4A	FALSE
0	0.453669899	0.642	0.393	0	C2	TUBB2B	FALSE
0	0.4481144	0.831	0.595	0	C2	CFL1	FALSE
0	0.447070025	0.782	0.542	0	C2	IER5	FALSE
0	0.443978	0.999	0.947	0	C2	RPS13	FALSE
0	0.438500647	0.287	0.087	0	C2	LCN2	FALSE
0	0.437335242	0.999	0.95	0	C2	RPL24	FALSE
0	0.434965279	0.81	0.56	0	C2	RPL9P9	FALSE
0	0.433161668	0.992	0.862	0	C2	RPS26	FALSE
0	0.430857167	0.989	0.89	0	C2	RPL23	FALSE
0	0.429886991	0.999	0.931	0	C2	RPS25	FALSE
0	0.424371495	1	0.97	0	C2	RPL30	FALSE
0	0.42082816	0.417	0.182	0	C2	FAM107B	FALSE
0	0.420456728	0.465	0.244	0	C2	KIF9	FALSE
0	0.420210298	0.512	0.241	0	C2	FTH1P10	FALSE
0	0.419752834	0.993	0.874	0	C2	RPL38	FALSE
0	0.418202554	0.658	0.409	0	C2	CDV3	FALSE
0	0.416606061	0.299	0.096	0	C2	AC015912	FALSE
0	0.413872522	0.816	0.59	0	C2	HMGN2	FALSE
0	0.412232394	0.474	0.244	0	C2	SNHG19	FALSE
0	0.410305899	0.856	0.605	0	C2	LMNA	FALSE
0	0.407794322	0.706	0.477	0	C2	TCEAL9	FALSE
0	0.407537037	0.501	0.282	0	C2	BCAS2	FALSE
0	0.406761879	0.585	0.351	0	C2	DCXR	FALSE
0	0.405326799	0.395	0.196	0	C2	DDIT4L	FALSE
0	0.40284518	0.999	0.964	0	C2	EIF1	FALSE
0	0.397573307	0.77	0.519	0	C2	TNFRSF12	FALSE
0	0.393819632	1	0.979	0	C2	RPS12	FALSE
0	0.393718354	0.478	0.257	0	C2	CDKN1C	FALSE
0	0.391517825	0.676	0.439	0	C2	ARL6IP1	FALSE
0	0.390905317	0.744	0.369	0	C2	HSPH1	FALSE
0	0.39066423	1	0.947	0	C2	RPL9	FALSE

0	0.387019259	0.518	0.294	0	C2	GTF2B	TRUE
0	0.384075382	0.921	0.704	0	C2	TUBB4B	FALSE
0	0.380446768	0.992	0.864	0	C2	RPS21	FALSE
0	0.380075933	1	0.966	0	C2	RPL32	FALSE
0	0.377658209	0.993	0.881	0	C2	RPL35	TRUE
0	0.375998275	0.667	0.441	0	C2	CCDC59	FALSE
0	0.374204133	0.971	0.816	0	C2	PNRC1	FALSE
0	0.370957553	0.288	0.117	0	C2	ZNF487	TRUE
0	0.370910743	0.432	0.241	0	C2	HIST1H4C	FALSE
0	0.370315498	0.998	0.942	0	C2	RPL18	FALSE
0	0.370298319	0.485	0.272	0	C2	DPH3	FALSE
0	0.36885497	0.576	0.376	0	C2	SNHG7	FALSE
0	0.367362932	1	0.978	0	C2	RPS27A	FALSE
0	0.366199788	0.998	0.922	0	C2	RPS15	FALSE
0	0.365864397	0.301	0.118	0	C2	ARG2	TRUE
0	0.359684025	0.998	0.92	0	C2	RPL36	FALSE
0	0.359630475	0.474	0.264	0	C2	EIF4A3	FALSE
0	0.358771846	0.998	0.954	0	C2	FTH1	FALSE
0	0.358598191	0.41	0.203	0	C2	KRT8	FALSE
0	0.358577982	1	0.971	0	C2	RPL34	FALSE
0	0.355994679	0.638	0.415	0	C2	SNRPF	FALSE
0	0.355729818	0.853	0.665	0	C2	NUPR1	FALSE
0	0.35426587	1	0.973	0	C2	RPS18	FALSE
0	0.35417453	0.888	0.707	0	C2	TSC22D1	TRUE
0	0.351872937	0.934	0.738	0	C2	RPL36AL	FALSE
0	0.349103951	0.999	0.963	0	C2	RPL11	FALSE
0	0.344994107	0.997	0.95	0	C2	H3F3B	FALSE
0	0.344578897	0.998	0.948	0	C2	RPL37A	FALSE
0	0.344281366	0.491	0.274	0	C2	NEU1	FALSE
0	0.34377111	0.469	0.278	0	C2	EFNA1	FALSE
0	0.341026977	0.999	0.954	0	C2	PTMA	FALSE
0	0.340739214	1	0.937	0	C2	RPL37	FALSE
0	0.339374388	0.999	0.943	0	C2	RPL26	FALSE
0	0.337242371	0.956	0.768	0	C2	BTF3	FALSE
0	0.332975946	0.998	0.927	0	C2	RPL35A	FALSE
0	0.332861198	0.982	0.828	0	C2	TOMM7	FALSE
0	0.332733091	0.362	0.176	0	C2	TPD52L1	FALSE
0	0.332332249	0.677	0.46	0	C2	TUBA1A	FALSE
0	0.329902138	1	0.972	0	C2	RPS27	FALSE
0	0.32982843	0.911	0.702	0	C2	GAS5	FALSE
0	0.329779942	0.553	0.351	0	C2	NLRP1	FALSE
0	0.327864481	1	0.985	0	C2	RPL41	FALSE
0	0.323580796	0.877	0.688	0	C2	EIF5	FALSE
0	0.322921319	0.584	0.378	0	C2	OTUD6B-A	FALSE
0	0.322780051	0.999	0.948	0	C2	RPS14	FALSE
0	0.320370965	1	0.969	0	C2	RPS23	FALSE
0	0.319683626	0.982	0.859	0	C2	RPL27A	FALSE
0	0.318715026	0.685	0.488	0	C2	RHEB	FALSE
0	0.31774183	0.794	0.583	0	C2	RSL24D1	FALSE
0	0.316842808	0.989	0.877	0	C2	RPS5	FALSE
0	0.316126953	0.932	0.735	0	C2	C12orf57	FALSE
0	0.316041519	0.994	0.907	0	C2	RPS7	FALSE
0	0.315614622	0.999	0.925	0	C2	RPL39	FALSE
0	0.314061254	1	0.989	0	C2	RPLP1	FALSE
0	0.310032326	0.776	0.584	0	C2	PTGES3	FALSE
0	0.308512625	0.554	0.357	0	C2	CCT4	FALSE
0	0.307752424	0.961	0.808	0	C2	S100B	FALSE
0	0.307359089	0.998	0.917	0	C2	FAU	FALSE

0	0.303664176	0.994	0.912	0	C2	NACA	FALSE
0	0.302887932	0.999	0.946	0	C2	RPL28	FALSE
0	0.302169498	0.291	0.113	0	C2	CPVL	FALSE
0	0.301278373	1	0.951	0	C2	RPS28	FALSE
0	0.295895773	0.997	0.92	0	C2	RPLP2	FALSE
0	0.295537522	0.98	0.844	0	C2	RPS10	TRUE
0	0.295496053	0.239	0.08	0	C2	ALDH1A2	FALSE
0	0.295024553	0.997	0.945	0	C2	RPL8	FALSE
0	0.293510326	0.998	0.934	0	C2	RPL18A	FALSE
0	0.286745291	0.982	0.855	0	C2	EEF1B2	FALSE
0	0.284360924	0.999	0.952	0	C2	RPL21	FALSE
0	0.281732084	0.931	0.741	0	C2	COMMD6	FALSE
0	0.280330658	1	0.964	0	C2	RPS3A	FALSE
0	0.280083154	0.278	0.123	0	C2	TGFBR3L	FALSE
0	0.280047335	0.991	0.889	0	C2	RPL10A	FALSE
0	0.279232984	0.863	0.654	0	C2	RPL36A	FALSE
0	0.278089089	1	0.981	0	C2	RPL13	FALSE
0	0.2753803	0.996	0.917	0	C2	RPL14	FALSE
0	0.269950372	0.962	0.797	0	C2	HNRNPA1	TRUE
0	0.267159435	0.997	0.928	0	C2	RPS15A	FALSE
0	0.260329696	0.997	0.94	0	C2	RPS19	FALSE
0	0.25478903	0.998	0.934	0	C2	RPL12	FALSE
#######	0.35695297	0.828	0.655	2.35E-303	C2	TMSB10	FALSE
#######	0.28981717	0.848	0.652	1.27E-301	C2	SAP18	FALSE
#######	0.32175028	0.518	0.327	1.80E-300	C2	DMKN	FALSE
#######	0.350393933	0.311	0.15	4.38E-295	C2	SOCS1	FALSE
#######	0.323075029	0.618	0.426	3.77E-293	C2	DDT	FALSE
#######	0.311068348	0.527	0.334	1.02E-290	C2	SVIP	FALSE
#######	0.343999308	0.324	0.161	2.23E-290	C2	OTOS	FALSE
#######	0.291431261	0.608	0.423	7.03E-282	C2	ELOC	FALSE
#######	0.25603964	0.961	0.785	1.08E-274	C2	LRRC75A-	FALSE
#######	0.343777231	0.719	0.514	6.16E-271	C2	SERTAD1	FALSE
#######	0.319679499	0.595	0.421	2.14E-268	C2	NUDC	FALSE
#######	0.259699726	0.755	0.561	1.39E-265	C2	COX8A	FALSE
#######	0.264523767	0.757	0.562	1.58E-263	C2	TUBA1B	FALSE
#######	0.297120207	0.729	0.539	5.02E-263	C2	DBI	FALSE
#######	0.293940154	0.398	0.228	1.48E-254	C2	H2AFX	FALSE
#######	0.293197245	0.314	0.165	3.54E-249	C2	AC084033	FALSE
#######	0.275274692	0.534	0.357	2.25E-248	C2	TPM3	FALSE
#######	0.290848649	0.48	0.313	1.03E-245	C2	PITHD1	FALSE
#######	0.342944181	0.49	0.325	7.67E-243	C2	CFAP20	FALSE
#######	0.258230538	0.575	0.402	1.37E-238	C2	MPC2	FALSE
#######	0.260004571	0.363	0.204	1.09E-236	C2	SLC25A33	FALSE
#######	0.258013861	0.263	0.127	1.97E-233	C2	AC087239	FALSE
#######	0.269304756	0.61	0.441	5.46E-229	C2	RHOC	FALSE
#######	0.328298045	0.423	0.259	1.35E-227	C2	AC044849	FALSE
#######	0.266472331	0.411	0.254	1.74E-223	C2	AHSA1	FALSE
#######	0.262811623	0.375	0.222	7.45E-219	C2	SNHG1	FALSE
#######	0.290778571	0.552	0.39	4.34E-215	C2	NDRG2	FALSE
#######	0.274920201	0.375	0.226	4.28E-207	C2	MED19	FALSE
#######	0.398818184	0.252	0.125	5.00E-204	C2	G0S2	FALSE
#######	0.250181895	0.336	0.199	1.30E-194	C2	TAF9	TRUE
#######	0.252835882	0.806	0.619	6.44E-187	C2	H1FX	TRUE
#######	0.28002944	0.374	0.239	4.90E-185	C2	CACYBP	FALSE
#######	0.250292236	0.386	0.245	2.82E-183	C2	ASXL1	FALSE
#######	0.267071096	0.606	0.456	6.30E-182	C2	GPX1	FALSE
#######	0.2593044	0.362	0.238	3.02E-158	C2	MRPL18	FALSE
#######	0.526288051	0.65	0.526	9.24E-152	C2	NFKBIA	FALSE

#######	0.255878066	0.385	0.258	3.39E-151	C2	SNHG3	FALSE
#######	0.639640671	0.417	0.29	4.76E-145	C2	CFH	FALSE
0	0.692962104	0.74	0.825	0	C3	MT-ND3	FALSE
0	0.641611209	0.806	0.881	0	C3	MT-CO2	FALSE
0	0.63079497	0.801	0.878	0	C3	MT-CO3	FALSE
0	0.626817535	0.842	0.909	0	C3	MT-ATP6	FALSE
0	0.621549801	0.772	0.745	0	C3	COL2A1	FALSE
0	0.57834616	0.708	0.857	0	C3	MT-ND2	FALSE
0	0.57534413	0.786	0.878	0	C3	MT-CYB	FALSE
0	0.569636992	0.829	0.914	0	C3	MT-ND4	FALSE
0	0.567692963	0.661	0.824	0	C3	MT-ND1	FALSE
0	0.563380364	0.971	0.978	0	C3	ACAN	FALSE
0	0.55357109	0.7	0.844	0	C3	MT-ND5	FALSE
0	0.498733916	0.909	0.94	0	C3	COMP	FALSE
0	0.48045856	0.793	0.882	0	C3	MT-CO1	FALSE
0	0.453983541	0.718	0.801	0	C3	CTGF	FALSE
0	0.450953384	0.738	0.857	0	C3	FMOD	FALSE
0	0.441851421	0.784	0.787	0	C3	EGR1	TRUE
0	0.282328019	0.173	0.353	0	C3	LRRC75A	FALSE
0	0.281825063	0.257	0.504	0	C3	TMSB4X	FALSE
#######	0.621749041	0.577	0.675	1.21E-290	C3	COL11A1	FALSE
#######	0.297277961	0.275	0.518	1.84E-246	C3	MELTF	FALSE
#######	0.272765374	0.265	0.491	6.28E-232	C3	PPP3CA	FALSE
#######	0.286823432	0.297	0.545	4.99E-221	C3	SLC38A2	FALSE
#######	0.295812956	0.205	0.381	6.12E-220	C3	MTATP6P1	FALSE
#######	0.260525525	0.312	0.564	1.26E-213	C3	PAPSS2	FALSE
#######	0.26636998	0.291	0.529	3.01E-213	C3	IVNS1ABP	FALSE
#######	0.281725586	0.171	0.323	1.14E-210	C3	LTBP3	FALSE
#######	0.28803389	0.236	0.431	1.76E-208	C3	SLC39A14	FALSE
#######	0.272922587	0.21	0.382	1.72E-194	C3	GLG1	FALSE
#######	0.28266247	0.215	0.385	4.20E-179	C3	CD109	FALSE
#######	0.250302623	0.166	0.3	7.39E-174	C3	GPC6	FALSE
#######	0.280222849	0.206	0.363	2.96E-170	C3	KLF9	TRUE
#######	0.281555442	0.157	0.282	3.59E-170	C3	C1orf56	FALSE
#######	0.295089658	0.24	0.42	6.30E-170	C3	SYNE1	FALSE
#######	0.285896053	0.277	0.482	9.65E-159	C3	GOLIM4	FALSE
#######	0.292988384	0.182	0.32	7.63E-157	C3	SOX5	TRUE
#######	0.263035748	0.378	0.664	5.81E-147	C3	SFPQ	TRUE
#######	0.299979503	0.226	0.387	1.06E-142	C3	DDR2	FALSE
#######	0.31320315	0.25	0.426	4.73E-135	C3	MRC2	FALSE
#######	0.381187195	0.684	0.824	1.27E-131	C3	COL9A3	FALSE
#######	0.271241984	0.344	0.582	1.17E-125	C3	GOLGB1	FALSE
#######	0.339703421	0.253	0.428	5.33E-125	C3	LINC-PINT	FALSE
#######	0.302848851	0.367	0.619	4.04E-120	C3	CTNNB1	TRUE
#######	0.343984278	0.217	0.362	2.60E-117	C3	ZNF207	TRUE
#######	0.261259307	0.123	0.21	5.52E-112	C3	AC027290	FALSE
#######	0.32125697	0.234	0.388	2.19E-111	C3	XYLT1	FALSE
#######	0.296373637	0.133	0.226	6.10E-109	C3	CSPG4	FALSE
#######	0.357588234	0.253	0.422	1.79E-108	C3	ITGA10	FALSE
#######	0.279788697	0.327	0.524	2.79E-108	C3	KLF6	TRUE
#######	0.260916846	0.13	0.218	4.73E-101	C3	EMILIN1	FALSE
9.32E-99	0.419056582	0.565	0.686	2.35E-94	C3	AEBP1	TRUE
9.54E-98	0.25872306	0.396	0.654	2.41E-93	C3	HIF1A	TRUE
6.02E-94	0.347444233	0.271	0.433	1.52E-89	C3	SLC26A2	FALSE
2.63E-87	0.335126763	0.257	0.408	6.65E-83	C3	TCF4	TRUE
9.54E-86	0.303199065	0.312	0.502	2.41E-81	C3	C1S	FALSE
1.29E-85	0.348985221	0.242	0.373	3.26E-81	C3	SERPINE1	FALSE
1.26E-84	0.302279755	0.238	0.361	3.19E-80	C3	OMD	FALSE

4.20E-82	0.278040665	0.128	0.204	1.06E-77	C3	TNRC6A	FALSE
1.50E-77	0.371639514	0.215	0.339	3.79E-73	C3	LAMB2	FALSE
2.57E-66	0.352912632	0.303	0.477	6.50E-62	C3	ZBTB20	TRUE
2.99E-56	0.39984775	0.254	0.39	7.56E-52	C3	SREK1	FALSE
2.83E-54	0.298114731	0.641	0.815	7.13E-50	C3	SPARC	FALSE
5.78E-49	0.543630841	0.318	0.329	1.46E-44	C3	COL1A2	FALSE
1.61E-48	0.392312686	0.339	0.533	4.07E-44	C3	AHI1	FALSE
4.05E-47	0.35521038	0.381	0.602	1.02E-42	C3	COL6A1	FALSE
3.59E-42	0.341480813	0.182	0.259	9.06E-38	C3	H19	FALSE
6.19E-40	0.521516114	0.491	0.667	1.56E-35	C3	COL11A2	FALSE
1.79E-35	0.255766273	0.463	0.739	4.52E-31	C3	RRBP1	FALSE
2.73E-32	0.344352688	0.155	0.217	6.89E-28	C3	AC058791	FALSE
1.52E-31	0.459249434	0.354	0.551	3.84E-27	C3	MT-ND4L	FALSE
1.02E-28	0.405772761	0.36	0.553	2.56E-24	C3	DDX17	FALSE
1.53E-25	0.503876081	0.218	0.217	3.85E-21	C3	COL3A1	FALSE
1.91E-19	0.426009208	0.197	0.266	4.81E-15	C3	CILP2	FALSE
3.00E-10	0.452498606	0.366	0.473	7.56E-06	C3	CILP	FALSE
8.23E-10	0.455741827	0.257	0.349	2.08E-05	C3	NKTR	FALSE
0	1.64630302	0.98	0.939	0	C4	NEAT1	FALSE
0	1.205344294	0.999	0.999	0	C4	MALAT1	FALSE
#######	1.203837775	0.62	0.613	9.90E-284	C4	DST	FALSE
#######	1.20125944	0.576	0.568	8.02E-202	C4	MEG3	FALSE
#######	0.26807006	0.131	0.379	9.27E-131	C4	RAB21	FALSE
#######	0.332189823	0.172	0.455	2.30E-130	C4	HP1BP3	TRUE
#######	0.263343834	0.174	0.446	1.45E-120	C4	CALD1	FALSE
#######	0.304277073	0.153	0.405	1.89E-117	C4	SCAF11	FALSE
#######	0.256460836	0.118	0.339	2.72E-115	C4	MPP6	FALSE
#######	0.280187384	0.137	0.368	8.28E-112	C4	YME1L1	FALSE
#######	0.470870911	0.245	0.578	3.44E-111	C4	HSPD1	FALSE
#######	0.313228962	0.221	0.526	5.80E-109	C4	DDX3Y	FALSE
#######	0.252303332	0.108	0.311	4.15E-106	C4	RHOQ	FALSE
#######	0.27845265	0.13	0.349	4.38E-105	C4	FERMT2	FALSE
#######	0.275380081	0.214	0.504	8.23E-105	C4	DDX3X	FALSE
#######	0.269603472	0.14	0.366	1.11E-104	C4	RUNX1	TRUE
#######	0.41600287	0.173	0.424	3.72E-101	C4	EIF5B	FALSE
#######	0.340712965	0.2	0.475	8.94E-101	C4	ARGLU1	FALSE
#######	0.341535906	0.153	0.384	1.59E-98	C4	NR3C1	TRUE
#######	0.25165415	0.094	0.275	1.06E-96	C4	HBP1	TRUE
#######	0.340178386	0.143	0.363	1.29E-96	C4	QKI	FALSE
#######	0.314031079	0.24	0.52	4.58E-96	C4	SOD2	FALSE
#######	0.257979227	0.11	0.3	1.56E-95	C4	PHF3	FALSE
5.14E-99	0.272185449	0.099	0.281	1.30E-94	C4	DHX36	TRUE
4.96E-98	0.336851426	0.117	0.313	1.25E-93	C4	CSNK1A1	FALSE
6.78E-97	0.336180346	0.193	0.452	1.71E-92	C4	SF1	TRUE
1.06E-96	0.274845828	0.179	0.419	2.67E-92	C4	MIDN	FALSE
2.87E-96	0.263850076	0.115	0.306	7.25E-92	C4	RIOK3	FALSE
1.35E-95	0.324754295	0.18	0.422	3.41E-91	C4	TRA2A	FALSE
2.43E-95	0.260009733	0.081	0.244	6.14E-91	C4	TROVE2	TRUE
1.76E-93	0.340836683	0.182	0.425	4.44E-89	C4	MEF2A	TRUE
1.80E-93	0.321338825	0.253	0.561	4.55E-89	C4	MBNL1	FALSE
7.39E-93	0.298876915	0.121	0.311	1.86E-88	C4	ELL2	FALSE
8.30E-93	0.28468782	0.115	0.302	2.10E-88	C4	C6orf62	FALSE
2.04E-92	0.261211858	0.099	0.272	5.15E-88	C4	ARHGAP5	FALSE
2.48E-91	0.285490126	0.104	0.281	6.26E-87	C4	VCP	FALSE
2.86E-90	0.369809256	0.192	0.44	7.23E-86	C4	IDS	FALSE
1.01E-88	0.3715338	0.249	0.553	2.54E-84	C4	PNN	FALSE
5.32E-88	0.279722067	0.099	0.268	1.34E-83	C4	ACTN1	FALSE
8.52E-88	0.254723927	0.317	0.687	2.15E-83	C4	HNRNPC	TRUE

2.07E-87	0.339214594	0.189	0.429	5.21E-83	C4	FNDC3B	FALSE
4.25E-87	0.36894008	0.158	0.373	1.07E-82	C4	USP16	FALSE
5.71E-87	0.347632877	0.272	0.598	1.44E-82	C4	HNRNPA3	FALSE
1.49E-86	0.394526382	0.234	0.52	3.76E-82	C4	PRRC2C	FALSE
6.14E-86	0.366885897	0.158	0.371	1.55E-81	C4	KMT2E	FALSE
9.36E-85	0.288191163	0.099	0.264	2.36E-80	C4	TCF7L2	TRUE
2.23E-84	0.346076984	0.192	0.433	5.63E-80	C4	SEC31A	FALSE
1.48E-83	0.289126924	0.098	0.26	3.73E-79	C4	KMT2A	TRUE
1.51E-83	0.25237519	0.086	0.24	3.80E-79	C4	SAFB2	TRUE
2.38E-83	0.358765378	0.219	0.478	6.00E-79	C4	UGP2	TRUE
4.99E-82	0.338845006	0.103	0.269	1.26E-77	C4	MAP4	FALSE
8.13E-82	0.3571763	0.117	0.293	2.05E-77	C4	G3BP1	FALSE
8.89E-81	0.257732025	0.297	0.623	2.24E-76	C4	PLOD2	FALSE
1.62E-80	0.397746455	0.174	0.393	4.09E-76	C4	SLC25A36	FALSE
2.12E-80	0.347166748	0.12	0.297	5.35E-76	C4	ZNF644	TRUE
6.56E-79	0.398405679	0.135	0.319	1.65E-74	C4	ZFHX3	TRUE
1.38E-78	0.256910164	0.097	0.252	3.48E-74	C4	ATP2B1	FALSE
1.59E-78	0.359351918	0.125	0.302	4.01E-74	C4	GFPT1	FALSE
3.34E-78	0.288709164	0.079	0.223	8.43E-74	C4	EPS8	FALSE
3.96E-78	0.268879133	0.101	0.259	9.99E-74	C4	TSC22D2	FALSE
6.90E-78	0.426075799	0.174	0.387	1.74E-73	C4	BCL6	TRUE
2.23E-77	0.330528698	0.124	0.298	5.62E-73	C4	SENP6	FALSE
3.25E-77	0.388867229	0.195	0.426	8.20E-73	C4	TSPYL2	FALSE
4.43E-77	0.344612107	0.129	0.308	1.12E-72	C4	GCC2	FALSE
2.12E-76	0.303001416	0.104	0.263	5.34E-72	C4	SFT2D2	FALSE
3.80E-76	0.615161081	0.167	0.376	9.59E-72	C4	BAG3	FALSE
1.08E-75	0.373639556	0.225	0.48	2.73E-71	C4	SLC25A37	FALSE
6.37E-75	0.346407305	0.122	0.292	1.61E-70	C4	ANKRD36	FALSE
7.47E-74	0.422852954	0.123	0.293	1.88E-69	C4	ETNK1	FALSE
8.74E-74	0.314119558	0.086	0.229	2.21E-69	C4	NUDT4	FALSE
1.04E-73	0.426159824	0.207	0.448	2.63E-69	C4	TCF25	FALSE
1.83E-73	0.374606412	0.124	0.294	4.63E-69	C4	PRPF38B	FALSE
1.86E-72	0.406997378	0.278	0.588	4.70E-68	C4	RTN4	FALSE
2.58E-72	0.250019376	0.081	0.218	6.51E-68	C4	UPF2	FALSE
3.84E-72	0.442172065	0.156	0.35	9.69E-68	C4	GPBP1	TRUE
6.08E-72	0.273476261	0.082	0.221	1.54E-67	C4	ARMCX3	FALSE
7.16E-72	0.380646305	0.197	0.423	1.81E-67	C4	FAM133B	FALSE
1.56E-71	0.43751211	0.124	0.292	3.94E-67	C4	MIR100HG	FALSE
1.77E-71	0.353952409	0.107	0.263	4.47E-67	C4	CCDC186	FALSE
4.02E-71	0.317324799	0.092	0.235	1.01E-66	C4	NSD3	FALSE
4.76E-71	0.308195772	0.112	0.27	1.20E-66	C4	INSIG1	FALSE
7.20E-71	0.28668911	0.092	0.236	1.82E-66	C4	ELF2	TRUE
1.13E-70	0.313636799	0.102	0.252	2.86E-66	C4	USP53	FALSE
1.16E-70	0.302020918	0.098	0.244	2.93E-66	C4	SNRNP70	TRUE
1.17E-70	0.366935522	0.135	0.309	2.95E-66	C4	CREBRF	FALSE
1.49E-70	0.250180247	0.081	0.217	3.75E-66	C4	HIPK3	FALSE
9.49E-69	0.307962703	0.09	0.229	2.39E-64	C4	TMEM165	FALSE
2.81E-67	0.324640967	0.1	0.244	7.10E-63	C4	SNHG25	FALSE
1.65E-66	0.271024044	0.084	0.216	4.16E-62	C4	PPP1R15B	FALSE
1.69E-66	0.38806744	0.134	0.302	4.28E-62	C4	PCF11	FALSE
4.57E-66	0.399737211	0.096	0.237	1.15E-61	C4	UBE2H	FALSE
5.03E-66	0.264499577	0.079	0.207	1.27E-61	C4	IPO7	FALSE
1.97E-65	0.373097293	0.188	0.397	4.98E-61	C4	RSRP1	FALSE
1.69E-64	0.288076404	0.093	0.228	4.25E-60	C4	MED13L	FALSE
1.00E-63	0.341997247	0.091	0.225	2.53E-59	C4	SRSF1	FALSE
1.26E-63	0.502321687	0.206	0.431	3.17E-59	C4	PDE4DIP	FALSE
1.33E-63	0.329637696	0.107	0.251	3.36E-59	C4	GFPT2	FALSE
2.00E-63	0.354427271	0.107	0.252	5.05E-59	C4	CTNNAL1	FALSE

4.60E-63	0.461587998	0.187	0.393	1.16E-58	C4	STAT3	TRUE
2.52E-62	0.293102932	0.082	0.209	6.36E-58	C4	MAP4K5	FALSE
7.99E-62	0.407854478	0.241	0.492	2.02E-57	C4	SKIL	TRUE
1.25E-61	0.303843503	0.095	0.228	3.17E-57	C4	PRDM2	TRUE
3.14E-61	0.411494041	0.145	0.313	7.93E-57	C4	PPP1R10	TRUE
3.24E-61	0.324541266	0.102	0.24	8.19E-57	C4	COPA	FALSE
5.48E-61	0.374082041	0.109	0.252	1.38E-56	C4	NDRG1	FALSE
1.78E-60	0.329122168	0.104	0.242	4.49E-56	C4	ZBTB21	TRUE
2.18E-59	0.404185671	0.268	0.534	5.51E-55	C4	MCL1	FALSE
9.27E-59	0.432549056	0.116	0.26	2.34E-54	C4	DNAJB4	FALSE
4.86E-58	0.341154055	0.121	0.267	1.23E-53	C4	PER1	FALSE
6.67E-58	0.342323953	0.1	0.233	1.68E-53	C4	KIF21A	FALSE
1.64E-57	0.424975226	0.166	0.342	4.13E-53	C4	LPP	FALSE
3.47E-57	0.419210969	0.128	0.278	8.75E-53	C4	RAPH1	FALSE
4.30E-57	0.301908161	0.087	0.21	1.08E-52	C4	ANKRD11	FALSE
4.01E-56	0.367887264	0.092	0.216	1.01E-51	C4	SAMD4A	FALSE
6.36E-56	0.36387616	0.099	0.228	1.60E-51	C4	NRP2	FALSE
8.14E-56	0.290457457	0.087	0.208	2.05E-51	C4	GPATCH2	FALSE
1.24E-55	0.284643218	0.087	0.207	3.13E-51	C4	NIPBL	FALSE
2.10E-55	0.363696404	0.106	0.239	5.30E-51	C4	RANBP2	FALSE
3.10E-55	0.462203949	0.252	0.504	7.82E-51	C4	SRSF11	FALSE
4.35E-55	0.364504058	0.09	0.212	1.10E-50	C4	AHCYL1	FALSE
8.00E-55	0.454660777	0.221	0.441	2.02E-50	C4	LRRFIP1	TRUE
1.09E-54	0.40903196	0.143	0.301	2.75E-50	C4	RNF19A	FALSE
1.20E-54	0.36630842	0.117	0.257	3.02E-50	C4	BOD1L1	FALSE
6.29E-54	0.386095172	0.121	0.261	1.59E-49	C4	UACA	FALSE
9.00E-54	0.281363274	0.361	0.704	2.27E-49	C4	HNRNPH1	FALSE
5.28E-53	0.510361001	0.219	0.437	1.33E-48	C4	TAF1D	FALSE
6.56E-53	0.440367239	0.121	0.261	1.66E-48	C4	SRSF4	FALSE
1.31E-52	0.431240908	0.229	0.45	3.30E-48	C4	TRPS1	TRUE
1.89E-52	0.361194113	0.344	0.694	4.77E-48	C4	SON	TRUE
2.20E-52	0.448353472	0.143	0.298	5.55E-48	C4	CREB5	TRUE
3.24E-52	0.490235608	0.148	0.306	8.18E-48	C4	CPEB4	FALSE
7.82E-52	0.299151859	0.089	0.206	1.97E-47	C4	PDXDC1	FALSE
2.23E-51	0.438053198	0.157	0.32	5.63E-47	C4	PCM1	FALSE
2.61E-51	0.394209403	0.137	0.285	6.58E-47	C4	EPB41L2	FALSE
8.59E-51	0.333136473	0.138	0.283	2.17E-46	C4	NFKBIZ	FALSE
9.41E-51	0.330889269	0.094	0.212	2.37E-46	C4	ETS1	TRUE
1.01E-50	0.315991138	0.089	0.203	2.54E-46	C4	MAP3K2	FALSE
8.87E-50	0.35641549	0.095	0.213	2.24E-45	C4	ARIH1	FALSE
1.75E-49	0.438427659	0.11	0.238	4.41E-45	C4	ZNF326	TRUE
3.10E-49	0.383377413	0.122	0.256	7.83E-45	C4	LINC0063[illegible]	FALSE
7.24E-49	0.50817244	0.194	0.382	1.83E-44	C4	CLK1	TRUE
8.40E-48	0.345425447	0.091	0.204	2.12E-43	C4	UBE3A	FALSE
1.60E-47	0.459556247	0.225	0.429	4.03E-43	C4	NOVA1	FALSE
7.02E-47	0.356736798	0.104	0.223	1.77E-42	C4	PKN2	FALSE
1.64E-46	1.071020402	0.366	0.362	4.13E-42	C4	KCNQ1OT	FALSE
2.03E-46	0.320175059	0.092	0.202	5.12E-42	C4	PLK3	FALSE
8.65E-46	0.373088508	0.101	0.217	2.18E-41	C4	MSI2	TRUE
1.77E-45	0.373488961	0.112	0.233	4.46E-41	C4	MDM4	FALSE
2.84E-45	0.489303165	0.128	0.261	7.16E-41	C4	PELI1	FALSE
3.25E-44	0.400453884	0.097	0.208	8.19E-40	C4	PLEKHA1	FALSE
3.28E-44	0.417900975	0.103	0.218	8.27E-40	C4	DAAM1	FALSE
1.18E-43	0.457538625	0.239	0.454	2.97E-39	C4	CCDC88A	FALSE
8.53E-43	0.389654128	0.103	0.216	2.15E-38	C4	FNBP4	FALSE
2.33E-41	0.387934014	0.111	0.226	5.88E-37	C4	RF00100.4	FALSE
5.13E-41	0.538930329	0.23	0.435	1.29E-36	C4	IFRD1	FALSE
2.63E-40	0.401907849	0.113	0.228	6.64E-36	C4	TUT4	FALSE

6.33E-40	0.518351937	0.127	0.25	1.60E-35	C4	NCKAP1	FALSE
3.08E-39	0.393177402	0.101	0.207	7.77E-35	C4	PSMA3-A	FALSE
7.61E-39	0.40782522	0.109	0.22	1.92E-34	C4	PRKCA	FALSE
9.34E-39	0.489877609	0.121	0.24	2.36E-34	C4	PGM3	FALSE
2.06E-38	0.558004711	0.24	0.449	5.21E-34	C4	CHD9	FALSE
2.24E-38	0.447923401	0.119	0.234	5.66E-34	C4	ZNF292	TRUE
4.79E-37	0.491966029	0.144	0.274	1.21E-32	C4	CENPC	FALSE
1.24E-36	0.555153347	0.192	0.354	3.12E-32	C4	TLE4	FALSE
1.72E-36	0.424392662	0.338	0.626	4.34E-32	C4	COL6A2	FALSE
6.91E-36	0.465582961	0.111	0.218	1.74E-31	C4	EPM2AIP1	FALSE
2.45E-35	0.468932305	0.196	0.357	6.17E-31	C4	AC020916	FALSE
3.24E-34	0.531444238	0.177	0.323	8.18E-30	C4	WEE1	FALSE
1.76E-33	0.497308862	0.155	0.286	4.43E-29	C4	ASH1L	TRUE
8.14E-33	0.420888472	0.114	0.216	2.05E-28	C4	KDM6B	FALSE
3.35E-32	0.513324749	0.15	0.276	8.46E-28	C4	NFAT5	TRUE
7.42E-32	0.267284121	0.421	0.786	1.87E-27	C4	YBX3	TRUE
7.02E-31	0.367351147	0.387	0.724	1.77E-26	C4	DDX24	FALSE
1.47E-30	0.593743978	0.21	0.375	3.71E-26	C4	ANKRD28	FALSE
3.45E-30	0.580392973	0.184	0.329	8.70E-26	C4	AFF4	TRUE
9.46E-30	0.596796719	0.225	0.402	2.39E-25	C4	NUFIP2	FALSE
9.99E-30	0.458715991	0.111	0.208	2.52E-25	C4	ZNF638	FALSE
1.03E-26	0.455953894	0.113	0.205	2.60E-22	C4	AFDN	FALSE
1.36E-26	0.517956167	0.312	0.564	3.42E-22	C4	TRA2B	FALSE
2.43E-26	0.579033833	0.173	0.303	6.14E-22	C4	FNIP1	FALSE
8.06E-25	0.481548552	0.136	0.237	2.03E-20	C4	NFATC1	TRUE
1.68E-24	0.573076198	0.152	0.263	4.23E-20	C4	SLC20A1	FALSE
7.59E-24	0.770252205	0.477	0.639	1.91E-19	C4	CCNL1	FALSE
8.31E-24	0.650362919	0.252	0.439	2.10E-19	C4	ANKRD12	FALSE
3.90E-23	0.548300946	0.54	0.789	9.83E-19	C4	HNRNPA2	FALSE
7.11E-23	0.576570389	0.163	0.277	1.79E-18	C4	CD44	FALSE
9.31E-23	0.584682286	0.187	0.317	2.35E-18	C4	LDLR	FALSE
2.41E-22	0.474393009	0.117	0.203	6.09E-18	C4	USP34	FALSE
6.44E-22	0.529137728	0.135	0.23	1.63E-17	C4	RB1CC1	FALSE
9.91E-22	0.513862575	0.16	0.269	2.50E-17	C4	COL6A3	FALSE
1.39E-20	0.621370269	0.276	0.477	3.51E-16	C4	THUMPD3	FALSE
1.88E-20	0.5605617	0.15	0.25	4.74E-16	C4	INTS6	FALSE
1.92E-20	0.510409726	0.128	0.216	4.86E-16	C4	MACF1	FALSE
3.79E-20	0.563686305	0.15	0.251	9.57E-16	C4	CHD1	FALSE
1.88E-19	0.605240757	0.172	0.285	4.73E-15	C4	AKAP13	FALSE
3.64E-19	0.641974202	0.17	0.279	9.18E-15	C4	HMGCS1	FALSE
7.26E-19	0.512297868	0.125	0.207	1.83E-14	C4	SEC24D	FALSE
2.31E-18	0.530177544	0.348	0.622	5.82E-14	C4	PNISR	FALSE
6.84E-18	0.60222333	0.285	0.486	1.73E-13	C4	RBM25	FALSE
9.07E-18	0.610050686	0.162	0.264	2.29E-13	C4	BDP1	TRUE
2.35E-17	0.668424897	0.277	0.47	5.94E-13	C4	LUC7L3	FALSE
2.73E-17	0.842853973	0.231	0.382	6.89E-13	C4	SLC5A3	FALSE
4.38E-17	0.650779547	0.237	0.391	1.10E-12	C4	BCLAF1	TRUE
1.46E-16	0.61831735	0.253	0.417	3.69E-12	C4	AKAP9	FALSE
4.85E-16	0.52369028	0.126	0.203	1.22E-11	C4	CDH19	FALSE
2.41E-15	0.634739652	0.147	0.235	6.08E-11	C4	SPEN	TRUE
1.93E-14	0.617566573	0.164	0.259	4.87E-10	C4	EWSR1	TRUE
7.54E-14	0.603813255	0.316	0.536	1.90E-09	C4	ZRANB2	FALSE
1.00E-13	0.670393968	0.268	0.439	2.53E-09	C4	SRSF10	FALSE
7.36E-13	0.774386278	0.449	0.628	1.86E-08	C4	WSB1	FALSE
1.21E-12	0.902314557	0.427	0.585	3.06E-08	C4	HNRNPU	FALSE
3.47E-11	0.619612515	0.134	0.203	8.76E-07	C4	GPSM2	FALSE
8.60E-11	0.680515344	0.22	0.341	2.17E-06	C4	PRPF4B	FALSE
8.88E-11	0.650894892	0.23	0.354	2.24E-06	C4	COL27A1	FALSE

1.22E-10	0.898996515	0.386	0.509	3.08E-06	C4	GLS	FALSE
1.83E-10	0.724770508	0.295	0.476	4.62E-06	C4	RNMT	FALSE
4.05E-10	0.64272233	0.189	0.286	1.02E-05	C4	SMC5	FALSE
7.13E-10	0.487373169	0.397	0.693	1.80E-05	C4	RBM39	FALSE
1.02E-08	0.68102214	0.304	0.49	0.0002576	C4	JMJD1C	FALSE
2.10E-08	0.651473824	0.155	0.227	0.0005311	C4	GLIS3	TRUE
2.84E-08	0.65542141	0.186	0.274	0.0007166	C4	GPRC5A	FALSE
3.33E-08	0.747826634	0.22	0.332	0.0008409	C4	ABI2	FALSE
7.05E-08	0.620935837	0.159	0.23	0.00178	C4	ARID1B	FALSE
4.39E-07	0.657425736	0.161	0.231	0.0110699	C4	NFATC2	TRUE
0	1.23900603	0.803	0.374	0	C5	CHRDL2	FALSE
0	0.997889444	0.936	0.521	0	C5	CAPS	FALSE
0	0.935854883	0.981	0.554	0	C5	OGN	FALSE
0	0.857982213	0.893	0.302	0	C5	VCAN	FALSE
0	0.841117968	0.916	0.386	0	C5	ABI3BP	FALSE
0	0.820296785	0.546	0.131	0	C5	CRISPLD1	FALSE
0	0.717771717	0.948	0.503	0	C5	CP	FALSE
0	0.668501548	0.582	0.131	0	C5	SPON2	FALSE
0	0.654663587	0.872	0.416	0	C5	IGFBP7	FALSE
0	0.649105758	0.993	0.888	0	C5	RPL13A	FALSE
0	0.646080138	0.973	0.712	0	C5	NDUFA4L2	FALSE
0	0.628525945	0.942	0.494	0	C5	PLXDC2	FALSE
0	0.620597284	0.905	0.563	0	C5	KLF2	TRUE
0	0.604907887	0.734	0.215	0	C5	ANGPTL5	FALSE
0	0.599871697	0.266	0.075	0	C5	ASPN	FALSE
0	0.582325139	0.987	0.861	0	C5	FOS	TRUE
0	0.559515771	0.934	0.769	0	C5	JUN	TRUE
0	0.554450325	0.979	0.741	0	C5	CNMD	FALSE
0	0.544907821	0.878	0.391	0	C5	SERPINA5	FALSE
0	0.521119965	0.514	0.1	0	C5	THY1	FALSE
0	0.515495717	0.543	0.218	0	C5	NRN1	FALSE
0	0.515171333	0.985	0.823	0	C5	RPL7	FALSE
0	0.47371978	0.682	0.252	0	C5	INHBA	FALSE
0	0.472857238	0.992	0.751	0	C5	BGN	FALSE
0	0.465359152	0.686	0.337	0	C5	ADM	FALSE
0	0.464122184	0.988	0.847	0	C5	RPS20	FALSE
0	0.4639879	0.949	0.606	0	C5	EID1	FALSE
0	0.462351933	0.911	0.561	0	C5	CYR61	FALSE
0	0.460925644	0.634	0.268	0	C5	TXNIP	FALSE
0	0.45458595	0.784	0.542	0	C5	ATF3	TRUE
0	0.451203705	0.24	0.074	0	C5	ORM1	FALSE
0	0.442749033	0.995	0.889	0	C5	RPS2	FALSE
0	0.440166525	0.864	0.444	0	C5	CTSD	FALSE
0	0.439501393	0.775	0.39	0	C5	ID1	TRUE
0	0.439099149	0.957	0.742	0	C5	FOSB	TRUE
0	0.430665304	0.994	0.887	0	C5	RPL3	FALSE
0	0.430121794	0.352	0.046	0	C5	WIF1	FALSE
0	0.429676829	0.678	0.242	0	C5	PAX1	TRUE
0	0.425736717	0.75	0.346	0	C5	FHL1	FALSE
0	0.424533693	1	0.981	0	C5	EEF1A1	FALSE
0	0.421039783	0.945	0.627	0	C5	EIF3E	FALSE
0	0.419667869	0.953	0.704	0	C5	RPL31	FALSE
0	0.406614211	0.871	0.488	0	C5	ENO1	TRUE
0	0.405081386	0.887	0.601	0	C5	DUSP1	FALSE
0	0.404153631	0.996	0.911	0	C5	PRELP	FALSE
0	0.399146012	0.822	0.569	0	C5	IER2	FALSE
0	0.399053334	0.811	0.372	0	C5	SDC2	FALSE
0	0.394163135	0.402	0.066	0	C5	MYLK	TRUE

0	0.393864158	0.55	0.291	0	C5	A2M	FALSE
0	0.386931853	0.675	0.222	0	C5	COL5A2	FALSE
0	0.386388761	0.809	0.411	0	C5	P4HA1	FALSE
0	0.381162314	0.81	0.402	0	C5	EIF4B	FALSE
0	0.376977288	0.992	0.867	0	C5	RPLP0	FALSE
0	0.376641267	0.826	0.461	0	C5	ID4	TRUE
0	0.376024423	0.974	0.764	0	C5	RPSA	FALSE
0	0.371080388	0.944	0.612	0	C5	HTRA1	FALSE
0	0.369521401	0.723	0.423	0	C5	BTG2	FALSE
0	0.368711516	0.945	0.633	0	C5	EEF2	FALSE
0	0.36824758	0.857	0.46	0	C5	P4HB	TRUE
0	0.367956743	0.863	0.481	0	C5	PDIA6	FALSE
0	0.366782807	0.515	0.131	0	C5	CD82	FALSE
0	0.363563282	0.966	0.741	0	C5	ACTG1	FALSE
0	0.361016834	0.718	0.365	0	C5	BNIP3	FALSE
0	0.35994068	0.509	0.232	0	C5	OTUD1	FALSE
0	0.359776249	0.947	0.631	0	C5	CD99	FALSE
0	0.35767296	0.45	0.101	0	C5	AGT	FALSE
0	0.357407318	0.995	0.9	0	C5	RPL5	FALSE
0	0.357375304	0.996	0.905	0	C5	RPS11	FALSE
0	0.356923742	0.645	0.231	0	C5	GALNT1	FALSE
0	0.354253428	0.884	0.518	0	C5	FXYD6	FALSE
0	0.353241846	0.245	0.042	0	C5	DCLK1	FALSE
0	0.351585306	0.845	0.454	0	C5	SSR2	FALSE
0	0.350939334	1	0.98	0	C5	FTL	FALSE
0	0.347691268	0.504	0.138	0	C5	ECM2	FALSE
0	0.347422434	0.777	0.527	0	C5	ZFP36	FALSE
0	0.346100251	0.999	0.943	0	C5	RPS8	FALSE
0	0.345570885	0.995	0.898	0	C5	RPS16	FALSE
0	0.344501807	0.846	0.43	0	C5	CPE	FALSE
0	0.341902617	0.891	0.532	0	C5	VKORC1	FALSE
0	0.339262161	0.589	0.19	0	C5	P4HA2	FALSE
0	0.337777542	1	0.975	0	C5	TPT1	FALSE
0	0.337597502	0.959	0.671	0	C5	HLA-A	FALSE
0	0.33579091	0.946	0.73	0	C5	AL078639	FALSE
0	0.335475823	0.925	0.604	0	C5	NPC2	FALSE
0	0.33460996	0.87	0.534	0	C5	RHOB	FALSE
0	0.332635227	0.998	0.946	0	C5	RPL19	FALSE
0	0.332422801	0.468	0.101	0	C5	ATRNL1	FALSE
0	0.332136939	0.913	0.579	0	C5	ST13	FALSE
0	0.330837862	0.562	0.202	0	C5	SNAI2	TRUE
0	0.330729603	0.649	0.419	0	C5	MT1F	FALSE
0	0.330189551	0.714	0.371	0	C5	TIPARP	FALSE
0	0.329778232	0.997	0.913	0	C5	RPL23A	FALSE
0	0.326857185	0.701	0.269	0	C5	TGOLN2	FALSE
0	0.323767074	0.879	0.512	0	C5	RPL17	FALSE
0	0.32331061	0.447	0.091	0	C5	SLC44A1	FALSE
0	0.322277882	0.738	0.359	0	C5	PGK1	FALSE
0	0.32171675	0.829	0.425	0	C5	CRTAP	FALSE
0	0.321657522	0.969	0.744	0	C5	RACK1	FALSE
0	0.320334639	0.368	0.096	0	C5	DIO2	FALSE
0	0.319711821	0.762	0.368	0	C5	EDIL3	FALSE
0	0.319537578	0.998	0.932	0	C5	RPL15	FALSE
0	0.317140699	0.664	0.323	0	C5	CDR1	FALSE
0	0.315422638	0.513	0.234	0	C5	TIMP4	FALSE
0	0.315209299	0.785	0.415	0	C5	RPS17	FALSE
0	0.313892138	0.262	0.036	0	C5	SPTSSB	FALSE
0	0.312792987	0.955	0.683	0	C5	RPL4	FALSE

0	0.311693511	0.545	0.156	0	C5	CSGALNA	FALSE
0	0.311271489	0.841	0.467	0	C5	SLC25A6	FALSE
0	0.309662203	0.256	0.073	0	C5	LTBP2	FALSE
0	0.305492323	0.713	0.388	0	C5	ID2	TRUE
0	0.305070761	1	0.972	0	C5	RPL10	FALSE
0	0.303982949	0.895	0.604	0	C5	KLF4	TRUE
0	0.301646011	0.967	0.744	0	C5	EEF1D	TRUE
0	0.301168852	0.848	0.48	0	C5	PKM	TRUE
0	0.301118184	0.953	0.659	0	C5	HLA-C	FALSE
0	0.300775353	0.417	0.101	0	C5	AHNAK2	FALSE
0	0.300715698	0.948	0.678	0	C5	RPS4Y1	FALSE
0	0.296486883	0.665	0.281	0	C5	BARX1	TRUE
0	0.296377018	0.394	0.079	0	C5	NRTN	FALSE
0	0.295659181	0.681	0.35	0	C5	BAMBI	FALSE
0	0.295503403	0.877	0.542	0	C5	PRDX4	FALSE
0	0.295246405	0.67	0.255	0	C5	CPQ	FALSE
0	0.294995772	0.766	0.376	0	C5	ISLR	FALSE
0	0.294156399	0.601	0.225	0	C5	MLEC	FALSE
0	0.293661861	0.39	0.076	0	C5	MATN2	FALSE
0	0.293462139	0.81	0.408	0	C5	TTC3	FALSE
0	0.293185565	0.996	0.909	0	C5	RPL7A	FALSE
0	0.291092652	0.889	0.539	0	C5	TOMM20	FALSE
0	0.290838794	0.542	0.167	0	C5	TLN1	FALSE
0	0.290801842	0.596	0.225	0	C5	NFIX	TRUE
0	0.288412388	0.533	0.205	0	C5	METRN	FALSE
0	0.286623417	0.729	0.324	0	C5	DPP7	FALSE
0	0.286246267	0.789	0.423	0	C5	WWP2	FALSE
0	0.286015991	0.962	0.689	0	C5	PSAP	FALSE
0	0.285245064	0.469	0.131	0	C5	PLOD1	FALSE
0	0.284334308	0.588	0.265	0	C5	TPM1	FALSE
0	0.284231378	0.914	0.58	0	C5	COL9A2	FALSE
0	0.283941623	0.788	0.411	0	C5	C6orf48	FALSE
0	0.279313333	0.983	0.801	0	C5	ACTB	FALSE
0	0.279172568	0.331	0.075	0	C5	NKX3-2	TRUE
0	0.278134221	0.996	0.916	0	C5	RPS3	FALSE
0	0.278028514	0.939	0.648	0	C5	ATP5MC2	FALSE
0	0.276107651	0.808	0.417	0	C5	SDF4	FALSE
0	0.274849915	0.821	0.453	0	C5	ATRAID	FALSE
0	0.273102046	0.921	0.629	0	C5	NAP1L1	TRUE
0	0.272956718	0.999	0.95	0	C5	RPS6	FALSE
0	0.271737991	0.967	0.729	0	C5	CD81	FALSE
0	0.270288153	0.644	0.282	0	C5	SSPN	FALSE
0	0.267964609	0.754	0.36	0	C5	CANX	TRUE
0	0.267287853	0.586	0.217	0	C5	CHADL	FALSE
0	0.265941446	0.5	0.162	0	C5	ATP1B1	FALSE
0	0.265356647	0.988	0.824	0	C5	RPL22	FALSE
0	0.263774739	0.812	0.458	0	C5	ENPP1	FALSE
0	0.263755284	0.919	0.606	0	C5	FBXO2	FALSE
0	0.26356087	0.993	0.881	0	C5	RPL29	FALSE
0	0.263039382	0.625	0.322	0	C5	RGS3	FALSE
0	0.262867723	0.295	0.048	0	C5	CCDC8	FALSE
0	0.262251689	0.712	0.328	0	C5	EIF3L	FALSE
0	0.260721019	0.472	0.15	0	C5	AL035258	FALSE
0	0.259035589	0.307	0.064	0	C5	DTNA	FALSE
0	0.257706616	0.907	0.566	0	C5	NUCKS1	FALSE
0	0.257342655	0.996	0.902	0	C5	RPS9	FALSE
0	0.25636811	0.613	0.231	0	C5	ASPH	FALSE
0	0.256169139	0.678	0.277	0	C5	NFIA	TRUE

0	0.255808591	0.743	0.355	0	C5	TMEM167	FALSE
0	0.253349795	0.33	0.082	0	C5	HS3ST1	FALSE
0	0.253023978	0.897	0.568	0	C5	ITM2A	FALSE
0	0.25233789	0.674	0.293	0	C5	RNF130	FALSE
0	0.252201388	0.963	0.732	0	C5	EIF3H	FALSE
0	0.25125755	0.359	0.075	0	C5	FGFR2	FALSE
0	0.250544755	0.623	0.243	0	C5	LAMP2	FALSE
0	1.593618575	0.719	0.298	0	C6	CHI3L2	FALSE
0	1.387460713	0.655	0.241	0	C6	CHI3L1	FALSE
0	0.886913574	0.976	0.805	0	C6	FN1	FALSE
0	0.870010461	0.785	0.542	0	C6	MT1M	FALSE
0	0.85665523	0.379	0.115	0	C6	PRG4	FALSE
0	0.717816921	0.984	0.923	0	C6	MT1E	FALSE
0	0.709268453	0.998	0.984	0	C6	MT2A	FALSE
0	0.659899186	0.39	0.187	0	C6	PLA2G2A	FALSE
0	0.622209398	0.998	0.964	0	C6	LUM	FALSE
0	0.617237168	0.895	0.613	0	C6	IFITM3	FALSE
0	0.592935383	0.892	0.632	0	C6	NNMT	FALSE
0	0.578259602	0.395	0.187	0	C6	MMP3	FALSE
0	0.546584418	0.848	0.624	0	C6	SERPINE2	FALSE
0	0.507747853	0.703	0.394	0	C6	IFITM2	FALSE
0	0.493846832	1	0.987	0	C6	CLU	FALSE
0	0.487855457	0.853	0.612	0	C6	GPX3	FALSE
0	0.474808057	0.459	0.143	0	C6	CRLF1	FALSE
0	0.473019535	0.998	0.964	0	C6	MT1X	FALSE
0	0.445557546	0.305	0.114	0	C6	CFD	FALSE
0	0.425035006	0.955	0.804	0	C6	GAPDH	FALSE
0	0.407131444	0.562	0.343	0	C6	MT1L	FALSE
0	0.406702315	0.908	0.7	0	C6	PCOLCE2	FALSE
0	0.396523718	0.29	0.109	0	C6	GLRX	FALSE
0	0.390028234	0.534	0.33	0	C6	CDO1	FALSE
0	0.384345803	0.959	0.773	0	C6	JUNB	TRUE
0	0.377066108	0.559	0.344	0	C6	SLC30A1	FALSE
0	0.376300438	0.904	0.665	0	C6	PLAC9	FALSE
0	0.351450037	0.251	0.072	0	C6	ANGPTL4	FALSE
0	0.336653941	1	0.99	0	C6	DCN	FALSE
0	0.329628262	0.584	0.375	0	C6	CLEC11A	FALSE
0	0.329240796	0.301	0.07	0	C6	CCND1	FALSE
0	0.324900795	0.438	0.214	0	C6	ZIC1	TRUE
0	0.316415665	0.87	0.645	0	C6	LDHA	FALSE
0	0.313376379	0.524	0.315	0	C6	PCOLCE	FALSE
0	0.287763258	0.85	0.629	0	C6	MFGE8	FALSE
0	0.263628595	0.725	0.47	0	C6	FXYD5	FALSE
#######	0.583394767	0.416	0.249	1.46E-300	C6	MT1A	FALSE
#######	0.621640238	0.912	0.836	2.04E-292	C6	MT1G	FALSE
#######	0.275711962	0.974	0.854	1.92E-240	C6	TIMP1	FALSE
