# Supplementary material for: Spatially defined single-cell transcriptional profiling characterizes diverse chondrocyte subtypes and nucleus pulposus progenitors in human intervertebral discs
Source: Bone Res. 2021 Aug 16;9:37. doi: 10.1038/s41413-021-00163-z (PMC8368097; doi:10.1038/s41413-021-00163-z)
Supplement: Supplementary file 5 — Supplementary Table 4 [file 41413_2021_163_MOESM5_ESM.pdf]

Supplementary Table 4.
Annotation of matrisome genes detected in IVD cells

Division	Category	Gene Symbol	Gene Name	Synonyms	HGNC_IDs	HGNC_IDs Links	UniProt_IDs	Refseq_IDs	Orthology	Notes
Core matrisome	ECM Glycoproteins	ABI3BP	ABI family, mem	FLJ41743|FLJ41	17265	17265	B4DSV9:D3YTG3:E9PP	NP_056244.2:XP	Mouse:Abi3bp|	NA
Core matrisome	ECM Glycoproteins	AEBP1	AE binding prote	ACLP; FLJ33612	303	303	C9JLQ8:H7C0W8:H7C1	NP_001120.3	Mouse:Aebp1|	NA
Core matrisome	ECM Glycoproteins	AGRN	agrin	FLJ45064	329	329	H0Y5U1:O00468	NP_940978.2:XP	Mouse:Agrn|	NA
Core matrisome	ECM Glycoproteins	AMBN	ameloblastin (en	-	452	452	Q9NP70	NP_057603.1	Mouse:Ambn|	NA
Core matrisome	ECM Glycoproteins	BGLAP	bone gamma-ca	BGP|OC|PMF1	1043	1043	P02818	NP_954642.1	Mouse:Bglap2|	NA
Core matrisome	ECM Glycoproteins	BMPER	BMP binding en	CRIM3|CV-2|CY	24154	24154	C9JY72:F8WDG9:G5E9	NP_597725.1	Mouse:Bmper|	Growth Factor-binding
Core matrisome	ECM Glycoproteins	CILP	cartilage interme	CILP-1|HsT188	1980	1980	O75339	NP_003604.3	Mouse:Cilp|	NA
Core matrisome	ECM Glycoproteins	CILP2	cartilage interme	CLIP-2|MGC45	24213	24213	K7EPJ4:Q8IUL8	NP_694953.2	Mouse:Cilp2|	NA
Core matrisome	ECM Glycoproteins	COCH	coagulation fact	COCH-5B2|CO	2180	2180	E7EN67:G3V4C4:G3V5	NP_001128530.1	Mouse:Coch|	NA
Core matrisome	ECM Glycoproteins	COLQ	collagen-like tai	EAD|FLJ55041	2226	2226	C9JBB2:F8WA67:Q9Y2	NP_005668.2:NP	Mouse:Colq|	Basement Membrane
Core matrisome	ECM Glycoproteins	COMP	cartilage oligom	EDM1|EPD1|MI	2227	2227	B4DKJ3:G3XAP6:P4974	NP_000086.2	Mouse:Comp|	NA
Core matrisome	ECM Glycoproteins	CRELD1	cysteine-rich wit	AVSD2|CIRRIN	14630	14630	F8WBY3:H7C2L3:Q96H	NP_001026887.1	Mouse:Creld1|	NA
Core matrisome	ECM Glycoproteins	CRELD2	cysteine-rich wit	DKFZp667O05	28150	28150	A6PWM2:Q6UXH1	NP_001128573.1	Mouse:Creld2|	NA
Core matrisome	ECM Glycoproteins	CRIM1	cysteine rich tra	MGC138194|S5	2359	2359	H7C1Z0:H7C2T6:H7C4	NP_057525.1	Mouse:Crim1|	NA
Core matrisome	ECM Glycoproteins	CRISPLD1	cysteine-rich se	CRISP10|DKFZ	18206	18206	B7Z8V9:E5RJS4:Q9H33	NP_001273707.1	Mouse:Crispld1|	NA
Core matrisome	ECM Glycoproteins	CRISPLD2	cysteine-rich se	CRISP11|DKFZ	25248	25248	H3BS62:H3BSZ9:H3BT	NP_113664.1:XP	Mouse:Crispld2|	NA
Core matrisome	ECM Glycoproteins	CTGF	connective tissu	CCN2|HCS24|I	2500	2500	P29279	NP_001892.1	Mouse:Ctgf|	CCN Family
Core matrisome	ECM Glycoproteins	CTHRC1	collagen triple h	-	18831	18831	E5RK99:E7EVQ5:Q96C	NP_001243028.1	Mouse:Cthrc1|	NA
Core matrisome	ECM Glycoproteins	CYR61	cysteine-rich, an	CCN1|GIG1|IGF	2654	2654	O00622	NP_001545.2	Mouse:Cyr61|	CCN Family
Core matrisome	ECM Glycoproteins	DPT	dermatopontin	TRAMP	3011	3011	Q07507	NP_001928.2	Mouse:Dpt|	NA
Core matrisome	ECM Glycoproteins	ECM1	extracellular mat	-	3153	3153	Q16610	NP_001189787.1	Mouse:Ecm1|	NA
Core matrisome	ECM Glycoproteins	ECM2	extracellular mat	MGC126355|M	3154	3154	O94769:Q5T9F3:Q5T9	NP_001184224.1	Mouse:Ecm2|	NA
Core matrisome	ECM Glycoproteins	EDIL3	EGF-like repeats	DEL1|MGC2628	3173	3173	O43854	NP_001265571.1	Mouse:Edil3|	NA
Core matrisome	ECM Glycoproteins	EFEMP1	EGF-containing	DHRD|DRAD|F	3218	3218	C9J4H7:C9J4J8:C9J8S9	NP_001034437.1	Mouse:Efemp1|	Fibulin
Core matrisome	ECM Glycoproteins	EFEMP2	EGF-containing	FBLN4|MBP1|U	3219	3219	E9PI47:E9PKA3:E9PNB	NP_058634.4	Mouse:Efemp2|	Fibulin
Core matrisome	ECM Glycoproteins	EGFLAM	EGF-like, fibrone	AGRINL|AGRN	26810	26810	D6RG24:D6RJD2:Q63H	NP_001192230.1	Mouse:Egflam|	NA
Core matrisome	ECM Glycoproteins	ELN	elastin	FLJ38671|FLJ43	3327	3327	E7EN51:E7EN65:E7EN	NP_000492.2:NP	Mouse:Eln|	NA
Core matrisome	ECM Glycoproteins	EMID1	EMI domain con	EMI5|EMU1|M	18036	18036	B0QYK2:B0QYK3:B0QY	NP_001254824.1	Mouse:Emid1|	NA
Core matrisome	ECM Glycoproteins	EMILIN1	elastin microfib	DKFZp586M12	19880	19880	H0Y7A0:Q9Y6C2	NP_008977.1	Mouse:Emilin1|	NA
Core matrisome	ECM Glycoproteins	EMILIN2	elastin microfib	EMILIN-2|FLJ3	19881	19881	Q9BXX0	NP_114437.2	Mouse:Emilin2|	NA
Core matrisome	ECM Glycoproteins	EMILIN3	elastin microfib	C20orf130|DKF	16123	16123	Q9NT22	NP_443078.1	Mouse:Emilin3|	NA
Core matrisome	ECM Glycoproteins	EYS	eyes shut homo	C6orf178|C6or	21555	21555	F8WDD3:H0Y3Q4:Q5T	NP_001136272.1	NA	NA
Core matrisome	ECM Glycoproteins	FBLN1	fibulin 1	FBLN|FIBL1	3600	3600	B1AHL2:B1AHM4:B1A	NP_001987.2:NP	Mouse:Fbln1|	Fibulin
Core matrisome	ECM Glycoproteins	FBLN2	fibulin 2	-	3601	3601	C9JQS6:F5H1F3:H7BX	NP_001004019.1	Mouse:Fbln2|	Fibulin
Core matrisome	ECM Glycoproteins	FBLN5	fibulin 5	ARMD3|DANC	3602	3602	G3V2P8:G3V329:G3V3	NP_006320.2	Mouse:Fbln5|	Fibulin
Core matrisome	ECM Glycoproteins	FBLN7	fibulin 7	DKFZp547D06	26740	26740	B8ZZC1:H7BZ65:Q53R	NP_001121637.1	Mouse:Fbln7|	Fibulin
Core matrisome	ECM Glycoproteins	FBN1	fibrillin 1	FBN|MASS|MF	3603	3603	F6U495:H0YN80:H0YN	NP_000129.3	Mouse:Fbn1|	NA
Core matrisome	ECM Glycoproteins	FBN2	fibrillin 2	CCA|DA9	3604	3604	D6REJ2:D6RJI3:E9PHW	NP_001990.2	Mouse:Fbn2|	NA
Core matrisome	ECM Glycoproteins	FGL1	fibrinogen-like 1	HFREP1|HP-04	3695	3695	Q08830	NP_004458.3:NP	Mouse:Fgl1|	NA
Core matrisome	ECM Glycoproteins	FGL2	fibrinogen-like 2	T49|pT49	3696	3696	Q14314	NP_006673.1	Mouse:Fgl2|	NA
Core matrisome	ECM Glycoproteins	FN1	fibronectin 1	CIG|DKFZp686	3778	3778	F8W7G7:H0Y4K8:H0Y7	NP_002017.1:NP	Mouse:Fn1|	NA
Core matrisome	ECM Glycoproteins	FNDC1	fibronectin type	AGS8|FNDC2|K	21184	21184	J3KNQ2:Q4ZHG4	NP_115921.2	Mouse:Fndc1|	NA
Core matrisome	ECM Glycoproteins	FNDC7	fibronectin type	FLJ35838|MGC	26668	26668	H7C2H6:J3KN65:Q5VT	NP_001138409.1	Mouse:Fndc7|	NA
Core matrisome	ECM Glycoproteins	FRAS1	Fraser syndrome	DKFZp686I051	19185	19185	D6RCP6:H0Y8V2:H0Y9	NP_001159605.1	Mouse:Fras1|	NA
Core matrisome	ECM Glycoproteins	GAS6	growth arrest-sp	AXLLG|AXSF|D	4168	4168	J3KP07:Q14393	NP_000811.1:NP	Mouse:Gas6|	NA
Core matrisome	ECM Glycoproteins	GLDN	gliomedin	CLOM|COLM|C	29514	29514	H0YM22:Q6ZMI3	NP_861454.2:XP	Mouse:Gldn|	NA
Core matrisome	ECM Glycoproteins	HMCN1	hemicentin 1	ARMD1|FBLN6	19194	19194	Q5TCP6:Q96RW7	NP_114141.2	Mouse:Hmcn1|	Fibulin

Core matrisome	ECM Glycoproteins	HMCN2	hemicentin 2	DKFZp434P021	21293	21293	A2A3K3:H0Y3X1:H7BZ	NP_001278744.	Mouse:Hmcn2|	NA
Core matrisome	ECM Glycoproteins	IBSP	integrin-binding	BNSP|BSP|BSP-	5341	5341	P21815	NP_004958.2	Mouse:Ibsp|	NA
Core matrisome	ECM Glycoproteins	IGFALS	insulin-like grow	ALS	5468	5468	H3BSX8:P35858	NP_001139478.	Mouse:Igfals|	Growth Factor-binding
Core matrisome	ECM Glycoproteins	IGFBP1	insulin-like grow	AFBP|IBP1|IGF-	5469	5469	C9J6H2:C9JXF9:P0883	NP_000587.1	Mouse:Igfbp1|	Growth Factor-binding
Core matrisome	ECM Glycoproteins	IGFBP2	insulin-like grow	IBP2|IGF-BP53	5471	5471	C9JMY1:C9JW52:H7C1	NP_000588.2	Mouse:Igfbp2|	Growth Factor-binding
Core matrisome	ECM Glycoproteins	IGFBP3	insulin-like grow	BP-53|IBP3	5472	5472	B3KWK7:C9JMX4:H0Y4	NP_000589.2:NP	Mouse:Igfbp3|	Growth Factor-binding
Core matrisome	ECM Glycoproteins	IGFBP4	insulin-like grow	BP-4|HT29-IGF	5473	5473	B4E351:P22692	NP_001543.2	Mouse:Igfbp4|	Growth Factor-binding
Core matrisome	ECM Glycoproteins	IGFBP5	insulin-like grow	IBP5	5474	5474	C9JXX4:P24593	NP_000590.1	Mouse:Igfbp5|	Growth Factor-binding
Core matrisome	ECM Glycoproteins	IGFBP6	insulin-like grow	IBP6	5475	5475	F8VVA5:F8VYK9:P2459	NP_002169.1	Mouse:Igfbp6|	Growth Factor-binding
Core matrisome	ECM Glycoproteins	IGFBP7	insulin-like grow	FSTL2|IGFBP-7	5476	5476	Q16270	NP_001240764.	Mouse:Igfbp7|	Growth Factor-binding
Core matrisome	ECM Glycoproteins	IGFBPL1	insulin-like grow	IGFBP-RP4|bA1	20081	20081	Q8WX77	NP_001007564.	Mouse:Igfbpl1|	Growth Factor-binding
Core matrisome	ECM Glycoproteins	IGSF10	immunoglobulin	CMF608|FLJ259	26384	26384	H7C4M2:Q6WRI0	NP_001171616.	Mouse:Igsf10|	NA
Core matrisome	ECM Glycoproteins	KCP	kielin/chordin-li	CRIM2|KCP1|N	17585	17585	Q6ZWJ8	NP_001129386.	Mouse:Kcp|	Growth Factor-binding
Core matrisome	ECM Glycoproteins	LAMA1	laminin, alpha 1	LAMA	6481	6481	P25391	NP_005550.2	Mouse:Lama1|	Laminin, Basement Membrane
Core matrisome	ECM Glycoproteins	LAMA2	laminin, alpha 2	LAMM	6482	6482	P24043	NP_000417.2:NP	Mouse:Lama2|	Laminin, Basement Membrane
Core matrisome	ECM Glycoproteins	LAMA3	laminin, alpha 3	BM600|E170|LA	6483	6483	K7EIP4:K7EMU9:K7EPP	NP_000218.2:NP	Mouse:Lama3|	Laminin, Basement Membrane
Core matrisome	ECM Glycoproteins	LAMA4	laminin, alpha 4	DKFZp686D231	6484	6484	E5RFD7:E5RFQ2:E5RHI	NP_001098676.2	Mouse:Lama4|	Laminin, Basement Membrane
Core matrisome	ECM Glycoproteins	LAMA5	laminin, alpha 5	KIAA1907	6485	6485	E2QRK0:F2Z2S4:H7C5J	NP_005551.3	Mouse:Lama5|	Laminin, Basement Membrane
Core matrisome	ECM Glycoproteins	LAMB1	laminin, beta 1	CLM|MGC1420	6486	6486	C9J296:E7EPA6:E9PCS	NP_002282.2	Mouse:Lamb1|	Laminin, Basement Membrane
Core matrisome	ECM Glycoproteins	LAMB2	laminin, beta 2 (	LAMS	6487	6487	F5H520:P55268	NP_002283.3:XP	Mouse:Lamb2|	Laminin, Basement Membrane
Core matrisome	ECM Glycoproteins	LAMB3	laminin, beta 3	BM600-125KD	6490	6490	Q13751:Q5THA1:X1W	NP_000219.2:NP	Mouse:Lamb3|	Laminin, Basement Membrane
Core matrisome	ECM Glycoproteins	LAMC1	laminin, gamma	LAMB2|MGC87	6492	6492	P11047:R4GNC7	NP_002284.3	Mouse:Lamc1|	Laminin, Basement Membrane
Core matrisome	ECM Glycoproteins	LAMC2	laminin, gamma	B2T|BM600|CSI	6493	6493	Q13753	NP_005553.2:NP	Mouse:Lamc2|	Laminin, Basement Membrane
Core matrisome	ECM Glycoproteins	LAMC3	laminin, gamma	DKFZp434E202	6494	6494	H7BY04:Q5JTC4:Q9Y6	NP_006050.3	Mouse:Lamc3|	Laminin, Basement Membrane
Core matrisome	ECM Glycoproteins	LGI4	leucine-rich rep	LGIL3	18712	18712	A8MVC2:K7ENQ0:Q8N	NP_644813.1	Mouse:Lgi4|	NA
Core matrisome	ECM Glycoproteins	LRG1	leucine-rich alph	HMFT1766|LRG	29480	29480	P02750	NP_443204.1	Mouse:Lrg1|	NA
Core matrisome	ECM Glycoproteins	LTBP1	latent transform	MGC163161	6714	6714	C9JD84:C9JDW2:E7EU	NP_000618.3:NP	Mouse:Ltbp1|	Growth Factor-binding
Core matrisome	ECM Glycoproteins	LTBP2	latent transform	C14orf141|GLC	6715	6715	G3V254:G3V3X5:G3V5	NP_000419.1	Mouse:Ltbp2|	Growth Factor-binding
Core matrisome	ECM Glycoproteins	LTBP3	latent transform	DKFZp586M21	6716	6716	E9PJR2:E9PKW1:E9PPT	NP_001123616.	Mouse:Ltbp3|	Growth Factor-binding
Core matrisome	ECM Glycoproteins	LTBP4	latent transform	FLJ46318|FLJ90	6717	6717	E7ENG9:E7EUU1:F5GX	NP_001036009.	Mouse:Ltbp4|	Growth Factor-binding
Core matrisome	ECM Glycoproteins	MATN1	matrilin 1, cartila	CMP|CRTM	6907	6907	P21941	NP_002370.1	Mouse:Matn1|	NA
Core matrisome	ECM Glycoproteins	MATN2	matrilin 2	-	6908	6908	E5RJM4:H0YBD5:H0YB	NP_002371.3:NP	Mouse:Matn2|	NA
Core matrisome	ECM Glycoproteins	MATN3	matrilin 3	DIPOA|EDM5|H	6909	6909	O15232	NP_002372.1	Mouse:Matn3|	NA
Core matrisome	ECM Glycoproteins	MATN4	matrilin 4	FLJ14417|HE6V	6910	6910	A6NNA4:O95460	NP_085080.1:NP	Mouse:Matn4|	NA
Core matrisome	ECM Glycoproteins	MEPE	matrix extracellu	OF45	13361	13361	D6RAC8:D6RFW6:Q9N	NP_001171623.	Mouse:Mepe|	NA
Core matrisome	ECM Glycoproteins	MFAP1	microfibrillar-as	-	7032	7032	P55081	NP_005917.2	Mouse:Mfap1a|M	NA
Core matrisome	ECM Glycoproteins	MFAP2	microfibrillar-as	FLJ50901|MAG	7033	7033	P55001	NP_001128719.	Mouse:Mfap2|	NA
Core matrisome	ECM Glycoproteins	MFAP3	microfibrillar-as	DKFZp586F021	7034	7034	E5RH26:E5RHQ6:E5RJ5	NP_001128509.	Mouse:Mfap3|	NA
Core matrisome	ECM Glycoproteins	MFAP4	microfibrillar-as	-	7035	7035	K7ES70:P55083	NP_001185624.	Mouse:Mfap4|	NA
Core matrisome	ECM Glycoproteins	MFAP5	microfibrillar ass	MAGP2|MP25	29673	29673	B3KW70:F5GYX4:F5H1	NP_003471.1:XP	Mouse:Mfap5|	NA
Core matrisome	ECM Glycoproteins	MFGE8	milk fat globule-	BA46|EDIL1|Hs	7036	7036	F5GZN3:F5H7N9:H0YK	NP_001108086.	Mouse:Mfge8|	NA
Core matrisome	ECM Glycoproteins	MGP	matrix Gla prote	GIG36|MGLAP|	7060	7060	H0YGZ6:P08493	NP_000891.2:NP	Mouse:Mgp|	NA
Core matrisome	ECM Glycoproteins	MMRN2	multimerin 2	EMILIN3|Endo	19888	19888	Q9H8L6:R4GMY6:V9G	NP_079032.2	Mouse:Mmrn2|	NA
Core matrisome	ECM Glycoproteins	MXRA5	matrix-remodell	DKFZp564I192	7539	7539	Q9NR99	NP_056234.2	NA	NA
Core matrisome	ECM Glycoproteins	NDNF	chromosome 4	FLJ23191|C4or	26256	26256	D6R972:D6RF18:Q8TB	NP_078850.3	Mouse:Ndnf|	NA
Core matrisome	ECM Glycoproteins	NELL2	NEL-like 2 (chick	NRP2	7751	7751	F8VRQ0:F8VVB6:F8VX	NP_001138579.	Mouse:Nell2|	NA
Core matrisome	ECM Glycoproteins	NID1	nidogen 1	NID	7821	7821	P14543	NP_002499.2	Mouse:Nid1|	Basement Membrane
Core matrisome	ECM Glycoproteins	NID2	nidogen 2 (oste	-	13389	13389	H0YJV3:Q14112	NP_031387.3	Mouse:Nid2|	Basement Membrane
Core matrisome	ECM Glycoproteins	NOV	nephroblastoma	CCN3|IGFBP9	7885	7885	P48745	NP_002505.1	Mouse:Nov|	CCN Family
Core matrisome	ECM Glycoproteins	NPNT	nephronectin	EGFL6L|POEM	27405	27405	D6RE63:D6RH31:H0Y8	NP_001028219.	Mouse:Npnt|	NA
Core matrisome	ECM Glycoproteins	NTN1	netrin 1	NTN1L	8029	8029	H7BZF4:O95631	NP_004813.2:XP	Mouse:Ntn1|	NA
Core matrisome	ECM Glycoproteins	NTN4	netrin 4	FLJ23180|PRO3	13658	13658	F8W0I7:Q9HB63	NP_067052.2	Mouse:Ntn4|	NA
Core matrisome	ECM Glycoproteins	NTN5	netrin 5	-	25208	25208	M0QXZ9:Q8WTR8	NP_665806.1:XP	Mouse:Ntn5|	NA
Core matrisome	ECM Glycoproteins	NTNG1	netrin G1	KIAA0976|Lmn	23319	23319	A6NK56:B4DKF0:F5GY	NP_001106697.	Mouse:Ntng1|	NA

Core matrisome	ECM Glycoproteins	NTNG2	netrin G2	KIAA0625|KIAA	14288	14288	A6NMX7:Q5JUJ3:Q96	NP_115925.2	Mouse:Ntng2|	NA
Core matrisome	ECM Glycoproteins	PAPLN	papilin, proteog	DKFZp434F053	19262	19262	B5MDP7:G3V5P6:H0Y	NP_775733.3	Mouse:Papln|	NA
Core matrisome	ECM Glycoproteins	PCOLCE	procollagen C-e	PCPE|PCPE1	8738	8738	Q15113	NP_002584.2	Mouse:Pcolce|	NA
Core matrisome	ECM Glycoproteins	PCOLCE2	procollagen C-e	PCPE2	8739	8739	C9JYX9:H7C520:H7C5	NP_037495.1	Mouse:Pcolce2|	NA
Core matrisome	ECM Glycoproteins	POMZP3	POM (POM121	MGC8359|POM	9203	9203	C9J370:C9JER0:H7C4D	NP_036362.3:NP	NA	NA
Core matrisome	ECM Glycoproteins	POSTN	periostin, osteob	MGC119510|M	16953	16953	B1ALD9:Q15063	NP_001129406.1	Mouse:Postn|	NA
Core matrisome	ECM Glycoproteins	PXDN	peroxidasin hom	D2S448|D2S44	14966	14966	C9J4I9:H7C1W1:H7C3	NP_036425.1	Mouse:Pxdn|	NA
Core matrisome	ECM Glycoproteins	RELN	reelin	PRO1598|RL	9957	9957	H7C2B0:J3KQ66:P785	NP_005036.2:NP	Mouse:Reln|	NA
Core matrisome	ECM Glycoproteins	RSPO1	R-spondin hom	CRISTIN3|FLJ4	21679	21679	Q2MKA7	NP_001033722.1	Mouse:Rspo1|	NA
Core matrisome	ECM Glycoproteins	RSPO2	R-spondin 2 hor	CRISTIN2|MGC	28583	28583	E5RGU9:E5RH25:E5RH	NP_001269792.1	Mouse:Rspo2|	NA
Core matrisome	ECM Glycoproteins	RSPO3	R-spondin 3 hor	CRISTIN1|FLJ14	20866	20866	Q9BXY4	NP_116173.2	Mouse:Rspo3|	NA
Core matrisome	ECM Glycoproteins	RSPO4	R-spondin famil	C20orf182|CRI	16175	16175	Q2I0M5	NP_001025042.2	Mouse:Rspo4|	NA
Core matrisome	ECM Glycoproteins	SBSPON	chromosome 8	FLJ40021|RPES	30362	30362	Q8IVN8	NP_694957.3	NA	NA
Core matrisome	ECM Glycoproteins	SLIT2	slit homolog 2 (	FLJ14420|SLIL3	11086	11086	E9PCX4:F5H0U4:H0Y9	NP_001276064.1	Mouse:Slit2|	NA
Core matrisome	ECM Glycoproteins	SLIT3	slit homolog 3 (	FLJ10764|MEG	11087	11087	O75094	NP_001258875.1	Mouse:Slit3|	NA
Core matrisome	ECM Glycoproteins	SMOC1	SPARC related m	-	20318	20318	Q9H4F8	NP_001030024.1	Mouse:Smoc1|	NA
Core matrisome	ECM Glycoproteins	SMOC2	SPARC related n	MST117|MSTP1	20323	20323	H0Y3J4:H0Y5I1:Q9H3U	NP_001159884.1	Mouse:Smoc2|	NA
Core matrisome	ECM Glycoproteins	SNED1	sushi, nidogen a	DKFZp586B242	24696	24696	B5MEF5:H7BYY9:H7C0	NP_001073906.1	Mouse:Sned1|	NA
Core matrisome	ECM Glycoproteins	SPARC	secreted protein	ON	11219	11219	E5RJA5:E5RK62:F5GY0	NP_003109.1	Mouse:Sparc|	NA
Core matrisome	ECM Glycoproteins	SPARCL1	SPARC-like 1 (h	PIG33|SC1	11220	11220	B4E2Z0:C9JJR8:D6RA2	NP_001121782.1	Mouse:Sparcl1|	NA
Core matrisome	ECM Glycoproteins	SPON1	spondin 1, extra	KIAA0762|MGC	11252	11252	Q9HCB6	NP_006099.2	Mouse:Spon1|	NA
Core matrisome	ECM Glycoproteins	SPON2	spondin 2, extra	DIL-1|DIL1|DK	11253	11253	D6RA41:D6RB12:D6RB	NP_001121797.1	Mouse:Spon2|	NA
Core matrisome	ECM Glycoproteins	SPP1	secreted phosph	BNSP|BSPI|ETA	11255	11255	D6R9C5:P10451	NP_000573.1:NP	Mouse:Spp1|	NA
Core matrisome	ECM Glycoproteins	SRPX	sushi-repeat-co	DRS|ETX1|SRP	11309	11309	P78539	NP_001164221.1	Mouse:Srpx|	NA
Core matrisome	ECM Glycoproteins	SRPX2	sushi-repeat-co	BPP|CBPS|PMG	30668	30668	O60687	NP_055282.1:XP	Mouse:Srpx2|	NA
Core matrisome	ECM Glycoproteins	SVEP1	sushi, von Willeb	C9orf13|CCP22	15985	15985	E9PBN8:Q4LDE5	NP_699197.3	Mouse:Svep1|	NA
Core matrisome	ECM Glycoproteins	TECTA	tectorin alpha	DFNA12|DFNA	11720	11720	O75443	NP_005413.2	Mouse:Tecta|	NA
Core matrisome	ECM Glycoproteins	TGFBI	transforming gr	BIGH3|CDB1|C	11771	11771	D6RBX4:G8JLA8:H0Y8	NP_000349.1	Mouse:Tgfbi|	NA
Core matrisome	ECM Glycoproteins	THBS1	thrombospondi	THBS|THBS-1|	11785	11785	A8MZG1:P07996	NP_003237.2	Mouse:Thbs1|	NA
Core matrisome	ECM Glycoproteins	THBS2	thrombospondi	TSP2	11786	11786	P35442:Q5RI53	NP_003238.2	Mouse:Thbs2|	NA
Core matrisome	ECM Glycoproteins	THBS3	thrombospondi	MGC119564|M	11787	11787	B4DQH6:F5H4Z8:P497	NP_001239536.1	Mouse:Thbs3|	NA
Core matrisome	ECM Glycoproteins	THBS4	thrombospondi	TSP4	11788	11788	E7ES19:P35443	NP_003239.2	Mouse:Thbs4|	NA
Core matrisome	ECM Glycoproteins	THSD4	thrombospondi	ADAMTSL6|FLJ	25835	25835	Q6ZMP0	NP_001273358.1	Mouse:Thsd4|	NA
Core matrisome	ECM Glycoproteins	TINAGL1	tubulointerstitial	ARG1|LCN7|LIE	19168	19168	B4DNS5:E7EUP2:Q9GZ	NP_001191343.1	Mouse:Tinagl1|	NA
Core matrisome	ECM Glycoproteins	TNC	tenascin C	150-225|GMEN	5318	5318	E9PC84:F5H5D6:F5H7	NP_002151.2:XP	Mouse:Tnc|	NA
Core matrisome	ECM Glycoproteins	TNFAIP6	tumor necrosis f	TSG-6|TSG6	11898	11898	P98066	NP_009046.2	Mouse:Tnfaip6|	NA
Core matrisome	ECM Glycoproteins	TNN	tenascin N	TN-W	22942	22942	Q9UQP3	NP_071376.1	Mouse:Tnn|	NA
Core matrisome	ECM Glycoproteins	TNR	tenascin R (restr	MGC149328|T	11953	11953	H0Y668:Q92752	NP_003276.3	Mouse:Tnr|	NA
Core matrisome	ECM Glycoproteins	TNXB	tenascin XB	HXBL|TENX|TN	11976	11976	C9J7W4:E7EPZ9:P221	NP_061978.6:NP	Mouse:Tnxb|	NA
Core matrisome	ECM Glycoproteins	TSKU	tsukushi small le	E2IG4|LRRC54|	28850	28850	E9PLG7:E9PN12:Q8W	NP_001245139.1	Mouse:Tsku|	NA
Core matrisome	ECM Glycoproteins	VIT	vitrin	DKFZp313L151	12697	12697	B5MD45:C9J6F5:H7C5	NP_001171440.1	Mouse:Vit|	NA
Core matrisome	ECM Glycoproteins	VTN	vitronectin	V75|VN|VNT	12724	12724	P04004	NP_000629.3	Mouse:Vtn|	Hemostasis
Core matrisome	ECM Glycoproteins	VWA1	von Willebrand	DKFZp761O05	30910	30910	J3QLP3:J3QRR0:Q6PC	NP_073745.2:NP	Mouse:Vwa1|	NA
Core matrisome	ECM Glycoproteins	VWA2	von Willebrand	AMACO|CCSP-	24709	24709	Q5GFL6	NP_001258975.1	Mouse:Vwa2|	NA
Core matrisome	ECM Glycoproteins	VWA3A	von Willebrand	FLJ40941|FLJ46	27088	27088	A6NCI4:H3BTG8:H3BT	NP_775886.3	Mouse:Vwa3a|	NA
Core matrisome	ECM Glycoproteins	VWA3B	von Willebrand	FLJ32686|MGC	28385	28385	B7Z7Q7:F8W737:F8W	NP_659429.4:XP	Mouse:Vwa3b|	NA
Core matrisome	ECM Glycoproteins	VWA5A	von Willebrand	BCSC-1|BCSC1	6658	6658	B4DHS6:O00534	NP_001123614.1	Mouse:AW551984	NA
Core matrisome	ECM Glycoproteins	VWA7	chromosome 6	FLJ25524|G7c|	13939	13939	E7EMA3:Q9Y334	NP_079534.2	Mouse:Vwa7|	NA
Core matrisome	ECM Glycoproteins	VWCE	von Willebrand	FLJ32009|URG1	26487	26487	B4DS56:B4DY31:Q96D	NP_689931.2	Mouse:Vwce|	NA
Core matrisome	ECM Glycoproteins	VWDE	von Willebrand	FLJ14712	21897	21897	E5RG96:J3KQJ9:Q8N2	NP_001129396.1	Mouse:Vwde|	NA
Core matrisome	ECM Glycoproteins	VWF	von Willebrand	F8VWF|VWD	12726	12726	I3L4K4:P04275:Q8TCE	NP_000543.2	Mouse:Vwf|	Hemostasis
Core matrisome	ECM Glycoproteins	WISP1	WNT1 inducible	CCN4|WISP1c|	12769	12769	E5RG88:O95388	NP_001191798.1	Mouse:Wisp1|	CCN Family
Core matrisome	ECM Glycoproteins	WISP2	WNT1 inducible	CCN5|CT58|CT	12770	12770	O76076:Q6PEG3	NP_003872.1:XP	Mouse:Wisp2|	CCN Family
Core matrisome	ECM Glycoproteins	WISP3	WNT1 inducible	CCN6|LIBC|MG	12771	12771	F8WC24:G3V0J1:O953	NP_003871.1:NP	Mouse:Wisp3|	CCN Family

Core matrisome	ECM Glycoproteins	ZP1	zona pellucida g	MGC87693	13187	13187	H0YG11:P60852	NP_997224.2	Mouse:Zp1|	NA
Core matrisome	ECM Glycoproteins	ZP3	zona pellucida g	ZP3A|ZP3B|ZP	13189	13189	E9PFI9:H0Y3M4:P2175	NP_001103824.1	Mouse:Zp3|	NA
Core matrisome	Collagens	COL10A1	collagen, type X	-	2185	2185	Q03692:Q5QPC7:Q5Q	NP_000484.2:XP	Mouse:Col10a1|	NA
Core matrisome	Collagens	COL11A1	collagen, type X	CO11A1|COLL	2186	2186	C9JMN2:H7C381:P121	NP_001177638.1	Mouse:Col11a1|	NA
Core matrisome	Collagens	COL11A2	collagen, type X	DFNA13|DFNB	2187	2187	A2AAS7:H0Y3B3:H0Y3	NP_001157243.1	Mouse:Col11a2|	NA
Core matrisome	Collagens	COL12A1	collagen, type X	BA209D8.1|CO	2188	2188	D6RGG3:H0Y4P7:H0Y5	NP_004361.3:NP	Mouse:Col12a1|	FACIT
Core matrisome	Collagens	COL13A1	collagen, type X	COLXIIIA1|FLJ4	2190	2190	E7ES46:E7ES47:E7ES49	NP_001123575.1	Mouse:Col13a1|	NA
Core matrisome	Collagens	COL14A1	collagen, type X	UND	2191	2191	H0YBB2:J3QT75:J3QT8	NP_066933.1:XP	Mouse:Col14a1|	FACIT
Core matrisome	Collagens	COL15A1	collagen, type X	FLJ38566	2192	2192	P39059	NP_001846.3	Mouse:Col15a1|	Basement Membrane
Core matrisome	Collagens	COL16A1	collagen, type X	447AA|FP1572	2193	2193	A6NCT7:A6NDR9:H7B	NP_001847.3	Mouse:Col16a1|	FACIT
Core matrisome	Collagens	COL17A1	collagen, type X	BA16H23.2|BP1	2194	2194	A2A2Y8:H0Y420:Q9UN	NP_000485.3	Mouse:Col17a1|	NA
Core matrisome	Collagens	COL18A1	collagen, type X	FLJ27325|FLJ34	2195	2195	H7BXV5:H7C457:P390	NP_085059.2:NP	Mouse:Col18a1|	Basement Membrane
Core matrisome	Collagens	COL19A1	collagen, type X	COL9A1L|D6S2	2196	2196	Q14993:Q5JVU1	NP_001849.2	Mouse:Col19a1|	FACIT
Core matrisome	Collagens	COL1A1	collagen, type I,	OI4	2197	2197	I3L3H7:P02452	NP_000079.2	Mouse:Col1a1|	NA
Core matrisome	Collagens	COL1A2	collagen, type I,	OI4	2198	2198	P08123	NP_000080.2	Mouse:Col1a2|	NA
Core matrisome	Collagens	COL20A1	collagen, type X	KIAA1510|bA2	14670	14670	B7ZBI4:B7ZBI5:Q9P21	NP_065933.2	Mouse:Col20a1|	FACIT
Core matrisome	Collagens	COL21A1	collagen, type X	COLA1L|DKFZ	17025	17025	A6PVD9:F5GZK2:H0Y4	NP_110447.2	NA	FACIT
Core matrisome	Collagens	COL22A1	collagen, type X	-	22989	22989	H0YAX7:Q8NFW1	NP_690848.1:XP	Mouse:Col22a1|	FACIT
Core matrisome	Collagens	COL23A1	collagen, type X	DKFZp434K062	22990	22990	Q86Y22	NP_775736.2	Mouse:Col23a1|	NA
Core matrisome	Collagens	COL24A1	collagen, type X	MGC142214	20821	20821	E9PNK8:F8WDM8:H0Y	NP_690850.2	Mouse:Col24a1|	NA
Core matrisome	Collagens	COL25A1	collagen, type X	CLAC|CLACP	18603	18603	A8MWQ5:D6R8Y2:E9P	NP_001243003.1	Mouse:Col25a1|	NA
Core matrisome	Collagens	COL26A1	EMI domain con	COL26A1|EMI6	18038	18038	C9JPW4:Q96A83	NP_001265492.1	Mouse:Col26a1|	NA
Core matrisome	Collagens	COL27A1	collagen, type X	FLJ11895|KIAA	22986	22986	H0YD40:Q5T1U7:Q8IZ	NP_116277.2	Mouse:Col27a1|	NA
Core matrisome	Collagens	COL28A1	collagen, type X	COL28	22442	22442	H7BZU0:H7C3P2:Q2U	NP_001032852.2	Mouse:Col28a1|	NA
Core matrisome	Collagens	COL2A1	collagen, type II,	ANFH|AOM|CC	2200	2200	P02458	NP_001835.3:NP	Mouse:Col2a1|	NA
Core matrisome	Collagens	COL3A1	collagen, type III	EDS4A|FLJ3453	2201	2201	E7ENY8:H7C435:P024	NP_000081.1	Mouse:Col3a1|	NA
Core matrisome	Collagens	COL4A1	collagen, type IV	arresten	2202	2202	F5H5K0:P02462	NP_001836.2	Mouse:Col4a1|	Basement Membrane
Core matrisome	Collagens	COL4A2	collagen, type IV	DKFZp686I142	2203	2203	A2A352:P08572	NP_001837.2	Mouse:Col4a2|	Basement Membrane
Core matrisome	Collagens	COL4A3	collagen, type IV	-	2204	2204	H7BXM4:Q01955	NP_000082.2	Mouse:Col4a3|	Basement Membrane
Core matrisome	Collagens	COL4A4	collagen, type IV	CA44	2206	2206	J3KNM7:P53420	NP_000083.3:XP	Mouse:Col4a4|	Basement Membrane
Core matrisome	Collagens	COL4A5	collagen, type IV	ASLN|ATS|CA5	2207	2207	H0Y998:H0Y9H0:H0Y9	NP_000486.1:NP	Mouse:Col4a5|	Basement Membrane
Core matrisome	Collagens	COL5A1	collagen, type V	-	2209	2209	H7BY82:P20908	NP_000084.3	Mouse:Col5a1|	NA
Core matrisome	Collagens	COL5A2	collagen, type V	MGC105115	2210	2210	P05997	NP_000384.2	Mouse:Col5a2|	NA
Core matrisome	Collagens	COL6A1	collagen, type V	OPLL	2211	2211	P12109	NP_001839.2	Mouse:Col6a1|	NA
Core matrisome	Collagens	COL6A2	collagen, type V	DKFZp586E132	2212	2212	C9JH44:H7C0M5:P121	NP_001840.3:NP	Mouse:Col6a2|	NA
Core matrisome	Collagens	COL6A3	collagen, type V	DKFZp686D231	2213	2213	C9JNG9:E7ENL6:E9PC	NP_004360.2:NP	Mouse:Col6a3|	NA
Core matrisome	Collagens	COL6A6	collagen, type V	-	27023	27023	A6NMZ7:F8W6Y7:H0Y	NP_001096078.1	Mouse:Col6a6|	NA
Core matrisome	Collagens	COL7A1	collagen, type V	EBD1|EBDCT|E	2214	2214	C9JBL3:Q02388	NP_000085.1	Mouse:Col7a1|	NA
Core matrisome	Collagens	COL8A1	collagen, type V	C3orf7|MGC95	2215	2215	C9JTN9:P27658	NP_001841.2:NP	Mouse:Col8a1|	NA
Core matrisome	Collagens	COL8A2	collagen, type V	FECD|FECD1|FL	2216	2216	E9PP49:P25067	NP_005193.1:XP	Mouse:Col8a2|	NA
Core matrisome	Collagens	COL9A1	collagen, type IX	DJ149L1.1.2|ED	2217	2217	A6NEQ6:P20849	NP_001842.3:NP	Mouse:Col9a1|	FACIT
Core matrisome	Collagens	COL9A2	collagen, type IX	DJ39G22.4|ED	2218	2218	B1AKJ1:B1AKJ3:H0Y40	NP_001843.1:XP	Mouse:Col9a2|	FACIT
Core matrisome	Collagens	COL9A3	collagen, type IX	DJ885L7.4.1|ED	2219	2219	Q14050:Q4VXW1	NP_001844.3	Mouse:Col9a3|	FACIT
Core matrisome	Proteoglycans	ACAN	aggrecan	AGC1|AGCAN|	319	319	E7ENV9:E7EX88:H0YK	NP_001126.3:NP	Mouse:Acan|	NA
Core matrisome	Proteoglycans	ASPN	asporin	FLJ20129|OS3|	14872	14872	C9JGC9:Q05CQ6:Q5T	NP_001180264.1	Mouse:Aspn|	NA
Core matrisome	Proteoglycans	BCAN	brevican	BEHAB|CSPG7|	23059	23059	Q5T3I6:Q5T3I7:Q5T3I	NP_068767.3:NP	Mouse:Bcan|	NA
Core matrisome	Proteoglycans	BGN	biglycan	DSPG1|PG-S1|	1044	1044	C9JKG1:P21810	NP_001702.1	Mouse:Bgn|	NA
Core matrisome	Proteoglycans	CHAD	chondroadherin	SLRR4A	1909	1909	H0YA03:O15335	NP_001258.2	Mouse:Chad|	NA
Core matrisome	Proteoglycans	CHADL	chondroadherin	SLRR4B	25165	25165	B0QYT2:H0Y4I5:Q6NU	NP_612490.1	Mouse:Chadl|	NA
Core matrisome	Proteoglycans	DCN	decorin	CSCD|DSPG2|P	2705	2705	F8VNV6:F8VNW0:F8V	NP_001911.1:NP	Mouse:Dcn|	NA
Core matrisome	Proteoglycans	EPYC	epiphycan	DSPG3|PGLB|P	3053	3053	F8VSI4:Q99645	NP_004941.2	Mouse:Epyc|	NA
Core matrisome	Proteoglycans	ESM1	endothelial cell-	endocan	3466	3466	M0R154:Q9NQ30	NP_001129076.1	Mouse:Esm1|	NA
Core matrisome	Proteoglycans	FMOD	fibromodulin	SLRR2E	3774	3774	Q06828	NP_002014.2	Mouse:Fmod|	NA
Core matrisome	Proteoglycans	HAPLN1	hyaluronan and	CRTL1	2380	2380	D6RAK7:D6RBS1:D6RB	NP_001875.1	Mouse:Hapln1|	NA

Core matrisome	Proteoglycans	HAPLN2	hyaluronan and	BRAL1	17410	17410	Q5T3J1:Q9GZV7	NP_068589.1:XP	Mouse:Hapln2|	NA
Core matrisome	Proteoglycans	HAPLN3	hyaluronan and	EXLD1|HsT1988	21446	21446	H3BRY4:H3BT26:H3BT	NP_839946.1	Mouse:Hapln3|	NA
Core matrisome	Proteoglycans	HSPG2	heparan sulfate	PLC|PRCAN|SJA	5273	5273	H0Y5A9:H7BYA5:H7C4	NP_001278789.1	Mouse:Hspg2|	Basement Membrane
Core matrisome	Proteoglycans	IMPG1	interphotorecep	GP147|IPM150	6055	6055	Q17R60:Q5JSC4:Q68D	NP_001269297.1	Mouse:Impg1|	NA
Core matrisome	Proteoglycans	IMPG2	interphotorecep	IPM200|SPACR	18362	18362	Q9BZV3	NP_057331.2	Mouse:Impg2|	NA
Core matrisome	Proteoglycans	KERA	keratocan	CNA2|SLRR2B	6309	6309	O60938	NP_008966.1	Mouse:Kera|	NA
Core matrisome	Proteoglycans	LUM	lumican	LDC|SLRR2D	6724	6724	P51884	NP_002336.1	Mouse:Lum|	NA
Core matrisome	Proteoglycans	OGN	osteoglycin	DKFZp586P242	8126	8126	P20774:Q5TBF5	NP_054776.1:NP	Mouse:Ogn|	NA
Core matrisome	Proteoglycans	OMD	osteomodulin	OSAD|SLRR2C	8134	8134	Q99983	NP_005005.1	Mouse:Omd|	NA
Core matrisome	Proteoglycans	OPTC	opticin	OPT	8158	8158	Q5T2G3:Q9UBM4	NP_055174.1	Mouse:Optc|	NA
Core matrisome	Proteoglycans	PODN	podocan	MGC24995|PCA	23174	23174	Q7Z5L7	NP_001186009.1	Mouse:Podn|	NA
Core matrisome	Proteoglycans	PODNL1	podocan-like 1	FLJ23447|SLRR	26275	26275	B7Z3M0:K7EJ07:K7EMY	NP_001139726.1	Mouse:Podnl1|	NA
Core matrisome	Proteoglycans	PRELP	proline/arginine	MGC45323|MS	9357	9357	P51888	NP_002716.1:NP	Mouse:Prelp|	NA
Core matrisome	Proteoglycans	PRG4	p53-responsive	-	9364	9364	E7EQ48:E9PLR3:J3KP74	NP_001121180.1	Mouse:Prg4|	NA
Core matrisome	Proteoglycans	SPOCK1	sparc/osteonect	FLJ37170|SPOC	11251	11251	D6RAM7:D6RB21:D6R	NP_004589.1	Mouse:Spock1|	NA
Core matrisome	Proteoglycans	SPOCK2	sparc/osteonect	FLJ97039|testic	13564	13564	F5H283:Q92563	NP_001127906.1	Mouse:Spock2|	NA
Core matrisome	Proteoglycans	SPOCK3	sparc/osteonect	HSAJ1454|TES-	13565	13565	B4DG04:E7EMP8:E7EN	NP_001035249.1	Mouse:Spock3|	NA
Core matrisome	Proteoglycans	SRGN	serglycin	FLJ12930|MGC	9361	9361	P10124	NP_002718.2	Mouse:Srgn|	NA
Core matrisome	Proteoglycans	VCAN	versican	CSPG2|DKFZp6	2464	2464	D6RGZ6:E9PF17:P1361	NP_001119808.1	Mouse:Vcan|	NA
Matrisome-associated	ECM-affiliated Proteins	ANXA1	annexin A1	ANX1|LPC1	533	533	P04083:Q5T3N0:Q5T3	NP_000691.1	Mouse:Anxa1|	Annexin
Matrisome-associated	ECM-affiliated Proteins	ANXA11	annexin A11	ANX11|CAP50	535	535	H0Y6E1:P50995:Q5T0	NP_001148.1:NP	Mouse:Anxa11|	Annexin
Matrisome-associated	ECM-affiliated Proteins	ANXA2	annexin A2	ANX2|ANX2L4|	537	537	H0YKL9:H0YKN4:H0YK	NP_001002857.1	Mouse:Anxa2|	Annexin
Matrisome-associated	ECM-affiliated Proteins	ANXA4	annexin A4	ANX4|DKFZp68	542	542	B4DDF9:P09525:Q6P4	NP_001144.1	Mouse:Anxa4|	Annexin
Matrisome-associated	ECM-affiliated Proteins	ANXA5	annexin A5	ANX5|ENX2|PP	543	543	D6RBE9:D6RBL5:D6RC	NP_001145.1	Mouse:Anxa5|	Annexin
Matrisome-associated	ECM-affiliated Proteins	ANXA6	annexin A6	ANX6|CBP68	544	544	A6NN80:E5RFF0:E5RI0	NP_001146.2:NP	Mouse:Anxa6|	Annexin
Matrisome-associated	ECM-affiliated Proteins	ANXA7	annexin A7	ANX7|SNX|SYN	545	545	B4DT77:B9ZVT2:P2007	NP_001147.1:NP	Mouse:Anxa7|	Annexin
Matrisome-associated	ECM-affiliated Proteins	ANXA8	annexin A8	ANX8|FLJ53095	546	546	B4DTC9:P13928:Q5VT	NP_001035173.1	Mouse:Anxa8|	Annexin
Matrisome-associated	ECM-affiliated Proteins	ANXA8L1	annexin A8-like	bA301J7.3	23334	23334	F5H7X5:Q5T2P7:Q5T2	NP_001258632.1	NA	Annexin
Matrisome-associated	ECM-affiliated Proteins	ANXA9	annexin A9	ANX31	547	547	O76027	NP_003559.2	Mouse:Anxa9|	Annexin
Matrisome-associated	ECM-affiliated Proteins	C1QL1	complement cor	C1QRF|CRF	24182	24182	O75973	NP_006679.1	Mouse:C1ql1|	NA
Matrisome-associated	ECM-affiliated Proteins	C1QL2	complement cor	C1QTNF10|CTF	24181	24181	Q7Z5L3	NP_872334.2	Mouse:C1ql2|	NA
Matrisome-associated	ECM-affiliated Proteins	C1QL3	complement cor	C1QTNF13|C1q	19359	19359	Q5VWW1	NP_001010908.1	Mouse:C1ql3|	NA
Matrisome-associated	ECM-affiliated Proteins	C1QL4	complement cor	C1QTNF11|MG	31416	31416	Q86Z23	NP_001008224.1	Mouse:C1ql4|	NA
Matrisome-associated	ECM-affiliated Proteins	C1QTNF1	C1q and tumor	CTRP1|FLJ9069	14324	14324	Q6ZMH6:Q9BXJ1	NP_112230.1:NP	Mouse:C1qtnf1|	NA
Matrisome-associated	ECM-affiliated Proteins	C1QTNF2	C1q and tumor	CTRP2|zacrp2	14325	14325	Q9BXJ5	NP_114114.2	Mouse:C1qtnf2|	NA
Matrisome-associated	ECM-affiliated Proteins	C1QTNF3	C1q and tumor	C1ATNF3|COR	14326	14326	Q9BXJ4	NP_112207.1:NP	Mouse:C1qtnf3|	NA
Matrisome-associated	ECM-affiliated Proteins	C1QTNF4	C1q and tumor	CTRP4|ZACRP4	14346	14346	E9PPZ5:Q9BXJ3	NP_114115.2	Mouse:C1qtnf4|	NA
Matrisome-associated	ECM-affiliated Proteins	C1QTNF6	C1q and tumor	CTRP6|ZACRP6	14343	14343	F8WC87:Q9BXI9	NP_114116.3:NP	Mouse:C1qtnf6|	NA
Matrisome-associated	ECM-affiliated Proteins	C1QTNF7	C1q and tumor	CTRP7|ZACRP7	14342	14342	J3KPK0:Q9BXJ2	NP_001128642.1	Mouse:C1qtnf7|	NA
Matrisome-associated	ECM-affiliated Proteins	C1QTNF8	C1q and tumor	CTRP8|UNQ58	31374	31374	P60827	NP_997302.2	NA	NA
Matrisome-associated	ECM-affiliated Proteins	C1QTNF9	C1q and tumor	C1QTNF9A|CTF	28732	28732	P0C862	NP_848635.2	Mouse:C1qtnf9|	NA
Matrisome-associated	ECM-affiliated Proteins	CLEC11A	C-type lectin do	CLECSF3|LSLCL	10576	10576	M0R081:Q9Y240	NP_002966.1	Mouse:Clec11a|	NA
Matrisome-associated	ECM-affiliated Proteins	CLEC18A	C-type lectin do	CLEC18C|FLJ77	30388	30388	A5D8T8:F8W692:H3BN	NP_001129686.1	Mouse:Clec18a|	NA
Matrisome-associated	ECM-affiliated Proteins	CLEC18B	C-type lectin do	MRCL2	33849	33849	Q6UXF7	NP_001011880.2	Mouse:Clec18a|	NA
Matrisome-associated	ECM-affiliated Proteins	CLEC18C	C-type lectin do	MGC34761|MR	28538	28538	F5H550:H3BSF2:H3BU	NP_775890.2	Mouse:Clec18a|	NA
Matrisome-associated	ECM-affiliated Proteins	CLEC2B	C-type lectin do	AICL|CLECSF2|	2053	2053	F5H631:Q92478	NP_005118.2	Mouse:Clec2d|Mo	NA
Matrisome-associated	ECM-affiliated Proteins	CLEC2D	C-type lectin do	CLAX|LLT1|OCI	14351	14351	F5H0P0:F5H5U4:F8WF	NP_001004419.1	Mouse:Clec2d|Mo	NA
Matrisome-associated	ECM-affiliated Proteins	CLEC3A	C-type lectin do	CLECSF1|MGC1	2052	2052	H3BTK1:J3KNC9:O755	NP_005743.4	Mouse:Clec3a|	NA
Matrisome-associated	ECM-affiliated Proteins	CLEC3B	C-type lectin do	DKFZp686H17	11891	11891	E9PHK0:P05452	NP_003269.2:XP	Mouse:Clec3b|	NA
Matrisome-associated	ECM-affiliated Proteins	CLEC4A	C-type lectin do	CLECSF6|DCIR|	13257	13257	H0YGF9:Q9UMR7	NP_057268.1:NP	Mouse:Clec4a1|M	NA
Matrisome-associated	ECM-affiliated Proteins	CLEC5A	C-type lectin do	CLECSF5|MDL-	2054	2054	C9JCE4:C9JPR7:F8WCL	NP_037384.1:XP	Mouse:Clec5a|	NA
Matrisome-associated	ECM-affiliated Proteins	COLEC10	collectin sub-far	CLL1|MGC1187	2220	2220	Q9Y6Z7	NP_006429.2	Mouse:Colec10|	NA
Matrisome-associated	ECM-affiliated Proteins	COLEC11	collectin sub-far	CL-K1-I|CL-K1	17213	17213	C9JWT5:F8WB29:Q9BV	NP_001242911.1	Mouse:Colec11|	NA
Matrisome-associated	ECM-affiliated Proteins	COLEC12	collectin sub-far	CLP1|NSR2|SCA	16016	16016	Q5KU26	NP_569057.1	Mouse:Colec12|	NA

Matrisome-associate	ECM-affiliated Proteins	CSPG4	chondroitin sulfa	HMW-MAA|M	2466	2466	Q6UVK1	NP_001888.2	Mouse:Cspg4|	NA
Matrisome-associate	ECM-affiliated Proteins	CSPG5	chondroitin sulfa	MGC44034|NG	2467	2467	O95196	NP_001193872.1	Mouse:Cspg5|	NA
Matrisome-associate	ECM-affiliated Proteins	EMCN	endomucin	EMCN2|MUC14	16041	16041	D6RHW5:H0YAA7:Q9U	NP_001153166.1	Mouse:Emcn|	NA
Matrisome-associate	ECM-affiliated Proteins	FCN1	ficolin (collagen/	FCNM	3623	3623	O00602	NP_001994.2	Mouse:Fcna|	NA
Matrisome-associate	ECM-affiliated Proteins	FCN2	ficolin (collagen/	EBP-37|FCNL|P	3624	3624	Q15485	NP_004099.2:NP	Mouse:Fcnb|	NA
Matrisome-associate	ECM-affiliated Proteins	FREM1	FRAS1 related ex	BNAR|C9orf143	23399	23399	C9JT55:F8WE85:Q5H8	NP_001171175.1	Mouse:Frem1|	NA
Matrisome-associate	ECM-affiliated Proteins	FREM3	FRAS1 related ex	-	25172	25172	P0C091	NP_001161707.1	Mouse:Frem3|	NA
Matrisome-associate	ECM-affiliated Proteins	GPC1	glypican 1	FLJ38078|glypi	4449	4449	C9J4Y6:H7BZE9:H7BZL	NP_002072.2	Mouse:Gpc1|	Glypican
Matrisome-associate	ECM-affiliated Proteins	GPC2	glypican 2	DKFZp547M10	4450	4450	Q8N158	NP_689955.1	Mouse:Gpc2|	Glypican
Matrisome-associate	ECM-affiliated Proteins	GPC5	glypican 5	-	4453	4453	P78333	NP_004457.1	Mouse:Gpc5|	Glypican
Matrisome-associate	ECM-affiliated Proteins	GPC6	glypican 6	MGC126288|O	4454	4454	Q9Y625	NP_005699.1	Mouse:Gpc6|	NA
Matrisome-associate	ECM-affiliated Proteins	GREM1	gremlin 1, cystei	CKTSF1B1|DAN	2001	2001	H0YLY2:O60565	NP_001178252.1	Mouse:Grem1|	NA
Matrisome-associate	ECM-affiliated Proteins	HPX	hemopexin	FLJ56652|HX	5171	5171	P02790	NP_000604.1:XP	Mouse:Hpx|	NA
Matrisome-associate	ECM-affiliated Proteins	LGALS1	lectin, galactosid	DKFZp686E231	6561	6561	F8WCQ5:F8WEI7:P093	NP_002296.1	Mouse:Lgals1|	Galectin
Matrisome-associate	ECM-affiliated Proteins	LGALS12	lectin, galactosid	GALECTIN-12|	15788	15788	G5E970:Q96DT0	NP_001136007.1	Mouse:Lgals12|	Galectin
Matrisome-associate	ECM-affiliated Proteins	LGALS2	lectin, galactosid	HL14|MGC750	6562	6562	B0QYC9:P05162	NP_006489.1	Mouse:Lgals2|	Galectin
Matrisome-associate	ECM-affiliated Proteins	LGALS3	lectin, galactosid	CBP35|GAL3|G	6563	6563	G3V3R6:G3V407:P179	NP_002297.2	Mouse:Lgals3|	Galectin
Matrisome-associate	ECM-affiliated Proteins	LGALS4	lectin, galactosid	GAL4|L36LBP	6565	6565	M0QZ93:M0R1B2:M0R	NP_006140.1	Mouse:Lgals4|	Galectin
Matrisome-associate	ECM-affiliated Proteins	LGALS7	lectin, galactosid	GAL7|LGALS7A	6568	6568	M0R281:P47929	NP_001035972.1	Mouse:Lgals7|	Galectin
Matrisome-associate	ECM-affiliated Proteins	LGALS8	lectin, galactosid	Gal-8|PCTA-1|	6569	6569	B1ANM0:E9PJ77:E9PN	NP_006490.3:NP	Mouse:Lgals8|	Galectin
Matrisome-associate	ECM-affiliated Proteins	LGALS9	lectin, galactosid	HUAT|LGALS9A	6570	6570	B4DWP7:F8W9W4:J3K	NP_002299.2:NP	Mouse:Lgals9|	Galectin
Matrisome-associate	ECM-affiliated Proteins	LMAN1	lectin, mannose-	ERGIC-53|ERGI	6631	6631	P49257	NP_005561.1	Mouse:Lman1|	NA
Matrisome-associate	ECM-affiliated Proteins	MUC1	mucin 1, cell sur	CD227|EMA|H2	7508	7508	B1AVQ5:B1AVQ7:P15	NP_001018016.1	Mouse:Muc1|	Mucin
Matrisome-associate	ECM-affiliated Proteins	MUC12	mucin 12, cell su	MUC11	7510	7510	F5GWV9:H7BXN1:Q9U	NP_001157934.1	Mouse:Muc12|	Mucin
Matrisome-associate	ECM-affiliated Proteins	MUC15	mucin 15, cell su	PAS3|PASIII	14956	14956	E9PII6:F8W945:Q8N38	NP_001128563.1	Mouse:Muc15|	Mucin
Matrisome-associate	ECM-affiliated Proteins	MUC17	mucin 17, cell su	MUC3	16800	16800	E7EPM4:Q685J3	NP_001035194.1	NA	Mucin
Matrisome-associate	ECM-affiliated Proteins	MUC20	mucin 20, cell su	FLJ14408|FLJ53	23282	23282	C9JJE7:E9PH32:H7C10	NP_001269435.1	Mouse:Muc20|	Mucin
Matrisome-associate	ECM-affiliated Proteins	MUC4	mucin 4, cell sur	HSA276359	7514	7514	E7ENC5:E7EQG8:E7EQ	NP_004523.3:NP	Mouse:Muc4|	Mucin
Matrisome-associate	ECM-affiliated Proteins	MUCL1	mucin-like 1	SBEM	30588	30588	F8VV13:Q96DR8	NP_477521.1	Mouse:Mucl1|	Mucin
Matrisome-associate	ECM-affiliated Proteins	OVGP1	oviductal glycop	CHIT5|EGP|MU	8524	8524	B4DHP1:Q12889	NP_002548.3	Mouse:Ovgp1|	NA
Matrisome-associate	ECM-affiliated Proteins	PARM1	prostate androg	Cipar1|DKFZP5	24536	24536	D6RBB6:Q6UWI2	NP_056208.2	Mouse:Parm1|	NA
Matrisome-associate	ECM-affiliated Proteins	PLXDC1	plexin domain c	DKFZp686F093	20945	20945	B3KSI0:B4E173:C9JCL2	NP_065138.2	Mouse:Plxdc1|	NA
Matrisome-associate	ECM-affiliated Proteins	PLXDC2	plexin domain c	FLJ14623|TEM7	21013	21013	Q6UX71	NP_001269665.1	Mouse:Plxdc2|	NA
Matrisome-associate	ECM-affiliated Proteins	PLXNA1	plexin A1	DKFZp761P191	9099	9099	F8VSZ4:Q9UIW2	NP_115618.3	Mouse:Plxna1|	Plexin
Matrisome-associate	ECM-affiliated Proteins	PLXNA2	plexin A2	FLJ11751|FLJ30	9100	9100	O75051	NP_079455.3	Mouse:Plxna2|	Plexin
Matrisome-associate	ECM-affiliated Proteins	PLXNA3	plexin A3	6.3|HSSEXGENI	9101	9101	P51805	NP_059984.3	Mouse:Plxna3|	Plexin
Matrisome-associate	ECM-affiliated Proteins	PLXNA4	plexin A4	DKFZp434G06	9102	9102	Q9HCM2	NP_001099013.1	Mouse:Plxna4|	Plexin
Matrisome-associate	ECM-affiliated Proteins	PLXNB1	plexin B1	KIAA0407|MGC	9103	9103	G8JLJ7:O43157	NP_001123554.1	Mouse:Plxnb1|	Plexin
Matrisome-associate	ECM-affiliated Proteins	PLXNB2	plexin B2	KIAA0315|MM1	9104	9104	A6QRG9:A6QRH1:E2P	NP_036533.2:XP	Mouse:Plxnb2|	Plexin
Matrisome-associate	ECM-affiliated Proteins	PLXNC1	plexin C1	CD232|PLXN-C	9106	9106	B4DHQ7:F5H3A2:F8VU	NP_005752.1	Mouse:Plxnc1|	Plexin
Matrisome-associate	ECM-affiliated Proteins	PLXND1	plexin D1	KIAA0620|MGC	9107	9107	D6RH25:H0Y9D1:H0YA	NP_055918.2	Mouse:Plxnd1|	Plexin
Matrisome-associate	ECM-affiliated Proteins	SDC1	syndecan 1	CD138|SDC|SY	10658	10658	E9PHH3:F8WCX9:H7C	NP_001006947.1	Mouse:Sdc1|	Syndecan
Matrisome-associate	ECM-affiliated Proteins	SDC2	syndecan 2	HSPG|HSPG1|S	10659	10659	E5RHU3:E5RJB8:E7ESK	NP_002989.2:XP	Mouse:Sdc2|	Syndecan
Matrisome-associate	ECM-affiliated Proteins	SDC3	syndecan 3	SDCN|SYND3	10660	10660	D3DPN2:O75056	NP_055469.3	Mouse:Sdc3|	Syndecan
Matrisome-associate	ECM-affiliated Proteins	SDC4	syndecan 4	MGC22217|SYN	10661	10661	B4E1S6:P31431	NP_002990.2	Mouse:Sdc4|	Syndecan
Matrisome-associate	ECM-affiliated Proteins	SEMA3A	sema domain, in	Hsema-I|Hsem	10723	10723	C9J9C4:C9JD25:Q1456	NP_006071.1:XP	Mouse:Sema3a|	Semaphorin
Matrisome-associate	ECM-affiliated Proteins	SEMA3B	sema domain, in	FLJ34863|LUCA	10724	10724	Q13214	NP_001005914.1	Mouse:Sema3b|	Semaphorin
Matrisome-associate	ECM-affiliated Proteins	SEMA3C	sema domain, in	SEMAE|SemE	10725	10725	B4DRL8:F2Z2Y0:F5H1Z	NP_006370.1	Mouse:Sema3c|	Semaphorin
Matrisome-associate	ECM-affiliated Proteins	SEMA3D	sema domain, in	MGC39708|Ser	10726	10726	C9JYT6:O95025	NP_689967.2	Mouse:Sema3d|	Semaphorin
Matrisome-associate	ECM-affiliated Proteins	SEMA3E	sema domain, in	KIAA0331|M-S	10727	10727	C9JVH5:F8WCZ5:O150	NP_001171600.1	Mouse:Sema3e|	Semaphorin
Matrisome-associate	ECM-affiliated Proteins	SEMA3G	sema domain, in	FLJ00014|MGC	30400	30400	C9JXB2:H7C5M7:Q9N	NP_064548.1	Mouse:Sema3g|	Semaphorin
Matrisome-associate	ECM-affiliated Proteins	SEMA4A	sema domain, in	CORD10|FLJ12	10729	10729	Q5TCI4:Q5TCI6:Q5TCJ	NP_001180229.1	Mouse:Sema4a|	Semaphorin
Matrisome-associate	ECM-affiliated Proteins	SEMA4B	sema domain, in	KIAA1745|MGC	10730	10730	H0YKP2:H0YKV7:H0YL	NP_064595.2:NP	Mouse:Sema4b|	Semaphorin
Matrisome-associate	ECM-affiliated Proteins	SEMA4C	sema domain, in	FLJ20369|KIAA	10731	10731	C9J4M7:C9JV89:Q9C0	NP_060259.4	Mouse:Sema4c|	Semaphorin

Matrisome-associate	ECM-affiliated Proteins	SEMA4D	sema domain, in	C9orf164|CD10	10732	10732	C9JD54:C9JFP1:C9JYS	NP_001135759.1	Mouse:Sema4d|	Semaphorin
Matrisome-associate	ECM-affiliated Proteins	SEMA4F	sema domain, in	M-SEMA|PRO2	10734	10734	B7Z931:C9JKW7:C9K0	NP_001258590.1	Mouse:Sema4f|	Semaphorin
Matrisome-associate	ECM-affiliated Proteins	SEMA4G	sema domain, in	FLJ20590|KIAA	10735	10735	E5RFG2:E5RGX4:H0YB	NP_001190173.1	Mouse:Sema4g|	Semaphorin
Matrisome-associate	ECM-affiliated Proteins	SEMA5A	sema domain, se	FLJ12815|SEMA	10736	10736	D6R9K6:D6RAF4:H0Y9	NP_003957.2:XP	Mouse:Sema5a|	Semaphorin
Matrisome-associate	ECM-affiliated Proteins	SEMA5B	sema domain, se	FLJ10372|KIAA	10737	10737	B5ME80:C9JKR3:C9JTV	NP_001026872.2	Mouse:Sema5b|	Semaphorin
Matrisome-associate	ECM-affiliated Proteins	SEMA6A	sema domain, tr	HT018|KIAA13	10738	10738	B3KU01:D6RAG9:D6R	NP_065847.1	Mouse:Sema6a|	Semaphorin
Matrisome-associate	ECM-affiliated Proteins	SEMA6B	sema domain, tr	SEM-SEMA-Y|	10739	10739	Q9H3T3	NP_115484.2	Mouse:Sema6b|	Semaphorin
Matrisome-associate	ECM-affiliated Proteins	SEMA6C	sema domain, tr	SEMAY|m-Sem	10740	10740	Q9H3T2	NP_001171532.1	Mouse:Sema6c|	Semaphorin
Matrisome-associate	ECM-affiliated Proteins	SEMA6D	sema domain, tr	FLJ11598|KIAA	16770	16770	H0YL82:H0YM97:H0YN	NP_001185928.1	Mouse:Sema6d|	Semaphorin
Matrisome-associate	ECM-affiliated Proteins	SEMA7A	semaphorin 7A,	CD108|CDw10	10741	10741	F5GYX3:H3BMF9:O75	NP_001139501.1	Mouse:Sema7a|	Semaphorin
Matrisome-associate	ECM-affiliated Proteins	SFTA2	surfactant assoc	GSGL541|SFTP	18386	18386	Q6UW10	NP_995326.1	Mouse:Sfta2|	NA
Matrisome-associate	ECM-affiliated Proteins	SFTA3	surfactant assoc	FLJ23494|SFTP	18387	18387	E5RG42:G5EA12:P0C7	NP_001094811.1	Mouse:E030019B1	NA
Matrisome-associate	ECM-affiliated Proteins	SFTPD	surfactant prote	COLEC7|PSP-D	10803	10803	P35247:Q5T0M2	NP_003010.4	Mouse:Sftpd|	NA
Matrisome-associate	ECM Regulators	A2M	alpha-2-macrog	CPAMD5|DKFZ	7	7	F5H1E8:F8W7L3:H0YF	NP_000005.2	Mouse:A2m|	NA
Matrisome-associate	ECM Regulators	A2ML1	alpha-2-macrog	CPAMD9|DKFZ	23336	23336	A8K2U0:F5GXP1:F5GY	NP_653271.2	NA	NA
Matrisome-associate	ECM Regulators	ADAM10	ADAM metallop	AD10|CD156c|	188	188	B4DU28:B5MC71:C9J9	NP_001101.1	Mouse:Adam10|	NA
Matrisome-associate	ECM Regulators	ADAM11	ADAM metallop	MDC	189	189	B4DKD2:H7BY08:K7EK	NP_002381.2	Mouse:Adam11|	NA
Matrisome-associate	ECM Regulators	ADAM12	ADAM metallop	MCMP|MCMP	190	190	O43184:Q5JRP2	NP_001275903.1	Mouse:Adam12|	NA
Matrisome-associate	ECM Regulators	ADAM15	ADAM metallop	MDC15	193	193	A6NG98:Q13444	NP_001248393.1	Mouse:Adam15|	NA
Matrisome-associate	ECM Regulators	ADAM17	ADAM metallop	ADAM18|CD15	195	195	E7EUI5:P78536	NP_003174.3	Mouse:Adam17|	NA
Matrisome-associate	ECM Regulators	ADAM19	ADAM metallop	FKSG34|MADD	197	197	E5RIS2:E7ENW4:E9PD	NP_150377.1:XP	Mouse:Adam19|	NA
Matrisome-associate	ECM Regulators	ADAM20	ADAM metallop	-	199	199	O43506	NP_003805.3:XP	NA	NA
Matrisome-associate	ECM Regulators	ADAM21	ADAM metallop	ADAM31|MGC	200	200	Q9UKJ8	NP_003804.2	Mouse:Adam21|	NA
Matrisome-associate	ECM Regulators	ADAM22	ADAM metallop	MDC2|MGC14	201	201	E7EPF1:E9PF78:F8WA	NP_004185.1:NP	Mouse:Adam22|	NA
Matrisome-associate	ECM Regulators	ADAM23	ADAM metallop	MDC3	202	202	E7EWD3:H7C2M6:O75	NP_003803.1:XP	Mouse:Adam23|	NA
Matrisome-associate	ECM Regulators	ADAM30	ADAM metallop	svph4	208	208	Q9UKF2	NP_068566.2	Mouse:Adam30|	NA
Matrisome-associate	ECM Regulators	ADAM32	ADAM metallop	FLJ26299|FLJ29	15479	15479	E3W988:E5RGK7:E5RJ	NP_659441.3	Mouse:Adam32|	NA
Matrisome-associate	ECM Regulators	ADAM33	ADAM metallop	C20orf153|DJ9	15478	15478	A2A2L3:Q9BZ11	NP_001269376.1	Mouse:Adam33|	NA
Matrisome-associate	ECM Regulators	ADAM8	ADAM metallop	CD156|MGC13	215	215	H0YKZ1:H0YMT6:P783	NP_001100.3:NP	Mouse:Adam8|	NA
Matrisome-associate	ECM Regulators	ADAM9	ADAM metallop	CORD9|KIAA0	216	216	A0AVL1:C9J6H5:C9JP	NP_003807.1	Mouse:Adam9|	NA
Matrisome-associate	ECM Regulators	ADAMTS1	ADAM metallop	C3-C5|KIAA13	217	217	E5RI60:E5RJR7:H7C20	NP_008919.3	Mouse:Adamts1|	NA
Matrisome-associate	ECM Regulators	ADAMTS10	ADAM metallop	ADAM-TS10|W	13201	13201	M0QY12:M0QY36:Q9	NP_001269281.1	Mouse:Adamts10	NA
Matrisome-associate	ECM Regulators	ADAMTS12	ADAM metallop	PRO4389	14605	14605	D6REX0:P58397	NP_112217.2	Mouse:Adamts12	NA
Matrisome-associate	ECM Regulators	ADAMTS13	ADAM metallop	C9orf8|DKFZp4	1366	1366	E7EV88:Q76LX8:Q9UG	NP_620594.1:NP	Mouse:Adamts13	NA
Matrisome-associate	ECM Regulators	ADAMTS14	ADAM metallop	FLJ32820	14899	14899	Q8WXS8	NP_542453.2:NP	Mouse:Adamts14	NA
Matrisome-associate	ECM Regulators	ADAMTS16	ADAM metallop	ADAMTS16s|FL	17108	17108	Q2XQZ0:Q8TE57	NP_620687.2	Mouse:Adamts16	NA
Matrisome-associate	ECM Regulators	ADAMTS17	ADAM metallop	FLJ16363|FLJ32	17109	17109	H0YMH1:H3BRA9:H3B	NP_620688.2	Mouse:Adamts17	NA
Matrisome-associate	ECM Regulators	ADAMTS18	ADAM metallop	ADAMTS21	17110	17110	B4DEX3:H3BMG1:H3B	NP_955387.1	Mouse:Adamts18	NA
Matrisome-associate	ECM Regulators	ADAMTS19	ADAM metallop	FLJ16042	17111	17111	D6R9M2:H0Y8Y0:Q8T	NP_598377.3	Mouse:Adamts19	NA
Matrisome-associate	ECM Regulators	ADAMTS2	ADAM metallop	ADAM-TS2|AD	218	218	O95450	NP_055059.2:NP	Mouse:Adamts2|	NA
Matrisome-associate	ECM Regulators	ADAMTS3	ADAM metallop	ADAMTS-4|KIA	219	219	O15072	NP_055058.2	Mouse:Adamts3|	NA
Matrisome-associate	ECM Regulators	ADAMTS4	ADAM metallop	ADAMTS-2|AD	220	220	O75173:Q5VTW1	NP_005090.3	Mouse:Adamts4|	NA
Matrisome-associate	ECM Regulators	ADAMTS5	ADAM metallop	ADAMTS11|AD	221	221	Q9UNA0	NP_008969.2	Mouse:Adamts5|	NA
Matrisome-associate	ECM Regulators	ADAMTS6	ADAM metallop	ADAM-TS6	222	222	D6R9L6:Q9UKP5	NP_922932.2	Mouse:Adamts6|	NA
Matrisome-associate	ECM Regulators	ADAMTS7	ADAM metallop	ADAM-TS7|DK	223	223	Q9UKP4	NP_055087.2	Mouse:Adamts7|	NA
Matrisome-associate	ECM Regulators	ADAMTS8	ADAM metallop	ADAM-TS8|FLJ	224	224	Q9UP79	NP_008968.4:XP	Mouse:Adamts8|	NA
Matrisome-associate	ECM Regulators	ADAMTS9	ADAM metallop	FLJ42955|KIAA	13202	13202	C9JWI2:H0Y859:Q9P2	NP_891550.1:XP	Mouse:Adamts9|	NA
Matrisome-associate	ECM Regulators	ADAMTSL1	ADAMTS-like 1	ADAMTSR1|C9	14632	14632	A2A343:A6NIB9:F8WE	NP_001035362.3	Mouse:Adamtsl1|	NA
Matrisome-associate	ECM Regulators	ADAMTSL2	ADAMTS-like 2	FLJ45164|KIAA	14631	14631	B1B0D4:Q86TH1	NP_001138792.1	Mouse:Adamtsl2|	NA
Matrisome-associate	ECM Regulators	ADAMTSL3	ADAMTS-like 3	KIAA1233|MGC	14633	14633	P82987	NP_997400.2:XP	Mouse:Adamtsl3|	NA
Matrisome-associate	ECM Regulators	ADAMTSL4	ADAMTS-like 4	TSRC1	19706	19706	F8WAD0:Q6UY14	NP_001275536.1	Mouse:Adamtsl4|	NA
Matrisome-associate	ECM Regulators	AGT	angiotensinogen	ANHU|FLJ9259	333	333	P01019	NP_000020.1	Mouse:Agt|	NA
Matrisome-associate	ECM Regulators	AMBP	alpha-1-microg	A1M|EDC1|HC	453	453	P02760:S4R3Y4:S4R47	NP_001624.1	Mouse:Ambp|	NA
Matrisome-associate	ECM Regulators	BMP1	bone morphoge	FLJ44432|PCOL	1067	1067	B7ZKR5:E5RH22:P134	NP_001190.1:NP	Mouse:Bmp1|	NA

Matrisome-associate	ECM Regulators	C17orf58	chromosome 17	MGC138278	27568	27568	Q2M2W7	NP_858041.2:NP	NA	NA
Matrisome-associate	ECM Regulators	CD109	CD109 molecule	CPAMD7|DKFZ	21685	21685	Q6YHK3	NP_001153059.1	Mouse:Cd109|	NA
Matrisome-associate	ECM Regulators	CPAMD8	C3 and PZP-like	FLJ42058|FLJ90	23228	23228	F8W7D1:M0QXH3:M0	NP_056507.2	Mouse:Mug2|	NA
Matrisome-associate	ECM Regulators	CPN2	carboxypeptidas	ACBP	2313	2313	P22792	XP_005269337.1	Mouse:Cpn2|	NA
Matrisome-associate	ECM Regulators	CST1	cystatin SN	-	2473	2473	P01037	NP_001889.2	NA	Cystatin
Matrisome-associate	ECM Regulators	CST3	cystatin C	ARMD11|MGC	2475	2475	P01034	NP_000090.1:NP	Mouse:Cst3|	Cystatin
Matrisome-associate	ECM Regulators	CST4	cystatin S	MGC71923	2476	2476	P01036	NP_001890.1	NA	Cystatin
Matrisome-associate	ECM Regulators	CST6	cystatin E/M	-	2478	2478	Q15828	NP_001314.1	Mouse:Cst6|	Cystatin
Matrisome-associate	ECM Regulators	CSTB	cystatin B (stefin	CST6|EPM1|PM	2482	2482	P04080	NP_000091.1	Mouse:Cstb|	Cystatin
Matrisome-associate	ECM Regulators	CTSA	cathepsin A	GLB2|GSL|NGB	9251	9251	P10619:Q5JZG9:Q5JZH	NP_000299.2:NP	Mouse:Ctsa|	Cathepsin
Matrisome-associate	ECM Regulators	CTSB	cathepsin B	APPS|CPSB	2527	2527	E9PCB3:E9PHZ5:E9PID	NP_001899.1:NP	Mouse:Ctsb|	Cathepsin
Matrisome-associate	ECM Regulators	CTSC	cathepsin C	CPPI|DPP1|DPP	2528	2528	H0YCY8:H0YDA2:P536	NP_001107645.1	Mouse:Ctsc|	Cathepsin
Matrisome-associate	ECM Regulators	CTSD	cathepsin D	CLN10|CPSD|M	2529	2529	C9JH19:F8W787:F8WD	NP_001900.1	Mouse:Ctsd|	Cathepsin
Matrisome-associate	ECM Regulators	CTSF	cathepsin F	CATSF	2531	2531	E9PSC2:H0YD65:H0YE	NP_003784.2	Mouse:Ctsf|	Cathepsin
Matrisome-associate	ECM Regulators	CTSH	cathepsin H	ACC-4|ACC-5|	2535	2535	E9PKT6:E9PN60:E9PN8	NP_004381.2	Mouse:Ctsh|	Cathepsin
Matrisome-associate	ECM Regulators	CTSK	cathepsin K	CTS02|CTSO|C	2536	2536	P43235:Q5QP40	NP_000387.1	Mouse:Ctsk|	Cathepsin
Matrisome-associate	ECM Regulators	CTSL	cathepsin L1	CATL|CTSL|FLJ	2537	2537	P07711:Q5T8F0	NP_001244900.1	NA	Cathepsin
Matrisome-associate	ECM Regulators	CTSO	cathepsin O	CTSO1	2542	2542	P43234	NP_001325.1	Mouse:Ctso|	Cathepsin
Matrisome-associate	ECM Regulators	CTSS	cathepsin S	MGC3886	2545	2545	P25774:U3KPS4:U3KQ	NP_001186668.1	Mouse:Ctss|	Cathepsin
Matrisome-associate	ECM Regulators	CTSV	cathepsin L2	CATL2|CTSU|C	2538	2538	O60911	NP_001188504.1	Mouse:Ctsl|	Cathepsin
Matrisome-associate	ECM Regulators	CTSW	cathepsin W	LYPN	2546	2546	E9PI30:H0YDT2:P5620	NP_001326.2	Mouse:Ctsw|	Cathepsin
Matrisome-associate	ECM Regulators	CTSZ	cathepsin Z	CTSX|FLJ17088	2547	2547	Q9UBR2	NP_001327.2	Mouse:Ctsz|	Cathepsin
Matrisome-associate	ECM Regulators	EGLN1	egl nine homolo	C1orf12|DKFZp	1232	1232	Q9GZT9	NP_071334.1	Mouse:Egln1|	NA
Matrisome-associate	ECM Regulators	EGLN2	egl nine homolo	DKFZp434E026	14660	14660	M0QXM8:M0QXR0:M0	NP_444274.1:NP	Mouse:Egln2|	NA
Matrisome-associate	ECM Regulators	EGLN3	egl nine homolo	FLJ21620|HIFPH	14661	14661	F8VR39:F8W1G2:G3V3	NP_071356.1:XP	Mouse:Egln3|	NA
Matrisome-associate	ECM Regulators	F10	coagulation fact	FX|FXA	3528	3528	B7ZBK1:F8WBM7:P007	NP_000495.1:XP	Mouse:F10|	NA
Matrisome-associate	ECM Regulators	F12	coagulation fact	HAE3|HAEX|HA	3530	3530	P00748	NP_000496.2	Mouse:F12|	NA
Matrisome-associate	ECM Regulators	F13A1	coagulation fact	F13A	3531	3531	A6PVK5:H0Y4W5:H0Y	NP_000120.2:XP	Mouse:F13a1|	NA
Matrisome-associate	ECM Regulators	F2	coagulation fact	PT	3535	3535	C9JV37:E9PIT3:P00734	NP_000497.1	Mouse:F2|	NA
Matrisome-associate	ECM Regulators	F7	coagulation fact	-	3544	3544	E9PH36:F5H8B0:P0870	NP_000122.1:NP	Mouse:F7|	NA
Matrisome-associate	ECM Regulators	FAM20A	family with sequ	DKFZp434F232	23015	23015	K7EIV7:K7EQL5:Q96M	NP_001230675.1	Mouse:Fam20a|	NA
Matrisome-associate	ECM Regulators	FAM20B	family with sequ	"glycosaminog	23017	23017	O75063:X6RH03	NP_055679.1	Mouse:Fam20b|	NA
Matrisome-associate	ECM Regulators	FAM20C	family with sequ	"dentin matrix	22140	22140	Q8IXL6	NP_064608.2:XP	Mouse:Fam20c|	NA
Matrisome-associate	ECM Regulators	HPSE	heparanase	HPA|HPR1|HPS	5164	5164	D6RAQ1:D6RHG4:Q9Y	NP_001092010.1	Mouse:Hpse|	NA
Matrisome-associate	ECM Regulators	HPSE2	heparanase 2	FLJ11684|FLJ44	18374	18374	Q8WWQ2	NP_001159716.1	Mouse:Hpse2|	NA
Matrisome-associate	ECM Regulators	HTRA1	HtrA serine pept	ARMD7|HtrA|L	9476	9476	H0Y7G9:Q92743	NP_002766.1	Mouse:Htra1|	NA
Matrisome-associate	ECM Regulators	HTRA3	HtrA serine pept	Prsp|Tasp	30406	30406	P83110	NP_444272.1:XP	Mouse:Htra3|	NA
Matrisome-associate	ECM Regulators	HTRA4	HtrA serine pept	FLJ90724	26909	26909	P83105	NP_710159.1	Mouse:Htra4|	NA
Matrisome-associate	ECM Regulators	HYAL1	hyaluronoglucos	HYAL-1|LUCA1	5320	5320	C9JB49:C9JRK1:Q1279	NP_149349.2:NP	Mouse:Hyal1|	NA
Matrisome-associate	ECM Regulators	HYAL2	hyaluronoglucos	LUCA2	5321	5321	C9J700:C9JBF5:C9JSD1	NP_003764.3:NP	Mouse:Hyal2|	NA
Matrisome-associate	ECM Regulators	HYAL3	hyaluronoglucos	LUCA-3|LUCA1	5322	5322	C9JB51:O43820	NP_001186958.1	Mouse:Hyal3|	NA
Matrisome-associate	ECM Regulators	ITIH2	inter-alpha (glob	H2P|SHAP	6167	6167	P19823:Q5T985:Q5T98	NP_002207.2	Mouse:Itih2|	NA
Matrisome-associate	ECM Regulators	ITIH4	inter-alpha (glob	DKFZp686G21	6169	6169	B7ZKJ8:F5GXQ1:H7C0	NP_001159921.1	Mouse:Itih4|	NA
Matrisome-associate	ECM Regulators	ITIH5	inter-alpha (glob	DKFZp686F014	21449	21449	C9J2H1:G5E9D8:H7C5	NP_001001851.1	Mouse:Itih5|	NA
Matrisome-associate	ECM Regulators	ITIH6	inter-alpha (glob	UNQ6369|dJ14	28907	28907	Q6UXX5	NP_940912.1	Mouse:Itih5l-ps|	NA
Matrisome-associate	ECM Regulators	KAZALD1	Kazal-type serin	BONO1|FKSG2	25460	25460	Q96I82	NP_112191.2:XP	Mouse:Kazald1|	NA
Matrisome-associate	ECM Regulators	KY	kyphoscoliosis p	FLJ33207	26576	26576	B4DGA7:Q8NBH2	NP_848649.3	Mouse:Ky|	NA
Matrisome-associate	ECM Regulators	LOX	lysyl oxidase	MGC105112	6664	6664	H0YAL3:P28300	NP_002308.2	Mouse:Lox|	NA
Matrisome-associate	ECM Regulators	LOXL1	lysyl oxidase-like	LOL|LOXL	6665	6665	H3BUV8:Q08397	NP_005567.2	Mouse:Loxl1|	NA
Matrisome-associate	ECM Regulators	LOXL2	lysyl oxidase-like	LOR2|WS9-14	6666	6666	E5RFE2:E5RFY0:E5RHH	NP_002309.1	Mouse:Loxl2|	NA
Matrisome-associate	ECM Regulators	LOXL3	lysyl oxidase-like	LOXL	13869	13869	B8ZZT6:B9A025:C9J5M	NP_001276093.1	Mouse:Loxl3|	NA
Matrisome-associate	ECM Regulators	LOXL4	lysyl oxidase-like	FLJ21889|LOXC	17171	17171	Q96JB6	NP_115587.6	Mouse:Loxl4|	NA
Matrisome-associate	ECM Regulators	MASP1	mannan-binding	CRARF|CRARF1	6901	6901	C9J1C7:C9JLU5:C9JMA	NP_001027019.1	Mouse:Masp1|	NA
Matrisome-associate	ECM Regulators	MASP2	mannan-binding	MAP19|MASP-	6902	6902	O00187	NP_006601.2:NP	Mouse:Masp2|	NA

Matrisome-associated	ECM Regulators	MMP10	matrix metallope	SL-2|STMY2	7156	7156	F5GYX7:P09238	NP_002416.1	Mouse:Mmp10|	NA
Matrisome-associated	ECM Regulators	MMP11	matrix metallope	SL-3|ST3|STMY	7157	7157	E9PED7:F8WDI7:H7C3	NP_005931.2	Mouse:Mmp11|	NA
Matrisome-associated	ECM Regulators	MMP12	matrix metallope	HME|MGC1385	7158	7158	P39900	NP_002417.2	Mouse:Mmp12|	NA
Matrisome-associated	ECM Regulators	MMP14	matrix metallope	MMP-X1|MT1-	7160	7160	F8VP90:F8W1B7:P502	NP_004986.1	Mouse:Mmp14|	NA
Matrisome-associated	ECM Regulators	MMP15	matrix metallope	MT2-MMP|MT	7161	7161	H3BT97:P51511	NP_002419.1	Mouse:Mmp15|	NA
Matrisome-associated	ECM Regulators	MMP19	matrix metallope	MMP18|RASI-1	7165	7165	A6ND33:B4DNP3:F8W	NP_001259030.1	Mouse:Mmp19|	NA
Matrisome-associated	ECM Regulators	MMP2	matrix metallope	CLG4|CLG4A|M	7166	7166	H3BR66:H3BS34:H3BV	NP_001121363.1	Mouse:Mmp2|	NA
Matrisome-associated	ECM Regulators	MMP21	matrix metallope	-	14357	14357	Q8N119	NP_671724.1	Mouse:Mmp21|	NA
Matrisome-associated	ECM Regulators	MMP23B	matrix metallope	MIFR|MIFR-1|M	7171	7171	B4DMZ6:D6REP6:H0Y	NP_008914.1	Mouse:Mmp23|	NA
Matrisome-associated	ECM Regulators	MMP24	matrix metallope	MMP25|MT-M	7172	7172	Q9Y5R2	NP_006681.1	Mouse:Mmp24|	NA
Matrisome-associated	ECM Regulators	MMP25	matrix metallope	MMP20|MMP2	14246	14246	Q9NPA2	NP_071913.1	Mouse:Mmp25|	NA
Matrisome-associated	ECM Regulators	MMP28	matrix metallope	EPILYSIN|MM2	14366	14366	K7ELN8:K7EMV8:K7EN	NP_001027449.1	Mouse:Mmp28|	NA
Matrisome-associated	ECM Regulators	MMP3	matrix metallope	CHDS6|MGC12	7173	7173	E9PKX2:H7C139:P0825	NP_002413.1	Mouse:Mmp3|	NA
Matrisome-associated	ECM Regulators	MMP7	matrix metallope	MMP-7|MPSL1	7174	7174	P09237	NP_002414.1	Mouse:Mmp7|	NA
Matrisome-associated	ECM Regulators	NGLY1	N-glycanase 1	FLJ11005|FLJ12	17646	17646	C9JU75:H0Y2P2:Q96IV	NP_001138765.1	Mouse:Ngly1|	NA
Matrisome-associated	ECM Regulators	OGFOD1	2-oxoglutarate a	FLJ10826|KIAA	25585	25585	H3BP48:H3BR02:H3BR	NP_060703.3	Mouse:Ogfod1|	NA
Matrisome-associated	ECM Regulators	OGFOD2	2-oxoglutarate a	DKFZp686H151	25823	25823	F5H145:F5H890:Q6N0	NP_078899.1	Mouse:Ogfod2|	NA
Matrisome-associated	ECM Regulators	P4HA1	prolyl 4-hydroxy	P4HA	8546	8546	P13674	NP_000908.2:NP	Mouse:P4ha1|	NA
Matrisome-associated	ECM Regulators	P4HA2	prolyl 4-hydroxy	-	8547	8547	A8MXE0:C9JCP0:C9JFJ	NP_001017973.1	Mouse:P4ha2|	NA
Matrisome-associated	ECM Regulators	P4HA3	prolyl 4-hydroxy	-	30135	30135	E9PM97:H0YCC3:Q7Z4	NP_001275677.1	Mouse:P4ha3|	NA
Matrisome-associated	ECM Regulators	P4HTM	prolyl 4-hydroxy	EGLN4|HIFPH4	28858	28858	H7C153:Q9NXG6:V9G	NP_808807.2:NP	Mouse:P4htm|	NA
Matrisome-associated	ECM Regulators	PAMR1	peptidase doma	DKFZP586H212	24554	24554	E9PIV1:E9PMN5:E9PQ	NP_001001991.1	Mouse:Pamr1|	NA
Matrisome-associated	ECM Regulators	PAPPA	pregnancy-asso	ASBABP2|DIPL	8602	8602	F5GZ19:Q13219	NP_002572.2	Mouse:Pappa|	NA
Matrisome-associated	ECM Regulators	PAPPA2	pappalysin 2	PAPP-A2|PAPP	14615	14615	Q9BXP8	NP_064714.2:NP	Mouse:Pappa2|	NA
Matrisome-associated	ECM Regulators	PCSK5	proprotein conv	PC5|PC6|PC6A	8747	8747	B1AMG5:B1AMG8:Q5J	NP_001177411.1	Mouse:Pcsk5|	NA
Matrisome-associated	ECM Regulators	PCSK6	proprotein conv	PACE4|SPC4	8569	8569	E7EM82:E7EQ62:E7EW	NP_001278238.1	Mouse:Pcsk6|	NA
Matrisome-associated	ECM Regulators	PLAT	plasminogen act	DKFZp686I031	9051	9051	B4DN26:B4DNJ1:E5RG	NP_000921.1:NP	Mouse:Plat|	NA
Matrisome-associated	ECM Regulators	PLAU	plasminogen act	ATF|UPA|URK|u	9052	9052	E7ESM2:E7ET40:P0074	NP_001138503.1	Mouse:Plau|	NA
Matrisome-associated	ECM Regulators	PLOD1	procollagen-lysi	FLJ42041|LH|LH	9081	9081	B4DR87:Q02809:Q5JX	NP_000293.2	Mouse:Plod1|	NA
Matrisome-associated	ECM Regulators	PLOD2	procollagen-lysi	LH2|TLH	9082	9082	B3KWS3:C9JXZ0:E7ET	NP_000926.2:NP	Mouse:Plod2|	NA
Matrisome-associated	ECM Regulators	PLOD3	procollagen-lysi	LH3	9083	9083	C9JIX5:C9JU11:H7C0B	NP_001075.1	Mouse:Plod3|	NA
Matrisome-associated	ECM Regulators	PRSS12	protease, serine,	BSSP-3|BSSP3|	9477	9477	P56730	NP_003610.2	Mouse:Prss12|	NA
Matrisome-associated	ECM Regulators	PRSS3	protease, serine,	MTG|PRSS4|TR	9486	9486	B1AN99:P35030	NP_001184026.2	Mouse:Gm10334|	NA
Matrisome-associated	ECM Regulators	PZP	pregnancy-zone	CPAMD6|MGC	9750	9750	F5GXY0:P20742	NP_002855.2	Mouse:Pzp|	NA
Matrisome-associated	ECM Regulators	SERPINA1	serpin peptidase	A1A|A1AT|AAT	8941	8941	G3V2B9:G3V387:G3V4	NP_000286.3:NP	Mouse:Serpina1a|	NA
Matrisome-associated	ECM Regulators	SERPINA10	serpin peptidase	PZI|ZPI	15996	15996	G3V2W1:Q9UK55	NP_001094077.1	Mouse:Serpina10|	NA
Matrisome-associated	ECM Regulators	SERPINA11	serpin peptidase	-	19193	19193	Q86U17	NP_001073920.1	Mouse:Serpina11|	NA
Matrisome-associated	ECM Regulators	SERPINA3	serpin peptidase	AACT|ACT|GIG	16	16	G3V3A0:G3V595:P010	NP_001076.2	Mouse:Serpina3a|	NA
Matrisome-associated	ECM Regulators	SERPINA5	serpin peptidase	PAI3|PCI|PLAN	8723	8723	G3V264:G3V265:G3V2	NP_000615.3	Mouse:Serpina5|	NA
Matrisome-associated	ECM Regulators	SERPINA6	serpin peptidase	CBG	1540	1540	G3V350:G3V4V7:P081	NP_001747.2	Mouse:Serpina6|	NA
Matrisome-associated	ECM Regulators	SERPINB1	serpin peptidase	EI|ELANH2|LEI|	3311	3311	B4DNT0:P30740	NP_109591.1:XP	Mouse:Serpinb1a	NA
Matrisome-associated	ECM Regulators	SERPINB10	serpin peptidase	PI10|bomapin	8942	8942	H7BYS2:H7C004:P485	NP_005015.1	Mouse:Serpinb10	NA
Matrisome-associated	ECM Regulators	SERPINB2	serpin peptidase	HsT1201|PAI|P	8584	8584	E7EPJ9:E7ERB5:E9PDK	NP_001137290.1	Mouse:Serpinb2|	NA
Matrisome-associated	ECM Regulators	SERPINB6	serpin peptidase	CAP|DKFZp686	8950	8950	P35237	NP_001182220.2	Mouse:Serpinb6a	NA
Matrisome-associated	ECM Regulators	SERPINB8	serpin peptidase	CAP2|PI8	8952	8952	C9JTJ8:C9JVA8:H7BXK	NP_001027018.1	Mouse:Serpinb8|	NA
Matrisome-associated	ECM Regulators	SERPINB9	serpin peptidase	CAP-3|CAP3|P	8955	8955	P50453	NP_004146.1:XP	Mouse:Serpinb9|	NA
Matrisome-associated	ECM Regulators	SERPINC1	serpin peptidase	AT3|ATIII|MGC	775	775	P01008	NP_000479.1	Mouse:Serpinc1|	NA
Matrisome-associated	ECM Regulators	SERPINE1	serpin peptidase	PAI|PAI-1|PAI1	8583	8583	P05121	NP_000593.1	Mouse:Serpine1|	NA
Matrisome-associated	ECM Regulators	SERPINE2	serpin peptidase	GDN|PI7|PN1|P	8951	8951	C9JN98:C9JRK5:C9K03	NP_001130000.1	Mouse:Serpine2|	NA
Matrisome-associated	ECM Regulators	SERPINE3	serpin peptidase	-	24774	24774	A8MV23:E5RG55	NP_001094790.1	Mouse:Serpine3|	NA
Matrisome-associated	ECM Regulators	SERPINF1	serpin peptidase	EPC-1|PEDF|PI	8824	8824	I3L107:I3L1U4:I3L2R7:I	NP_002606.3	Mouse:Serpinf1|	NA
Matrisome-associated	ECM Regulators	SERPINF2	serpin peptidase	A2AP|AAP|ALP	9075	9075	C9JMH6:C9JPV4:P086	NP_000925.2:NP	Mouse:Serpinf2|	NA
Matrisome-associated	ECM Regulators	SERPING1	serpin peptidase	C1IN|C1INH|C1	1228	1228	B4E1F0:B4E1H2:B5MC	NP_000053.2:NP	Mouse:Serping1|	NA
Matrisome-associated	ECM Regulators	SERPINH1	serpin peptidase	AsTP3|CBP1|CB	1546	1546	E9PIG2:E9PJH8:E9PK8	NP_001193943.1	Mouse:Serpinh1|	NA

Matrisome-associated	ECM Regulators	SERPINI1	serpin peptidase	DKFZp781N131	8943	8943	C9JDY5:C9JQU8:H7C5	NP_001116224.1	Mouse:Serpini1|	NA
Matrisome-associated	ECM Regulators	SERPINI2	serpin peptidase	MEPI|PANCPIN	8945	8945	C9J7N5:C9JWE1:O758	NP_001012303.1	Mouse:Serpini2|	NA
Matrisome-associated	ECM Regulators	SLPI	secretory leukoc	ALK1|ALP|BLPI|	11092	11092	P03973	NP_003055.1	Mouse:Slpi|	NA
Matrisome-associated	ECM Regulators	SULF1	sulfatase 1	FLJ30905|FLJ38	20391	20391	E9PI06:E9PJL8:E9PLS5:	NP_001121676.1	Mouse:Sulf1|	NA
Matrisome-associated	ECM Regulators	SULF2	sulfatase 2	DKFZp313E091	20392	20392	B1AMP9:G3XAE6:H0Y8	NP_001155313.1	Mouse:Sulf2|	NA
Matrisome-associated	ECM Regulators	TGM1	transglutaminase	ICR2|KTG|LI|LI1	11777	11777	B4DWR7:H0YKI6:H0YL	NP_000350.1	Mouse:Tgm1|	NA
Matrisome-associated	ECM Regulators	TGM2	transglutaminase	G-ALPHA-h|GN	11778	11778	A2A299:A2A2A0:B4DIT	NP_004604.2:NP	Mouse:Tgm2|	NA
Matrisome-associated	ECM Regulators	TGM4	transglutaminase	FLJ26776|TGP|h	11780	11780	F8WCU8:P49221	NP_003232.2	Mouse:Tgm4|	NA
Matrisome-associated	ECM Regulators	TGM5	transglutaminase	MGC141907|TG	11781	11781	O43548	NP_004236.1:NP	Mouse:Tgm5|	NA
Matrisome-associated	ECM Regulators	TIMP1	TIMP metallopep	CLGI|EPA|EPO|I	11820	11820	B4DJK3:H0Y789:P0103	NP_003245.1:XP	Mouse:Timp1|	NA
Matrisome-associated	ECM Regulators	TIMP2	TIMP metallopep	CSC-21K	11821	11821	B4DFW2:K7EIX4:P1603	NP_003246.1	Mouse:Timp2|	NA
Matrisome-associated	ECM Regulators	TIMP3	TIMP metallopep	HSMRK222|K22	11822	11822	P35625	NP_000353.1	Mouse:Timp3|	NA
Matrisome-associated	ECM Regulators	TIMP4	TIMP metallopep	-	11823	11823	Q99727	NP_003247.1	Mouse:Timp4|	NA
Matrisome-associated	ECM Regulators	TLL2	tolloid-like 2	KIAA0932|MGC	11844	11844	Q9Y6L7	NP_036597.1	Mouse:Tll2|	NA
Matrisome-associated	Secreted Factors	AMH	anti-Mullerian h	MIF|MIS	464	464	K7EJU5:P03971	NP_000470.2	Mouse:Amh|	NA
Matrisome-associated	Secreted Factors	ANGPT1	angiopoietin 1	AGP1|AGPT|AN	484	484	B4DTQ9:E5RFF4:E7ERK	NP_001137.2:NP	Mouse:Angpt1|	NA
Matrisome-associated	Secreted Factors	ANGPT2	angiopoietin 2	AGPT2|ANG2	485	485	E7EVQ3:O15123	NP_001112359.1	Mouse:Angpt2|	NA
Matrisome-associated	Secreted Factors	ANGPTL1	angiopoietin-like	ANG3|ANGPT3	489	489	O95841	NP_004664.1:XP	Mouse:Angptl1|	NA
Matrisome-associated	Secreted Factors	ANGPTL2	angiopoietin-like	ARP2|HARP|MG	490	490	Q8NCH7:Q9UKU9	NP_036230.1	Mouse:Angptl2|	NA
Matrisome-associated	Secreted Factors	ANGPTL3	angiopoietin-like	ANGPT5	491	491	Q9Y5C1	NP_055310.1	Mouse:Angptl3|	NA
Matrisome-associated	Secreted Factors	ANGPTL4	angiopoietin-like	ANGPTL2|ARP4	16039	16039	M0QZ51:M0R0N8:M0R	NP_001034756.1	Mouse:Angptl4|	NA
Matrisome-associated	Secreted Factors	ANGPTL5	angiopoietin-like	-	19705	19705	E9PKF7:Q86XS5	NP_835228.2	NA	NA
Matrisome-associated	Secreted Factors	ANGPTL6	angiopoietin-like	AGF|ARP5	23140	23140	K7EKF6:Q8NI99	NP_114123.2	Mouse:Angptl6|	NA
Matrisome-associated	Secreted Factors	ANGPTL7	angiopoietin-like	AngX|CDT6|RP5	24078	24078	O43827	NP_066969.1	Mouse:Angptl7|	NA
Matrisome-associated	Secreted Factors	AREG	amphiregulin	AR|AREGB|CRD	651	651	D6RFX5:P15514	NP_001648.1	Mouse:Areg|	NA
Matrisome-associated	Secreted Factors	ARTN	artemin	ENOVIN|EVN|N	727	727	E9PIK2:E9PLG3:E9PN2	NP_001129687.1	Mouse:Artn|	NA
Matrisome-associated	Secreted Factors	BDNF	brain-derived ne	MGC34632	1033	1033	E9PMP3:P23560	NP_001137277.1	Mouse:Bdnf|	NA
Matrisome-associated	Secreted Factors	BMP2	bone morphoge	BMP2A	1069	1069	P12643	NP_001191.1	Mouse:Bmp2|	NA
Matrisome-associated	Secreted Factors	BMP3	bone morphoge	BMP-3A	1070	1070	P12645	NP_001192.2	Mouse:Bmp3|	NA
Matrisome-associated	Secreted Factors	BMP4	bone morphoge	BMP2B|BMP2B	1071	1071	H0YLW3:H0YM53:H0Y	NP_001193.2:NP	Mouse:Bmp4|	NA
Matrisome-associated	Secreted Factors	BMP6	bone morphoge	VGR|VGR1	1073	1073	P22004	NP_001709.1	Mouse:Bmp6|	NA
Matrisome-associated	Secreted Factors	BMP7	bone morphoge	OP-1	1074	1074	B1AKZ9:B1AL00:H0Y4	NP_001710.1	Mouse:Bmp7|	NA
Matrisome-associated	Secreted Factors	BMP8A	bone morphoge	FLJ14351|FLJ45	21650	21650	Q7Z5Y6	NP_861525.2	Mouse:Bmp8a|	NA
Matrisome-associated	Secreted Factors	BMP8B	bone morphoge	BMP8|MGC131	1075	1075	E7EMY8:P34820	NP_001711.2	Mouse:Bmp8b|	NA
Matrisome-associated	Secreted Factors	BTC	betacellulin	-	1121	1121	H0Y8Q5:P35070	NP_001720.1	Mouse:Btc|	NA
Matrisome-associated	Secreted Factors	C1QTNF9B	C1q and tumor	RP11-45B20.2|	34072	34072	B2RNN3	NP_001007538.1	NA	NA
Matrisome-associated	Secreted Factors	CBLN3	cerebellin 3 pre	PRO1486	20146	20146	G3V2Y8:Q6UW01	NP_001034860.1	Mouse:Cbln3|	NA
Matrisome-associated	Secreted Factors	CCBE1	collagen and cal	FLJ30681|MGC	29426	29426	K7EQ81:Q6UXH8	NP_597716.1:XP	Mouse:Ccbe1|	NA
Matrisome-associated	Secreted Factors	CCL13	chemokine (C-C	CKb10|MCP-4|	10611	10611	J3QLQ9:Q99616	NP_005399.1	NA	NA
Matrisome-associated	Secreted Factors	CCL18	chemokine (C-C	AMAC-1|AMAC	10616	10616	P55774	NP_002979.1	NA	NA
Matrisome-associated	Secreted Factors	CCL2	chemokine (C-C	GDCF-2|HC11|	10618	10618	J3KRT7:P13500	NP_002973.1	Mouse:Ccl2|	NA
Matrisome-associated	Secreted Factors	CCL25	chemokine (C-C	Ckb15|MGC150	10624	10624	C9JDZ7:O15444	NP_001188288.1	Mouse:Ccl25|	NA
Matrisome-associated	Secreted Factors	CCL26	chemokine (C-C	IMAC|MGC126	10625	10625	Q9Y258	NP_006063.1	Mouse:Ccl26|	NA
Matrisome-associated	Secreted Factors	CCL28	chemokine (C-C	CCK1|MEC|MG	17700	17700	D6RC73:Q9NRJ3	NP_683513.1:XP	Mouse:Ccl28|	NA
Matrisome-associated	Secreted Factors	CCL3	chemokine (C-C	G0S19-1|LD78	10627	10627	P10147	NP_002974.1	Mouse:Ccl3|	NA
Matrisome-associated	Secreted Factors	CCL4	chemokine (C-C	ACT2|AT744.1|	10630	10630	P13236:Q7M4M2	NP_002975.1:NP	Mouse:Ccl4|	NA
Matrisome-associated	Secreted Factors	CCL4L2	chemokine (C-C	AT744.2|CCL4L	24066	24066	F8W8W8:Q8NHW4	NP_001278397.1	Mouse:Ccl4|	NA
Matrisome-associated	Secreted Factors	CCL5	chemokine (C-C	D17S136E|MG	10632	10632	K7EJM0:P13501	NP_002976.2	Mouse:Ccl5|	NA
Matrisome-associated	Secreted Factors	CCL7	chemokine (C-C	FIC|MARC|MCP	10634	10634	A8MVH1:A8MX17:P80	NP_006264.2	Mouse:Ccl7|	NA
Matrisome-associated	Secreted Factors	CCL8	chemokine (C-C	HC14|MCP-2|M	10635	10635	P80075	NP_005614.2	Mouse:Ccl8|	NA
Matrisome-associated	Secreted Factors	CHRD	chordin	MGC133038	1949	1949	B7Z6F4:E7ESX1:Q9H2X	NP_003732.2:XP	Mouse:Chrd|	NA
Matrisome-associated	Secreted Factors	CHRDL2	chordin-like 2	BNF1|CHL2|DK	24168	24168	A8MQ10:H0YCU0:H0Y	NP_001265402.1	Mouse:Chrdl2|	NA
Matrisome-associated	Secreted Factors	CLCF1	cardiotrophin-lik	BSF-3|BSF3|CIS	17412	17412	Q9UBD9	NP_001159684.1	Mouse:Clcf1|	NA
Matrisome-associated	Secreted Factors	CRHBP	corticotropin rel	CRF-BP|CRFBP	2356	2356	D6RHH7:P24387	NP_001873.2	Mouse:Crhbp|	NA

Matrisome-associated	Secreted Factors	CRLF1	cytokine recepto	CISS|CISS1|CLF	2364	2364	M0QZL6:O75462	NP_004741.1	Mouse:Crlf1|	NA
Matrisome-associated	Secreted Factors	CRLF3	cytokine recepto	CREME9|CYTO	17177	17177	B4DJU5:J3KST8:J3QLS	NP_057070.3	Mouse:Crlf3|	NA
Matrisome-associated	Secreted Factors	CSF1	colony stimulati	MCSF|MGC319	2432	2432	E9PJA2:E9PKP4:E9PQ0	NP_000748.3:NP	Mouse:Csf1|	NA
Matrisome-associated	Secreted Factors	CSF3	colony stimulati	C17orf33|CSF3	2438	2438	J3KTH8:J3QRD4:J3QRX	NP_000750.1:NP	Mouse:Csf3|	NA
Matrisome-associated	Secreted Factors	CTF1	cardiotrophin 1	CT-1|CT1	2499	2499	Q16619	NP_001136016.1	Mouse:Ctf1|	NA
Matrisome-associated	Secreted Factors	CX3CL1	chemokine (C-X	ABCD-3|C3Xki	10647	10647	H3BSR6:H3BV86:J3QR	NP_002987.1	Mouse:Cx3cl1|	NA
Matrisome-associated	Secreted Factors	CXCL1	chemokine (C-X	FSP|GRO1|GRO	4602	4602	P09341	NP_001502.1	Mouse:Cxcl1|	NA
Matrisome-associated	Secreted Factors	CXCL10	chemokine (C-X	C7|IFI10|INP10	10637	10637	P02778	NP_001556.2	Mouse:Cxcl10|	NA
Matrisome-associated	Secreted Factors	CXCL12	chemokine (C-X	PBSF|SCYB12|S	10672	10672	P48061	NP_000600.1:NP	Mouse:Cxcl12|	NA
Matrisome-associated	Secreted Factors	CXCL14	chemokine (C-X	BMAC|BRAK|KE	10640	10640	O95715	NP_004878.2	Mouse:Cxcl14|	NA
Matrisome-associated	Secreted Factors	CXCL2	chemokine (C-X	CINC-2a|GRO2	4603	4603	P19875	NP_002080.1	Mouse:Cxcl2|	NA
Matrisome-associated	Secreted Factors	CXCL3	chemokine (C-X	CINC-2b|GRO3	4604	4604	P19876	NP_002081.2	Mouse:Cxcl3|	NA
Matrisome-associated	Secreted Factors	CXCL5	chemokine (C-X	ENA-78|SCYB5	10642	10642	P42830	NP_002985.1	Mouse:Cxcl5|	NA
Matrisome-associated	Secreted Factors	CXCL8	interleukin 8	CXCL8|GCP-1|(	6025	6025	C9J4T6:P10145	NP_000575.1	NA	NA
Matrisome-associated	Secreted Factors	EDA	ectodysplasin A	ED1|ED1-A1|ED	3157	3157	D6RA95:Q92838	NP_001005609.1	Mouse:Eda|	NA
Matrisome-associated	Secreted Factors	EGF	epidermal growt	HOMG4|URG	3229	3229	P01133	NP_001171601.1	Mouse:Egf|	NA
Matrisome-associated	Secreted Factors	EGFL7	EGF-like-domai	MGC111117|RI	20594	20594	Q9UHF1:R4GMT3	NP_057299.1:NP	Mouse:Egfl7|	NA
Matrisome-associated	Secreted Factors	EGFL8	EGF-like-domai	C6orf8|FLJ4449	13944	13944	Q5JP22:Q99944	NP_085155.1	Mouse:Egfl8|	NA
Matrisome-associated	Secreted Factors	EREG	epiregulin	ER	3443	3443	O14944	NP_001423.1	Mouse:Ereg|	NA
Matrisome-associated	Secreted Factors	FGF1	fibroblast growt	AFGF|ECGF|EC(	3665	3665	B5MCF4:C9JDC5:C9JU	NP_000791.1:NP	Mouse:Fgf1|	NA
Matrisome-associated	Secreted Factors	FGF10	fibroblast growt	-	3666	3666	D6RG33:O15520	NP_004456.1:XP	Mouse:Fgf10|	NA
Matrisome-associated	Secreted Factors	FGF11	fibroblast growt	FHF3|FLJ16061	3667	3667	I3L4N4:Q92914	NP_004103.1	Mouse:Fgf11|	NA
Matrisome-associated	Secreted Factors	FGF12	fibroblast growt	FGF12B|FHF1	3668	3668	C9JEN8:C9JIN3:C9JUK	NP_004104.3:NP	Mouse:Fgf12|	NA
Matrisome-associated	Secreted Factors	FGF13	fibroblast growt	FGF2|FHF-2|FH	3670	3670	B1AJW0:B1AK17:B1B1	NP_001132970.1	Mouse:Fgf13|	NA
Matrisome-associated	Secreted Factors	FGF14	fibroblast growt	FHF4|MGC1191	3671	3671	Q92915	NP_004106.1:NP	Mouse:Fgf14|	NA
Matrisome-associated	Secreted Factors	FGF17	fibroblast growt	FGF-13	3673	3673	O60258	NP_003858.1:XP	Mouse:Fgf17|	NA
Matrisome-associated	Secreted Factors	FGF18	fibroblast growt	FGF-18|ZFGF5	3674	3674	O76093	NP_003853.1	Mouse:Fgf18|	NA
Matrisome-associated	Secreted Factors	FGF2	fibroblast growt	BFGF|FGFB|HB(	3676	3676	P09038	NP_001997.5	Mouse:Fgf2|	NA
Matrisome-associated	Secreted Factors	FGF22	fibroblast growt	-	3679	3679	K7ELB9:O60371:Q9HC	NP_065688.1:XP	Mouse:Fgf22|	NA
Matrisome-associated	Secreted Factors	FGF23	fibroblast growt	ADHR|HPDR2|I	3680	3680	Q9GZV9	NP_065689.1	Mouse:Fgf23|	NA
Matrisome-associated	Secreted Factors	FGF5	fibroblast growt	HBGF-5|Smag-	3683	3683	H0Y9E2:P12034	NP_004455.2:NP	Mouse:Fgf5|	NA
Matrisome-associated	Secreted Factors	FGF7	fibroblast growt	HBGF-7|KGF	3685	3685	H0YNE7:P21781	NP_002000.1	Mouse:Fgf7|	NA
Matrisome-associated	Secreted Factors	FGF8	fibroblast growt	AIGF|HBGF-8|K	3686	3686	P55075:R4GMQ3	NP_006110.1:NP	Mouse:Fgf8|	NA
Matrisome-associated	Secreted Factors	FGF9	fibroblast growt	GAF|HBFG-9|M	3687	3687	P31371	NP_002001.1	Mouse:Fgf9|	NA
Matrisome-associated	Secreted Factors	FGFBP1	fibroblast growt	FGFBP|HBP17	19695	19695	Q14512	NP_005121.1	Mouse:Fgfbp1|	NA
Matrisome-associated	Secreted Factors	FGFBP2	fibroblast growt	HBP17RP|KSP3	29451	29451	Q9BYJ0	NP_114156.1	NA	NA
Matrisome-associated	Secreted Factors	FGFBP3	fibroblast growt	C10orf13|FGF-	23428	23428	Q8TAT2	NP_689642.3	Mouse:Fgfbp3|	NA
Matrisome-associated	Secreted Factors	FLG	filaggrin	ATOD2	3748	3748	P20930	NP_002007.1	Mouse:Flg|	NA
Matrisome-associated	Secreted Factors	FLG2	filaggrin family r	IFPS	33276	33276	Q5D862	NP_001014364.1	Mouse:Flg2|	NA
Matrisome-associated	Secreted Factors	FLT3LG	fms-related tyro	FL	3766	3766	M0QXI0:M0QYM9:M0	NP_001191431.1	Mouse:Flt3l|	NA
Matrisome-associated	Secreted Factors	FRZB	frizzled-related	FRE|FRITZ|FRP-	3959	3959	Q92765	NP_001454.2	Mouse:Frzb|	NA
Matrisome-associated	Secreted Factors	FST	follistatin	FS	3971	3971	H0YA75:H0YAF9:P198	NP_006341.1:NP	Mouse:Fst|	NA
Matrisome-associated	Secreted Factors	FSTL1	follistatin-like 1	FLJ50214|FLJ52	3972	3972	C9J5G4:H7C4W4:Q128	NP_009016.1	Mouse:Fstl1|	NA
Matrisome-associated	Secreted Factors	FSTL3	follistatin-like 3	FLRG|FSRP	3973	3973	K7EM71:K7ES54:O956	NP_005851.1	Mouse:Fstl3|	NA
Matrisome-associated	Secreted Factors	GDF10	growth different	BMP-3b|BMP3	4215	4215	P55107	NP_004953.1	Mouse:Gdf10|	NA
Matrisome-associated	Secreted Factors	GDF11	growth different	BMP-11|BMP1	4216	4216	H0YI30:O95390	NP_005802.1:XP	Mouse:Gdf11|	NA
Matrisome-associated	Secreted Factors	GDF15	growth different	GDF-15|MIC-1	30142	30142	Q99988	NP_004855.2	Mouse:Gdf15|	NA
Matrisome-associated	Secreted Factors	GDF5	growth different	BMP14|CDMP1	4220	4220	P43026	NP_000548.2	Mouse:Gdf5|	NA
Matrisome-associated	Secreted Factors	GDF6	growth different	BMP13|CDMP2	4221	4221	Q6KF10	NP_001001557.1	Mouse:Gdf6|	NA
Matrisome-associated	Secreted Factors	GDF7	growth different	BMP12	4222	4222	Q7Z4P5	NP_878248.2	Mouse:Gdf7|	NA
Matrisome-associated	Secreted Factors	GDF9	growth different	-	4224	4224	O60383	NP_001275753.1	Mouse:Gdf9|	NA
Matrisome-associated	Secreted Factors	GDNF	glial cell derived	ATF1|ATF2|HFB	4232	4232	P39905	NP_000505.1:NP	Mouse:Gdnf|	NA
Matrisome-associated	Secreted Factors	GH1	growth hormone	GH|GH-N|GHN	4261	4261	B1A4G9:P01241	NP_000506.2:NP	Mouse:Gh|	NA
Matrisome-associated	Secreted Factors	HBEGF	heparin-binding	DTR|DTS|DTSF|	3059	3059	Q99075	NP_001936.1	Mouse:Hbegf|	NA

Matrisome-associate	Secreted Factors	HCFC1	host cell factor C	CFF|HCF-1|HCF	4839	4839	A6NEM2:H7C1C4:P51	NP_005325.2:XP	Mouse:Hcfc1|	NA
Matrisome-associate	Secreted Factors	HCFC2	host cell factor C	FLJ94012|HCF-	24972	24972	C0H5X3:F8VU09:Q9Y5	NP_037452.1	Mouse:Hcfc2|	NA
Matrisome-associate	Secreted Factors	HGF	hepatocyte grow	DFNB39|F-TCF	4893	4893	C9JDP4:C9JJ35:C9JS80	NP_000592.3:NP	Mouse:Hgf|	NA
Matrisome-associate	Secreted Factors	HGFAC	HGF activator	HGFA|MGC138	4894	4894	D6RAR4:Q04756	NP_001519.1:XP	Mouse:Hgfac|	NA
Matrisome-associate	Secreted Factors	HHIP	hedgehog intera	FLJ20992|FLJ90	14866	14866	Q96QV1	NP_071920.1	Mouse:Hhip|	NA
Matrisome-associate	Secreted Factors	HRNR	hornerin	S100A16|S100a	20846	20846	Q86YZ3	NP_001009931.1	Mouse:Hrnr|	NA
Matrisome-associate	Secreted Factors	IGF1	insulin-like grow	IGF1A|IGFI	5464	5464	P05019:Q13429	NP_000609.1:NP	Mouse:Igf1|	NA
Matrisome-associate	Secreted Factors	IGF2	insulin-like grow	C11orf43|FLJ22	5466	5466	P01344	NP_000603.1:NP	Mouse:Igf2|	NA
Matrisome-associate	Secreted Factors	IL10	interleukin 10	CSIF|IL-10|IL10	5962	5962	P22301	NP_000563.1	Mouse:Il10|	NA
Matrisome-associate	Secreted Factors	IL11	interleukin 11	AGIF|IL-11	5966	5966	K7ESD5:P20809	NP_000632.1:NP	Mouse:Il11|	NA
Matrisome-associate	Secreted Factors	IL12A	interleukin 12A (	CLMF|IL-12A|N	5969	5969	E7ENE1:E9PGR3:P2945	NP_000873.2:XP	Mouse:Il12a|	NA
Matrisome-associate	Secreted Factors	IL13	interleukin 13	ALRH|BHR1|IL-	5973	5973	P35225	NP_002179.2	Mouse:Il13|	NA
Matrisome-associate	Secreted Factors	IL15	interleukin 15	IL-15|MGC972	5977	5977	P40933	NP_000576.1:NP	Mouse:Il15|	NA
Matrisome-associate	Secreted Factors	IL16	interleukin 16 (ly	FLJ16806|FLJ42	5980	5980	H0YKB7:H0YLB1:H0YL	NP_001165599.1	Mouse:Il16|	NA
Matrisome-associate	Secreted Factors	IL17B	interleukin 17B	IL-17B|IL-20|M	5982	5982	Q9UHF5	NP_055258.1	Mouse:Il17b|	NA
Matrisome-associate	Secreted Factors	IL17C	interleukin 17C	CX2|IL-17C|IL-	5983	5983	Q9P0M4	NP_037410.1	Mouse:Il17c|	NA
Matrisome-associate	Secreted Factors	IL17D	interleukin 17D	FLJ30846|IL-17	5984	5984	Q8TAD2	NP_612141.1	Mouse:Il17d|	NA
Matrisome-associate	Secreted Factors	IL18	interleukin 18 (in	IGIF|IL-18|IL-1	5986	5986	Q14116	NP_001230140.1	Mouse:Il18|	NA
Matrisome-associate	Secreted Factors	IL1A	interleukin 1, alp	IL-1A|IL1|IL1-A	5991	5991	P01583	NP_000566.3	Mouse:Il1a|	NA
Matrisome-associate	Secreted Factors	IL1B	interleukin 1, be	IL-1|IL1-BETA|I	5992	5992	C9JSC2:C9JVK0:C9JWV	NP_000567.1	Mouse:Il1b|	NA
Matrisome-associate	Secreted Factors	IL1RN	interleukin 1 rec	DIRA|ICIL-1RA|	6000	6000	P18510	NP_000568.1:NP	Mouse:Il1rn|	NA
Matrisome-associate	Secreted Factors	IL20	interleukin 20	IL-20|IL10D|MG	6002	6002	Q9NYY1	NP_061194.2:XP	Mouse:Il20|	NA
Matrisome-associate	Secreted Factors	IL23A	interleukin 23, a	IL-23|IL-23A|IL	15488	15488	Q9NPF7	NP_057668.1	Mouse:Il23a|	NA
Matrisome-associate	Secreted Factors	IL24	interleukin 24	C49A|FISP|IL-2	11346	11346	E7ER64:E7ETP6:Q1300	NP_001172085.1	Mouse:Il24|	NA
Matrisome-associate	Secreted Factors	IL25	interleukin 25	IL17E	13765	13765	Q9H293	NP_073626.1:NP	Mouse:Il25|	NA
Matrisome-associate	Secreted Factors	IL26	interleukin 26	AK155|IL-26	17119	17119	Q9NPH9	NP_060872.1	NA	NA
Matrisome-associate	Secreted Factors	IL34	interleukin 34	C16orf77|IL-34	28529	28529	H3BPB7:J3QQT3:Q6ZN	NP_001166242.1	Mouse:Il34|	NA
Matrisome-associate	Secreted Factors	IL4	interleukin 4	BCGF-1|BCGF1	6014	6014	P05112	NP_000580.1:NP	Mouse:Il4|	NA
Matrisome-associate	Secreted Factors	IL6	interleukin 6 (int	BSF2|HGF|HSF|	6018	6018	B4DNQ5:B4DNV3:B5M	NP_000591.1:XP	Mouse:Il6|	NA
Matrisome-associate	Secreted Factors	IL7	interleukin 7	IL-7	6023	6023	P13232:Q5FBY3:Q5FBY	NP_000871.1:NP	Mouse:Il7|	NA
Matrisome-associate	Secreted Factors	INHA	inhibin, alpha	-	6065	6065	P05111	NP_002182.1	Mouse:Inha|	NA
Matrisome-associate	Secreted Factors	INHBA	inhibin, beta A	EDF|FRP	6066	6066	P08476	NP_002183.1	Mouse:Inhba|	NA
Matrisome-associate	Secreted Factors	INHBB	inhibin, beta B	MGC157939	6067	6067	P09529	NP_002184.2	Mouse:Inhbb|	NA
Matrisome-associate	Secreted Factors	INHBE	inhibin, beta E	MGC4638	24029	24029	F8VNP4:P58166	NP_113667.1	Mouse:Inhbe|	NA
Matrisome-associate	Secreted Factors	INSL3	insulin-like 3 (Le	MGC119818|M	6086	6086	M0QXQ3:P51460	NP_001252516.1	Mouse:Insl3|	NA
Matrisome-associate	Secreted Factors	INSL6	insulin-like 6	RIF1	6089	6089	Q9Y581	NP_009110.2	Mouse:Insl6|	NA
Matrisome-associate	Secreted Factors	ISM1	isthmin 1 homol	C20orf82|ISM|I	16213	16213	B1AKI9	NP_543016.1	Mouse:Ism1|	NA
Matrisome-associate	Secreted Factors	ISM2	isthmin 2 homol	DKFZp686E021	23176	23176	G3V2Q2:G3XAI3:H7C4	NP_872315.2:NP	Mouse:Ism2|	NA
Matrisome-associate	Secreted Factors	KITLG	KIT ligand	DKFZp686F225	6343	6343	P21583:S4R384:S4R44	NP_000890.1:NP	Mouse:Kitl|	NA
Matrisome-associate	Secreted Factors	LEFTY1	left-right determ	LEFTB|LEFTYB	6552	6552	O75610	NP_066277.1	Mouse:Lefty1|	NA
Matrisome-associate	Secreted Factors	LEFTY2	left-right determ	EBAF|LEFTA|LE	3122	3122	O00292	NP_001165896.1	Mouse:Lefty2|	NA
Matrisome-associate	Secreted Factors	LEP	leptin	FLJ94114|OB|O	6553	6553	P41159	NP_000221.1:XP	Mouse:Lep|	NA
Matrisome-associate	Secreted Factors	LIF	leukemia inhibit	CDF|DIA|HILDA	6596	6596	P15018	NP_001244064.1	Mouse:Lif|	NA
Matrisome-associate	Secreted Factors	LTA	lymphotoxin alp	LT|TNFB|TNFSF	6709	6709	P01374	NP_000586.2:NP	Mouse:Lta|	NA
Matrisome-associate	Secreted Factors	LTB	lymphotoxin bet	TNFC|TNFSF3|p	6711	6711	Q06643	NP_002332.1:NP	Mouse:Ltb|	NA
Matrisome-associate	Secreted Factors	MDK	midkine (neurite	FLJ27379|MK|N	6972	6972	C9JHA4:E9PLM6:E9PPJ	NP_001012333.1	Mouse:Mdk|	NA
Matrisome-associate	Secreted Factors	MEGF10	multiple EGF-like	DKFZp781K185	29634	29634	Q96KG7	NP_001243474.1	Mouse:Megf10|	NA
Matrisome-associate	Secreted Factors	MEGF11	multiple EGF-like	DKFZp434L121	29635	29635	A6BM72:C9JYE7:H3BR	NP_115821.2	Mouse:Megf11|	NA
Matrisome-associate	Secreted Factors	MEGF6	multiple EGF-like	EGFL3|KIAA081	3232	3232	H7C557:H7C5N9:O75	NP_001400.3	Mouse:Megf6|	NA
Matrisome-associate	Secreted Factors	MEGF8	multiple EGF-like	C19orf49|EGFL	3233	3233	F5GZG7:M0QZL2:M0R	NP_001258867.1	Mouse:Megf8|	NA
Matrisome-associate	Secreted Factors	MEGF9	multiple EGF-like	EGFL5	3234	3234	C9J1K8:Q9H1U4	NP_001073966.2	Mouse:Megf9|	NA
Matrisome-associate	Secreted Factors	MST1	macrophage stin	D3F15S2|DNF1	7380	7380	F5H6L8:G3XAK1:H7C0	NP_066278.3	Mouse:Mst1|	NA
Matrisome-associate	Secreted Factors	MST1L	macrophage stin	D1F15S1A|MSF	7390	7390	Q2TV78	NP_001258662.1	NA	NA
Matrisome-associate	Secreted Factors	NGF	nerve growth fa	Beta-NGF|HSA	7808	7808	P01138	NP_002497.2:XP	Mouse:Ngf|	NA

Matrisome-associate	Secreted Factors	NODAL	nodal homolog	MGC138230	7865	7865	H7C0E4:Q96S42	NP_060525.3	Mouse:Nodal|	NA
Matrisome-associate	Secreted Factors	NRG2	neuregulin 2	Don-1|HRG2|N	7998	7998	F5GZS7:F5H0N2:O145	NP_001171864.1	Mouse:Nrg2|	NA
Matrisome-associate	Secreted Factors	NRG4	neuregulin 4	DKFZp779N05	29862	29862	E9PJI7:F8W729:H3BP6	NP_612640.1	Mouse:Nrg4|	NA
Matrisome-associate	Secreted Factors	NRTN	neurturin	NTN	8007	8007	Q99748	NP_004549.1	Mouse:Nrtn|	NA
Matrisome-associate	Secreted Factors	OSM	oncostatin M	MGC20461	8506	8506	B5MC70:B5MCX1:P13	NP_065391.1	Mouse:Osm|	NA
Matrisome-associate	Secreted Factors	PDGFA	platelet-derived	PDGF-A|PDGF1	8799	8799	H7BYW6:P04085	NP_002598.4:NP	Mouse:Pdgfa|	NA
Matrisome-associate	Secreted Factors	PDGFB	platelet-derived	FLJ12858|PDGF	8800	8800	A9UJN9:A9UJP0:P0112	NP_002599.1:NP	Mouse:Pdgfb|	NA
Matrisome-associate	Secreted Factors	PDGFC	platelet derived	FALLOTEIN|SC	8801	8801	B4E3A5:H0Y9N2:J3KN	NP_057289.1	Mouse:Pdgfc|	NA
Matrisome-associate	Secreted Factors	PDGFD	platelet derived	IEGF|MGC2686	30620	30620	H0YD37:Q9GZP0	NP_079484.1:NP	Mouse:Pdgfd|	NA
Matrisome-associate	Secreted Factors	PGF	placental growth	D12S1900|PGF	8893	8893	G3XA84:P49763	NP_001193941.1	Mouse:Pgf|	NA
Matrisome-associate	Secreted Factors	PIK3IP1	phosphoinositid	HGFL|MGC173	24942	24942	C9JMK5:Q96FE7	NP_001129383.1	Mouse:Pik3ip1|	NA
Matrisome-associate	Secreted Factors	PSPN	persephin	PSP	9579	9579	M0QYK2:O60542	NP_004149.1	Mouse:Pspn|	NA
Matrisome-associate	Secreted Factors	PTN	pleiotrophin	HARP|HBGF8|H	9630	9630	C9JR52:P21246	NP_002816.1:XP	Mouse:Ptn|	NA
Matrisome-associate	Secreted Factors	S100A1	S100 calcium bi	S100|S100-alp	10486	10486	P23297:Q5T7Y4:Q5T7	NP_006262.1	Mouse:S100a1|	NA
Matrisome-associate	Secreted Factors	S100A10	S100 calcium bi	42C|ANX2L|AN	10487	10487	P60903	NP_002957.1	Mouse:S100a10|	NA
Matrisome-associate	Secreted Factors	S100A11	S100 calcium bi	MLN70|S100C	10488	10488	P31949	NP_005611.1	Mouse:S100a11|	NA
Matrisome-associate	Secreted Factors	S100A13	S100 calcium bi	-	10490	10490	Q99584	NP_001019381.1	Mouse:S100a13|	NA
Matrisome-associate	Secreted Factors	S100A14	S100 calcium bi	BCMP84|S100A	18901	18901	Q9HCY8	NP_065723.1:XP	Mouse:S100a14|	NA
Matrisome-associate	Secreted Factors	S100A16	S100 calcium bi	AAG13|DT1P1A	20441	20441	Q96FQ6	NP_525127.1:XP	Mouse:S100a16|	NA
Matrisome-associate	Secreted Factors	S100A2	S100 calcium bi	CAN19|MGC11	10492	10492	P29034:Q5RHS7:R4GN	NP_005969.1	Mouse:S100a2|	NA
Matrisome-associate	Secreted Factors	S100A3	S100 calcium bi	S100E	10493	10493	P33764	NP_002951.1	Mouse:S100a3|	NA
Matrisome-associate	Secreted Factors	S100A4	S100 calcium bi	18A2|42A|CAP	10494	10494	P26447	NP_002952.1:NP	Mouse:S100a4|	NA
Matrisome-associate	Secreted Factors	S100A5	S100 calcium bi	S100D	10495	10495	P33763:Q52LE7	NP_002953.2:XP	Mouse:S100a5|	NA
Matrisome-associate	Secreted Factors	S100A6	S100 calcium bi	2A9|5B10|CAB	10496	10496	P06703:R4GN98	NP_055439.1	Mouse:S100a6|	NA
Matrisome-associate	Secreted Factors	S100A7	S100 calcium bi	PSOR1|S100A7	10497	10497	P31151	NP_002954.2:XP	Mouse:S100a7a|	NA
Matrisome-associate	Secreted Factors	S100A8	S100 calcium bi	60B8AG|CAGA	10498	10498	P05109	NP_002955.2	Mouse:S100a8|	NA
Matrisome-associate	Secreted Factors	S100A9	S100 calcium bi	60B8AG|CAGB|	10499	10499	P06702	NP_002956.1	Mouse:S100a9|	NA
Matrisome-associate	Secreted Factors	S100B	S100 calcium bi	NEF|S100|S100	10500	10500	A8MRB1:P04271	NP_006263.1	Mouse:S100b|	NA
Matrisome-associate	Secreted Factors	S100P	S100 calcium bi	MIG9	10504	10504	P25815	NP_005971.1	NA	NA
Matrisome-associate	Secreted Factors	SCUBE1	signal peptide, C	-	13441	13441	B1AH90:H0Y4Y9:Q8IW	NP_766638.2	Mouse:Scube1|	NA
Matrisome-associate	Secreted Factors	SCUBE2	signal peptide, C	CEGB1|CEGF1|	30425	30425	E7EQD6:H0YD28:H0Y	NP_001164161.1	Mouse:Scube2|	NA
Matrisome-associate	Secreted Factors	SCUBE3	signal peptide, C	CEGF3|DKFZp6	13655	13655	Q8IX30	NP_689966.2:XP	Mouse:Scube3|	NA
Matrisome-associate	Secreted Factors	SFRP1	secreted frizzled	FRP|FRP-1|FRP	10776	10776	Q6ZSL4:Q8N474	NP_003003.3	Mouse:Sfrp1|	NA
Matrisome-associate	Secreted Factors	SFRP2	secreted frizzled	FRP-2|SARP1|S	10777	10777	Q96HF1	NP_003004.1	Mouse:Sfrp2|	NA
Matrisome-associate	Secreted Factors	SFRP4	secreted frizzled	FRP-4|FRPHE|N	10778	10778	C9JMJ2:Q6FHJ7	NP_003005.2	Mouse:Sfrp4|	NA
Matrisome-associate	Secreted Factors	SFRP5	secreted frizzled	SARP3	10779	10779	Q5T4F7	NP_003006.2	Mouse:Sfrp5|	NA
Matrisome-associate	Secreted Factors	TCHH	trichohyalin	MGC157889|M	11791	11791	Q07283	NP_009044.2	Mouse:Tchh|	NA
Matrisome-associate	Secreted Factors	TCHHL1	trichohyalin-like	S100A17|THHL	31796	31796	Q5QJ38	NP_001008536.1	Mouse:Tchhl1|	NA
Matrisome-associate	Secreted Factors	TDGF1	teratocarcinoma	CR|CRGF|CRIPT	11701	11701	F5H1T8:P13385	NP_001167607.1	Mouse:Tdgf1|	NA
Matrisome-associate	Secreted Factors	TGFA	transforming gr	TFGA	11765	11765	E7EPT6:F8VNR3:F8WA	NP_001093161.1	Mouse:Tgfa|	NA
Matrisome-associate	Secreted Factors	TGFB1	transforming gr	CED|DPD1|LAP	11766	11766	M0R2S0:P01137	NP_000651.3	Mouse:Tgfb1|	NA
Matrisome-associate	Secreted Factors	TGFB2	transforming gr	MGC116892|T	11768	11768	P61812	NP_001129071.1	Mouse:Tgfb2|	NA
Matrisome-associate	Secreted Factors	TGFB3	transforming gr	ARVD|FLJ1657	11769	11769	P10600:Q8WV88	NP_003230.1:XP	Mouse:Tgfb3|	NA
Matrisome-associate	Secreted Factors	THPO	thrombopoietin	MGC163194|M	11795	11795	F8W6L1:P40225	NP_000451.1:NP	Mouse:Thpo|	NA
Matrisome-associate	Secreted Factors	TNF	tumor necrosis f	DIF|TNF-alpha	11892	11892	P01375	NP_000585.2	Mouse:Tnf|	NA
Matrisome-associate	Secreted Factors	TNFSF10	tumor necrosis f	APO2L|Apo-2L	11925	11925	H7C246:P50591	NP_001177871.1	Mouse:Tnfsf10|	NA
Matrisome-associate	Secreted Factors	TNFSF11	tumor necrosis f	CD254|ODF|O	11926	11926	O14788	NP_003692.1:NP	Mouse:Tnfsf11|	NA
Matrisome-associate	Secreted Factors	TNFSF12	tumor necrosis f	APO3L|DR3LG|	11927	11927	C0H5Y4:O43508	NP_003800.1:NP	Mouse:Tnfsf12|	NA
Matrisome-associate	Secreted Factors	TNFSF13	tumor necrosis f	APRIL|CD256|T	11928	11928	C9JF68:C9JFN2:K7EJ28	NP_001185552.1	Mouse:Tnfsf13|	NA
Matrisome-associate	Secreted Factors	TNFSF13B	tumor necrosis f	BAFF|BLYS|CD2	11929	11929	Q9Y275	NP_001139117.1	Mouse:Tnfsf13b|	NA
Matrisome-associate	Secreted Factors	TNFSF15	tumor necrosis f	MGC129934|M	11931	11931	O95150:X6R8I9	NP_001191273.1	Mouse:Tnfsf15|	NA
Matrisome-associate	Secreted Factors	TNFSF4	tumor necrosis f	CD134L|CD252	11934	11934	P23510:Q8IV74	NP_003317.1:XP	Mouse:Tnfsf4|	NA
Matrisome-associate	Secreted Factors	TNFSF9	tumor necrosis f	4-1BB-L|CD13	11939	11939	P41273	NP_003802.1:XP	Mouse:Tnfsf9|	NA
Matrisome-associate	Secreted Factors	VEGFA	vascular endoth	MGC70609|MV	12680	12680	A2A2V4:H0YBI8:H0YB	NP_001020537.2	Mouse:Vegfa|	NA

Matrisome-associated	Secreted Factors	VEGFB	vascular endothe	VEGFL|VRF	12681	12681	H0YGB6:P49765	NP_001230662.1	Mouse:Vegfb|	NA
Matrisome-associated	Secreted Factors	VWC2	von Willebrand f	MGC131845|PS	30200	30200	Q2TAL6	NP_940972.2	Mouse:Vwc2|	NA
Matrisome-associated	Secreted Factors	WFIKKN1	WAP, follistatin/	C16orf12|MGC	30912	30912	Q96NZ8	NP_444514.1	Mouse:Wfikkn1|	NA
Matrisome-associated	Secreted Factors	WFIKKN2	WAP, follistatin/	WFIKKNRP	30916	30916	C9J6G4:Q8TEU8	NP_783165.1	Mouse:Wfikkn2|	NA
Matrisome-associated	Secreted Factors	WIF1	WNT inhibitory f	WIF-1	18081	18081	F5H8A3:H0YFK7:Q9Y5	NP_009122.2	Mouse:Wif1|	NA
Matrisome-associated	Secreted Factors	WNT10A	wingless-type M	FLJ14301|SSPS	13829	13829	H7BZB8:Q9GZT5	NP_079492.2	Mouse:Wnt10a|	NA
Matrisome-associated	Secreted Factors	WNT10B	wingless-type M	SHFM6|WNT-1	12775	12775	B5MCC8:C9J3H3:C9JC	NP_003385.2	Mouse:Wnt10b|	NA
Matrisome-associated	Secreted Factors	WNT11	wingless-type M	HWNT11|MGC	12776	12776	O96014	NP_004617.2:XP	Mouse:Wnt11|	NA
Matrisome-associated	Secreted Factors	WNT16	wingless-type M	-	16267	16267	E9PH60:Q9UBV4	NP_057171.2:NP	Mouse:Wnt16|	NA
Matrisome-associated	Secreted Factors	WNT2B	wingless-type M	WNT13|XWNT2	12781	12781	Q5TEH8:Q93097	NP_001278809.1	Mouse:Wnt2b|	NA
Matrisome-associated	Secreted Factors	WNT3	wingless-type M	INT4|MGC1319	12782	12782	P56703	NP_110380.1	Mouse:Wnt3|	NA
Matrisome-associated	Secreted Factors	WNT4	wingless-type M	SERKAL|WNT-4	12783	12783	B1AJZ6:H0Y663:P5670	NP_110388.2:XP	Mouse:Wnt4|	NA
Matrisome-associated	Secreted Factors	WNT5B	wingless-type M	MGC2648	16265	16265	F5GYM2:F5H034:F5H3	NP_110402.2:NP	Mouse:Wnt5b|	NA
Matrisome-associated	Secreted Factors	WNT7B	wingless-type M	-	12787	12787	A8K0G1:B8A595:B8A5	NP_478679.1	Mouse:Wnt7b|	NA
Matrisome-associated	Secreted Factors	WNT9A	wingless-type M	MGC138165|M	12778	12778	O14904	NP_003386.1	Mouse:Wnt9a|	NA
Matrisome-associated	Secreted Factors	WNT9B	wingless-type M	WNT14B|WNT1	12779	12779	E7EPC3:I3L0L8:O14905	NP_003387.1:XP	Mouse:Wnt9b|	NA
Matrisome-associated	Secreted Factors	XCL1	chemokine (C m	ATAC|LPTN|LTN	10645	10645	P47992	NP_002986.1	Mouse:Xcl1|	NA
Matrisome-associated	Secreted Factors	ZFP91	zinc finger prote	FKSG11|PZF|ZN	14983	14983	Q96JP5	NP_001183980.1	Mouse:Zfp91-Cnt	NA
