# Supplementary material for: Spatially defined single-cell transcriptional profiling characterizes diverse chondrocyte subtypes and nucleus pulposus progenitors in human intervertebral discs
Source: Bone Res. 2021 Aug 16;9:37. doi: 10.1038/s41413-021-00163-z (PMC8368097; doi:10.1038/s41413-021-00163-z)
Supplement: Supplementary file 6 — Supplementary Table 5 [file 41413_2021_163_MOESM6_ESM.pdf]

Supplementary Table 5. DEGs among the 4 NPPC subclusters								
p_val	avg_logFC	pct.1	pct.2	p_val_adj	cluster	gene	is.TF	is.surface
#######	1.585020824	0.999	0.838	4.34E-227	NPPC-1	COMP	FALSE	FALSE
#######	1.522864699	0.983	0.743	1.72E-203	NPPC-1	FN1	FALSE	FALSE
#######	1.241786535	1	0.985	3.09E-190	NPPC-1	CLU	FALSE	FALSE
#######	1.28535403	0.968	0.676	3.15E-189	NPPC-1	AEBP1	TRUE	FALSE
#######	1.389865971	0.923	0.472	7.36E-181	NPPC-1	FMOD	FALSE	FALSE
#######	1.391894882	0.901	0.481	3.37E-174	NPPC-1	MFGE8	FALSE	FALSE
#######	2.207113374	0.723	0.178	6.67E-162	NPPC-1	RARRES1	FALSE	FALSE
#######	1.310383835	0.713	0.165	5.98E-155	NPPC-1	ENPP1	FALSE	TRUE
#######	0.977377285	0.988	0.896	4.47E-151	NPPC-1	PRELP	FALSE	FALSE
#######	1.381563675	0.825	0.347	2.68E-144	NPPC-1	THBS4	FALSE	FALSE
#######	1.47720271	0.946	0.675	1.92E-142	NPPC-1	IGFBP6	FALSE	FALSE
#######	1.148588457	0.912	0.528	5.68E-137	NPPC-1	COL12A1	FALSE	FALSE
#######	1.092167844	0.976	0.795	3.20E-131	NPPC-1	MT1X	FALSE	FALSE
#######	1.064393856	0.885	0.453	1.51E-126	NPPC-1	ASPN	FALSE	FALSE
#######	1.43795298	0.844	0.369	4.85E-126	NPPC-1	WISP2	TRUE	FALSE
#######	1.171226399	0.544	0.09	2.26E-123	NPPC-1	LRMDA	FALSE	FALSE
#######	1.031982818	0.987	0.887	1.31E-118	NPPC-1	MT2A	FALSE	FALSE
#######	1.028570379	0.498	0.064	4.52E-118	NPPC-1	MYLK	TRUE	FALSE
#######	1.561936527	0.362	0.012	2.03E-109	NPPC-1	KERA	FALSE	FALSE
#######	1.034244531	0.967	0.765	2.88E-109	NPPC-1	GPX3	FALSE	FALSE
#######	0.571151261	1	0.996	7.81E-106	NPPC-1	DCN	FALSE	FALSE
#######	0.938098309	0.789	0.36	1.19E-99	NPPC-1	ANGPTL5	FALSE	FALSE
#######	1.332035778	0.707	0.259	2.75E-96	NPPC-1	MYOC	FALSE	FALSE
#######	0.934427083	0.758	0.317	6.25E-96	NPPC-1	RBP4	FALSE	FALSE
1.01E-99	0.842198241	0.932	0.77	2.55E-95	NPPC-1	OGN	FALSE	FALSE
7.73E-98	1.647831991	0.341	0.02	1.95E-93	NPPC-1	SAA1	FALSE	FALSE
1.27E-95	1.020643808	0.638	0.212	3.20E-91	NPPC-1	MAF	TRUE	FALSE
7.06E-95	1.006352703	0.848	0.528	1.78E-90	NPPC-1	THBS2	FALSE	FALSE
8.45E-86	0.727523089	0.376	0.048	2.13E-81	NPPC-1	PDE3B	FALSE	FALSE
1.47E-83	0.929996694	0.678	0.304	3.70E-79	NPPC-1	NBL1	FALSE	FALSE
1.23E-81	0.979523371	0.508	0.138	3.11E-77	NPPC-1	TNC	FALSE	FALSE
4.81E-81	0.896500626	0.723	0.391	1.21E-76	NPPC-1	SSR3	FALSE	FALSE
4.32E-77	1.020256073	0.614	0.245	1.09E-72	NPPC-1	LTBP2	FALSE	FALSE
2.39E-75	0.871177922	0.74	0.392	6.02E-71	NPPC-1	NTRK2	FALSE	TRUE
6.86E-73	0.671025381	0.439	0.101	1.73E-68	NPPC-1	KCNMA1	FALSE	FALSE
1.44E-72	0.752856492	0.781	0.405	3.63E-68	NPPC-1	GAS1	FALSE	TRUE
2.05E-72	0.696851255	0.806	0.51	5.18E-68	NPPC-1	NPC2	FALSE	FALSE
3.91E-72	0.850870201	0.414	0.091	9.87E-68	NPPC-1	CHAD	FALSE	FALSE
1.72E-70	0.707188637	0.848	0.547	4.34E-66	NPPC-1	CRYAB	FALSE	FALSE
1.15E-69	0.828246738	0.883	0.629	2.90E-65	NPPC-1	MT1E	FALSE	FALSE
1.70E-69	1.808960481	0.592	0.259	4.30E-65	NPPC-1	CHI3L1	FALSE	FALSE
1.96E-69	0.849181271	0.407	0.098	4.94E-65	NPPC-1	KCNK15	FALSE	FALSE
3.45E-67	1.206980123	0.517	0.169	8.70E-63	NPPC-1	ANGPTL7	FALSE	FALSE
2.59E-63	0.545291443	0.964	0.922	6.55E-59	NPPC-1	NEAT1	FALSE	FALSE
1.26E-61	0.740842871	0.449	0.135	3.19E-57	NPPC-1	ITGA10	FALSE	TRUE
1.55E-61	2.150458912	0.515	0.22	3.92E-57	NPPC-1	PTGDS	FALSE	FALSE
1.74E-60	0.85935577	0.734	0.442	4.40E-56	NPPC-1	TSC22D1	TRUE	FALSE
3.25E-60	0.778199593	0.685	0.333	8.21E-56	NPPC-1	SLPI	FALSE	FALSE
2.55E-59	0.763206116	0.714	0.412	6.44E-55	NPPC-1	PLPP3	FALSE	FALSE
2.64E-58	0.463141869	0.961	0.849	6.66E-54	NPPC-1	TIMP2	FALSE	FALSE
1.37E-57	0.696151121	0.443	0.146	3.47E-53	NPPC-1	AFF3	FALSE	FALSE
1.72E-57	0.628796629	0.869	0.64	4.33E-53	NPPC-1	MXRA8	FALSE	TRUE
1.34E-55	0.632462786	0.364	0.091	3.39E-51	NPPC-1	PIEZO2	FALSE	TRUE
2.83E-55	0.612429235	0.703	0.36	7.14E-51	NPPC-1	MGST1	FALSE	FALSE
5.50E-55	0.576564917	0.924	0.773	1.39E-50	NPPC-1	ADIRF	FALSE	FALSE
6.95E-55	0.563019058	0.313	0.062	1.75E-50	NPPC-1	SEMA3B	FALSE	FALSE
1.34E-54	0.70627048	0.771	0.528	3.38E-50	NPPC-1	PIK3R1	FALSE	FALSE
7.23E-53	0.690288241	0.584	0.272	1.83E-48	NPPC-1	SLC40A1	FALSE	TRUE
2.32E-52	0.724866373	0.593	0.293	5.85E-48	NPPC-1	CDKN1C	FALSE	FALSE
2.74E-52	0.822089699	0.477	0.187	6.90E-48	NPPC-1	LOX	FALSE	FALSE
2.98E-52	0.588619008	0.288	0.054	7.51E-48	NPPC-1	LMO3	FALSE	FALSE
7.88E-51	0.590651482	0.432	0.144	1.99E-46	NPPC-1	PAX1	TRUE	FALSE
2.66E-50	0.388917137	0.997	0.97	6.71E-46	NPPC-1	FTL	FALSE	FALSE
4.69E-49	0.984348783	0.78	0.547	1.18E-44	NPPC-1	CTGF	FALSE	FALSE
6.35E-46	0.39696456	0.232	0.035	1.60E-41	NPPC-1	MKX	TRUE	FALSE
1.23E-44	0.749737107	0.382	0.127	3.11E-40	NPPC-1	ADAMTS5	FALSE	FALSE
2.58E-44	0.500849487	0.268	0.057	6.51E-40	NPPC-1	HSPA2	FALSE	FALSE
1.30E-43	0.55149877	0.748	0.508	3.28E-39	NPPC-1	APLP2	FALSE	TRUE
1.31E-42	0.596386861	0.431	0.172	3.29E-38	NPPC-1	IVNS1ABP	FALSE	FALSE
1.34E-42	0.564501208	0.551	0.264	3.38E-38	NPPC-1	ITGBL1	FALSE	FALSE
2.58E-42	0.519656872	0.569	0.282	6.52E-38	NPPC-1	MATN2	FALSE	FALSE
3.21E-42	0.518017454	0.302	0.082	8.10E-38	NPPC-1	HACD4	FALSE	FALSE
4.59E-42	0.605458534	0.465	0.202	1.16E-37	NPPC-1	LIMCH1	FALSE	FALSE
7.32E-42	0.55657728	0.849	0.691	1.85E-37	NPPC-1	NNMT	FALSE	FALSE
8.16E-42	0.55145528	0.259	0.057	2.06E-37	NPPC-1	VIPR2	FALSE	TRUE
2.03E-40	0.490966931	0.242	0.05	5.12E-36	NPPC-1	TCEAL2	TRUE	FALSE
7.32E-40	0.460300706	0.218	0.038	1.85E-35	NPPC-1	MYO10	FALSE	FALSE
1.28E-39	0.542560702	0.571	0.301	3.22E-35	NPPC-1	ARHGAP2	FALSE	FALSE
2.02E-39	0.902328707	0.319	0.103	5.10E-35	NPPC-1	NOV	FALSE	FALSE
2.97E-39	0.559835652	0.435	0.181	7.50E-35	NPPC-1	ELL2	FALSE	FALSE
1.03E-38	0.536015188	0.546	0.281	2.61E-34	NPPC-1	SCARA3	FALSE	FALSE
2.08E-38	0.483666557	0.459	0.196	5.25E-34	NPPC-1	STEAP2	FALSE	FALSE
2.55E-38	0.54429608	0.704	0.475	6.44E-34	NPPC-1	PPP3CA	FALSE	FALSE
2.85E-38	0.541143219	0.318	0.101	7.19E-34	NPPC-1	PTPRD	FALSE	TRUE
6.76E-38	0.40461566	0.887	0.71	1.71E-33	NPPC-1	NUPR1	FALSE	FALSE

1.03E-37	0.425180047	0.238	0.052	2.61E-33	NPPC-1	GPM6B	FALSE	TRUE
1.09E-37	0.495994805	0.681	0.425	2.74E-33	NPPC-1	CTSK	FALSE	FALSE
2.47E-37	0.26705147	0.997	0.999	6.22E-33	NPPC-1	MALAT1	FALSE	FALSE
3.13E-37	0.545090516	0.395	0.152	7.90E-33	NPPC-1	CMYA5	FALSE	FALSE
1.10E-36	0.433454462	0.826	0.682	2.77E-32	NPPC-1	ARL6IP5	FALSE	FALSE
9.27E-36	0.491722138	0.721	0.466	2.34E-31	NPPC-1	METTL7A	FALSE	FALSE
2.25E-34	0.560896431	0.678	0.446	5.68E-30	NPPC-1	SCRG1	FALSE	FALSE
3.17E-34	0.483593834	0.202	0.04	7.99E-30	NPPC-1	FRZB	FALSE	FALSE
1.09E-33	0.496759868	0.735	0.515	2.76E-29	NPPC-1	LIMA1	FALSE	FALSE
9.38E-33	0.365911604	0.931	0.841	2.37E-28	NPPC-1	S100A4	FALSE	FALSE
2.89E-31	0.541250089	0.596	0.367	7.30E-27	NPPC-1	SAT1	FALSE	FALSE
5.14E-31	0.300105499	0.987	0.976	1.30E-26	NPPC-1	CST3	FALSE	FALSE
6.49E-31	0.404975416	0.206	0.048	1.64E-26	NPPC-1	PTGIS	FALSE	FALSE
7.09E-31	0.494498738	0.417	0.188	1.79E-26	NPPC-1	USP53	FALSE	FALSE
2.30E-30	0.497180751	0.505	0.268	5.79E-26	NPPC-1	FZD8	FALSE	TRUE
3.46E-30	0.61224689	0.535	0.296	8.74E-26	NPPC-1	CILP	FALSE	FALSE
8.74E-30	0.43009359	0.308	0.112	2.20E-25	NPPC-1	DNM3OS	FALSE	FALSE
4.29E-29	0.444434424	0.44	0.22	1.08E-24	NPPC-1	AIG1	FALSE	FALSE
1.50E-28	0.426634152	0.669	0.45	3.77E-24	NPPC-1	TSPO	FALSE	FALSE
5.63E-28	0.489110816	0.432	0.222	1.42E-23	NPPC-1	VAMP5	FALSE	FALSE
8.44E-28	0.438036668	0.26	0.088	2.13E-23	NPPC-1	LAG3	FALSE	TRUE
8.54E-28	0.434601596	0.256	0.087	2.16E-23	NPPC-1	NOX4	FALSE	TRUE
1.52E-27	0.503646545	0.264	0.091	3.84E-23	NPPC-1	TIPARP	FALSE	FALSE
1.58E-27	0.466090721	0.444	0.228	3.98E-23	NPPC-1	AR	TRUE	FALSE
1.68E-27	0.482235606	0.82	0.649	4.24E-23	NPPC-1	IGFBP5	FALSE	FALSE
2.44E-27	0.563267615	0.519	0.299	6.15E-23	NPPC-1	MT1M	FALSE	FALSE
5.64E-27	0.446439469	0.624	0.4	1.42E-22	NPPC-1	ECM2	FALSE	FALSE
7.60E-27	0.367487451	0.306	0.118	1.92E-22	NPPC-1	FAM107B	FALSE	FALSE
2.20E-26	0.421624951	0.246	0.08	5.54E-22	NPPC-1	CACNB4	FALSE	FALSE
2.56E-26	0.633516911	0.477	0.259	6.45E-22	NPPC-1	THBS1	FALSE	FALSE
3.82E-26	0.4052145	0.207	0.059	9.64E-22	NPPC-1	CDH13	FALSE	TRUE
1.06E-25	0.434494123	0.669	0.451	2.66E-21	NPPC-1	PLXDC2	FALSE	TRUE
2.39E-25	0.314674761	0.95	0.766	6.02E-21	NPPC-1	FBLN1	FALSE	FALSE
2.50E-25	0.352710077	0.203	0.057	6.30E-21	NPPC-1	ST8SIA1	FALSE	FALSE
3.08E-25	0.343343308	0.21	0.062	7.78E-21	NPPC-1	CAVIN2	FALSE	FALSE
3.27E-25	0.393589654	0.929	0.854	8.25E-21	NPPC-1	COL1A2	FALSE	FALSE
1.15E-24	0.400940396	0.211	0.065	2.89E-20	NPPC-1	LXN	FALSE	FALSE
1.31E-24	0.294486368	0.923	0.829	3.32E-20	NPPC-1	PSAP	FALSE	FALSE
1.40E-24	0.389216388	0.381	0.182	3.53E-20	NPPC-1	DSEL	FALSE	FALSE
1.57E-24	0.39569326	0.607	0.4	3.96E-20	NPPC-1	CYB5R3	FALSE	FALSE
1.72E-24	0.402725173	0.389	0.19	4.34E-20	NPPC-1	COL8A1	FALSE	FALSE
2.90E-24	0.400618268	0.429	0.223	7.32E-20	NPPC-1	KIF22	TRUE	FALSE
3.70E-24	0.731782112	0.228	0.075	9.35E-20	NPPC-1	CHI3L2	FALSE	FALSE
5.75E-24	0.398666534	0.776	0.621	1.45E-19	NPPC-1	S100A13	FALSE	FALSE
1.09E-23	0.373333253	0.781	0.61	2.76E-19	NPPC-1	BGN	FALSE	FALSE
1.66E-23	0.411990381	0.669	0.467	4.18E-19	NPPC-1	MRC2	FALSE	TRUE
1.84E-23	0.401195639	0.674	0.495	4.65E-19	NPPC-1	LINC0157	FALSE	FALSE
3.84E-23	0.558553524	0.337	0.156	9.70E-19	NPPC-1	HIST1H1C	FALSE	FALSE
5.46E-23	0.394555472	0.317	0.138	1.38E-18	NPPC-1	FGD4	FALSE	FALSE
2.13E-22	0.404602628	0.488	0.289	5.38E-18	NPPC-1	BEX3	FALSE	FALSE
2.80E-22	0.392888665	0.591	0.387	7.06E-18	NPPC-1	TRPS1	TRUE	FALSE
3.29E-22	0.397137111	0.373	0.181	8.30E-18	NPPC-1	DHRS3	FALSE	FALSE
3.96E-22	0.394995534	0.408	0.22	9.99E-18	NPPC-1	MIR100HG	FALSE	FALSE
4.73E-22	0.306836264	0.87	0.766	1.19E-17	NPPC-1	CD99	FALSE	FALSE
7.95E-22	0.407675353	0.342	0.168	2.01E-17	NPPC-1	CKB	FALSE	FALSE
8.51E-22	0.368992874	0.551	0.348	2.15E-17	NPPC-1	AHI1	FALSE	FALSE
3.53E-21	0.470464949	0.462	0.277	8.91E-17	NPPC-1	RHOB	FALSE	FALSE
9.05E-21	0.349324979	0.309	0.141	2.28E-16	NPPC-1	VASN	FALSE	TRUE
1.36E-20	0.394764253	0.331	0.16	3.43E-16	NPPC-1	NHLRC3	FALSE	FALSE
3.28E-20	0.323623187	0.201	0.068	8.28E-16	NPPC-1	CADM1	FALSE	TRUE
3.62E-20	0.366075111	0.385	0.206	9.15E-16	NPPC-1	MPDZ	FALSE	FALSE
8.53E-20	0.352770044	0.354	0.18	2.15E-15	NPPC-1	FAXDC2	FALSE	FALSE
1.00E-19	0.387503896	0.389	0.209	2.53E-15	NPPC-1	PLA2R1	FALSE	TRUE
1.06E-19	0.350279682	0.302	0.141	2.66E-15	NPPC-1	HOXA10	TRUE	FALSE
1.08E-19	0.455740703	0.311	0.151	2.73E-15	NPPC-1	ITM2C	FALSE	TRUE
1.56E-19	0.354192725	0.257	0.109	3.93E-15	NPPC-1	MYO1D	FALSE	FALSE
2.72E-19	0.335787114	0.308	0.146	6.86E-15	NPPC-1	ADARB1	TRUE	FALSE
3.21E-19	0.390338161	0.546	0.365	8.11E-15	NPPC-1	SYNE1	FALSE	FALSE
3.26E-19	0.305706324	0.34	0.167	8.24E-15	NPPC-1	FAT4	FALSE	TRUE
3.36E-19	0.302319336	0.225	0.088	8.47E-15	NPPC-1	CACHD1	FALSE	TRUE
4.73E-19	0.423444908	0.291	0.137	1.19E-14	NPPC-1	DCXR	FALSE	FALSE
4.84E-19	0.348119377	0.364	0.191	1.22E-14	NPPC-1	PKD1	FALSE	TRUE
1.25E-18	0.294168521	0.609	0.399	3.15E-14	NPPC-1	CPE	FALSE	FALSE
1.60E-18	0.368119869	0.583	0.407	4.04E-14	NPPC-1	H2AFJ	FALSE	FALSE
1.76E-18	0.39240894	0.51	0.328	4.45E-14	NPPC-1	TSPAN4	FALSE	TRUE
3.43E-18	0.344277258	0.484	0.301	8.66E-14	NPPC-1	TM4SF1	FALSE	TRUE
7.78E-18	0.363232572	0.62	0.463	1.96E-13	NPPC-1	NDUFA11	FALSE	FALSE
9.77E-18	0.305310815	0.811	0.638	2.47E-13	NPPC-1	PDGFRA	FALSE	TRUE
1.34E-17	0.258464493	0.202	0.075	3.37E-13	NPPC-1	COL11A1	FALSE	FALSE
1.35E-17	0.32590367	0.237	0.102	3.41E-13	NPPC-1	C14orf132	FALSE	TRUE
2.50E-17	0.380891429	0.561	0.381	6.31E-13	NPPC-1	BCL6	TRUE	FALSE
4.67E-17	0.323561065	0.441	0.266	1.18E-12	NPPC-1	C9orf3	FALSE	FALSE
8.02E-17	0.30996271	0.239	0.104	2.02E-12	NPPC-1	CPXM2	FALSE	FALSE
1.10E-16	0.323107275	0.591	0.422	2.77E-12	NPPC-1	PLS3	FALSE	FALSE
1.25E-16	0.283708966	0.319	0.165	3.15E-12	NPPC-1	COPRS	FALSE	FALSE
1.36E-16	0.287264356	0.223	0.093	3.44E-12	NPPC-1	APBB2	FALSE	FALSE
1.88E-16	0.321519632	0.326	0.173	4.74E-12	NPPC-1	IRS2	FALSE	FALSE
2.29E-16	0.341708953	0.23	0.101	5.78E-12	NPPC-1	FIBIN	FALSE	FALSE
2.37E-16	0.298925102	0.479	0.296	5.98E-12	NPPC-1	CCDC88A	FALSE	FALSE

2.49E-16	0.307043936	0.314	0.161	6.29E-12	NPPC-1	MXRA5	FALSE	FALSE
2.65E-16	0.3394966	0.573	0.402	6.68E-12	NPPC-1	ZIC1	TRUE	FALSE
3.95E-16	0.330124546	0.683	0.531	9.97E-12	NPPC-1	RABAC1	FALSE	FALSE
5.27E-16	0.267564033	0.857	0.734	1.33E-11	NPPC-1	SELENOM	FALSE	FALSE
7.30E-16	0.346137501	0.519	0.339	1.84E-11	NPPC-1	GAS6	FALSE	FALSE
1.11E-15	0.328167309	0.39	0.23	2.81E-11	NPPC-1	PRCP	FALSE	FALSE
2.91E-15	0.298261847	0.201	0.085	7.33E-11	NPPC-1	COL8A2	FALSE	FALSE
7.57E-15	0.271463421	0.247	0.119	1.91E-10	NPPC-1	IDH1	FALSE	FALSE
7.80E-15	0.314498278	0.717	0.6	1.97E-10	NPPC-1	SOD1	TRUE	FALSE
9.55E-15	0.308061564	0.355	0.206	2.41E-10	NPPC-1	PLOD2	FALSE	FALSE
9.96E-15	0.344074784	0.489	0.33	2.51E-10	NPPC-1	SEPT11	FALSE	FALSE
1.01E-14	0.291209313	0.224	0.101	2.54E-10	NPPC-1	F2R	FALSE	TRUE
1.04E-14	0.306409897	0.538	0.378	2.62E-10	NPPC-1	ISCU	FALSE	FALSE
2.67E-14	0.258930226	0.251	0.122	6.73E-10	NPPC-1	STEAP1	FALSE	FALSE
2.75E-14	0.267499715	0.211	0.095	6.93E-10	NPPC-1	PMEPA1	FALSE	TRUE
5.27E-14	0.260989282	0.329	0.183	1.33E-09	NPPC-1	PRKAG2	FALSE	FALSE
7.94E-14	0.291150406	0.336	0.194	2.00E-09	NPPC-1	SLC43A3	FALSE	TRUE
8.42E-14	0.278667984	0.25	0.124	2.12E-09	NPPC-1	NID2	FALSE	FALSE
9.78E-14	0.357400073	0.381	0.238	2.47E-09	NPPC-1	SERTAD4-	FALSE	FALSE
1.57E-13	0.360897897	0.414	0.272	3.97E-09	NPPC-1	OAF	FALSE	FALSE
1.67E-13	0.256361505	0.206	0.094	4.20E-09	NPPC-1	MAN1C1	FALSE	FALSE
2.02E-13	0.32510956	0.259	0.138	5.10E-09	NPPC-1	AKR7A2	FALSE	FALSE
2.78E-13	0.313216774	0.309	0.178	7.02E-09	NPPC-1	CCNDBP1	FALSE	FALSE
4.74E-13	0.258870699	0.43	0.278	1.20E-08	NPPC-1	TMED4	FALSE	FALSE
5.05E-13	0.291317274	0.269	0.146	1.27E-08	NPPC-1	GRAMD2B	FALSE	FALSE
6.46E-13	0.280879517	0.324	0.189	1.63E-08	NPPC-1	GYPC	FALSE	TRUE
1.15E-12	0.268478916	0.453	0.299	2.91E-08	NPPC-1	LRPAP1	FALSE	FALSE
1.84E-12	0.257405946	0.431	0.286	4.65E-08	NPPC-1	SGCB	FALSE	TRUE
2.02E-12	0.294852714	0.673	0.547	5.11E-08	NPPC-1	BRI3	FALSE	FALSE
2.41E-12	0.274910892	0.435	0.291	6.08E-08	NPPC-1	GLS	FALSE	FALSE
3.80E-12	0.273050506	0.373	0.233	9.60E-08	NPPC-1	ANG	FALSE	FALSE
3.84E-12	0.304388791	0.275	0.155	9.70E-08	NPPC-1	FAM180B	FALSE	FALSE
4.94E-12	0.254821268	0.277	0.156	1.25E-07	NPPC-1	PKIG	FALSE	FALSE
5.83E-12	0.298365501	0.338	0.209	1.47E-07	NPPC-1	SLCO3A1	FALSE	TRUE
6.47E-12	0.280684891	0.439	0.296	1.63E-07	NPPC-1	ZNF704	TRUE	FALSE
8.15E-12	0.284472366	0.363	0.23	2.06E-07	NPPC-1	RHEB	FALSE	FALSE
2.53E-11	0.343143244	0.364	0.236	6.39E-07	NPPC-1	HIF1A	TRUE	FALSE
5.26E-11	0.281277944	0.208	0.109	1.33E-06	NPPC-1	SLC29A1	FALSE	TRUE
7.17E-11	0.254846778	0.355	0.228	1.81E-06	NPPC-1	RCN3	FALSE	FALSE
1.79E-10	0.277118481	0.474	0.344	4.52E-06	NPPC-1	REXO2	FALSE	FALSE
1.79E-10	0.27085399	0.261	0.153	4.52E-06	NPPC-1	PLA2G16	FALSE	FALSE
1.05E-09	0.254850311	0.431	0.3	2.65E-05	NPPC-1	CRIP1	FALSE	FALSE
1.37E-09	0.348099755	0.411	0.301	3.45E-05	NPPC-1	DSE	FALSE	FALSE
2.07E-09	0.255965626	0.471	0.339	5.22E-05	NPPC-1	CTSD	FALSE	FALSE
3.02E-09	0.264123772	0.224	0.128	7.62E-05	NPPC-1	ZNF703	TRUE	FALSE
3.53E-09	0.279449554	0.396	0.282	8.92E-05	NPPC-1	COPZ2	FALSE	FALSE
7.83E-09	0.303742268	0.306	0.201	0.0001976	NPPC-1	ZNF503	TRUE	FALSE
1.58E-08	0.298123425	0.579	0.46	0.000399348	NPPC-1	DUSP1	FALSE	FALSE
7.40E-07	0.369625517	0.245	0.163	0.018666449	NPPC-1	MT1F	FALSE	FALSE
1.04E-06	0.256719504	0.221	0.141	0.026130584	NPPC-1	HELLPAR	FALSE	FALSE
#######	2.095103228	0.991	0.451	2.13E-268	NPPC-2	CFH	FALSE	FALSE
#######	2.55064238	0.775	0.065	2.21E-259	NPPC-2	C7	FALSE	FALSE
#######	1.43688626	0.684	0.12	1.43E-164	NPPC-2	FGF7	FALSE	FALSE
#######	1.078851017	0.766	0.172	3.26E-149	NPPC-2	EFEMP1	FALSE	FALSE
#######	1.129496178	0.929	0.542	2.30E-133	NPPC-2	SPARCL1	FALSE	FALSE
#######	0.964963645	0.942	0.542	1.25E-121	NPPC-2	CFD	FALSE	FALSE
#######	1.043607237	0.914	0.692	1.18E-113	NPPC-2	TXNIP	FALSE	FALSE
#######	0.781438971	0.988	0.935	5.27E-108	NPPC-2	GSN	FALSE	FALSE
1.59E-97	0.965942995	0.765	0.387	4.01E-93	NPPC-2	SELENOP	FALSE	FALSE
9.66E-94	2.544288147	0.503	0.139	2.44E-89	NPPC-2	APOE	FALSE	FALSE
1.40E-91	0.834622766	0.969	0.904	3.53E-87	NPPC-2	IGFBP7	FALSE	FALSE
1.89E-91	1.42854657	0.597	0.201	4.76E-87	NPPC-2	CXCL12	FALSE	FALSE
9.37E-91	0.938943785	0.739	0.339	2.36E-86	NPPC-2	NR2F2	TRUE	FALSE
1.99E-88	0.963126598	0.493	0.121	5.02E-84	NPPC-2	NRP1	FALSE	TRUE
1.64E-86	1.030557227	0.846	0.611	4.14E-82	NPPC-2	COL6A3	FALSE	FALSE
2.66E-86	1.124465347	0.481	0.117	6.72E-82	NPPC-2	SPRY1	FALSE	FALSE
3.00E-81	0.952015256	0.688	0.313	7.58E-77	NPPC-2	ABCA6	FALSE	TRUE
7.80E-80	0.93306833	0.665	0.307	1.97E-75	NPPC-2	GGT5	FALSE	FALSE
1.64E-79	0.827119297	0.571	0.196	4.14E-75	NPPC-2	IFITM1	FALSE	FALSE
2.75E-74	0.851023263	0.792	0.527	6.94E-70	NPPC-2	ZFP36L2	FALSE	FALSE
1.39E-70	0.855461345	0.694	0.334	3.50E-66	NPPC-2	CP	FALSE	FALSE
6.01E-68	0.921119899	0.579	0.245	1.52E-63	NPPC-2	PTGFR	FALSE	TRUE
5.92E-65	0.739319074	0.536	0.187	1.49E-60	NPPC-2	TFPI	FALSE	TRUE
1.23E-63	0.854955166	0.561	0.233	3.09E-59	NPPC-2	ZEB2	TRUE	FALSE
2.97E-62	0.77135024	0.424	0.124	7.49E-58	NPPC-2	CLEC2B	FALSE	FALSE
2.43E-61	0.817335079	0.495	0.177	6.13E-57	NPPC-2	TSHZ2	TRUE	FALSE
2.36E-60	0.54901217	0.924	0.867	5.95E-56	NPPC-2	CALD1	FALSE	FALSE
3.22E-60	0.544015392	0.971	0.932	8.13E-56	NPPC-2	TMSB4X	FALSE	FALSE
6.94E-58	0.651065915	0.936	0.838	1.75E-53	NPPC-2	FOS	TRUE	FALSE
1.16E-57	0.638797584	0.244	0.027	2.92E-53	NPPC-2	DACT1	FALSE	FALSE
4.96E-57	0.761971309	0.559	0.256	1.25E-52	NPPC-2	EPHX1	FALSE	FALSE
4.46E-56	0.695417053	0.319	0.065	1.13E-51	NPPC-2	NOTCH3	FALSE	TRUE
1.84E-55	0.807132529	0.472	0.178	4.65E-51	NPPC-2	CAMK2N1	FALSE	FALSE
9.08E-52	0.847537187	0.728	0.505	2.29E-47	NPPC-2	FOSB	TRUE	FALSE
2.16E-51	0.630938993	0.264	0.045	5.46E-47	NPPC-2	PREX2	FALSE	FALSE
3.79E-51	0.590251175	0.21	0.019	9.56E-47	NPPC-2	TMEM176	FALSE	FALSE
5.58E-51	0.845773677	0.524	0.251	1.41E-46	NPPC-2	COL4A1	FALSE	FALSE
1.02E-50	0.858856028	0.767	0.581	2.58E-46	NPPC-2	JUN	TRUE	FALSE
2.14E-50	0.657059193	0.412	0.14	5.41E-46	NPPC-2	ANTXR2	FALSE	TRUE

9.01E-50	0.418887306	0.971	0.963	2.27E-45	NPPC-2	FTH1	FALSE	FALSE
2.98E-49	0.714305933	0.281	0.058	7.53E-45	NPPC-2	ANGPT1	FALSE	FALSE
7.92E-49	0.768955179	0.452	0.183	2.00E-44	NPPC-2	ID4	TRUE	FALSE
1.71E-48	0.780903629	0.761	0.537	4.32E-44	NPPC-2	EGR1	TRUE	FALSE
5.99E-48	0.689534328	0.43	0.162	1.51E-43	NPPC-2	NEGR1	FALSE	TRUE
1.46E-47	0.611740706	0.771	0.593	3.68E-43	NPPC-2	PRRX1	TRUE	FALSE
1.81E-46	0.676268707	0.48	0.198	4.56E-42	NPPC-2	FLRT2	FALSE	TRUE
4.56E-46	0.568975786	0.286	0.064	1.15E-41	NPPC-2	KCNT2	FALSE	FALSE
2.49E-44	0.893666956	0.565	0.284	6.27E-40	NPPC-2	C3	FALSE	FALSE
1.26E-43	0.707424726	0.783	0.608	3.18E-39	NPPC-2	JUNB	TRUE	FALSE
9.18E-43	0.604141032	0.241	0.046	2.32E-38	NPPC-2	CCDC102	FALSE	FALSE
2.92E-42	0.381032003	0.995	0.992	7.36E-38	NPPC-2	MT-RNR2	FALSE	FALSE
4.86E-42	0.743450804	0.617	0.38	1.23E-37	NPPC-2	UACA	FALSE	FALSE
6.72E-41	0.654930081	0.553	0.296	1.69E-36	NPPC-2	LAMA2	FALSE	FALSE
2.29E-40	0.667312213	0.393	0.15	5.77E-36	NPPC-2	LAMB1	FALSE	FALSE
1.00E-39	0.401187029	0.967	0.932	2.52E-35	NPPC-2	MT-RNR1	FALSE	FALSE
7.84E-39	0.649615886	0.234	0.051	1.98E-34	NPPC-2	SYNPO2	FALSE	FALSE
1.57E-38	0.64038144	0.358	0.134	3.97E-34	NPPC-2	CYGB	FALSE	FALSE
9.14E-37	1.410388286	0.289	0.088	2.31E-32	NPPC-2	CXCL14	FALSE	FALSE
2.03E-36	0.286958943	0.996	0.997	5.12E-32	NPPC-2	VIM	FALSE	FALSE
2.75E-36	0.502909679	0.808	0.594	6.93E-32	NPPC-2	COL14A1	FALSE	FALSE
4.34E-35	0.538942692	0.29	0.09	1.10E-30	NPPC-2	ABCC9	FALSE	TRUE
1.10E-34	0.551282368	0.248	0.066	2.79E-30	NPPC-2	SLC7A2	FALSE	TRUE
2.54E-34	0.606981233	0.475	0.242	6.41E-30	NPPC-2	PPP1R15A	FALSE	FALSE
3.65E-34	0.546833553	0.254	0.071	9.21E-30	NPPC-2	PDE5A	FALSE	FALSE
4.54E-34	0.551654303	0.472	0.229	1.15E-29	NPPC-2	RARRES2	FALSE	FALSE
1.65E-33	0.554160348	0.319	0.111	4.17E-29	NPPC-2	AHR	TRUE	FALSE
1.35E-32	0.514391348	0.848	0.729	3.42E-28	NPPC-2	ZFP36L1	FALSE	FALSE
1.36E-32	0.572604747	0.539	0.317	3.44E-28	NPPC-2	EPS8	FALSE	FALSE
4.05E-32	0.669432117	0.627	0.425	1.02E-27	NPPC-2	ZFP36	FALSE	FALSE
5.06E-32	0.543008941	0.422	0.197	1.28E-27	NPPC-2	SRPX	FALSE	FALSE
2.94E-31	0.477101278	0.237	0.068	7.43E-27	NPPC-2	TMEM204	FALSE	TRUE
2.96E-31	0.531012688	0.354	0.142	7.47E-27	NPPC-2	HTRA3	FALSE	FALSE
8.03E-31	0.516361521	0.74	0.604	2.03E-26	NPPC-2	SPTBN1	FALSE	FALSE
5.61E-30	0.525444281	0.566	0.35	1.42E-25	NPPC-2	BOC	FALSE	TRUE
1.35E-29	0.588863935	0.301	0.111	3.41E-25	NPPC-2	SLC2A3	FALSE	TRUE
5.10E-29	0.460220345	0.718	0.565	1.29E-24	NPPC-2	IFI16	FALSE	FALSE
1.41E-28	0.25873091	0.973	0.96	3.55E-24	NPPC-2	ITM2B	FALSE	TRUE
3.17E-28	0.563481033	0.582	0.383	8.01E-24	NPPC-2	TAGLN	FALSE	FALSE
7.07E-28	0.391037167	0.204	0.052	1.78E-23	NPPC-2	STXBP6	FALSE	FALSE
8.98E-28	0.561040993	0.435	0.241	2.27E-23	NPPC-2	COL4A2	FALSE	FALSE
3.09E-27	0.531805294	0.497	0.287	7.81E-23	NPPC-2	PODN	FALSE	FALSE
5.46E-27	0.546119804	0.356	0.167	1.38E-22	NPPC-2	SVEP1	FALSE	FALSE
6.24E-27	0.469311744	0.358	0.162	1.57E-22	NPPC-2	LGALS3BP	FALSE	FALSE
6.43E-27	0.540496802	0.541	0.353	1.62E-22	NPPC-2	CD302	FALSE	TRUE
6.84E-27	0.316262023	0.973	0.972	1.73E-22	NPPC-2	MT-ND3	FALSE	FALSE
4.40E-26	0.537481385	0.651	0.491	1.11E-21	NPPC-2	EMP1	FALSE	TRUE
1.09E-25	0.538134464	0.367	0.183	2.75E-21	NPPC-2	GHR	FALSE	TRUE
1.76E-25	0.509657382	0.618	0.442	4.45E-21	NPPC-2	ARID5B	TRUE	FALSE
1.98E-25	0.484322035	0.379	0.191	4.99E-21	NPPC-2	ETS1	TRUE	FALSE
3.76E-25	0.463459587	0.561	0.385	9.49E-21	NPPC-2	ITM2A	FALSE	FALSE
6.58E-24	0.440643995	0.314	0.14	1.66E-19	NPPC-2	SERPINE2	FALSE	FALSE
8.00E-24	0.475963701	0.269	0.109	2.02E-19	NPPC-2	ARHGAP6	FALSE	FALSE
9.53E-24	0.455533157	0.29	0.124	2.41E-19	NPPC-2	PHLDA3	FALSE	FALSE
5.81E-23	0.547097952	0.29	0.126	1.47E-18	NPPC-2	HES1	TRUE	FALSE
6.43E-23	0.450024614	0.545	0.351	1.62E-18	NPPC-2	IGFBP4	FALSE	FALSE
7.15E-23	0.31488004	0.931	0.912	1.80E-18	NPPC-2	MT-ND1	FALSE	FALSE
7.77E-23	0.501620527	0.609	0.457	1.96E-18	NPPC-2	PDGFRB	FALSE	TRUE
1.68E-22	0.264350838	0.954	0.956	4.24E-18	NPPC-2	MT-ND2	FALSE	FALSE
2.80E-22	0.51284242	0.417	0.235	7.07E-18	NPPC-2	CELF2	FALSE	FALSE
3.79E-22	0.416509471	0.285	0.122	9.56E-18	NPPC-2	PHLDB2	FALSE	FALSE
1.27E-21	0.412733206	0.533	0.346	3.20E-17	NPPC-2	CDH11	FALSE	TRUE
1.84E-21	0.468102315	0.362	0.194	4.65E-17	NPPC-2	EPHA3	FALSE	TRUE
5.66E-21	0.50028493	0.549	0.397	1.43E-16	NPPC-2	DDR2	FALSE	TRUE
1.29E-20	0.699724845	0.353	0.19	3.25E-16	NPPC-2	SOX4	TRUE	FALSE
1.68E-20	0.401473379	0.339	0.172	4.24E-16	NPPC-2	ZCCHC24	FALSE	FALSE
2.91E-20	0.416628308	0.642	0.498	7.34E-16	NPPC-2	NAP1L1	TRUE	FALSE
1.04E-18	0.408089051	0.254	0.113	2.62E-14	NPPC-2	SAMHD1	FALSE	FALSE
1.21E-18	0.435816329	0.373	0.218	3.05E-14	NPPC-2	PALLD	FALSE	FALSE
1.23E-18	0.402357655	0.281	0.133	3.11E-14	NPPC-2	BASP1	FALSE	FALSE
3.86E-18	0.253014658	0.98	0.982	9.75E-14	NPPC-2	MT-ATP6	FALSE	FALSE
5.86E-17	0.422673698	0.303	0.157	1.48E-12	NPPC-2	EDIL3	FALSE	FALSE
6.10E-17	0.362072066	0.681	0.596	1.54E-12	NPPC-2	NPM1	FALSE	FALSE
8.16E-17	0.440632539	0.507	0.367	2.06E-12	NPPC-2	SASH1	FALSE	FALSE
1.08E-16	0.398288561	0.356	0.205	2.73E-12	NPPC-2	TNRC6C	FALSE	FALSE
1.94E-16	0.372121861	0.229	0.103	4.89E-12	NPPC-2	ENG	FALSE	TRUE
5.37E-16	0.381710439	0.305	0.166	1.35E-11	NPPC-2	ADAMTSL	FALSE	FALSE
6.01E-16	0.454223214	0.4	0.252	1.52E-11	NPPC-2	MAFB	TRUE	FALSE
1.24E-15	0.390407764	0.663	0.541	3.12E-11	NPPC-2	EBF1	TRUE	FALSE
2.46E-15	0.392590467	0.299	0.165	6.20E-11	NPPC-2	ENAH	FALSE	FALSE
3.01E-15	0.704935352	0.209	0.094	7.60E-11	NPPC-2	LEPR	FALSE	TRUE
5.91E-15	0.406891891	0.384	0.246	1.49E-10	NPPC-2	PTPRG	FALSE	TRUE
6.00E-15	0.262821027	0.923	0.912	1.51E-10	NPPC-2	RPL5	FALSE	FALSE
6.85E-15	0.339836624	0.241	0.115	1.73E-10	NPPC-2	NID1	FALSE	FALSE
9.81E-15	0.474613903	0.353	0.223	2.48E-10	NPPC-2	VMP1	FALSE	FALSE
1.01E-14	0.319789276	0.731	0.668	2.54E-10	NPPC-2	NFIA	TRUE	FALSE
1.17E-14	0.40625648	0.341	0.208	2.96E-10	NPPC-2	TNFSF10	FALSE	FALSE
1.20E-14	0.357826482	0.695	0.601	3.02E-10	NPPC-2	NFIB	TRUE	FALSE
1.57E-14	0.413988109	0.396	0.251	3.95E-10	NPPC-2	RUNX1	TRUE	FALSE

1.62E-14	0.376891981	0.477	0.344	4.10E-10	NPPC-2	CCNL1	FALSE	FALSE
1.67E-14	0.291662546	0.208	0.093	4.21E-10	NPPC-2	FAM102B	FALSE	FALSE
2.56E-14	0.388266893	0.264	0.141	6.45E-10	NPPC-2	SULF1	FALSE	FALSE
4.70E-14	0.411431852	0.576	0.473	1.19E-09	NPPC-2	COLEC12	FALSE	FALSE
5.50E-14	0.358051749	0.269	0.147	1.39E-09	NPPC-2	PLEKHA5	FALSE	FALSE
1.14E-13	0.381575228	0.366	0.236	2.86E-09	NPPC-2	AFF1	FALSE	FALSE
1.54E-13	0.481946358	0.903	0.841	3.89E-09	NPPC-2	HSPA1A	FALSE	FALSE
1.62E-13	0.380093891	0.465	0.339	4.08E-09	NPPC-2	GCC2	FALSE	FALSE
3.12E-13	0.351196974	0.244	0.128	7.87E-09	NPPC-2	FZD4	FALSE	TRUE
8.58E-13	0.363941242	0.41	0.282	2.17E-08	NPPC-2	IRF2BP2	FALSE	FALSE
1.60E-12	0.401390063	0.38	0.257	4.04E-08	NPPC-2	PCDH18	FALSE	TRUE
2.03E-12	0.461267922	0.507	0.384	5.12E-08	NPPC-2	IER2	FALSE	FALSE
2.11E-12	0.355221317	0.244	0.135	5.32E-08	NPPC-2	NET1	FALSE	FALSE
2.14E-12	0.363277973	0.671	0.577	5.39E-08	NPPC-2	TIMP3	FALSE	FALSE
2.49E-12	0.281288242	0.808	0.77	6.28E-08	NPPC-2	AHNAK	FALSE	FALSE
2.72E-12	0.306555758	0.226	0.117	6.86E-08	NPPC-2	AP001528	FALSE	FALSE
2.75E-12	0.272091724	0.684	0.611	6.93E-08	NPPC-2	RPL4	FALSE	FALSE
3.06E-12	0.30336714	0.29	0.168	7.72E-08	NPPC-2	DCLK1	FALSE	FALSE
3.38E-12	0.28249104	0.204	0.098	8.53E-08	NPPC-2	KRT222	FALSE	FALSE
3.43E-12	0.299908981	0.345	0.218	8.66E-08	NPPC-2	SYNE3	FALSE	FALSE
3.84E-12	0.379216846	0.814	0.774	9.69E-08	NPPC-2	HSP90AA1	FALSE	FALSE
4.12E-12	0.329516554	0.563	0.455	1.04E-07	NPPC-2	LPP	FALSE	FALSE
7.62E-12	0.354483081	0.237	0.128	1.92E-07	NPPC-2	SLIT2	FALSE	FALSE
1.06E-11	0.396283009	0.407	0.289	2.67E-07	NPPC-2	FGL2	FALSE	FALSE
1.36E-11	0.450578624	0.426	0.309	3.44E-07	NPPC-2	SOCS3	FALSE	FALSE
1.50E-11	0.28087982	0.782	0.708	3.78E-07	NPPC-2	LTBP4	FALSE	FALSE
2.14E-11	0.347110567	0.25	0.141	5.41E-07	NPPC-2	RAB31	FALSE	FALSE
3.13E-11	0.322029772	0.51	0.399	7.89E-07	NPPC-2	PLAGL1	TRUE	FALSE
4.08E-11	0.458926703	0.271	0.162	1.03E-06	NPPC-2	SPON2	FALSE	FALSE
5.12E-11	0.353378656	0.576	0.481	1.29E-06	NPPC-2	DAB2	TRUE	FALSE
6.46E-11	0.321962565	0.505	0.4	1.63E-06	NPPC-2	SDC2	FALSE	TRUE
8.88E-11	0.278965209	0.289	0.177	2.24E-06	NPPC-2	CLEC11A	FALSE	FALSE
9.69E-11	0.366207574	0.237	0.136	2.44E-06	NPPC-2	EPAS1	TRUE	FALSE
1.82E-10	0.345592699	0.284	0.18	4.58E-06	NPPC-2	MITF	TRUE	FALSE
1.90E-10	0.370127381	0.25	0.151	4.80E-06	NPPC-2	NFIL3	TRUE	FALSE
3.02E-10	0.376326186	0.275	0.17	7.61E-06	NPPC-2	NAV1	FALSE	FALSE
4.07E-10	0.444077204	0.512	0.421	1.03E-05	NPPC-2	COL15A1	FALSE	FALSE
5.65E-10	0.288121643	0.395	0.278	1.43E-05	NPPC-2	LIMS1	FALSE	FALSE
5.83E-10	0.329388972	0.397	0.275	1.47E-05	NPPC-2	DEPP1	FALSE	FALSE
7.55E-10	0.324815659	0.361	0.254	1.90E-05	NPPC-2	MYCBP2	FALSE	FALSE
1.04E-09	0.358033074	0.459	0.356	2.63E-05	NPPC-2	SNED1	FALSE	FALSE
1.22E-09	0.489378754	0.297	0.198	3.08E-05	NPPC-2	NR4A1	TRUE	FALSE
1.66E-09	0.342281279	0.297	0.195	4.19E-05	NPPC-2	MAP1A	FALSE	FALSE
1.98E-09	0.292819138	0.315	0.211	4.99E-05	NPPC-2	SIPA1L1	FALSE	FALSE
2.00E-09	0.260085834	0.336	0.231	5.05E-05	NPPC-2	ARHGAP2	FALSE	FALSE
2.57E-09	0.32275596	0.358	0.252	6.50E-05	NPPC-2	PLTP	FALSE	FALSE
4.24E-09	0.257439441	0.708	0.647	0.000106891	NPPC-2	WSB1	FALSE	FALSE
4.38E-09	0.337900013	0.481	0.396	0.000110468	NPPC-2	AKAP13	FALSE	FALSE
4.80E-09	0.317651912	0.332	0.233	0.000121136	NPPC-2	C6orf48	FALSE	FALSE
5.26E-09	0.346453566	0.484	0.39	0.000132678	NPPC-2	SRSF7	FALSE	FALSE
5.81E-09	0.289539764	0.478	0.38	0.000146587	NPPC-2	NSA2	FALSE	FALSE
9.13E-09	0.31002622	0.264	0.17	0.000230465	NPPC-2	FBLN5	FALSE	FALSE
1.02E-08	0.31354603	0.225	0.135	0.000256618	NPPC-2	MIDN	FALSE	FALSE
1.04E-08	0.278356615	0.301	0.205	0.000262481	NPPC-2	ARHGAP1	FALSE	FALSE
1.18E-08	0.278005157	0.21	0.123	0.000297726	NPPC-2	GAB1	FALSE	FALSE
1.36E-08	0.286849539	0.286	0.19	0.000342518	NPPC-2	KANK2	FALSE	FALSE
1.61E-08	0.293723676	0.378	0.28	0.000406457	NPPC-2	CAMK2D	FALSE	FALSE
2.39E-08	0.30813803	0.518	0.44	0.000604326	NPPC-2	KMT2E	FALSE	FALSE
3.58E-08	0.597130418	0.524	0.441	0.00090307	NPPC-2	DNAJB1	FALSE	FALSE
3.72E-08	0.260664785	0.221	0.135	0.000938681	NPPC-2	SIX1	TRUE	FALSE
5.09E-08	0.252847363	0.459	0.368	0.001284677	NPPC-2	MAN1A1	FALSE	FALSE
6.04E-08	0.265058347	0.341	0.242	0.001523125	NPPC-2	PLSCR1	TRUE	FALSE
6.72E-08	0.296384553	0.28	0.19	0.001694538	NPPC-2	RNF146	FALSE	FALSE
6.75E-08	0.359448305	0.275	0.19	0.001702396	NPPC-2	B4GALT1	FALSE	FALSE
7.58E-08	0.277337686	0.422	0.338	0.001913109	NPPC-2	SNX9	FALSE	FALSE
8.74E-08	0.291925357	0.275	0.188	0.002205472	NPPC-2	BHLHE41	TRUE	FALSE
1.29E-07	0.270107917	0.498	0.421	0.003260468	NPPC-2	PLSCR4	FALSE	FALSE
1.30E-07	0.281636082	0.294	0.205	0.003286492	NPPC-2	PHACTR2	FALSE	FALSE
2.11E-07	0.278229929	0.37	0.281	0.005326641	NPPC-2	MMP14	FALSE	TRUE
2.23E-07	0.255167551	0.386	0.292	0.00562147	NPPC-2	IL1R1	FALSE	TRUE
2.41E-07	0.292737606	0.35	0.261	0.006089545	NPPC-2	OMD	FALSE	FALSE
2.42E-07	0.254361038	0.224	0.144	0.006110796	NPPC-2	LTBR	FALSE	TRUE
2.59E-07	0.284350349	0.371	0.287	0.006541774	NPPC-2	ANKRD11	FALSE	FALSE
3.40E-07	0.297277004	0.392	0.313	0.008567613	NPPC-2	NSD3	FALSE	FALSE
3.91E-07	0.266680231	0.349	0.261	0.009865136	NPPC-2	KCTD12	FALSE	FALSE
4.14E-07	0.264279351	0.204	0.128	0.010456335	NPPC-2	A4GALT	FALSE	FALSE
4.39E-07	0.27503569	0.442	0.364	0.011082702	NPPC-2	PNN	FALSE	FALSE
9.60E-07	0.269850692	0.214	0.139	0.024219507	NPPC-2	GOLGA8A	FALSE	FALSE
1.10E-06	0.292670159	0.431	0.351	0.027771022	NPPC-2	LMO4	FALSE	FALSE
1.76E-06	0.273914548	0.314	0.234	0.044294508	NPPC-2	MYH9	FALSE	FALSE
#######	2.439634984	0.702	0.153	1.72E-123	NPPC-3	PRG4	FALSE	FALSE
#######	1.642907715	0.952	0.637	2.84E-96	NPPC-3	PLA2G2A	FALSE	FALSE
3.03E-79	1.01251202	0.881	0.439	7.65E-75	NPPC-3	PCOLCE2	FALSE	FALSE
1.04E-70	1.352115624	0.426	0.08	2.63E-66	NPPC-3	CRTAC1	FALSE	FALSE
1.36E-66	0.96681109	0.854	0.421	3.44E-62	NPPC-3	TNXB	FALSE	FALSE
2.29E-59	0.53043001	0.277	0.031	5.79E-55	NPPC-3	DPP4	FALSE	TRUE
2.47E-59	0.964307095	0.423	0.093	6.23E-55	NPPC-3	CRLF1	FALSE	FALSE
1.03E-55	0.525685411	0.214	0.016	2.60E-51	NPPC-3	DEFB1	FALSE	FALSE
1.00E-51	0.732351265	0.97	0.793	2.53E-47	NPPC-3	ABI3BP	FALSE	FALSE

1.37E-47	0.736617569	0.652	0.267	3.45E-43	NPPC-3	SEMA3C	FALSE	FALSE
2.02E-46	0.752473566	0.396	0.099	5.09E-42	NPPC-3	MFAP5	FALSE	FALSE
3.70E-44	0.787578237	0.58	0.228	9.33E-40	NPPC-3	NOVA1	FALSE	FALSE
1.86E-42	0.759763159	0.911	0.645	4.70E-38	NPPC-3	CCDC80	FALSE	FALSE
3.26E-40	0.717359345	0.461	0.158	8.23E-36	NPPC-3	BCAT1	FALSE	FALSE
7.63E-40	0.835679171	0.53	0.211	1.93E-35	NPPC-3	DPT	FALSE	FALSE
1.35E-39	0.783766263	0.577	0.251	3.40E-35	NPPC-3	PROCR	FALSE	TRUE
1.91E-37	0.673948192	0.688	0.346	4.81E-33	NPPC-3	FBLN2	FALSE	FALSE
8.94E-35	0.479741794	0.988	0.873	2.26E-30	NPPC-3	C1R	FALSE	FALSE
1.81E-33	0.685026782	0.634	0.316	4.57E-29	NPPC-3	ACKR3	FALSE	TRUE
1.48E-32	0.48246552	0.923	0.693	3.73E-28	NPPC-3	VCAN	FALSE	FALSE
2.53E-32	0.429481317	0.97	0.837	6.39E-28	NPPC-3	LRP1	FALSE	TRUE
8.49E-32	0.551364755	0.443	0.164	2.14E-27	NPPC-3	SEMA3E	FALSE	FALSE
1.64E-31	0.655401564	0.464	0.183	4.14E-27	NPPC-3	GALNT15	FALSE	FALSE
8.41E-29	0.700603112	0.637	0.343	2.12E-24	NPPC-3	SFRP2	FALSE	FALSE
2.18E-28	0.613267424	0.438	0.176	5.51E-24	NPPC-3	SCARA5	FALSE	TRUE
3.73E-27	0.629729809	0.685	0.407	9.42E-23	NPPC-3	CDO1	FALSE	FALSE
4.96E-25	0.443362303	0.967	0.848	1.25E-20	NPPC-3	CD9	FALSE	TRUE
4.03E-24	0.583079842	0.357	0.135	1.02E-19	NPPC-3	TNFRSF11	FALSE	FALSE
8.20E-24	0.43460434	0.557	0.278	2.07E-19	NPPC-3	RETREG1	FALSE	FALSE
5.93E-23	0.447929385	0.741	0.485	1.50E-18	NPPC-3	FSTL1	FALSE	FALSE
1.21E-22	0.416050151	0.327	0.12	3.06E-18	NPPC-3	RERG	FALSE	FALSE
2.44E-22	0.380222542	0.286	0.094	6.17E-18	NPPC-3	CREB5	TRUE	FALSE
2.45E-22	0.403652282	0.304	0.105	6.19E-18	NPPC-3	PRSS23	FALSE	FALSE
6.72E-22	0.49224842	0.673	0.407	1.70E-17	NPPC-3	FBN1	FALSE	FALSE
7.72E-22	0.462491034	0.348	0.135	1.95E-17	NPPC-3	SLC39A14	FALSE	TRUE
4.72E-21	0.345627595	0.259	0.082	1.19E-16	NPPC-3	PRUNE2	FALSE	FALSE
2.78E-20	0.395574826	0.562	0.303	7.02E-16	NPPC-3	FXYD5	FALSE	FALSE
4.81E-20	0.377050908	0.241	0.076	1.21E-15	NPPC-3	CLEC3B	FALSE	FALSE
8.84E-20	0.443645884	0.658	0.405	2.23E-15	NPPC-3	SLC25A37	FALSE	FALSE
9.62E-20	0.421439958	0.973	0.93	2.43E-15	NPPC-3	LUM	FALSE	FALSE
2.28E-19	0.385822294	0.327	0.13	5.75E-15	NPPC-3	ITGA11	FALSE	TRUE
2.37E-18	0.880492575	0.366	0.166	5.98E-14	NPPC-3	MT1G	FALSE	FALSE
3.54E-18	0.455351138	0.75	0.517	8.93E-14	NPPC-3	TENT5A	FALSE	FALSE
3.85E-18	0.46112103	0.726	0.49	9.70E-14	NPPC-3	HTRA1	FALSE	FALSE
5.34E-18	0.401855425	0.396	0.186	1.35E-13	NPPC-3	AHNAK2	FALSE	FALSE
1.90E-17	0.426548218	0.732	0.525	4.79E-13	NPPC-3	SH3BGRL3	FALSE	FALSE
2.38E-16	0.368047594	0.295	0.121	6.02E-12	NPPC-3	NTN4	FALSE	FALSE
3.47E-16	0.515819117	0.22	0.077	8.76E-12	NPPC-3	CPB1	FALSE	FALSE
4.96E-16	0.342884095	0.327	0.144	1.25E-11	NPPC-3	PDE1A	FALSE	FALSE
1.05E-15	0.363589399	0.438	0.225	2.65E-11	NPPC-3	NT5E	FALSE	TRUE
2.14E-15	0.317540842	0.673	0.427	5.39E-11	NPPC-3	CD44	FALSE	TRUE
5.07E-15	0.382673164	0.423	0.22	1.28E-10	NPPC-3	SLC16A7	FALSE	TRUE
6.82E-15	0.325792375	0.756	0.527	1.72E-10	NPPC-3	NDUFA4L2	FALSE	FALSE
5.64E-14	0.295461485	0.277	0.12	1.42E-09	NPPC-3	CAMK1D	FALSE	FALSE
8.31E-14	0.308279838	0.408	0.215	2.10E-09	NPPC-3	PIGT	FALSE	TRUE
2.86E-13	0.366131749	0.622	0.41	7.23E-09	NPPC-3	ISLR	FALSE	FALSE
3.55E-13	0.269855285	0.551	0.34	8.95E-09	NPPC-3	SMIM14	FALSE	FALSE
5.99E-13	0.321816953	0.443	0.253	1.51E-08	NPPC-3	MEDAG	FALSE	FALSE
6.12E-13	0.290852914	0.762	0.533	1.54E-08	NPPC-3	FHL1	FALSE	FALSE
8.02E-13	0.271280299	0.515	0.308	2.02E-08	NPPC-3	OS9	FALSE	FALSE
8.34E-13	0.394906803	0.47	0.278	2.10E-08	NPPC-3	FAP	FALSE	TRUE
1.23E-12	0.322910561	0.387	0.21	3.09E-08	NPPC-3	PLPP1	FALSE	FALSE
1.52E-12	0.316734405	0.86	0.707	3.84E-08	NPPC-3	CYBRD1	FALSE	FALSE
9.25E-12	0.253499929	0.452	0.259	2.33E-07	NPPC-3	ABLIM1	FALSE	FALSE
1.43E-11	0.417656367	0.693	0.495	3.62E-07	NPPC-3	SOD2	FALSE	FALSE
1.69E-11	0.309775666	0.381	0.213	4.26E-07	NPPC-3	FKBP10	FALSE	FALSE
1.73E-11	0.287800063	0.256	0.117	4.36E-07	NPPC-3	PDLIM3	FALSE	FALSE
2.52E-11	0.283795811	0.274	0.131	6.35E-07	NPPC-3	FYN	FALSE	FALSE
3.05E-11	0.361547839	0.488	0.314	7.71E-07	NPPC-3	TCF7L2	TRUE	FALSE
4.47E-11	0.31306399	0.863	0.696	1.13E-06	NPPC-3	DST	FALSE	FALSE
4.56E-11	0.378548394	0.53	0.336	1.15E-06	NPPC-3	AKR1C1	FALSE	FALSE
8.56E-11	0.327193493	0.324	0.176	2.16E-06	NPPC-3	PSD3	FALSE	FALSE
1.13E-10	0.264230825	0.399	0.224	2.84E-06	NPPC-3	NFE2L1	TRUE	FALSE
1.94E-10	0.301128142	0.414	0.243	4.88E-06	NPPC-3	MFAP4	FALSE	FALSE
1.96E-10	0.266947442	0.223	0.1	4.94E-06	NPPC-3	AGAP1	FALSE	FALSE
2.45E-10	0.370846234	0.83	0.716	6.19E-06	NPPC-3	MMP2	FALSE	FALSE
3.18E-10	0.525601662	0.815	0.672	8.04E-06	NPPC-3	COL3A1	FALSE	FALSE
4.50E-10	0.282420052	0.446	0.283	1.14E-05	NPPC-3	UGP2	TRUE	FALSE
5.17E-10	0.311262141	0.369	0.212	1.31E-05	NPPC-3	AKR1C2	FALSE	FALSE
9.19E-10	0.260112268	0.917	0.815	2.32E-05	NPPC-3	S100A10	FALSE	FALSE
6.61E-09	0.266750887	0.804	0.682	0.000166849	NPPC-3	HLA-C	FALSE	TRUE
7.20E-09	0.282556944	0.783	0.647	0.000181619	NPPC-3	PDGFRL	FALSE	FALSE
9.70E-09	0.322341226	0.5	0.334	0.000244679	NPPC-3	REV3L	FALSE	FALSE
1.91E-08	0.256618173	0.366	0.224	0.000482771	NPPC-3	PPIC	FALSE	FALSE
2.68E-08	0.255931374	0.688	0.528	0.000675075	NPPC-3	PCOLCE	FALSE	FALSE
3.00E-08	0.270458775	0.396	0.247	0.000757226	NPPC-3	KLF4	TRUE	FALSE
4.04E-08	0.262667231	0.345	0.208	0.001018869	NPPC-3	ANGPTL2	FALSE	FALSE
#######	2.008821462	0.71	0.015	1.55E-272	NPPC-4	CDH19	FALSE	TRUE
#######	0.986803912	0.412	0.004	1.43E-161	NPPC-4	TENM2	FALSE	TRUE
#######	2.951581478	1	0.64	5.47E-151	NPPC-4	APOD	FALSE	FALSE
#######	1.071228484	0.423	0.01	9.20E-151	NPPC-4	CLDN1	FALSE	TRUE
#######	1.811238104	0.735	0.124	8.28E-142	NPPC-4	MIA	FALSE	FALSE
#######	1.459904153	0.667	0.12	1.08E-116	NPPC-4	SOX9	TRUE	FALSE
#######	1.170406886	0.373	0.017	2.45E-113	NPPC-4	FOXS1	TRUE	FALSE
#######	1.05415839	0.276	0.001	9.65E-111	NPPC-4	GJB2	FALSE	TRUE
#######	1.168507984	0.487	0.049	1.59E-109	NPPC-4	SLC12A2	FALSE	TRUE
#######	1.061744973	0.513	0.057	1.66E-108	NPPC-4	PHLDA1	FALSE	FALSE
#######	1.321390529	0.821	0.231	2.51E-108	NPPC-4	VIT	FALSE	FALSE

#######	1.264986341	0.649	0.116	1.66E-106	NPPC-4	EBF2	TRUE	FALSE
#######	1.192501066	0.767	0.168	1.94E-105	NPPC-4	ABCA10	FALSE	FALSE
#######	0.943848355	0.362	0.018	2.89E-104	NPPC-4	L1TD1	FALSE	FALSE
#######	0.913635685	0.362	0.019	4.95E-104	NPPC-4	TNFRSF19	FALSE	TRUE
#######	1.200167776	0.466	0.048	1.94E-102	NPPC-4	DUSP6	FALSE	FALSE
#######	0.861033371	0.376	0.024	5.28E-99	NPPC-4	TIAM1	FALSE	FALSE
5.65E-97	0.71942281	0.301	0.012	1.43E-92	NPPC-4	CHN1	FALSE	FALSE
8.31E-93	1.117309945	0.982	0.849	2.10E-88	NPPC-4	LGALS1	FALSE	FALSE
1.01E-92	0.883646968	0.312	0.016	2.56E-88	NPPC-4	ITGB4	FALSE	TRUE
2.08E-88	0.768384311	0.319	0.02	5.24E-84	NPPC-4	C3orf70	FALSE	FALSE
1.09E-87	0.717649986	0.297	0.015	2.75E-83	NPPC-4	TSPAN5	FALSE	TRUE
2.23E-87	1.024919073	0.448	0.06	5.63E-83	NPPC-4	MRAS	FALSE	FALSE
6.04E-83	0.618023871	0.251	0.009	1.53E-78	NPPC-4	MCTP1	FALSE	FALSE
2.34E-82	0.56824732	0.201	0.001	5.92E-78	NPPC-4	WFDC1	FALSE	FALSE
3.04E-82	0.873173038	0.373	0.039	7.67E-78	NPPC-4	SBSPON	FALSE	FALSE
3.21E-82	0.71059029	0.305	0.02	8.10E-78	NPPC-4	LZTS1	FALSE	FALSE
5.08E-80	0.883304364	0.348	0.034	1.28E-75	NPPC-4	FOXD2	TRUE	FALSE
4.82E-78	1.001479551	0.975	0.88	1.22E-73	NPPC-4	CD63	FALSE	TRUE
7.77E-77	0.876307579	0.323	0.029	1.96E-72	NPPC-4	FRMD4A	FALSE	FALSE
6.51E-75	0.810941825	0.391	0.051	1.64E-70	NPPC-4	9-Sep	FALSE	FALSE
2.24E-74	1.208598745	0.835	0.488	5.66E-70	NPPC-4	TXN	FALSE	FALSE
1.66E-73	0.846425098	0.423	0.065	4.18E-69	NPPC-4	LPCAT2	FALSE	FALSE
1.70E-71	0.708298827	0.326	0.033	4.30E-67	NPPC-4	PRKCA	FALSE	FALSE
1.29E-70	1.063464493	0.928	0.758	3.25E-66	NPPC-4	PLAC9	FALSE	FALSE
4.05E-70	0.601773552	0.233	0.011	1.02E-65	NPPC-4	SOX8	TRUE	FALSE
3.35E-69	0.919661431	0.409	0.063	8.45E-65	NPPC-4	AC009041	FALSE	FALSE
5.63E-69	0.771950864	0.975	0.927	1.42E-64	NPPC-4	PTMA	FALSE	FALSE
1.65E-68	1.045220304	0.541	0.132	4.17E-64	NPPC-4	NRP2	FALSE	TRUE
4.40E-68	0.954326438	0.358	0.048	1.11E-63	NPPC-4	CC2D1A	TRUE	FALSE
5.60E-68	0.863113187	0.398	0.062	1.41E-63	NPPC-4	ETV1	TRUE	FALSE
8.11E-66	0.724240619	0.315	0.035	2.05E-61	NPPC-4	PLK2	FALSE	FALSE
1.65E-65	1.035490739	0.516	0.126	4.17E-61	NPPC-4	CHSY1	FALSE	FALSE
2.32E-65	0.901944032	0.315	0.035	5.85E-61	NPPC-4	EGR3	TRUE	FALSE
3.15E-65	0.49004606	0.215	0.01	7.95E-61	NPPC-4	AFAP1L2	FALSE	FALSE
2.23E-64	1.135899111	0.728	0.324	5.62E-60	NPPC-4	TPM4	FALSE	FALSE
1.02E-63	0.734582833	0.33	0.043	2.57E-59	NPPC-4	PODNL1	FALSE	FALSE
8.04E-63	0.576271328	0.201	0.009	2.03E-58	NPPC-4	DUSP5	FALSE	FALSE
3.45E-60	0.566380224	0.265	0.024	8.70E-56	NPPC-4	SPRY4	FALSE	FALSE
9.35E-60	0.787889411	0.287	0.031	2.36E-55	NPPC-4	MARCKSL	FALSE	FALSE
4.45E-59	0.900941265	0.746	0.268	1.12E-54	NPPC-4	A2M	FALSE	FALSE
1.93E-57	0.914817545	0.968	0.922	4.88E-53	NPPC-4	TMSB10	FALSE	FALSE
2.99E-57	0.57958091	0.244	0.021	7.54E-53	NPPC-4	CAPN5	FALSE	FALSE
6.10E-57	0.713175779	0.244	0.022	1.54E-52	NPPC-4	FZD2	FALSE	TRUE
2.85E-56	0.997081002	0.62	0.222	7.20E-52	NPPC-4	EGFR	FALSE	TRUE
1.50E-53	0.697244317	0.308	0.045	3.78E-49	NPPC-4	EFNB1	FALSE	TRUE
4.25E-53	0.849956447	0.509	0.134	1.07E-48	NPPC-4	ABCA9	FALSE	TRUE
1.23E-52	0.78979226	0.968	0.898	3.11E-48	NPPC-4	ACTB	FALSE	FALSE
2.80E-52	0.875551973	0.477	0.126	7.06E-48	NPPC-4	TGFBI	FALSE	FALSE
3.62E-52	0.831732602	0.441	0.105	9.14E-48	NPPC-4	PKDCC	FALSE	FALSE
9.40E-52	0.841388065	0.953	0.841	2.37E-47	NPPC-4	TCF4	TRUE	FALSE
2.21E-51	0.629016997	0.975	0.907	5.58E-47	NPPC-4	H3F3B	FALSE	FALSE
4.14E-50	0.654231759	0.219	0.02	1.05E-45	NPPC-4	SHISA3	FALSE	FALSE
3.79E-49	0.821574253	0.401	0.093	9.55E-45	NPPC-4	GAS7	FALSE	FALSE
9.49E-49	0.508247	0.254	0.031	2.39E-44	NPPC-4	PGF	FALSE	FALSE
3.07E-48	0.586209348	0.262	0.034	7.74E-44	NPPC-4	TNFRSF12	FALSE	FALSE
3.31E-48	0.82509168	0.416	0.101	8.35E-44	NPPC-4	PTCH1	FALSE	TRUE
5.18E-47	0.904239952	0.466	0.136	1.31E-42	NPPC-4	PEAK1	FALSE	FALSE
1.25E-46	0.458691412	0.204	0.019	3.16E-42	NPPC-4	RASL12	FALSE	FALSE
1.95E-45	0.880991399	0.416	0.109	4.92E-41	NPPC-4	SLC20A1	FALSE	FALSE
3.34E-45	0.611801539	0.996	0.98	8.43E-41	NPPC-4	S100A6	FALSE	FALSE
1.31E-44	0.854870407	0.423	0.109	3.30E-40	NPPC-4	IFI27	FALSE	FALSE
1.78E-44	0.653388118	0.373	0.083	4.49E-40	NPPC-4	COL9A3	FALSE	FALSE
2.19E-44	0.56597217	0.251	0.035	5.52E-40	NPPC-4	BTBD3	FALSE	FALSE
2.16E-43	0.820078794	0.541	0.196	5.44E-39	NPPC-4	ACTN4	FALSE	FALSE
2.44E-43	0.788288188	0.43	0.118	6.15E-39	NPPC-4	INHBA	FALSE	FALSE
7.68E-42	0.607460068	0.297	0.056	1.94E-37	NPPC-4	MOK	FALSE	FALSE
8.36E-42	0.741318528	0.756	0.436	2.11E-37	NPPC-4	CFL1	FALSE	FALSE
1.74E-41	0.493440299	0.211	0.025	4.39E-37	NPPC-4	MICALL2	FALSE	FALSE
2.66E-41	0.878723659	0.552	0.215	6.71E-37	NPPC-4	RND3	FALSE	FALSE
7.83E-40	0.806709411	0.789	0.531	1.98E-35	NPPC-4	COX6C	FALSE	FALSE
1.87E-39	0.731383633	0.24	0.037	4.71E-35	NPPC-4	ITGA6	FALSE	TRUE
4.67E-39	0.481366021	0.229	0.033	1.18E-34	NPPC-4	NES	FALSE	FALSE
1.16E-38	0.834705115	0.738	0.405	2.92E-34	NPPC-4	CYP1B1	FALSE	FALSE
1.26E-38	0.856496803	0.717	0.449	3.18E-34	NPPC-4	TUBA1B	FALSE	FALSE
7.54E-38	0.705860017	0.509	0.179	1.90E-33	NPPC-4	PTBP3	FALSE	FALSE
8.16E-38	0.78106336	0.638	0.307	2.06E-33	NPPC-4	HSPA8	FALSE	FALSE
1.05E-37	0.704690245	0.362	0.096	2.65E-33	NPPC-4	INPP1	FALSE	FALSE
1.21E-37	0.846128744	0.401	0.119	3.06E-33	NPPC-4	OLFML2B	FALSE	FALSE
2.40E-37	0.567487165	0.323	0.072	6.06E-33	NPPC-4	SLIT3	FALSE	FALSE
1.41E-36	0.661149479	0.896	0.739	3.56E-32	NPPC-4	DDX5	FALSE	FALSE
2.70E-36	0.723993789	0.767	0.5	6.81E-32	NPPC-4	HNRNPA1	TRUE	FALSE
4.63E-36	0.63943009	0.33	0.08	1.17E-31	NPPC-4	STARD13	FALSE	FALSE
5.01E-36	0.802011267	0.509	0.196	1.26E-31	NPPC-4	PLXDC1	FALSE	TRUE
1.73E-35	0.536484567	0.244	0.044	4.37E-31	NPPC-4	BAMBI	FALSE	TRUE
2.13E-35	0.628371439	0.384	0.113	5.37E-31	NPPC-4	HNRNPAB	FALSE	FALSE
2.62E-35	0.604742097	0.312	0.073	6.62E-31	NPPC-4	FAM198B	FALSE	FALSE
3.64E-35	0.631231373	0.319	0.076	9.18E-31	NPPC-4	GUCY1B1	FALSE	FALSE
4.25E-35	0.707143335	0.362	0.102	1.07E-30	NPPC-4	LSP1	FALSE	FALSE
4.91E-34	0.473972869	0.229	0.039	1.24E-29	NPPC-4	RASSF2	FALSE	FALSE

6.86E-34	0.654699265	0.387	0.12	1.73E-29	NPPC-4	EZR	TRUE	FALSE
2.25E-33	0.666952669	0.538	0.231	5.67E-29	NPPC-4	LY6E	FALSE	TRUE
2.31E-33	0.659649822	0.875	0.7	5.83E-29	NPPC-4	EID1	FALSE	FALSE
5.15E-33	0.475333862	0.222	0.038	1.30E-28	NPPC-4	TSHZ3	TRUE	FALSE
1.10E-32	0.762946321	0.398	0.132	2.79E-28	NPPC-4	SRM	FALSE	FALSE
2.52E-32	0.741305212	0.323	0.086	6.35E-28	NPPC-4	NGFR	FALSE	TRUE
4.82E-32	0.613090375	0.312	0.078	1.22E-27	NPPC-4	PTCH2	FALSE	FALSE
7.14E-32	0.677978315	0.308	0.08	1.80E-27	NPPC-4	KLF5	TRUE	FALSE
2.10E-31	0.666812259	0.444	0.162	5.30E-27	NPPC-4	AFDN	FALSE	FALSE
4.03E-31	0.657970783	0.523	0.223	1.02E-26	NPPC-4	PHLDB1	FALSE	FALSE
5.73E-31	0.504822006	0.258	0.056	1.45E-26	NPPC-4	PTPN9	FALSE	FALSE
1.62E-30	0.62135636	0.358	0.112	4.09E-26	NPPC-4	AMD1	FALSE	FALSE
2.28E-30	0.60417887	0.81	0.597	5.75E-26	NPPC-4	PTMS	FALSE	FALSE
5.38E-30	0.641058901	0.724	0.458	1.36E-25	NPPC-4	PSMA7	FALSE	FALSE
1.35E-29	0.652073245	0.491	0.202	3.42E-25	NPPC-4	TUBB4B	FALSE	FALSE
1.42E-29	0.806573969	0.778	0.628	3.58E-25	NPPC-4	HSP90B1	FALSE	FALSE
2.57E-29	0.460652283	0.258	0.058	6.49E-25	NPPC-4	PDLIM7	FALSE	FALSE
2.75E-29	0.619241215	0.384	0.129	6.95E-25	NPPC-4	PLEKHA4	FALSE	FALSE
3.86E-29	0.645129416	0.401	0.139	9.74E-25	NPPC-4	SH3PXD2/	FALSE	FALSE
6.82E-29	0.576534165	0.946	0.882	1.72E-24	NPPC-4	ACTG1	FALSE	FALSE
2.18E-27	0.564138945	0.301	0.086	5.49E-23	NPPC-4	DKK3	FALSE	FALSE
1.29E-26	0.483560626	0.215	0.046	3.24E-22	NPPC-4	ARHGAP3	FALSE	FALSE
1.38E-26	0.447478148	0.247	0.06	3.47E-22	NPPC-4	NME1	TRUE	FALSE
1.75E-26	0.706990883	0.498	0.229	4.41E-22	NPPC-4	RNF24	FALSE	FALSE
2.22E-26	0.557777286	0.348	0.114	5.59E-22	NPPC-4	RASSF8-A	FALSE	FALSE
4.06E-26	0.768148983	0.971	0.941	1.02E-21	NPPC-4	TIMP1	FALSE	FALSE
4.38E-26	0.539384542	0.337	0.106	1.11E-21	NPPC-4	ARHGAP4	FALSE	FALSE
5.53E-26	0.489483788	0.265	0.069	1.39E-21	NPPC-4	ARL4A	FALSE	FALSE
6.13E-26	0.609274518	0.448	0.19	1.55E-21	NPPC-4	DRAP1	TRUE	FALSE
1.58E-25	0.580243843	0.584	0.305	4.00E-21	NPPC-4	SFPQ	TRUE	FALSE
1.58E-24	0.426571632	0.215	0.048	3.99E-20	NPPC-4	MAP3K1	FALSE	FALSE
3.06E-24	0.665813502	0.28	0.08	7.72E-20	NPPC-4	PI16	FALSE	TRUE
3.19E-24	0.693373239	0.631	0.397	8.06E-20	NPPC-4	DLC1	FALSE	FALSE
5.04E-24	0.563633936	0.763	0.625	1.27E-19	NPPC-4	ATP5F1E	FALSE	FALSE
1.32E-23	0.569767102	0.566	0.308	3.33E-19	NPPC-4	BZW1	FALSE	FALSE
1.46E-23	0.678749543	0.584	0.343	3.67E-19	NPPC-4	ARPC5	FALSE	FALSE
2.40E-23	0.81021389	0.52	0.266	6.05E-19	NPPC-4	C11orf96	FALSE	FALSE
1.92E-22	0.463282796	0.226	0.059	4.84E-18	NPPC-4	GLCE	FALSE	FALSE
2.43E-22	0.557032949	0.634	0.381	6.13E-18	NPPC-4	SRSF3	FALSE	FALSE
2.58E-22	0.667676218	0.527	0.28	6.50E-18	NPPC-4	RHOC	FALSE	FALSE
2.91E-22	0.417879765	0.222	0.056	7.34E-18	NPPC-4	SMTN	FALSE	FALSE
3.07E-22	0.548776996	0.677	0.431	7.76E-18	NPPC-4	SET	FALSE	FALSE
3.41E-22	0.506666558	0.312	0.105	8.60E-18	NPPC-4	MYO6	FALSE	FALSE
5.83E-22	0.446913405	0.24	0.067	1.47E-17	NPPC-4	STIM2	FALSE	FALSE
9.32E-22	0.402187917	0.229	0.061	2.35E-17	NPPC-4	ARPC5L	FALSE	FALSE
1.15E-21	0.388411275	0.28	0.086	2.91E-17	NPPC-4	PRKG1	FALSE	FALSE
1.25E-21	0.566695371	0.48	0.229	3.17E-17	NPPC-4	MEOX2	TRUE	FALSE
1.46E-21	0.58590766	0.552	0.303	3.69E-17	NPPC-4	WWTR1	FALSE	FALSE
1.59E-21	0.617216972	0.545	0.307	4.00E-17	NPPC-4	TPM3	FALSE	FALSE
1.61E-21	0.458917939	0.301	0.103	4.07E-17	NPPC-4	CYCS	TRUE	FALSE
1.79E-21	0.562031376	0.72	0.511	4.51E-17	NPPC-4	ELOB	FALSE	FALSE
1.83E-21	0.479531275	0.849	0.744	4.61E-17	NPPC-4	CALM2	FALSE	FALSE
1.94E-21	0.539682773	0.595	0.347	4.89E-17	NPPC-4	PRKAR1A	FALSE	FALSE
3.06E-21	0.458907725	0.437	0.187	7.72E-17	NPPC-4	HMGN1	FALSE	FALSE
3.24E-21	0.504941768	0.86	0.762	8.17E-17	NPPC-4	HSP90AB1	FALSE	FALSE
4.44E-21	0.453640984	0.907	0.804	1.12E-16	NPPC-4	HMGB1	TRUE	FALSE
8.35E-21	0.52225962	0.674	0.44	2.11E-16	NPPC-4	NFE2L2	TRUE	FALSE
2.28E-20	0.556628379	0.548	0.321	5.75E-16	NPPC-4	RBM3	TRUE	FALSE
3.41E-20	0.560584147	0.563	0.334	8.60E-16	NPPC-4	TMEM167	FALSE	FALSE
3.49E-20	0.642206772	0.398	0.18	8.80E-16	NPPC-4	S100B	FALSE	FALSE
3.95E-20	0.467316451	0.269	0.086	9.96E-16	NPPC-4	NR4A2	TRUE	FALSE
8.49E-20	0.508594963	0.799	0.628	2.14E-15	NPPC-4	EEF1B2	FALSE	FALSE
1.39E-19	0.57134712	0.226	0.067	3.50E-15	NPPC-4	TLE2	FALSE	FALSE
1.39E-19	0.593744184	0.667	0.493	3.52E-15	NPPC-4	CAVIN1	FALSE	FALSE
2.23E-19	0.455738066	0.276	0.096	5.63E-15	NPPC-4	TUBB6	FALSE	FALSE
2.31E-19	0.434188198	0.208	0.057	5.83E-15	NPPC-4	ZDHHC9	FALSE	FALSE
2.83E-19	0.441651545	0.208	0.057	7.14E-15	NPPC-4	NCK2	FALSE	FALSE
3.98E-19	0.541581452	0.523	0.29	1.00E-14	NPPC-4	FKBP2	FALSE	FALSE
5.24E-19	0.448206462	0.401	0.18	1.32E-14	NPPC-4	PFDN2	FALSE	FALSE
5.96E-19	0.44739197	0.247	0.079	1.50E-14	NPPC-4	OLFML2A	FALSE	FALSE
6.61E-19	0.393308429	0.265	0.088	1.67E-14	NPPC-4	ARHGEF4(	FALSE	FALSE
1.12E-18	0.356749587	0.237	0.072	2.84E-14	NPPC-4	ACLY	FALSE	FALSE
1.33E-18	0.536464248	0.649	0.445	3.35E-14	NPPC-4	PPDPF	FALSE	FALSE
1.53E-18	0.551968569	0.756	0.585	3.86E-14	NPPC-4	C2orf40	FALSE	FALSE
1.73E-18	0.46773503	0.319	0.124	4.37E-14	NPPC-4	SRPX2	FALSE	FALSE
4.00E-18	0.592841379	0.459	0.234	1.01E-13	NPPC-4	BNC2	TRUE	FALSE
4.57E-18	0.409663941	0.247	0.08	1.15E-13	NPPC-4	CSPG4	FALSE	TRUE
6.25E-18	0.357940087	0.208	0.06	1.58E-13	NPPC-4	NQO1	FALSE	FALSE
6.45E-18	0.377544179	0.301	0.113	1.63E-13	NPPC-4	SPTSSA	FALSE	FALSE
6.97E-18	0.53692114	0.599	0.392	1.76E-13	NPPC-4	CAVIN3	FALSE	FALSE
7.78E-18	0.514328159	0.509	0.28	1.96E-13	NPPC-4	LAMA4	FALSE	FALSE
1.05E-17	0.546337975	0.527	0.316	2.65E-13	NPPC-4	PDIA6	FALSE	FALSE
1.12E-17	0.513947719	0.516	0.302	2.82E-13	NPPC-4	DNAJA1	FALSE	FALSE
1.15E-17	0.526764461	0.459	0.24	2.90E-13	NPPC-4	CSRP1	FALSE	FALSE
1.56E-17	0.673740098	0.53	0.321	3.94E-13	NPPC-4	HSPA5	TRUE	FALSE
2.01E-17	0.384227345	0.28	0.101	5.07E-13	NPPC-4	C16orf45	FALSE	FALSE
3.50E-17	0.499173438	0.595	0.384	8.83E-13	NPPC-4	TPM2	FALSE	FALSE
3.57E-17	0.420447372	0.294	0.11	9.01E-13	NPPC-4	HSPH1	FALSE	FALSE
4.27E-17	0.43412596	0.348	0.15	1.08E-12	NPPC-4	SNRPD1	FALSE	FALSE

4.50E-17	0.449361488	0.703	0.512	1.14E-12	NPPC-4	RHOA	FALSE	FALSE
7.68E-17	0.409350593	0.832	0.739	1.94E-12	NPPC-4	HNRNPA2	FALSE	FALSE
9.00E-17	0.513692084	0.602	0.389	2.27E-12	NPPC-4	AES	FALSE	FALSE
9.81E-17	0.483890108	0.523	0.301	2.48E-12	NPPC-4	MAML2	FALSE	FALSE
1.03E-16	0.336967678	0.914	0.812	2.61E-12	NPPC-4	NUCKS1	FALSE	FALSE
1.34E-16	0.386327858	0.258	0.092	3.38E-12	NPPC-4	ASAP1	FALSE	FALSE
1.34E-16	0.488036635	0.53	0.307	3.39E-12	NPPC-4	PRNP	TRUE	TRUE
1.35E-16	0.499003235	0.344	0.154	3.40E-12	NPPC-4	ROBO1	FALSE	TRUE
1.67E-16	0.473074019	0.391	0.185	4.22E-12	NPPC-4	AMOTL1	FALSE	FALSE
1.83E-16	0.343244808	0.229	0.075	4.63E-12	NPPC-4	PHC2	FALSE	FALSE
3.78E-16	0.467040357	0.409	0.202	9.53E-12	NPPC-4	PDS5B	FALSE	FALSE
4.00E-16	0.433440979	0.308	0.127	1.01E-11	NPPC-4	KRAS	FALSE	FALSE
4.15E-16	0.380195351	0.226	0.075	1.05E-11	NPPC-4	COL21A1	FALSE	FALSE
6.78E-16	0.446338887	0.817	0.729	1.71E-11	NPPC-4	CALM1	FALSE	FALSE
6.86E-16	0.409162228	0.287	0.112	1.73E-11	NPPC-4	PSMD1	FALSE	FALSE
7.56E-16	0.483786372	0.692	0.545	1.91E-11	NPPC-4	HNRNPA3	FALSE	FALSE
9.21E-16	0.425209835	0.398	0.198	2.32E-11	NPPC-4	TMED9	FALSE	FALSE
1.06E-15	0.408060768	0.358	0.162	2.68E-11	NPPC-4	MTSS1	FALSE	FALSE
1.15E-15	0.539034204	0.355	0.163	2.90E-11	NPPC-4	DAAM1	FALSE	FALSE
1.58E-15	0.536952039	0.448	0.24	3.99E-11	NPPC-4	KIF5B	FALSE	FALSE
1.68E-15	0.372119555	0.867	0.755	4.25E-11	NPPC-4	GAPDH	FALSE	FALSE
3.26E-15	0.468969441	0.362	0.171	8.23E-11	NPPC-4	COL18A1	FALSE	FALSE
4.49E-15	0.514769728	0.462	0.258	1.13E-10	NPPC-4	HNRNPM	FALSE	FALSE
5.21E-15	0.376182286	0.297	0.125	1.31E-10	NPPC-4	YIF1A	FALSE	FALSE
5.57E-15	0.445900649	0.667	0.518	1.41E-10	NPPC-4	YWHAE	TRUE	FALSE
9.29E-15	0.292241961	0.265	0.101	2.34E-10	NPPC-4	GTF3C6	FALSE	FALSE
1.08E-14	0.443315185	0.728	0.601	2.74E-10	NPPC-4	YBX1	TRUE	FALSE
1.15E-14	0.424434657	0.495	0.301	2.89E-10	NPPC-4	SPATS2L	FALSE	FALSE
1.73E-14	0.439971379	0.763	0.647	4.36E-10	NPPC-4	MYL12B	FALSE	FALSE
1.81E-14	0.375199271	0.337	0.152	4.57E-10	NPPC-4	XBP1	TRUE	FALSE
2.16E-14	0.707802836	0.505	0.324	5.45E-10	NPPC-4	FGFBP2	FALSE	FALSE
2.72E-14	0.46654567	0.652	0.497	6.87E-10	NPPC-4	PFN1	FALSE	FALSE
3.76E-14	0.33079769	0.204	0.07	9.49E-10	NPPC-4	AKIRIN2	FALSE	FALSE
4.10E-14	0.347083473	0.308	0.134	1.03E-09	NPPC-4	NDUFS8	FALSE	FALSE
4.14E-14	0.351867988	0.81	0.665	1.04E-09	NPPC-4	SKP1	FALSE	FALSE
4.36E-14	0.430433257	0.362	0.169	1.10E-09	NPPC-4	VEGFA	FALSE	FALSE
4.69E-14	0.433625128	0.71	0.568	1.18E-09	NPPC-4	HNRNPDL	FALSE	FALSE
4.77E-14	0.372396023	0.229	0.084	1.20E-09	NPPC-4	RRP15	FALSE	FALSE
5.19E-14	0.484953774	0.251	0.101	1.31E-09	NPPC-4	RNASET2	FALSE	FALSE
6.28E-14	0.428205314	0.631	0.44	1.59E-09	NPPC-4	SEC61B	FALSE	FALSE
8.53E-14	0.395212495	0.595	0.388	2.15E-09	NPPC-4	PPFIBP1	FALSE	FALSE
1.01E-13	0.511711004	0.498	0.319	2.54E-09	NPPC-4	CALU	FALSE	FALSE
1.65E-13	0.381611288	0.455	0.258	4.16E-09	NPPC-4	EIF5A	FALSE	FALSE
2.25E-13	0.298062502	0.208	0.073	5.68E-09	NPPC-4	MAP9	FALSE	FALSE
2.51E-13	0.474389946	0.516	0.323	6.34E-09	NPPC-4	HSPD1	FALSE	FALSE
2.76E-13	0.390710086	0.434	0.24	6.96E-09	NPPC-4	RASSF8	FALSE	FALSE
3.39E-13	0.304948689	0.211	0.076	8.56E-09	NPPC-4	TRIB2	TRUE	FALSE
3.95E-13	0.460131924	0.398	0.215	9.96E-09	NPPC-4	TBCB	FALSE	FALSE
4.13E-13	0.458413729	0.47	0.275	1.04E-08	NPPC-4	MAP1B	FALSE	FALSE
4.17E-13	0.414109636	0.634	0.469	1.05E-08	NPPC-4	TUBB	FALSE	FALSE
6.06E-13	0.501195314	0.477	0.278	1.53E-08	NPPC-4	JAG1	FALSE	TRUE
6.76E-13	0.280249096	0.201	0.071	1.71E-08	NPPC-4	LPAR6	FALSE	TRUE
7.52E-13	0.598338569	0.29	0.132	1.90E-08	NPPC-4	IGFBP3	FALSE	FALSE
7.58E-13	0.288876842	0.968	0.945	1.91E-08	NPPC-4	RPS6	FALSE	FALSE
7.64E-13	0.352936201	0.294	0.132	1.93E-08	NPPC-4	CCT6A	FALSE	FALSE
7.82E-13	0.398520865	0.724	0.581	1.97E-08	NPPC-4	LMNA	FALSE	FALSE
7.90E-13	0.382629875	0.272	0.118	1.99E-08	NPPC-4	CCT5	FALSE	FALSE
9.55E-13	0.338010593	0.262	0.108	2.41E-08	NPPC-4	SRP19	FALSE	FALSE
9.63E-13	0.401822644	0.337	0.165	2.43E-08	NPPC-4	SPRED1	FALSE	FALSE
1.14E-12	0.422219661	0.588	0.405	2.89E-08	NPPC-4	PARK7	FALSE	FALSE
1.68E-12	0.394943532	0.538	0.334	4.23E-08	NPPC-4	HNRNPR	FALSE	FALSE
1.84E-12	0.445781972	0.419	0.239	4.65E-08	NPPC-4	SNRPG	FALSE	FALSE
2.07E-12	0.332414641	0.222	0.088	5.23E-08	NPPC-4	LARP4	TRUE	FALSE
2.10E-12	0.369043381	0.226	0.09	5.30E-08	NPPC-4	RHBDF1	FALSE	FALSE
2.21E-12	0.388829726	0.38	0.204	5.57E-08	NPPC-4	NDUFS6	FALSE	FALSE
2.81E-12	0.427796675	0.204	0.077	7.08E-08	NPPC-4	ARHGEF2	FALSE	FALSE
3.57E-12	0.360977897	0.204	0.076	9.00E-08	NPPC-4	GFRA1	FALSE	TRUE
3.85E-12	0.362836128	0.315	0.151	9.72E-08	NPPC-4	NAB1	FALSE	FALSE
7.86E-12	0.399618694	0.452	0.272	1.98E-07	NPPC-4	RAN	TRUE	FALSE
8.37E-12	0.377102317	0.416	0.243	2.11E-07	NPPC-4	PSMD8	FALSE	FALSE
1.16E-11	0.478745282	0.613	0.462	2.92E-07	NPPC-4	PDIA3	FALSE	FALSE
1.28E-11	0.408832819	0.355	0.189	3.23E-07	NPPC-4	PERP	FALSE	FALSE
1.35E-11	0.393481126	0.423	0.248	3.40E-07	NPPC-4	SNRPF	FALSE	FALSE
1.59E-11	0.338144429	0.789	0.674	4.02E-07	NPPC-4	H3F3A	FALSE	FALSE
1.78E-11	0.355033095	0.695	0.539	4.49E-07	NPPC-4	HMGN2	FALSE	FALSE
1.89E-11	0.389720384	0.43	0.245	4.77E-07	NPPC-4	IFI27L2	FALSE	FALSE
2.20E-11	0.400835941	0.419	0.247	5.56E-07	NPPC-4	ARF4	FALSE	FALSE
2.21E-11	0.312414851	0.204	0.078	5.57E-07	NPPC-4	LINC0119	FALSE	FALSE
2.72E-11	0.40857988	0.581	0.397	6.86E-07	NPPC-4	CLIC4	FALSE	FALSE
2.79E-11	0.377653849	0.742	0.629	7.04E-07	NPPC-4	DYNLL1	FALSE	FALSE
3.47E-11	0.319542383	0.233	0.098	8.75E-07	NPPC-4	DCBLD2	FALSE	TRUE
3.70E-11	0.276224011	0.961	0.904	9.34E-07	NPPC-4	RPS3	FALSE	FALSE
3.76E-11	0.715395568	0.204	0.081	9.49E-07	NPPC-4	CCL2	FALSE	FALSE
4.68E-11	0.344108455	0.444	0.264	1.18E-06	NPPC-4	SYNCRIP	FALSE	FALSE
5.12E-11	0.375956755	0.627	0.474	1.29E-06	NPPC-4	ARF1	FALSE	FALSE
5.93E-11	0.341829726	0.294	0.144	1.50E-06	NPPC-4	C9orf16	FALSE	FALSE
6.36E-11	0.277097848	0.312	0.157	1.60E-06	NPPC-4	SLC25A5	FALSE	FALSE
9.92E-11	0.275346103	0.233	0.101	2.50E-06	NPPC-4	TARS	FALSE	FALSE
9.99E-11	0.37722314	0.652	0.516	2.52E-06	NPPC-4	NCL	FALSE	FALSE

1.06E-10	0.311010739	0.222	0.094	2.69E-06	NPPC-4	USP11	FALSE	FALSE
1.08E-10	0.354903844	0.541	0.37	2.74E-06	NPPC-4	ARPC2	FALSE	FALSE
1.31E-10	0.331020268	0.319	0.162	3.31E-06	NPPC-4	ACSL3	FALSE	FALSE
1.48E-10	0.397307133	0.685	0.559	3.73E-06	NPPC-4	ANXA5	FALSE	FALSE
1.50E-10	0.266980558	0.935	0.894	3.80E-06	NPPC-4	RPL3	FALSE	FALSE
1.74E-10	0.350314267	0.674	0.534	4.40E-06	NPPC-4	CNN3	FALSE	FALSE
2.09E-10	0.435733301	0.495	0.338	5.29E-06	NPPC-4	ATP5MC3	FALSE	FALSE
2.14E-10	0.330876727	0.269	0.127	5.41E-06	NPPC-4	HACD3	FALSE	FALSE
2.19E-10	0.360153223	0.409	0.248	5.52E-06	NPPC-4	PSMB3	FALSE	FALSE
2.55E-10	0.344213144	0.269	0.129	6.43E-06	NPPC-4	NCOR2	TRUE	FALSE
2.58E-10	0.376055706	0.247	0.114	6.51E-06	NPPC-4	RASA2	FALSE	FALSE
2.62E-10	0.36036176	0.254	0.12	6.61E-06	NPPC-4	SLC26A2	FALSE	TRUE
2.63E-10	0.337361418	0.785	0.694	6.63E-06	NPPC-4	7-Sep	FALSE	FALSE
2.68E-10	0.336493222	0.344	0.191	6.76E-06	NPPC-4	COX17	FALSE	FALSE
2.83E-10	0.371445133	0.348	0.186	7.14E-06	NPPC-4	CRYBG3	FALSE	FALSE
3.52E-10	0.391859163	0.706	0.629	8.87E-06	NPPC-4	RPS26	FALSE	FALSE
3.90E-10	0.319763987	0.215	0.093	9.83E-06	NPPC-4	SERTAD1	FALSE	FALSE
3.99E-10	0.352708975	0.459	0.289	1.01E-05	NPPC-4	SRSF2	FALSE	FALSE
4.02E-10	0.418488396	0.434	0.273	1.01E-05	NPPC-4	BLOC1S1	FALSE	FALSE
4.43E-10	0.402265228	0.502	0.349	1.12E-05	NPPC-4	KDELR2	FALSE	FALSE
5.24E-10	0.3495252	0.305	0.159	1.32E-05	NPPC-4	NARS	FALSE	FALSE
5.46E-10	0.278006912	0.251	0.117	1.38E-05	NPPC-4	HSD17B10	FALSE	FALSE
5.59E-10	0.339397732	0.28	0.141	1.41E-05	NPPC-4	GLRX5	FALSE	FALSE
5.81E-10	0.254480538	0.215	0.093	1.47E-05	NPPC-4	HSPA4	FALSE	FALSE
8.47E-10	0.29421515	0.272	0.134	2.14E-05	NPPC-4	UBE2N	FALSE	FALSE
9.22E-10	0.399213072	0.351	0.198	2.33E-05	NPPC-4	ECM1	FALSE	FALSE
1.04E-09	0.258969649	0.222	0.096	2.63E-05	NPPC-4	MAN2A1	FALSE	FALSE
1.05E-09	0.337512276	0.441	0.278	2.66E-05	NPPC-4	ROMO1	FALSE	FALSE
1.17E-09	0.284982592	0.283	0.142	2.96E-05	NPPC-4	SH3KBP1	FALSE	FALSE
1.29E-09	0.297229966	0.355	0.198	3.25E-05	NPPC-4	CHD4	FALSE	FALSE
1.43E-09	0.263095238	0.301	0.153	3.61E-05	NPPC-4	ZNF428	TRUE	FALSE
1.52E-09	0.42540208	0.387	0.235	3.84E-05	NPPC-4	EIF3A	FALSE	FALSE
1.85E-09	0.392774814	0.491	0.329	4.68E-05	NPPC-4	TAGLN2	TRUE	FALSE
2.17E-09	0.346746954	0.391	0.24	5.48E-05	NPPC-4	LSM7	FALSE	FALSE
2.19E-09	0.273515872	0.842	0.755	5.52E-05	NPPC-4	SON	TRUE	FALSE
2.37E-09	0.251730213	0.237	0.112	5.98E-05	NPPC-4	PBDC1	FALSE	FALSE
2.80E-09	0.358425118	0.423	0.26	7.08E-05	NPPC-4	SNRPE	FALSE	FALSE
2.83E-09	0.264995912	0.222	0.101	7.14E-05	NPPC-4	SLC39A7	FALSE	FALSE
2.94E-09	0.348994581	0.283	0.149	7.41E-05	NPPC-4	CAV2	FALSE	FALSE
2.99E-09	0.314959308	0.262	0.13	7.55E-05	NPPC-4	ADGRD1	FALSE	FALSE
3.23E-09	0.310658504	0.638	0.484	8.14E-05	NPPC-4	ADD3	FALSE	FALSE
3.46E-09	0.338979063	0.538	0.392	8.72E-05	NPPC-4	COX8A	FALSE	FALSE
4.00E-09	0.255254204	0.81	0.711	0.000100948	NPPC-4	UBB	TRUE	FALSE
4.92E-09	0.342329912	0.509	0.354	0.000124146	NPPC-4	SUB1	FALSE	FALSE
5.28E-09	0.360953844	0.333	0.189	0.000133289	NPPC-4	ATP2B1	FALSE	FALSE
6.31E-09	0.305975008	0.254	0.128	0.000159288	NPPC-4	TXNL4A	FALSE	FALSE
8.34E-09	0.355435802	0.416	0.265	0.000210508	NPPC-4	ZC3H13	FALSE	FALSE
8.48E-09	0.290876508	0.219	0.103	0.000213997	NPPC-4	EIF4E	FALSE	FALSE
8.78E-09	0.394830854	0.265	0.137	0.000221431	NPPC-4	GFPT1	FALSE	FALSE
1.01E-08	0.255185535	0.244	0.121	0.000254609	NPPC-4	CDK2AP1	TRUE	FALSE
1.16E-08	0.333392065	0.735	0.641	0.000292303	NPPC-4	MARCKS	FALSE	FALSE
1.61E-08	0.384934287	0.254	0.133	0.000406899	NPPC-4	PSME2	FALSE	FALSE
1.66E-08	0.33281092	0.606	0.475	0.000419915	NPPC-4	APP	FALSE	TRUE
1.69E-08	0.326158057	0.595	0.432	0.000427488	NPPC-4	LTBP1	FALSE	FALSE
2.27E-08	0.348216188	0.477	0.316	0.000571846	NPPC-4	POLR2L	FALSE	FALSE
2.48E-08	0.272831985	0.785	0.674	0.000625949	NPPC-4	SEC62	FALSE	FALSE
2.49E-08	0.333367154	0.509	0.356	0.000627813	NPPC-4	PCM1	FALSE	FALSE
2.50E-08	0.28439846	0.272	0.143	0.000630657	NPPC-4	C1QBP	FALSE	FALSE
2.59E-08	0.287160696	0.781	0.688	0.00065421	NPPC-4	GSTP1	FALSE	FALSE
2.86E-08	0.284367544	0.265	0.14	0.000720433	NPPC-4	CCT3	FALSE	FALSE
3.15E-08	0.277008278	0.699	0.595	0.00079447	NPPC-4	DAD1	FALSE	FALSE
3.17E-08	0.263742885	0.229	0.112	0.000799235	NPPC-4	ILF2	TRUE	FALSE
3.36E-08	0.331748145	0.523	0.383	0.000846663	NPPC-4	TMED10	FALSE	FALSE
3.46E-08	0.313687483	0.728	0.652	0.000872059	NPPC-4	PPIB	FALSE	FALSE
3.79E-08	0.391593458	0.391	0.252	0.000955117	NPPC-4	RBM8A	TRUE	FALSE
4.03E-08	0.331691257	0.466	0.315	0.001017276	NPPC-4	2-Sep	FALSE	FALSE
4.11E-08	0.25112866	0.867	0.81	0.001036742	NPPC-4	RPL27	FALSE	FALSE
5.46E-08	0.328480262	0.427	0.275	0.001377476	NPPC-4	PRKDC	FALSE	FALSE
5.49E-08	0.288859125	0.301	0.167	0.001385068	NPPC-4	SPATA6	FALSE	FALSE
5.73E-08	0.285047626	0.215	0.104	0.001445511	NPPC-4	SRFBP1	FALSE	FALSE
6.19E-08	0.335256778	0.344	0.211	0.001563166	NPPC-4	TPBG	FALSE	TRUE
6.47E-08	0.293030423	0.301	0.173	0.00163314	NPPC-4	PRELID1	FALSE	FALSE
6.90E-08	0.307969672	0.548	0.414	0.001741883	NPPC-4	PJA2	FALSE	FALSE
7.68E-08	0.325858907	0.412	0.273	0.001937886	NPPC-4	CBX5	FALSE	FALSE
7.98E-08	0.339633305	0.434	0.301	0.002012607	NPPC-4	ACTR2	FALSE	FALSE
8.55E-08	0.274820708	0.24	0.124	0.002156707	NPPC-4	ERCC6L2	FALSE	FALSE
8.75E-08	0.261472887	0.226	0.113	0.002208111	NPPC-4	GNL3	FALSE	FALSE
9.32E-08	0.306890424	0.491	0.342	0.002352191	NPPC-4	EIF4G2	FALSE	FALSE
1.06E-07	0.403879313	0.638	0.53	0.002669802	NPPC-4	CALR	FALSE	FALSE
1.19E-07	0.418524805	0.448	0.299	0.002993338	NPPC-4	CRISPLD2	FALSE	FALSE
1.33E-07	0.286700867	0.53	0.389	0.003349528	NPPC-4	FIS1	FALSE	FALSE
1.34E-07	0.322942506	0.323	0.189	0.003393826	NPPC-4	PPP1R14B	FALSE	FALSE
1.37E-07	0.268544762	0.38	0.235	0.003469159	NPPC-4	NDUFAB1	FALSE	FALSE
1.43E-07	0.288227708	0.434	0.292	0.003606208	NPPC-4	BANF1	FALSE	FALSE
1.51E-07	0.283344623	0.28	0.158	0.003814908	NPPC-4	PDIA4	FALSE	FALSE
1.59E-07	0.278740865	0.283	0.158	0.004021094	NPPC-4	WDR82	FALSE	FALSE
1.72E-07	0.29794249	0.301	0.175	0.004333429	NPPC-4	HSPA9	FALSE	FALSE
2.06E-07	0.274047707	0.287	0.165	0.005189903	NPPC-4	FYTTD1	FALSE	FALSE
2.20E-07	0.266551008	0.563	0.431	0.005551159	NPPC-4	HNRNPD	FALSE	FALSE

2.20E-07	0.287641758	0.323	0.19	0.005559533	NPPC-4	DDX21	FALSE	FALSE
2.57E-07	0.301350502	0.28	0.161	0.006490017	NPPC-4	CDC42EP5	FALSE	FALSE
3.38E-07	0.31419822	0.348	0.224	0.00853949	NPPC-4	ABI2	FALSE	FALSE
3.41E-07	0.288374539	0.487	0.349	0.008599035	NPPC-4	RAB14	TRUE	FALSE
3.45E-07	0.274658176	0.373	0.241	0.00871711	NPPC-4	RPN2	FALSE	FALSE
3.85E-07	0.27418246	0.394	0.259	0.009707684	NPPC-4	ZNF644	TRUE	FALSE
4.12E-07	0.257950976	0.251	0.137	0.01038582	NPPC-4	CKAP4	FALSE	FALSE
4.26E-07	0.278470434	0.301	0.176	0.010759513	NPPC-4	PHF20L1	FALSE	FALSE
4.68E-07	0.301800881	0.491	0.343	0.011816038	NPPC-4	ANP32B	FALSE	FALSE
4.90E-07	0.277433179	0.319	0.194	0.012355644	NPPC-4	USP47	FALSE	FALSE
4.96E-07	0.295613837	0.47	0.33	0.012514518	NPPC-4	TMED2	FALSE	FALSE
5.56E-07	0.262390556	0.312	0.19	0.014026805	NPPC-4	GLO1	FALSE	FALSE
6.13E-07	0.342584476	0.315	0.195	0.015457278	NPPC-4	ZFYVE16	FALSE	FALSE
6.77E-07	0.408821949	0.441	0.313	0.017073479	NPPC-4	BTG2	FALSE	FALSE
6.84E-07	0.319852939	0.71	0.617	0.017260772	NPPC-4	PRDX1	FALSE	FALSE
7.22E-07	0.304290465	0.344	0.217	0.018221632	NPPC-4	NHP2	FALSE	FALSE
7.52E-07	0.320640941	0.351	0.234	0.018977839	NPPC-4	IFNGR1	FALSE	TRUE
8.77E-07	0.260473482	0.821	0.737	0.02213396	NPPC-4	RPS10	TRUE	FALSE
9.73E-07	0.264755165	0.358	0.227	0.024555936	NPPC-4	PSMB6	FALSE	FALSE
1.05E-06	0.327342553	0.409	0.285	0.026525017	NPPC-4	ITPRIPL2	FALSE	FALSE
1.07E-06	0.296601019	0.638	0.518	0.02697121	NPPC-4	ST13	FALSE	FALSE
1.21E-06	0.256490368	0.305	0.188	0.030530405	NPPC-4	VDAC1	FALSE	FALSE
1.22E-06	0.291508339	0.376	0.258	0.030858217	NPPC-4	ATP5MC1	FALSE	FALSE
1.34E-06	0.261927063	0.405	0.269	0.033926616	NPPC-4	CLTC	FALSE	FALSE
1.39E-06	0.315475905	0.362	0.237	0.035199757	NPPC-4	CREB3L2	TRUE	FALSE
1.48E-06	0.340549398	0.477	0.354	0.037440297	NPPC-4	XRCC5	FALSE	FALSE
1.64E-06	0.253202669	0.713	0.633	0.041391293	NPPC-4	RAC1	FALSE	FALSE
1.64E-06	0.265734089	0.498	0.38	0.041450186	NPPC-4	ERH	FALSE	FALSE
1.82E-06	0.289780029	0.251	0.141	0.045833187	NPPC-4	SELENOT	FALSE	FALSE
1.82E-06	0.273257087	0.627	0.501	0.045908707	NPPC-4	ATP5MG	FALSE	FALSE
