# Supplementary material for: Spatially defined single-cell transcriptional profiling characterizes diverse chondrocyte subtypes and nucleus pulposus progenitors in human intervertebral discs
Source: Bone Res. 2021 Aug 16;9:37. doi: 10.1038/s41413-021-00163-z (PMC8368097; doi:10.1038/s41413-021-00163-z)
Supplement: Supplementary file 7 — Supplementary Table 6 [file 41413_2021_163_MOESM7_ESM.pdf]

Supplementary Table 6. Scaled activity values of 227 Regulon enriched in NPPC subclusters				
	NPPC-1	NPPC-2	NPPC-3	NPPC-4
HSF2 (28g)	-1.309150292	0.881901686	-0.243050769	0.670299376
PBX3_extended (124g)	-0.787923256	1.386117409	0.074357974	-0.672552127
KLF2_extended (65g)	-0.188584335	1.086112817	0.382504731	-1.280033214
KLF4_extended (17g)	-1.107129734	0.786762976	0.903133694	-0.582766936
MAFF_extended (15g)	-0.911372485	1.245486141	-0.697315491	0.363201834
NFIL3_extended (106g)	-1.190031553	1.135933611	-0.355061249	0.409159192
ATF3 (233g)	-1.137558223	0.994917907	-0.522786952	0.665427269
JUN (44g)	-1.053250037	1.202833641	-0.539908972	0.390325368
FOS (26g)	-0.82436681	1.417160298	-0.035270345	-0.557523143
FOSB (56g)	-1.071906271	1.344742009	-0.084138783	-0.188696954
JUND (26g)	-1.041306249	1.259615682	-0.499808095	0.281498662
JUNB (81g)	-0.89227891	1.425965378	-0.148415061	-0.385271407
PML_extended (54g)	-1.084437344	1.273357519	-0.398360449	0.209440273
ETS2 (64g)	-0.903783223	1.379781989	-0.526174793	0.050176027
ELF2_extended (85g)	-0.769785259	1.438850295	-0.572562424	-0.096502613
POLR2A (339g)	-1.317752376	1.076574831	-0.073617308	0.314794853
MEIS1 (21g)	-0.956312758	1.210648623	0.414797221	-0.669133086
RBPJ (10g)	-0.215931241	1.102303563	0.377018877	-1.263391199
HOXB4_extended (10g)	-0.464746761	1.073263896	0.546320207	-1.154837343
FOXC1 (21g)	0.289901892	1.044717803	0.016033675	-1.35065337
SIX1 (20g)	-0.491722552	1.072406763	0.558055087	-1.138739297
TFAP4 (25g)	-0.420000815	1.476296533	-0.735834571	-0.320461147
MAFB (68g)	0.052496681	0.939670501	0.404490001	-1.396657183
DEAF1 (83g)	-0.476666732	1.059076374	0.569476759	-1.151886401
BACH2_extended (44g)	0.124027125	0.526709467	0.791651255	-1.442387847
ZFP14 (25g)	0.101013173	0.367009682	0.937649261	-1.405672115
TCF12_extended (19g)	-1.360514563	0.58353642	-0.114818913	0.891797055
EP300 (18g)	-0.841213944	0.857124838	0.874532382	-0.890443275
TCF7L2_extended (22g)	-0.277100801	-0.082615593	1.373946106	-1.014229712
SMAD3_extended (13g)	-0.509672449	-0.233422532	1.469193344	-0.726098363
KLF7_extended (74g)	-0.504147192	0.102589214	1.352882859	-0.951324882
ELF1_extended (58g)	0.080405602	1.009696811	0.285576764	-1.375679178
ELK1 (24g)	-0.586740846	0.959243858	0.726493571	-1.098996583
GLIS2_extended (18g)	-0.172350509	0.501695082	0.989529478	-1.31887405
ZNF445 (19g)	-0.655282247	1.441942172	-0.091675332	-0.694984593
FOXK1_extended (41g)	-1.150224627	1.219836423	-0.340223242	0.270611445
NFIA_extended (11g)	-0.312990699	1.281236492	0.145051132	-1.113296925
HSF1 (30g)	-1.07176058	0.937234508	-0.626953107	0.761479178
CTCF (27g)	-0.944987189	1.258685709	-0.643422028	0.329723508
SIN3A (57g)	-0.362609954	0.037790488	-1.023830558	1.348650024
ETS1 (32g)	-0.997046734	1.140160998	0.518226668	-0.661340932
ELK4 (127g)	-1.219291917	0.512270314	-0.353937632	1.060959236
SP1 (243g)	-1.202870328	0.877543576	-0.441530921	0.766857673
MSX1 (161g)	-1.19531455	1.227376888	-0.195272772	0.163210434
SOX4 (83g)	-1.151237532	1.246435099	-0.288970282	0.193772715
RARA_extended (24g)	-0.73075179	1.473714191	-0.27616137	-0.466801031
FOXJ2_extended (91g)	-0.968908408	1.1207597	-0.705840237	0.553988945
TCF3_extended (61g)	-0.913033825	0.5692309	-0.772356233	1.116159158
MEOX2_extended (13g)	-0.894833561	0.563017466	-0.789924906	1.121741001
BHLHE40 (13g)	-1.32602348	1.098691306	0.047714643	0.179617532
DLX5_extended (20g)	-0.341367755	1.463214039	-0.323012245	-0.798834039
MAZ_extended (17g)	-0.56641441	1.494238108	-0.374472523	-0.553351175
FOXN3 (107g)	0.49344599	-0.003973004	0.903458475	-1.392931461
FOXO3 (159g)	0.609598135	0.278818598	0.594061386	-1.482478119

FOXP1 (364g)	0.41640392	0.421479694	0.652957947	-1.490841562
KAT2A (13g)	1.180833463	-0.817685742	0.478382284	-0.841530005
ZFP64_extended (17g)	1.020988199	-0.454080954	0.608370819	-1.175278065
SP2 (25g)	-0.337256185	-0.416039404	-0.725496106	1.478791696
HOXA6_extended (103g)	1.455151102	-0.68205535	-0.143137474	-0.629958278
HOXA5_extended (13g)	1.474266765	-0.546028308	-0.243237727	-0.68500073
HOXD9 (22g)	0.926533804	-0.317093733	0.65842016	-1.267860232
ZNF568 (28g)	1.274296711	-0.422590413	0.220244145	-1.071950443
GSC (18g)	0.926785339	-0.008320857	0.467916937	-1.386381419
JDP2_extended (100g)	0.815393536	-1.135513366	0.865221039	-0.54510121
ERG (43g)	1.322438532	-0.747443597	0.225910173	-0.800905107
HOXC10_extended (18g)	1.064779933	-0.043496424	0.308761001	-1.33004451
CREB5 (45g)	0.802381276	-0.67731066	0.909476324	-1.034546941
FOXP4_extended (108g)	1.290634192	-0.439366298	0.19842219	-1.049690084
MXI1_extended (207g)	1.294725018	-0.392143684	0.166197765	-1.068779099
NFATC4_extended (43g)	1.092809492	-0.367111259	0.476101948	-1.20180018
MAF (11g)	1.297298234	-0.598165277	0.248272191	-0.947405147
HOXA7_extended (157g)	1.193031965	-0.56508523	0.414104534	-1.042051269
HOXA10 (77g)	1.23381936	-0.676950354	0.378975742	-0.935844748
PAX9_extended (62g)	0.947676651	-0.501800503	0.712663992	-1.158540139
MBNL2 (18g)	1.071821775	-0.485745499	0.55652254	-1.142598816
FOXO1 (77g)	1.175206966	-0.639240969	0.462301123	-0.99826712
CEBPD (130g)	1.060368361	-0.30761611	0.488977356	-1.241729606
RARG (12g)	1.100564457	-0.231942719	0.389615277	-1.258237015
TGIF1 (29g)	1.078100831	-0.638444347	0.596700539	-1.036357023
MZF1 (19g)	1.238055717	-0.565959227	0.342572175	-1.014668665
TWIST1_extended (11g)	1.432147061	-0.243317685	-0.291847622	-0.896981753
KLF3_extended (45g)	0.911137679	0.340783103	0.170921716	-1.422842498
STAT3_extended (16g)	0.829180136	-1.356343636	-0.144977649	0.672141149
SIX5 (45g)	1.364495219	-1.022887229	-0.04136964	-0.300238351
CREB3L1_extended (28g)	1.246025299	-0.811508987	0.372009146	-0.806525458
STAT2_extended (18g)	0.810200945	-0.005841301	0.602818459	-1.407178103
ZNF358_extended (11g)	1.053025245	0.115581212	0.188664523	-1.35727098
ZBTB17_extended (117g)	-0.776449551	0.0583156	-0.674341753	1.392475704
SOX9 (98g)	-0.766181528	-0.063094889	-0.601515129	1.430791547
ZEB1_extended (265g)	-0.891383661	0.351940496	-0.714167027	1.253610192
SREBF1 (11g)	0.947592561	0.702463876	-1.176645708	-0.47341073
VEZF1_extended (224g)	-0.766464144	0.143458817	-0.736801793	1.359807119
KLF5 (13g)	-0.50576979	-0.356389392	-0.628525121	1.490684303
BCLAF1 (126g)	-0.662938344	0.178547581	-0.855829279	1.340220041
SP3_extended (335g)	-0.9453284	0.424279387	-0.685677159	1.206726172
SMARCA4_extended (324g)	-0.88143396	0.119809037	-0.598647519	1.360272442
ETV1_extended (307g)	-0.83464659	0.059837315	-0.612500323	1.387309598
ETV6_extended (81g)	-0.895828207	0.565773899	-0.789677982	1.119732291
CHD1 (79g)	-0.961114746	0.450816158	-0.678344389	1.188642977
EGR2 (39g)	-0.466627313	-0.389639148	-0.635785036	1.492051498
HCFC1_extended (190g)	-0.935205982	0.394351671	-0.684458921	1.225313232
GMEB1 (202g)	-0.899460274	0.562935101	-0.785008281	1.121533454
ERF (80g)	-0.969172288	1.234892178	-0.633081323	0.367361433
KLF13_extended (297g)	-1.261932392	0.676749585	-0.331234365	0.916417172
ELK3 (15g)	-0.066682011	0.452756437	-1.358378019	0.972303593
EGR1 (81g)	-1.05334624	0.822342332	-0.655060628	0.886064535
NR2F2_extended (161g)	-1.103641041	0.729090157	-0.580923691	0.955474575
ZNF143 (124g)	-1.039568474	0.038324941	-0.340979172	1.342222705
HDAC2 (11g)	-0.818497759	-0.439091948	-0.191721369	1.449311076
SREBF2 (58g)	-0.895666829	0.089089994	-0.562258269	1.368835104
XBP1 (142g)	-0.67463067	-0.184027763	-0.605649065	1.464307499
TFDP1_extended (13g)	-0.812532259	0.092403577	-0.657766747	1.377895429

E2F6 (76g)	-0.702679455	0.139016437	-0.797070251	1.360733269
GTF2B (28g)	-0.721766015	0.663834419	-0.980167407	1.038099003
NFYB_extended (53g)	-0.933209017	0.316802298	-0.650644546	1.267051265
NFE2L2_extended (39g)	-0.764069911	0.14163256	-0.738167636	1.360604987
YY1 (654g)	-0.938561941	0.177673245	-0.567727022	1.328615719
MYC (13g)	-0.603185366	1.140121291	-1.040523907	0.503587981
TAF7 (13g)	-0.798259834	0.089475964	-0.671178151	1.379962021
CEBPB_extended (605g)	-0.941486468	0.199258104	-0.577160031	1.319388395
RAD21 (16g)	-0.992914547	0.435375184	-0.634779464	1.192318827
ATF4_extended (120g)	-0.43779814	-0.198264442	-0.814827084	1.450889666
UQCRB (194g)	-0.948644595	-0.134793105	-0.322857817	1.406295517
GTF2F1 (385g)	-0.874151826	0.134300786	-0.616008581	1.355859621
TAF1 (160g)	-1.030367799	0.306398026	-0.527508321	1.251478094
XRCC4_extended (201g)	-0.724338938	0.11518829	-0.76232029	1.371470939
THAP1 (59g)	-0.45155022	-0.284297496	-0.737639664	1.47348738
RBBP5 (14g)	-0.240889118	0.178091835	-1.174479458	1.237276741
IRF7 (24g)	-0.903019343	0.177873792	-0.609538058	1.334683609
RELA_extended (56g)	-1.155725537	0.538455628	-0.460732627	1.078002536
PATZ1_extended (18g)	-1.20439444	0.97764071	-0.419513391	0.64626712
ARNT (27g)	-0.909675062	1.12148181	-0.773862498	0.562055749
CNOT3_extended (32g)	-0.593205013	0.447522837	-1.031065492	1.176747668
METTL14 (14g)	-0.599839771	0.851084006	-1.096262739	0.845018504
PBX1 (17g)	-0.511298154	1.427944025	-0.084538082	-0.832107788
MAFG (12g)	-0.256581716	1.371073837	-0.09180218	-1.022689941
ZNF189 (11g)	-1.01689665	1.280336477	-0.513792837	0.25035301
TRIM28 (20g)	-0.461480528	1.15085905	-1.127293183	0.437914661
CREM_extended (12g)	0.431754456	-0.338993581	1.114302368	-1.207063243
POLR3A (24g)	-0.996217332	0.011911852	-0.380481347	1.364786826
IRF9 (32g)	-0.654927413	1.418526627	-0.747388455	-0.016210759
NR1D2 (49g)	-0.68959818	0.338953539	-0.908211024	1.258855665
IRF2 (70g)	-0.772729367	1.142809396	-0.90211616	0.532036131
TFCP2 (13g)	-0.441372619	1.483678984	-0.695345038	-0.346961326
PPARD (14g)	-0.045021456	0.26148661	-1.313672173	1.097207019
RFXAP (30g)	-0.642297397	0.380295125	-0.966383728	1.228385999
RFX1 (11g)	-0.223257036	0.195987867	1.220152974	-1.192883805
RXRA_extended (30g)	-1.488300214	0.399583148	0.416602208	0.672114858
CENPB (12g)	-0.422246914	1.378620074	-0.959741056	0.003367896
TAF9 (11g)	-0.746400012	1.333157137	0.203329153	-0.790086278
ZNF140 (10g)	-1.062607824	0.517072303	-0.580079338	1.125614858
NFATC1_extended (19g)	1.380059404	-0.419516136	-0.958874062	-0.001669206
NFYC_extended (22g)	-0.110239397	0.970189892	-1.345622948	0.485672453
ZNF414 (14g)	-1.148591361	0.424685208	-0.424926933	1.148833087
MEF2C_extended (21g)	0.209290417	1.187302629	-0.163846376	-1.23274667
ZNF250_extended (11g)	-0.368348401	1.123108125	-1.189203881	0.434444157
SETDB1 (55g)	-0.3030285	-0.419086397	-0.750656399	1.472771296
ZNF37A (22g)	-1.129778733	0.784301052	0.896891608	-0.551413926
CEBPG_extended (12g)	-0.495593915	-0.091246132	-0.841011595	1.427851642
TBX15_extended (21g)	-1.350233384	0.956716302	-0.10666845	0.500185532
SMC3_extended (49g)	-0.509163119	0.066507867	-0.927227507	1.369882759
CEBPZ_extended (12g)	-0.503539518	0.534146964	-1.124507619	1.093900173
RB1_extended (25g)	0.635073829	-1.077088992	-0.597183054	1.039198218
UBTF_extended (23g)	-0.838022496	0.010837011	-0.575215364	1.402400849
MTA3_extended (12g)	-0.143793269	-0.133606439	1.347624483	-1.070224775
ZNF664 (11g)	-0.869191866	0.613103563	-0.830031762	1.086120065
BRF2_extended (22g)	1.386907115	-0.2855869	-0.109935892	-0.991384323
HOXC9_extended (20g)	-1.031753823	1.267526604	-0.505872185	0.270099404
SAP30_extended (20g)	-1.284731855	0.975369413	-0.263807734	0.573170176
PSMD12_extended (13g)	-0.580620983	-0.279311544	-0.622441063	1.48237359

NR2C2 (64g)	-0.572759399	0.081788266	-0.882506239	1.373477371
ATF6 (21g)	0.347245978	0.403307179	-1.478702727	0.72814957
RFX2_extended (26g)	-0.501109351	-0.240178423	1.469836764	-0.72854899
ZNF148_extended (23g)	-0.742495724	1.474839048	-0.314275087	-0.418068237
TFE3_extended (14g)	1.493667734	-0.627637929	-0.429585803	-0.436444002
POLI (69g)	-0.048121348	0.2397771	1.112854322	-1.304510075
ZNF629 (25g)	-1.058815145	1.322722217	0.085409011	-0.349316083
CHD2 (62g)	-0.645684049	0.738361194	-1.054708626	0.962031481
SMAD1_extended (38g)	0.005704483	-0.913442502	-0.482505942	1.390243961
ZNF91_extended (116g)	0.889487881	0.747573005	-0.424854345	-1.212206541
FOXP2_extended (88g)	0.49729851	-1.08387592	-0.545123679	1.131701089
SMAD5 (62g)	-0.768396389	0.221464804	-0.777950692	1.324882278
RCOR1_extended (32g)	-0.814068883	0.778055359	-0.912389182	0.948402706
USF2_extended (86g)	0.229865965	0.599050833	-1.473879988	0.64496319
TBX18_extended (81g)	-0.93070099	0.379489	-0.68300405	1.23421604
ZFX_extended (72g)	-0.376299602	0.041320144	-1.01584996	1.350829417
NCALD (17g)	-0.864940238	0.892169584	-0.866709363	0.839480017
TBP (10g)	-0.212610952	-0.50591452	-0.745433524	1.463958996
TP53 (13g)	-0.170622954	-0.05030525	-1.102786192	1.323714397
FLI1 (52g)	-0.974539118	1.087530758	-0.712684361	0.599692721
NFIC_extended (18g)	-0.415016221	-0.652585487	1.490881575	-0.423279867
NR3C1 (11g)	-0.615597367	-0.048553148	-0.76294404	1.427094554
CREB3L2 (78g)	-0.628839965	-0.770838084	-0.018081453	1.417759503
ATF6B (55g)	-0.86015867	0.391557294	-0.764953114	1.233554491
HOXB2 (49g)	-1.107452352	0.205661229	-0.362934621	1.264725744
IRF3_extended (18g)	1.332543819	-1.089291749	-0.057654671	-0.185597399
NR2F1 (18g)	-0.443909922	-0.431393615	-0.619174941	1.494478478
CREB3 (43g)	-0.732993451	-0.114507013	-0.59861962	1.446120084
REST_extended (100g)	-0.798960989	0.310141324	-0.79144763	1.280267295
ETV5 (10g)	-0.956301476	0.771256156	-0.76582766	0.95087298
SFPQ (35g)	-0.661396614	-0.099818667	-0.683080848	1.444296129
CREB1 (191g)	-0.589847412	0.072793745	-0.862434552	1.379488219
GABPA_extended (192g)	-0.825517651	0.909282529	-0.904499855	0.820734977
CLOCK_extended (41g)	-0.768081101	-0.116004425	-0.559403888	1.443489413
KDM5A_extended (453g)	-1.047142154	0.378410537	-0.542189319	1.210920937
RFX3 (17g)	-0.308521846	0.08404212	-1.086208221	1.310687947
HINFP (46g)	-0.866090614	0.384525037	-0.755729841	1.237295418
STAT1 (20g)	-0.40549732	-0.126749221	-0.890998885	1.423245427
ZFP82 (22g)	0.100792062	-0.342791131	-1.071278416	1.313277486
GLI1_extended (10g)	-0.550638485	-0.290785121	-0.641889823	1.483313429
REL (88g)	0.513267789	-0.325147932	-1.237112959	1.048993101
FOXJ3_extended (26g)	-0.277003529	1.35661201	-1.03985207	-0.039756411
HMGA1 (10g)	1.184087622	0.357043824	-0.394458596	-1.146672851
MAX (78g)	-0.257370016	0.787569275	-1.307438271	0.777239012
KLF6 (15g)	-0.606604188	0.338390076	-0.97979467	1.248008782
ZNF502 (31g)	-0.323013091	-0.253294409	1.442588529	-0.866281029
GABPB1 (41g)	-1.102294308	0.112018347	-0.305510479	1.295786439
BACH1_extended (52g)	-1.248662813	0.785346938	-0.366982424	0.8302983
E2F4_extended (11g)	-1.4212217	0.73361278	0.023228295	0.664380625
NFKB1 (49g)	-1.134599378	0.959509139	0.710721727	-0.535631488
CUX1_extended (139g)	-0.998218033	0.581555667	-0.680080321	1.096742687
BRF1_extended (114g)	-0.255967749	0.025123249	-1.090232083	1.321076583
MGA_extended (22g)	-1.1333325	1.304169033	-0.078069194	-0.092767339
NFKB2 (44g)	-0.728696558	0.622100143	-0.965601037	1.072197452
DDIT3 (10g)	1.415932375	-0.005188191	-0.707495416	-0.703248768
ATF1_extended (87g)	-0.667202675	0.836799855	-1.044023535	0.874426355
IRF1_extended (26g)	-0.570288822	1.437133689	-0.775261145	-0.091583723
