# Supplementary material for: Spatially defined single-cell transcriptional profiling characterizes diverse chondrocyte subtypes and nucleus pulposus progenitors in human intervertebral discs
Source: Bone Res. 2021 Aug 16;9:37. doi: 10.1038/s41413-021-00163-z (PMC8368097; doi:10.1038/s41413-021-00163-z)
Supplement: Supplementary file 8 — Supplementary Table 7 [file 41413_2021_163_MOESM8_ESM.pdf]

Supplementary Table 7.
DEGs among the 6 stromal cell subclusters

p_val	avg_logFC	pct.1	pct.2	p_val_adj	cluster	gene	is.TF	is.surface
4.15E-137	2.293926874	0.691	0.049	1.05E-132	Fib1	CEMIP	FALSE	FALSE
6.24E-133	1.239546689	0.614	0.027	1.57E-128	Fib1	SPON1	FALSE	FALSE
5.83E-128	2.035684002	0.928	0.188	1.47E-123	Fib1	MMP2	FALSE	FALSE
6.95E-121	1.369138239	0.89	0.151	1.75E-116	Fib1	MEDAG	FALSE	FALSE
8.71E-93	0.89075896	0.754	0.14	2.20E-88	Fib1	SNED1	FALSE	FALSE
1.84E-87	1.315400996	0.733	0.146	4.64E-83	Fib1	COL15A1	FALSE	FALSE
2.58E-83	1.252554803	0.619	0.102	6.52E-79	Fib1	HTRA3	FALSE	FALSE
1.05E-82	0.813633191	0.559	0.064	2.64E-78	Fib1	TSHZ2	TRUE	FALSE
3.40E-82	1.571537377	0.581	0.082	8.58E-78	Fib1	ADAMTS5	FALSE	FALSE
1.32E-80	1.194084608	0.932	0.351	3.33E-76	Fib1	MMP14	FALSE	TRUE
7.52E-79	0.761225937	0.559	0.073	1.90E-74	Fib1	A4GALT	FALSE	FALSE
6.91E-77	1.240939488	0.852	0.265	1.74E-72	Fib1	GJA1	FALSE	TRUE
1.02E-74	1.174399147	0.733	0.173	2.56E-70	Fib1	AKR1C1	FALSE	FALSE
1.85E-73	1.283731551	0.839	0.291	4.67E-69	Fib1	ANKH	FALSE	TRUE
8.17E-73	1.164082428	0.962	0.461	2.06E-68	Fib1	LRP1	FALSE	TRUE
3.68E-72	2.462005516	0.754	0.208	9.28E-68	Fib1	PRG4	FALSE	FALSE
6.81E-68	0.953046087	0.568	0.101	1.72E-63	Fib1	MGST1	FALSE	FALSE
8.87E-68	1.291690607	0.915	0.399	2.24E-63	Fib1	CTSL	FALSE	FALSE
1.56E-67	1.222946465	0.784	0.252	3.95E-63	Fib1	UAP1	FALSE	FALSE
2.86E-66	1.043792579	0.877	0.327	7.23E-62	Fib1	PRRX1	TRUE	FALSE
1.82E-65	0.689557857	0.606	0.121	4.59E-61	Fib1	MAN1A1	FALSE	FALSE
2.54E-65	0.92353296	0.581	0.102	6.40E-61	Fib1	TNXB	FALSE	FALSE
1.57E-64	0.690519829	0.419	0.043	3.97E-60	Fib1	GALNT15	FALSE	FALSE
5.91E-64	1.052214799	0.847	0.306	1.49E-59	Fib1	MXRA8	FALSE	TRUE
1.03E-59	0.97739256	0.542	0.114	2.59E-55	Fib1	PTGES	FALSE	FALSE
2.50E-59	0.850414998	0.699	0.21	6.30E-55	Fib1	PRRX2	TRUE	FALSE
5.06E-58	1.306637425	0.411	0.051	1.28E-53	Fib1	IGF1	FALSE	FALSE
1.00E-55	1.241811165	0.869	0.402	2.53E-51	Fib1	IGFBP4	FALSE	FALSE
2.49E-55	0.644351021	0.47	0.081	6.28E-51	Fib1	STEAP1	FALSE	FALSE
5.46E-55	0.539546801	0.343	0.033	1.38E-50	Fib1	CMKLR1	FALSE	TRUE
6.64E-54	0.975394587	0.72	0.268	1.67E-49	Fib1	ENG	FALSE	TRUE
1.37E-53	0.629291318	0.576	0.142	3.45E-49	Fib1	BICC1	FALSE	FALSE
2.11E-52	0.650243318	0.547	0.124	5.33E-48	Fib1	KDM7A	TRUE	FALSE
2.77E-52	0.704046131	0.771	0.27	6.99E-48	Fib1	LTBP3	FALSE	FALSE
1.22E-51	0.984834458	0.75	0.257	3.07E-47	Fib1	CD55	FALSE	TRUE
1.95E-51	0.817354535	0.483	0.094	4.93E-47	Fib1	MSX1	TRUE	FALSE
1.56E-50	0.428516098	0.424	0.062	3.94E-46	Fib1	ABCA6	FALSE	TRUE
2.05E-50	0.81245553	0.75	0.278	5.17E-46	Fib1	SMIM14	FALSE	FALSE
5.79E-50	0.810010547	0.555	0.144	1.46E-45	Fib1	SLC16A7	FALSE	TRUE
9.42E-50	0.998654209	0.894	0.428	2.38E-45	Fib1	SERPINE2	FALSE	FALSE
9.67E-50	0.723412852	0.623	0.18	2.44E-45	Fib1	PROCR	FALSE	TRUE
3.14E-48	1.096751865	0.614	0.174	7.93E-44	Fib1	CRLF1	FALSE	FALSE
2.11E-47	0.820539004	0.983	0.735	5.33E-43	Fib1	ITM2B	FALSE	TRUE
1.12E-46	0.707366598	0.631	0.196	2.83E-42	Fib1	NBL1	FALSE	FALSE
2.16E-46	0.726908066	0.831	0.35	5.45E-42	Fib1	C1R	FALSE	FALSE
8.37E-46	0.936555659	0.568	0.148	2.11E-41	Fib1	ADAMTS4	FALSE	FALSE
1.55E-45	0.776580897	0.733	0.279	3.91E-41	Fib1	FOXC1	TRUE	FALSE
3.27E-45	0.506144752	0.314	0.036	8.24E-41	Fib1	CHST2	FALSE	FALSE
5.92E-45	0.821804461	0.64	0.224	1.49E-40	Fib1	CLMP	FALSE	TRUE
1.11E-44	0.703254176	0.682	0.237	2.80E-40	Fib1	FAP	FALSE	TRUE
4.52E-43	0.443769004	0.403	0.07	1.14E-38	Fib1	NTN4	FALSE	FALSE
5.79E-43	0.756797301	0.78	0.3	1.46E-38	Fib1	SLC39A14	FALSE	TRUE
6.33E-43	0.83899401	0.708	0.262	1.60E-38	Fib1	UGDH	FALSE	FALSE
1.03E-42	0.659648528	0.788	0.308	2.61E-38	Fib1	TRPS1	TRUE	FALSE
1.06E-42	0.795236542	0.657	0.245	2.67E-38	Fib1	RAB31	FALSE	FALSE
2.16E-42	0.694812465	0.627	0.218	5.45E-38	Fib1	MIR100HG	FALSE	FALSE
1.06E-41	0.702892363	0.801	0.354	2.69E-37	Fib1	RUNX1	TRUE	FALSE
1.18E-41	0.603966753	0.525	0.148	2.98E-37	Fib1	PTPRG	FALSE	TRUE
1.69E-41	0.588642605	0.466	0.114	4.27E-37	Fib1	ANPEP	FALSE	TRUE
3.54E-41	0.473992448	0.653	0.199	8.94E-37	Fib1	IFITM1	FALSE	FALSE
6.56E-41	0.60145934	0.818	0.358	1.65E-36	Fib1	C1S	FALSE	FALSE
8.06E-41	0.271844898	0.22	0.013	2.03E-36	Fib1	ADAMTSL3	FALSE	FALSE
1.55E-40	0.629971185	0.669	0.246	3.92E-36	Fib1	RASSF8	FALSE	FALSE
3.10E-39	0.723440234	0.555	0.175	7.82E-35	Fib1	PDGFRA	FALSE	TRUE
3.15E-39	0.556291029	0.627	0.233	7.95E-35	Fib1	RECQL	FALSE	FALSE
6.42E-39	0.493903542	0.318	0.049	1.62E-34	Fib1	KDM7A-DT	FALSE	FALSE
6.96E-39	0.448324952	0.369	0.072	1.76E-34	Fib1	PTGFRN	FALSE	TRUE
1.04E-38	0.428986259	0.326	0.05	2.61E-34	Fib1	TMTC1	FALSE	FALSE
1.14E-38	0.786857398	0.75	0.315	2.88E-34	Fib1	SOD3	FALSE	FALSE
1.36E-38	0.581291547	0.746	0.273	3.43E-34	Fib1	NT5E	FALSE	TRUE
1.51E-38	0.902906605	0.555	0.179	3.82E-34	Fib1	CRISPLD2	FALSE	FALSE
3.71E-38	0.484029098	0.585	0.191	9.37E-34	Fib1	ANTXR1	FALSE	TRUE
1.77E-37	0.440603619	0.373	0.074	4.46E-33	Fib1	PTGFR	FALSE	TRUE
1.81E-37	0.683332566	0.847	0.41	4.58E-33	Fib1	HIF1A	TRUE	FALSE
2.57E-37	0.856563379	0.966	0.765	6.49E-33	Fib1	NEAT1	FALSE	FALSE
3.15E-37	0.528905181	0.699	0.262	7.95E-33	Fib1	LTBP1	FALSE	FALSE
3.60E-37	0.616406887	0.894	0.44	9.09E-33	Fib1	BZW1	FALSE	FALSE
6.73E-37	0.354334824	0.297	0.042	1.70E-32	Fib1	PDE1A	FALSE	FALSE
2.71E-36	0.629713794	0.835	0.396	6.85E-32	Fib1	IFI16	FALSE	FALSE
4.51E-36	0.610734034	0.551	0.17	1.14E-31	Fib1	CDH11	FALSE	TRUE
6.41E-36	0.635704	0.936	0.522	1.62E-31	Fib1	TIMP2	FALSE	FALSE
1.11E-35	0.69606128	0.691	0.269	2.79E-31	Fib1	EDIL3	FALSE	FALSE
1.62E-35	0.639348555	0.716	0.299	4.10E-31	Fib1	FBN1	FALSE	FALSE

1.85E-35	0.466603819	0.339	0.062	4.67E-31	Fib1	PDE3A	FALSE	FALSE
4.53E-35	0.721621543	0.411	0.1	1.14E-30	Fib1	BASP1	FALSE	FALSE
8.02E-35	0.462854652	0.288	0.043	2.02E-30	Fib1	SCARA5	FALSE	TRUE
8.32E-35	0.550135797	0.758	0.34	2.10E-30	Fib1	MRC2	FALSE	TRUE
9.75E-35	0.454357378	0.398	0.095	2.46E-30	Fib1	FLRT2	FALSE	TRUE
1.96E-34	0.431110019	0.424	0.107	4.94E-30	Fib1	AKR1C3	FALSE	FALSE
2.21E-34	0.851050353	0.852	0.441	5.57E-30	Fib1	VCAN	FALSE	FALSE
2.23E-34	0.563395932	0.653	0.267	5.62E-30	Fib1	DAB2	TRUE	FALSE
2.60E-34	0.498937576	0.25	0.032	6.57E-30	Fib1	ABHD17C	FALSE	FALSE
3.14E-34	0.408836488	0.398	0.096	7.93E-30	Fib1	BNC2	TRUE	FALSE
3.72E-34	0.893851217	0.576	0.217	9.38E-30	Fib1	GLRX	FALSE	FALSE
4.40E-34	0.464664769	0.22	0.021	1.11E-29	Fib1	NTRK3	FALSE	TRUE
6.10E-34	0.616826585	0.623	0.246	1.54E-29	Fib1	FAM20C	FALSE	FALSE
8.34E-34	0.572348533	0.648	0.266	2.10E-29	Fib1	IL1R1	FALSE	TRUE
1.05E-33	0.412959416	0.678	0.277	2.65E-29	Fib1	KDSR	FALSE	FALSE
1.97E-33	0.658489912	0.682	0.303	4.96E-29	Fib1	SKIL	TRUE	FALSE
2.80E-33	0.336672802	0.462	0.129	7.08E-29	Fib1	LPAR1	FALSE	TRUE
3.81E-33	0.692158958	0.572	0.208	9.62E-29	Fib1	ARRDC3	FALSE	FALSE
4.51E-33	0.37748524	0.343	0.071	1.14E-28	Fib1	TMEM204	FALSE	TRUE
6.82E-33	0.31789566	0.275	0.04	1.72E-28	Fib1	ARHGAP6	FALSE	FALSE
1.07E-32	0.571371392	0.445	0.137	2.69E-28	Fib1	SYNJ2	FALSE	FALSE
2.33E-32	0.700396951	0.322	0.062	5.87E-28	Fib1	THBD	FALSE	TRUE
3.02E-32	0.530594672	0.657	0.281	7.62E-28	Fib1	B4GALT1	FALSE	FALSE
8.62E-32	0.364261333	0.288	0.049	2.18E-27	Fib1	ETV1	TRUE	FALSE
8.88E-32	0.525130893	0.784	0.362	2.24E-27	Fib1	GPX1	FALSE	FALSE
1.13E-31	0.524219721	0.564	0.222	2.86E-27	Fib1	CD276	FALSE	TRUE
1.21E-31	0.591534778	0.436	0.124	3.04E-27	Fib1	ADAMTS6	FALSE	FALSE
1.46E-31	0.729155852	0.597	0.245	3.69E-27	Fib1	SLC20A1	FALSE	FALSE
2.26E-31	0.460742456	0.36	0.087	5.69E-27	Fib1	TRAF3IP2	FALSE	FALSE
2.46E-31	0.504958819	0.479	0.138	6.20E-27	Fib1	SPRY1	FALSE	FALSE
2.91E-31	0.752754151	0.258	0.039	7.35E-27	Fib1	SFRP1	FALSE	FALSE
1.66E-30	0.538218089	0.873	0.418	4.18E-26	Fib1	CD44	FALSE	TRUE
1.99E-30	0.784961698	0.606	0.249	5.01E-26	Fib1	NAMPT	FALSE	FALSE
2.16E-30	0.373634844	0.28	0.049	5.46E-26	Fib1	STEAP2	FALSE	FALSE
2.43E-30	0.460468524	0.775	0.364	6.12E-26	Fib1	TMEM167A	FALSE	FALSE
2.59E-30	0.991640999	0.504	0.173	6.53E-26	Fib1	FGF7	FALSE	FALSE
2.81E-30	0.401980968	0.513	0.175	7.08E-26	Fib1	MVP	FALSE	FALSE
3.84E-30	0.444985159	0.636	0.26	9.69E-26	Fib1	TYMP	FALSE	FALSE
8.41E-30	1.379190696	0.822	0.465	2.12E-25	Fib1	IGFBP5	FALSE	FALSE
8.77E-30	0.423063178	0.275	0.049	2.21E-25	Fib1	FAM20A	FALSE	FALSE
9.52E-30	0.371465741	0.695	0.31	2.40E-25	Fib1	MXRA7	FALSE	FALSE
1.69E-29	0.437661406	0.585	0.225	4.27E-25	Fib1	OSMR	FALSE	TRUE
1.94E-29	0.441218854	0.771	0.369	4.90E-25	Fib1	TM9SF3	FALSE	TRUE
2.12E-29	0.372305039	0.356	0.087	5.34E-25	Fib1	SULF2	FALSE	FALSE
2.75E-29	0.508832752	0.809	0.413	6.94E-25	Fib1	SELENOS	FALSE	FALSE
4.52E-29	0.630002882	0.941	0.609	1.14E-24	Fib1	NDUFA4L2	FALSE	FALSE
7.30E-29	0.429773015	0.322	0.075	1.84E-24	Fib1	HRH1	FALSE	TRUE
9.44E-29	0.485132952	0.661	0.279	2.38E-24	Fib1	SLC25A37	FALSE	FALSE
1.08E-28	0.61024481	0.695	0.318	2.72E-24	Fib1	PLAUR	FALSE	TRUE
3.89E-28	0.459506048	0.623	0.261	9.81E-24	Fib1	ITGB5	FALSE	TRUE
4.33E-28	0.299461889	0.555	0.209	1.09E-23	Fib1	MAP3K20	FALSE	FALSE
4.75E-28	0.471975508	0.661	0.303	1.20E-23	Fib1	DNAJC3	FALSE	FALSE
5.27E-28	0.636408608	0.729	0.333	1.33E-23	Fib1	SOX4	TRUE	FALSE
5.64E-28	0.378593611	0.703	0.32	1.42E-23	Fib1	FOXP1	TRUE	FALSE
5.94E-28	0.413136686	0.636	0.285	1.50E-23	Fib1	XBP1	TRUE	FALSE
8.68E-28	0.380899876	0.534	0.208	2.19E-23	Fib1	SNX9	FALSE	FALSE
1.16E-27	0.259706647	0.364	0.095	2.93E-23	Fib1	C16orf45	FALSE	FALSE
1.21E-27	0.526558922	0.487	0.169	3.04E-23	Fib1	GFPT2	FALSE	FALSE
1.29E-27	0.431809989	0.555	0.22	3.25E-23	Fib1	FAM89B	FALSE	FALSE
1.63E-27	0.278217663	0.691	0.31	4.10E-23	Fib1	CYBRD1	FALSE	FALSE
1.72E-27	0.543107684	0.551	0.208	4.33E-23	Fib1	THBS2	FALSE	FALSE
2.21E-27	0.267185562	0.22	0.032	5.57E-23	Fib1	PRICKLE1	FALSE	FALSE
2.31E-27	0.753569623	0.208	0.026	5.82E-23	Fib1	DNASE1L3	FALSE	FALSE
4.61E-27	0.391040353	0.458	0.157	1.16E-22	Fib1	RNF24	FALSE	FALSE
5.65E-27	0.492325635	0.564	0.237	1.43E-22	Fib1	CSGALNACT2	FALSE	FALSE
8.49E-27	0.399011258	0.462	0.15	2.14E-22	Fib1	USP53	FALSE	FALSE
8.92E-27	0.403303487	0.729	0.343	2.25E-22	Fib1	SPCS3	FALSE	FALSE
1.04E-26	0.450372625	0.86	0.437	2.64E-22	Fib1	CEBPB	TRUE	FALSE
1.38E-26	0.286206436	0.864	0.442	3.48E-22	Fib1	SERPING1	FALSE	FALSE
1.67E-26	0.554394813	0.953	0.635	4.21E-22	Fib1	IFITM2	FALSE	FALSE
1.85E-26	0.370053496	0.483	0.168	4.66E-22	Fib1	CSGALNACT1	FALSE	FALSE
4.24E-26	0.402358227	0.674	0.312	1.07E-21	Fib1	PPFIBP1	FALSE	FALSE
4.97E-26	0.357266928	0.242	0.042	1.25E-21	Fib1	STMN3	FALSE	FALSE
5.24E-26	0.320398847	0.322	0.082	1.32E-21	Fib1	WBP1L	FALSE	FALSE
1.33E-25	0.599813898	0.466	0.176	3.36E-21	Fib1	ACSL4	FALSE	FALSE
1.44E-25	0.41962258	0.547	0.233	3.62E-21	Fib1	NOTCH2	FALSE	TRUE
1.44E-25	0.385115231	0.5	0.193	3.63E-21	Fib1	CTBS	FALSE	FALSE
1.67E-25	0.404333521	0.941	0.593	4.22E-21	Fib1	SH3BGRL3	FALSE	FALSE
2.00E-25	0.296356794	0.203	0.028	5.03E-21	Fib1	HELLPAR	FALSE	FALSE
2.52E-25	0.433753523	0.831	0.452	6.36E-21	Fib1	ARL6IP5	FALSE	FALSE
3.08E-25	0.292887486	0.72	0.312	7.77E-21	Fib1	ANGPTL4	FALSE	FALSE
4.25E-25	0.34770929	0.708	0.348	1.07E-20	Fib1	SYNCRIP	FALSE	FALSE
5.20E-25	0.369403797	0.521	0.205	1.31E-20	Fib1	PHC2	FALSE	FALSE
6.59E-25	0.393153454	0.805	0.435	1.66E-20	Fib1	UBXN4	FALSE	FALSE
8.14E-25	0.373466627	0.534	0.218	2.05E-20	Fib1	SKI	TRUE	FALSE
8.15E-25	0.328223212	0.538	0.228	2.06E-20	Fib1	MPG	FALSE	FALSE

1.19E-24	0.403622887	0.78	0.387	3.01E-20	Fib1	LMAN1	FALSE	FALSE
1.21E-24	0.339481629	0.475	0.18	3.06E-20	Fib1	NUMB	FALSE	FALSE
1.22E-24	0.396723081	0.606	0.276	3.09E-20	Fib1	DHRS7	FALSE	FALSE
1.33E-24	0.399901344	0.725	0.367	3.35E-20	Fib1	MSN	FALSE	FALSE
1.76E-24	0.877346243	0.699	0.364	4.43E-20	Fib1	ID1	TRUE	FALSE
2.48E-24	0.393061448	0.572	0.252	6.27E-20	Fib1	DDR2	FALSE	TRUE
2.54E-24	0.343609194	0.508	0.202	6.40E-20	Fib1	SFT2D2	FALSE	FALSE
3.06E-24	0.356423956	0.288	0.069	7.72E-20	Fib1	SMAD9	TRUE	FALSE
4.77E-24	0.356988531	0.559	0.246	1.20E-19	Fib1	CUX1	TRUE	FALSE
6.12E-24	0.361385134	0.784	0.408	1.55E-19	Fib1	TMEM50A	FALSE	FALSE
8.40E-24	0.343710185	0.619	0.29	2.12E-19	Fib1	HM13	FALSE	TRUE
1.06E-23	0.517821452	0.547	0.245	2.67E-19	Fib1	VASN	FALSE	TRUE
1.30E-23	0.36343206	0.292	0.071	3.27E-19	Fib1	SAMD11	FALSE	FALSE
1.91E-23	0.349119495	0.941	0.577	4.82E-19	Fib1	YBX3	TRUE	FALSE
2.30E-23	0.40918735	0.415	0.15	5.81E-19	Fib1	PHACTR2	FALSE	FALSE
2.77E-23	0.669802963	0.525	0.234	6.98E-19	Fib1	CHMP1B	FALSE	FALSE
2.78E-23	0.429624516	0.504	0.209	7.01E-19	Fib1	CHSY1	FALSE	FALSE
3.01E-23	0.341381057	0.763	0.415	7.60E-19	Fib1	NFIC	TRUE	FALSE
3.22E-23	0.341786057	0.551	0.246	8.12E-19	Fib1	TMEM87A	FALSE	TRUE
4.18E-23	0.306476646	0.496	0.195	1.06E-18	Fib1	SH3BP5	FALSE	FALSE
4.84E-23	0.288002132	0.407	0.143	1.22E-18	Fib1	DESI2	FALSE	FALSE
6.03E-23	0.45630647	0.5	0.212	1.52E-18	Fib1	STK24	FALSE	FALSE
7.59E-23	0.314093535	0.449	0.173	1.92E-18	Fib1	MAPKAPK2	FALSE	FALSE
9.73E-23	0.637634187	0.606	0.27	2.46E-18	Fib1	RBP4	FALSE	FALSE
1.26E-22	0.298618076	0.483	0.198	3.18E-18	Fib1	IL13RA1	FALSE	TRUE
1.63E-22	0.414309438	0.538	0.238	4.10E-18	Fib1	KLF3	TRUE	FALSE
2.39E-22	0.345247041	0.669	0.334	6.03E-18	Fib1	REXO2	FALSE	FALSE
2.41E-22	0.352323003	0.517	0.219	6.08E-18	Fib1	CHPF	FALSE	FALSE
2.49E-22	0.323895696	0.398	0.142	6.29E-18	Fib1	MCUB	FALSE	FALSE
4.26E-22	0.438238592	0.441	0.178	1.08E-17	Fib1	DPYSL3	FALSE	FALSE
4.86E-22	0.366689548	0.525	0.236	1.23E-17	Fib1	SRPRA	FALSE	FALSE
6.17E-22	0.258125286	0.258	0.062	1.56E-17	Fib1	TWIST2	TRUE	FALSE
6.72E-22	0.307915972	0.373	0.116	1.70E-17	Fib1	CLEC2B	FALSE	FALSE
7.45E-22	0.306388492	0.691	0.344	1.88E-17	Fib1	STAT3	TRUE	FALSE
7.47E-22	0.283283857	0.394	0.142	1.88E-17	Fib1	TMEM173	FALSE	FALSE
9.37E-22	0.279881935	0.771	0.421	2.36E-17	Fib1	SQSTM1	FALSE	FALSE
9.90E-22	0.264447666	0.492	0.197	2.50E-17	Fib1	CFLAR	FALSE	FALSE
1.10E-21	0.352022332	0.754	0.393	2.77E-17	Fib1	PTP4A2	FALSE	FALSE
1.21E-21	0.269111955	0.657	0.314	3.04E-17	Fib1	VGLL4	FALSE	FALSE
1.46E-21	0.319189798	0.513	0.208	3.67E-17	Fib1	ECM1	FALSE	FALSE
1.56E-21	0.321523389	0.686	0.347	3.93E-17	Fib1	NFE2L2	TRUE	FALSE
2.21E-21	0.306891487	0.665	0.334	5.57E-17	Fib1	AKAP13	FALSE	FALSE
2.38E-21	0.320902904	0.564	0.257	6.01E-17	Fib1	PCMTD1	FALSE	FALSE
3.26E-21	0.293475926	0.242	0.055	8.24E-17	Fib1	TBX15	TRUE	FALSE
3.44E-21	0.295179313	0.839	0.452	8.69E-17	Fib1	SPCS2	FALSE	FALSE
5.49E-21	0.278449219	0.271	0.069	1.38E-16	Fib1	C1RL	FALSE	FALSE
5.74E-21	0.384209964	0.712	0.397	1.45E-16	Fib1	GSTO1	FALSE	FALSE
1.06E-20	0.289215803	0.758	0.425	2.68E-16	Fib1	TMBIM4	FALSE	FALSE
1.09E-20	0.428387115	0.377	0.135	2.74E-16	Fib1	PLEKHH2	FALSE	FALSE
1.29E-20	0.28454668	0.28	0.077	3.27E-16	Fib1	PRKAR2B	FALSE	FALSE
1.38E-20	0.318088734	0.538	0.245	3.49E-16	Fib1	CASC4	FALSE	FALSE
2.08E-20	0.53555166	0.212	0.045	5.25E-16	Fib1	FJX1	FALSE	FALSE
2.15E-20	0.277853229	0.419	0.164	5.42E-16	Fib1	TPBG	FALSE	TRUE
2.50E-20	0.303243083	0.572	0.275	6.32E-16	Fib1	CFAP97	FALSE	FALSE
2.73E-20	0.374458284	0.466	0.197	6.89E-16	Fib1	ZMIZ1	FALSE	FALSE
2.75E-20	0.309909808	0.614	0.312	6.95E-16	Fib1	SDCBP	FALSE	FALSE
3.18E-20	0.300422335	0.534	0.242	8.03E-16	Fib1	MAP3K2	FALSE	FALSE
3.41E-20	0.407261472	0.343	0.108	8.60E-16	Fib1	AKR1C2	FALSE	FALSE
4.87E-20	0.258723806	0.699	0.366	1.23E-15	Fib1	ATP6AP2	FALSE	FALSE
5.22E-20	0.321357109	0.775	0.442	1.32E-15	Fib1	PRRC2C	FALSE	FALSE
5.86E-20	0.334815383	0.301	0.091	1.48E-15	Fib1	ITPRIP	FALSE	FALSE
7.01E-20	0.337974266	0.953	0.663	1.77E-15	Fib1	DDX5	FALSE	FALSE
7.39E-20	0.354663894	0.809	0.478	1.86E-15	Fib1	COL3A1	FALSE	FALSE
9.92E-20	0.379314823	0.699	0.368	2.50E-15	Fib1	IGFBP6	FALSE	FALSE
1.21E-19	0.362015819	0.725	0.407	3.06E-15	Fib1	PRKAR1A	FALSE	FALSE
1.75E-19	0.268995555	0.555	0.267	4.41E-15	Fib1	RAB21	FALSE	FALSE
1.83E-19	0.280069441	0.504	0.22	4.62E-15	Fib1	DLGAP4	FALSE	FALSE
1.83E-19	0.313579	0.466	0.191	4.63E-15	Fib1	NR4A2	TRUE	FALSE
2.00E-19	0.319134242	0.462	0.195	5.06E-15	Fib1	REV3L	FALSE	FALSE
2.05E-19	0.349775676	0.369	0.131	5.17E-15	Fib1	XYLT1	FALSE	FALSE
2.14E-19	0.326597981	0.441	0.181	5.40E-15	Fib1	FAT1	FALSE	TRUE
2.32E-19	0.29591823	0.441	0.191	5.86E-15	Fib1	NQO2	FALSE	FALSE
2.46E-19	0.336106758	0.661	0.357	6.21E-15	Fib1	ANKRD12	FALSE	FALSE
2.59E-19	0.256225643	0.581	0.282	6.53E-15	Fib1	SMAD5	TRUE	FALSE
3.89E-19	0.288269277	0.343	0.116	9.83E-15	Fib1	CPT1A	FALSE	FALSE
3.90E-19	0.277211961	0.453	0.187	9.85E-15	Fib1	PLSCR1	TRUE	FALSE
4.28E-19	0.336671958	0.691	0.371	1.08E-14	Fib1	CD164	FALSE	TRUE
4.51E-19	0.47164607	0.373	0.138	1.14E-14	Fib1	GPRC5A	FALSE	TRUE
5.28E-19	0.347361195	0.801	0.431	1.33E-14	Fib1	PPP1R14B	FALSE	FALSE
5.29E-19	0.326700358	0.589	0.304	1.34E-14	Fib1	ITPRIPL2	FALSE	FALSE
5.48E-19	0.315643378	0.581	0.29	1.38E-14	Fib1	XRN2	FALSE	FALSE
6.15E-19	0.375187577	0.288	0.089	1.55E-14	Fib1	ARRDC2	FALSE	FALSE
6.51E-19	0.31374301	0.585	0.293	1.64E-14	Fib1	WWTR1	FALSE	FALSE
6.88E-19	0.840766662	0.47	0.201	1.74E-14	Fib1	IGFBP3	FALSE	FALSE
9.92E-19	0.364835079	0.352	0.127	2.50E-14	Fib1	FOXD1	TRUE	FALSE
1.02E-18	0.315108954	0.373	0.139	2.57E-14	Fib1	ACVR1	FALSE	TRUE

1.04E-18	0.250392024	0.661	0.335	2.62E-14	Fib1	ARPC1B	FALSE	FALSE
1.06E-18	0.264736015	0.386	0.154	2.67E-14	Fib1	ABRACL	FALSE	FALSE
1.13E-18	0.337722797	0.555	0.286	2.85E-14	Fib1	STAU1	FALSE	FALSE
1.25E-18	0.38205645	0.216	0.05	3.14E-14	Fib1	TMEM100	FALSE	FALSE
1.31E-18	0.276693716	0.449	0.188	3.31E-14	Fib1	KANK2	FALSE	FALSE
1.47E-18	0.264755491	0.39	0.144	3.70E-14	Fib1	FYN	FALSE	FALSE
1.53E-18	0.297001903	0.644	0.328	3.87E-14	Fib1	PDIA4	FALSE	FALSE
1.54E-18	0.329891378	0.462	0.201	3.88E-14	Fib1	TANK	FALSE	FALSE
2.29E-18	0.28058351	0.551	0.256	5.78E-14	Fib1	FAM129B	FALSE	FALSE
2.38E-18	0.360645249	0.822	0.471	6.01E-14	Fib1	AEBP1	TRUE	FALSE
3.15E-18	0.402545802	0.606	0.31	7.94E-14	Fib1	CTSB	FALSE	FALSE
4.02E-18	0.296136061	0.271	0.082	1.01E-13	Fib1	FOXP4	TRUE	FALSE
4.84E-18	0.292019041	0.712	0.396	1.22E-13	Fib1	LEPROT	FALSE	FALSE
5.22E-18	0.387917624	0.369	0.134	1.32E-13	Fib1	RETREG1	FALSE	FALSE
5.87E-18	0.333738903	0.801	0.437	1.48E-13	Fib1	CALU	FALSE	FALSE
6.24E-18	0.537428313	0.564	0.288	1.57E-13	Fib1	RND3	FALSE	FALSE
7.24E-18	0.323109574	0.513	0.246	1.83E-13	Fib1	ARFGAP3	FALSE	FALSE
7.91E-18	0.316509753	0.275	0.085	2.00E-13	Fib1	SLC9B2	FALSE	FALSE
1.02E-17	0.250934064	0.322	0.111	2.58E-13	Fib1	BOC	FALSE	TRUE
1.15E-17	0.27166331	0.292	0.092	2.90E-13	Fib1	CTSO	FALSE	FALSE
1.20E-17	0.312280772	0.623	0.333	3.03E-13	Fib1	LIMS1	FALSE	FALSE
1.37E-17	0.279508292	0.432	0.193	3.46E-13	Fib1	IFNGR1	FALSE	TRUE
1.49E-17	0.295006939	0.771	0.449	3.77E-13	Fib1	ARF1	FALSE	FALSE
1.75E-17	0.25347023	0.695	0.365	4.41E-13	Fib1	PPP3CA	FALSE	FALSE
1.86E-17	0.289423451	0.309	0.101	4.69E-13	Fib1	PALMD	FALSE	FALSE
2.14E-17	0.392583919	0.436	0.196	5.39E-13	Fib1	EDEM3	FALSE	FALSE
2.34E-17	0.37371027	0.987	0.736	5.91E-13	Fib1	S100A11	FALSE	FALSE
2.35E-17	0.316276639	0.254	0.072	5.93E-13	Fib1	PALM2	FALSE	FALSE
2.49E-17	0.521347305	0.572	0.288	6.28E-13	Fib1	ID2	TRUE	FALSE
2.69E-17	0.336476289	0.339	0.127	6.78E-13	Fib1	SIPA1L1	FALSE	FALSE
3.30E-17	0.252247236	0.581	0.298	8.34E-13	Fib1	SERINC3	FALSE	TRUE
4.12E-17	0.317331345	0.394	0.158	1.04E-12	Fib1	ANGPTL2	FALSE	FALSE
4.56E-17	0.291089025	0.305	0.104	1.15E-12	Fib1	RASA3	FALSE	FALSE
4.83E-17	0.302892917	0.517	0.247	1.22E-12	Fib1	ATP2B1	FALSE	FALSE
5.25E-17	0.281219742	0.619	0.325	1.32E-12	Fib1	CLTC	FALSE	FALSE
6.05E-17	0.309400566	0.381	0.156	1.53E-12	Fib1	TAF11	FALSE	FALSE
6.63E-17	0.251338387	0.661	0.357	1.67E-12	Fib1	EIF3A	FALSE	FALSE
7.14E-17	0.27206898	0.242	0.068	1.80E-12	Fib1	CDK14	FALSE	FALSE
7.51E-17	0.378795505	0.386	0.155	1.90E-12	Fib1	PLPP3	FALSE	FALSE
8.34E-17	0.397289499	0.475	0.224	2.11E-12	Fib1	SDF2L1	FALSE	FALSE
9.45E-17	0.338222714	0.483	0.228	2.38E-12	Fib1	PAM	FALSE	TRUE
1.01E-16	0.291600293	0.742	0.411	2.54E-12	Fib1	VMP1	FALSE	FALSE
1.09E-16	0.281353941	0.661	0.349	2.74E-12	Fib1	HNRNPAB	FALSE	FALSE
1.09E-16	0.278372165	0.47	0.224	2.74E-12	Fib1	WASL	FALSE	FALSE
1.20E-16	0.251136409	0.436	0.198	3.02E-12	Fib1	LPGAT1	FALSE	FALSE
1.38E-16	0.275729726	0.513	0.257	3.49E-12	Fib1	DNAJC1	FALSE	FALSE
1.60E-16	0.275811016	0.212	0.055	4.03E-12	Fib1	SMAD6	TRUE	FALSE
1.63E-16	0.334014666	0.508	0.256	4.12E-12	Fib1	SYAP1	FALSE	FALSE
1.68E-16	0.264586741	0.453	0.21	4.24E-12	Fib1	GLUD1	FALSE	FALSE
1.75E-16	0.264419295	0.686	0.384	4.42E-12	Fib1	XRCC5	FALSE	FALSE
2.00E-16	0.273951698	0.797	0.445	5.05E-12	Fib1	YWHAB	FALSE	FALSE
2.24E-16	0.323204952	0.436	0.202	5.66E-12	Fib1	THUMPD1	FALSE	FALSE
2.59E-16	0.269683017	0.475	0.225	6.54E-12	Fib1	CLIP1	FALSE	FALSE
2.64E-16	0.264673605	0.309	0.111	6.66E-12	Fib1	DIP2C	FALSE	FALSE
3.19E-16	0.353353788	0.987	0.843	8.04E-12	Fib1	IFITM3	FALSE	FALSE
3.25E-16	0.335518888	0.487	0.232	8.19E-12	Fib1	ZBTB7A	TRUE	FALSE
3.75E-16	0.3360118	0.479	0.232	9.46E-12	Fib1	TCF7L2	TRUE	FALSE
3.78E-16	0.32042481	0.576	0.314	9.53E-12	Fib1	RAB10	FALSE	FALSE
4.62E-16	0.26450684	0.233	0.066	1.17E-11	Fib1	LDLRAD3	FALSE	TRUE
5.96E-16	0.253629986	0.492	0.241	1.50E-11	Fib1	PITPNB	FALSE	FALSE
8.17E-16	0.290638078	0.521	0.27	2.06E-11	Fib1	PLIN3	FALSE	FALSE
9.26E-16	0.250237975	0.254	0.08	2.34E-11	Fib1	FBH1	FALSE	FALSE
1.10E-15	0.365682409	0.432	0.188	2.77E-11	Fib1	TWIST1	TRUE	FALSE
1.36E-15	0.299195561	0.462	0.216	3.44E-11	Fib1	ZCCHC24	FALSE	FALSE
2.59E-15	0.269861953	0.661	0.354	6.54E-11	Fib1	ACTN1	FALSE	FALSE
2.65E-15	0.856222194	0.356	0.143	6.68E-11	Fib1	TNFAIP6	FALSE	FALSE
2.99E-15	0.298686143	0.424	0.191	7.53E-11	Fib1	SCARA3	FALSE	FALSE
3.38E-15	0.306508154	0.873	0.495	8.53E-11	Fib1	PDIA3	FALSE	FALSE
3.86E-15	0.276302879	0.453	0.224	9.75E-11	Fib1	SZRD1	FALSE	FALSE
4.20E-15	0.341716674	0.373	0.157	1.06E-10	Fib1	SOCS2	FALSE	FALSE
4.24E-15	0.352779522	0.284	0.1	1.07E-10	Fib1	IRX3	TRUE	FALSE
4.53E-15	0.29413553	0.492	0.224	1.14E-10	Fib1	MEG3	FALSE	FALSE
4.85E-15	0.34385183	0.907	0.59	1.22E-10	Fib1	NNMT	FALSE	FALSE
6.83E-15	0.539714243	0.356	0.153	1.72E-10	Fib1	PLAGL1	TRUE	FALSE
8.90E-15	0.274660237	0.564	0.3	2.25E-10	Fib1	UBE2K	TRUE	FALSE
9.29E-15	0.284453484	0.314	0.125	2.34E-10	Fib1	WDFY1	FALSE	FALSE
9.56E-15	0.367767568	0.258	0.088	2.41E-10	Fib1	SBNO2	FALSE	FALSE
9.96E-15	0.277281442	0.521	0.266	2.51E-10	Fib1	PDLIM7	FALSE	FALSE
1.15E-14	0.332065975	0.335	0.137	2.90E-10	Fib1	BAG2	FALSE	FALSE
1.33E-14	0.283392858	0.428	0.199	3.36E-10	Fib1	SLC4A7	FALSE	TRUE
1.43E-14	0.26612152	0.547	0.29	3.60E-10	Fib1	SEC61A1	FALSE	FALSE
1.64E-14	0.256673573	0.364	0.159	4.15E-10	Fib1	ANO6	FALSE	TRUE
1.70E-14	0.292781879	0.521	0.276	4.30E-10	Fib1	ATL3	FALSE	FALSE
1.77E-14	0.33589646	0.339	0.141	4.46E-10	Fib1	GABARAPL1	FALSE	FALSE
1.78E-14	0.411991185	0.432	0.207	4.50E-10	Fib1	GEM	FALSE	FALSE
1.88E-14	0.276696903	0.301	0.114	4.74E-10	Fib1	ZBED1	TRUE	FALSE

2.00E-14	0.262771871	0.271	0.097	5.06E-10	Fib1	SOAT1	FALSE	FALSE
2.52E-14	0.296141057	0.309	0.119	6.36E-10	Fib1	MAFG	TRUE	FALSE
3.32E-14	0.257059075	0.665	0.386	8.38E-10	Fib1	SEC31A	FALSE	FALSE
4.19E-14	0.380565891	0.839	0.509	1.06E-09	Fib1	HSPA5	TRUE	FALSE
4.20E-14	0.262880994	0.36	0.151	1.06E-09	Fib1	BCL3	TRUE	FALSE
4.89E-14	0.251787961	0.602	0.332	1.23E-09	Fib1	SSR1	FALSE	TRUE
4.92E-14	0.522505319	0.983	0.834	1.24E-09	Fib1	TIMP1	FALSE	FALSE
5.48E-14	0.330410858	0.576	0.31	1.38E-09	Fib1	MANF	FALSE	FALSE
7.40E-14	0.296158497	0.267	0.096	1.87E-09	Fib1	P4HA3	FALSE	FALSE
8.78E-14	0.297352222	0.428	0.206	2.22E-09	Fib1	ADK	FALSE	FALSE
9.61E-14	0.265854964	0.322	0.133	2.42E-09	Fib1	ITPR2	FALSE	FALSE
1.03E-13	0.306392828	0.297	0.117	2.60E-09	Fib1	UNC5B	FALSE	TRUE
1.04E-13	0.287091483	0.564	0.323	2.63E-09	Fib1	LMO4	FALSE	FALSE
1.18E-13	0.257813067	0.483	0.253	2.98E-09	Fib1	ARHGAP21	FALSE	FALSE
1.19E-13	0.284301231	0.369	0.165	3.00E-09	Fib1	CBLB	FALSE	FALSE
1.20E-13	0.270440808	0.479	0.242	3.03E-09	Fib1	TMEM45A	FALSE	FALSE
1.45E-13	0.260590295	0.254	0.083	3.66E-09	Fib1	SGIP1	FALSE	FALSE
1.57E-13	0.297761174	0.462	0.22	3.95E-09	Fib1	CP	FALSE	FALSE
2.16E-13	0.260969679	0.576	0.325	5.45E-09	Fib1	PRPF4B	FALSE	FALSE
2.63E-13	0.260630892	0.322	0.136	6.64E-09	Fib1	ATF6	TRUE	FALSE
2.97E-13	0.878468896	0.403	0.199	7.50E-09	Fib1	ACKR3	FALSE	TRUE
3.82E-13	0.31679289	0.534	0.275	9.64E-09	Fib1	TFPI	FALSE	TRUE
4.38E-13	0.255960867	0.237	0.08	1.11E-08	Fib1	FAM110B	FALSE	FALSE
4.61E-13	0.255920968	0.28	0.11	1.16E-08	Fib1	PLXNA1	FALSE	TRUE
5.75E-13	0.252671712	0.466	0.239	1.45E-08	Fib1	GOLT1B	FALSE	FALSE
6.65E-13	0.303079457	0.288	0.12	1.68E-08	Fib1	ST3GAL1	FALSE	FALSE
7.28E-13	0.274103527	0.309	0.129	1.84E-08	Fib1	RASSF8-AS1	FALSE	FALSE
1.27E-12	0.272920303	0.258	0.096	3.21E-08	Fib1	SPOCK1	FALSE	FALSE
1.34E-12	0.281614121	0.445	0.235	3.37E-08	Fib1	MIA3	FALSE	FALSE
1.83E-12	0.391850191	0.364	0.165	4.62E-08	Fib1	GAS1	FALSE	TRUE
2.38E-12	0.353016343	0.653	0.393	6.02E-08	Fib1	SOD2	FALSE	FALSE
2.73E-12	0.302618714	0.309	0.132	6.89E-08	Fib1	SMOX	FALSE	FALSE
2.88E-12	0.253442735	0.292	0.121	7.26E-08	Fib1	HIVEP1	TRUE	FALSE
3.15E-12	0.255710323	0.39	0.195	7.95E-08	Fib1	SPRED1	FALSE	FALSE
4.03E-12	0.36392021	0.958	0.794	1.02E-07	Fib1	MT-ND2	FALSE	FALSE
5.74E-12	0.286820826	0.339	0.158	1.45E-07	Fib1	SETD7	FALSE	FALSE
6.65E-12	0.257539304	0.369	0.181	1.68E-07	Fib1	SRFBP1	FALSE	FALSE
6.94E-12	0.263336679	0.352	0.167	1.75E-07	Fib1	UGGT2	FALSE	FALSE
1.34E-11	0.279220367	0.254	0.102	3.39E-07	Fib1	ABL1	TRUE	FALSE
1.44E-11	0.723180625	0.28	0.112	3.65E-07	Fib1	CHI3L1	FALSE	FALSE
1.48E-11	0.262286924	0.326	0.152	3.73E-07	Fib1	TUT7	FALSE	FALSE
1.62E-11	0.310283437	0.453	0.24	4.09E-07	Fib1	TGFBR1	FALSE	TRUE
2.68E-11	0.452351787	0.233	0.086	6.77E-07	Fib1	HMOX1	FALSE	FALSE
1.16E-10	0.269743511	0.39	0.209	2.93E-06	Fib1	HPF1	FALSE	FALSE
3.69E-10	0.305452381	0.28	0.13	9.32E-06	Fib1	TMEM181	FALSE	FALSE
6.58E-10	0.649745113	0.322	0.159	1.66E-05	Fib1	RASD1	FALSE	FALSE
8.84E-10	0.263357384	0.559	0.329	2.23E-05	Fib1	BHLHE40	TRUE	FALSE
1.48E-09	0.261937795	0.771	0.47	3.73E-05	Fib1	COL6A3	FALSE	FALSE
1.61E-09	0.265397849	0.606	0.363	4.06E-05	Fib1	TNFRSF12A	FALSE	FALSE
1.09E-08	0.403813547	0.979	0.924	0.000275284	Fib1	RPL39	FALSE	FALSE
1.99E-08	0.342343677	0.432	0.257	0.000501495	Fib1	SGK1	FALSE	FALSE
2.58E-08	0.286560826	0.305	0.155	0.000651301	Fib1	ABCA1	FALSE	TRUE
8.89E-08	0.284751917	0.314	0.171	0.002244382	Fib1	DNAJB9	FALSE	FALSE
4.18E-75	0.833518193	0.944	0.907	1.06E-70	Fib2	MGP	FALSE	FALSE
4.14E-61	1.201116435	0.871	0.876	1.05E-56	Fib2	COMP	FALSE	FALSE
4.06E-55	0.979122269	0.857	0.885	1.02E-50	Fib2	MT1X	FALSE	FALSE
7.63E-47	0.582063466	0.941	0.963	1.92E-42	Fib2	RPL30	FALSE	FALSE
5.38E-45	0.923148037	0.934	0.937	1.36E-40	Fib2	DCN	FALSE	FALSE
5.32E-44	0.51832043	0.946	0.982	1.34E-39	Fib2	RPS27A	FALSE	FALSE
4.87E-41	0.534043612	0.952	0.966	1.23E-36	Fib2	RPL34	FALSE	FALSE
1.29E-39	0.545088572	0.937	0.964	3.24E-35	Fib2	RPS28	FALSE	FALSE
1.99E-39	0.451341821	0.978	0.987	5.03E-35	Fib2	RPL10	FALSE	FALSE
1.55E-35	0.468220939	0.951	0.976	3.91E-31	Fib2	RPS27	FALSE	FALSE
7.54E-35	0.496065343	0.942	0.976	1.90E-30	Fib2	RPL32	FALSE	FALSE
9.78E-35	0.412153417	0.963	0.982	2.47E-30	Fib2	RPL41	FALSE	FALSE
1.82E-34	0.543482525	0.935	0.962	4.58E-30	Fib2	FTL	FALSE	FALSE
9.79E-33	0.748489444	0.885	0.927	2.47E-28	Fib2	CLU	FALSE	FALSE
5.98E-30	0.514741143	0.878	0.943	1.51E-25	Fib2	RPL8	FALSE	FALSE
1.09E-29	0.410484726	0.964	0.969	2.76E-25	Fib2	RPS12	FALSE	FALSE
1.92E-29	2.012791524	0.621	0.53	4.85E-25	Fib2	HBB	FALSE	FALSE
4.17E-29	0.479154219	0.91	0.951	1.05E-24	Fib2	RPL11	FALSE	FALSE
1.11E-28	0.431488937	0.934	0.969	2.80E-24	Fib2	RPS14	FALSE	FALSE
4.37E-27	0.39273323	0.952	0.978	1.10E-22	Fib2	RPS18	FALSE	FALSE
5.09E-27	0.3663736	0.954	0.985	1.28E-22	Fib2	RPL13	FALSE	FALSE
1.51E-25	0.695902126	0.795	0.788	3.81E-21	Fib2	PRELP	FALSE	FALSE
6.59E-25	0.500911103	0.856	0.912	1.66E-20	Fib2	RPS3	FALSE	FALSE
1.54E-24	0.490951996	0.859	0.92	3.90E-20	Fib2	RPS4X	TRUE	FALSE
5.38E-24	0.467204593	0.851	0.936	1.36E-19	Fib2	RPL35A	FALSE	FALSE
7.86E-24	0.327420195	0.975	0.991	1.98E-19	Fib2	RPLP1	FALSE	FALSE
5.43E-23	0.859797006	0.565	0.402	1.37E-18	Fib2	SPARCL1	FALSE	FALSE
8.26E-23	0.422216524	0.903	0.949	2.08E-18	Fib2	RPS3A	FALSE	FALSE
2.43E-21	1.541797834	0.489	0.336	6.13E-17	Fib2	PLA2G2A	FALSE	FALSE
6.01E-21	1.251500802	0.54	0.455	1.52E-16	Fib2	C2orf40	FALSE	FALSE
1.41E-20	0.42037384	0.876	0.953	3.56E-16	Fib2	RPL26	FALSE	FALSE
1.43E-20	1.772986674	0.458	0.321	3.60E-16	Fib2	HBA2	FALSE	FALSE
3.62E-20	0.387506875	0.902	0.972	9.13E-16	Fib2	RPS23	FALSE	FALSE

3.80E-20	0.47149941	0.866	0.914	9.59E-16	Fib2	TMSB4X	FALSE	FALSE
3.84E-20	0.460778563	0.847	0.927	9.68E-16	Fib2	RPL18	FALSE	FALSE
9.65E-20	0.352212542	0.932	0.971	2.44E-15	Fib2	RPL37	FALSE	FALSE
1.21E-19	0.428519439	0.852	0.93	3.04E-15	Fib2	RPL29	FALSE	FALSE
3.47E-19	0.444705016	0.827	0.943	8.76E-15	Fib2	FAU	FALSE	FALSE
4.37E-18	0.271579956	0.202	0.521	1.10E-13	Fib2	DDT	FALSE	FALSE
1.67E-17	0.383105544	0.864	0.935	4.21E-13	Fib2	RPS13	FALSE	FALSE
2.10E-17	1.050097049	0.559	0.52	5.30E-13	Fib2	COL2A1	FALSE	FALSE
4.10E-17	0.346234526	0.903	0.944	1.04E-12	Fib2	RPL18A	FALSE	FALSE
6.00E-17	0.43543809	0.839	0.928	1.51E-12	Fib2	RPL24	FALSE	FALSE
9.30E-17	0.443019207	0.832	0.918	2.35E-12	Fib2	RPL9	FALSE	FALSE
1.67E-16	0.256384995	0.226	0.535	4.21E-12	Fib2	VEGFA	FALSE	FALSE
5.46E-16	0.287887994	0.2	0.497	1.38E-11	Fib2	AES	FALSE	FALSE
6.15E-16	0.253782194	0.182	0.458	1.55E-11	Fib2	UBXN1	TRUE	FALSE
6.15E-16	0.361563548	0.9	0.963	1.55E-11	Fib2	RPL19	FALSE	FALSE
1.02E-15	1.584835877	0.333	0.192	2.59E-11	Fib2	HBA1	FALSE	FALSE
1.50E-15	1.022755585	0.564	0.548	3.79E-11	Fib2	SCRG1	FALSE	FALSE
4.00E-15	0.330872851	0.903	0.946	1.01E-10	Fib2	RPS19	FALSE	FALSE
6.42E-15	0.353862622	0.154	0.404	1.62E-10	Fib2	ALKBH7	FALSE	FALSE
8.25E-15	0.705762251	0.727	0.831	2.08E-10	Fib2	IGFBP7	FALSE	FALSE
3.11E-14	1.745133205	0.491	0.41	7.85E-10	Fib2	APOD	FALSE	FALSE
4.17E-14	0.265607852	0.178	0.437	1.05E-09	Fib2	SMDT1	FALSE	FALSE
5.84E-14	0.375296606	0.82	0.927	1.47E-09	Fib2	RPL3	FALSE	FALSE
1.13E-13	0.326192364	0.878	0.948	2.86E-09	Fib2	RPL21	FALSE	FALSE
1.15E-13	0.256635771	0.275	0.616	2.89E-09	Fib2	FKBP1A	FALSE	FALSE
1.72E-13	0.360018134	0.815	0.936	4.33E-09	Fib2	RPL38	FALSE	FALSE
2.23E-13	0.284979379	0.949	0.978	5.63E-09	Fib2	TPT1	FALSE	FALSE
2.43E-13	0.315624827	0.146	0.372	6.13E-09	Fib2	FHL1	FALSE	FALSE
2.60E-13	0.285075723	0.238	0.535	6.56E-09	Fib2	RPL9P9	FALSE	FALSE
3.08E-13	0.325575464	0.153	0.381	7.78E-09	Fib2	GYPC	FALSE	TRUE
6.73E-13	0.298528202	0.246	0.548	1.70E-08	Fib2	H1FX	TRUE	FALSE
1.00E-12	0.812043153	0.577	0.577	2.52E-08	Fib2	ACAN	FALSE	FALSE
2.98E-12	0.258804091	0.063	0.211	7.52E-08	Fib2	NET1	FALSE	FALSE
2.98E-12	0.250609708	0.214	0.479	7.53E-08	Fib2	COX7A1	FALSE	FALSE
3.12E-12	0.388001868	0.771	0.911	7.86E-08	Fib2	NACA	FALSE	FALSE
5.39E-12	1.041279596	0.474	0.424	1.36E-07	Fib2	TAGLN	FALSE	FALSE
6.67E-12	0.335974544	0.214	0.479	1.68E-07	Fib2	SYF2	FALSE	FALSE
6.87E-12	0.455280561	0.735	0.872	1.73E-07	Fib2	RPS5	FALSE	FALSE
7.95E-12	0.309357465	0.898	0.959	2.01E-07	Fib2	RPS6	FALSE	FALSE
1.17E-11	0.315129225	0.141	0.346	2.95E-07	Fib2	BAG1	FALSE	FALSE
1.18E-11	0.311721038	0.846	0.931	2.97E-07	Fib2	RPL7A	FALSE	FALSE
4.92E-11	0.264314096	0.89	0.94	1.24E-06	Fib2	RPS2	FALSE	FALSE
1.23E-10	0.321081085	0.84	0.935	3.10E-06	Fib2	RPL36	FALSE	FALSE
2.27E-10	0.325113121	0.825	0.916	5.74E-06	Fib2	RPL14	FALSE	FALSE
2.28E-10	0.323626433	0.216	0.457	5.75E-06	Fib2	CRIP2	FALSE	FALSE
3.59E-10	0.546460833	0.654	0.83	9.05E-06	Fib2	RPS21	FALSE	FALSE
4.28E-10	0.37306995	0.8	0.926	1.08E-05	Fib2	RPL5	FALSE	FALSE
4.41E-10	0.416301137	0.256	0.538	1.11E-05	Fib2	NOP53	FALSE	FALSE
4.51E-10	0.374508187	0.75	0.889	1.14E-05	Fib2	RPL10A	FALSE	FALSE
5.97E-10	0.310355693	0.862	0.927	1.51E-05	Fib2	RPS15A	FALSE	FALSE
7.68E-10	0.317358824	0.297	0.609	1.94E-05	Fib2	RPL17	FALSE	FALSE
9.03E-10	0.297655607	0.265	0.544	2.28E-05	Fib2	LPP	FALSE	FALSE
1.08E-09	0.31454733	0.098	0.249	2.73E-05	Fib2	SORBS3	FALSE	FALSE
1.32E-09	0.259436739	0.35	0.709	3.34E-05	Fib2	CIRBP	FALSE	FALSE
1.49E-09	0.360595735	0.796	0.918	3.76E-05	Fib2	RPS7	FALSE	FALSE
2.62E-09	0.319955114	0.878	0.977	6.61E-05	Fib2	B2M	FALSE	FALSE
2.81E-09	0.304954999	0.301	0.596	7.09E-05	Fib2	NUPR1	FALSE	FALSE
2.88E-09	0.278248131	0.895	0.968	7.26E-05	Fib2	RPL12	FALSE	FALSE
3.48E-09	0.349571825	0.161	0.352	8.78E-05	Fib2	EPS8	FALSE	FALSE
4.64E-09	0.678529104	0.591	0.637	0.000117054	Fib2	ADIRF	FALSE	FALSE
9.65E-09	0.268205545	0.238	0.478	0.000243504	Fib2	KLF2	TRUE	FALSE
1.72E-08	0.271892052	0.34	0.665	0.000435148	Fib2	MGST3	FALSE	FALSE
3.14E-08	0.380568156	0.2	0.413	0.000792771	Fib2	SNHG8	FALSE	FALSE
4.32E-08	0.363581408	0.139	0.303	0.001090172	Fib2	SLC9A3R2	FALSE	FALSE
5.01E-08	0.272134453	0.144	0.304	0.001263768	Fib2	YPEL3	FALSE	FALSE
5.27E-08	0.267132076	0.846	0.918	0.001328795	Fib2	RPS16	FALSE	FALSE
6.37E-08	0.305514683	0.156	0.327	0.001608026	Fib2	SNHG7	FALSE	FALSE
1.31E-07	0.290717246	0.331	0.64	0.003315599	Fib2	ZFAS1	FALSE	FALSE
1.63E-07	0.5497776	0.194	0.393	0.004110544	Fib2	LRRC75A	FALSE	FALSE
1.64E-07	0.275220126	0.857	0.939	0.004134587	Fib2	RPL15	FALSE	FALSE
2.65E-07	0.295730955	0.127	0.269	0.00669844	Fib2	C1orf56	FALSE	FALSE
2.71E-07	0.254392482	0.178	0.351	0.00684905	Fib2	IFITM1	FALSE	FALSE
3.63E-07	0.298971203	0.362	0.699	0.009149035	Fib2	EIF3E	FALSE	FALSE
4.00E-07	0.286406425	0.812	0.922	0.01008438	Fib2	RPL23A	FALSE	FALSE
4.42E-07	0.339268264	0.798	0.917	0.011157077	Fib2	RPS9	FALSE	FALSE
7.22E-07	0.31676239	0.265	0.497	0.018223643	Fib2	TSC22D1	TRUE	FALSE
8.72E-07	0.278630343	0.825	0.922	0.022007511	Fib2	RPS11	FALSE	FALSE
1.43E-06	0.363810217	0.207	0.395	0.036167992	Fib2	EPAS1	TRUE	FALSE
7.76E-167	1.06238872	0.761	0.025	1.96E-162	Fib3	DNER	FALSE	TRUE
6.24E-134	0.773316326	0.732	0.041	1.57E-129	Fib3	DSG2	FALSE	TRUE
2.61E-127	1.164564001	0.838	0.073	6.59E-123	Fib3	MELTF	FALSE	FALSE
1.13E-122	1.161246114	0.817	0.074	2.85E-118	Fib3	SMOC1	FALSE	FALSE
1.24E-121	1.268946341	0.641	0.033	3.12E-117	Fib3	LAMB3	FALSE	FALSE
1.22E-120	1.324005464	0.951	0.137	3.09E-116	Fib3	SLC16A3	FALSE	FALSE
2.29E-119	1.866914494	0.958	0.151	5.77E-115	Fib3	TGM2	FALSE	FALSE
2.39E-118	1.118203401	0.873	0.102	6.04E-114	Fib3	LOXL2	FALSE	FALSE

4.53E-117	0.958159134	0.655	0.038	1.14E-112	Fib3	P3H2	FALSE	FALSE
1.21E-116	0.846272086	0.739	0.056	3.05E-112	Fib3	MALL	FALSE	FALSE
5.24E-114	1.922916186	0.803	0.081	1.32E-109	Fib3	S100A2	FALSE	FALSE
8.60E-105	1.839354447	0.817	0.093	2.17E-100	Fib3	CHI3L2	FALSE	FALSE
1.63E-101	1.368323664	0.88	0.126	4.10E-97	Fib3	KCNMA1	FALSE	FALSE
3.14E-101	0.568342208	0.5	0.02	7.92E-97	Fib3	DSC2	FALSE	TRUE
1.68E-100	1.342792689	0.754	0.085	4.24E-96	Fib3	TMEM158	FALSE	TRUE
4.69E-94	0.870857102	0.704	0.079	1.18E-89	Fib3	GALNT7	FALSE	FALSE
9.99E-94	1.03025153	0.81	0.117	2.52E-89	Fib3	ADAM12	FALSE	TRUE
2.93E-91	1.446999559	0.852	0.129	7.38E-87	Fib3	HAPLN1	FALSE	FALSE
5.24E-91	3.005604794	0.697	0.081	1.32E-86	Fib3	MMP3	FALSE	FALSE
5.73E-89	0.788018737	0.859	0.137	1.45E-84	Fib3	PFKP	FALSE	FALSE
1.24E-87	0.540111447	0.556	0.039	3.12E-83	Fib3	DSP	FALSE	FALSE
3.27E-86	1.342262215	0.493	0.029	8.24E-82	Fib3	LAMC2	FALSE	FALSE
5.39E-86	0.50443912	0.549	0.041	1.36E-81	Fib3	SUSD5	FALSE	TRUE
6.21E-86	1.216125829	0.944	0.207	1.57E-81	Fib3	ITGA5	FALSE	TRUE
2.37E-85	1.514160268	0.852	0.156	5.97E-81	Fib3	SERPINE1	FALSE	FALSE
1.87E-83	0.553076933	0.57	0.048	4.73E-79	Fib3	ADTRP	FALSE	FALSE
2.92E-83	1.804705641	1	0.369	7.37E-79	Fib3	PLOD2	FALSE	FALSE
3.22E-83	0.980907582	0.852	0.166	8.13E-79	Fib3	ACLY	FALSE	FALSE
3.85E-83	0.472925101	0.5	0.033	9.70E-79	Fib3	STEAP3	FALSE	FALSE
8.54E-83	0.539716259	0.57	0.048	2.16E-78	Fib3	NEBL	FALSE	FALSE
3.26E-82	0.897629785	0.732	0.105	8.22E-78	Fib3	CDK6	FALSE	FALSE
2.02E-81	0.279960445	0.345	0.007	5.11E-77	Fib3	PKP1	FALSE	FALSE
6.15E-81	0.622544562	0.465	0.027	1.55E-76	Fib3	AOC2	FALSE	FALSE
1.12E-80	0.97191307	0.887	0.192	2.83E-76	Fib3	GNPNAT1	FALSE	FALSE
8.02E-80	0.724750666	0.746	0.105	2.02E-75	Fib3	ENO2	FALSE	FALSE
3.49E-79	1.080514121	0.824	0.146	8.82E-75	Fib3	PDPN	FALSE	FALSE
1.11E-78	0.516120708	0.43	0.022	2.81E-74	Fib3	SFN	FALSE	FALSE
1.78E-78	0.698429112	0.838	0.147	4.48E-74	Fib3	QSOX1	FALSE	TRUE
2.26E-78	0.845696861	0.859	0.156	5.69E-74	Fib3	FGFRL1	FALSE	TRUE
3.10E-78	0.918995406	0.873	0.193	7.83E-74	Fib3	PLOD1	FALSE	FALSE
3.31E-78	1.024141919	0.775	0.125	8.35E-74	Fib3	CSPG4	FALSE	TRUE
1.95E-77	0.318646319	0.317	0.005	4.91E-73	Fib3	AC010343.3	FALSE	FALSE
2.51E-77	0.293446877	0.324	0.006	6.35E-73	Fib3	AL139220.2	FALSE	FALSE
3.36E-77	0.61136903	0.62	0.071	8.48E-73	Fib3	TNFRSF10D	FALSE	TRUE
1.06E-76	1.160303733	0.725	0.113	2.67E-72	Fib3	MSMO1	FALSE	FALSE
1.80E-76	0.688230992	0.634	0.073	4.55E-72	Fib3	FAM180A	FALSE	FALSE
2.74E-76	0.585183283	0.761	0.115	6.92E-72	Fib3	GPC6	FALSE	TRUE
3.11E-76	0.834643462	0.746	0.113	7.85E-72	Fib3	ATP1B1	FALSE	TRUE
5.83E-74	1.723941289	0.62	0.081	1.47E-69	Fib3	DCLK1	FALSE	FALSE
1.20E-73	0.585199036	0.5	0.041	3.03E-69	Fib3	SLC2A1	FALSE	TRUE
2.35E-73	0.847939573	0.845	0.171	5.93E-69	Fib3	ERO1A	FALSE	FALSE
3.19E-73	0.406685203	0.331	0.009	8.05E-69	Fib3	CDCP1	FALSE	TRUE
8.28E-73	0.81234432	0.711	0.101	2.09E-68	Fib3	SERPINA5	FALSE	FALSE
1.03E-72	0.598922373	0.5	0.041	2.59E-68	Fib3	LRRC8C	FALSE	FALSE
4.03E-72	0.423070003	0.458	0.033	1.02E-67	Fib3	ADCY7	FALSE	TRUE
4.77E-71	1.222632809	0.965	0.284	1.20E-66	Fib3	NT5E	FALSE	TRUE
5.54E-71	0.370480214	0.275	0.002	1.40E-66	Fib3	AL033397.2	FALSE	FALSE
7.83E-71	0.901190287	0.873	0.19	1.98E-66	Fib3	SDC4	FALSE	FALSE
1.52E-70	0.884602747	0.908	0.207	3.83E-66	Fib3	BCAT1	FALSE	FALSE
1.04E-69	0.656272332	0.775	0.133	2.61E-65	Fib3	HIPK2	FALSE	FALSE
1.95E-69	0.716565921	0.486	0.044	4.92E-65	Fib3	S100A3	FALSE	FALSE
2.20E-67	0.40169718	0.394	0.024	5.55E-63	Fib3	DGKI	FALSE	FALSE
6.84E-67	0.463987537	0.563	0.067	1.73E-62	Fib3	ACOT7	FALSE	FALSE
2.01E-66	1.182308718	0.979	0.274	5.08E-62	Fib3	PRSS23	FALSE	FALSE
1.02E-65	1.178197531	0.739	0.128	2.57E-61	Fib3	TNFRSF11B	FALSE	FALSE
1.06E-65	0.626542088	0.592	0.078	2.68E-61	Fib3	CAPG	FALSE	FALSE
1.44E-65	0.606059093	0.641	0.098	3.62E-61	Fib3	HMGCR	FALSE	FALSE
2.91E-64	0.439564668	0.507	0.054	7.34E-60	Fib3	GALE	FALSE	FALSE
6.13E-64	0.302445923	0.394	0.026	1.55E-59	Fib3	STX1A	FALSE	FALSE
2.68E-63	0.425092822	0.387	0.025	6.75E-59	Fib3	TRPV4	FALSE	TRUE
2.70E-63	0.581290932	0.634	0.093	6.80E-59	Fib3	CMIP	FALSE	FALSE
2.82E-63	0.93200303	0.746	0.146	7.12E-59	Fib3	CCND1	FALSE	FALSE
2.89E-63	0.350100157	0.415	0.031	7.29E-59	Fib3	TPD52	FALSE	FALSE
3.03E-63	0.668734428	0.817	0.18	7.64E-59	Fib3	GOLM1	FALSE	FALSE
3.12E-63	0.536134786	0.824	0.164	7.87E-59	Fib3	SLC9A3R2	FALSE	FALSE
3.49E-63	0.405472983	0.5	0.052	8.82E-59	Fib3	NGEF	FALSE	FALSE
8.59E-63	0.707649196	0.817	0.178	2.17E-58	Fib3	PAPSS2	FALSE	FALSE
1.46E-62	1.341003486	0.944	0.318	3.68E-58	Fib3	SLC39A14	FALSE	TRUE
1.98E-62	0.895817034	0.894	0.228	4.99E-58	Fib3	METRNL	FALSE	FALSE
3.40E-62	1.571941199	0.817	0.186	8.59E-58	Fib3	SERPINA1	FALSE	FALSE
5.29E-62	0.794959786	0.845	0.209	1.33E-57	Fib3	P4HA2	FALSE	FALSE
8.92E-62	0.644003508	0.754	0.144	2.25E-57	Fib3	CD82	FALSE	TRUE
3.91E-61	0.529905132	0.331	0.016	9.86E-57	Fib3	FERMT1	FALSE	FALSE
1.99E-60	0.793048494	0.761	0.162	5.03E-56	Fib3	GFPT2	FALSE	FALSE
2.41E-60	1.270743054	0.972	0.431	6.08E-56	Fib3	PMEPA1	FALSE	TRUE
2.48E-60	0.294277355	0.437	0.038	6.26E-56	Fib3	TNFAIP8L3	FALSE	FALSE
9.00E-60	0.31842755	0.43	0.037	2.27E-55	Fib3	KCNN4	FALSE	FALSE
1.68E-59	0.741087946	0.697	0.128	4.24E-55	Fib3	SCD	FALSE	FALSE
2.03E-59	0.362613941	0.43	0.04	5.13E-55	Fib3	WNT5B	FALSE	FALSE
2.38E-59	0.873550496	0.845	0.214	6.01E-55	Fib3	CD109	FALSE	TRUE
2.72E-59	0.357695513	0.451	0.045	6.87E-55	Fib3	PLA2G4A	FALSE	FALSE
2.79E-59	0.540907879	0.549	0.074	7.04E-55	Fib3	SCIN	FALSE	FALSE
4.01E-59	0.357024018	0.401	0.032	1.01E-54	Fib3	SDK2	FALSE	TRUE
4.63E-59	0.579550676	0.697	0.13	1.17E-54	Fib3	PXDN	FALSE	FALSE

5.98E-59	1.241460148	0.993	0.541	1.51E-54	Fib3	ENO1	TRUE	FALSE
1.23E-58	0.411893286	0.366	0.025	3.11E-54	Fib3	DHCR7	FALSE	FALSE
1.36E-58	1.361081123	0.704	0.15	3.43E-54	Fib3	LOX	FALSE	FALSE
1.81E-58	1.37122758	0.993	0.614	4.57E-54	Fib3	SH3BGRL3	FALSE	FALSE
2.96E-58	1.671382241	1	0.591	7.46E-54	Fib3	COL6A1	FALSE	FALSE
3.81E-58	0.364219695	0.563	0.079	9.61E-54	Fib3	BARX1	TRUE	FALSE
4.29E-58	0.55399842	0.585	0.085	1.08E-53	Fib3	CDON	FALSE	TRUE
1.04E-57	0.507798789	0.711	0.138	2.63E-53	Fib3	AK1	FALSE	FALSE
4.48E-57	1.660537047	0.986	0.453	1.13E-52	Fib3	SERPINE2	FALSE	FALSE
4.83E-57	1.389603705	0.993	0.467	1.22E-52	Fib3	COL6A3	FALSE	FALSE
5.84E-57	0.776628125	0.338	0.02	1.47E-52	Fib3	FABP3	FALSE	FALSE
9.51E-57	0.96564588	0.915	0.334	2.40E-52	Fib3	PGAM1	FALSE	FALSE
9.69E-57	0.34077794	0.465	0.05	2.45E-52	Fib3	PXYLP1	FALSE	FALSE
1.51E-56	0.657445658	0.662	0.125	3.82E-52	Fib3	STK38L	FALSE	FALSE
2.73E-56	0.432461991	0.479	0.057	6.88E-52	Fib3	FADS2	FALSE	FALSE
2.09E-55	0.359220434	0.331	0.02	5.27E-51	Fib3	C10orf90	FALSE	FALSE
2.17E-55	0.826898341	0.951	0.296	5.48E-51	Fib3	PLEC	FALSE	FALSE
4.29E-55	0.43029146	0.57	0.082	1.08E-50	Fib3	SLC38A1	FALSE	TRUE
4.93E-55	0.970332307	0.937	0.331	1.24E-50	Fib3	LRRFIP1	TRUE	FALSE
5.21E-55	1.552346885	1	0.647	1.31E-50	Fib3	COL6A2	FALSE	FALSE
8.02E-55	0.757342343	0.831	0.215	2.02E-50	Fib3	BNIP3	FALSE	FALSE
3.80E-54	0.589236955	0.754	0.172	9.58E-50	Fib3	PSD3	FALSE	FALSE
3.87E-54	0.497479079	0.81	0.196	9.76E-50	Fib3	TMCO3	FALSE	TRUE
4.24E-54	0.280523584	0.627	0.1	1.07E-49	Fib3	PERP	FALSE	FALSE
4.68E-54	0.508140386	0.676	0.134	1.18E-49	Fib3	MSI2	TRUE	FALSE
4.77E-54	0.375490234	0.528	0.075	1.20E-49	Fib3	PDGFC	FALSE	FALSE
8.83E-54	1.268953198	1	0.808	2.23E-49	Fib3	S100A10	FALSE	FALSE
1.48E-53	0.46305335	0.507	0.065	3.74E-49	Fib3	ITGA2	FALSE	TRUE
1.89E-53	0.784829644	0.803	0.201	4.76E-49	Fib3	NRP2	FALSE	TRUE
2.21E-53	0.946258417	0.908	0.303	5.58E-49	Fib3	GLS	FALSE	FALSE
2.37E-53	0.307771271	0.521	0.07	5.97E-49	Fib3	PXN	FALSE	FALSE
2.84E-53	0.425076423	0.732	0.151	7.17E-49	Fib3	CHST3	FALSE	FALSE
2.85E-53	1.150700632	0.937	0.393	7.18E-49	Fib3	PGK1	FALSE	FALSE
3.72E-53	1.105594416	0.986	0.519	9.39E-49	Fib3	PKM	TRUE	FALSE
3.97E-53	0.492687703	0.577	0.096	1.00E-48	Fib3	AK4	FALSE	FALSE
1.19E-52	0.380784629	0.5	0.065	3.00E-48	Fib3	ITPR3	FALSE	FALSE
1.30E-52	0.468939319	0.606	0.107	3.27E-48	Fib3	PGM1	FALSE	FALSE
1.68E-52	0.439013061	0.275	0.012	4.25E-48	Fib3	AL513283.1	FALSE	FALSE
1.96E-52	0.353626132	0.669	0.124	4.95E-48	Fib3	ASAP2	FALSE	FALSE
4.48E-52	0.490903198	0.81	0.189	1.13E-47	Fib3	ATP2B4	FALSE	TRUE
4.62E-52	1.131707876	1	0.802	1.17E-47	Fib3	GAPDH	FALSE	FALSE
6.98E-52	1.164541886	0.817	0.222	1.76E-47	Fib3	TNC	FALSE	FALSE
1.07E-51	1.101804394	0.986	0.617	2.69E-47	Fib3	LDHA	FALSE	FALSE
3.70E-51	0.54439292	0.57	0.099	9.33E-47	Fib3	PID1	FALSE	FALSE
4.23E-51	0.401668619	0.697	0.143	1.07E-46	Fib3	DIXDC1	FALSE	FALSE
4.54E-51	0.796432295	0.803	0.216	1.15E-46	Fib3	TGFBR1	FALSE	TRUE
5.58E-51	0.373868203	0.239	0.007	1.41E-46	Fib3	C19orf33	FALSE	FALSE
8.90E-51	0.402718156	0.57	0.095	2.25E-46	Fib3	PYGB	FALSE	FALSE
2.49E-50	0.319601462	0.556	0.089	6.29E-46	Fib3	TES	FALSE	FALSE
2.77E-50	0.264572496	0.338	0.025	7.00E-46	Fib3	ADGRG1	FALSE	FALSE
4.46E-50	0.540177185	0.718	0.161	1.13E-45	Fib3	HMGA1	TRUE	FALSE
4.56E-50	1.154445097	0.937	0.345	1.15E-45	Fib3	PCOLCE2	FALSE	FALSE
5.66E-50	0.405986362	0.451	0.059	1.43E-45	Fib3	PTGER2	FALSE	TRUE
7.47E-50	0.523506444	0.662	0.141	1.89E-45	Fib3	PTPN14	FALSE	FALSE
1.24E-49	0.342075166	0.401	0.043	3.14E-45	Fib3	DHCR24	FALSE	FALSE
1.39E-49	0.621603454	0.852	0.232	3.51E-45	Fib3	CLMP	FALSE	TRUE
4.08E-49	0.694256812	0.887	0.255	1.03E-44	Fib3	TM4SF1	FALSE	TRUE
5.38E-49	0.421390234	0.697	0.15	1.36E-44	Fib3	9-Sep	FALSE	FALSE
5.59E-49	0.464336917	0.655	0.136	1.41E-44	Fib3	PIEZO1	FALSE	TRUE
7.22E-49	0.357856994	0.676	0.141	1.82E-44	Fib3	GLTP	FALSE	FALSE
1.33E-48	0.415062982	0.535	0.085	3.35E-44	Fib3	AHNAK2	FALSE	FALSE
1.44E-48	0.592216244	0.824	0.238	3.64E-44	Fib3	CDK2AP1	TRUE	FALSE
1.49E-48	0.291625379	0.408	0.046	3.76E-44	Fib3	PPP1R14C	FALSE	FALSE
2.52E-48	0.54783385	0.718	0.174	6.35E-44	Fib3	AGTRAP	FALSE	FALSE
4.25E-48	0.955389366	0.986	0.441	1.07E-43	Fib3	P4HB	TRUE	FALSE
4.45E-48	0.38943085	0.556	0.098	1.12E-43	Fib3	PYGL	FALSE	FALSE
4.65E-48	0.860101091	0.775	0.206	1.17E-43	Fib3	SLC5A3	FALSE	TRUE
4.76E-48	0.575685214	0.711	0.159	1.20E-43	Fib3	FGF2	FALSE	FALSE
5.31E-48	0.460915932	0.655	0.137	1.34E-43	Fib3	TRIM47	FALSE	FALSE
6.20E-48	0.37090945	0.465	0.064	1.56E-43	Fib3	PC	FALSE	FALSE
8.52E-48	0.626253647	0.599	0.115	2.15E-43	Fib3	SLC29A1	FALSE	TRUE
9.93E-48	0.26756914	0.465	0.064	2.51E-43	Fib3	SREBF2	TRUE	FALSE
1.32E-47	1.589087633	1	0.902	3.33E-43	Fib3	MT2A	FALSE	FALSE
1.49E-47	1.106322918	0.655	0.156	3.77E-43	Fib3	MT1L	FALSE	FALSE
1.64E-47	0.389672608	0.5	0.074	4.13E-43	Fib3	SYNPO	FALSE	FALSE
5.64E-47	0.621333286	0.599	0.123	1.42E-42	Fib3	SQLE	FALSE	FALSE
2.34E-46	0.894275444	0.951	0.392	5.90E-42	Fib3	VKORC1	FALSE	FALSE
1.50E-45	0.887368351	0.944	0.393	3.79E-41	Fib3	FXYD5	FALSE	FALSE
1.84E-45	0.450989019	0.746	0.188	4.65E-41	Fib3	SMS	FALSE	FALSE
2.03E-45	0.396198929	0.697	0.159	5.12E-41	Fib3	SHMT2	FALSE	FALSE
4.24E-45	1.366945643	0.859	0.327	1.07E-40	Fib3	ANGPTL4	FALSE	FALSE
4.97E-45	0.944183003	0.88	0.306	1.25E-40	Fib3	UGP2	TRUE	FALSE
6.17E-45	0.413584897	0.718	0.176	1.56E-40	Fib3	SLC39A6	FALSE	TRUE
9.40E-45	0.281846898	0.5	0.08	2.37E-40	Fib3	RASA1	FALSE	FALSE
1.11E-44	0.620248259	0.613	0.128	2.80E-40	Fib3	ADAMTS6	FALSE	FALSE
2.59E-44	0.791871398	0.937	0.372	6.52E-40	Fib3	PLP2	FALSE	FALSE

2.79E-44	0.798550757	0.789	0.232	7.05E-40	Fib3	IVNS1ABP	FALSE	FALSE
5.66E-44	0.27899592	0.528	0.087	1.43E-39	Fib3	FBXO2	FALSE	FALSE
7.80E-44	0.815627518	0.923	0.342	1.97E-39	Fib3	VEGFA	FALSE	FALSE
1.23E-43	0.65086926	0.88	0.297	3.10E-39	Fib3	P4HA1	FALSE	FALSE
1.35E-43	0.571668723	0.831	0.246	3.40E-39	Fib3	FAM129B	FALSE	FALSE
1.39E-43	0.365664359	0.662	0.143	3.51E-39	Fib3	SORBS2	FALSE	FALSE
1.42E-43	0.313252187	0.606	0.124	3.58E-39	Fib3	MPP6	FALSE	FALSE
1.61E-43	0.658597667	0.761	0.201	4.08E-39	Fib3	SOX9	TRUE	FALSE
2.09E-43	0.515637658	0.901	0.305	5.27E-39	Fib3	ACTN4	FALSE	FALSE
2.48E-43	0.297871577	0.444	0.067	6.27E-39	Fib3	ACAT2	FALSE	FALSE
3.78E-43	1.00260678	0.986	0.646	9.55E-39	Fib3	PRDX1	FALSE	FALSE
4.40E-43	0.286886251	0.486	0.08	1.11E-38	Fib3	NOMO1	FALSE	FALSE
4.42E-43	0.846940877	0.824	0.283	1.11E-38	Fib3	UGDH	FALSE	FALSE
4.55E-43	0.3338462	0.493	0.082	1.15E-38	Fib3	KIAA0040	FALSE	FALSE
4.56E-43	0.724047543	0.563	0.111	1.15E-38	Fib3	BMP2	FALSE	FALSE
2.26E-42	0.709366902	0.852	0.283	5.69E-38	Fib3	EDIL3	FALSE	FALSE
2.38E-42	0.450512006	0.725	0.187	6.00E-38	Fib3	MAGED1	FALSE	FALSE
3.33E-42	0.590492167	0.852	0.287	8.41E-38	Fib3	FAM114A1	FALSE	FALSE
3.75E-42	0.321201737	0.366	0.044	9.46E-38	Fib3	FUT4	FALSE	FALSE
4.65E-42	0.439341561	0.697	0.176	1.17E-37	Fib3	GARS	FALSE	FALSE
4.96E-42	0.331888681	0.493	0.088	1.25E-37	Fib3	PARVB	FALSE	FALSE
6.25E-42	0.507932219	0.824	0.227	1.58E-37	Fib3	MIR100HG	FALSE	FALSE
6.63E-42	0.397439193	0.542	0.108	1.67E-37	Fib3	BPGM	FALSE	FALSE
8.97E-42	0.369889588	0.585	0.119	2.26E-37	Fib3	PAX1	TRUE	FALSE
1.49E-41	0.356489731	0.423	0.063	3.76E-37	Fib3	SC5D	FALSE	FALSE
2.72E-41	0.436773087	0.662	0.163	6.87E-37	Fib3	SH3KBP1	FALSE	FALSE
2.78E-41	1.005461255	1	0.959	7.00E-37	Fib3	S100A6	FALSE	FALSE
3.15E-41	0.320118526	0.57	0.115	7.95E-37	Fib3	SKAP2	FALSE	FALSE
3.77E-41	0.299933794	0.697	0.173	9.50E-37	Fib3	MARVELD1	FALSE	FALSE
5.43E-41	0.423347301	0.303	0.028	1.37E-36	Fib3	ADGRG2	FALSE	FALSE
6.39E-41	0.828959114	0.894	0.348	1.61E-36	Fib3	TNFRSF12A	FALSE	FALSE
8.04E-41	0.399204478	0.585	0.128	2.03E-36	Fib3	SACS	FALSE	FALSE
8.27E-41	0.427874529	0.613	0.137	2.09E-36	Fib3	FZD8	FALSE	TRUE
1.07E-40	0.558748989	0.606	0.141	2.71E-36	Fib3	INSIG1	FALSE	FALSE
1.15E-40	0.394668341	0.662	0.167	2.90E-36	Fib3	FSCN1	FALSE	FALSE
1.22E-40	0.497802596	0.754	0.215	3.09E-36	Fib3	GFPT1	FALSE	FALSE
1.34E-40	0.278034279	0.521	0.101	3.39E-36	Fib3	YIF1B	FALSE	FALSE
2.54E-40	0.329049537	0.423	0.066	6.42E-36	Fib3	MINPP1	FALSE	FALSE
2.55E-40	0.649243963	0.915	0.321	6.44E-36	Fib3	PLAUR	FALSE	TRUE
2.60E-40	0.578992193	0.676	0.179	6.55E-36	Fib3	SLC26A2	FALSE	TRUE
2.84E-40	0.607023775	0.754	0.228	7.16E-36	Fib3	TMEM45A	FALSE	FALSE
2.95E-40	0.932614309	0.972	0.539	7.46E-36	Fib3	EMP3	FALSE	TRUE
3.85E-40	0.331977312	0.796	0.233	9.70E-36	Fib3	GPI	FALSE	FALSE
4.35E-40	0.931183506	0.944	0.492	1.10E-35	Fib3	TPI1	TRUE	FALSE
4.61E-40	0.400703457	0.296	0.027	1.16E-35	Fib3	TSPAN2	FALSE	TRUE
5.29E-40	1.294556933	0.5	0.098	1.33E-35	Fib3	G0S2	FALSE	FALSE
6.34E-40	0.506741235	0.408	0.061	1.60E-35	Fib3	TREM1	FALSE	TRUE
7.11E-40	0.55734843	0.775	0.236	1.79E-35	Fib3	MRPS6	FALSE	FALSE
9.33E-40	0.491540287	0.613	0.146	2.36E-35	Fib3	ABHD2	FALSE	FALSE
1.12E-39	0.889861993	0.866	0.314	2.83E-35	Fib3	FBN1	FALSE	FALSE
1.26E-39	0.897841427	1	0.611	3.17E-35	Fib3	ITGB1	FALSE	TRUE
1.49E-39	0.340033292	0.634	0.146	3.76E-35	Fib3	DLG1	FALSE	FALSE
3.57E-39	0.344304807	0.725	0.196	9.01E-35	Fib3	GALNT2	FALSE	FALSE
4.48E-39	0.311903598	0.577	0.129	1.13E-34	Fib3	FHL2	TRUE	FALSE
6.95E-39	0.308020801	0.5	0.094	1.75E-34	Fib3	MTHFD1L	FALSE	FALSE
8.28E-39	0.897392059	0.986	0.539	2.09E-34	Fib3	CD59	TRUE	TRUE
1.32E-38	0.543764908	0.683	0.175	3.33E-34	Fib3	FXYD6	FALSE	FALSE
1.63E-38	0.305085051	0.444	0.073	4.10E-34	Fib3	SLC35E4	FALSE	FALSE
1.95E-38	0.303090716	0.585	0.131	4.92E-34	Fib3	SLC25A39	FALSE	FALSE
4.89E-38	0.415628345	0.683	0.181	1.23E-33	Fib3	ZEB1	TRUE	FALSE
5.43E-38	0.344590796	0.69	0.183	1.37E-33	Fib3	ABI1	FALSE	FALSE
7.48E-38	0.417098904	0.662	0.171	1.89E-33	Fib3	DSE	FALSE	FALSE
1.14E-37	0.63880245	0.803	0.257	2.87E-33	Fib3	FAP	FALSE	TRUE
1.77E-37	0.307554658	0.606	0.146	4.48E-33	Fib3	ORMDL2	FALSE	FALSE
2.82E-37	0.439175117	0.697	0.19	7.11E-33	Fib3	RAPH1	FALSE	FALSE
3.03E-37	0.745340051	0.937	0.399	7.66E-33	Fib3	LMAN1	FALSE	FALSE
3.05E-37	0.820529808	0.683	0.193	7.69E-33	Fib3	ENPP1	FALSE	TRUE
3.12E-37	0.607697565	0.634	0.168	7.87E-33	Fib3	NREP	FALSE	FALSE
3.47E-37	0.582025975	0.93	0.354	8.75E-33	Fib3	FNDC3B	FALSE	FALSE
4.14E-37	0.471425011	0.725	0.213	1.04E-32	Fib3	EIF4EBP1	FALSE	FALSE
5.76E-37	0.251174518	0.486	0.092	1.45E-32	Fib3	KLHL21	FALSE	FALSE
6.83E-37	0.271214253	0.585	0.132	1.72E-32	Fib3	PRKCA	FALSE	FALSE
8.87E-37	0.461951279	0.761	0.234	2.24E-32	Fib3	ERGIC1	FALSE	FALSE
1.62E-36	0.4902868	0.725	0.23	4.09E-32	Fib3	CAPN2	FALSE	FALSE
1.78E-36	0.262428809	0.528	0.113	4.49E-32	Fib3	RMDN1	FALSE	FALSE
2.00E-36	0.443755599	0.69	0.181	5.05E-32	Fib3	ACKR3	FALSE	TRUE
2.06E-36	0.377511006	0.486	0.099	5.19E-32	Fib3	ITGBL1	FALSE	FALSE
2.42E-36	0.405844954	0.873	0.282	6.10E-32	Fib3	UAP1	FALSE	FALSE
2.63E-36	0.533348742	0.775	0.256	6.64E-32	Fib3	PDLIM7	FALSE	FALSE
5.57E-36	0.333699781	0.676	0.184	1.41E-31	Fib3	SMARCA2	FALSE	FALSE
8.11E-36	0.320593389	0.444	0.079	2.05E-31	Fib3	ZNF385A	TRUE	FALSE
8.87E-36	0.250669396	0.303	0.034	2.24E-31	Fib3	B3GNT5	FALSE	FALSE
1.07E-35	0.262460654	0.507	0.108	2.70E-31	Fib3	SLC25A1	FALSE	FALSE
1.14E-35	0.323389774	0.535	0.116	2.88E-31	Fib3	GCLM	FALSE	FALSE
1.29E-35	0.361037202	0.648	0.171	3.25E-31	Fib3	SLC1A5	FALSE	TRUE
2.22E-35	0.28444502	0.57	0.133	5.61E-31	Fib3	HK1	FALSE	FALSE

2.27E-35	0.636969132	0.93	0.379	5.72E-31	Fib3	TMED9	FALSE	FALSE
2.31E-35	0.613134877	0.831	0.278	5.84E-31	Fib3	COL5A1	FALSE	FALSE
2.57E-35	0.697673723	0.958	0.446	6.50E-31	Fib3	CALU	FALSE	FALSE
2.58E-35	0.362746623	0.754	0.237	6.52E-31	Fib3	DARS	FALSE	FALSE
3.52E-35	0.431759564	0.289	0.031	8.89E-31	Fib3	CYP26B1	FALSE	FALSE
5.02E-35	0.613712135	0.838	0.305	1.27E-30	Fib3	RCN3	FALSE	FALSE
6.97E-35	0.716148278	0.873	0.364	1.76E-30	Fib3	MYDGF	FALSE	FALSE
7.00E-35	0.576122162	0.838	0.269	1.77E-30	Fib3	RBP4	FALSE	FALSE
7.30E-35	0.257383997	0.514	0.109	1.84E-30	Fib3	TRIQK	FALSE	FALSE
7.46E-35	0.329406065	0.746	0.227	1.88E-30	Fib3	C5orf15	FALSE	FALSE
7.50E-35	0.25386836	0.697	0.192	1.89E-30	Fib3	CLSTN1	FALSE	TRUE
9.34E-35	0.382196102	0.796	0.269	2.36E-30	Fib3	RRAS	FALSE	FALSE
1.20E-34	0.602545548	0.739	0.235	3.03E-30	Fib3	JAG1	FALSE	TRUE
1.39E-34	0.535891485	0.317	0.041	3.50E-30	Fib3	CLIC3	FALSE	FALSE
1.52E-34	0.632064435	0.444	0.082	3.82E-30	Fib3	TFPI2	FALSE	FALSE
1.77E-34	0.323420826	0.704	0.198	4.47E-30	Fib3	EHD2	FALSE	FALSE
2.70E-34	0.579769376	0.866	0.336	6.80E-30	Fib3	TRPS1	TRUE	FALSE
2.72E-34	0.726288025	0.986	0.67	6.87E-30	Fib3	ANXA2	FALSE	FALSE
2.82E-34	0.558320291	0.803	0.276	7.11E-30	Fib3	GALNT1	FALSE	FALSE
3.05E-34	0.723758834	0.908	0.368	7.69E-30	Fib3	COL5A2	FALSE	FALSE
4.13E-34	0.284993309	0.359	0.054	1.04E-29	Fib3	MEG8	FALSE	FALSE
4.89E-34	0.439561104	0.789	0.273	1.23E-29	Fib3	NPTN	FALSE	TRUE
6.04E-34	0.330179032	0.732	0.22	1.52E-29	Fib3	IMPAD1	FALSE	FALSE
8.01E-34	0.254301228	0.359	0.055	2.02E-29	Fib3	ARHGAP22	FALSE	FALSE
8.12E-34	0.271315255	0.592	0.138	2.05E-29	Fib3	SEMA3C	FALSE	FALSE
1.31E-33	0.63182515	0.613	0.172	3.30E-29	Fib3	HMGCS1	FALSE	FALSE
2.49E-33	0.744978572	1	0.632	6.27E-29	Fib3	HSP90B1	FALSE	FALSE
3.46E-33	0.344669778	0.739	0.233	8.74E-29	Fib3	BIRC2	FALSE	FALSE
4.44E-33	0.263256214	0.62	0.155	1.12E-28	Fib3	COL11A1	FALSE	FALSE
5.69E-33	0.274284068	0.725	0.221	1.43E-28	Fib3	SREK1IP1	FALSE	FALSE
6.16E-33	0.942356562	1	0.82	1.55E-28	Fib3	LGALS1	FALSE	FALSE
6.83E-33	0.327829281	0.683	0.202	1.72E-28	Fib3	TMED3	FALSE	FALSE
7.71E-33	0.440455483	0.824	0.273	1.95E-28	Fib3	PLXDC2	FALSE	TRUE
7.93E-33	0.620932708	0.93	0.42	2.00E-28	Fib3	CSTB	FALSE	FALSE
8.27E-33	0.884371541	0.972	0.598	2.09E-28	Fib3	ANXA1	TRUE	FALSE
8.31E-33	0.390799301	0.88	0.338	2.10E-28	Fib3	CAPNS1	FALSE	FALSE
8.49E-33	0.357337748	0.817	0.27	2.14E-28	Fib3	CBX5	FALSE	FALSE
1.02E-32	0.710750917	0.993	0.754	2.56E-28	Fib3	S100A11	FALSE	FALSE
1.06E-32	0.484963128	0.796	0.269	2.67E-28	Fib3	ITGB5	FALSE	TRUE
1.57E-32	0.384119036	0.563	0.141	3.96E-28	Fib3	C1GALT1	FALSE	FALSE
2.47E-32	0.626006789	0.965	0.478	6.23E-28	Fib3	YWHAZ	TRUE	FALSE
2.53E-32	0.368947456	0.303	0.039	6.39E-28	Fib3	CA9	FALSE	FALSE
2.84E-32	0.338265327	0.282	0.033	7.17E-28	Fib3	ANKRD6	FALSE	FALSE
4.98E-32	0.545321413	0.81	0.291	1.26E-27	Fib3	SLC25A37	FALSE	FALSE
5.38E-32	0.312416228	0.739	0.24	1.36E-27	Fib3	TKT	FALSE	FALSE
6.58E-32	0.318565	0.289	0.036	1.66E-27	Fib3	BMP6	FALSE	FALSE
6.91E-32	0.498582822	0.866	0.356	1.74E-27	Fib3	VDAC1	FALSE	FALSE
6.99E-32	0.362661638	0.648	0.18	1.76E-27	Fib3	EZR	TRUE	FALSE
7.12E-32	0.642319513	0.937	0.391	1.80E-27	Fib3	FSTL1	FALSE	FALSE
7.54E-32	0.265153293	0.528	0.128	1.90E-27	Fib3	FDFT1	FALSE	FALSE
9.06E-32	0.289964555	0.592	0.16	2.29E-27	Fib3	COPG1	FALSE	FALSE
1.09E-31	0.430598658	0.873	0.331	2.75E-27	Fib3	ASPH	FALSE	FALSE
1.13E-31	0.321851927	0.648	0.19	2.85E-27	Fib3	LARP6	FALSE	FALSE
1.21E-31	0.259457787	0.394	0.071	3.05E-27	Fib3	SOWAHC	FALSE	FALSE
1.24E-31	0.689288069	0.782	0.286	3.12E-27	Fib3	LTBP1	FALSE	FALSE
1.40E-31	0.379458731	0.648	0.188	3.54E-27	Fib3	ANKLE2	FALSE	FALSE
1.82E-31	0.414317485	0.549	0.142	4.59E-27	Fib3	SLC16A1	FALSE	TRUE
2.01E-31	0.625714495	0.824	0.324	5.06E-27	Fib3	CKAP4	FALSE	FALSE
2.71E-31	0.328031433	0.634	0.186	6.84E-27	Fib3	SMARCA1	TRUE	FALSE
3.68E-31	0.384877666	0.775	0.266	9.29E-27	Fib3	ATL3	FALSE	FALSE
3.77E-31	0.27950135	0.739	0.239	9.50E-27	Fib3	ERGIC3	FALSE	FALSE
4.55E-31	0.265951114	0.746	0.241	1.15E-26	Fib3	JOSD2	FALSE	FALSE
5.49E-31	0.392052911	0.894	0.347	1.39E-26	Fib3	GNB1	FALSE	FALSE
7.84E-31	0.269155923	0.577	0.15	1.98E-26	Fib3	NT5DC2	FALSE	FALSE
8.06E-31	0.256746262	0.662	0.197	2.03E-26	Fib3	RPA3	FALSE	FALSE
1.13E-30	0.357205555	0.563	0.142	2.84E-26	Fib3	ANGPTL5	FALSE	FALSE
1.52E-30	0.323001339	0.754	0.248	3.84E-26	Fib3	RNF145	FALSE	FALSE
1.71E-30	0.455014376	0.88	0.357	4.32E-26	Fib3	CIB1	FALSE	FALSE
3.02E-30	0.406501903	0.775	0.269	7.63E-26	Fib3	PDLIM5	TRUE	FALSE
4.16E-30	0.268426001	0.592	0.163	1.05E-25	Fib3	CETN2	FALSE	FALSE
4.44E-30	0.455712489	0.739	0.264	1.12E-25	Fib3	PLIN3	FALSE	FALSE
4.83E-30	0.32526156	0.606	0.172	1.22E-25	Fib3	IDI1	FALSE	FALSE
5.47E-30	0.285660299	0.704	0.209	1.38E-25	Fib3	ECM1	FALSE	FALSE
6.27E-30	0.264947034	0.62	0.18	1.58E-25	Fib3	TP53I13	FALSE	TRUE
6.37E-30	0.604093452	0.972	0.507	1.61E-25	Fib3	RTN4	FALSE	FALSE
9.80E-30	0.28002784	0.831	0.307	2.47E-25	Fib3	FAM177A1	FALSE	FALSE
1.27E-29	0.463599111	0.894	0.384	3.20E-25	Fib3	IQGAP1	FALSE	FALSE
1.60E-29	0.963090801	0.373	0.072	4.05E-25	Fib3	PTGS2	FALSE	FALSE
3.03E-29	0.256870526	0.408	0.085	7.66E-25	Fib3	FASN	FALSE	FALSE
3.08E-29	0.313349686	0.493	0.113	7.77E-25	Fib3	OGFRL1	FALSE	FALSE
3.29E-29	0.298446851	0.5	0.124	8.31E-25	Fib3	PIGK	FALSE	FALSE
3.84E-29	0.254757155	0.423	0.089	9.69E-25	Fib3	SPTLC2	FALSE	FALSE
4.16E-29	0.366465968	0.852	0.333	1.05E-24	Fib3	VDAC2	FALSE	FALSE
5.01E-29	0.397512192	0.556	0.152	1.26E-24	Fib3	P3H4	FALSE	FALSE
6.09E-29	0.303254049	0.761	0.268	1.54E-24	Fib3	FAM120A	FALSE	FALSE
6.49E-29	0.802555092	0.81	0.337	1.64E-24	Fib3	AL078639.1	FALSE	FALSE

7.81E-29	0.250466537	0.261	0.033	1.97E-24	Fib3	HMGA2	TRUE	FALSE
7.99E-29	0.369621398	0.852	0.32	2.02E-24	Fib3	CLTC	FALSE	FALSE
8.80E-29	0.413056305	0.803	0.288	2.22E-24	Fib3	FKBP10	FALSE	FALSE
8.93E-29	0.355087053	0.838	0.32	2.25E-24	Fib3	RAB1A	FALSE	FALSE
9.09E-29	0.466372231	0.739	0.254	2.29E-24	Fib3	MYADM	FALSE	TRUE
1.44E-28	0.395015774	0.775	0.285	3.62E-24	Fib3	PTPN12	FALSE	FALSE
1.48E-28	0.267517214	0.725	0.24	3.72E-24	Fib3	TMX1	FALSE	FALSE
1.50E-28	0.308312428	0.824	0.307	3.78E-24	Fib3	PDLIM4	FALSE	FALSE
1.77E-28	0.34392012	0.486	0.12	4.47E-24	Fib3	MYO10	FALSE	FALSE
2.26E-28	0.315867909	0.761	0.267	5.71E-24	Fib3	ARF6	FALSE	FALSE
2.44E-28	0.260024375	0.683	0.213	6.16E-24	Fib3	STK24	FALSE	FALSE
2.67E-28	0.267625322	0.69	0.223	6.75E-24	Fib3	SEC23A	FALSE	FALSE
2.76E-28	0.435288678	0.93	0.42	6.97E-24	Fib3	YWHAQ	FALSE	FALSE
2.95E-28	0.258236212	0.535	0.139	7.45E-24	Fib3	TMTC3	FALSE	FALSE
3.25E-28	0.340216839	0.69	0.234	8.19E-24	Fib3	FAM162A	FALSE	FALSE
3.71E-28	0.270537368	0.697	0.22	9.35E-24	Fib3	TMX4	FALSE	TRUE
3.80E-28	0.278670335	0.69	0.224	9.58E-24	Fib3	GLO1	FALSE	FALSE
3.82E-28	0.553147306	0.951	0.458	9.64E-24	Fib3	PDIA6	FALSE	FALSE
3.95E-28	0.31493076	0.732	0.247	9.97E-24	Fib3	STT3B	FALSE	TRUE
4.15E-28	0.285178517	0.542	0.148	1.05E-23	Fib3	VASP	FALSE	FALSE
5.45E-28	0.421145193	0.951	0.402	1.38E-23	Fib3	FLNA	FALSE	FALSE
7.24E-28	1.226933864	0.704	0.281	1.83E-23	Fib3	ISLR	FALSE	FALSE
7.83E-28	0.404100046	0.845	0.339	1.98E-23	Fib3	ATP1A1	FALSE	TRUE
8.10E-28	0.341710974	0.585	0.174	2.04E-23	Fib3	ITGA10	FALSE	TRUE
1.39E-27	0.305280617	0.528	0.141	3.52E-23	Fib3	TNS3	FALSE	FALSE
1.40E-27	0.41435389	0.824	0.341	3.53E-23	Fib3	FERMT2	FALSE	FALSE
1.52E-27	1.204588469	0.965	0.611	3.82E-23	Fib3	MT1E	FALSE	FALSE
1.57E-27	0.667504531	0.972	0.525	3.97E-23	Fib3	AHNAK	FALSE	FALSE
1.92E-27	0.316388675	0.563	0.165	4.84E-23	Fib3	SSBP3	TRUE	FALSE
3.41E-27	0.675604103	0.965	0.548	8.61E-23	Fib3	SEC61G	FALSE	FALSE
3.84E-27	0.28196199	0.796	0.28	9.70E-23	Fib3	SEC61A1	FALSE	FALSE
4.44E-27	0.274321574	0.711	0.233	1.12E-22	Fib3	NAV1	FALSE	FALSE
5.38E-27	0.370362284	0.915	0.356	1.36E-22	Fib3	TTC3	FALSE	FALSE
6.58E-27	0.392048829	0.739	0.272	1.66E-22	Fib3	HCFC1R1	FALSE	FALSE
6.66E-27	0.31075718	0.768	0.272	1.68E-22	Fib3	COPB1	FALSE	FALSE
7.00E-27	0.306791314	0.697	0.24	1.77E-22	Fib3	IKBIP	FALSE	FALSE
8.08E-27	0.443680844	0.88	0.376	2.04E-22	Fib3	MSN	FALSE	FALSE
8.14E-27	0.263877014	0.31	0.051	2.05E-22	Fib3	COL7A1	FALSE	FALSE
9.73E-27	0.364160177	0.838	0.34	2.46E-22	Fib3	GNB2	FALSE	FALSE
1.09E-26	0.293124524	0.5	0.133	2.74E-22	Fib3	KLHL42	FALSE	FALSE
1.13E-26	0.295832404	0.634	0.203	2.85E-22	Fib3	FDPS	FALSE	FALSE
1.21E-26	0.324617214	0.873	0.365	3.06E-22	Fib3	CYB5R3	FALSE	FALSE
1.34E-26	0.583499085	0.979	0.511	3.39E-22	Fib3	PDIA3	FALSE	FALSE
1.54E-26	0.712541694	0.993	0.628	3.88E-22	Fib3	NDUFA4L2	FALSE	FALSE
1.54E-26	0.3791128	0.887	0.361	3.88E-22	Fib3	RPN2	FALSE	FALSE
1.68E-26	0.407575569	0.838	0.33	4.24E-22	Fib3	PDIA4	FALSE	FALSE
1.99E-26	0.286940884	0.669	0.22	5.03E-22	Fib3	SEL1L	FALSE	FALSE
2.32E-26	0.298774939	0.732	0.246	5.85E-22	Fib3	VASN	FALSE	TRUE
3.63E-26	0.554232957	0.958	0.467	9.16E-22	Fib3	BZW1	FALSE	FALSE
5.17E-26	0.477733398	0.782	0.291	1.31E-21	Fib3	CD55	FALSE	TRUE
6.12E-26	0.558951731	0.93	0.462	1.54E-21	Fib3	TSPO	FALSE	FALSE
6.32E-26	0.326100538	0.451	0.111	1.60E-21	Fib3	CPD	FALSE	TRUE
6.55E-26	0.347771321	0.923	0.401	1.65E-21	Fib3	GNAI2	FALSE	FALSE
1.32E-25	0.500428581	0.958	0.443	3.33E-21	Fib3	CD44	FALSE	TRUE
1.33E-25	0.504572866	0.676	0.223	3.35E-21	Fib3	MEG3	FALSE	FALSE
1.52E-25	0.306756407	0.634	0.203	3.83E-21	Fib3	ELL2	FALSE	FALSE
1.57E-25	0.501579073	0.923	0.426	3.97E-21	Fib3	RRBP1	FALSE	FALSE
3.14E-25	0.666525815	0.972	0.541	7.93E-21	Fib3	MIF	FALSE	FALSE
3.35E-25	0.250945159	0.676	0.221	8.45E-21	Fib3	TUBB2A	FALSE	FALSE
3.86E-25	0.46158114	0.972	0.476	9.73E-21	Fib3	CANX	TRUE	FALSE
4.00E-25	0.44362321	0.88	0.392	1.01E-20	Fib3	TRAM1	FALSE	FALSE
5.45E-25	0.392859467	0.923	0.408	1.38E-20	Fib3	TPM3	FALSE	FALSE
6.84E-25	0.42339136	0.831	0.354	1.73E-20	Fib3	STMP1	FALSE	FALSE
8.17E-25	0.383728037	0.887	0.385	2.06E-20	Fib3	PTTG1IP	FALSE	TRUE
8.53E-25	1.010791913	0.697	0.285	2.15E-20	Fib3	TGFBI	FALSE	FALSE
8.53E-25	0.38578964	0.789	0.31	2.15E-20	Fib3	SMIM14	FALSE	FALSE
9.95E-25	0.31986358	0.852	0.349	2.51E-20	Fib3	HDLBP	FALSE	FALSE
1.03E-24	0.276066247	0.761	0.285	2.61E-20	Fib3	ATP1B3	FALSE	TRUE
1.73E-24	0.299005068	0.768	0.297	4.37E-20	Fib3	UBE2K	TRUE	FALSE
1.96E-24	0.27413907	0.817	0.312	4.95E-20	Fib3	DNAJC3	FALSE	FALSE
2.18E-24	0.429236206	0.838	0.357	5.51E-20	Fib3	ACTN1	FALSE	FALSE
2.25E-24	0.320994184	0.725	0.257	5.68E-20	Fib3	SLC20A1	FALSE	FALSE
2.41E-24	0.257437843	0.704	0.257	6.08E-20	Fib3	NCKAP1	FALSE	FALSE
2.58E-24	0.452082806	0.951	0.467	6.51E-20	Fib3	ARL6IP5	FALSE	FALSE
2.80E-24	0.287294595	0.782	0.305	7.05E-20	Fib3	MLEC	FALSE	FALSE
2.92E-24	0.472414972	0.648	0.222	7.36E-20	Fib3	PHLDA2	TRUE	FALSE
2.96E-24	0.30657969	0.613	0.207	7.47E-20	Fib3	TWSG1	FALSE	FALSE
3.19E-24	1.083690938	0.577	0.201	8.04E-20	Fib3	INHBA	FALSE	FALSE
3.79E-24	0.324911362	0.831	0.341	9.57E-20	Fib3	ARPC1B	FALSE	FALSE
4.10E-24	0.25883106	0.852	0.343	1.03E-19	Fib3	MAP4	FALSE	FALSE
4.70E-24	0.313060672	0.782	0.298	1.18E-19	Fib3	ACADVL	FALSE	FALSE
5.34E-24	0.393442719	0.754	0.287	1.35E-19	Fib3	S100A16	FALSE	FALSE
5.49E-24	0.26448775	0.415	0.099	1.38E-19	Fib3	PDE4B	FALSE	FALSE
8.21E-24	0.27595919	0.782	0.307	2.07E-19	Fib3	RNH1	FALSE	FALSE
9.08E-24	0.29238699	0.81	0.304	2.29E-19	Fib3	LTBP3	FALSE	FALSE
1.07E-23	0.416508395	0.944	0.462	2.69E-19	Fib3	CD151	FALSE	TRUE

1.43E-23	0.279226961	0.831	0.331	3.60E-19	Fib3	LIMS1	FALSE	FALSE
1.59E-23	0.312564403	0.824	0.327	4.01E-19	Fib3	SSR1	FALSE	TRUE
2.40E-23	0.265983107	0.662	0.241	6.05E-19	Fib3	TMX3	FALSE	TRUE
2.48E-23	0.33625231	0.789	0.306	6.26E-19	Fib3	MANF	FALSE	FALSE
2.91E-23	0.279475876	0.69	0.247	7.33E-19	Fib3	MEF2A	TRUE	FALSE
3.78E-23	0.250155794	0.782	0.312	9.53E-19	Fib3	RCN2	FALSE	FALSE
4.76E-23	0.282582161	0.676	0.246	1.20E-18	Fib3	WDR1	FALSE	FALSE
4.83E-23	0.269449769	0.768	0.298	1.22E-18	Fib3	HM13	FALSE	TRUE
5.06E-23	0.262867522	0.662	0.233	1.28E-18	Fib3	PTBP3	FALSE	FALSE
5.79E-23	0.456381065	0.782	0.31	1.46E-18	Fib3	MFGE8	FALSE	FALSE
6.13E-23	1.220141984	0.915	0.622	1.55E-18	Fib3	S100A4	FALSE	FALSE
6.54E-23	0.346576705	0.57	0.184	1.65E-18	Fib3	SNAI2	TRUE	FALSE
6.85E-23	0.280785966	0.831	0.333	1.73E-18	Fib3	C6orf62	FALSE	FALSE
1.01E-22	0.255991494	0.866	0.371	2.55E-18	Fib3	PPP3CA	FALSE	FALSE
1.03E-22	0.314062562	0.676	0.241	2.60E-18	Fib3	FRMD6	FALSE	FALSE
1.29E-22	0.584403694	1	0.783	3.27E-18	Fib3	ACTG1	FALSE	FALSE
1.56E-22	0.379259332	0.338	0.075	3.94E-18	Fib3	CA12	FALSE	TRUE
1.59E-22	0.292770272	0.754	0.304	4.00E-18	Fib3	LMAN2	FALSE	TRUE
1.63E-22	0.285051467	0.754	0.281	4.12E-18	Fib3	RCN1	FALSE	FALSE
1.70E-22	0.377837287	0.655	0.239	4.29E-18	Fib3	C4orf48	FALSE	FALSE
1.79E-22	0.513011181	1	0.963	4.51E-18	Fib3	VIM	FALSE	FALSE
1.96E-22	0.293019568	0.739	0.293	4.94E-18	Fib3	TXNDC17	FALSE	FALSE
2.11E-22	0.33560713	0.606	0.212	5.32E-18	Fib3	PSMD2	FALSE	FALSE
2.35E-22	0.288795901	0.873	0.379	5.93E-18	Fib3	MORF4L2	FALSE	FALSE
2.57E-22	0.436741091	0.937	0.434	6.48E-18	Fib3	KDELR2	FALSE	FALSE
2.70E-22	0.339537583	0.775	0.322	6.82E-18	Fib3	BHLHE40	TRUE	FALSE
3.03E-22	0.351663979	0.38	0.094	7.64E-18	Fib3	SPOCK1	FALSE	FALSE
4.88E-22	0.623963849	0.965	0.565	1.23E-17	Fib3	ANXA5	FALSE	FALSE
8.17E-22	0.270708429	0.423	0.115	2.06E-17	Fib3	ADAMTS2	FALSE	FALSE
8.52E-22	1.371647318	0.923	0.688	2.15E-17	Fib3	FN1	FALSE	FALSE
1.11E-21	1.205911146	0.556	0.211	2.81E-17	Fib3	IGFBP3	FALSE	FALSE
1.18E-21	0.258438893	0.711	0.288	2.97E-17	Fib3	DGUOK	FALSE	FALSE
1.24E-21	0.283887319	0.676	0.242	3.13E-17	Fib3	SGK1	FALSE	FALSE
1.26E-21	0.434436336	0.81	0.363	3.19E-17	Fib3	SPCS3	FALSE	FALSE
1.32E-21	0.360231198	0.894	0.407	3.34E-17	Fib3	ARF4	FALSE	FALSE
1.52E-21	0.266327269	0.317	0.067	3.85E-17	Fib3	AGPAT4	FALSE	FALSE
1.53E-21	0.251407269	0.676	0.258	3.86E-17	Fib3	SFT2D1	TRUE	FALSE
1.63E-21	0.323858905	0.5	0.151	4.12E-17	Fib3	PTGES	FALSE	FALSE
1.84E-21	0.551509079	0.986	0.59	4.65E-17	Fib3	CALR	FALSE	FALSE
2.10E-21	0.363779462	0.887	0.418	5.30E-17	Fib3	HTRA1	FALSE	FALSE
2.43E-21	0.32862388	0.923	0.442	6.12E-17	Fib3	BRI3	FALSE	FALSE
2.74E-21	0.325280788	0.746	0.302	6.92E-17	Fib3	CAPZA1	FALSE	FALSE
3.64E-21	0.274958158	0.887	0.385	9.20E-17	Fib3	CBX3	FALSE	FALSE
4.32E-21	0.256915204	0.662	0.254	1.09E-16	Fib3	TMOD3	FALSE	FALSE
8.73E-21	0.281889607	0.556	0.185	2.20E-16	Fib3	FKBP14	FALSE	FALSE
9.88E-21	0.527720816	0.958	0.576	2.49E-16	Fib3	TXN	FALSE	FALSE
1.23E-20	0.537385227	0.993	0.678	3.11E-16	Fib3	PPIA	FALSE	FALSE
1.49E-20	0.360504172	0.683	0.283	3.77E-16	Fib3	PPIC	FALSE	FALSE
1.66E-20	0.261244061	0.754	0.298	4.18E-16	Fib3	XBP1	TRUE	FALSE
1.91E-20	0.388279835	0.93	0.473	4.82E-16	Fib3	TAGLN2	TRUE	FALSE
2.33E-20	0.302371725	0.43	0.127	5.88E-16	Fib3	SPRY2	FALSE	FALSE
2.38E-20	0.291813901	0.683	0.279	6.00E-16	Fib3	YIF1A	FALSE	FALSE
2.74E-20	0.259164154	0.739	0.3	6.92E-16	Fib3	B4GALT1	FALSE	FALSE
6.06E-20	0.257981333	0.739	0.302	1.53E-15	Fib3	EVA1B	FALSE	FALSE
7.22E-20	0.814796296	0.951	0.521	1.82E-15	Fib3	HSPA5	TRUE	FALSE
7.46E-20	0.5325159	0.965	0.572	1.88E-15	Fib3	PFN1	FALSE	FALSE
1.37E-19	0.375281633	0.866	0.399	3.46E-15	Fib3	PHPT1	FALSE	FALSE
1.61E-19	0.637153612	0.887	0.457	4.06E-15	Fib3	FMOD	FALSE	FALSE
1.88E-19	0.400514055	0.93	0.478	4.75E-15	Fib3	TMED10	FALSE	FALSE
1.90E-19	0.286715881	0.838	0.387	4.80E-15	Fib3	KDELR1	FALSE	FALSE
2.18E-19	0.266170337	0.81	0.363	5.50E-15	Fib3	BNIP3L	FALSE	FALSE
2.25E-19	0.258336494	0.873	0.389	5.67E-15	Fib3	TMED2	FALSE	FALSE
2.61E-19	0.35176233	0.331	0.08	6.58E-15	Fib3	PAPPA	FALSE	FALSE
2.86E-19	0.299917039	0.831	0.389	7.21E-15	Fib3	PRDX5	TRUE	FALSE
3.20E-19	0.323982155	0.944	0.491	8.09E-15	Fib3	MZT2B	FALSE	FALSE
3.25E-19	0.469781306	1	0.924	8.20E-15	Fib3	TMSB10	FALSE	FALSE
5.55E-19	0.284975812	0.732	0.307	1.40E-14	Fib3	KLF4	TRUE	FALSE
5.74E-19	0.265901478	0.923	0.446	1.45E-14	Fib3	APLP2	FALSE	TRUE
6.30E-19	0.526342646	0.972	0.615	1.59E-14	Fib3	GNAS	FALSE	FALSE
7.05E-19	0.305457189	0.415	0.122	1.78E-14	Fib3	TFRC	FALSE	FALSE
7.32E-19	0.814014855	0.838	0.442	1.85E-14	Fib3	TIMP3	FALSE	FALSE
8.53E-19	0.261237522	0.599	0.219	2.15E-14	Fib3	RGS3	FALSE	FALSE
1.46E-18	0.552758972	0.394	0.12	3.68E-14	Fib3	AKR1C2	FALSE	FALSE
1.50E-18	0.370045917	0.887	0.439	3.80E-14	Fib3	HIF1A	TRUE	FALSE
1.97E-18	0.290536067	0.901	0.426	4.98E-14	Fib3	CAVIN3	FALSE	FALSE
3.14E-18	0.387358594	0.866	0.429	7.93E-14	Fib3	OSTC	FALSE	FALSE
3.60E-18	0.369110286	0.599	0.23	9.09E-14	Fib3	TSC22D3	FALSE	FALSE
4.62E-18	0.302014917	0.599	0.227	1.17E-13	Fib3	TOB1	FALSE	FALSE
5.26E-18	0.253736842	0.366	0.102	1.33E-13	Fib3	MIR222HG	FALSE	FALSE
6.03E-18	0.359830805	0.923	0.501	1.52E-13	Fib3	CLIC1	FALSE	FALSE
8.99E-18	0.563447381	0.57	0.213	2.27E-13	Fib3	SLPI	FALSE	FALSE
1.12E-17	0.435676718	1	0.746	2.83E-13	Fib3	MYL6	FALSE	FALSE
1.78E-17	0.497934823	0.93	0.463	4.49E-13	Fib3	VCAN	FALSE	FALSE
2.47E-17	0.342155121	0.887	0.461	6.24E-13	Fib3	ARPC2	FALSE	FALSE
5.98E-17	0.474974469	1	0.898	1.51E-12	Fib3	ACTB	FALSE	FALSE
6.30E-17	0.36537703	0.676	0.284	1.59E-12	Fib3	TYMP	FALSE	FALSE

1.31E-16	0.263049334	0.866	0.392	3.30E-12	Fib3	ATF4	TRUE	FALSE
1.70E-16	0.34449076	0.951	0.541	4.29E-12	Fib3	RABAC1	FALSE	FALSE
1.88E-16	0.305802749	0.923	0.468	4.75E-12	Fib3	EIF4G2	FALSE	FALSE
3.73E-16	0.263670273	0.894	0.439	9.40E-12	Fib3	HSBP1	FALSE	FALSE
3.79E-16	0.326447796	0.887	0.467	9.57E-12	Fib3	RHOC	FALSE	FALSE
4.87E-16	0.353496933	0.958	0.533	1.23E-11	Fib3	TMBIM6	FALSE	FALSE
4.92E-16	0.557334063	0.38	0.118	1.24E-11	Fib3	RRAD	FALSE	FALSE
1.85E-15	0.291752538	0.937	0.498	4.67E-11	Fib3	S100A13	FALSE	FALSE
2.08E-15	0.265048207	0.866	0.468	5.25E-11	Fib3	ATP5MD	FALSE	FALSE
9.62E-15	0.371542443	1	0.822	2.43E-10	Fib3	CD63	FALSE	TRUE
1.05E-14	0.323107914	0.901	0.448	2.64E-10	Fib3	PPP1R14B	FALSE	FALSE
2.89E-14	0.315134234	0.556	0.215	7.29E-10	Fib3	CRLF1	FALSE	FALSE
3.40E-14	0.444478062	0.831	0.43	8.59E-10	Fib3	EMP1	FALSE	TRUE
5.14E-14	0.296317162	0.88	0.476	1.30E-09	Fib3	DBI	FALSE	FALSE
7.24E-14	0.316190802	0.894	0.481	1.83E-09	Fib3	RAN	TRUE	FALSE
1.13E-13	0.360297444	0.965	0.624	2.84E-09	Fib3	OAZ1	FALSE	FALSE
1.64E-13	0.301601954	0.894	0.454	4.15E-09	Fib3	TUBB	FALSE	FALSE
1.75E-13	0.884169627	0.761	0.4	4.41E-09	Fib3	SOD2	FALSE	FALSE
6.87E-13	0.290357837	0.556	0.236	1.73E-08	Fib3	AKR1C1	FALSE	FALSE
2.70E-12	0.267111404	0.965	0.55	6.81E-08	Fib3	CD99	FALSE	FALSE
3.52E-12	0.361041506	0.965	0.608	8.88E-08	Fib3	CFL1	FALSE	FALSE
3.74E-12	0.37698689	0.831	0.436	9.45E-08	Fib3	KLF6	TRUE	FALSE
4.71E-12	0.323999666	0.937	0.581	1.19E-07	Fib3	OST4	FALSE	FALSE
6.40E-12	0.254665032	0.915	0.532	1.61E-07	Fib3	RHOA	FALSE	FALSE
2.08E-11	0.388752203	0.894	0.544	5.25E-07	Fib3	ABI3BP	FALSE	FALSE
4.46E-11	0.409711765	0.648	0.31	1.13E-06	Fib3	S100A1	FALSE	FALSE
1.54E-10	0.4370912	0.993	0.834	3.89E-06	Fib3	MT-CO2	FALSE	FALSE
1.58E-10	0.284134527	0.972	0.687	3.99E-06	Fib3	CALM1	FALSE	FALSE
3.12E-10	0.28649215	0.958	0.607	7.87E-06	Fib3	GSTP1	FALSE	FALSE
4.82E-10	0.577485027	0.317	0.12	1.22E-05	Fib3	SLC7A2	FALSE	TRUE
5.98E-10	0.28962487	0.965	0.675	1.51E-05	Fib3	DSTN	FALSE	FALSE
2.60E-09	0.251578241	0.627	0.327	6.56E-05	Fib3	CYBA	FALSE	FALSE
7.80E-09	0.383127089	0.993	0.844	0.000196864	Fib3	MT-CO3	FALSE	FALSE
2.01E-07	0.269502022	0.972	0.63	0.005068847	Fib3	PPIB	FALSE	FALSE
2.95E-07	0.737443407	1	0.843	0.007446945	Fib3	TIMP1	FALSE	FALSE
3.16E-07	0.6055058	0.782	0.497	0.007986211	Fib3	CTGF	FALSE	FALSE
4.71E-07	0.447191209	0.69	0.42	0.011894164	Fib3	FGFBP2	FALSE	FALSE
1.34E-06	0.292096295	0.88	0.627	0.033850591	Fib3	CRYAB	FALSE	FALSE
2.30E-127	1.177774665	0.61	0.025	5.81E-123	Osteo	ALPL	FALSE	TRUE
2.67E-97	0.904484	0.715	0.091	6.74E-93	Osteo	RUNX2	TRUE	FALSE
3.36E-96	0.908859539	0.58	0.051	8.48E-92	Osteo	ARL4C	FALSE	FALSE
1.96E-95	0.690899929	0.475	0.022	4.95E-91	Osteo	TMEM119	FALSE	FALSE
3.67E-88	0.951143231	0.68	0.097	9.26E-84	Osteo	OLFML2B	FALSE	FALSE
2.82E-86	0.593257697	0.7	0.104	7.11E-82	Osteo	CRNDE	FALSE	FALSE
9.02E-86	0.56103493	0.525	0.043	2.27E-81	Osteo	OLFML3	FALSE	FALSE
3.86E-85	0.474140149	0.385	0.012	9.74E-81	Osteo	SLC1A3	FALSE	TRUE
1.01E-84	1.154890311	0.865	0.205	2.54E-80	Osteo	CLEC11A	FALSE	FALSE
2.70E-78	0.558510361	0.54	0.054	6.82E-74	Osteo	SRGN	FALSE	FALSE
3.22E-78	0.448289239	0.375	0.015	8.12E-74	Osteo	DLX5	TRUE	FALSE
4.84E-78	0.72030478	0.415	0.023	1.22E-73	Osteo	PTX3	FALSE	FALSE
1.61E-77	0.613399329	0.495	0.043	4.05E-73	Osteo	PTN	FALSE	FALSE
4.96E-77	0.874968825	0.855	0.207	1.25E-72	Osteo	EMILIN1	FALSE	FALSE
4.69E-75	0.638969503	0.575	0.076	1.18E-70	Osteo	CNN2	FALSE	FALSE
1.64E-74	0.323622615	0.44	0.031	4.14E-70	Osteo	ENPP2	FALSE	FALSE
1.71E-74	0.441114791	0.33	0.009	4.31E-70	Osteo	PMAIP1	FALSE	FALSE
3.67E-73	0.664773022	0.73	0.137	9.25E-69	Osteo	SNAI2	TRUE	FALSE
9.72E-73	0.539403173	0.43	0.032	2.45E-68	Osteo	VCAM1	FALSE	TRUE
1.35E-70	0.51220933	0.595	0.088	3.40E-66	Osteo	LSP1	FALSE	FALSE
8.79E-70	0.391862419	0.615	0.096	2.22E-65	Osteo	MDFI	FALSE	FALSE
1.35E-69	0.734028715	0.73	0.155	3.41E-65	Osteo	JPT1	FALSE	FALSE
4.83E-69	0.310411166	0.49	0.051	1.22E-64	Osteo	STK17B	FALSE	FALSE
5.24E-68	0.706775302	0.7	0.143	1.32E-63	Osteo	ERRFI1	FALSE	FALSE
3.53E-67	1.942080678	0.48	0.055	8.91E-63	Osteo	POSTN	FALSE	FALSE
6.06E-67	0.644499337	0.705	0.142	1.53E-62	Osteo	MYC	TRUE	FALSE
1.39E-66	0.666982203	0.61	0.095	3.51E-62	Osteo	MDK	FALSE	FALSE
1.49E-66	0.410185191	0.605	0.094	3.76E-62	Osteo	SKA2	FALSE	FALSE
8.73E-65	0.3605559	0.51	0.065	2.20E-60	Osteo	HNMT	FALSE	FALSE
1.49E-64	0.470053637	0.41	0.036	3.75E-60	Osteo	CENPW	FALSE	FALSE
1.78E-64	0.71579284	0.72	0.153	4.49E-60	Osteo	CDH11	FALSE	TRUE
1.95E-64	0.46408417	0.44	0.044	4.91E-60	Osteo	MXRA5	FALSE	FALSE
2.05E-64	1.023092028	0.665	0.131	5.18E-60	Osteo	CYP1B1	FALSE	FALSE
5.79E-64	0.273299324	0.26	0.003	1.46E-59	Osteo	FAT3	FALSE	TRUE
6.50E-64	0.369009895	0.595	0.094	1.64E-59	Osteo	PLEKHA5	FALSE	FALSE
2.43E-63	0.355373368	0.315	0.014	6.14E-59	Osteo	TNFSF11	FALSE	TRUE
8.78E-63	0.70651619	0.7	0.147	2.21E-58	Osteo	GADD45A	TRUE	FALSE
1.17E-61	0.836786794	0.675	0.136	2.95E-57	Osteo	MAFB	TRUE	FALSE
2.74E-61	0.491896145	0.375	0.03	6.90E-57	Osteo	TYMS	FALSE	FALSE
1.91E-60	0.49790417	0.52	0.077	4.81E-56	Osteo	PIM1	FALSE	FALSE
7.10E-60	0.803731914	0.67	0.148	1.79E-55	Osteo	IRF1	TRUE	FALSE
7.99E-60	0.326288103	0.29	0.012	2.02E-55	Osteo	LRRC15	FALSE	TRUE
1.03E-59	0.748533131	0.52	0.079	2.59E-55	Osteo	CKS2	FALSE	FALSE
2.80E-59	0.601621439	0.62	0.123	7.06E-55	Osteo	CTHRC1	FALSE	FALSE
3.74E-59	0.950146148	0.905	0.333	9.43E-55	Osteo	PCOLCE	FALSE	FALSE
5.16E-59	0.514939472	0.7	0.149	1.30E-54	Osteo	TWIST1	TRUE	FALSE
5.79E-59	0.462605576	0.4	0.04	1.46E-54	Osteo	SATB2	TRUE	FALSE
2.83E-58	0.787277622	0.845	0.248	7.14E-54	Osteo	COL5A1	FALSE	FALSE

3.11E-58	0.502278016	0.665	0.14	7.85E-54	Osteo	IER5	FALSE	FALSE
4.44E-58	0.450641538	0.67	0.149	1.12E-53	Osteo	COTL1	FALSE	FALSE
5.25E-58	1.035033023	0.915	0.345	1.32E-53	Osteo	TPM1	FALSE	FALSE
7.35E-58	0.373235551	0.64	0.126	1.85E-53	Osteo	MARCKSL1	FALSE	FALSE
9.91E-58	0.590030007	0.46	0.062	2.50E-53	Osteo	BAMBI	FALSE	TRUE
1.35E-57	0.514301822	0.575	0.105	3.42E-53	Osteo	FOXC2	TRUE	FALSE
3.95E-57	0.440665769	0.66	0.137	9.98E-53	Osteo	COLEC12	FALSE	FALSE
4.36E-57	0.38821595	0.515	0.077	1.10E-52	Osteo	COL16A1	FALSE	FALSE
4.83E-57	0.393029179	0.355	0.029	1.22E-52	Osteo	SOCS1	FALSE	FALSE
7.05E-57	0.355760716	0.33	0.023	1.78E-52	Osteo	CREB3L1	TRUE	FALSE
1.76E-56	0.620977798	0.81	0.239	4.44E-52	Osteo	PSME2	FALSE	FALSE
1.56E-55	0.567320069	0.73	0.177	3.93E-51	Osteo	RGS3	FALSE	FALSE
1.85E-55	0.477472916	0.535	0.093	4.66E-51	Osteo	GLIPR1	FALSE	TRUE
4.30E-55	0.502265761	0.66	0.146	1.09E-50	Osteo	CHCHD10	FALSE	FALSE
6.36E-55	0.407890718	0.315	0.021	1.61E-50	Osteo	PTH1R	FALSE	TRUE
7.41E-55	0.436439766	0.645	0.145	1.87E-50	Osteo	NME4	FALSE	FALSE
3.65E-54	0.358663676	0.31	0.021	9.20E-50	Osteo	TK1	FALSE	FALSE
8.06E-54	0.573778555	0.685	0.166	2.03E-49	Osteo	FKBP11	FALSE	FALSE
1.28E-53	2.259114432	0.89	0.402	3.24E-49	Osteo	COL1A1	FALSE	FALSE
1.41E-53	0.649311226	0.355	0.034	3.55E-49	Osteo	PCLAF	FALSE	FALSE
4.83E-53	0.354536245	0.46	0.066	1.22E-48	Osteo	LMO7	FALSE	FALSE
7.05E-53	0.931090151	0.87	0.348	1.78E-48	Osteo	SERPINH1	FALSE	FALSE
7.87E-53	0.828486767	0.725	0.197	1.99E-48	Osteo	UBE2S	FALSE	FALSE
2.53E-52	0.495691307	0.62	0.139	6.38E-48	Osteo	ODF2L	FALSE	FALSE
4.93E-52	0.58535704	0.34	0.031	1.24E-47	Osteo	CKAP2	FALSE	FALSE
1.60E-51	1.200120065	0.935	0.445	4.05E-47	Osteo	MARCKS	FALSE	FALSE
2.62E-51	1.2515219	0.82	0.29	6.61E-47	Osteo	IER3	FALSE	FALSE
2.66E-51	0.718653439	0.58	0.118	6.72E-47	Osteo	DDIT4	FALSE	FALSE
3.31E-51	0.32368758	0.455	0.066	8.36E-47	Osteo	PCDH18	FALSE	TRUE
4.03E-51	0.269611308	0.385	0.042	1.02E-46	Osteo	GMFG	FALSE	FALSE
4.24E-51	1.062678774	0.335	0.031	1.07E-46	Osteo	CXCL2	FALSE	FALSE
1.28E-50	0.467891798	0.505	0.088	3.24E-46	Osteo	LIFR	FALSE	TRUE
1.81E-50	0.451125704	0.75	0.202	4.56E-46	Osteo	C4orf48	FALSE	FALSE
3.26E-50	0.635960552	0.4	0.051	8.22E-46	Osteo	TNFAIP3	FALSE	FALSE
1.93E-49	0.338692219	0.295	0.021	4.86E-45	Osteo	MAD2L1	FALSE	FALSE
2.50E-49	0.379573264	0.725	0.187	6.30E-45	Osteo	CYTOR	FALSE	FALSE
4.04E-49	0.279936889	0.595	0.13	1.02E-44	Osteo	OSTF1	FALSE	FALSE
4.80E-49	0.346901985	0.475	0.08	1.21E-44	Osteo	CSF1	FALSE	TRUE
7.73E-49	0.296224128	0.37	0.042	1.95E-44	Osteo	NID2	FALSE	FALSE
9.54E-49	0.30342907	0.495	0.087	2.41E-44	Osteo	ADAMTS2	FALSE	FALSE
1.43E-48	0.347405966	0.46	0.073	3.60E-44	Osteo	S1PR3	FALSE	TRUE
1.79E-48	0.514095624	0.405	0.054	4.53E-44	Osteo	ADM	FALSE	FALSE
2.59E-48	0.291377475	0.49	0.083	6.54E-44	Osteo	H2AFX	FALSE	FALSE
6.03E-48	0.567907399	0.305	0.026	1.52E-43	Osteo	CDKN3	FALSE	FALSE
6.09E-48	0.577394484	0.675	0.165	1.54E-43	Osteo	COL12A1	FALSE	FALSE
6.99E-48	0.374887246	0.59	0.131	1.76E-43	Osteo	EML4	FALSE	FALSE
4.89E-47	0.403272588	0.74	0.211	1.23E-42	Osteo	GPX8	FALSE	FALSE
6.37E-47	0.36791858	0.41	0.061	1.61E-42	Osteo	PTPRD	FALSE	TRUE
6.62E-47	0.361257642	0.22	0.008	1.67E-42	Osteo	CCNB2	FALSE	FALSE
8.54E-47	0.253916846	0.51	0.097	2.16E-42	Osteo	DTYMK	FALSE	FALSE
1.03E-46	0.479281402	0.735	0.21	2.61E-42	Osteo	FRMD6	FALSE	FALSE
1.88E-46	0.908623058	0.55	0.115	4.74E-42	Osteo	STMN1	FALSE	FALSE
1.98E-46	0.304277832	0.295	0.025	5.01E-42	Osteo	HEY2	TRUE	FALSE
2.70E-46	0.522640056	0.865	0.303	6.82E-42	Osteo	AP2S1	FALSE	FALSE
2.75E-46	0.358084114	0.235	0.011	6.95E-42	Osteo	PBK	FALSE	FALSE
5.91E-46	0.295887127	0.345	0.038	1.49E-41	Osteo	FAM241A	FALSE	FALSE
7.64E-46	0.317696438	0.285	0.023	1.93E-41	Osteo	CD83	FALSE	TRUE
8.99E-46	0.430831025	0.755	0.231	2.27E-41	Osteo	11-Sep	FALSE	FALSE
9.35E-46	0.391475419	0.805	0.257	2.36E-41	Osteo	GTF3C6	FALSE	FALSE
1.96E-45	0.301747645	0.65	0.164	4.95E-41	Osteo	FKBP7	FALSE	FALSE
3.69E-45	0.489224303	0.465	0.081	9.32E-41	Osteo	SULF1	FALSE	FALSE
1.06E-44	0.345400531	0.55	0.117	2.68E-40	Osteo	TLE4	FALSE	FALSE
1.25E-44	0.386641798	0.805	0.242	3.16E-40	Osteo	PPIC	FALSE	FALSE
2.51E-44	0.3536029	0.3	0.029	6.34E-40	Osteo	FRMD6-AS1	FALSE	FALSE
3.20E-44	0.341674782	0.71	0.209	8.08E-40	Osteo	NDUFAF8	FALSE	FALSE
3.27E-44	0.274923801	0.45	0.082	8.25E-40	Osteo	RAP2B	FALSE	FALSE
3.35E-44	0.314195	0.59	0.144	8.45E-40	Osteo	NUDT1	FALSE	FALSE
3.96E-44	0.57285911	0.87	0.323	9.99E-40	Osteo	HMGN1	FALSE	FALSE
7.97E-44	0.372713852	0.205	0.007	2.01E-39	Osteo	CDK1	FALSE	FALSE
8.39E-44	0.526282602	0.705	0.207	2.12E-39	Osteo	NFKBIZ	FALSE	FALSE
9.37E-44	0.321442118	0.42	0.069	2.36E-39	Osteo	CCDC34	FALSE	FALSE
1.00E-43	0.285007072	0.66	0.171	2.53E-39	Osteo	TGFB1	FALSE	FALSE
1.09E-43	0.482136177	0.765	0.247	2.74E-39	Osteo	KLF10	TRUE	FALSE
1.52E-43	0.583516957	0.6	0.147	3.84E-39	Osteo	SLC2A3	FALSE	TRUE
1.71E-43	0.572884286	0.235	0.014	4.31E-39	Osteo	NUSAP1	FALSE	FALSE
2.13E-43	0.285893726	0.355	0.047	5.37E-39	Osteo	GGH	FALSE	FALSE
2.43E-43	0.573126691	0.79	0.259	6.13E-39	Osteo	ANKRD28	FALSE	FALSE
4.08E-43	0.568143445	0.24	0.015	1.03E-38	Osteo	UBE2C	FALSE	FALSE
5.16E-43	0.669677624	0.23	0.013	1.30E-38	Osteo	MKI67	FALSE	FALSE
8.78E-43	0.397146759	0.775	0.249	2.22E-38	Osteo	HMGN3	TRUE	FALSE
1.07E-42	0.402409453	0.795	0.27	2.69E-38	Osteo	SPATS2L	FALSE	FALSE
1.66E-42	0.298646073	0.46	0.088	4.19E-38	Osteo	TUSC3	FALSE	FALSE
2.70E-42	0.935604943	0.27	0.024	6.82E-38	Osteo	CENPF	FALSE	FALSE
4.04E-42	0.435503384	0.73	0.226	1.02E-37	Osteo	ENAH	FALSE	FALSE
4.39E-42	0.298846294	0.48	0.096	1.11E-37	Osteo	DSEL	FALSE	FALSE
5.35E-42	0.777652975	0.25	0.019	1.35E-37	Osteo	TOP2A	FALSE	FALSE

5.73E-42	0.315273394	0.67	0.186	1.44E-37	Osteo	BAX	TRUE	FALSE
7.38E-42	0.880480644	0.865	0.371	1.86E-37	Osteo	ZFP36	FALSE	FALSE
9.05E-42	0.27840701	0.46	0.088	2.28E-37	Osteo	TIPARP	FALSE	FALSE
1.01E-41	0.344512829	0.725	0.224	2.55E-37	Osteo	GTF3A	TRUE	FALSE
1.11E-41	0.531203111	0.265	0.022	2.80E-37	Osteo	BIRC5	FALSE	FALSE
1.28E-41	0.427814689	0.48	0.096	3.23E-37	Osteo	SMC4	FALSE	FALSE
1.96E-41	0.269951737	0.65	0.177	4.95E-37	Osteo	EFEMP2	FALSE	FALSE
3.63E-41	0.503680161	0.845	0.329	9.17E-37	Osteo	LDHB	FALSE	FALSE
3.91E-41	0.305777919	0.775	0.251	9.87E-37	Osteo	LY6E	FALSE	TRUE
5.56E-41	0.308423194	0.52	0.117	1.40E-36	Osteo	MIR4435-2HG	FALSE	FALSE
6.29E-41	0.376817446	0.46	0.09	1.59E-36	Osteo	C12orf75	FALSE	FALSE
6.85E-41	0.26725216	0.75	0.23	1.73E-36	Osteo	CTSZ	FALSE	FALSE
1.36E-40	0.307827115	0.38	0.059	3.43E-36	Osteo	GADD45G	FALSE	FALSE
1.56E-40	0.487353269	0.275	0.026	3.93E-36	Osteo	CCNB1	FALSE	FALSE
1.59E-40	0.357847009	0.735	0.235	4.00E-36	Osteo	TIMM8B	FALSE	FALSE
2.16E-40	0.289288612	0.245	0.019	5.46E-36	Osteo	LMNB1	FALSE	FALSE
2.76E-40	0.718105446	0.57	0.146	6.97E-36	Osteo	HMGB2	TRUE	FALSE
3.83E-40	0.452450784	0.76	0.254	9.68E-36	Osteo	NME1	TRUE	FALSE
6.36E-40	0.441082537	0.48	0.098	1.60E-35	Osteo	BASP1	FALSE	FALSE
3.61E-39	0.360695178	0.795	0.264	9.11E-35	Osteo	FKBP10	FALSE	FALSE
1.09E-38	0.452566448	0.62	0.175	2.74E-34	Osteo	COPZ2	FALSE	FALSE
1.53E-38	0.275198688	0.6	0.161	3.86E-34	Osteo	MGMT	FALSE	FALSE
1.55E-38	0.325578665	0.71	0.215	3.91E-34	Osteo	IKBIP	FALSE	FALSE
2.50E-38	0.266109054	0.55	0.137	6.30E-34	Osteo	DCUN1D5	FALSE	FALSE
2.57E-38	0.540456303	0.815	0.289	6.47E-34	Osteo	GJA1	FALSE	TRUE
3.36E-38	0.302252088	0.53	0.128	8.48E-34	Osteo	CERCAM	FALSE	FALSE
5.39E-38	0.258225398	0.38	0.063	1.36E-33	Osteo	SLC40A1	FALSE	TRUE
5.46E-38	0.369948285	0.765	0.256	1.38E-33	Osteo	RCN1	FALSE	FALSE
5.88E-38	0.326873723	0.625	0.176	1.48E-33	Osteo	NUDT4	FALSE	FALSE
7.45E-38	0.603671434	0.94	0.433	1.88E-33	Osteo	TPM2	FALSE	FALSE
9.91E-38	0.417328021	0.81	0.283	2.50E-33	Osteo	RCN3	FALSE	FALSE
1.03E-37	0.473505648	0.22	0.015	2.59E-33	Osteo	TPX2	FALSE	FALSE
1.08E-37	0.279470132	0.375	0.063	2.73E-33	Osteo	KPNA2	FALSE	FALSE
2.77E-37	0.337840352	0.855	0.316	6.99E-33	Osteo	SLC25A5	FALSE	FALSE
2.89E-37	0.75549204	0.365	0.065	7.30E-33	Osteo	PTTG1	FALSE	FALSE
3.64E-37	0.80158402	0.975	0.533	9.19E-33	Osteo	BGN	FALSE	FALSE
3.75E-37	0.348736961	0.84	0.306	9.47E-33	Osteo	DNAJC15	FALSE	FALSE
3.88E-37	1.030252772	0.89	0.462	9.80E-33	Osteo	GADD45B	FALSE	FALSE
5.91E-37	1.000278076	0.965	0.625	1.49E-32	Osteo	LUM	FALSE	FALSE
6.85E-37	0.324662119	0.525	0.13	1.73E-32	Osteo	MAFF	TRUE	FALSE
1.48E-36	0.47715829	0.6	0.176	3.74E-32	Osteo	INTS6	FALSE	FALSE
1.69E-36	0.271627818	0.76	0.249	4.26E-32	Osteo	UBE2N	FALSE	FALSE
1.70E-36	0.710822582	0.665	0.206	4.29E-32	Osteo	KCNQ1OT1	FALSE	FALSE
4.63E-36	0.57552754	0.885	0.346	1.17E-31	Osteo	RPL22L1	FALSE	FALSE
5.51E-36	0.325640303	0.53	0.135	1.39E-31	Osteo	SNHG3	FALSE	FALSE
5.96E-36	1.64673793	0.98	0.589	1.50E-31	Osteo	COL1A2	FALSE	FALSE
6.86E-36	0.45419505	0.855	0.326	1.73E-31	Osteo	HNRNPAB	FALSE	FALSE
7.05E-36	0.253087874	0.595	0.167	1.78E-31	Osteo	CKLF	FALSE	FALSE
7.97E-36	0.587771658	0.925	0.401	2.01E-31	Osteo	RRBP1	FALSE	FALSE
8.94E-36	0.399513689	0.86	0.322	2.26E-31	Osteo	SDC2	FALSE	TRUE
1.06E-35	0.327384056	0.785	0.259	2.67E-31	Osteo	RND3	FALSE	FALSE
1.48E-35	0.423525696	0.655	0.203	3.74E-31	Osteo	SERTAD1	FALSE	FALSE
3.95E-35	0.558712374	0.44	0.095	9.98E-31	Osteo	RRAD	FALSE	FALSE
6.93E-35	1.665316291	0.885	0.475	1.75E-30	Osteo	COL3A1	FALSE	FALSE
7.82E-35	0.570413966	0.43	0.096	1.97E-30	Osteo	SGMS2	FALSE	FALSE
8.02E-35	0.28038988	0.29	0.038	2.02E-30	Osteo	HMGB3	TRUE	FALSE
9.38E-35	0.432153831	0.855	0.344	2.37E-30	Osteo	DRAP1	TRUE	FALSE
1.15E-34	0.343397178	0.39	0.075	2.91E-30	Osteo	RGS2	FALSE	FALSE
1.26E-34	0.497520863	1	0.842	3.19E-30	Osteo	SERF2	FALSE	FALSE
2.05E-34	0.645176421	0.74	0.257	5.16E-30	Osteo	TGFBI	FALSE	FALSE
2.10E-34	0.299587172	0.635	0.191	5.29E-30	Osteo	MRPL18	FALSE	FALSE
2.35E-34	0.321948694	0.58	0.163	5.92E-30	Osteo	FKBP14	FALSE	FALSE
2.84E-34	0.398832797	0.9	0.358	7.16E-30	Osteo	MCL1	FALSE	FALSE
4.23E-34	0.664347479	0.915	0.402	1.07E-29	Osteo	KLF6	TRUE	FALSE
4.47E-34	0.29472049	0.615	0.182	1.13E-29	Osteo	RPA3	FALSE	FALSE
4.73E-34	0.471646393	0.945	0.439	1.19E-29	Osteo	SUB1	FALSE	FALSE
5.11E-34	0.336103695	0.26	0.031	1.29E-29	Osteo	KIF20B	FALSE	FALSE
6.30E-34	0.613826257	0.84	0.326	1.59E-29	Osteo	SOX4	TRUE	FALSE
6.41E-34	0.444956896	0.895	0.401	1.62E-29	Osteo	ATP5MC3	FALSE	FALSE
6.61E-34	0.437555975	0.83	0.334	1.67E-29	Osteo	RANBP1	FALSE	FALSE
1.02E-33	0.320195965	0.395	0.08	2.57E-29	Osteo	ABHD5	FALSE	FALSE
1.13E-33	0.323283169	0.635	0.202	2.86E-29	Osteo	HSPB11	FALSE	FALSE
1.39E-33	0.670770707	0.9	0.432	3.51E-29	Osteo	TUBB	FALSE	FALSE
1.64E-33	0.807049169	0.725	0.278	4.14E-29	Osteo	APOE	FALSE	FALSE
1.66E-33	0.478131233	0.705	0.241	4.19E-29	Osteo	PHLDA1	FALSE	FALSE
1.98E-33	0.397855887	0.205	0.016	4.99E-29	Osteo	CENPE	FALSE	FALSE
2.38E-33	0.3682406	0.72	0.258	6.01E-29	Osteo	LSM5	FALSE	FALSE
2.75E-33	0.266161726	0.65	0.202	6.93E-29	Osteo	RBBP6	FALSE	FALSE
3.14E-33	0.316968068	0.835	0.311	7.92E-29	Osteo	TCEAL9	FALSE	FALSE
4.12E-33	0.25864021	0.57	0.156	1.04E-28	Osteo	NREP	FALSE	FALSE
5.68E-33	0.255480249	0.435	0.098	1.43E-28	Osteo	ING2	FALSE	FALSE
1.42E-32	0.550753106	0.915	0.425	3.60E-28	Osteo	EIF5A	FALSE	FALSE
1.53E-32	0.359321077	0.835	0.339	3.86E-28	Osteo	CCNL1	FALSE	FALSE
1.79E-32	0.868782949	0.96	0.782	4.51E-28	Osteo	MT-CO1	FALSE	FALSE
2.09E-32	0.256041896	0.685	0.225	5.28E-28	Osteo	MRPL14	FALSE	FALSE
2.59E-32	0.31330035	0.825	0.318	6.53E-28	Osteo	AURKAIP1	FALSE	FALSE

2.70E-32	0.295575134	0.86	0.369	6.82E-28	Osteo	NDUFB11	FALSE	FALSE
2.82E-32	0.380590984	0.86	0.349	7.12E-28	Osteo	SNRPF	FALSE	FALSE
1.09E-31	0.678061244	0.83	0.381	2.76E-27	Osteo	SOCS3	FALSE	FALSE
1.23E-31	0.308339453	0.81	0.313	3.10E-27	Osteo	PRELID1	FALSE	FALSE
1.44E-31	0.258697941	0.82	0.328	3.64E-27	Osteo	NDUFS6	FALSE	FALSE
1.46E-31	0.711916509	0.765	0.322	3.68E-27	Osteo	NFKBIA	FALSE	FALSE
1.62E-31	0.396254199	0.88	0.374	4.09E-27	Osteo	PHPT1	FALSE	FALSE
2.20E-31	0.272281119	0.245	0.03	5.55E-27	Osteo	SGO2	FALSE	FALSE
2.22E-31	0.307835588	0.775	0.287	5.59E-27	Osteo	CYBA	FALSE	FALSE
2.55E-31	0.379155717	0.845	0.352	6.45E-27	Osteo	SNRPE	FALSE	FALSE
2.80E-31	0.253613071	0.65	0.197	7.07E-27	Osteo	NRP2	FALSE	TRUE
3.71E-31	0.261700337	0.625	0.207	9.35E-27	Osteo	LSM4	FALSE	FALSE
3.89E-31	0.252241305	0.75	0.27	9.82E-27	Osteo	PSMA3	FALSE	FALSE
4.19E-31	0.525810485	0.94	0.453	1.06E-26	Osteo	RAN	TRUE	FALSE
5.38E-31	0.47368937	0.89	0.417	1.36E-26	Osteo	KDELR2	FALSE	FALSE
5.99E-31	0.863008911	0.885	0.439	1.51E-26	Osteo	H2AFZ	TRUE	FALSE
6.98E-31	0.317131675	0.245	0.032	1.76E-26	Osteo	UBE2T	FALSE	FALSE
7.65E-31	0.582311453	0.985	0.587	1.93E-26	Osteo	CFL1	FALSE	FALSE
1.85E-30	0.407531342	0.9	0.402	4.66E-26	Osteo	OSTC	FALSE	FALSE
1.92E-30	0.362101515	0.825	0.324	4.84E-26	Osteo	TSC22D1	TRUE	FALSE
2.44E-30	0.313173406	0.87	0.364	6.15E-26	Osteo	SRSF2	FALSE	FALSE
2.84E-30	0.522777456	0.985	0.613	7.15E-26	Osteo	RPS26	FALSE	FALSE
2.90E-30	0.471421853	0.875	0.363	7.31E-26	Osteo	PPP1R15A	FALSE	FALSE
3.02E-30	0.264836065	0.7	0.237	7.62E-26	Osteo	MYADM	FALSE	TRUE
3.86E-30	0.303659579	0.855	0.345	9.73E-26	Osteo	SNRPD1	FALSE	FALSE
4.59E-30	0.445948239	0.855	0.366	1.16E-25	Osteo	CBX3	FALSE	FALSE
4.97E-30	0.315922323	0.48	0.129	1.26E-25	Osteo	KDELR3	FALSE	FALSE
7.24E-30	0.453997505	0.91	0.439	1.83E-25	Osteo	UQCRQ	FALSE	FALSE
8.32E-30	0.592809478	0.54	0.164	2.10E-25	Osteo	HIST1H4C	FALSE	FALSE
1.05E-29	0.255624807	0.84	0.329	2.64E-25	Osteo	PRDX4	FALSE	FALSE
1.15E-29	0.502474218	0.7	0.257	2.91E-25	Osteo	ATF3	TRUE	FALSE
1.24E-29	0.634295986	0.22	0.025	3.14E-25	Osteo	CXCL3	FALSE	FALSE
1.29E-29	0.421629136	0.975	0.527	3.26E-25	Osteo	ATP5MC2	FALSE	FALSE
1.53E-29	0.445551301	0.84	0.349	3.87E-25	Osteo	TUBA1C	FALSE	FALSE
1.92E-29	0.310217498	0.875	0.378	4.84E-25	Osteo	UQCR10	FALSE	FALSE
2.58E-29	0.432623123	0.655	0.22	6.52E-25	Osteo	TNC	FALSE	FALSE
3.41E-29	2.086378	0.395	0.097	8.59E-25	Osteo	IBSP	FALSE	FALSE
4.68E-29	0.630687445	0.83	0.355	1.18E-24	Osteo	COL5A2	FALSE	FALSE
6.48E-29	0.324276695	0.915	0.404	1.63E-24	Osteo	ATP5MF	FALSE	FALSE
7.44E-29	0.260290675	0.28	0.046	1.88E-24	Osteo	FNDC1	FALSE	FALSE
9.20E-29	0.278149399	0.88	0.381	2.32E-24	Osteo	BANF1	FALSE	FALSE
1.70E-28	0.364597369	0.48	0.141	4.28E-24	Osteo	CKS1B	FALSE	FALSE
1.96E-28	0.701769571	0.945	0.617	4.94E-24	Osteo	MT-ND5	FALSE	FALSE
2.86E-28	0.308103333	0.875	0.359	7.21E-24	Osteo	GPX1	FALSE	FALSE
3.39E-28	0.630305823	0.95	0.526	8.55E-24	Osteo	TUBA1B	FALSE	FALSE
3.81E-28	0.283407736	0.725	0.277	9.62E-24	Osteo	BRD2	FALSE	FALSE
4.14E-28	0.338166017	0.74	0.285	1.05E-23	Osteo	H2AFV	FALSE	FALSE
4.15E-28	0.293445158	0.81	0.334	1.05E-23	Osteo	CYCS	TRUE	FALSE
6.02E-28	0.423786246	0.79	0.338	1.52E-23	Osteo	DUT	FALSE	FALSE
1.55E-27	0.336284541	0.465	0.123	3.92E-23	Osteo	CXCL12	FALSE	FALSE
1.92E-27	0.259760461	0.675	0.242	4.84E-23	Osteo	UACA	FALSE	FALSE
2.35E-27	0.433570238	0.845	0.387	5.94E-23	Osteo	HMGN2	FALSE	FALSE
3.35E-27	0.525085189	0.95	0.5	8.46E-23	Osteo	TPM4	FALSE	FALSE
3.56E-27	0.412187744	0.915	0.444	8.98E-23	Osteo	POLR2L	FALSE	FALSE
4.35E-27	0.356650719	0.65	0.228	1.10E-22	Osteo	ADAMTS1	FALSE	FALSE
4.57E-27	0.285940728	0.705	0.265	1.15E-22	Osteo	MAP4K4	FALSE	FALSE
5.58E-27	0.872055253	0.57	0.212	1.41E-22	Osteo	THY1	FALSE	TRUE
5.96E-27	0.469586363	0.865	0.413	1.50E-22	Osteo	HSPD1	FALSE	FALSE
8.13E-27	0.270225148	0.845	0.344	2.05E-22	Osteo	PFDN2	FALSE	FALSE
9.48E-27	0.649699252	0.97	0.83	2.39E-22	Osteo	MT-CO2	FALSE	FALSE
9.75E-27	0.492227219	0.675	0.252	2.46E-22	Osteo	ARL6IP1	FALSE	FALSE
1.42E-26	0.353893164	0.92	0.435	3.59E-22	Osteo	NDUFS5	FALSE	FALSE
1.47E-26	0.481019104	0.945	0.52	3.70E-22	Osteo	NDUFA4	FALSE	FALSE
1.71E-26	0.347308448	0.955	0.491	4.33E-22	Osteo	UQCRH	FALSE	FALSE
1.79E-26	0.549225715	0.67	0.248	4.53E-22	Osteo	HES1	TRUE	FALSE
1.84E-26	0.543266212	0.36	0.084	4.63E-22	Osteo	OMD	FALSE	FALSE
2.01E-26	0.526770181	0.965	0.614	5.08E-22	Osteo	PPIB	FALSE	FALSE
2.62E-26	0.907417883	0.325	0.071	6.62E-22	Osteo	HSPA6	FALSE	FALSE
4.02E-26	0.480449316	0.97	0.577	1.01E-21	Osteo	HNRNPA1	TRUE	FALSE
5.75E-26	0.490039942	0.89	0.438	1.45E-21	Osteo	TUBB4B	FALSE	FALSE
5.86E-26	0.270653274	0.81	0.346	1.48E-21	Osteo	ATP5MC1	FALSE	FALSE
7.75E-26	0.264363257	0.595	0.211	1.96E-21	Osteo	ANP32E	FALSE	FALSE
1.22E-25	0.254935875	0.35	0.082	3.07E-21	Osteo	WEE1	FALSE	FALSE
1.58E-25	0.338723428	0.315	0.069	3.98E-21	Osteo	LEPR	FALSE	TRUE
2.77E-25	0.296894187	0.78	0.341	6.99E-21	Osteo	MTATP6P1	FALSE	FALSE
3.07E-25	0.357515829	0.41	0.113	7.76E-21	Osteo	TRIB1	TRUE	FALSE
4.97E-25	0.435487154	0.915	0.45	1.25E-20	Osteo	HSPE1	FALSE	FALSE
5.64E-25	0.323365064	0.925	0.433	1.42E-20	Osteo	ARPC2	FALSE	FALSE
5.74E-25	0.252563574	0.46	0.14	1.45E-20	Osteo	BMP1	FALSE	FALSE
5.93E-25	0.272026037	0.845	0.363	1.50E-20	Osteo	KDELR1	FALSE	FALSE
6.15E-25	0.254206582	0.875	0.404	1.55E-20	Osteo	MRPL51	FALSE	FALSE
6.76E-25	0.25709045	0.845	0.364	1.71E-20	Osteo	TMEM167A	FALSE	FALSE
7.34E-25	0.434737718	0.36	0.09	1.85E-20	Osteo	DPT	FALSE	FALSE
8.27E-25	0.345251648	0.395	0.105	2.09E-20	Osteo	WDFY2	FALSE	FALSE
9.30E-25	0.494375414	0.965	0.547	2.35E-20	Osteo	PTMS	FALSE	FALSE
9.51E-25	0.367707628	0.915	0.48	2.40E-20	Osteo	SEM1	FALSE	FALSE

1.11E-24	0.468533933	0.99	0.663	2.81E-20	Osteo	PPIA	FALSE	FALSE
1.14E-24	0.377014493	0.685	0.27	2.87E-20	Osteo	HSPH1	FALSE	FALSE
1.17E-24	0.37067107	0.915	0.45	2.95E-20	Osteo	DBI	FALSE	FALSE
1.21E-24	0.321157823	0.875	0.375	3.04E-20	Osteo	FSTL1	FALSE	FALSE
1.26E-24	0.280646546	0.515	0.163	3.19E-20	Osteo	UBALD2	FALSE	FALSE
1.82E-24	0.259294161	0.325	0.075	4.60E-20	Osteo	FZD1	FALSE	TRUE
1.85E-24	0.395175417	0.965	0.517	4.68E-20	Osteo	MYL12A	FALSE	FALSE
2.35E-24	0.301713249	0.47	0.147	5.94E-20	Osteo	PCNA	FALSE	FALSE
2.36E-24	0.514246888	0.9	0.462	5.96E-20	Osteo	CTGF	FALSE	FALSE
3.71E-24	0.276744056	0.94	0.445	9.36E-20	Osteo	POMP	FALSE	FALSE
3.79E-24	0.252222355	0.535	0.179	9.55E-20	Osteo	CTSK	FALSE	FALSE
4.01E-24	0.328535515	0.76	0.309	1.01E-19	Osteo	KLF2	TRUE	FALSE
5.66E-24	0.280854501	0.89	0.402	1.43E-19	Osteo	TMEM50A	FALSE	FALSE
5.91E-24	0.381406925	0.62	0.23	1.49E-19	Osteo	SGK1	FALSE	FALSE
7.62E-24	1.468279059	0.29	0.061	1.92E-19	Osteo	SPP1	FALSE	FALSE
2.21E-23	0.572267429	0.97	0.84	5.57E-19	Osteo	MT-ND4	FALSE	FALSE
2.54E-23	0.354824666	0.96	0.526	6.40E-19	Osteo	NAP1L1	TRUE	FALSE
2.65E-23	0.371506242	0.385	0.11	6.69E-19	Osteo	MFAP2	FALSE	FALSE
4.34E-23	0.384349826	0.56	0.204	1.10E-18	Osteo	ABL2	FALSE	FALSE
5.04E-23	0.344674279	0.93	0.485	1.27E-18	Osteo	ATP5PF	FALSE	FALSE
5.31E-23	0.411249445	0.975	0.557	1.34E-18	Osteo	OST4	FALSE	FALSE
5.55E-23	0.653921007	0.86	0.399	1.40E-18	Osteo	HTRA1	FALSE	FALSE
7.98E-23	0.268720623	0.94	0.44	2.01E-18	Osteo	GNG5	FALSE	FALSE
8.67E-23	0.335783967	0.905	0.468	2.19E-18	Osteo	SRSF3	FALSE	FALSE
1.75E-22	0.265850311	0.785	0.343	4.40E-18	Osteo	SRSF10	FALSE	FALSE
2.96E-22	0.344799664	0.915	0.471	7.48E-18	Osteo	RPS27L	FALSE	FALSE
2.97E-22	0.296279389	0.915	0.439	7.50E-18	Osteo	SERBP1	FALSE	FALSE
3.00E-22	0.270506098	0.87	0.427	7.57E-18	Osteo	COX8A	FALSE	FALSE
7.65E-22	0.956828369	0.965	0.662	1.93E-17	Osteo	SPARC	FALSE	FALSE
9.98E-22	0.331569221	0.9	0.444	2.52E-17	Osteo	DNAJA1	FALSE	FALSE
1.05E-21	0.304449894	0.89	0.4	2.64E-17	Osteo	EMP1	FALSE	TRUE
1.15E-21	0.53881049	0.965	0.841	2.91E-17	Osteo	MT-CO3	FALSE	FALSE
1.16E-21	0.325619824	0.935	0.497	2.93E-17	Osteo	SEC61B	FALSE	FALSE
1.53E-21	0.444698161	0.945	0.556	3.86E-17	Osteo	PFN1	FALSE	FALSE
2.10E-21	0.408236088	0.97	0.622	5.29E-17	Osteo	NPM1	FALSE	FALSE
2.43E-21	0.525986195	0.99	0.813	6.13E-17	Osteo	LGALS1	FALSE	FALSE
2.71E-21	0.275606681	0.64	0.253	6.85E-17	Osteo	SELENOP	FALSE	FALSE
3.61E-21	0.355059341	0.95	0.52	9.10E-17	Osteo	FOSB	TRUE	FALSE
5.25E-21	0.312184963	0.905	0.425	1.33E-16	Osteo	PPP1R14B	FALSE	FALSE
5.96E-21	0.425128208	0.99	0.735	1.50E-16	Osteo	MYL6	FALSE	FALSE
6.49E-21	0.289880607	0.5	0.179	1.64E-16	Osteo	FAT1	FALSE	TRUE
9.20E-21	0.324143797	0.96	0.501	2.32E-16	Osteo	LMNA	FALSE	FALSE
9.94E-21	0.258332635	0.31	0.077	2.51E-16	Osteo	CCDC102B	FALSE	FALSE
2.97E-20	0.274066514	0.84	0.415	7.50E-16	Osteo	HNRNPD	FALSE	FALSE
3.17E-20	0.382453158	0.975	0.631	7.99E-16	Osteo	YBX1	TRUE	FALSE
3.74E-20	0.259448376	0.9	0.466	9.43E-16	Osteo	SNRPD2	FALSE	FALSE
4.09E-20	0.509602155	0.885	0.46	1.03E-15	Osteo	COL6A3	FALSE	FALSE
4.74E-20	0.595386098	1	0.921	1.19E-15	Osteo	TMSB10	FALSE	FALSE
7.33E-20	0.260457603	0.745	0.324	1.85E-15	Osteo	RHEB	FALSE	FALSE
7.65E-20	0.392560081	0.925	0.488	1.93E-15	Osteo	NCL	FALSE	FALSE
9.37E-20	0.27606222	0.925	0.498	2.36E-15	Osteo	FUS	FALSE	FALSE
1.09E-19	0.272755441	0.435	0.147	2.74E-15	Osteo	ADAM12	FALSE	TRUE
1.11E-19	0.52378518	0.96	0.882	2.79E-15	Osteo	MT-ATP6	FALSE	FALSE
1.47E-19	0.31960878	0.77	0.35	3.71E-15	Osteo	NUCB2	FALSE	FALSE
7.30E-19	0.396412298	0.835	0.433	1.84E-14	Osteo	CYR61	FALSE	FALSE
1.55E-18	0.278105656	0.7	0.299	3.90E-14	Osteo	P4HA1	FALSE	FALSE
3.47E-18	0.383848491	0.895	0.521	8.75E-14	Osteo	ZFP36L1	FALSE	FALSE
5.66E-18	0.54354618	0.935	0.774	1.43E-13	Osteo	MT-RNR1	FALSE	FALSE
6.31E-18	0.295221199	0.93	0.495	1.59E-13	Osteo	HNRNPA3	FALSE	FALSE
7.91E-18	0.387781232	0.97	0.604	2.00E-13	Osteo	UBB	TRUE	FALSE
9.48E-18	0.482143194	0.955	0.635	2.39E-13	Osteo	JUN	TRUE	FALSE
1.19E-17	0.275143119	0.92	0.507	3.01E-13	Osteo	HINT1	FALSE	FALSE
1.40E-17	0.268315784	0.865	0.451	3.53E-13	Osteo	MT-ND4L	FALSE	FALSE
1.78E-17	0.434400945	0.9	0.468	4.49E-13	Osteo	AEBP1	TRUE	FALSE
3.92E-17	0.516938899	0.93	0.718	9.90E-13	Osteo	MT-ND1	FALSE	FALSE
7.15E-17	0.254171243	0.825	0.407	1.80E-12	Osteo	VMP1	FALSE	FALSE
1.31E-16	0.56273366	0.43	0.165	3.30E-12	Osteo	COL11A1	FALSE	FALSE
2.39E-16	0.287234665	0.97	0.518	6.03E-12	Osteo	EMP3	FALSE	TRUE
2.58E-16	0.274060406	0.965	0.603	6.50E-12	Osteo	H3F3A	FALSE	FALSE
7.34E-16	0.622900264	0.715	0.37	1.85E-11	Osteo	DNAJB1	FALSE	FALSE
9.57E-16	0.315500385	0.99	0.921	2.41E-11	Osteo	RPL28	FALSE	FALSE
1.74E-15	0.338006643	0.875	0.513	4.40E-11	Osteo	C11orf96	FALSE	FALSE
1.78E-15	0.429216262	0.95	0.799	4.49E-11	Osteo	MT-CYB	FALSE	FALSE
2.46E-15	0.396074448	0.935	0.535	6.20E-11	Osteo	CD99	FALSE	FALSE
1.71E-14	0.475134595	0.98	0.753	4.32E-10	Osteo	HSP90AA1	FALSE	FALSE
2.07E-14	0.325639845	0.915	0.51	5.22E-10	Osteo	HNRNPU	FALSE	FALSE
1.24E-13	0.353474289	0.995	0.905	3.13E-09	Osteo	RPS2	FALSE	FALSE
1.44E-13	0.27755787	0.965	0.585	3.64E-09	Osteo	DYNLL1	FALSE	FALSE
1.47E-13	0.346292296	0.98	0.776	3.72E-09	Osteo	ACTG1	FALSE	FALSE
1.96E-13	0.428770327	0.97	0.942	4.95E-09	Osteo	MT-RNR2	FALSE	FALSE
2.00E-13	0.323231622	0.57	0.251	5.03E-09	Osteo	SERPINF1	FALSE	FALSE
3.47E-13	0.271000928	1	0.929	8.75E-09	Osteo	RPS24	FALSE	FALSE
4.81E-13	0.326509725	0.95	0.602	1.21E-08	Osteo	NUCKS1	FALSE	FALSE
7.37E-13	0.385555882	0.515	0.253	1.86E-08	Osteo	CPE	FALSE	FALSE
7.95E-13	0.347983822	0.84	0.483	2.01E-08	Osteo	ID3	FALSE	FALSE
9.63E-13	0.26766049	1	0.912	2.43E-08	Osteo	RPLP2	FALSE	FALSE

1.16E-12	0.405620273	0.995	0.71	2.92E-08	Osteo	HMGB1	TRUE	FALSE
2.41E-12	0.298735285	0.955	0.576	6.07E-08	Osteo	CALR	FALSE	FALSE
6.05E-12	0.416379015	0.955	0.799	1.53E-07	Osteo	MT-ND2	FALSE	FALSE
6.40E-12	0.260931534	0.285	0.096	1.61E-07	Osteo	DNAL1	FALSE	FALSE
8.15E-12	0.282055884	0.98	0.781	2.06E-07	Osteo	RPL27A	FALSE	FALSE
5.32E-11	0.293248302	0.99	0.906	1.34E-06	Osteo	RPL13A	FALSE	FALSE
5.90E-11	0.262766161	0.99	0.917	1.49E-06	Osteo	RPS19	FALSE	FALSE
6.49E-10	0.274430887	0.96	0.62	1.64E-05	Osteo	HSP90B1	FALSE	FALSE
1.24E-09	0.263483451	1	0.893	3.14E-05	Osteo	ACTB	FALSE	FALSE
1.10E-07	0.523973736	0.955	0.671	0.002767705	Osteo	FN1	FALSE	FALSE
8.03E-07	0.283027812	0.985	0.878	0.020258087	Osteo	TMSB4X	FALSE	FALSE
2.66E-207	2.925165201	0.783	0.011	6.72E-203	Neu1	NRXN1	FALSE	TRUE
1.19E-140	2.833535	0.867	0.121	3.00E-136	Neu1	CDH19	FALSE	TRUE
1.05E-130	3.028028106	0.8	0.098	2.65E-126	Neu1	ITGB8	FALSE	TRUE
8.62E-118	2.051310706	0.622	0.038	2.17E-113	Neu1	PLP1	FALSE	TRUE
1.51E-114	2.221124492	0.794	0.121	3.81E-110	Neu1	GPM6B	FALSE	TRUE
1.92E-107	1.577715684	0.422	0.005	4.85E-103	Neu1	SOX2	TRUE	FALSE
2.95E-96	1.5845963	0.439	0.015	7.45E-92	Neu1	ERBB3	FALSE	TRUE
7.30E-92	1.87778271	0.55	0.049	1.84E-87	Neu1	SEMA3B	FALSE	FALSE
1.95E-86	1.841165398	0.533	0.052	4.93E-82	Neu1	GPR155	FALSE	TRUE
6.82E-85	1.370104545	0.367	0.009	1.72E-80	Neu1	ADGRB3	FALSE	FALSE
2.33E-84	1.946731708	0.628	0.095	5.88E-80	Neu1	ANK3	FALSE	FALSE
4.86E-70	1.340995604	0.261	0.001	1.23E-65	Neu1	XKR4	FALSE	FALSE
2.37E-68	1.547725031	0.539	0.08	5.97E-64	Neu1	CADM1	FALSE	TRUE
9.21E-68	1.260819904	0.339	0.016	2.32E-63	Neu1	ASPA	FALSE	FALSE
1.67E-66	1.595727352	0.444	0.047	4.22E-62	Neu1	HSPA12A	FALSE	FALSE
2.10E-65	1.234876172	0.272	0.005	5.29E-61	Neu1	SCN9A	FALSE	TRUE
1.55E-64	1.266856031	0.283	0.008	3.91E-60	Neu1	IQGAP2	FALSE	FALSE
5.11E-62	1.89958372	0.656	0.174	1.29E-57	Neu1	AHR	TRUE	FALSE
3.85E-61	1.366977984	0.339	0.021	9.71E-57	Neu1	AATK	FALSE	FALSE
3.19E-58	1.340712852	0.317	0.019	8.05E-54	Neu1	PTPRZ1	FALSE	TRUE
7.85E-58	1.130026289	0.278	0.011	1.98E-53	Neu1	SORCS1	FALSE	TRUE
2.09E-57	1.194720706	0.306	0.018	5.27E-53	Neu1	C2orf88	FALSE	FALSE
7.66E-57	1.49551866	0.411	0.049	1.93E-52	Neu1	NGFR	FALSE	TRUE
2.00E-56	1.107184837	0.211	0.001	5.04E-52	Neu1	GALR1	FALSE	TRUE
9.19E-55	1.197186404	0.261	0.01	2.32E-50	Neu1	SOX2-OT	FALSE	FALSE
4.87E-52	1.72636838	0.467	0.079	1.23E-47	Neu1	LGI4	FALSE	FALSE
7.83E-51	1.132326518	0.283	0.018	1.98E-46	Neu1	NRXN3	FALSE	TRUE
1.14E-50	2.198673164	0.456	0.078	2.87E-46	Neu1	FGL2	FALSE	FALSE
1.08E-47	1.111701238	0.222	0.008	2.72E-43	Neu1	CADM2	FALSE	TRUE
2.67E-47	1.225979719	0.283	0.02	6.73E-43	Neu1	COL28A1	FALSE	FALSE
4.78E-46	1.320402999	0.417	0.069	1.21E-41	Neu1	SORBS1	FALSE	FALSE
5.99E-46	1.125911697	0.289	0.023	1.51E-41	Neu1	RELN	FALSE	FALSE
1.86E-44	1.788160493	0.411	0.07	4.71E-40	Neu1	HLA-DRA	FALSE	TRUE
4.41E-43	1.19912969	0.239	0.015	1.11E-38	Neu1	COBL	FALSE	FALSE
8.27E-42	1.637484447	0.361	0.057	2.09E-37	Neu1	GAP43	FALSE	FALSE
3.20E-38	1.179728649	0.289	0.035	8.07E-34	Neu1	KCNMB4	FALSE	TRUE
4.15E-38	1.140613365	0.311	0.041	1.05E-33	Neu1	ITGB4	FALSE	TRUE
3.30E-37	1.376834766	0.722	0.394	8.33E-33	Neu1	ZEB2	TRUE	FALSE
8.16E-37	1.191247879	0.317	0.048	2.06E-32	Neu1	TMOD2	FALSE	FALSE
9.84E-36	1.871391896	0.544	0.183	2.48E-31	Neu1	CD74	FALSE	TRUE
1.15E-33	1.117206632	0.261	0.033	2.90E-29	Neu1	MYOT	FALSE	FALSE
3.10E-33	1.286056375	0.467	0.141	7.82E-29	Neu1	GAS7	FALSE	FALSE
3.97E-33	1.360602336	0.328	0.057	1.00E-28	Neu1	HLA-DRB1	FALSE	TRUE
1.95E-31	1.314184237	0.378	0.091	4.92E-27	Neu1	COL8A1	FALSE	FALSE
2.55E-31	1.354367922	0.367	0.082	6.43E-27	Neu1	ITGA6	FALSE	TRUE
1.94E-30	0.965255263	0.278	0.043	4.90E-26	Neu1	DMD	FALSE	FALSE
5.32E-30	0.811878911	0.211	0.022	1.34E-25	Neu1	CADM4	FALSE	TRUE
2.09E-28	1.273443648	0.761	0.551	5.28E-24	Neu1	DST	FALSE	FALSE
1.79E-27	1.161254154	0.294	0.06	4.52E-23	Neu1	CNKSR3	FALSE	FALSE
1.65E-26	1.153832453	0.617	0.294	4.17E-22	Neu1	S100B	FALSE	FALSE
1.70E-26	1.220747394	0.489	0.199	4.29E-22	Neu1	VWA1	FALSE	FALSE
1.93E-25	0.820842299	0.711	0.502	4.88E-21	Neu1	PMP22	FALSE	FALSE
2.20E-25	1.379368535	0.461	0.184	5.56E-21	Neu1	EHBP1	FALSE	FALSE
4.14E-25	1.192291448	0.389	0.118	1.04E-20	Neu1	NRN1	FALSE	TRUE
4.96E-25	1.239660687	0.628	0.393	1.25E-20	Neu1	RDX	FALSE	FALSE
1.21E-24	1.220536364	0.339	0.092	3.05E-20	Neu1	SOX6	TRUE	FALSE
2.09E-24	1.084259291	0.267	0.054	5.27E-20	Neu1	FIGN	FALSE	FALSE
4.41E-24	1.312475798	0.428	0.15	1.11E-19	Neu1	NDRG2	FALSE	FALSE
1.19E-23	0.81886344	0.811	0.608	2.99E-19	Neu1	CD9	FALSE	TRUE
1.72E-23	1.195470335	0.328	0.09	4.35E-19	Neu1	DOCK5	FALSE	FALSE
4.66E-23	1.238956122	0.372	0.118	1.18E-18	Neu1	MATN2	FALSE	FALSE
5.32E-23	0.979122915	0.311	0.081	1.34E-18	Neu1	ZNRF2	FALSE	FALSE
4.53E-22	0.723761856	0.206	0.032	1.14E-17	Neu1	SBSPON	FALSE	FALSE
1.15E-21	1.082400973	0.383	0.13	2.91E-17	Neu1	RARRES2	FALSE	FALSE
3.12E-21	1.117658564	0.656	0.472	7.87E-17	Neu1	CLIC4	FALSE	FALSE
3.18E-21	0.875224112	0.789	0.633	8.03E-17	Neu1	CRYAB	FALSE	FALSE
1.95E-20	1.044349139	0.433	0.175	4.91E-16	Neu1	SYNE2	FALSE	FALSE
2.21E-20	1.156474822	0.3	0.085	5.57E-16	Neu1	KHDRBS3	FALSE	FALSE
5.87E-20	1.357687998	0.456	0.216	1.48E-15	Neu1	CTSC	FALSE	FALSE
6.14E-20	0.878429589	0.739	0.571	1.55E-15	Neu1	DDX17	FALSE	FALSE
1.27E-19	1.053802621	0.256	0.06	3.21E-15	Neu1	EDNRB	FALSE	TRUE
3.91E-19	1.076454357	0.389	0.155	9.87E-15	Neu1	STARD13	FALSE	FALSE
5.91E-19	0.997901067	0.628	0.433	1.49E-14	Neu1	SPTBN1	FALSE	FALSE
7.43E-19	0.966300149	0.294	0.09	1.87E-14	Neu1	GNG2	FALSE	FALSE
1.24E-18	1.003193992	0.383	0.146	3.13E-14	Neu1	CELF2	FALSE	FALSE

2.25E-18	1.038395603	0.294	0.088	5.69E-14	Neu1	SAMHD1	FALSE	FALSE
6.04E-18	1.235112047	0.222	0.048	1.52E-13	Neu1	ANGPTL7	FALSE	FALSE
8.68E-18	1.002333981	0.322	0.114	2.19E-13	Neu1	C1orf198	FALSE	FALSE
1.05E-17	1.0346532	0.622	0.464	2.65E-13	Neu1	QKI	FALSE	FALSE
4.38E-17	0.834119714	0.222	0.052	1.11E-12	Neu1	GAS2L3	FALSE	FALSE
4.90E-17	0.766804478	0.7	0.451	1.24E-12	Neu1	TIMP3	FALSE	FALSE
1.88E-16	0.906143788	0.317	0.108	4.75E-12	Neu1	PLAT	FALSE	FALSE
3.78E-16	0.981908272	0.306	0.109	9.55E-12	Neu1	MICALL2	FALSE	FALSE
4.22E-16	0.976215824	0.594	0.445	1.07E-11	Neu1	CNN3	FALSE	FALSE
5.52E-16	1.194546366	0.378	0.174	1.39E-11	Neu1	DAG1	FALSE	TRUE
9.62E-16	1.161577392	0.467	0.258	2.43E-11	Neu1	LTBP4	FALSE	FALSE
2.07E-15	1.011453725	0.261	0.081	5.22E-11	Neu1	PCDH9	FALSE	TRUE
6.21E-15	0.818603512	0.206	0.051	1.57E-10	Neu1	SHC4	FALSE	FALSE
9.85E-15	0.907025482	0.272	0.094	2.49E-10	Neu1	SIPA1L2	FALSE	FALSE
1.09E-14	1.140686494	0.389	0.191	2.74E-10	Neu1	AP1S2	FALSE	FALSE
1.55E-14	1.518296049	0.228	0.066	3.90E-10	Neu1	BIRC3	FALSE	FALSE
2.62E-14	0.466351832	0.917	0.857	6.61E-10	Neu1	H3F3B	FALSE	FALSE
1.57E-13	0.78583253	0.206	0.058	3.95E-09	Neu1	NCAM1	FALSE	TRUE
2.70E-13	0.849817053	0.228	0.072	6.82E-09	Neu1	SPATA13	FALSE	FALSE
6.69E-13	0.850077836	0.228	0.073	1.69E-08	Neu1	SDC3	FALSE	FALSE
1.11E-12	1.117592371	0.344	0.167	2.80E-08	Neu1	ZBTB44	TRUE	FALSE
1.96E-12	0.879333759	0.594	0.461	4.95E-08	Neu1	TUBA1A	FALSE	FALSE
2.51E-12	0.845893694	0.283	0.112	6.34E-08	Neu1	FAM129A	FALSE	FALSE
8.60E-12	0.880272417	0.339	0.162	2.17E-07	Neu1	SEMA3C	FALSE	FALSE
1.11E-11	0.812484318	0.617	0.506	2.79E-07	Neu1	WSB1	FALSE	FALSE
1.18E-11	0.875949797	0.222	0.076	2.97E-07	Neu1	MEGF9	FALSE	TRUE
5.38E-11	0.588576052	0.644	0.497	1.36E-06	Neu1	MARCKS	FALSE	FALSE
1.82E-10	1.067084069	0.444	0.306	4.59E-06	Neu1	ITGAV	FALSE	TRUE
5.58E-10	0.712437472	0.517	0.353	1.41E-05	Neu1	NR2F2	TRUE	FALSE
9.28E-10	0.810377235	0.45	0.299	2.34E-05	Neu1	COL18A1	FALSE	FALSE
1.08E-09	0.292983589	0.961	0.93	2.73E-05	Neu1	B2M	FALSE	FALSE
1.38E-09	0.808848033	0.639	0.57	3.47E-05	Neu1	BTG1	FALSE	FALSE
1.73E-09	0.734574465	0.211	0.08	4.37E-05	Neu1	DKK3	FALSE	FALSE
2.07E-09	0.594043157	0.261	0.113	5.23E-05	Neu1	PLA2G16	FALSE	FALSE
2.39E-09	1.031921707	0.461	0.346	6.03E-05	Neu1	PDLIM4	FALSE	FALSE
2.48E-09	0.80314876	0.272	0.122	6.25E-05	Neu1	MCAM	FALSE	TRUE
2.73E-09	0.890465877	0.389	0.24	6.88E-05	Neu1	MAF	TRUE	FALSE
3.33E-09	0.937892831	0.522	0.424	8.41E-05	Neu1	ZBTB20	TRUE	FALSE
6.60E-09	0.87621705	0.394	0.253	0.000166522	Neu1	PIK3R1	FALSE	FALSE
6.79E-09	0.816209733	0.456	0.323	0.000171351	Neu1	TGFBR2	FALSE	TRUE
7.71E-09	0.741225707	0.228	0.096	0.000194617	Neu1	FRMD4A	FALSE	FALSE
1.42E-08	0.771772721	0.511	0.415	0.000358916	Neu1	EMP2	FALSE	TRUE
1.83E-08	0.772147332	0.261	0.128	0.00046197	Neu1	SCCPDH	FALSE	FALSE
4.72E-08	0.877157785	0.567	0.475	0.001189891	Neu1	PMEPA1	FALSE	TRUE
5.47E-08	0.809641801	0.478	0.393	0.001380333	Neu1	CTNNA1	FALSE	FALSE
9.36E-08	0.832331956	0.289	0.157	0.002360733	Neu1	RETREG1	FALSE	FALSE
1.60E-07	0.547107557	0.65	0.623	0.004043328	Neu1	SON	TRUE	FALSE
1.88E-07	0.83350093	0.4	0.285	0.004740138	Neu1	VPS13C	FALSE	FALSE
4.30E-07	1.131556576	0.294	0.174	0.010861094	Neu1	SESN3	FALSE	FALSE
5.14E-07	0.929198377	0.294	0.176	0.012957967	Neu1	GPC1	FALSE	TRUE
7.74E-07	0.833484474	0.367	0.253	0.019539784	Neu1	ADD3	FALSE	FALSE
8.58E-07	0.913129673	0.428	0.339	0.021651368	Neu1	PRNP	TRUE	TRUE
1.54E-06	0.534849114	0.661	0.652	0.038870416	Neu1	PSAP	FALSE	FALSE
2.56E-253	2.232530276	0.92	0.001	6.47E-249	Neu2	NCMAP	FALSE	TRUE
1.35E-210	2.787834274	0.96	0.007	3.41E-206	Neu2	MLIP	FALSE	FALSE
1.47E-208	2.060025668	0.96	0.007	3.71E-204	Neu2	AZGP1	FALSE	FALSE
4.10E-205	2.846295361	1	0.009	1.03E-200	Neu2	PRX	FALSE	FALSE
1.05E-191	2.113592277	0.8	0.004	2.65E-187	Neu2	SFRP5	FALSE	FALSE
1.38E-170	1.772199475	0.8	0.006	3.48E-166	Neu2	TMPRSS5	FALSE	TRUE
4.17E-170	1.484039553	0.64	0.001	1.05E-165	Neu2	KIF19	FALSE	FALSE
1.31E-167	1.58694701	0.56	0	3.31E-163	Neu2	DRP2	FALSE	FALSE
1.02E-164	1.597761224	0.68	0.003	2.56E-160	Neu2	BCAS1	FALSE	FALSE
2.93E-158	1.724440182	0.6	0.001	7.40E-154	Neu2	CLDN19	FALSE	TRUE
2.61E-144	1.522091393	0.52	0.001	6.58E-140	Neu2	SLC25A48	FALSE	FALSE
4.46E-126	1.374683385	0.52	0.002	1.13E-121	Neu2	GJC3	FALSE	TRUE
3.25E-125	1.83330728	0.76	0.011	8.21E-121	Neu2	MT3	FALSE	FALSE
6.05E-120	0.938968003	0.4	0	1.53E-115	Neu2	CDH1	FALSE	TRUE
1.66E-114	1.157091361	0.48	0.002	4.18E-110	Neu2	PEX5L	FALSE	FALSE
2.62E-111	1.063779621	0.44	0.001	6.62E-107	Neu2	KIF1A	FALSE	FALSE
1.55E-104	1.6561145	0.76	0.016	3.91E-100	Neu2	PRRG4	FALSE	FALSE
3.32E-96	0.938957878	0.32	0	8.39E-92	Neu2	GRIA2	FALSE	TRUE
5.04E-96	1.388282195	0.52	0.006	1.27E-91	Neu2	KCNK12	FALSE	FALSE
8.46E-95	1.003718789	0.48	0.004	2.14E-90	Neu2	FA2H	FALSE	FALSE
2.39E-91	2.21097356	0.92	0.035	6.04E-87	Neu2	GATM	FALSE	FALSE
4.28E-88	0.831669112	0.36	0.001	1.08E-83	Neu2	CTNNA3	FALSE	FALSE
1.89E-85	0.873080977	0.4	0.003	4.78E-81	Neu2	SLC44A4	FALSE	TRUE
2.51E-85	4.17989476	1	0.051	6.34E-81	Neu2	MPZ	FALSE	TRUE
6.81E-84	2.325162093	0.92	0.04	1.72E-79	Neu2	PLLP	FALSE	FALSE
5.12E-80	1.05079297	0.4	0.004	1.29E-75	Neu2	LRRC7	FALSE	FALSE
1.94E-76	1.786492873	0.92	0.042	4.90E-72	Neu2	RELN	FALSE	FALSE
2.40E-75	1.939140804	0.88	0.039	6.06E-71	Neu2	COL28A1	FALSE	FALSE
1.59E-72	1.287625741	0.64	0.019	4.00E-68	Neu2	ARHGAP19	FALSE	FALSE
1.61E-72	0.687092198	0.24	0	4.06E-68	Neu2	SMCO3	FALSE	FALSE
5.35E-71	1.155962078	0.6	0.016	1.35E-66	Neu2	TNFRSF25	FALSE	TRUE
8.82E-70	0.847978847	0.32	0.002	2.23E-65	Neu2	PACRG	FALSE	FALSE
2.44E-67	1.31087597	0.56	0.015	6.17E-63	Neu2	ART3	FALSE	TRUE

2.83E-67	1.274576539	0.52	0.013	7.15E-63	Neu2	UGT8	FALSE	TRUE
4.05E-66	3.25177499	1	0.073	1.02E-61	Neu2	MBP	FALSE	FALSE
3.30E-65	0.855285322	0.44	0.008	8.34E-61	Neu2	ROPN1	FALSE	FALSE
3.49E-62	0.538124929	0.24	0.001	8.81E-58	Neu2	SLC6A4	FALSE	TRUE
3.65E-62	0.896959827	0.4	0.007	9.20E-58	Neu2	LAMA3	FALSE	FALSE
6.06E-62	2.230537822	0.88	0.056	1.53E-57	Neu2	GLDN	FALSE	TRUE
6.60E-60	1.189589871	0.52	0.016	1.66E-55	Neu2	PLEKHA7	FALSE	FALSE
8.50E-60	0.8001214	0.36	0.005	2.14E-55	Neu2	SV2C	FALSE	TRUE
4.83E-58	1.110508284	0.6	0.022	1.22E-53	Neu2	PPP1R9A	FALSE	FALSE
1.38E-56	1.79188211	0.88	0.059	3.48E-52	Neu2	GAS2L3	FALSE	FALSE
1.99E-54	0.634088403	0.24	0.001	5.03E-50	Neu2	NKAIN2	FALSE	TRUE
2.18E-54	1.11762643	0.52	0.018	5.49E-50	Neu2	SPTBN5	FALSE	FALSE
2.69E-54	0.973761907	0.24	0.001	6.79E-50	Neu2	AP001350.2	FALSE	FALSE
1.30E-52	1.397504633	0.68	0.034	3.29E-48	Neu2	SNCA	FALSE	FALSE
2.71E-51	1.378065505	0.8	0.049	6.83E-47	Neu2	AATK	FALSE	FALSE
5.69E-51	1.269321606	0.68	0.037	1.44E-46	Neu2	NFASC	FALSE	TRUE
1.19E-48	1.72617404	0.8	0.059	3.01E-44	Neu2	SEMA5A	FALSE	TRUE
2.41E-48	0.660603673	0.24	0.002	6.07E-44	Neu2	PPP1R1C	FALSE	FALSE
1.04E-43	1.089288898	0.36	0.01	2.64E-39	Neu2	FXYD3	FALSE	FALSE
1.04E-43	0.747353157	0.36	0.01	2.64E-39	Neu2	DUSP15	FALSE	FALSE
1.65E-42	1.920271505	0.96	0.099	4.17E-38	Neu2	PLP1	FALSE	TRUE
3.89E-42	1.584444346	0.64	0.041	9.83E-38	Neu2	POU3F1	TRUE	FALSE
1.69E-41	2.014261392	0.8	0.073	4.27E-37	Neu2	LPL	FALSE	TRUE
1.32E-39	0.482628545	0.24	0.004	3.32E-35	Neu2	CAMK2B	FALSE	FALSE
2.05E-39	1.063450603	0.6	0.036	5.18E-35	Neu2	CADM4	FALSE	TRUE
4.11E-36	0.984365983	0.52	0.03	1.04E-31	Neu2	CMTM5	FALSE	FALSE
5.05E-35	0.950497803	0.36	0.013	1.27E-30	Neu2	RASGEF1A	FALSE	FALSE
9.46E-35	0.818970749	0.32	0.01	2.39E-30	Neu2	OR7E14P	FALSE	FALSE
1.28E-34	1.384637721	0.44	0.022	3.24E-30	Neu2	CAHM	FALSE	FALSE
4.47E-33	1.130972222	0.56	0.042	1.13E-28	Neu2	SERPINB9	FALSE	FALSE
5.61E-33	1.03704119	0.6	0.045	1.42E-28	Neu2	SBSPON	FALSE	FALSE
3.84E-32	1.376199773	0.68	0.062	9.69E-28	Neu2	DMD	FALSE	FALSE
4.89E-32	1.704657494	0.88	0.114	1.23E-27	Neu2	FGL2	FALSE	FALSE
6.56E-32	1.788874059	0.84	0.117	1.66E-27	Neu2	TSC22D4	TRUE	FALSE
9.72E-31	1.115228201	0.52	0.036	2.45E-26	Neu2	CADM3	FALSE	TRUE
2.17E-30	2.042677695	0.92	0.16	5.47E-26	Neu2	CNP	FALSE	FALSE
6.92E-30	1.166848485	0.52	0.038	1.75E-25	Neu2	SOX8	TRUE	FALSE
1.16E-29	2.24661323	0.84	0.13	2.93E-25	Neu2	LIMCH1	FALSE	FALSE
3.04E-29	1.487684603	0.84	0.12	7.67E-25	Neu2	SVIP	FALSE	FALSE
5.47E-29	0.916175533	0.52	0.037	1.38E-24	Neu2	SORCS1	FALSE	TRUE
1.29E-28	1.130426171	0.76	0.087	3.27E-24	Neu2	HSPA12A	FALSE	FALSE
3.80E-28	0.75109058	0.32	0.014	9.60E-24	Neu2	CPM	FALSE	TRUE
2.01E-27	2.235160877	0.96	0.205	5.07E-23	Neu2	SECISBP2L	FALSE	FALSE
2.15E-27	1.887127211	0.76	0.111	5.44E-23	Neu2	NEK1	FALSE	FALSE
2.34E-27	1.058976346	0.44	0.028	5.89E-23	Neu2	PMP2	FALSE	FALSE
2.54E-27	1.191499526	0.6	0.061	6.41E-23	Neu2	KCTD11	FALSE	FALSE
3.88E-27	0.832766409	0.44	0.03	9.80E-23	Neu2	FRMD3	FALSE	FALSE
6.17E-27	0.961841098	0.48	0.038	1.56E-22	Neu2	IL16	FALSE	FALSE
8.44E-27	1.707508433	1	0.195	2.13E-22	Neu2	GPM6B	FALSE	TRUE
8.92E-27	2.049262282	0.88	0.16	2.25E-22	Neu2	CDKN1C	FALSE	FALSE
3.21E-26	0.762269244	0.28	0.011	8.10E-22	Neu2	AIF1L	FALSE	FALSE
5.01E-26	0.633960753	0.32	0.015	1.26E-21	Neu2	GABRA2	FALSE	TRUE
2.40E-25	1.306187132	0.76	0.102	6.05E-21	Neu2	SORBS1	FALSE	FALSE
3.91E-25	1.027055166	0.68	0.085	9.86E-21	Neu2	MEGF9	FALSE	TRUE
2.31E-24	1.273746864	0.44	0.036	5.83E-20	Neu2	MAPK8IP1	FALSE	FALSE
5.36E-24	0.543898641	0.24	0.009	1.35E-19	Neu2	ZNF185	FALSE	FALSE
5.39E-24	0.672092474	0.28	0.013	1.36E-19	Neu2	NRIP2	FALSE	FALSE
1.15E-23	0.945332093	0.48	0.043	2.89E-19	Neu2	CASKIN2	FALSE	FALSE
2.47E-23	1.474279994	0.72	0.111	6.23E-19	Neu2	SRGAP2B	FALSE	FALSE
3.31E-23	1.472004603	0.76	0.121	8.35E-19	Neu2	PLA2G16	FALSE	FALSE
6.33E-23	0.768939183	0.28	0.013	1.60E-18	Neu2	TRPM3	FALSE	FALSE
7.58E-22	1.193239742	0.64	0.093	1.91E-17	Neu2	SLC20A2	FALSE	TRUE
1.19E-21	1.339355241	0.6	0.078	2.99E-17	Neu2	OLFML2A	FALSE	FALSE
2.05E-21	0.963087008	0.36	0.026	5.18E-17	Neu2	FAM19A5	FALSE	FALSE
2.16E-21	0.650716562	0.32	0.02	5.44E-17	Neu2	GPRC5B	FALSE	TRUE
2.72E-20	1.005965562	0.84	0.152	6.87E-16	Neu2	ANK3	FALSE	FALSE
3.40E-20	0.870750069	0.44	0.042	8.58E-16	Neu2	LIMS2	FALSE	FALSE
3.55E-20	0.850105896	0.72	0.108	8.97E-16	Neu2	ITGA6	FALSE	TRUE
4.83E-20	0.905256279	0.72	0.104	1.22E-15	Neu2	SEMA3B	FALSE	FALSE
1.00E-19	0.856385067	0.32	0.022	2.53E-15	Neu2	AURKB	FALSE	FALSE
1.43E-19	3.164772317	1	0.52	3.62E-15	Neu2	PMP22	FALSE	FALSE
1.48E-19	1.317071859	0.8	0.184	3.73E-15	Neu2	TMEM98	FALSE	FALSE
1.67E-19	0.758648363	0.4	0.037	4.21E-15	Neu2	FGF1	FALSE	FALSE
2.65E-19	0.975120695	0.48	0.054	6.69E-15	Neu2	FBLN5	FALSE	FALSE
2.68E-19	1.137137134	0.64	0.108	6.75E-15	Neu2	SGCD	FALSE	TRUE
3.50E-19	0.683206081	0.4	0.035	8.82E-15	Neu2	AFAP1L2	FALSE	FALSE
1.11E-18	0.833392783	0.32	0.024	2.81E-14	Neu2	ANKRD29	FALSE	FALSE
2.27E-18	0.685895505	0.32	0.024	5.74E-14	Neu2	LRRC4C	FALSE	TRUE
2.73E-18	0.815881276	0.28	0.019	6.90E-14	Neu2	GLDC	FALSE	FALSE
3.35E-18	1.652218584	0.8	0.18	8.46E-14	Neu2	PLEKHA4	FALSE	FALSE
4.16E-18	0.763213503	0.24	0.013	1.05E-13	Neu2	CYP19A1	FALSE	FALSE
8.35E-18	0.746027601	0.32	0.025	2.11E-13	Neu2	C5orf30	FALSE	FALSE
1.06E-17	0.814096863	0.72	0.119	2.67E-13	Neu2	LGI4	FALSE	FALSE
3.12E-17	1.353472511	0.48	0.064	7.87E-13	Neu2	SCRN1	FALSE	FALSE
3.56E-17	0.590845021	0.24	0.014	8.99E-13	Neu2	TRPV1	FALSE	FALSE
5.37E-17	1.850596952	0.92	0.326	1.36E-12	Neu2	S100B	FALSE	FALSE

7.50E-17	1.303216995	0.88	0.248	1.89E-12	Neu2	MAF	TRUE	FALSE
7.60E-17	1.102196273	0.28	0.02	1.92E-12	Neu2	PLEKHB1	FALSE	FALSE
1.16E-16	1.031193115	0.48	0.065	2.93E-12	Neu2	TRAK1	FALSE	FALSE
3.64E-16	1.059316244	0.48	0.064	9.19E-12	Neu2	SHC4	FALSE	FALSE
4.31E-16	0.924687526	0.36	0.036	1.09E-11	Neu2	CPXM2	FALSE	FALSE
5.74E-16	1.258174059	0.68	0.145	1.45E-11	Neu2	SRGAP2C	FALSE	FALSE
2.04E-15	1.344032468	0.84	0.269	5.15E-11	Neu2	TJP1	FALSE	FALSE
2.27E-15	1.115727905	0.76	0.191	5.73E-11	Neu2	DAG1	FALSE	TRUE
5.07E-15	1.636512973	0.8	0.248	1.28E-10	Neu2	TAX1BP3	FALSE	FALSE
7.47E-15	0.780041399	0.44	0.06	1.88E-10	Neu2	NAV3	FALSE	FALSE
8.87E-15	1.468313029	1	0.647	2.24E-10	Neu2	CRYAB	FALSE	FALSE
2.04E-14	1.494827672	0.96	0.476	5.15E-10	Neu2	QKI	FALSE	FALSE
2.20E-14	1.305672471	0.64	0.145	5.55E-10	Neu2	SRGAP2	FALSE	FALSE
2.58E-14	1.56730926	0.84	0.315	6.51E-10	Neu2	RHOBTB3	FALSE	FALSE
4.51E-14	1.041071168	0.36	0.043	1.14E-09	Neu2	SPTLC3	FALSE	FALSE
9.33E-14	1.09110558	0.44	0.065	2.35E-09	Neu2	CAB39L	FALSE	FALSE
1.98E-13	1.284490855	1	0.628	5.01E-09	Neu2	CD9	FALSE	TRUE
2.48E-13	1.03386063	0.4	0.056	6.26E-09	Neu2	SGK3	FALSE	FALSE
2.96E-13	1.370254046	0.6	0.14	7.48E-09	Neu2	LRRC8A	FALSE	FALSE
3.98E-13	1.333902599	0.92	0.43	1.01E-08	Neu2	MYL9	FALSE	FALSE
4.89E-13	1.129271752	0.6	0.132	1.23E-08	Neu2	SLC7A2	FALSE	TRUE
5.89E-13	1.264027448	0.96	0.635	1.49E-08	Neu2	DYNLL1	FALSE	FALSE
6.04E-13	0.993930993	0.6	0.132	1.52E-08	Neu2	BICD1	FALSE	FALSE
7.66E-13	0.760806362	0.32	0.037	1.93E-08	Neu2	SHROOM1	FALSE	FALSE
7.95E-13	1.228017337	0.92	0.419	2.01E-08	Neu2	EMP2	FALSE	TRUE
9.85E-13	0.625384279	0.32	0.036	2.49E-08	Neu2	TSPAN15	FALSE	TRUE
1.16E-12	0.692294376	0.28	0.029	2.94E-08	Neu2	MME	FALSE	TRUE
1.28E-12	0.885679508	0.48	0.085	3.22E-08	Neu2	SEMA4C	FALSE	TRUE
1.41E-12	1.309103128	0.88	0.299	3.56E-08	Neu2	SELENOP	FALSE	FALSE
1.56E-12	0.744654763	0.44	0.071	3.93E-08	Neu2	SORT1	FALSE	TRUE
2.30E-12	0.892484426	0.4	0.062	5.81E-08	Neu2	CACNA1A	FALSE	FALSE
2.91E-12	0.614509017	0.24	0.022	7.35E-08	Neu2	SHROOM3	FALSE	FALSE
3.17E-12	1.024551293	0.44	0.077	8.01E-08	Neu2	KAZN	FALSE	FALSE
3.38E-12	0.707325794	0.32	0.036	8.52E-08	Neu2	THSD7A	FALSE	TRUE
3.40E-12	0.635253956	0.28	0.03	8.57E-08	Neu2	TMEM117	FALSE	FALSE
4.04E-12	0.811844301	0.32	0.039	1.02E-07	Neu2	FLRT3	FALSE	TRUE
4.31E-12	0.799219805	0.44	0.076	1.09E-07	Neu2	NEDD9	FALSE	FALSE
4.71E-12	0.841416109	0.48	0.091	1.19E-07	Neu2	MOSPD2	FALSE	FALSE
5.51E-12	1.091498843	1	0.671	1.39E-07	Neu2	CALM2	FALSE	FALSE
1.67E-11	1.297955587	0.72	0.23	4.22E-07	Neu2	ITM2C	FALSE	TRUE
1.70E-11	0.713399056	0.44	0.07	4.29E-07	Neu2	ITGB4	FALSE	TRUE
2.06E-11	1.010063082	0.84	0.334	5.19E-07	Neu2	PTEN	FALSE	FALSE
2.07E-11	0.464203576	0.28	0.031	5.21E-07	Neu2	JUP	FALSE	FALSE
2.29E-11	1.381934771	0.88	0.472	5.77E-07	Neu2	DYNLRB1	FALSE	FALSE
2.39E-11	0.846090307	0.4	0.065	6.02E-07	Neu2	RTKN	FALSE	FALSE
2.72E-11	0.49357336	0.24	0.023	6.86E-07	Neu2	PIEZO2	FALSE	TRUE
3.45E-11	0.512745602	0.32	0.041	8.69E-07	Neu2	BMP8B	FALSE	FALSE
3.83E-11	0.821193433	0.56	0.131	9.67E-07	Neu2	PTPRE	FALSE	FALSE
6.58E-11	0.747091563	0.4	0.069	1.66E-06	Neu2	BBS2	FALSE	FALSE
8.14E-11	1.095782315	0.72	0.229	2.05E-06	Neu2	NR4A2	TRUE	FALSE
9.16E-11	0.516755778	0.28	0.033	2.31E-06	Neu2	FAXDC2	FALSE	FALSE
1.02E-10	0.792610425	0.44	0.081	2.56E-06	Neu2	FST	FALSE	FALSE
1.44E-10	0.783292109	0.56	0.135	3.64E-06	Neu2	GULP1	FALSE	FALSE
1.91E-10	0.940958316	0.44	0.089	4.81E-06	Neu2	PPM1F	FALSE	FALSE
2.10E-10	0.737490161	0.4	0.065	5.30E-06	Neu2	ERBB3	FALSE	TRUE
2.20E-10	0.663908872	0.4	0.069	5.54E-06	Neu2	STARD9	FALSE	FALSE
2.27E-10	0.596422266	0.28	0.034	5.74E-06	Neu2	EFNB2	FALSE	TRUE
2.43E-10	1.142344375	0.72	0.256	6.12E-06	Neu2	SLC5A3	FALSE	TRUE
2.65E-10	0.562016208	0.24	0.026	6.68E-06	Neu2	ZBED3	TRUE	FALSE
2.83E-10	1.186322175	0.92	0.532	7.14E-06	Neu2	PEBP1	FALSE	FALSE
2.83E-10	0.888023874	1	0.701	7.15E-06	Neu2	SPARC	FALSE	FALSE
3.20E-10	1.034565809	0.6	0.173	8.06E-06	Neu2	MAP1LC3A	FALSE	FALSE
3.44E-10	1.136003528	0.88	0.519	8.69E-06	Neu2	SELENOW	FALSE	FALSE
3.65E-10	0.911139648	0.44	0.092	9.21E-06	Neu2	GNAI1	FALSE	FALSE
4.24E-10	0.643225332	0.28	0.036	1.07E-05	Neu2	MAGI1	FALSE	FALSE
4.27E-10	0.844887937	1	0.918	1.08E-05	Neu2	FTH1	FALSE	FALSE
7.85E-10	0.964305919	0.84	0.362	1.98E-05	Neu2	EPB41L2	FALSE	FALSE
9.82E-10	0.952437098	0.68	0.219	2.48E-05	Neu2	FXYD6	FALSE	FALSE
1.02E-09	0.984382993	0.48	0.119	2.58E-05	Neu2	FUT8	FALSE	FALSE
1.18E-09	0.821304547	0.32	0.05	2.97E-05	Neu2	LHPP	FALSE	FALSE
1.21E-09	0.729179684	0.32	0.05	3.05E-05	Neu2	COQ8B	FALSE	FALSE
1.22E-09	0.727075895	0.36	0.065	3.07E-05	Neu2	IQCK	FALSE	FALSE
1.28E-09	1.164831946	0.96	0.586	3.23E-05	Neu2	DDX17	FALSE	FALSE
1.34E-09	0.808711998	0.32	0.048	3.38E-05	Neu2	CXCL14	FALSE	FALSE
1.75E-09	0.922358821	0.6	0.178	4.43E-05	Neu2	SSFA2	FALSE	FALSE
1.83E-09	0.953374597	0.8	0.29	4.62E-05	Neu2	MIA	FALSE	FALSE
2.02E-09	1.308146369	0.72	0.304	5.10E-05	Neu2	CD47	FALSE	TRUE
2.47E-09	0.995458033	0.6	0.189	6.24E-05	Neu2	OXR1	FALSE	FALSE
2.56E-09	0.918616484	0.44	0.1	6.46E-05	Neu2	RAP1GDS1	FALSE	FALSE
2.79E-09	0.943676345	0.6	0.176	7.03E-05	Neu2	GAS7	FALSE	FALSE
3.57E-09	1.038769533	0.72	0.282	9.01E-05	Neu2	GLUL	FALSE	FALSE
4.17E-09	1.108814708	0.56	0.169	0.000105203	Neu2	FRYL	FALSE	FALSE
4.66E-09	0.598269626	0.28	0.04	0.000117694	Neu2	ELMO1	FALSE	FALSE
4.77E-09	0.734243064	1	0.967	0.000120399	Neu2	VIM	FALSE	FALSE
5.50E-09	0.573522643	0.24	0.03	0.000138756	Neu2	ELN	FALSE	FALSE
5.83E-09	1.02770818	0.68	0.245	0.000147015	Neu2	UTRN	FALSE	FALSE

5.97E-09	0.812165988	0.44	0.102	0.00015074	Neu2	LCORL	TRUE	FALSE
6.02E-09	0.887971108	0.52	0.138	0.000151816	Neu2	SCCPDH	FALSE	FALSE
7.40E-09	0.398890605	0.24	0.03	0.000186721	Neu2	ARHGAP26	FALSE	FALSE
1.00E-08	1.072739688	0.68	0.27	0.000252236	Neu2	MBNL2	TRUE	FALSE
1.08E-08	1.011859509	0.8	0.402	0.000272108	Neu2	BLOC1S1	FALSE	FALSE
1.13E-08	0.760743835	0.44	0.104	0.000285623	Neu2	AGAP1	FALSE	FALSE
1.18E-08	0.840466466	0.68	0.256	0.000296888	Neu2	TEAD1	TRUE	FALSE
1.30E-08	0.57760991	0.24	0.032	0.000329001	Neu2	CYB5R2	FALSE	FALSE
1.38E-08	0.888276394	0.48	0.12	0.000347733	Neu2	NTRK2	FALSE	TRUE
1.45E-08	0.892537848	0.4	0.088	0.000365419	Neu2	ZDHHC8	FALSE	FALSE
1.80E-08	0.380706019	0.28	0.039	0.000453308	Neu2	IQGAP2	FALSE	FALSE
2.13E-08	0.959083693	0.56	0.183	0.000536339	Neu2	CFL2	TRUE	FALSE
2.61E-08	0.778013105	0.32	0.059	0.000657648	Neu2	ADPRHL1	FALSE	FALSE
2.65E-08	1.23213994	0.52	0.163	0.000667839	Neu2	RASAL2	FALSE	FALSE
2.65E-08	0.699354554	0.4	0.088	0.000669296	Neu2	ALDH6A1	FALSE	FALSE
3.05E-08	0.637323609	0.24	0.033	0.000768953	Neu2	LRRC8B	FALSE	FALSE
3.69E-08	0.675903856	0.4	0.089	0.000931534	Neu2	BCL2	FALSE	FALSE
3.95E-08	0.680830815	0.4	0.088	0.000996062	Neu2	SDC3	FALSE	FALSE
4.11E-08	0.81804341	0.6	0.212	0.001037547	Neu2	PFKL	FALSE	FALSE
4.48E-08	0.926447747	0.4	0.093	0.001129743	Neu2	RBM43	FALSE	FALSE
4.62E-08	1.045328494	0.76	0.359	0.001165076	Neu2	NDRG1	FALSE	FALSE
5.20E-08	0.525865858	0.24	0.034	0.001311754	Neu2	TMCO4	FALSE	FALSE
5.28E-08	0.488896276	0.24	0.032	0.001332932	Neu2	CADM2	FALSE	TRUE
6.28E-08	0.556537417	0.32	0.06	0.00158535	Neu2	NGF	FALSE	FALSE
6.78E-08	0.933536379	0.44	0.117	0.001709637	Neu2	FLNB	FALSE	FALSE
7.07E-08	0.507320467	0.24	0.035	0.001783557	Neu2	AC012360.3	FALSE	FALSE
7.26E-08	0.840562223	0.68	0.275	0.001832003	Neu2	C9orf3	FALSE	FALSE
7.30E-08	0.642068944	0.4	0.091	0.001842284	Neu2	PTPRF	FALSE	TRUE
7.56E-08	0.940809761	0.48	0.129	0.001908943	Neu2	PLAT	FALSE	FALSE
8.69E-08	0.682396642	0.4	0.092	0.002192058	Neu2	AC009041.2	FALSE	FALSE
9.42E-08	0.897205073	0.6	0.215	0.002375832	Neu2	SGCE	FALSE	TRUE
9.74E-08	0.755160355	0.24	0.036	0.002458746	Neu2	LYRM9	FALSE	FALSE
1.00E-07	0.607297585	0.32	0.062	0.002527614	Neu2	CASC15	FALSE	FALSE
1.12E-07	1.001018332	0.68	0.284	0.002836033	Neu2	MRPS6	FALSE	FALSE
1.43E-07	0.843333099	0.28	0.05	0.003602359	Neu2	TXNRD2	FALSE	FALSE
1.51E-07	0.834752	0.72	0.324	0.003803505	Neu2	VAMP2	FALSE	FALSE
1.53E-07	0.935203406	0.44	0.122	0.00385226	Neu2	CEMIP2	FALSE	FALSE
1.65E-07	0.583700981	0.36	0.079	0.004174531	Neu2	MTHFR	FALSE	FALSE
1.79E-07	0.478198364	0.32	0.061	0.004505588	Neu2	GFRA1	FALSE	TRUE
1.81E-07	0.810431909	0.56	0.171	0.004568953	Neu2	STMN1	FALSE	FALSE
2.00E-07	0.416253361	0.52	0.133	0.005042899	Neu2	CADM1	FALSE	TRUE
2.02E-07	0.587280993	0.28	0.05	0.005086159	Neu2	NUDT12	FALSE	FALSE
2.27E-07	0.690593945	0.32	0.065	0.005739726	Neu2	TOM1L2	FALSE	FALSE
2.28E-07	0.538297084	0.36	0.079	0.005746896	Neu2	SYT11	FALSE	FALSE
2.51E-07	0.666659402	0.36	0.083	0.006331339	Neu2	AC005154.1	FALSE	FALSE
2.58E-07	0.940926656	0.68	0.298	0.006502817	Neu2	UACA	FALSE	FALSE
2.66E-07	1.03622572	0.56	0.197	0.00671511	Neu2	USP53	FALSE	FALSE
2.80E-07	0.461334196	0.28	0.051	0.007055275	Neu2	AL450998.2	FALSE	FALSE
3.06E-07	0.561004168	0.56	0.17	0.007727104	Neu2	CELF2	FALSE	FALSE
3.16E-07	0.971656129	0.52	0.177	0.007985918	Neu2	ARHGAP10	FALSE	FALSE
3.47E-07	0.801281058	0.64	0.251	0.008745764	Neu2	LAMB1	FALSE	FALSE
4.61E-07	0.967941204	0.36	0.088	0.01162257	Neu2	IGSF8	FALSE	TRUE
5.52E-07	0.794605006	0.68	0.307	0.013938688	Neu2	SLC44A1	FALSE	TRUE
6.06E-07	0.619734406	0.28	0.05	0.015302094	Neu2	FGD4	FALSE	FALSE
6.12E-07	0.800311214	0.48	0.152	0.015433048	Neu2	FMNL2	FALSE	FALSE
6.53E-07	0.911517455	0.44	0.131	0.016472396	Neu2	TDRD3	FALSE	FALSE
7.11E-07	0.587838874	0.24	0.04	0.017939162	Neu2	PDZD2	FALSE	FALSE
7.51E-07	0.467556537	0.28	0.054	0.018959496	Neu2	TLN2	FALSE	FALSE
7.74E-07	0.585601356	0.52	0.18	0.019533737	Neu2	AASDHPPT	FALSE	FALSE
8.31E-07	0.594936488	0.32	0.07	0.020970755	Neu2	SLC23A2	FALSE	TRUE
8.79E-07	0.914070271	0.56	0.22	0.02219135	Neu2	ADAM10	FALSE	TRUE
9.70E-07	0.525949918	0.28	0.055	0.024482027	Neu2	PELI2	FALSE	FALSE
9.96E-07	0.725920298	0.32	0.069	0.025127794	Neu2	FXYD1	FALSE	FALSE
1.02E-06	1.516794321	0.52	0.199	0.025732755	Neu2	CUEDC2	FALSE	FALSE
1.04E-06	0.797428318	0.64	0.294	0.026246581	Neu2	CUX1	TRUE	FALSE
1.05E-06	0.753110173	0.6	0.23	0.026492435	Neu2	VWA1	FALSE	FALSE
1.08E-06	0.73432315	0.72	0.323	0.027338685	Neu2	PLXDC2	FALSE	TRUE
1.09E-06	0.754719195	0.32	0.071	0.027445199	Neu2	ABCA2	FALSE	TRUE
1.10E-06	0.688125652	0.88	0.471	0.027654679	Neu2	TUBA1A	FALSE	FALSE
1.16E-06	0.803244457	0.72	0.379	0.029350232	Neu2	RHEB	FALSE	FALSE
1.33E-06	0.724807341	0.72	0.344	0.033515443	Neu2	PRNP	TRUE	TRUE
1.38E-06	0.853420019	0.6	0.275	0.03469757	Neu2	NDUFA10	FALSE	FALSE
1.45E-06	0.604064218	0.52	0.18	0.036711379	Neu2	FYN	FALSE	FALSE
1.56E-06	0.755128551	0.48	0.168	0.039456844	Neu2	MPP6	FALSE	FALSE
1.66E-06	0.812073726	0.64	0.301	0.041857098	Neu2	TMEM123	FALSE	TRUE
1.80E-06	0.525127751	0.24	0.043	0.045447147	Neu2	NOL4L	FALSE	FALSE
1.81E-06	0.693157913	0.44	0.134	0.045795377	Neu2	RNPC3	FALSE	FALSE
1.88E-06	1.105831707	0.56	0.237	0.047476182	Neu2	IP6K2	FALSE	FALSE
