# Supplementary material for: Spatially defined single-cell transcriptional profiling characterizes diverse chondrocyte subtypes and nucleus pulposus progenitors in human intervertebral discs
Source: Bone Res. 2021 Aug 16;9:37. doi: 10.1038/s41413-021-00163-z (PMC8368097; doi:10.1038/s41413-021-00163-z)
Supplement: Supplementary file 9 — Supplementary Table 8 [file 41413_2021_163_MOESM9_ESM.pdf]

Supplementary Table 8. Information of 19 gene modules by Monocle 3 analysis					
	id	module	supermodule	dim_1	dim_2
6	PARK7	1	1	2.34415758	1.629257298
21	WASF2	1	1	0.269908134	0.461846328
22	ATP5IF1	1	1	2.507701997	1.679545379
29	YBX1	1	1	1.136398558	2.562663532
32	PRDX1	1	1	1.627008561	0.348121679
33	UQCRH	1	1	1.595473651	1.966002202
42	SERBP1	1	1	0.987501387	1.047510243
57	S100A10	1	1	1.41831458	0.352954305
58	S100A11	1	1	1.234839443	0.192739404
64	SSR2	1	1	2.004076127	1.831393934
80	PRDX6	1	1	2.527957562	1.603643156
81	GAS5	1	1	1.076460008	2.103023506
95	NUCKS1	1	1	0.699249987	0.669512248
100	NENF	1	1	1.323266748	1.302152491
105	H3F3A	1	1	1.960776929	0.738868928
106	TOMM20	1	1	0.998886947	1.758951641
110	LAPTM4A	1	1	0.885827068	0.489610768
114	OST4	1	1	2.119259004	0.854516244
116	MRPL33	1	1	2.014263514	1.812074876
120	COX7A2L	1	1	1.716072205	2.025498844
121	CALM2	1	1	1.479880814	0.420437491
131	RPL31	1	1	0.761850599	2.857171989
140	MZT2B	1	1	1.397203926	2.319166398
155	HSPD1	1	1	0.822091941	1.048277951
160	EEF1B2	1	1	0.873216037	2.777833915
168	NCL	1	1	0.820594791	1.121000863
187	TMA7	1	1	2.002481107	0.639967537
189	SPCS1	1	1	2.182579163	1.154444313
190	SELENOK	1	1	2.293847922	1.376252032
193	THOC7	1	1	1.369699005	1.963236309
207	CNBP	1	1	1.01208711	1.372802353
223	SEC62	1	1	0.782617096	0.959361411
242	OCIAD1	1	1	1.437230591	0.930238104
248	CCNI	1	1	0.67553759	1.533813215
260	SNHG8	1	1	1.242166523	2.032068706
262	ANXA5	1	1	1.640977744	0.259641564
265	NDUFC1	1	1	2.088527564	1.074166751
272	HMGB2	1	1	0.964013342	1.845007396
289	BTF3	1	1	1.001677875	2.500843025
290	NSA2	1	1	1.068667773	1.932040668
291	TBCA	1	1	2.123462323	1.057664848
297	COX7C	1	1	1.179785971	2.341192699
300	EPB41L4A-/	1	1	1.195185307	2.040703989
306	HINT1	1	1	2.155560616	1.947542167
310	SKP1	1	1	1.282876972	1.860329128
328	NPM1	1	1	0.780562524	1.752391792
330	ATP6V0E1	1	1	2.331438188	1.640626884
342	TMEM14C	1	1	1.703877095	1.551551557
344	DEK	1	1	0.966193799	0.948986865
350	C6orf48	1	1	0.951932315	1.874510265
356	RPS10	1	1	0.859835986	2.881567932
362	HSP90AB1	1	1	0.759805802	1.690399862
367	COX7A2	1	1	2.431373719	1.413856006
371	SNHG5	1	1	1.084659938	2.103975273
373	SNX3	1	1	1.59297717	1.478392816

379	RWDD1	1	1	1.645265464	1.700648761
395	PSMB1	1	1	2.341238383	1.55438993
396	RAC1	1	1	1.368044499	0.922341443
398	NDUFA4	1	1	2.412076835	1.395166374
404	TOMM7	1	1	1.013115291	2.507935262
409	7-Sep	1	1	1.171939734	0.886942602
415	PPIA	1	1	1.635514144	0.740013934
420	CHCHD2	1	1	2.444229964	1.754114366
440	MRPS33	1	1	2.045486573	1.654425836
452	UXT	1	1	1.814274196	1.736097551
462	RPL36A	1	1	1.078133587	2.212032295
467	TCEAL4	1	1	1.532839898	1.205454445
472	CDR1	1	1	0.605848435	1.26569376
475	SSR4	1	1	2.117856149	0.422122217
492	SNHG6	1	1	0.978784684	2.048529125
498	UQCRB	1	1	1.07569075	2.40913055
503	EIF3E	1	1	1.020235304	2.002360321
511	EEF1D	1	1	0.944879059	2.700384117
527	ANP32B	1	1	0.926534418	1.534430838
535	ATP6V1G1	1	1	2.278053883	1.266939497
543	SET	1	1	0.875809077	1.173927045
548	EDF1	1	1	2.302152757	1.444330788
574	TMEM258	1	1	2.00509036	0.400658584
575	COX8A	1	1	2.536195401	1.802838064
579	TRMT112	1	1	2.245248918	1.343250609
580	PRDX5	1	1	2.655470971	1.993718601
588	GSTP1	1	1	2.676108722	1.833022094
603	ATP5MG	1	1	1.677224997	2.241944766
624	ANAPC16	1	1	1.43374813	1.844934202
626	VDAC2	1	1	1.562363986	1.806859231
650	PTMS	1	1	1.903216247	0.579239524
653	C12orf57	1	1	1.117090706	1.582400299
667	LDHB	1	1	1.54112399	2.018284536
675	ZCRB1	1	1	1.781705026	1.680227018
681	EIF4B	1	1	0.575749997	1.659420228
684	ATP5MC2	1	1	1.234057669	2.283235288
685	HNRNPA1	1	1	0.632951383	1.692140556
687	CD63	1	1	1.631147031	0.441157258
689	PTGES3	1	1	1.063786153	1.302606559
699	NAP1L1	1	1	0.745876197	1.552250839
712	PEBP1	1	1	0.904999141	0.521552957
720	SAP18	1	1	2.22034872	1.584759808
732	COMMD6	1	1	1.239295606	2.213208652
738	DAD1	1	1	2.011762265	0.551244355
740	RPL36AL	1	1	1.090259317	2.290227151
744	LGALS3	1	1	1.585986022	1.936659551
757	IFI27L2	1	1	1.887691621	1.659471488
761	ATP5MPL	1	1	2.38625491	0.994561172
762	SNRPN	1	1	2.084847335	1.950051046
765	SRP14	1	1	1.560182933	0.654184974
771	EID1	1	1	0.940515522	0.521914936
773	RSL24D1	1	1	1.06919289	1.828577257
778	RPS27L	1	1	2.321940307	1.505802608
783	RPL4	1	1	0.531768266	1.776368833
790	MORF4L1	1	1	1.041613463	0.480419672
792	RPL9P9	1	1	1.245465759	2.096071458
793	RPS17	1	1	1.164109591	1.898364997
795	SEC11A	1	1	1.596845273	2.036074853

805	RSL1D1	1	1	0.940481667	1.616814113
831	YWHAE	1	1	1.342403416	0.781418658
838	LRRC75A-A	1	1	0.931476954	2.55725143
843	KRT10	1	1	2.294807319	0.772053814
845	RPL27	1	1	0.743876223	2.971106029
868	SUMO2	1	1	1.536357287	0.805297232
883	MYL12B	1	1	1.761894349	0.655558503
891	RPL17	1	1	1.283656005	2.053455806
902	SNRPB2	1	1	2.32082117	1.21491704
920	ZFAS1	1	1	1.157299165	2.05347631
930	PPDPF	1	1	1.380060557	1.146837211
933	CIRBP	1	1	1.097904805	2.39198587
934	OAZ1	1	1	2.331684951	1.465391016
938	NDUFA11	1	1	2.403218869	2.081408716
944	WDR83OS	1	1	2.232510451	2.000863767
946	PRDX2	1	1	2.149806146	2.046890712
959	COX6B1	1	1	2.344677571	1.14714036
961	EIF3K	1	1	2.096264962	2.263234354
971	SNRPD2	1	1	2.389238242	1.472760416
973	KDELR1	1	1	1.563505534	1.2288687
975	RPS4Y1	1	1	1.113859538	2.191899991
976	MIF	1	1	2.088010673	0.430030561
977	DDT	1	1	1.661215667	1.965691543
982	SELENOM	1	1	1.779676084	1.06841538
984	ST13	1	1	0.75627232	1.402179933
997	SOD1	1	1	1.836249474	2.136499382
1029	BRK1	1	1	1.841250781	2.051454044
1030	SF3B5	1	1	2.588309888	1.799278951
1034	EIF3L	1	1	0.688193087	1.635809637
1037	ATP5PO	1	1	1.71460164	2.139380193
1044	TUBB	1	1	2.100652818	0.533823646
1046	MRPS21	1	1	2.507023457	1.359748817
1051	UFC1	1	1	1.830690268	2.006792522
1053	PABPC1	1	1	0.672378901	1.564206577
1057	CDC42	1	1	1.221429709	0.608200288
1063	UBB	1	1	1.730839375	0.624626255
1065	MGST3	1	1	2.66247547	1.72922299
1074	TMEM230	1	1	2.123493318	1.131782508
1084	RPA3	1	1	1.976166848	1.504758454
1091	RPS21	1	1	0.780847315	2.919053531
1103	METAP2	1	1	1.106450561	1.651751614
1107	LGALS1	1	1	2.04969037	0.544400549
1117	SSB	1	1	1.874390248	0.831005788
1137	GTF2A2	1	1	2.15306795	0.892133689
1160	MTDH	1	1	1.363584165	0.208305097
1161	C4orf3	1	1	1.765663985	0.778765655
1163	PA2G4	1	1	1.332297448	1.171669102
1166	RHEB	1	1	2.062607888	1.275872088
1185	RHOA	1	1	1.515173558	0.546565688
1188	CMPK1	1	1	0.690237287	1.223981715
1189	C11orf58	1	1	1.357824568	1.486725188
1191	SERF2	1	1	1.496923808	0.449545658
1194	TIMP1	1	1	1.304687504	0.315999783
1196	SERP1	1	1	2.10425413	1.760671592
1197	MRFAP1	1	1	1.676622275	1.241126872
1198	EEF2	1	1	0.511661056	1.778006769
1200	BNIP3L	1	1	0.80482078	1.096253968
1202	TMBIM4	1	1	1.926739577	1.458643055

1210	MRPL51	1	1	2.537958268	1.618431545
1213	SLC25A3	1	1	1.604438428	2.408272958
1215	TRIR	1	1	1.495661382	1.969301916
1218	SRP9	1	1	1.133401755	0.887252069
1230	CUTA	1	1	2.437059287	2.304828144
1233	SMIM26	1	1	1.859443311	1.796333051
1239	COX4I1	1	1	0.790606026	2.990830398
1243	SNRPD3	1	1	2.238769893	1.081756807
1257	CLTA	1	1	1.92448533	2.177376485
1261	UBXN4	1	1	1.275326613	0.18773619
1270	MTPN	1	1	1.277606729	1.057088352
1281	RAB13	1	1	2.696778659	2.134131647
1285	LSM5	1	1	2.294021491	1.379754162
1290	MRPL57	1	1	2.414126042	1.81288836
1301	GPX4	1	1	2.146026973	2.137633062
1308	HMGN2	1	1	0.936743144	1.254188276
1337	EIF4A2	1	1	0.624557618	1.494864321
1339	REEP5	1	1	1.957996253	1.042219377
1346	FAM96B	1	1	2.722280625	1.7935879
1355	PDLIM4	1	1	2.78214157	1.829039312
1356	C1orf21	1	1	0.803789858	1.404077268
1357	DUT	1	1	2.051470879	1.603762961
1361	HNRNPDL	1	1	0.836815242	0.994558311
1374	EBPL	1	1	1.747711305	1.799189067
1381	SH3BGRL	1	1	1.404169206	1.031726098
1391	ESD	1	1	1.509544257	1.516575909
1395	TMEM14B	1	1	2.0253166	1.78822229
1401	POLR2K	1	1	2.294834022	0.912051058
1405	ACTG1	1	1	1.518592242	0.492369688
1421	CHURC1	1	1	1.473001603	1.554154611
1438	HOXC10	1	1	1.147467736	1.434656239
1442	DCXR	1	1	2.030277375	2.274574972
1443	EIF3I	1	1	2.602014665	1.76247356
1444	SQSTM1	1	1	0.437377099	0.622016108
1447	SRRM1	1	1	0.98637009	1.119426942
1454	CNPY2	1	1	2.116882924	0.463981545
1460	FIS1	1	1	2.599016789	1.869574523
1468	NOP53	1	1	0.59494007	1.786852575
1481	CBX1	1	1	1.057029609	1.025994516
1490	TMEM179B	1	1	2.196288947	1.975363708
1508	ATP5F1D	1	1	2.6866895	1.960612035
1512	HNRNPD	1	1	0.710598949	1.079190469
1522	NUDC	1	1	1.595606927	1.280156589
1537	GDI2	1	1	1.371540789	1.179550505
1547	EIF3M	1	1	1.086859588	1.970918155
1579	SMDT1	1	1	1.545530919	2.173127389
1586	SIVA1	1	1	2.193074349	1.935591913
1594	SYF2	1	1	1.409503344	1.964855886
1595	VAMP2	1	1	0.540125493	0.741055108
1607	AIMP1	1	1	1.850682858	1.441727496
1608	MRPL40	1	1	2.152530078	1.60709188
1615	DNAJC8	1	1	1.391312961	1.132423258
1619	YWHAQ	1	1	1.792065267	0.985379434
1621	SLC25A6	1	1	1.269901875	2.554600454
1624	MZT2A	1	1	2.250220183	2.170913196
1631	EIF1AX	1	1	0.924990419	1.367032624
1651	NOL7	1	1	2.299086932	1.255047179
1658	C8orf59	1	1	2.359226588	1.573017812

1666	PYURF	1	1	1.604260568	1.162472344
1668	TCEA1	1	1	0.983732346	1.071560359
1677	RTF1	1	1	1.303574923	0.890564895
1680	CALM1	1	1	1.768155698	0.671153999
1685	PPIG	1	1	0.789598707	0.98139844
1699	RNF7	1	1	2.616744164	1.857997632
1701	SUMO1	1	1	2.470269326	1.499247885
1709	ZC3H15	1	1	1.452411536	0.860755539
1715	LRRC75A	1	1	0.844915394	2.716294027
1727	PERP	1	1	0.229485039	0.428278006
1734	PSMB4	1	1	2.148552779	1.976872898
1743	MDH1	1	1	2.439865712	1.667177177
1755	VAPA	1	1	0.881954316	0.572628355
1758	EIF2A	1	1	0.984227423	1.721852518
1765	HEBP2	1	1	2.546730164	1.914682842
1789	CHMP5	1	1	2.237461451	1.554213024
1803	SCOC	1	1	1.736486081	0.761140323
1805	ERP29	1	1	2.224141482	2.303017354
1811	ANP32E	1	1	0.983281139	1.031814313
1813	KTN1	1	1	0.868856791	0.980896688
1818	DCTN3	1	1	2.240867261	2.167847372
1819	CAMLG	1	1	1.432792429	1.933350301
1847	CSDE1	1	1	0.651592258	1.444118
1859	TCEA3	1	1	1.795170907	2.121288753
1860	DGUOK	1	1	2.424878005	1.285313464
1862	NDUFS4	1	1	1.822696332	1.77453897
1864	CKS1B	1	1	1.934289817	1.861787296
1867	KHDRBS1	1	1	0.775624279	1.141578532
1868	SFT2D1	1	1	2.536192063	1.827909923
1893	ARL6IP4	1	1	2.283035878	2.222475267
1898	GINM1	1	1	1.522574071	1.227864957
1906	SARAF	1	1	0.882501964	0.400390125
1911	PPP1CB	1	1	1.176594142	0.327261663
1912	EIF2S3	1	1	0.787828807	1.680966115
1932	CNIH4	1	1	2.042355184	1.351678944
1946	ANP32A	1	1	1.267343048	0.712112403
1947	EIF1B	1	1	1.202218298	1.585980154
1968	EIF3G	1	1	2.299470548	2.222629047
1970	METTL26	1	1	2.129018668	2.223909355
1977	REX1BD	1	1	2.42816532	2.12046907
1986	TLN1	1	1	1.084707622	0.964235163
1999	ZFR	1	1	0.586073283	1.246855474
2002	RAD23A	1	1	2.396391753	1.702013946
2010	HIST1H1C	1	1	1.093057994	1.867295242
2013	CAMTA1	1	1	2.48174632	1.66080091
2018	TCEAL8	1	1	2.027868871	1.452191806
2019	CFDP1	1	1	1.207470063	0.950869537
2023	DNAJC19	1	1	2.146585587	1.644497014
2028	GHITM	1	1	1.433651928	1.350741601
2044	ARL3	1	1	2.218609933	2.294779516
2045	CSNK2B	1	1	2.071573142	2.145549274
2048	PRPF6	1	1	0.937757019	1.598840571
2060	SRI	1	1	2.561170463	1.67847035
2066	PDCD6	1	1	2.050767783	0.991323328
2068	IGBP1	1	1	1.558873061	2.136365152
2078	PAIP2	1	1	1.400755171	1.261679149
2085	GLO1	1	1	1.171540622	0.435648656
2086	HADHA	1	1	0.849567059	1.194542742

2106	CPNE3	1	1	0.482779447	0.549583888
2107	POLR2I	1	1	2.593452577	2.065610862
2113	DHRS7	1	1	1.500908259	1.271842933
2125	RRAGA	1	1	1.775682811	0.465703345
2163	CWC15	1	1	2.082934264	1.308192707
2164	NDUFB5	1	1	2.1556927	2.264604545
2167	EMC2	1	1	1.898482922	1.142201758
2168	UBE2D2	1	1	1.855353002	1.98789928
2172	SNX6	1	1	1.725774888	1.241662837
2178	TMEM219	1	1	2.2996496	2.267160392
2179	RAB7A	1	1	1.445290927	0.327787972
2181	RPS19BP1	1	1	2.584224824	1.906549669
2193	ANXA7	1	1	0.693841342	1.149440504
2214	PTPRD-AS1	1	1	1.096976165	1.775339103
2217	FBL	1	1	1.058642987	1.888940072
2225	H1FX	1	1	1.147033576	2.280918098
2231	UBE2V2	1	1	2.240452174	0.936165309
2249	POLE3	1	1	1.369012479	1.00432036
2250	MRPL18	1	1	2.458474521	1.845324493
2256	FERMT2	1	1	0.560272817	0.180314041
2261	CIR1	1	1	1.254818086	1.508764005
2266	FUNDC2	1	1	1.378682617	1.501445151
2269	IK	1	1	1.060747389	1.124609566
2270	CDC42EP5	1	1	2.777075652	1.865891433
2280	NR3C1	1	1	0.44289488	0.60241685
2286	DANCR	1	1	1.872912291	2.143584705
2289	CYSTM1	1	1	0.570191149	0.422841406
2291	LSM2	1	1	2.270087842	2.294681764
2304	DMAC1	1	1	2.20019472	1.478640056
2308	TERF2IP	1	1	0.683573488	0.536837435
2309	PNKD	1	1	2.184794311	2.095920301
2313	RNASEH2C	1	1	2.669090632	1.906012512
2317	CCT4	1	1	0.865653876	1.200057483
2323	NDUFA5	1	1	2.158975486	0.819325185
2337	HAX1	1	1	2.211221579	1.894015527
2346	PAK2	1	1	0.932964806	0.750676728
2347	METTL5	1	1	2.313476209	1.261237598
2354	ECHS1	1	1	1.984612826	2.143218732
2358	THRAP3	1	1	1.115531568	1.191474414
2367	EIF3D	1	1	1.455574993	2.013303733
2381	RNF13	1	1	0.559345726	1.244963384
2387	TAX1BP1	1	1	1.410784725	0.316575921
2391	AP3S1	1	1	1.147890691	0.485265291
2392	OTUD6B-AS	1	1	1.702060107	1.421815849
2408	ALKBH7	1	1	2.230925683	2.318877197
2418	HIST1H4C	1	1	1.592129592	1.765528655
2422	URI1	1	1	0.960069303	1.046255804
2424	RAB14	1	1	1.548551444	1.045921779
2427	NSRP1	1	1	1.008356456	1.113728381
2428	EBAG9	1	1	0.902544383	1.461904383
2430	SUGT1	1	1	1.208847765	0.404287136
2434	SRP72	1	1	1.19491506	0.919821835
2435	EIF3F	1	1	1.736355666	2.253147102
2449	ETFB	1	1	2.507359389	2.186948276
2454	COPRS	1	1	2.427260283	1.894929386
2460	MRPS36	1	1	2.588611249	1.549790836
2470	TAF7	1	1	0.995646957	0.925220466
2471	PCNP	1	1	0.920557026	1.52393291

2477	PCMT1	1	1	1.754187468	1.008562541
2481	CCT8	1	1	1.632387046	1.389586783
2495	MPG	1	1	2.708180312	1.832357383
2504	CEP63	1	1	1.124882225	1.235633946
2505	MMP24OS	1	1	1.986690406	2.06709764
2540	ACP1	1	1	2.171408538	1.382232762
2560	UFL1	1	1	1.25084186	0.249450303
2564	TXNL1	1	1	1.898747806	1.430669642
2567	EIF1AY	1	1	1.907534007	1.08890996
2569	C1orf43	1	1	1.61972535	1.755391813
2575	VPS28	1	1	2.483217839	2.08081863
2578	FXR1	1	1	0.762739901	1.44265697
2580	ECH1	1	1	2.647781018	1.673971391
2582	TMX4	1	1	0.627779368	1.134206272
2599	TMEM256	1	1	1.8895973	2.258378244
2600	PRRC2C	1	1	0.691427354	0.812122918
2607	ARL6IP1	1	1	0.977598432	1.300119615
2650	LZIC	1	1	1.056092385	0.98529706
2654	PSMG2	1	1	1.927657727	1.610675908
2660	YTHDC1	1	1	0.839117173	1.263752914
2677	YIPF3	1	1	2.624360923	1.675991512
2682	PNRC2	1	1	0.970797662	0.777786947
2688	MRPS26	1	1	2.021810416	2.036717868
2701	CBR1	1	1	2.621675853	1.829970098
2704	APPL1	1	1	0.330992345	0.583797372
2710	RTF2	1	1	1.95904148	1.881664014
2723	ARPP19	1	1	1.149670366	0.56342969
2730	PJA2	1	1	0.708766107	0.198169685
2762	POLR1D	1	1	1.443612222	2.093487478
2765	ZNF706	1	1	1.951870564	1.530444837
2767	PGLS	1	1	2.349922065	2.221672035
2774	CCNB1IP1	1	1	1.418421153	2.046428895
2779	TXN2	1	1	2.406825665	1.980226255
2789	MRPL43	1	1	2.374722842	1.524125433
2792	EMC4	1	1	2.228043918	1.348494506
2805	SWI5	1	1	2.360294703	1.937091327
2807	COPS2	1	1	0.910563592	1.453842378
2813	TKT	1	1	1.935948256	1.062131739
2818	MYL6B	1	1	2.481819991	1.450724698
2821	CDC26	1	1	2.307394151	2.231838441
2834	HPF1	1	1	1.89561737	0.512467719
2839	HDGF	1	1	1.731001023	1.258634663
2843	CFAP97	1	1	1.547168378	0.538706577
2848	AC113935.1	1	1	1.590976838	2.142998195
2849	DYNC1I2	1	1	1.084630135	0.982904411
2873	OSBPL1A	1	1	0.218091909	0.429101861
2878	TYW3	1	1	1.238197926	1.899458862
2879	CAPZA2	1	1	2.010556106	1.317815757
2905	PHB2	1	1	1.349588756	2.11423347
2908	RAB34	1	1	2.472499732	1.988968587
2911	DPY30	1	1	1.743034247	1.432283855
2937	ZC3H13	1	1	1.122605328	0.807224846
2944	HOXB7	1	1	1.635365609	1.404924846
2947	MRPL54	1	1	2.467327956	1.654271937
2950	SNW1	1	1	1.087165479	1.083150363
2951	CCDC90B	1	1	2.317030076	1.626298523
2965	TMEM216	1	1	1.516188148	0.91743145
2966	POLD2	1	1	2.53887618	2.152078128

2969	MMADHC	1	1	1.284058455	1.351952649
2978	PPP1CC	1	1	1.229813341	0.730206347
4	MRPL20	2	1	3.060851697	1.172585345
26	NDUFS5	2	1	3.163807277	1.672543502
45	GNG5	2	1	2.86261094	0.690986968
69	PFDN2	2	1	3.152455214	0.489757455
94	SNRPE	2	1	2.857791547	1.043445921
112	SF3B6	2	1	2.505472068	0.944646097
127	SNRPG	2	1	3.282138947	0.391317642
130	COX5B	2	1	3.082520369	1.754301048
139	DBI	2	1	3.464785699	1.147332049
149	ATP5MC3	2	1	3.559430722	1.30013094
156	HSPE1	2	1	3.09330285	0.708096242
163	ARPC2	2	1	3.409903888	1.120316244
188	MANF	2	1	2.681225184	-0.233015874
203	NDUFB4	2	1	2.706681613	1.601753092
231	C4orf48	2	1	3.691182736	0.98322115
277	SUB1	2	1	3.219093684	1.17748437
308	UQCRQ	2	1	3.552446011	1.107566929
331	PRELID1	2	1	3.453278665	0.632326401
334	HNRNPAB	2	1	3.071795587	-0.39512716
354	UQCC2	2	1	3.78578103	0.366062677
374	GTF3C6	2	1	3.724347714	0.26127497
407	CYCS	2	1	3.347805385	-0.289092411
427	SEM1	2	1	3.294485215	1.70349834
430	ATP5MF	2	1	3.40147293	0.837275362
470	NDUFA1	2	1	2.555588368	1.080602503
501	COX6C	2	1	2.403826837	0.573573387
529	SEC61B	2	1	2.32753003	0.312798119
532	TXN	2	1	2.459288243	0.378446913
547	PHPT1	2	1	3.413832787	1.201434112
555	POLR2L	2	1	3.301390532	0.93185184
576	FKBP2	2	1	2.278288964	0.394407726
577	PPP1R14B	2	1	3.753178719	0.237646258
584	CFL1	2	1	2.553543214	0.409737623
586	DRAP1	2	1	3.682202462	0.617788768
587	BANF1	2	1	2.960690383	0.599657572
593	NDUFC2	2	1	2.58807886	1.10155449
677	TUBA1C	2	1	2.989011887	0.265968895
688	RPS26	2	1	3.181882743	1.14125309
697	CCT2	2	1	3.053981666	-0.386748192
705	SNRPF	2	1	3.00625265	0.841448999
711	ARPC3	2	1	2.432476405	1.046551443
713	COX6A1	2	1	2.76429427	1.674244142
714	DYNLL1	2	1	2.542081241	0.831905222
718	RAN	2	1	3.473621491	0.273188389
723	POMP	2	1	3.302844171	1.036609626
748	ERH	2	1	2.455805425	0.95763681
803	ELOB	2	1	3.370141868	1.067243314
826	HSBP1	2	1	3.148311976	1.0296242
833	PFN1	2	1	2.644544963	0.449982918
834	TXNDC17	2	1	3.740637902	0.618527687
835	EIF5A	2	1	3.686903123	0.279855288
854	NME1	2	1	3.848148946	0.386977351
866	ATP5PD	2	1	2.953812007	1.238469458
872	SNHG16	2	1	2.619269732	0.459259844
886	SNRPD1	2	1	3.152512435	0.659945703
910	DYNLRB1	2	1	2.453553323	1.051918602

927	ATP5F1E	2	1	2.33518279	0.640358305
928	PSMA7	2	1	3.015451316	1.32003007
935	MYDGF	2	1	2.667553071	0.254547155
942	UBL5	2	1	2.591255073	1.116796112
949	C19orf53	2	1	2.637197379	1.53425107
951	TPM4	2	1	2.510630492	0.106479353
980	UQCR10	2	1	3.130132798	1.368769384
985	RBX1	2	1	3.43731821	1.257307983
987	SNU13	2	1	3.093323592	0.964714385
994	ATP5PF	2	1	2.752018813	1.347990489
1016	MYL12A	2	1	2.510311011	0.699297524
1020	PSMB5	2	1	2.983531121	0.992469407
1021	ARPC1B	2	1	2.525545243	0.023733622
1026	DNAJC15	2	1	3.908395413	0.715291357
1033	NEDD8	2	1	3.320976857	1.370483137
1045	NOP10	2	1	3.643303994	0.807350135
1047	TPM3	2	1	2.384485606	-0.075385415
1048	COX7B	2	1	3.435609464	1.339083529
1050	RPL22L1	2	1	3.806068067	0.454491473
1055	NDUFB2	2	1	3.488113526	1.213850832
1067	CLIC1	2	1	2.300845508	0.695598102
1075	ATP5MD	2	1	3.257512692	1.473980642
1077	EIF2S2	2	1	2.524559382	0.791273928
1088	NDUFB3	2	1	3.489136104	1.228278375
1089	COPZ1	2	1	2.709062222	1.143728352
1092	GSTO1	2	1	3.190286759	1.011237598
1096	LSM7	2	1	3.554936532	1.134812808
1101	ABRACL	2	1	3.906420354	0.229954815
1102	ATP5MC1	2	1	3.492387418	0.772300697
1109	UQCR11	2	1	3.363292817	1.439488745
1113	COPS9	2	1	3.475808267	1.462779618
1114	PDCD5	2	1	3.320129041	0.939596749
1115	MRPL52	2	1	3.607005242	0.455265022
1122	C1QBP	2	1	3.558059339	0.411659992
1128	TUBA1B	2	1	2.570213918	0.309735275
1132	LY6E	2	1	2.87803996	1.662432647
1133	CCT5	2	1	2.90516508	-0.60057699
1141	EIF3J	2	1	3.202126388	-0.169213676
1142	AURKAIP1	2	1	3.581490163	0.648648358
1148	H2AFZ	2	1	3.233778123	1.556790567
1153	NAA38	2	1	2.921425227	1.724757171
1155	SLC25A5	2	1	3.606048707	0.237662054
1157	RHOC	2	1	2.41480935	0.347819782
1158	SSBP1	2	1	2.682354573	1.402783847
1162	ANAPC11	2	1	3.08584607	1.424469686
1165	LRRC59	2	1	3.281269196	-0.283904874
1172	RTRAF	2	1	2.522213344	1.158582902
1174	SPTSSA	2	1	2.717261199	0.139974243
1178	GTF3A	2	1	3.501579884	0.070203937
1183	AP2S1	2	1	3.819874886	0.649058021
1187	LAMTOR5	2	1	2.588011865	0.977017141
1201	TUBB6	2	1	2.599693421	-0.188686946
1219	TIMM8B	2	1	3.684548501	0.563085711
1220	CCT6A	2	1	2.763274077	-0.299446211
1224	COX5A	2	1	3.466529969	0.53737191
1231	NDUFAB1	2	1	3.413153295	1.007959223
1236	TALDO1	2	1	2.813217048	1.188963628
1237	NDUFA13	2	1	2.885432366	1.636443115

1240	ARPC5L	2	1	3.474339847	-0.158677274
1246	NDUFB11	2	1	3.294974927	1.602860189
1265	PPA1	2	1	3.356207732	0.949662543
1282	PSMA4	2	1	3.534919385	0.082035667
1287	SLIRP	2	1	3.114344243	1.179917551
1294	DYNLT1	2	1	3.519980077	1.095193363
1298	ELOC	2	1	2.746544723	0.889557577
1300	CBX3	2	1	3.387872342	0.298446274
1305	ROMO1	2	1	3.457274083	1.33897934
1311	COPE	2	1	3.486922387	1.066364861
1312	FKBP1A	2	1	2.474743728	0.431242741
1320	SRM	2	1	3.434427146	0.523211754
1326	GPX1	2	1	3.921330098	0.672450519
1330	TPM2	2	1	2.416026238	0.55744294
1331	TXNL4A	2	1	3.499531154	0.551699913
1334	S100A16	2	1	2.357270602	0.055783248
1336	SDF2L1	2	1	3.42109907	0.327477372
1338	ATOX1	2	1	3.527403001	1.158953405
1345	NHP2	2	1	3.411002521	1.054776288
1354	ARPC5	2	1	2.519778136	0.251041866
1358	SRSF9	2	1	2.956206922	1.22663734
1371	TMEM147	2	1	3.216712359	1.374861694
1380	NDUFAF3	2	1	2.70680416	1.481869078
1385	ATP6V0B	2	1	3.376546267	0.384297765
1393	CAVIN3	2	1	2.574283961	0.599269426
1407	NDUFB8	2	1	3.009214286	1.664565063
1416	PSME2	2	1	3.971583013	0.608526981
1425	MINOS1	2	1	3.409664277	1.045911646
1427	NDUFB6	2	1	3.583734158	1.021598077
1432	PSMB6	2	1	3.478908423	0.568190849
1457	MRPL47	2	1	3.630230073	0.343244708
1461	NDUFB1	2	1	3.100858334	1.009485102
1477	FAM177A1	2	1	2.638793591	0.860699511
1483	RANBP1	2	1	3.674713258	0.333252287
1484	SNRPB	2	1	3.194776181	-0.311885159
1488	DEGS1	2	1	3.313506726	0.09345401
1494	ATP5ME	2	1	2.954763774	1.019264555
1500	UBE2N	2	1	3.287520055	-0.271423542
1502	CIB1	2	1	3.333866719	1.218480206
1505	ALYREF	2	1	3.326594237	-0.015508943
1533	GTF2H5	2	1	2.788839225	0.887116647
1536	CAP1	2	1	3.185706262	-0.412813404
1541	KRTCAP2	2	1	2.967292432	1.073605633
1543	CAPZB	2	1	3.229073171	0.286745763
1552	TBCB	2	1	2.861150626	1.631675816
1553	ENY2	2	1	2.944388989	1.185280538
1554	CCT3	2	1	2.984395627	-0.521656119
1559	SELENOW	2	1	3.28502715	1.840674615
1560	MDH2	2	1	3.348923091	1.35146997
1600	PSMD7	2	1	3.278861646	0.002499915
1605	MRPL12	2	1	3.882499818	0.506304181
1610	NIFK	2	1	2.951240186	0.314288891
1612	MRPL14	2	1	3.730911855	0.529011226
1618	CCDC167	2	1	3.921315793	0.601807332
1620	PSMD1	2	1	3.076138619	-0.327457802
1622	SRP19	2	1	2.764765147	-0.406029844
1626	PSMB3	2	1	3.530468825	0.576099492
1629	NDUFA6	2	1	3.650519494	0.190618134

1633	ATP5F1C	2	1	3.141644124	1.695181346
1635	NDUFS6	2	1	3.768944386	0.568616128
1636	MYL6	2	1	2.30633438	0.582245088
1641	RBM8A	2	1	3.084849242	-0.029347831
1642	GADD45GIF	2	1	3.241140965	1.540195203
1648	NDUFB10	2	1	3.198979978	1.533035136
1662	CYBA	2	1	3.506736163	1.264168716
1664	UBE2L3	2	1	3.113538865	0.505974329
1674	PSMA2	2	1	2.514375333	1.252835489
1675	CHMP2A	2	1	2.815403823	1.748994804
1682	RGS3	2	1	3.833857659	0.607088304
1686	NDUFB7	2	1	3.492614392	1.478118992
1688	CLEC11A	2	1	2.890747909	1.676593996
1690	NDUFB9	2	1	2.831774119	1.63156817
1693	NOP56	2	1	2.999907617	-0.537828528
1703	YIF1A	2	1	3.615250234	0.472773707
1712	ZNF593	2	1	3.472649459	0.0452478
1717	NDUFS8	2	1	3.828745488	0.544446624
1719	LSM3	2	1	2.940957908	1.453194356
1724	TMEM208	2	1	3.608212117	0.535591877
1728	BOLA3	2	1	3.738230351	0.491499877
1738	LAMTOR4	2	1	3.129902009	1.928034997
1753	NUDT1	2	1	4.03405774	0.242160893
1757	C1orf122	2	1	3.529324416	1.414345837
1759	MPC2	2	1	3.000838641	1.448077059
1761	RER1	2	1	3.019172315	1.339292622
1762	PSMA1	2	1	2.793876294	0.9191427
1771	C19orf24	2	1	3.412621621	0.758801437
1774	POLR2F	2	1	2.728043441	1.738270021
1779	LSM4	2	1	3.685199861	0.499185241
1781	EMC7	2	1	2.820600871	1.477440572
1783	IKBIP	2	1	3.583636884	0.527959979
1793	JTB	2	1	2.638384465	1.116555429
1796	TCEAL9	2	1	3.10808957	1.130168534
1798	NDUFA3	2	1	3.392858151	1.402862764
1814	ATP5PB	2	1	2.748266105	1.15491674
1816	PSMD8	2	1	3.40622342	0.786287165
1817	ATP1B3	2	1	3.548076753	-0.003055059
1822	PSMD14	2	1	3.2984134	-0.186759719
1833	C19orf70	2	1	3.153038148	1.708757616
1842	NUTF2	2	1	3.703822736	0.507083393
1844	MRPL17	2	1	3.36622012	0.662787056
1856	BCAP31	2	1	2.709879998	1.353888607
1858	FKBP3	2	1	3.175994758	0.253527916
1872	LAMTOR2	2	1	3.659028176	1.146630621
1876	GPATCH4	2	1	3.11765993	-0.246825063
1883	MRPL41	2	1	3.25506318	1.073999382
1887	EFHD2	2	1	3.775643948	-0.178773993
1900	RNF181	2	1	2.462559346	1.070533014
1901	TIMM13	2	1	3.57594407	0.730085826
1909	SCAND1	2	1	3.414964084	1.136894918
1916	APRT	2	1	3.597766046	1.113202429
1918	POLR2J	2	1	3.265999917	1.783553338
1927	GUK1	2	1	3.415520314	1.462074733
1930	NBDY	2	1	2.98418868	1.724993444
1935	NDUFAF8	2	1	3.727600697	0.790867305
1936	NDUFAF2	2	1	3.710655335	0.619192398
1948	CD320	2	1	3.021232728	0.965795017

1963	COX7A1	2	1	3.249545697	1.373728848
1969	ATP6V1F	2	1	3.192664746	1.605398035
1972	ATP5F1B	2	1	3.378319148	0.226648188
1982	TOMM5	2	1	3.788148049	0.383372283
1987	MRPS34	2	1	3.065067891	1.021181322
1988	OSTF1	2	1	3.905170564	0.783617354
1989	LAMTOR1	2	1	3.004896525	1.79763887
2007	PDAP1	2	1	2.988505486	1.062806106
2009	ATP5F1A	2	1	2.927722577	1.767607665
2011	MRPL13	2	1	3.768953923	0.710373378
2017	COMT	2	1	3.108496789	1.550685859
2025	HNRNPF	2	1	2.804186467	0.305450356
2030	ORMDL2	2	1	3.291452292	0.267299033
2038	SRA1	2	1	3.477693442	-0.061645352
2042	PTS	2	1	3.532747392	0.282823778
2047	RPL26L1	2	1	3.729052667	0.507052339
2052	BLOC1S1	2	1	3.688044194	1.005840517
2053	DBN1	2	1	3.856676701	0.2693573
2054	HMGN3	2	1	2.804095153	1.730113244
2059	LMAN2	2	1	2.707757119	1.015124655
2063	MRPL34	2	1	2.97577155	1.942576385
2065	APEX1	2	1	3.122412805	0.278887845
2067	SMIM37	2	1	3.199426774	1.288699365
2071	COA4	2	1	3.057394628	0.808003998
2072	STRAP	2	1	2.974612121	-0.390306664
2074	PET100	2	1	2.559943084	0.796368933
2083	PSME1	2	1	3.15328658	1.632523036
2084	LSM1	2	1	2.804521922	0.171951569
2089	CALM3	2	1	3.407056693	1.161627746
2095	PSMA3	2	1	3.344747905	0.066157705
2099	CAPNS1	2	1	2.587180499	0.377978778
2104	TWF1	2	1	3.009623889	-0.482273289
2110	MRPS15	2	1	3.488452081	0.333777762
2111	HIGD1A	2	1	3.421236638	0.360667086
2138	HDDC2	2	1	3.17759693	1.362643338
2144	SSU72	2	1	2.824661855	1.327656246
2148	CISD2	2	1	3.352535371	0.703586674
2158	CACYBP	2	1	3.157180671	0.969155288
2162	PSMB7	2	1	3.272407178	1.218036151
2171	UBE2I	2	1	2.939528111	1.050388432
2174	ACAA2	2	1	3.183341388	1.607040143
2188	EBNA1BP2	2	1	3.170317534	-0.226610088
2199	PSMC4	2	1	2.918304805	-0.408061766
2206	MCTS1	2	1	3.801894788	0.662773645
2212	ARPC4	2	1	3.086093787	0.817431307
2233	PSMC5	2	1	3.30243266	0.626980997
2243	GLRX3	2	1	3.415567521	-0.116429799
2252	BAX	2	1	3.679462079	0.431239045
2254	PSMA5	2	1	2.663281802	0.580592072
2259	PHB	2	1	3.575245027	0.769934512
2267	BZW2	2	1	3.685669545	0.13235811
2273	PPM1G	2	1	3.068339232	0.203703499
2274	TIMM17A	2	1	3.116776113	-0.004104548
2277	NDUFA12	2	1	3.498375777	1.100840307
2284	EIF6	2	1	3.390674952	0.402338779
2299	PSMB2	2	1	3.055678252	0.517128504
2314	AP2M1	2	1	2.847366694	0.596194363
2316	NANS	2	1	3.475287799	-0.070351833

2325	CKLF	2	1	3.112916354	1.447697616
2327	NCBP2	2	1	3.289559726	-0.089653247
2332	DDOST	2	1	2.922780875	1.174329615
2335	PSMD2	2	1	3.132924918	-0.241935396
2342	C9orf16	2	1	3.07426298	1.378270245
2356	ZNHIT1	2	1	3.525582437	1.485077954
2370	XRN2	2	1	2.771159057	-0.261964933
2371	GTF2F2	2	1	3.040429954	0.415748215
2374	FIBP	2	1	3.564109925	0.528658188
2394	CCDC124	2	1	3.508145455	0.579951859
2410	ADRM1	2	1	3.387464646	0.263881004
2416	MAGOH	2	1	2.65262926	1.077404952
2417	ISOC2	2	1	3.877658013	0.479612983
2431	NDUFA2	2	1	3.05000985	1.630375243
2444	UTP11	2	1	3.587552194	0.067229993
2459	EMC6	2	1	3.260515574	0.783658481
2465	UQCRFS1	2	1	2.729159955	1.161660409
2466	EIF4E2	2	1	3.647852067	0.259695447
2468	GSPT1	2	1	2.84590352	-0.406945401
2476	NDUFA8	2	1	2.881583575	1.039304591
2484	NPM3	2	1	3.933432225	0.280558265
2491	TCP1	2	1	3.133495931	-0.353667596
2496	MRPL3	2	1	3.304952983	-0.070486062
2501	PSMD4	2	1	2.911228541	1.292469359
2508	DCUN1D5	2	1	3.77774537	0.379574097
2510	BUD31	2	1	3.270922784	1.706889129
2513	ITPA	2	1	3.733797196	0.305463231
2517	PRDX3	2	1	3.03968871	0.727041698
2520	H2AFY	2	1	3.330598239	-0.361607182
2533	CYC1	2	1	3.389733437	0.641737259
2539	C3orf14	2	1	3.6344353	0.742990113
2552	CAPZA1	2	1	2.650425795	-0.186884695
2553	MAGOHB	2	1	3.741227273	0.543090022
2557	MRPL27	2	1	3.342310075	0.749923206
2581	POLE4	2	1	3.698156003	1.217904783
2606	MSRB2	2	1	3.106922988	1.574833966
2610	MORN2	2	1	3.557087544	1.34141562
2612	TIMM10	2	1	3.384229306	0.804931856
2613	LSM8	2	1	2.916131619	1.268624998
2622	MYCBP	2	1	3.874565724	0.353454686
2635	SNRPC	2	1	3.322426681	0.980563736
2648	CD3EAP	2	1	2.912170056	-0.359421424
2658	NAA10	2	1	3.687389497	0.59948746
2661	TRAPPC1	2	1	3.624845151	1.357230401
2662	EVA1B	2	1	2.946943168	0.914214945
2663	COA6	2	1	3.536625985	0.417597568
2667	GCSH	2	1	3.784839753	0.740203715
2669	UBE2K	2	1	2.952685718	-0.504006186
2672	MRPS12	2	1	3.448587779	0.586226201
2675	LSM10	2	1	3.327247027	1.110398984
2685	RAB5C	2	1	3.109507684	0.653595484
2697	PPP1R7	2	1	2.603536252	1.300623036
2703	PTRHD1	2	1	2.813449506	1.429459668
2707	NME4	2	1	3.072896604	1.538729644
2726	UCHL3	2	1	3.909201745	0.347397602
2727	SNRPA1	2	1	3.463979844	0.927930928
2747	MRTO4	2	1	3.199219827	-0.272365996
2748	GTPBP4	2	1	2.878145818	-0.478595861

2768	PSENEN	2	1	3.52423108	0.942469335
2776	NIP7	2	1	3.081949834	-0.534415224
2778	RAB5IF	2	1	3.415186528	0.046047605
2783	JOSD2	2	1	3.491376046	1.043204761
2786	TAF10	2	1	3.653273229	1.11782775
2790	POLR2H	2	1	3.051727656	0.641725636
2797	SMIM7	2	1	3.005204324	1.486984587
2798	MRPS16	2	1	2.812191371	0.259703076
2801	LSM6	2	1	3.017834548	1.314990974
2803	MIEN1	2	1	2.765743855	0.182580507
2808	TOMM22	2	1	2.733317021	0.765671111
2819	SNHG15	2	1	3.079203967	-0.206222468
2822	DENR	2	1	2.965345506	-0.177052849
2824	RALA	2	1	3.041597012	-0.362823994
2825	RPF2	2	1	2.855723266	-0.021622473
2831	UBE2A	2	1	2.834195498	0.425849534
2837	SMIM30	2	1	3.753748063	0.820321894
2844	LYAR	2	1	2.795719985	0.071050263
2846	WDR43	2	1	2.873958472	-0.457665221
2847	PPP1CA	2	1	3.528608207	0.410402155
2853	NIPA2	2	1	2.969905023	-0.410430693
2857	CMSS1	2	1	2.900532607	0.246500051
2858	EIF4E	2	1	2.727542285	-0.469709494
2863	TXNDC9	2	1	3.675308351	0.221513427
2864	MRPL42	2	1	3.059287433	0.632819748
2866	GLRX5	2	1	3.01302636	0.541121698
2872	HSD17B10	2	1	2.862900857	1.671224332
2874	SUMO3	2	1	2.709982518	0.409580803
2876	TPRKB	2	1	2.834537152	1.136023021
2884	UFD1	2	1	3.514869813	0.155583895
2887	LEPROTL1	2	1	3.628010396	0.008093155
2891	TXNDC12	2	1	2.755962972	0.719103909
2893	RUVBL1	2	1	3.245531205	-0.297578135
2894	OLA1	2	1	3.268150929	0.371806717
2895	PFDN6	2	1	3.284914378	1.369919754
2900	PEA15	2	1	2.909728412	-0.487284222
2901	SELENOH	2	1	3.310614232	1.721207834
2902	GRPEL1	2	1	3.000996713	0.147456861
2909	PGRMC1	2	1	3.563978795	1.453700281
2915	MRPL22	2	1	3.072170381	0.904018617
2916	STUB1	2	1	2.737649564	1.268172121
2919	NXT1	2	1	2.864952449	0.503919816
2923	SNRNP27	2	1	2.802307729	0.81385181
2925	MCUR1	2	1	2.733446244	-0.005712801
2927	COX14	2	1	3.237986688	1.447156525
2929	VPS29	2	1	3.246341828	1.859325624
2949	UBE2E3	2	1	2.69656742	0.584940112
2974	SMARCB1	2	1	3.633989457	0.40625254
2975	POP7	2	1	3.126314286	0.666402674
2980	NAA20	2	1	3.442139033	0.269394374
2986	FAAP20	2	1	3.663346414	1.155551291
2989	AHSA1	2	1	2.916959647	0.819195605
2994	UBE2M	2	1	3.289576177	0.724405623
2995	HRAS	2	1	3.171928052	0.289335645
2998	DECR1	2	1	3.869147424	0.671511865
3000	CHCHD1	2	1	3.721300248	0.62498585
7	ENO1	3	1	1.365113977	-1.862490201
10	PLOD1	3	1	1.421127919	-2.184888029

16	RUNX3	3	1	2.672457103	-3.668419146
20	SFN	3	1	2.417245988	-3.025152468
37	TACSTD2	3	1	2.676390294	-3.678668284
47	LRRC8C	3	1	2.792366151	-2.82472589
51	CHI3L2	3	1	2.054675702	-3.221395993
60	S100A3	3	1	2.629467849	-2.809638762
61	S100A2	3	1	2.244953517	-2.93457725
76	ATP1B1	3	1	2.368643884	-2.659907603
83	LAMC2	3	1	2.806048278	-2.753608965
97	LAMB3	3	1	2.050830249	-3.075747752
102	AL513283.1	3	1	2.755084638	-3.491954112
107	COX20	3	1	1.21096075	-2.085303926
133	FHL2	3	1	2.760040406	-2.7693825
135	MALL	3	1	2.500713948	-2.878026747
164	SERPINE2	3	1	1.277768616	-2.491011405
165	PID1	3	1	1.366477016	-2.353130126
166	DNER	3	1	1.829804543	-3.071364665
185	CDCP1	3	1	2.274883155	-3.125097298
186	TMEM158	3	1	2.763142709	-2.599452996
204	FAM162A	3	1	1.364158753	-2.237086796
224	P3H2	3	1	2.049667481	-2.921780133
269	MSMO1	3	1	2.248132352	-2.414211773
278	AC010343.3	3	1	2.736121301	-2.772307419
303	LOX	3	1	2.05779231	-3.156673932
304	PPIC	3	1	1.11657906	-1.287520074
307	P4HA2	3	1	1.122763161	-1.842140102
309	VDAC1	3	1	1.549357299	-1.612989449
312	TGFBI	3	1	1.956165914	-2.149954342
332	PDLIM7	3	1	2.44889367	-1.985436344
343	ADTRP	3	1	2.643516902	-2.776615166
355	HMGA1	3	1	2.587051276	-3.136768603
359	TREM1	3	1	2.308788899	-2.394079232
361	VEGFA	3	1	1.960436706	-3.008037114
364	AL033397.2	3	1	2.779176358	-2.693186307
378	COL10A1	3	1	2.584889535	-3.676338219
390	PPP1R14C	3	1	2.208457831	-2.864272141
416	IGFBP3	3	1	2.359317426	-1.770469093
417	UPP1	3	1	2.446917418	-1.823076748
425	TFPI2	3	1	2.813479546	-2.815951132
431	SERPINE1	3	1	2.65749133	-2.651602768
436	FAM180A	3	1	2.458229903	-2.851446652
450	PRDX4	3	1	1.000655893	-1.325889968
459	PGK1	3	1	1.428247575	-2.141299748
481	LOXL2	3	1	2.677942876	-2.754901433
487	EIF4EBP1	3	1	2.266271237	-3.137817645
514	CA9	3	1	1.295604829	-2.441864514
540	FAM129B	3	1	1.611567382	-2.546813035
542	LCN2	3	1	2.568724994	-3.611950898
558	PHLDA2	3	1	2.483775739	-2.318192982
564	LDHA	3	1	1.111505512	-1.853070879
569	CD82	3	1	1.56531227	-2.23923161
578	AP001453.2	3	1	2.842685346	-2.740508818
585	FOSL1	3	1	2.464745406	-2.096979045
589	CCND1	3	1	2.761844758	-2.822548651
594	PRSS23	3	1	2.463981275	-2.938577675
598	MMP3	3	1	2.10803235	-3.206954979
605	MIR100HG	3	1	2.348487262	-2.402830624
628	KCNMA1	3	1	2.161284331	-3.161503338

635	PGAM1	3	1	1.638192538	-1.906482243
637	SCD	3	1	2.043400411	-2.58252933
644	ADAM12	3	1	2.695985679	-2.894121432
645	C10orf90	3	1	2.408393268	-3.053567671
647	BNIP3	3	1	1.326125268	-2.412437701
652	TPI1	3	1	1.803567294	-1.576929116
686	ITGA5	3	1	1.968416099	-2.786884331
695	HMGA2	3	1	2.34216154	-2.87707355
709	CKAP4	3	1	1.4527185	-1.553244137
716	CDK2AP1	3	1	1.217529181	-1.317347073
741	ERO1A	3	1	1.987513904	-3.108693862
742	GNPNAT1	3	1	2.811661366	-2.691285872
749	SMOC1	3	1	2.355670337	-2.546643042
763	AC022613.1	3	1	2.964495305	-2.627701067
779	CA12	3	1	1.897448424	-3.085461402
804	TNFRSF12A	3	1	2.475475434	-2.176186823
808	VKORC1	3	1	1.183351163	-1.603253626
814	MT1L	3	1	1.980821494	-3.255530142
844	AOC2	3	1	2.385741357	-2.888445639
878	P4HB	3	1	1.408078197	-1.619730854
879	SLC16A3	3	1	2.166954163	-3.009586358
880	METRNL	3	1	1.938186768	-2.931621813
887	DSC2	3	1	2.591743354	-2.821960711
888	DSG2	3	1	1.740607146	-2.779104018
898	SMOX	3	1	1.874405507	-2.247379684
900	BMP2	3	1	2.6789161	-3.230683827
904	RRBP1	3	1	1.304801468	-1.546660089
912	RPN2	3	1	1.409501795	-1.366013312
913	TGM2	3	1	2.081140164	-2.967925334
941	ANGPTL4	3	1	2.309886579	-2.188400054
966	PLAUR	3	1	2.317784194	-2.176428222
967	KCNN4	3	1	1.888268594	-2.629757428
974	IL11	3	1	2.684163455	-3.585456156
998	MRPS6	3	1	1.194237594	-2.246847534
999	HMGN1	3	1	1.808314685	-1.646250271
1017	LRRFIP1	3	1	2.131808642	-2.817392611
1043	GALNT7	3	1	2.665171508	-2.756675982
1072	SDC4	3	1	1.934829835	-2.92296555
1093	ACLY	3	1	2.136599187	-2.384423518
1099	PFKP	3	1	1.834323768	-2.962157273
1111	FERMT1	3	1	2.751966838	-2.752780222
1119	CYTOR	3	1	3.027427796	-2.483625912
1131	ABCA13	3	1	2.649266604	-3.551355385
1147	SLAMF9	3	1	2.155044679	-3.297402167
1159	RCN3	3	1	1.624101047	-1.463152551
1169	CSPG4	3	1	1.205891971	-2.909243607
1177	MIR222HG	3	1	1.83533204	-2.576713109
1223	TTC3	3	1	0.850609187	-1.563898348
1249	AL139220.2	3	1	2.042225007	-3.127431416
1272	MIR4435-2l	3	1	2.916572455	-2.602692151
1303	MAGED1	3	1	1.443040971	-1.613835001
1309	SLC9A3R2	3	1	2.617621306	-2.927633786
1321	DCLK1	3	1	2.301106099	-2.592812562
1327	MANCR	3	1	2.774305705	-2.75800445
1329	GOLM1	3	1	2.73362482	-2.647096896
1348	G0S2	3	1	2.218852405	-3.015107655
1352	STK17A	3	1	2.033263806	-2.593188548
1376	HMGCR	3	1	2.315011147	-2.461712861

1383	AL049629.2	3	1	2.687143449	-2.748060488
1439	TNFRSF10D	3	1	2.389152888	-2.900342726
1462	COL5A1	3	1	1.745559815	-2.016556525
1463	DGKI	3	1	2.703201417	-2.737224364
1465	STEAP3	3	1	1.726405744	-2.624128365
1475	PNP	3	1	2.578541879	-2.339514994
1486	CDK6	3	1	2.57174528	-2.762732529
1487	BEND6	3	1	2.823072556	-2.528046393
1498	CMIP	3	1	2.044153575	-3.027745509
1504	MIR210HG	3	1	1.109314088	-2.265333914
1519	FADS2	3	1	2.589908723	-2.75221827
1524	ENO2	3	1	1.818212394	-3.102901721
1530	QSOX1	3	1	1.337213282	-2.609872365
1535	PTGER2	3	1	2.953719739	-2.923186802
1545	PSMA6P2	3	1	2.659979466	-3.64747312
1581	TUSC3	3	1	1.365604881	-1.508179688
1613	ADGRG1	3	1	2.49440277	-2.924499297
1614	AK1	3	1	2.448169593	-2.507922673
1627	ARHGAP22	3	1	2.924345139	-2.729187035
1653	USP43	3	1	2.889611129	-2.701321625
1654	SLC5A3	3	1	1.507450704	-2.678718352
1661	SQLE	3	1	2.24085677	-2.45787909
1672	FLNA	3	1	1.818912629	-2.191389703
1681	ACOT7	3	1	2.648043994	-2.728314185
1689	ADCY7	3	1	2.442701701	-2.753345751
1691	SLC2A1	3	1	1.945073966	-3.231101775
1722	P4HA3	3	1	2.42060912	-2.375726246
1731	DARS	3	1	1.741365317	-2.95789411
1742	SORBS2	3	1	2.598631267	-2.877198958
1747	SLC38A5	3	1	2.513182286	-2.973870301
1776	APCDD1L	3	1	2.871554021	-3.137434029
1787	P3H4	3	1	1.535062555	-1.584682607
1826	AL391056.1	3	1	2.626341704	-2.362099909
1827	DAP	3	1	1.02264953	-1.388776564
1834	BPGM	3	1	2.731133107	-2.702171349
1841	HCFC1R1	3	1	1.825695638	-2.943419957
1845	IDI1	3	1	1.93889058	-2.506498122
1854	BMP6	3	1	2.051859025	-2.636249327
1878	SLC35E4	3	1	2.586089019	-2.81691482
1881	WDR1	3	1	2.728346471	-1.729416275
1886	RGS2	3	1	1.30887318	-2.717051768
1891	ELOVL1	3	1	2.880561713	-2.50137856
1896	RFLNB	3	1	2.767282132	-3.436935925
1920	C3orf52	3	1	2.701218728	-2.463946127
1921	PGM1	3	1	1.645769242	-2.494643235
1923	APCDD1L-[illegible]	3	1	2.875710849	-3.151783728
1929	MSANTD3	3	1	2.524610166	-2.330626869
1931	AL845321.1	3	1	2.578676824	-3.633714222
1940	RBMS1	3	1	2.204913262	-1.654463076
1944	ZNF385A	3	1	2.187524203	-2.815843844
1945	PARVB	3	1	2.723092202	-2.818086886
1952	TGFBR1	3	1	1.354068402	-2.529348635
1961	CITED4	3	1	1.298225645	-2.948794388
1980	SLC7A5	3	1	2.342688922	-2.30364027
1990	IL1RL1	3	1	2.478587274	-1.377842688
1998	HIPK2	3	1	2.598636512	-2.697612547
2003	PDGFC	3	1	2.650301579	-2.58779695
2015	FKBP10	3	1	1.3718195	-1.668656969

2026	PTGS2	3	1	1.926901463	-3.100832724
2034	MYOT	3	1	1.563518409	-2.333749079
2036	GARS	3	1	2.827201251	-2.187309885
2049	NRN1	3	1	1.677653674	-3.00495603
2108	ADPRHL1	3	1	1.799721364	-2.494186902
2123	ALDOC	3	1	1.279278759	-2.633540654
2126	ABLIM3	3	1	2.62088478	-2.686527514
2128	C19orf33	3	1	2.692276601	-2.753175282
2147	HMGCS1	3	1	1.96505535	-2.276867532
2149	GPI	3	1	1.92445052	-3.011252665
2160	ACAT2	3	1	2.785393599	-2.691348338
2177	AC002454.1	3	1	2.845482472	-2.688538813
2180	PCDH10	3	1	2.429758672	-3.037989163
2201	AGTRAP	3	1	2.53974903	-2.183462762
2203	PLEC	3	1	1.318426732	-2.601899647
2209	LDLR	3	1	1.726562623	-2.213824653
2218	MTHFD1L	3	1	2.589055184	-2.557298207
2242	AK4	3	1	1.827664737	-2.578082346
2245	STX1A	3	1	2.610983018	-2.733537459
2262	AC007686.1	3	1	2.71309555	-3.575339579
2275	SH3KBP1	3	1	2.445080403	-2.498116278
2279	EMID1	3	1	2.720124606	-3.723828101
2297	ELK3	3	1	2.497596148	-2.388087534
2319	TMEM165	3	1	2.583985928	-1.512524747
2329	RCAN1	3	1	1.925992135	-2.43420484
2341	FURIN	3	1	1.427775506	-2.563594126
2368	SPAG4	3	1	1.799756411	-3.150264763
2382	ARF6	3	1	2.671940688	-2.20726552
2401	FSCN1	3	1	1.604349259	-1.722502493
2413	TMOD3	3	1	2.371771459	-1.865759277
2426	PKP1	3	1	2.647223357	-2.734930777
2429	ARNTL2	3	1	2.76390231	-2.621629262
2441	SNAPC1	3	1	1.114241485	-2.419318223
2447	SPINK1	3	1	2.696046714	-3.6107445
2452	SMS	3	1	2.682748441	-2.145617151
2458	GLTP	3	1	2.573235873	-2.933085465
2487	TRIM47	3	1	2.437631492	-2.938918376
2488	COL7A1	3	1	2.524677876	-3.029042983
2492	IGF2BP2	3	1	2.871936921	-3.04771974
2500	VASP	3	1	2.512132529	-2.850688719
2514	RNF145	3	1	2.493543748	-2.437535786
2516	NPTN	3	1	1.26888812	-1.545172953
2521	FABP3	3	1	1.877091054	-2.547226214
2525	PLOD3	3	1	1.445075396	-1.997640395
2527	YKT6	3	1	2.653845433	-1.662363195
2543	PLEK2	3	1	2.65991867	-3.632499718
2549	PYGL	3	1	1.71834505	-2.963936114
2559	GCLM	3	1	2.596452121	-2.067681694
2573	ASAH1	3	1	0.821478847	-1.55907073
2592	DHCR7	3	1	2.622519854	-2.76918962
2602	B4GALT2	3	1	1.545766477	-1.751887583
2609	BICDL2	3	1	1.809589032	-3.121304297
2628	SHMT2	3	1	1.786567334	-3.058473372
2629	YIF1B	3	1	2.549307946	-2.262682819
2640	PLA2G4A	3	1	1.881976012	-2.932445788
2657	DIXDC1	3	1	2.742910031	-2.676982903
2670	ITPR3	3	1	2.232836846	-2.955992245
2674	CYP27C1	3	1	2.059957866	-2.531882309

2681	PPP2CB	3	1	2.413357381	-1.906740093
2687	TES	3	1	2.336136226	-2.751936936
2695	POPDC3	3	1	2.71199644	-2.707427287
2705	EHD1	3	1	2.492189292	-3.076680445
2706	TMCO3	3	1	2.335690621	-2.793963933
2714	RABGGTB	3	1	1.822174672	-2.996871018
2715	BMP1	3	1	2.509127025	-1.830715441
2724	TBX3	3	1	2.67111576	-2.116882824
2735	GBE1	3	1	1.755974654	-3.059204363
2739	EVA1A	3	1	2.950010423	-2.959748053
2741	ABHD2	3	1	2.441105727	-2.46667149
2756	AC105383.1	3	1	2.588546876	-2.790681862
2781	AC106786.1	3	1	2.732922677	-3.657041573
2796	PXDN	3	1	2.558119897	-2.701643014
2809	NT5DC2	3	1	2.64719284	-2.479796671
2816	GALNT2	3	1	1.437565807	-2.212282562
2817	MGAT4B	3	1	2.210121755	-2.561975026
2827	SLC16A1	3	1	1.693843726	-2.52590444
2835	FDPS	3	1	2.919299725	-2.552216315
2840	PC	3	1	1.491512541	-2.8173702
2852	PTPN12	3	1	2.001702908	-1.434439206
2871	MBD2	3	1	2.821959142	-2.98052051
2875	CMTM3	3	1	2.625837449	-1.43086996
2881	SPRY2	3	1	2.011152152	-3.216657662
2932	TSPAN2	3	1	1.967145566	-2.421164298
2973	MAD1L1	3	1	1.777563695	-3.111110711
2997	NRIP1	3	1	2.02231491	-1.599689149
3	MXRA8	4	1	-2.739514705	-1.60101881
13	PLA2G2A	4	1	-2.608675595	-0.469812953
34	PODN	4	1	-3.089282151	-0.357459494
39	NFIA	4	1	-3.199606772	-0.796019995
44	PTGFR	4	1	-3.095420714	-0.113304877
48	CNN3	4	1	-3.327309962	-1.370205783
52	OLFML3	4	1	-3.319830533	-1.382863545
68	IFI16	4	1	-3.023939248	-0.28013922
75	DPT	4	1	-2.294861313	-0.749129438
78	PRRX1	4	1	-3.118470307	-0.093791851
82	LAMC1	4	1	-2.819760676	-0.131941074
85	PRG4	4	1	-1.574195381	-0.832723373
117	LTBP1	4	1	-3.562390204	-1.120389306
124	EFEMP1	4	1	-3.117654201	-0.027620309
126	ANTXR1	4	1	-2.97050273	-1.139731311
132	IL1R1	4	1	-2.394902583	-0.34039513
144	DPP4	4	1	-1.692306157	-0.915474319
145	FAP	4	1	-1.648859497	-0.599190914
174	SNED1	4	1	-2.224570986	-0.234322467
177	FBLN2	4	1	-2.563737031	-0.400303536
179	RBMS3	4	1	-3.479194757	-1.638451957
195	MITF	4	1	-3.034738895	-0.410990463
196	FOXP1	4	1	-2.907370444	-1.019285881
201	CCDC80	4	1	-2.758764144	-0.474446618
202	FSTL1	4	1	-1.917497512	-1.117886507
208	ACKR4	4	1	-2.920222875	-0.608619713
212	SLC9A9	4	1	-2.928281661	-1.309867525
214	ZIC1	4	1	-1.856046196	-1.783279562
215	CPB1	4	1	-2.588601466	-0.557988414
218	SSR3	4	1	-1.900565024	-2.19773128
234	HTRA3	4	1	-2.372894403	-0.253460282

243	PDGFRA	4	1	-2.861516591	-1.21863445
250	HSD17B11	4	1	-3.181838389	-0.618985587
261	USP53	4	1	-2.18654513	-2.292670869
264	PCDH18	4	1	-3.208064195	-0.131939226
267	SFRP2	4	1	-2.704837915	-0.590154552
275	RETREG1	4	1	-2.242443796	-1.706225299
276	BASP1	4	1	-2.481868383	-0.298217272
280	DAB2	4	1	-2.996334907	-0.462122434
301	CDO1	4	1	-1.988341685	-1.759123468
311	CXCL14	4	1	-3.16425645	-0.147651889
324	EBF1	4	1	-3.036450263	-0.458064654
351	TNXB	4	1	-2.249052282	-0.554624804
372	PNRC1	4	1	-1.678717848	-1.2664034
377	MARCKS	4	1	-3.492623445	-1.191787743
380	MAN1A1	4	1	-2.77686226	-0.319838486
384	EPB41L2	4	1	-2.969127294	-1.308592104
385	ENPP1	4	1	-1.989034887	-2.606796526
389	SASH1	4	1	-3.066410895	-0.479697847
391	AKAP12	4	1	-3.606571075	-1.056580984
392	SNX9	4	1	-3.079412337	-0.58634653
403	TWIST1	4	1	-3.310911771	-1.312349581
405	GPNMB	4	1	-2.957952376	-0.902298772
411	SFRP4	4	1	-3.053177472	-0.879441642
421	FGL2	4	1	-3.11914384	-0.741784477
423	SEMA3E	4	1	-1.952599879	-2.275759124
429	BRI3	4	1	-1.996547338	-1.502125287
477	PDGFRL	4	1	-3.129200812	-0.978101217
486	SCARA5	4	1	-2.261806484	-0.482291215
488	SFRP1	4	1	-2.37503707	-0.272412398
489	CEBPD	4	1	-1.848441359	-1.532893204
508	COL14A1	4	1	-3.055597182	-0.700123691
512	NFIB	4	1	-3.130013581	-0.864570641
525	ECM2	4	1	-2.062993761	-2.193507695
528	COL15A1	4	1	-2.694821712	-0.591336005
571	FAM180B	4	1	-3.132867213	-1.398203754
597	ANGPTL5	4	1	-1.848455425	-2.166237258
599	PDGFD	4	1	-3.267407294	-1.384445571
608	AKR1C1	4	1	-1.561794396	-0.596191251
609	CELF2	4	1	-2.626041051	-0.349485918
618	BICC1	4	1	-2.879616376	-0.914131903
620	ARID5B	4	1	-3.036978837	-0.470281744
636	CRTAC1	4	1	-1.442433294	-1.029443526
640	ABLIM1	4	1	-2.596181031	-0.524249935
648	CD9	4	1	-2.299099442	-1.340579295
654	C1S	4	1	-2.775801536	-0.980011963
655	C1R	4	1	-2.888265725	-0.964746975
657	MFAP5	4	1	-2.030620214	-0.660088384
662	RERG	4	1	-2.388396736	-1.499286198
680	METTL7A	4	1	-3.05947816	-1.623189592
704	NTN4	4	1	-1.744439956	-0.822756403
706	HELLPAR	4	1	-2.914296266	-1.044005298
707	IGF1	4	1	-2.841522809	-0.880276286
726	LHFPL6	4	1	-3.19845688	-1.405787849
747	ZFP36L1	4	1	-3.091144677	-0.393814602
751	NPC2	4	1	-1.802747961	-2.08884921
752	LTBP2	4	1	-1.890800592	-2.448247218
756	FBLN5	4	1	-3.238732692	-0.690718495
760	SYNE3	4	1	-2.824347611	-0.271297128

770	FBN1	4	1	-2.015529033	-0.611106628
807	NUPR1	4	1	-2.167294975	-1.495549225
810	IRX3	4	1	-2.196246024	-0.207925865
811	MMP2	4	1	-2.722554561	-0.937540853
815	MT1E	4	1	-2.019245978	-1.978633546
816	MT1M	4	1	-2.417599436	-1.815849447
817	MT1G	4	1	-1.720937487	-1.99814918
829	CRISPLD2	4	1	-2.832479592	-0.551908874
832	SERPINF1	4	1	-3.213782426	-1.110539758
837	PMP22	4	1	-2.025176045	-1.335336768
839	MFAP4	4	1	-2.988006469	-1.11591103
851	COL1A1	4	1	-3.470933553	-1.212381208
881	COLEC12	4	1	-3.323724862	-0.60603311
911	PROCR	4	1	-1.655293342	-0.863241815
917	SLPI	4	1	-2.041543957	-2.049173855
919	PLTP	4	1	-2.737994548	-0.341899352
931	CFD	4	1	-2.831496354	-0.289123681
940	C3	4	1	-2.921738502	-0.378509288
954	CRLF1	4	1	-1.327351626	-0.813590908
983	TIMP3	4	1	-2.158576485	-1.281280183
991	FBLN1	4	1	-3.018503543	-1.153578007
1031	KRT222	4	1	-2.778584357	-0.344232852
1036	MAP1A	4	1	-2.750024434	-0.373109725
1040	BOC	4	1	-3.134765025	-0.315793249
1060	PLEKHH2	4	1	-3.041941281	-0.979150021
1081	CD302	4	1	-3.436862823	-0.48799839
1082	YPEL3	4	1	-2.183993574	-1.579499864
1085	REV3L	4	1	-2.631267425	-1.649397516
1100	PDE1A	4	1	-2.481090184	-1.336162471
1105	NCOA7	4	1	-2.624891397	-0.60478335
1139	APBB1IP	4	1	-1.68387806	-0.720833623
1149	RF00100.4	4	1	-3.783015605	-1.177703583
1150	IL6ST	4	1	-2.73003399	-1.213716769
1167	AFF3	4	1	-2.128199454	-2.40756967
1203	MT1F	4	1	-1.953183409	-1.994841122
1222	GLUL	4	1	-2.680402871	-1.37212243
1253	REXO2	4	1	-1.897627588	-1.748741173
1278	SERINC1	4	1	-1.943446275	-1.265983963
1284	RUNX1T1	4	1	-3.658164617	-0.423840099
1293	GALNT15	4	1	-1.545450803	-1.031610155
1295	CPED1	4	1	-3.437420722	-1.377151632
1314	DEFB1	4	1	-1.627696391	-0.897763156
1343	NDNF	4	1	-3.062532064	-1.435939693
1372	AGTR1	4	1	-2.825046655	-0.393240241
1375	ASPH	4	1	-3.114633676	-1.459955358
1378	ATP6AP2	4	1	-2.931875106	-1.175883972
1428	SH3BP5	4	1	-2.635206576	-1.510548377
1453	PLSCR4	4	1	-3.438208695	-0.640958392
1470	NHSL2	4	1	-3.448564168	-0.643712723
1511	BTG1	4	1	-1.634995457	-1.26351937
1531	TMEM100	4	1	-2.317182776	-0.418105834
1548	GBP2	4	1	-2.48985922	-1.435458684
1550	FAM129A	4	1	-3.048540469	-1.328335428
1555	ID3	4	1	-2.831727143	-1.328900659
1557	LRRK2	4	1	-3.120819207	-1.235642099
1564	HP1BP3	4	1	-2.832270022	-1.320340597
1566	SYNE2	4	1	-3.584761735	-1.378111743
1567	CAST	4	1	-2.569289561	-1.222761714

1572	VASN	4	1	-2.172702786	-2.03587749
1578	NDFIP1	4	1	-2.666049596	-1.289346242
1580	UBE2B	4	1	-2.07860994	-1.431138777
1617	EPB41L3	4	1	-1.925838824	-0.655740106
1644	XAF1	4	1	-3.287159082	-0.956181669
1647	AP001528.2	4	1	-3.282034274	-0.168703222
1650	CAMK2D	4	1	-3.116494056	-0.216964723
1713	FYN	4	1	-2.291624185	-0.435149008
1729	VEGFB	4	1	-2.273636099	-1.508255624
1733	NOVA1	4	1	-1.637567397	-0.926072919
1739	HLA-E	4	1	-2.335380908	-1.55034759
1791	DEPP1	4	1	-2.938377734	-0.405100078
1795	H2AFJ	4	1	-1.943013664	-1.624759936
1809	CDON	4	1	-2.721712943	-0.717769557
1825	C9orf3	4	1	-2.321532246	-2.239586138
1846	MAP3K20	4	1	-2.346261736	-0.542435327
1848	SDCBP	4	1	-3.473507997	-0.37690894
1851	SULF2	4	1	-2.409822699	-1.006762409
1853	RIN2	4	1	-2.837648507	-1.408884429
1865	SLFN5	4	1	-2.274180051	-0.219248989
1877	CFLAR	4	1	-2.879233476	-1.524424099
1894	ZNF704	4	1	-2.261222836	-2.077060365
1914	MTSS1	4	1	-3.826467391	-1.586726212
1919	APOL6	4	1	-2.999270793	-0.128414684
1942	AKR1C2	4	1	-1.649566647	-0.908623182
1967	SGCE	4	1	-3.172139522	-0.91213485
2005	ADGRD1	4	1	-4.073696014	-1.364313983
2016	DPYSL2	4	1	-2.550899383	-1.429857039
2058	GNG12	4	1	-3.509876367	-0.594209188
2073	JAK1	4	1	-2.993456002	-0.589937889
2077	LAMP2	4	1	-2.813138839	-0.72671085
2081	CADM3	4	1	-1.900807854	-0.754533523
2097	BDH2	4	1	-2.904056426	-1.275327408
2103	TSPAN4	4	1	-2.044649597	-1.636855745
2115	OLFML1	4	1	-3.340274688	-0.727714771
2120	PLPP1	4	1	-2.042917963	-2.100885534
2122	ADH5	4	1	-3.188517924	-1.472925329
2135	SLC25A37	4	1	-1.291218873	-0.993859255
2150	SCX	4	1	-1.964313146	-2.152439856
2155	NAV1	4	1	-1.930084821	-0.597464197
2169	PCDH9	4	1	-3.102296945	-1.520160102
2185	APP	4	1	-1.970171209	-1.29596492
2194	RGL1	4	1	-3.372645732	-1.305229866
2229	BLVRB	4	1	-2.002402183	-1.505129361
2241	CALCOCO1	4	1	-2.955953952	-1.45311191
2251	ZFHX4	4	1	-2.989282247	-1.31371423
2290	CERCAM	4	1	-2.142096277	-0.597012364
2315	ANG	4	1	-2.079833861	-1.525660538
2334	LY96	4	1	-2.705750342	-0.849629545
2336	CREBRF	4	1	-2.490431663	-1.133560741
2362	LRRN4CL	4	1	-3.563751336	-1.394575977
2372	HSD11B1	4	1	-2.389903899	-0.137062722
2373	RTN3	4	1	-2.418141719	-1.636701846
2378	ANK2	4	1	-2.846751567	-1.008884275
2405	RBPJ	4	1	-2.980858441	-0.831557893
2409	SGCD	4	1	-3.213328239	-0.268144311
2420	ADAM9	4	1	-1.924863454	-0.750402921
2423	LPAR1	4	1	-2.610835668	-0.189771989

2425	CD34	4	1	-2.499465343	-0.538770058
2445	MPZL1	4	1	-2.892374869	-0.653839314
2446	TGFBR3	4	1	-2.067136642	-2.021596336
2456	DOCK11	4	1	-2.646063682	-1.456424737
2478	PSIP1	4	1	-3.735570546	-0.489249074
2502	SCARB2	4	1	-2.689781066	-1.042475724
2512	HOXA10	4	1	-1.872149941	-2.121266388
2518	EZH1	4	1	-2.902508374	-0.954338514
2530	TACC1	4	1	-3.375097152	-1.581763172
2531	SNTB2	4	1	-2.995216008	-1.392687702
2537	PLSCR1	4	1	-3.016207572	-0.220611536
2588	GLT8D2	4	1	-3.389011737	-0.747513347
2589	PALMD	4	1	-1.26737773	-0.884218239
2608	CTSA	4	1	-2.332998272	-1.351796054
2623	GALNT13	4	1	-1.605088588	-0.918822908
2631	GNS	4	1	-2.406213876	-0.907759809
2633	ATL3	4	1	-2.052145477	-1.54554584
2686	CCNG2	4	1	-2.436988231	-1.45319345
2689	RAB3IL1	4	1	-2.967080947	-0.927653217
2692	PTGFRN	4	1	-2.382076856	-0.300505159
2698	SAT2	4	1	-2.195226308	-1.671288991
2700	KLHL24	4	1	-2.977916118	-0.6579104
2708	FOXN3	4	1	-2.679118987	-0.726133966
2712	RNF19A	4	1	-3.005604383	-1.065268123
2713	STOM	4	1	-3.342664357	-0.96074953
2742	SVIL	4	1	-3.295631524	-1.63726654
2766	BMPER	4	1	-2.988163348	-0.25639806
2787	SMARCE1	4	1	-3.083579894	-0.616763794
2799	CDKN1B	4	1	-2.667668458	-1.245526158
2806	CLEC3B	4	1	-1.874265548	-0.850572967
2829	PCMTD1	4	1	-2.803712007	-1.422709965
2830	ADAM22	4	1	-2.831929561	-0.666227364
2836	ADGRG2	4	1	-2.314693924	-2.210606956
2845	DIAPH2	4	1	-2.920480844	-0.313558185
2865	SP110	4	1	-2.632350322	-0.299801898
2880	STAG2	4	1	-2.135129329	-1.277478182
2890	MBNL2	4	1	-2.086486217	-2.149114632
2913	ADAMTS16	4	1	-1.711768027	-0.855085277
2941	ANGPTL1	4	1	-2.766872521	-0.678712123
2943	PHF3	4	1	-2.41011345	-1.182000184
2948	TBC1D2B	4	1	-3.063266393	-0.462350019
2976	AL110292.1	4	1	-1.61164796	-0.920081698
2983	EFNA5	4	1	-2.960866328	-0.189744943
2990	ANKRD28	4	1	-1.162071701	-1.226032459
2	SDF4	5	1	-0.192558672	-1.968392753
19	SH3BGRL3	5	1	0.602256898	-0.197598138
35	TMEM59	5	1	0.08617479	-1.126454258
59	S100A4	5	1	-0.077165958	-0.666208678
62	S100A13	5	1	-0.175372418	-1.401145839
65	LMNA	5	1	0.947075967	0.06849984
92	FMOD	5	1	-0.908053454	-2.526095414
93	PRELP	5	1	-1.125914451	-2.354220414
98	SERTAD4-A	5	1	0.138357762	-1.260781431
103	HHIPL2	5	1	-0.423364602	-2.920141959
115	ATRAID	5	1	0.431516651	-1.217957937
125	UGP2	5	1	-0.74443775	-2.441720032
147	CYBRD1	5	1	-1.448178228	-1.616000557
152	COL3A1	5	1	-0.632026162	-1.570219063

158	BZW1	5	1	0.487935547	-0.540032559
180	TGFBR2	5	1	-1.441288944	-1.926349186
194	ARL6IP5	5	1	0.021889064	-0.733272665
199	ABI3BP	5	1	-0.952364709	-2.58240273
210	PCOLCE2	5	1	-0.548105594	-1.775501394
222	GOLIM4	5	1	0.660946015	-0.864333534
236	NKX3-2	5	1	-0.538583491	-2.911662125
240	UGDH	5	1	-0.359709907	-0.593930298
270	CPE	5	1	-0.271659058	-1.980142736
294	VCAN	5	1	-0.716628965	-2.234421396
322	GPX3	5	1	-1.16389155	-2.243801498
337	FOXC1	5	1	-0.58532853	-2.498552584
348	HLA-C	5	1	-0.394920226	-1.601738476
370	NT5E	5	1	-0.473184522	-0.663555556
412	INHBA	5	1	-0.649546813	-2.929256224
414	TMED4	5	1	0.286989037	-1.390958809
426	COL1A2	5	1	-0.845798042	-1.832964921
433	CAV1	5	1	-0.278733399	-0.835421586
446	CD99	5	1	-0.092964109	-0.912236893
453	PLP2	5	1	-0.166695859	-0.730126792
471	FHL1	5	1	-1.114165243	-2.416293168
474	BGN	5	1	-0.660870608	-2.208842659
483	CLU	5	1	-1.12791359	-2.180608058
504	TRPS1	5	1	-0.737704422	-2.588680768
506	TNFRSF11B	5	1	-0.507786922	-1.819922351
522	OGN	5	1	-0.790887322	-2.709190392
546	ASS1	5	1	-0.183256533	-2.059698367
556	CD81	5	1	-0.666825857	-1.469807767
572	SERPING1	5	1	-1.581861254	-1.599065208
583	LTBP3	5	1	-0.975375649	-1.586024904
600	CRYAB	5	1	-1.157270666	-2.040885949
601	NNMT	5	1	-1.175263163	-2.347573065
611	PLXDC2	5	1	-0.809495624	-2.611100935
622	PSAP	5	1	-0.990724858	-1.830129289
629	PLAC9	5	1	-0.79603776	-1.55373373
634	RBP4	5	1	-0.093061712	-1.691115164
641	ATRNL1	5	1	-0.534172233	-2.886280798
642	HTRA1	5	1	-0.635534938	-1.991391682
670	BCAT1	5	1	-1.162978705	-1.454175019
672	SSPN	5	1	-0.010681357	-1.241001451
691	LRP1	5	1	-1.012477692	-1.730668926
693	OS9	5	1	-0.351928968	-2.002106452
702	LUM	5	1	-0.8726657	-1.92520585
703	DCN	5	1	-1.04276013	-2.054011249
729	ITM2B	5	1	0.271755401	-0.289223758
775	ANXA2	5	1	0.822135452	-0.025067055
787	ISLR	5	1	-0.540203866	-1.91584959
818	MT1X	5	1	-1.180743095	-2.268798732
823	GABARAPL2	5	1	0.322109882	-1.349057102
848	COPZ2	5	1	0.393721227	-1.109764063
857	MRC2	5	1	-0.827530917	-1.922907614
876	TIMP2	5	1	-1.112773653	-1.724874758
903	DSTN	5	1	-0.126927134	-1.16632744
905	PAX1	5	1	-0.713500973	-2.801709675
907	CST3	5	1	-0.895611759	-1.519470834
926	GNAS	5	1	0.007816438	-0.873180353
937	NRTN	5	1	-0.332358759	-2.887596631
955	COMP	5	1	-1.124137696	-2.360852742

957	FXYD5	5	1	-0.242677789	-0.869882905
965	RABAC1	5	1	-0.045132395	-1.252190852
972	EMP3	5	1	0.808217529	-0.07039299
989	TSPO	5	1	-0.231142055	-1.495937132
1000	CSTB	5	1	0.624717955	-0.087467336
1001	COL6A2	5	1	-0.306437802	-1.921749138
1027	YWHAB	5	1	1.020615462	-0.171291971
1038	PCOLCE	5	1	-0.556391243	-1.517090344
1042	FGFR2	5	1	-0.468332545	-2.984172606
1049	PIGP	5	1	0.623198632	-1.34085896
1056	TENT5A	5	1	-1.143904146	-2.641844058
1058	HS6ST3	5	1	-0.632786822	-2.866749548
1059	MSN	5	1	-0.227046665	-0.163841092
1087	ARFGEF3	5	1	-0.523625176	-3.004588866
1120	ANXA4	5	1	0.050172512	-1.1315858
1179	LRPAP1	5	1	0.120014433	-1.43031826
1186	CYB5R3	5	1	-0.561439846	-1.56116774
1195	EZR	5	1	0.191253189	0.029900259
1245	CPQ	5	1	-0.139755603	-1.503283166
1247	LEPROT	5	1	0.517705027	-0.686298126
1248	CLCF1	5	1	0.14683247	-0.523521864
1259	GNAI2	5	1	-0.132369574	-1.45365026
1263	CLIC4	5	1	0.508349244	-0.506377348
1277	PTTG1IP	5	1	-0.315468352	-1.940600538
1292	STK32A	5	1	-0.520874102	-3.084278369
1315	GLS	5	1	-0.72788712	-2.930207276
1316	FNDC3B	5	1	-0.483531173	-2.278791093
1328	FKBP7	5	1	0.535705749	-1.228287184
1332	GLCCI1	5	1	-0.510374662	-2.895911717
1341	ITGB8	5	1	-0.616658878	-2.976943039
1362	HLA-B	5	1	-0.443860551	-1.665643715
1364	PLIN3	5	1	0.51453406	-0.333754442
1366	HLA-A	5	1	-0.439663297	-1.730333113
1368	SLC38A1	5	1	-0.662188347	-2.770601534
1382	CD109	5	1	-0.256156441	-2.100279235
1392	BAALC	5	1	-0.227310296	-3.033656144
1397	NORAD	5	1	1.00468934	-0.837974631
1409	RUNX1	5	1	-0.957004007	-0.967240953
1413	SGCB	5	1	0.639458183	-0.749290549
1445	CD151	5	1	0.782446388	-0.411933617
1452	GAS6	5	1	-0.80062693	-1.96488347
1464	NFIX	5	1	-0.724566814	-1.884230518
1466	NFIC	5	1	-0.682758387	-1.251716756
1472	RDX	5	1	-0.536714356	-2.77218368
1515	GFPT2	5	1	-0.843644943	-0.992444837
1551	PLD3	5	1	-0.765976962	-1.926482462
1571	HSD3B7	5	1	-0.274984237	-0.720348799
1583	CAVIN1	5	1	-0.356946107	-1.448291206
1588	AHNAK	5	1	-0.535246234	-1.910213613
1591	PCBP2	5	1	-0.113747235	-0.894893252
1596	CTSF	5	1	-1.424911376	-1.695347094
1602	MXRA7	5	1	0.000787649	-0.777325534
1655	CKB	5	1	-0.578497726	-1.590304875
1657	ITGB5	5	1	-0.672477376	-1.377609753
1670	CBX5	5	1	0.90912259	-0.680664622
1676	YWHAH	5	1	0.31596506	-0.350904407
1698	PCBP1	5	1	-0.158290651	-1.897600793
1704	FCGRT	5	1	-0.51311307	-1.418224716

1714	NUCB1	5	1	-0.20213991	-1.875154161
1748	RASSF8	5	1	0.425893907	-0.487423354
1764	BSG	5	1	-0.651480179	-1.571433448
1766	ABHD14A	5	1	0.121540073	-1.59843316
1790	LIMS1	5	1	-0.3919526	-0.392069222
1799	IQGAP1	5	1	-1.233955976	-2.724956774
1801	LAMP1	5	1	-0.725584861	-1.891043806
1806	FAM3C	5	1	0.875421766	-0.827800536
1815	RECQL	5	1	0.164602343	-0.477771857
1823	NDRG1	5	1	0.310122017	-0.342375581
1875	CMTM6	5	1	1.098988775	-0.321623338
1905	RBMX	5	1	-0.606023621	-3.125206971
1954	MVP	5	1	-0.316567223	-0.303503593
1962	DTNA	5	1	-0.519699909	-3.0418823
1976	CRIP2	5	1	-0.118273821	-1.489004159
2012	CTSD	5	1	-0.705985915	-1.922855281
2027	ID2	5	1	-1.164477762	-1.554855966
2033	YWHAZ	5	1	0.771457437	-0.163468653
2050	LAG3	5	1	-1.097735997	-2.406273388
2092	FAM20C	5	1	-1.006805297	-1.658998751
2117	CRIP1	5	1	-0.498198364	-1.259493434
2156	IFNAR1	5	1	0.619470004	-0.304899573
2159	SERINC3	5	1	-1.631161686	-1.363338255
2173	EMILIN1	5	1	-0.337010827	-1.391994381
2189	DPP7	5	1	-0.640147325	-1.90971806
2190	WARS	5	1	0.500704233	-0.599728637
2197	RRAS	5	1	-0.409823552	-1.192559921
2200	OXR1	5	1	-0.522238057	-2.877556586
2202	PDE4B	5	1	0.568035249	-0.77300986
2216	CTSB	5	1	-0.85003927	-1.777344369
2255	JADE1	5	1	-0.58204614	-2.935055518
2302	WNK4	5	1	-0.329733085	-3.002127432
2312	DYNLT3	5	1	-0.740297403	-1.321162605
2318	TM2D1	5	1	0.323733691	-1.109966421
2343	GNB2	5	1	0.722586993	-0.279901133
2402	CNIH1	5	1	0.135875288	-0.337539635
2411	FAM89B	5	1	-0.007362243	-0.214441695
2414	SUN2	5	1	-0.877904084	-2.391026282
2419	BCAP29	5	1	0.836105112	-0.719644928
2453	ARL2BP	5	1	0.627063517	-1.183456921
2467	GLG1	5	1	-0.768971916	-1.892836713
2498	PRUNE2	5	1	-0.779220726	-2.486894393
2515	CTSZ	5	1	-0.360591788	-1.321413779
2545	NOTCH2	5	1	-0.950463291	-1.478968644
2558	EFEMP2	5	1	0.416262034	-1.337185883
2566	SUMF2	5	1	-0.226002213	-1.74744966
2587	TSPYL1	5	1	-0.411938872	-2.75163176
2651	RTL8C	5	1	0.861712936	-0.048324728
2655	CASC4	5	1	-0.117525008	-1.535749578
2656	LAMB2	5	1	-0.78247967	-1.883486294
2665	AXL	5	1	-0.434032459	-0.746630215
2684	RNH1	5	1	0.688467506	0.101225561
2694	ERGIC1	5	1	-0.510966651	-1.901318693
2702	AHNAK2	5	1	-1.217317279	-1.29184463
2720	ADI1	5	1	-1.47024655	-1.720222377
2737	PAM	5	1	-1.415080126	-1.493186855
2758	SMARCA1	5	1	0.774188999	-1.011296653
2761	DESI2	5	1	1.029926781	-0.067262613

2794	GLUD1	5	1	0.524432901	-0.275080801
2859	KLHL2	5	1	-0.455880339	-2.796758675
2903	ANK3	5	1	-1.100784179	-1.356956982
2912	DPYSL3	5	1	-0.785328295	-2.050720596
2920	TMEM98	5	1	0.251623336	-1.261280798
2924	PCYOX1	5	1	-0.497020252	-1.76512494
2926	CHPT1	5	1	0.236964647	-1.353173041
2934	DDAH2	5	1	-0.615020972	-1.152037286
2988	CTNNA1	5	1	0.18117911	-0.495973447
27	BMP8B	6	1	-5.591784354	0.018895036
40	L1TD1	6	1	-5.334185	-0.210189023
86	KCNT2	6	1	-3.942690249	0.37721119
87	CFH	6	1	-3.983405467	0.230336643
118	VIT	6	1	-4.869608279	-0.080523544
119	CYP1B1	6	1	-4.452399846	-0.033525908
122	SPTBN1	6	1	-4.147572156	-0.183645361
137	SLC20A1	6	1	-5.104931708	-0.264594519
148	CHN1	6	1	-5.472000476	-0.201597803
150	NFE2L2	6	1	-4.638832923	-0.098326647
151	TFPI	6	1	-3.99607956	0.30699364
159	NRP2	6	1	-5.340767737	-0.067549014
197	EPHA3	6	1	-3.922237512	0.236581421
209	MRAS	6	1	-5.412603732	0.053744054
225	CLDN1	6	1	-5.221707221	0.137439079
227	APOD	6	1	-4.639984008	0.283014036
241	SHISA3	6	1	-5.191951629	0.179626799
246	ART3	6	1	-5.713674422	-0.000284963
251	SPARCL1	6	1	-4.146000739	0.451323247
268	GUCY1B1	6	1	-4.690469619	0.28647635
288	MAP1B	6	1	-4.223180171	0.378577626
298	MCTP1	6	1	-5.577375289	0.123128272
305	SLC12A2	6	1	-5.617003795	-0.059831911
317	SPRY4	6	1	-5.374968883	-0.290867229
327	TENM2	6	1	-5.485701915	-0.049528026
358	PI16	6	1	-4.969035502	-0.089124614
376	LAMA4	6	1	-4.088857289	0.210435367
383	LAMA2	6	1	-4.087703343	0.018271721
393	SLC22A3	6	1	-5.244802829	0.09674365
400	ETV1	6	1	-5.175384875	0.026219106
401	MEOX2	6	1	-4.620211955	0.082465148
402	AHR	6	1	-4.254615422	0.289513445
419	EGFR	6	1	-5.129835959	-0.111855515
432	LAMB1	6	1	-4.132280942	0.133982933
442	RARRES2	6	1	-4.314268228	0.257524944
457	EFNB1	6	1	-5.398918983	-0.27475657
476	DLC1	6	1	-4.820594188	-0.088483819
479	LZTS1	6	1	-5.465606566	-0.056540364
482	EBF2	6	1	-4.978969451	0.058157987
537	GSN	6	1	-4.170573588	-0.373221976
610	FRMD4A	6	1	-5.572313663	-0.132766658
638	ADD3	6	1	-4.359697934	-0.256401234
656	SLC2A3	6	1	-4.13422906	0.359954811
658	A2M	6	1	-4.476063605	0.342435039
666	SLCO1C1	6	1	-5.459056254	0.113464958
698	PHLDA1	6	1	-5.543940898	-0.170922705
700	DUSP6	6	1	-5.314749595	-0.38431703
719	GJB2	6	1	-5.461197253	-0.095086613
722	TNFRSF19	6	1	-5.559440013	-0.02281838

755	FLRT2	6	1	-4.334488984	0.235044635
789	PEAK1	6	1	-5.342093345	0.055874294
800	NR2F2	6	1	-4.290493842	0.33481232
802	AC009041.2	6	1	-5.443677779	-0.360473189
812	LPCAT2	6	1	-5.533455726	-0.035215014
827	WFDC1	6	1	-5.418913241	0.122165686
840	PLXDC1	6	1	-5.120390292	-0.156944879
847	FZD2	6	1	-5.414418574	-0.238132433
849	NGFR	6	1	-4.673886653	0.314437068
858	FAM20A	6	1	-4.503526565	-0.051127219
859	ABCA9	6	1	-4.723786708	0.102571881
860	ABCA6	6	1	-4.208712932	0.183262206
861	ABCA10	6	1	-4.768882152	0.224620796
869	ITGB4	6	1	-5.62203538	0.197205282
877	LGALS3BP	6	1	-3.894507524	0.16870091
895	TCF4	6	1	-4.738754149	-0.17888216
897	CDH19	6	1	-5.598323222	0.08374179
899	RASSF2	6	1	-5.20386922	-0.181319364
901	JAG1	6	1	-4.08868086	0.20779202
909	FOXS1	6	1	-5.464980956	-0.048143351
924	BMP7	6	1	-5.461821433	-0.183584609
963	LTBP4	6	1	-4.153352853	-0.404121862
996	TIAM1	6	1	-5.423411723	0.085292674
1003	MT-RNR1	6	1	-12.97205484	0.224233962
1004	MT-RNR2	6	1	-12.97387063	0.223049081
1005	MT-ND1	6	1	-12.97547662	0.221887684
1006	MT-ND2	6	1	-12.97616803	0.221580005
1007	MT-CO1	6	1	-12.97580659	0.221580303
1008	MT-CO2	6	1	-12.97507703	0.221941627
1009	MT-ATP6	6	1	-12.97424352	0.222467161
1010	MT-CO3	6	1	-12.97430932	0.222115016
1011	MT-ND3	6	1	-12.9744476	0.222257591
1012	MT-ND4	6	1	-12.9669069	0.226041711
1013	MT-ND5	6	1	-12.98085248	0.218661106
1014	MT-CYB	6	1	-12.9439367	0.241020775
1022	KCTD12	6	1	-4.195186969	0.11193747
1023	BNC2	6	1	-4.974980231	-0.034836405
1032	CALD1	6	1	-3.902768251	0.274980045
1079	FOXD2	6	1	-5.374084827	-0.188998052
1080	EGR3	6	1	-5.528235313	-0.212882706
1118	AFAP1L2	6	1	-5.567348834	0.089801556
1134	RNF157	6	1	-5.593385097	-0.004458957
1144	PCDH8	6	1	-5.206146117	0.123022473
1151	9-Sep	6	1	-5.354022857	0.044739223
1168	FAM102B	6	1	-4.182464477	0.210640228
1204	CAPN5	6	1	-5.528702613	-0.029349619
1209	LINC01197	6	1	-4.388107654	0.329401052
1212	GAS7	6	1	-5.451967593	-0.388128103
1226	ACTN4	6	1	-5.273326751	0.047005362
1232	FREM1	6	1	-5.404729243	-0.151881226
1241	EMP1	6	1	-3.833762761	0.066646731
1242	DKK3	6	1	-4.820304271	0.239335156
1250	SBSPON	6	1	-5.533203002	0.02949459
1254	GFRA1	6	1	-4.375154134	0.335387385
1276	NEGR1	6	1	-4.030271407	0.272669888
1313	SLIT3	6	1	-4.826736327	0.258622027
1384	CHSY1	6	1	-5.328636046	-0.119481915
1402	ZCCHC24	6	1	-3.870423909	0.429515696

1414	FAM198B	6	1	-5.366441127	-0.125387856
1417	KLF5	6	1	-5.075621005	0.201178945
1418	PRKG1	6	1	-4.630598422	0.236377335
1424	AC025280.2	6	1	-5.533186313	0.002746708
1441	SH3PXD2A	6	1	-4.845569011	0.196330166
1455	NES	6	1	-5.362827655	0.069436765
1469	TSPAN5	6	1	-5.500157233	-0.029983067
1471	MTATP6P1	6	1	-12.97604692	0.221479035
1489	PODNL1	6	1	-5.374317046	-0.409647257
1497	PLK2	6	1	-5.626496192	-0.043346548
1501	HIC1	6	1	-4.49650323	0.140780604
1514	PTCH1	6	1	-4.996152755	0.238965667
1518	C16orf45	6	1	-4.953157779	-0.140072503
1532	PLEKHA4	6	1	-4.731878158	0.213192559
1556	RNF24	6	1	-4.592343207	0.066666848
1584	CC2D1A	6	1	-5.442070838	-0.200956949
1592	P2RY1	6	1	-4.516745444	0.251585281
1639	OLFML2A	6	1	-4.950351115	0.01254139
1718	LSP1	6	1	-5.427686091	-0.38951495
1723	PSAT1	6	1	-5.567082759	-0.048107707
1745	CDKN2B	6	1	-5.5264672	-0.087313959
1763	PHLDA3	6	1	-4.211186763	0.348199344
1772	CH25H	6	1	-5.614414569	-0.088258454
1804	F3	6	1	-3.892357703	0.507549262
1828	TSHZ3	6	1	-5.131622668	-0.149092727
1832	C3orf70	6	1	-5.473287936	-0.348903383
1836	PPL	6	1	-4.167207595	-0.418341578
1840	PRKCA	6	1	-5.273047324	-0.148971849
1888	MT-ND4L	6	1	-12.95989167	0.230439043
1902	ARHGAP42	6	1	-4.635630961	0.147989428
1904	ITGA6	6	1	-5.585918304	0.162527806
1941	TSPAN11	6	1	-4.456658956	0.319322503
2062	COL21A1	6	1	-5.088797923	0.057366139
2082	SCN9A	6	1	-5.678707954	0.095995939
2094	SPATS2L	6	1	-4.850440856	0.172217107
2146	RNASET2	6	1	-5.406095382	-0.175064438
2157	KCNK2	6	1	-5.498575088	0.016427166
2170	NLGN4X	6	1	-5.656103488	0.016089923
2176	AGAP2	6	1	-5.545726653	-0.037933969
2184	PHLDB1	6	1	-5.182553168	-0.048131609
2198	ROBO1	6	1	-5.069805499	-0.096347281
2210	PPFIA2	6	1	-3.852689143	0.433603383
2237	ARL4A	6	1	-5.601170894	-0.139436671
2239	NR2F2-AS1	6	1	-3.925915834	0.5416852
2244	LMO4	6	1	-4.321657296	0.315053261
2247	GAS2L3	6	1	-5.652808066	0.035712487
2271	CRYBG3	6	1	-4.264611836	0.1998801
2288	PTPN13	6	1	-4.398968812	0.15375737
2361	STXBP6	6	1	-4.056183215	0.314440823
2369	ETV4	6	1	-5.495350238	-0.116923654
2385	WWTR1	6	1	-4.653881427	0.071862525
2393	IFI27	6	1	-5.663516875	0.245483435
2397	CNTN1	6	1	-4.86316764	0.132094658
2436	PTBP3	6	1	-5.090158816	-0.05866369
2450	PALLD	6	1	-3.797833081	0.234963215
2462	LIPA	6	1	-4.039204236	-0.026937568
2472	EFNB2	6	1	-5.635853644	0.177874959
2474	LDB2	6	1	-3.738291617	0.368279791

2482	SCUBE3	6	1	-5.542318221	0.028752751
2490	PTCH2	6	1	-4.824486133	0.22485075
2493	IFNGR1	6	1	-4.233128902	0.049352235
2507	PPFIBP1	6	1	-5.011313792	-0.079312467
2574	ZEB1	6	1	-4.145772573	0.002005166
2579	PKDCC	6	1	-5.486938354	-0.067778939
2593	TRAC	6	1	-5.188272353	0.129249967
2604	MEST	6	1	-5.619245406	-0.031189137
2626	SOX8	6	1	-5.608435985	-0.044347786
2632	INMT	6	1	-4.702445861	0.228443897
2639	MICALL2	6	1	-5.456727859	0.102443761
2691	RN7SL674P	6	1	-5.607758876	-0.143858948
2744	EPDR1	6	1	-3.706131097	0.466095126
2755	KCNG3	6	1	-5.572280761	0.039932377
2763	UCHL1	6	1	-5.620428439	0.024538315
2771	VGLL4	6	1	-4.165758487	0.020992524
2802	DAAM1	6	1	-4.50298297	0.315462923
2833	PTPN9	6	1	-5.532331344	-0.116285139
2856	ROCK1	6	1	-3.955448505	-0.143575215
2870	PAQR5	6	1	-4.947566386	-0.259844528
2940	FILIP1L	6	1	-3.875111934	0.280469573
2955	BEND4	6	1	-5.609986659	0.072606183
2957	UNC5C	6	1	-4.089261886	0.257204748
2967	LPAR6	6	1	-4.270700093	0.033878512
2971	CYP26B1	6	1	-5.308320876	-0.211825305
11	PDPN	7	1	1.246203665	-3.578343892
38	JUN	7	1	-1.318448242	-4.425825858
46	CYR61	7	1	-0.944530304	-4.012214922
50	COL11A1	7	1	-0.317454498	-3.444537186
53	ITGA10	7	1	-0.362922113	-3.628567242
84	IVNS1ABP	7	1	-0.361429449	-3.511876607
111	RHOB	7	1	-0.565058496	-3.563024306
134	C2orf40	7	1	0.07962114	-3.316191697
173	RAMP1	7	1	0.726458911	-4.030264401
219	CCNL1	7	1	-1.309426721	-4.174414181
233	CYTL1	7	1	0.517932002	-3.939332509
239	SOD3	7	1	0.202303533	-3.727710509
252	IBSP	7	1	0.691694621	-4.00659492
253	MEPE	7	1	0.677023415	-3.948940062
254	SPP1	7	1	0.677054409	-3.967735076
255	BANK1	7	1	0.532670561	-3.91203382
302	LVRN	7	1	0.677278999	-3.990808034
316	EGR1	7	1	-1.306426998	-4.32625916
318	IL17B	7	1	0.658500794	-3.890985989
329	DUSP1	7	1	-1.222024318	-4.198306107
340	F13A1	7	1	0.583662871	-3.892508292
345	ID4	7	1	-1.335720595	-4.343232417
349	HSPA1A	7	1	-1.477647301	-4.643981242
352	COL11A2	7	1	-0.114020403	-3.252945923
357	SRSF3	7	1	-1.259471055	-4.301478648
365	DST	7	1	-1.420468207	-3.297007346
386	CTGF	7	1	-0.774441149	-3.760296607
399	SCIN	7	1	0.015736137	-3.254273438
408	AC005165.1	7	1	-0.270049613	-3.344728493
468	TSC22D3	7	1	-0.358327489	-3.506481671
516	ANXA1	7	1	-1.086192664	-3.961482548
530	KLF4	7	1	-1.136081751	-4.091577792
534	COL27A1	7	1	-0.359330062	-3.475098872

550	CLIC3	7	1	0.48907036	-3.782315516
551	TUBB4B	7	1	-1.048053201	-4.120047593
581	NEAT1	7	1	-1.682739492	-3.592185521
617	DKK1	7	1	0.5959487	-3.900082373
669	AC084816.1	7	1	0.670310978	-3.990806841
674	KIF21A	7	1	-0.851038124	-3.495564961
676	COL2A1	7	1	-0.177895691	-3.424700045
727	RGCC	7	1	-0.217368733	-3.454110646
737	NDRG2	7	1	0.065968696	-3.626062417
754	FOS	7	1	-1.344915863	-4.358319068
777	TPM1	7	1	-0.773417052	-3.256771349
782	CILP	7	1	-0.343423624	-3.300069117
796	ACAN	7	1	-0.265728038	-3.416676545
821	WWP2	7	1	-0.326984774	-3.423639798
822	CLEC18A	7	1	-0.663663696	-3.592167878
846	AC109326.1	7	1	-0.96882784	-3.773766064
852	CHAD	7	1	0.095219795	-3.434901261
853	TOB1	7	1	-0.586811613	-3.636641049
864	SOX9	7	1	-0.393210363	-3.201358819
875	SOCS3	7	1	-1.244612154	-4.464583659
890	SLC14A1	7	1	-0.382446405	-3.364755654
939	CAPS	7	1	-0.346903954	-3.219532752
945	JUNB	7	1	-1.241328057	-4.379529261
948	IER2	7	1	-1.22038376	-4.402816081
952	KLF2	7	1	-1.180552538	-4.352490687
953	CPAMD8	7	1	-0.354699548	-3.466603541
956	CILP2	7	1	-0.366254952	-3.302678132
962	ZFP36	7	1	-1.32013702	-4.482074523
970	FOSB	7	1	-1.280273672	-4.260349536
990	SCUBE1	7	1	0.670915727	-3.98763659
1019	FRZB	7	1	0.595250133	-3.886258149
1035	HSPA8	7	1	-1.558967587	-4.358486914
1064	SSTR5-AS1	7	1	-0.258050572	-3.426605963
1070	MEG3	7	1	-1.652445789	-3.527791762
1086	SAT1	7	1	-1.585085984	-3.73833754
1090	HSPH1	7	1	-1.472777482	-4.577776217
1097	SRSF7	7	1	-1.255613085	-4.111104512
1181	KLF10	7	1	-1.195946511	-4.445653224
1225	BHLHE40	7	1	-0.874047484	-4.378023886
1228	BTG2	7	1	-1.416964289	-4.452690386
1234	AC020916.1	7	1	-1.173798259	-4.261485123
1262	NFKBIZ	7	1	-1.270853635	-4.337417864
1267	SOX5	7	1	-0.818096098	-3.220722937
1269	FGFR3	7	1	-0.100472953	-3.369475865
1280	GADD45B	7	1	-1.284776088	-4.502363944
1286	DDX17	7	1	-1.689554211	-3.529386544
1297	RND3	7	1	-1.588040825	-4.235812449
1306	CREB5	7	1	-0.804332729	-3.253919386
1340	ID1	7	1	-0.019855436	-3.741528296
1353	DDX5	7	1	-1.39991891	-4.102041745
1390	SRSF5	7	1	-1.497067686	-3.756818318
1398	HSP90AA1	7	1	-1.39038026	-4.507000708
1399	DNAJA1	7	1	-1.481572743	-4.549260401
1410	N4BP2L2	7	1	-1.753533479	-3.504900956
1435	C12orf75	7	1	0.784997586	-3.784740948
1458	RASD1	7	1	-1.24381822	-4.482684397
1485	SFPQ	7	1	-1.410713907	-4.043099188
1503	VAV3	7	1	0.656388048	-3.933317208

1517	FKBP5	7	1	-0.326212387	-3.431037926
1542	HES1	7	1	-1.49185228	-4.659003996
1558	EIF5	7	1	-1.32053661	-4.21502974
1609	RNU2-63P	7	1	-0.960325059	-3.585481667
1616	SERTAD1	7	1	-1.413156982	-4.581875586
1623	COLGALT2	7	1	-0.187913772	-3.287369036
1634	BMP3	7	1	0.680566553	-3.977846884
1643	FRMD4B	7	1	-0.751365628	-3.407306218
1695	MIDN	7	1	-1.23345935	-4.311903739
1696	BCL6	7	1	-1.273158487	-3.495349907
1706	DDX24	7	1	-1.468784686	-4.153196835
1707	TIPARP	7	1	-1.10641342	-4.111994767
1716	JUND	7	1	-1.273722049	-4.357883238
1721	SBDS	7	1	-1.43847185	-4.339786315
1725	LPP	7	1	-0.426860161	-3.252885603
1740	PNISR	7	1	-1.678263184	-3.567090058
1750	PPP1R15A	7	1	-1.441433426	-4.615544581
1754	MCL1	7	1	-1.271900829	-4.281209492
1756	AUTS2	7	1	-0.701999139	-3.362905526
1785	IER5	7	1	-1.262090322	-4.500481391
1808	HNRNPA2B	7	1	-1.419359799	-4.018339657
1830	TRA2B	7	1	-1.311437543	-4.198674702
1852	RNU4-2	7	1	-1.03087258	-3.760389113
1863	ATF3	7	1	-1.379472252	-4.569138789
1890	FGFR1	7	1	0.514243428	-3.520087504
1915	SON	7	1	-1.612529751	-3.632451796
1924	NR4A1	7	1	-1.426962193	-4.580035948
1965	ZBTB20	7	1	-1.670505282	-3.464223885
1984	ODC1	7	1	-1.088996347	-4.228077197
1991	PER1	7	1	-1.167570706	-4.157412076
1997	ZRANB2	7	1	-1.677922007	-3.519341254
2001	FRY	7	1	-0.27129766	-3.390841507
2004	CYSLTR1	7	1	0.64824355	-3.94846847
2008	AMD1	7	1	-1.468968507	-3.833141827
2029	CCDC88A	7	1	-0.514607776	-3.599970364
2031	OTUD1	7	1	-0.674402695	-3.527768397
2037	UACA	7	1	0.328105751	-3.824330592
2039	DDX3Y	7	1	-1.344780024	-4.07856419
2040	PRKCZ	7	1	-0.209560256	-3.407047295
2043	AC044849.1	7	1	-1.06518453	-3.957176709
2061	HSPB1	7	1	-1.453878459	-4.601043963
2069	GOLGB1	7	1	-1.443783697	-3.901111149
2070	PLAGL1	7	1	-1.978167411	-3.19232037
2121	LAMC3	7	1	0.649321441	-3.866971993
2191	WSB1	7	1	-1.686548587	-3.627903962
2205	SRRM2	7	1	-1.731637474	-3.493146681
2213	SKIL	7	1	-1.143443998	-4.105724358
2230	TMEM107	7	1	-0.912082758	-3.546451115
2260	HSPA1B	7	1	-1.412034746	-4.614222312
2265	SLC44A2	7	1	0.57267702	-3.755270743
2278	CD14	7	1	0.185638431	-3.713500523
2281	SNORD3A	7	1	-1.05967116	-3.800596499
2292	KLF6	7	1	-1.323439177	-4.442609572
2294	MPHOSPH8	7	1	-1.794529077	-3.378705763
2306	GABBR2	7	1	0.71106172	-3.935397648
2307	MIR99AHG	7	1	-1.830140587	-3.278786921
2320	EMP2	7	1	0.58786476	-3.960097575
2326	PPP1R10	7	1	-1.179007765	-4.124192261

2345	IFITM10	7	1	-0.702562463	-3.597914958
2355	MBNL1	7	1	-1.292073127	-3.215064311
2380	PER3	7	1	-0.454202018	-3.461601519
2383	PNN	7	1	-1.115435954	-3.617048764
2384	MYADM	7	1	-1.231455024	-4.43074634
2439	PDE4DIP	7	1	-1.14180636	-3.354746365
2440	PTP4A1	7	1	-1.150326367	-4.317602419
2494	SERPINI1	7	1	0.572984342	-3.772183442
2499	PAPOLA	7	1	-1.082828876	-3.504139685
2524	RBM39	7	1	-1.518245932	-3.916483187
2551	EPS8L2	7	1	-0.554607343	-3.605896735
2561	MEF2C	7	1	0.65598977	-3.890841031
2563	YBX3	7	1	-1.289368626	-4.184773945
2568	MAFF	7	1	-1.360910352	-4.521011614
2576	ZBTB16	7	1	-0.473844153	-3.426170373
2577	MAP1LC3B	7	1	-1.358339902	-4.542571806
2594	RAB11FIP4	7	1	-0.197583105	-3.43954494
2603	SF1	7	1	-1.539668318	-3.809522414
2642	STC2	7	1	0.284679893	-3.778177762
2643	HSPB8	7	1	-0.83223414	-3.574033999
2722	SRSF2	7	1	-1.318441924	-4.165231251
2731	B3GNT5	7	1	1.126621131	-3.647612834
2736	STAT3	7	1	-1.3161599	-4.447390818
2749	LINC01578	7	1	-1.627147432	-3.68012073
2753	CDKN1A	7	1	-1.306612011	-4.541116499
2785	FUS	7	1	-1.484680053	-3.965160632
2815	CHD9	7	1	-1.474091526	-3.408863568
2828	SOX6	7	1	-0.867079284	-3.324508929
2838	PREX1	7	1	-0.314416867	-3.431147599
2841	FTX	7	1	-1.910418507	-3.341809535
2862	DNAJB1	7	1	-1.363578137	-4.552726054
2877	GOLGA8A	7	1	-1.500366446	-3.557864451
2883	LINC00632	7	1	-0.983988699	-3.295592808
2899	TRH	7	1	0.65683854	-3.936652445
2907	LUC7L3	7	1	-1.839576002	-3.456185603
2921	ADRB2	7	1	0.159021739	-3.422419571
2960	ARGLU1	7	1	-1.762191173	-3.50387671
74	TMCO1	8	1	1.78059781	0.149824626
109	PDIA6	8	1	1.484764937	-1.176696682
175	HDLBP	8	1	1.423087481	-1.050234818
191	ARF4	8	1	2.337213162	-0.743171805
258	OSTC	8	1	1.990946416	0.117070264
293	TMEM167A	8	1	1.71511877	-0.636628919
333	TMED9	8	1	1.685550336	-0.993093395
397	KDELR2	8	1	1.706771497	-1.059074902
418	SEC61G	8	1	2.108250026	0.20139221
434	CALU	8	1	1.813322429	-0.845395112
435	STMP1	8	1	1.640350226	-0.590175682
441	PDIA4	8	1	1.625719193	-1.067132377
491	RAB2A	8	1	1.581847791	-0.155274385
539	HSPA5	8	1	1.671251182	-0.971390867
562	NUCB2	8	1	1.304455522	-0.978953683
566	RCN1	8	1	1.575990085	-1.111425185
591	SPCS2	8	1	1.834493998	0.118365622
613	ITGB1	8	1	1.209485892	-1.040298843
678	TMBIM6	8	1	1.317075256	-0.130035021
708	HSP90B1	8	1	1.459666614	-0.949049854
715	MLEC	8	1	1.597765092	-0.816178315

717	TMED2	8	1	1.791615371	-0.109221854
753	TMED10	8	1	1.489458088	-1.136994981
769	PDIA3	8	1	1.542462233	-1.123060309
780	PPIB	8	1	2.057917956	0.243697978
801	SELENOS	8	1	1.826152209	-0.042557144
947	CALR	8	1	1.555688743	-1.147759223
979	XBP1	8	1	1.762380485	-0.53954547
1018	EDNRB	8	1	1.910835389	-1.203794205
1039	LMAN1	8	1	1.694290046	-0.492937782
1052	RBM3	8	1	1.643344048	-0.142024526
1083	DDX21	8	1	2.702151898	-0.681337767
1136	MORF4L2	8	1	1.363411788	-0.661090874
1176	CANX	8	1	1.493640903	-0.759596461
1182	ARF1	8	1	1.589190129	-0.095504769
1193	COX17	8	1	2.173245553	-0.219988302
1214	TMEM45A	8	1	1.820321683	-0.200729453
1274	TAGLN2	8	1	1.151915673	-0.083204263
1349	FAM114A1	8	1	1.330060843	-1.030080223
1350	ARL1	8	1	1.670778874	-0.738199943
1363	TRAM1	8	1	1.409106616	-0.877615416
1373	KDELR3	8	1	1.80251253	-1.198729598
1386	ERLEC1	8	1	1.490834001	-0.31206396
1400	CDV3	8	1	2.495042924	-0.985973441
1436	HNRNPM	8	1	1.84273899	-0.346770731
1440	IER3IP1	8	1	1.769003991	-0.861566984
1448	SEC61A1	8	1	2.319298629	-1.082620942
1451	GPX8	8	1	1.762878541	-0.788196766
1491	HNRNPC	8	1	1.385044698	-0.328707641
1521	CCDC47	8	1	1.440192584	-0.443035142
1525	HNRNPK	8	1	1.609410886	-0.416815215
1576	DNAJC3	8	1	1.603154067	-0.625181639
1585	FKBP11	8	1	2.180935983	-0.781206095
1597	SPCS3	8	1	2.29814351	-0.643801444
1652	CD164	8	1	1.526402835	-0.208064788
1669	CRELD2	8	1	1.739397649	-0.854392314
1671	TMED3	8	1	1.850259665	-1.267311298
1673	TMEM30A	8	1	1.33535767	-0.808934205
1687	TMEM263	8	1	1.639312152	-0.988892221
1705	SRPRB	8	1	2.181392077	-0.900413417
1710	RAB10	8	1	2.71216524	-0.560033643
1726	PYCR1	8	1	2.191594247	-0.897234046
1732	RCC2	8	1	2.447049502	-0.720940077
1735	CLINT1	8	1	1.668656234	-0.274923389
1736	ADSS	8	1	1.948905591	-0.319059235
1741	CETN2	8	1	2.082952146	-0.707651132
1749	HSPA9	8	1	2.49569023	-1.031645858
1775	FKBP14	8	1	2.356366519	-0.70758512
1782	UXS1	8	1	1.187682156	-1.160615408
1792	PLPP5	8	1	2.085254315	-0.449189686
1800	ACTR3	8	1	1.841152791	-0.843877935
1810	BUD23	8	1	1.226126198	-0.86550256
1812	RAP1B	8	1	2.27593768	-0.026670688
1835	EIF5B	8	1	1.708742503	0.116540409
1870	KIF5B	8	1	2.600071553	-0.573137128
1871	MCFD2	8	1	1.824131612	-0.15955148
1879	PTP4A2	8	1	2.405085925	-0.527342313
1892	DNAJB11	8	1	2.64982212	-0.742669189
1897	SEC31A	8	1	1.549967889	-0.545026192

1908	SYNCRIP	8	1	2.307025317	-0.493078717
1910	TCEAL3	8	1	1.564243201	0.005362726
1949	XRCC5	8	1	1.85079873	0.026873416
1955	UBE2D3	8	1	1.152358178	-0.14776186
1956	YWHAG	8	1	2.358965758	-0.198134937
1964	SSR1	8	1	1.427975778	-1.007476115
1966	SYAP1	8	1	2.04894245	-0.321974802
1973	TM9SF3	8	1	1.500455264	-0.658318185
1974	ACBD3	8	1	1.648512486	-0.200816878
1995	SAR1B	8	1	1.90769494	-0.526680746
2051	PNO1	8	1	2.23256195	-0.347851299
2056	TARS	8	1	2.325681809	-0.502267414
2080	GFPT1	8	1	1.42701352	-0.727211111
2090	SURF4	8	1	1.732266072	-1.189217293
2091	SAR1A	8	1	1.860715274	-0.666669958
2109	STAU1	8	1	2.192515735	-0.309937586
2112	PRKAR1A	8	1	1.841671828	-0.548899108
2132	HACD3	8	1	1.896933679	-0.548237958
2136	DNAJB9	8	1	1.660085801	-0.438191504
2139	MESD	8	1	1.838783626	0.21686647
2140	ARFGAP3	8	1	1.837654714	-0.440135003
2141	HNRNPR	8	1	1.553292874	-0.25488078
2142	EIF3A	8	1	1.942057494	-0.470230469
2145	RPN1	8	1	1.636992101	-1.122925543
2152	YIPF5	8	1	1.651816014	-0.091694095
2166	DNAJC1	8	1	1.724831704	0.173908031
2187	GNB1	8	1	1.394748811	-0.581544601
2211	HM13	8	1	2.201306943	-0.906041765
2215	RAB1A	8	1	1.711920623	-0.271334351
2227	DNMT1	8	1	1.537568573	-0.258842454
2235	IMPAD1	8	1	1.713576678	-0.899660372
2238	GOLT1B	8	1	2.589684609	-0.741215223
2253	ACTR2	8	1	1.320176367	-0.448757955
2268	TMED5	8	1	2.54707611	-1.114697241
2272	ARCN1	8	1	1.909554604	-0.865886831
2285	SELENOF	8	1	1.646655683	-0.262629503
2295	UFM1	8	1	1.590045337	-0.535777115
2305	SRPRA	8	1	1.835263375	-0.485260808
2330	SEC13	8	1	2.29855502	-0.735937947
2348	SH3GLB1	8	1	1.656864766	-0.680809998
2376	EIF4G2	8	1	2.380537871	-0.596841955
2400	COPB1	8	1	1.693890218	-0.774308973
2403	EIF4G1	8	1	2.473932866	-1.184201502
2404	ATP2B1	8	1	1.452245478	-0.87269541
2406	CLTC	8	1	1.415083889	-0.767777645
2443	C5orf15	8	1	1.605794076	-1.141357505
2451	HERPUD1	8	1	1.261012439	-0.273799577
2455	ARHGDIA	8	1	2.29074562	-1.259385788
2464	SPPL2A	8	1	1.259028319	-0.953522586
2475	TXNDC15	8	1	1.633417014	-0.740861469
2528	SEC22B	8	1	1.517084364	-0.957929039
2538	XRCC6	8	1	1.721477393	-0.977341973
2542	ILF2	8	1	2.688844327	-0.721810573
2572	ATP13A3	8	1	2.48584092	-1.009864533
2595	TMX1	8	1	1.335212711	-0.895696961
2597	MZT1	8	1	1.892763738	-0.080705607
2598	SELENOT	8	1	2.405300979	-0.31093626
2601	SLC39A7	8	1	1.903564338	-0.922924125

2605	TM9SF2	8	1	1.398435835	-0.825201565
2617	HDAC2	8	1	1.897092704	-0.812106901
2619	GNL3	8	1	2.045942668	-0.47741937
2621	DDX1	8	1	1.816873435	-0.910352552
2624	MAGEH1	8	1	1.467888001	-1.042095685
2627	ERCC1	8	1	1.514126185	-1.00446918
2637	HYOU1	8	1	1.893170957	-0.959211075
2638	TOP1	8	1	2.587731961	-0.867969119
2641	FAM98A	8	1	1.910999183	-0.979320192
2645	IPO7	8	1	2.479306582	-0.678523087
2646	EIF2AK1	8	1	1.344344858	-0.688192182
2653	NAA50	8	1	2.241400603	-0.712514841
2659	PRKDC	8	1	2.003402118	-0.355650374
2666	PAICS	8	1	2.786405925	-0.774151617
2671	2-Sep	8	1	1.956653956	-0.504735523
2683	DDX18	8	1	1.618990783	-0.020169729
2693	HSPA4	8	1	2.568430308	-0.525359565
2696	ABCF1	8	1	2.72936428	-0.692452156
2728	BACH1	8	1	2.091053609	-1.118783855
2738	TAF13	8	1	2.529187802	-0.861271226
2740	PGP	8	1	2.947817449	-0.913161182
2750	PSMD12	8	1	1.873951558	0.188171184
2770	PGM3	8	1	2.261094455	-0.877281987
2777	MAPRE1	8	1	2.46991289	-0.413446137
2780	DPH3	8	1	2.876575831	-0.976611101
2791	JKAMP	8	1	1.595796231	-0.288075701
2793	MAPK1IP1L	8	1	1.42456663	-0.050998383
2795	MOB1A	8	1	2.343710307	-0.866815888
2800	ETF1	8	1	2.627784137	-0.862347805
2832	USO1	8	1	1.390608076	-0.941689455
2842	TMED7	8	1	1.441456202	-0.872886025
2850	MAP7D1	8	1	2.593290452	-0.53122408
2861	HNRNPA3	8	1	1.542208795	-0.255494156
2867	ACSL3	8	1	1.458571319	-0.630628669
2882	ATP6V1D	8	1	2.469571237	-0.821379268
2888	RAD21	8	1	1.338164095	-0.06552639
2889	EPRS	8	1	1.8550607	-0.260683493
2898	COPB2	8	1	1.98260105	-0.846234524
2914	VCP	8	1	2.458638076	-1.012467587
2918	ILF3	8	1	2.154019479	-0.704406255
2922	STT3A	8	1	1.483845715	-1.138634169
2930	ICMT	8	1	2.911936644	-0.906134927
2963	GORASP2	8	1	1.886708859	-0.92245903
2972	SMIM15	8	1	1.968876246	-0.410490037
2991	ERGIC2	8	1	1.857464675	0.087621636
9	FBXO2	9	1	0.236824874	-2.441782021
28	COL9A2	9	1	0.066491071	-3.003760361
63	S100A1	9	1	0.270427052	-3.097532296
129	CAPG	9	1	0.236341123	-2.587905192
153	COL5A2	9	1	0.692623261	-2.768201851
167	ITM2C	9	1	0.027887825	-2.566334271
169	SNORC	9	1	0.032061223	-2.983275675
170	NGEF	9	1	0.144466404	-3.178667092
176	TIMP4	9	1	0.248591844	-2.484693551
181	CRTAP	9	1	0.32221115	-2.049654984
213	PLOD2	9	1	0.881729368	-2.850773358
216	CP	9	1	-0.249071907	-2.459170126
228	MELTF	9	1	0.849083904	-2.840120577

229	FGFRL1	9	1	0.812169794	-2.79937675
235	HS3ST1	9	1	-0.149096843	-2.656967663
237	FGFBP2	9	1	0.350405399	-3.207302832
271	MFAP3L	9	1	-0.051064994	-2.738511824
273	SCRG1	9	1	0.090458099	-2.982424283
295	HAPLN1	9	1	0.639063481	-3.154171967
296	EDIL3	9	1	0.997266535	-2.404141449
320	SLC26A2	9	1	-0.236884069	-2.789104008
323	SPARC	9	1	0.442630414	-3.150377297
336	FOXF2	9	1	-0.267941471	-2.676358246
338	TUBB2A	9	1	0.817035679	-2.343691372
339	TUBB2B	9	1	0.142229859	-2.654134535
341	DSP	9	1	0.578588132	-2.838138365
360	MDFI	9	1	0.131890778	-2.979639077
375	WISP3	9	1	0.329243187	-2.556102538
388	CITED2	9	1	-0.400870074	-2.673445725
406	MPP6	9	1	0.293151263	-2.991543555
438	CREB3L2	9	1	-0.086501595	-2.693267369
454	ITIH6	9	1	0.288774017	-3.10599091
455	MAGED2	9	1	0.2719847	-2.289794707
460	ITM2A	9	1	0.134803776	-2.292916917
461	TSPAN6	9	1	0.275466446	-2.470474267
463	TCEAL2	9	1	0.26714069	-2.095281028
464	TCEAL6	9	1	0.28445131	-2.228538775
465	BEX4	9	1	0.242034677	-2.132717633
466	BEX3	9	1	0.255963627	-2.010434532
473	AL078639.1	9	1	0.517230812	-2.485243582
478	LPL	9	1	-0.164046552	-2.604894661
490	SNAI2	9	1	0.09115136	-2.437323832
495	CRISPLD1	9	1	0.019065741	-2.449229264
496	TPD52	9	1	0.857503179	-2.467279458
499	SDC2	9	1	0.102353994	-2.231997394
505	EIF3H	9	1	0.210816268	-2.036848807
513	ENHO	9	1	0.210416678	-2.736415171
523	OMD	9	1	0.036399189	-2.370614075
526	BARX1	9	1	0.057219867	-2.709034705
538	GGTA1P	9	1	0.33597279	-2.452627205
590	CHRDL2	9	1	0.277003709	-2.684761309
602	FXYD6	9	1	0.319901768	-2.607288861
604	THY1	9	1	-0.255440276	-2.709206128
612	BAMBI	9	1	-0.090857472	-2.743137621
623	CHST3	9	1	0.443020228	-2.975537562
625	P4HA1	9	1	0.747427706	-2.034576439
631	PAPSS2	9	1	0.46290249	-2.821257853
632	PPP1R3C	9	1	-0.006479677	-2.713235163
671	BHLHE41	9	1	-0.001012798	-2.393317008
692	NDUFA4L2	9	1	0.767126206	-2.617172503
730	CNMD	9	1	0.016596619	-2.753605866
733	GPC6	9	1	0.519547287	-2.652940774
758	SERPINA1	9	1	0.608140711	-3.158849263
759	SERPINA5	9	1	-0.024515565	-2.630346798
786	PKM	9	1	0.883159284	-2.508132958
788	RCN2	9	1	0.672496442	-1.586717271
806	XYLT1	9	1	-0.22109298	-2.828836703
824	CLEC3A	9	1	0.393190805	-3.19974997
862	ROCR	9	1	-0.092871543	-2.558885121
863	SOX9-AS1	9	1	0.086125854	-2.737409615
865	GPRC5C	9	1	0.118717495	-3.049276852

889	GALNT1	9	1	0.253239993	-2.420531535
906	AL035258.1	9	1	0.244528476	-2.196405196
925	PMEPA1	9	1	1.009116773	-2.882436776
929	COL9A3	9	1	0.030637357	-3.207294249
958	DMKN	9	1	0.439845923	-1.738574171
960	SPINT2	9	1	0.553690199	-3.452774787
964	MIA	9	1	0.289948884	-3.100049758
986	CHADL	9	1	-0.001158591	-2.888071799
1002	S100B	9	1	0.054125849	-2.86205151
1028	TSPAN13	9	1	0.257622842	-2.715367817
1108	LSAMP	9	1	-0.255371388	-2.684710288
1112	METRN	9	1	0.107964341	-2.537227416
1116	GALNT18	9	1	-0.109357592	-2.721733117
1130	TGOLN2	9	1	0.40369809	-2.071427846
1135	SLC29A1	9	1	0.617776874	-3.272228503
1138	COL6A1	9	1	0.949216608	-2.390743756
1152	SRPX2	9	1	0.582215909	-2.874781632
1164	SLC39A6	9	1	0.740312818	-2.055472159
1217	B3GNT7	9	1	0.240725998	-3.013830447
1229	CD59	9	1	0.716524605	-1.684159779
1235	CHID1	9	1	0.138331894	-1.770916247
1264	CHST6	9	1	0.48486853	-3.159607434
1307	NEBL	9	1	0.764275316	-3.112839961
1319	GALE	9	1	0.980129842	-2.947931313
1347	BEX2	9	1	0.442005459	-2.171677851
1365	SUSD5	9	1	0.609278206	-2.689231658
1369	SPON2	9	1	-0.207593139	-2.672420048
1379	TRPV4	9	1	0.665437225	-3.05719068
1403	SMIM5	9	1	-0.087171342	-2.615278506
1411	PAPSS1	9	1	0.31321061	-2.380353236
1415	RHOBTB3	9	1	-0.246422823	-2.645587945
1419	PABPC4	9	1	0.167933587	-2.935601496
1450	LAGE3	9	1	0.463210408	-1.779942894
1474	NREP	9	1	0.673118953	-3.033117079
1492	SDK2	9	1	0.408560697	-3.016635918
1506	GPC1	9	1	0.061916832	-2.988368535
1523	HOXB8	9	1	0.180043582	-2.461873793
1528	ATP1A1	9	1	0.604233149	-3.099857831
1534	HRCT1	9	1	0.066308025	-2.307253861
1540	CSGALNAC	9	1	-0.198478874	-2.643451714
1544	CREG1	9	1	0.360524241	-1.892892623
1568	C11orf1	9	1	0.607586745	-2.002617979
1575	ZCCHC17	9	1	0.186680201	-2.213437342
1587	HILS1	9	1	-0.258540343	-2.685288453
1628	GCHFR	9	1	0.020860199	-2.18616774
1646	PCSK1N	9	1	0.17377824	-2.745324873
1679	SLC1A5	9	1	0.766779427	-2.742284798
1683	IQSEC1	9	1	0.017880026	-3.143462443
1684	NINJ1	9	1	0.009431664	-2.098238372
1708	MEF2A	9	1	-0.376320105	-2.431957984
1777	FYB1	9	1	-0.039260443	-3.144841694
1784	RAPH1	9	1	0.745998744	-2.900511288
1794	CHSY3	9	1	-0.082613524	-2.673615002
1820	TCEAL5	9	1	0.407482866	-2.041499519
1837	CD8B2	9	1	0.22921956	-2.148974442
1861	AC068888.1	9	1	0.204616074	-2.220052981
1907	PCSK2	9	1	0.023212854	-2.536894583
1922	CD8B	9	1	-0.017911311	-2.473943257

1938	STK38L	9	1	1.117121581	-3.304309153
1978	ANKRD36BI	9	1	0.186438862	-1.903926396
1979	HHIP-AS1	9	1	-0.210776221	-2.471612715
2020	AC245297.1	9	1	0.2909385	-2.147286677
2021	FGF2	9	1	1.121337298	-3.334519887
2022	GCNT1	9	1	0.486967448	-2.884849333
2035	RNF130	9	1	-0.266335886	-2.562745595
2057	FGGY	9	1	-0.133515295	-2.51633265
2098	GREM1	9	1	0.20814288	-2.358378911
2119	CA5B	9	1	0.460332278	-2.850403094
2186	SPTSSB	9	1	-0.069049951	-2.502988839
2195	TWSG1	9	1	0.63988686	-2.41306355
2221	PPP1R16A	9	1	-0.293612327	-2.792225384
2223	PPP1R1B	9	1	-0.149444457	-2.542155051
2240	H19	9	1	-0.229751702	-2.686768317
2246	USH1C	9	1	-0.18948644	-2.611381316
2257	TNS3	9	1	0.96156526	-3.107517027
2282	INSIG1	9	1	0.871139292	-3.066076541
2283	PEG10	9	1	0.208616737	-2.35106399
2360	ADSSL1	9	1	0.39450616	-2.259614372
2364	LITAF	9	1	0.302424673	-2.093262338
2375	STK26	9	1	0.549710993	-3.471341157
2379	ZNF385D	9	1	0.284434561	-2.468302989
2396	RCAN3	9	1	0.21071947	-3.049258256
2412	SERTAD4	9	1	-0.009912129	-2.45719769
2432	MSI2	9	1	0.30600727	-3.024059796
2506	PHYH	9	1	-0.221973952	-2.345804715
2526	FUCA1	9	1	0.212565247	-1.685548687
2544	MIR497HG	9	1	0.329515461	-2.18136909
2547	ATF4	9	1	1.047399882	-2.717955851
2548	UBL3	9	1	0.767093781	-1.82796123
2584	KMT5A	9	1	0.210576836	-3.020438218
2652	DIO2	9	1	-0.399165373	-2.566380524
2680	ERGIC3	9	1	0.691186789	-2.042265319
2690	CHST1	9	1	0.606645349	-3.126672291
2725	FILIP1	9	1	-0.244350966	-2.632375979
2743	SHC4	9	1	0.423464481	-2.918666863
2746	PART1	9	1	0.320936147	-2.177058482
2760	SDC3	9	1	0.028628234	-2.699388527
2764	IGF2.1	9	1	0.31501675	-2.289592886
2769	UMAD1	9	1	-0.250120755	-2.667064213
2772	CYB5D2	9	1	0.085673574	-2.363339924
2804	PON3	9	1	0.168503586	-2.311395788
2826	DOK1	9	1	0.681785349	-2.455643439
2928	WSCD2	9	1	0.13613886	-3.069102311
2933	HOXB6	9	1	0.091249589	-1.750850343
2938	OBSL1	9	1	-0.085581984	-2.68616321
2942	MATN4	9	1	0.108597998	-2.54703238
2959	CTBS	9	1	-0.006283995	-1.83176949
2968	POFUT2	9	1	0.566368584	-2.357079052
2977	SYTL4	9	1	0.176027719	-3.050470614
2996	EFS	9	1	0.069995645	-2.736084008
8	ANGPTL7	10	1	-3.623620387	-2.40910151
12	NBL1	10	1	-3.081705924	-2.167768979
36	PLPP3	10	1	-2.768597718	-1.849018836
56	CTSK	10	1	-2.739278194	-1.928592944
79	MYOC	10	1	-3.527719375	-2.25153234
91	CHI3L1	10	1	-3.58285391	-2.378055596

123	RTN4	10	1	-2.703311082	-1.660884881
143	CACNB4	10	1	-3.512169953	-2.405620837
154	SLC40A1	10	1	-3.428523894	-2.50058701
161	FN1	10	1	-2.770933744	-2.389907622
162	IGFBP5	10	1	-3.188408013	-2.232583665
171	ACKR3	10	1	-2.504996177	-1.860612535
198	COL8A1	10	1	-3.5968219	-2.119539642
205	MYLK	10	1	-3.578509208	-2.691915297
217	TM4SF1	10	1	-3.625932571	-2.620976948
221	RARRES1	10	1	-3.597522613	-2.519694352
247	11-Sep	10	1	-3.529133912	-2.137882137
286	PIK3R1	10	1	-3.068846103	-2.034670972
292	THBS4	10	1	-3.341868516	-2.272779965
366	COL12A1	10	1	-3.098366614	-1.964849376
394	THBS2	10	1	-2.853596088	-2.161553883
413	AEBP1	10	1	-2.7895776	-2.094885253
422	SEMA3C	10	1	-2.616858121	-1.943879747
424	STEAP2	10	1	-3.407991286	-2.372504735
445	VIPR2	10	1	-3.608869668	-2.591157221
447	MXRA5	10	1	-3.374866363	-2.232860589
456	AR	10	1	-3.152039167	-2.030238294
469	PLS3	10	1	-2.521132823	-1.7874408
500	MATN2	10	1	-2.915238257	-2.30134716
507	NOV	10	1	-3.603734847	-2.510623955
515	MAMDC2	10	1	-2.915233251	-1.83235898
517	NTRK2	10	1	-3.017440911	-2.022926235
518	GAS1	10	1	-2.634916183	-1.938046479
524	ASPN	10	1	-2.725712653	-2.015975737
536	TNC	10	1	-3.703424092	-2.451160693
549	PTGDS	10	1	-3.661336537	-2.535833382
557	CDKN1C	10	1	-3.427770253	-2.325461173
560	PDE3B	10	1	-3.548549052	-2.631205105
563	SAA1	10	1	-3.54516327	-2.639158034
595	NOX4	10	1	-2.969280597	-2.118783497
607	APLP2	10	1	-2.683575507	-2.000794315
615	FZD8	10	1	-2.651193257	-1.988465213
627	LRMDA	10	1	-3.186856862	-2.588646197
630	ADIRF	10	1	-3.048730966	-2.275391602
643	CPXM2	10	1	-3.485298511	-2.383571648
664	MGST1	10	1	-2.689072963	-1.818563365
665	LMO3	10	1	-3.455450889	-2.54582026
673	FGD4	10	1	-3.183425542	-2.219568157
679	LIMA1	10	1	-3.128516313	-2.071013593
682	IGFBP6	10	1	-3.240548011	-2.210835003
701	KERA	10	1	-3.594890948	-2.692670607
728	TSC22D1	10	1	-3.458573457	-2.742313408
734	ITGBL1	10	1	-2.694240686	-2.005632305
764	THBS1	10	1	-3.394553777	-2.609874987
797	MFGE8	10	1	-2.843081828	-2.612998271
799	SLCO3A1	10	1	-3.458809014	-2.142591023
825	MAF	10	1	-3.347602721	-2.402290606
885	PIEZO2	10	1	-3.269188996	-2.372382426
915	WISP2	10	1	-2.921891089	-2.008708024
916	KCNK15	10	1	-3.208329555	-2.399567627
1024	NID2	10	1	-3.429549333	-2.467030072
1062	LIMCH1	10	1	-3.111935731	-2.441522622
1071	PROS1	10	1	-3.346037742	-1.784381175
1094	CDH13	10	1	-3.006040689	-2.318333768

1124	ZIC2	10	1	-3.541792508	-2.572380805
1140	SEMA3B	10	1	-3.581420537	-2.554656052
1184	SEMA3D	10	1	-3.520032044	-2.617526793
1199	LGR5	10	1	-3.581830379	-2.644003415
1238	SCARA3	10	1	-2.975239154	-2.51772716
1268	PPP3CA	10	1	-2.594148513	-2.411562705
1271	NTN1	10	1	-2.748772498	-1.892010355
1289	RNF144A	10	1	-3.436117526	-2.277513289
1302	FAT4	10	1	-3.290870782	-1.848517203
1310	CAVIN2	10	1	-3.542654153	-2.548486971
1318	PDZRN4	10	1	-2.909295198	-2.027835989
1351	CMYA5	10	1	-3.439813968	-2.637037062
1377	ISCU	10	1	-3.225830909	-2.08817377
1387	FAXDC2	10	1	-3.020898696	-2.372002863
1431	PLA2R1	10	1	-3.494462844	-2.368744397
1449	ARHGAP29	10	1	-3.556402322	-2.631253266
1459	CASP4	10	1	-2.801607724	-1.804674053
1478	SLC43A3	10	1	-2.894003507	-1.981930518
1527	FAM13C	10	1	-3.384358522	-2.283631348
1582	ST8SIA1	10	1	-3.451394673	-2.387775683
1590	TGFB2	10	1	-3.535652515	-2.573359513
1604	DSE	10	1	-2.683173772	-1.949208402
1606	DIO3OS	10	1	-3.240555879	-1.876454854
1625	TAC1	10	1	-3.581521627	-2.726774239
1640	ELL2	10	1	-3.165907737	-2.566416764
1656	COL23A1	10	1	-3.572597619	-2.585349106
1667	ZNF503	10	1	-3.258206721	-2.001685762
1720	PPM1L	10	1	-2.793961402	-1.932340169
1737	DHRS3	10	1	-2.560933944	-1.944834613
1744	AP002004.1	10	1	-3.463102456	-2.579300427
1752	SSC5D	10	1	-2.622207757	-1.780524754
1760	AIG1	10	1	-2.788830634	-2.091825389
1855	HSPA2	10	1	-2.86948311	-2.515143656
1869	MAN1C1	10	1	-3.308224078	-1.920499706
1926	MYO10	10	1	-3.24997985	-2.682118916
1928	PTPRD	10	1	-3.341297265	-2.410631442
1939	XG	10	1	-2.563079473	-1.868433976
1957	MPDZ	10	1	-3.378621694	-2.325162315
1975	HACD4	10	1	-3.101829406	-2.562514329
2000	ADARB1	10	1	-2.998631354	-2.082465553
2024	VAMP5	10	1	-3.201391812	-2.383227849
2032	F2R	10	1	-3.290388461	-2.331309819
2046	RBP1	10	1	-3.535231944	-2.711073899
2064	OAF	10	1	-3.697492477	-2.43990519
2075	CACHD1	10	1	-3.118056651	-2.258525037
2096	KIF22	10	1	-3.130739804	-2.384241366
2116	RGS6	10	1	-3.432056066	-2.401596331
2183	TMOD1	10	1	-3.141260978	-2.333761239
2219	RARRES3	10	1	-3.435543653	-2.150641703
2222	GPM6B	10	1	-3.485360261	-2.59706428
2303	CEP126	10	1	-2.67253029	-1.750500464
2310	LEFTY2	10	1	-3.574249383	-2.50441482
2340	SLC26A4	10	1	-3.274574157	-2.520177388
2344	ADAMTSL2	10	1	-3.615608569	-2.51295259
2352	LXN	10	1	-3.55753934	-2.394606852
2353	GDF7	10	1	-3.514912721	-2.624713921
2359	NLGN1	10	1	-3.486477729	-1.915617966
2389	AL139082.1	10	1	-3.547986384	-2.609324002

2390	MKX	10	1	-3.23005807	-2.518303418
2395	IL32	10	1	-3.291087743	-2.459761881
2398	PRCP	10	1	-3.238674995	-2.13736751
2399	MPPED2	10	1	-3.466387387	-2.622075104
2407	FAM107B	10	1	-3.122188207	-2.26481142
2433	IDS	10	1	-2.970505353	-2.270328545
2438	WNT5B	10	1	-3.204530355	-2.490906977
2497	ITGA1	10	1	-3.207736369	-2.332396292
2503	SLC44A1	10	1	-3.05649459	-2.308715844
2550	DSEL	10	1	-3.315470573	-2.457611346
2562	TJP1	10	1	-3.699009772	-1.969745421
2591	ECHDC2	10	1	-3.327450629	-2.591081404
2620	BMP4	10	1	-2.716764327	-1.91260662
2717	FIBIN	10	1	-2.736724015	-2.109701895
2745	PAPPA2	10	1	-3.579302903	-2.649545931
2759	AC092376.1	10	1	-3.616331693	-2.557524466
2788	ELMO1	10	1	-3.525539514	-1.782732033
2810	SYNE1	10	1	-2.548921939	-2.02398386
2812	ANXA11	10	1	-2.827708598	-1.685887956
2823	MYO1D	10	1	-3.186705228	-2.49572947
2897	SEZ6L	10	1	-3.521909829	-2.617536568
2958	DIRAS3	10	1	-3.382844087	-2.462131047
2999	DNM3OS	10	1	-3.499073859	-2.660697484
1	HES4	11	1	-2.6067692	1.213998294
15	ALPL	11	1	-3.090721246	2.64090941
23	MARCKSL1	11	1	-3.179768678	2.011201358
41	SGIP1	11	1	-2.632834073	1.054856158
49	VCAM1	11	1	-3.032427903	1.815996147
71	OLFML2B	11	1	-3.334864493	2.05129335
220	PTX3	11	1	-2.765613195	2.350370384
245	CXCL2	11	1	-2.542746421	2.082934356
257	LEF1	11	1	-3.170430537	2.304163433
259	ENPEP	11	1	-3.070766088	1.804498649
274	NKD2	11	1	-3.152955171	2.657106376
279	SLC1A3	11	1	-2.926092502	1.903780914
319	CARMN	11	1	-2.791260358	1.414931632
347	IER3	11	1	-2.276535507	1.81634543
363	RUNX2	11	1	-3.150987979	2.877427078
387	SGK1	11	1	-2.130399819	1.720157361
428	DLX5	11	1	-3.016320344	2.638766742
509	MYC	11	1	-2.709507581	2.110510087
520	S1PR3	11	1	-2.970785495	1.976191259
568	C11orf96	11	1	-2.479701634	1.299364186
570	MDK	11	1	-3.099509355	2.192578531
596	FAT3	11	1	-3.17062461	2.876041866
639	DUSP5	11	1	-2.481219884	1.868849731
725	POSTN	11	1	-2.963030454	2.118989206
735	COL4A1	11	1	-2.945076104	0.848975993
736	COL4A2	11	1	-2.726943608	0.916757679
828	COTL1	11	1	-3.14330947	2.871878362
850	ITGA3	11	1	-2.533319827	1.104334808
870	SPHK1	11	1	-2.222900029	0.988614655
893	RAB27B	11	1	-3.191937324	2.668288923
894	CCDC68	11	1	-3.076541539	2.611864067
896	PMAIP1	11	1	-2.64315426	2.155427909
932	CNN2	11	1	-3.01658785	1.972837425
950	NOTCH3	11	1	-2.836843844	1.045934058
981	LIF	11	1	-2.220311876	1.738014913

1069	TAGLN	11	1	-3.117099162	1.712082839
1123	ARL4C	11	1	-2.717259522	2.032646156
1127	VGLL3	11	1	-3.105362292	1.383322573
1129	FABP5	11	1	-3.054494258	1.956650234
1143	CXCL3	11	1	-2.544787284	2.089863277
1175	MAP4K4	11	1	-2.660500642	0.773278571
1180	FRAS1	11	1	-3.119147416	2.5707402
1206	PI15	11	1	-2.554705974	1.16621635
1208	PAPPA	11	1	-2.888314601	1.683074213
1227	HRH2	11	1	-2.558026906	1.160447693
1244	QPRT	11	1	-2.988699075	2.250075555
1251	TMEM108	11	1	-2.976688024	1.76284287
1258	SRGN	11	1	-2.774898883	2.077275253
1288	MYH9	11	1	-2.780433055	1.014726139
1291	TNFAIP3	11	1	-2.654634114	2.081787086
1304	RRAD	11	1	-2.318439957	1.926535106
1323	EDNRA	11	1	-2.689452048	1.314790106
1388	SPOCD1	11	1	-2.860861417	2.141835189
1394	ABL2	11	1	-1.957413908	1.406535721
1396	DLX6	11	1	-2.782923099	1.438611007
1406	KLK4	11	1	-3.202483531	2.715526319
1422	FOXC2	11	1	-2.921877023	2.45094297
1423	TRIB1	11	1	-2.31763744	1.877931095
1456	PGF	11	1	-3.052878257	2.035456872
1473	TGFB1	11	1	-2.850142118	2.821525073
1482	IRF1	11	1	-2.77639186	2.130045867
1495	HEY2	11	1	-3.134279367	2.336772895
1507	ITGA4	11	1	-3.130234595	2.539557195
1520	CRNDE	11	1	-2.997852679	2.931346631
1526	TBX2	11	1	-2.497404929	1.184407211
1539	HEY1	11	1	-2.880041476	2.148762918
1565	LINC01705	11	1	-2.4110558	1.999062515
1569	GMFG	11	1	-3.102378484	2.076362586
1599	PLAU	11	1	-2.621072169	1.183569289
1611	EML4	11	1	-3.114781734	1.847797609
1630	DDIT4	11	1	-2.704396125	2.256419397
1645	EPAS1	11	1	-2.646203395	1.29384861
1649	LIMD2	11	1	-3.070639964	2.062829471
1678	ADAMTS9	11	1	-2.781718846	1.912320352
1694	PIK3R3	11	1	-2.47056305	1.264354921
1768	VCL	11	1	-2.661729213	1.187862969
1773	DUSP4	11	1	-2.629626866	1.852013088
1786	ENPP2	11	1	-3.122290965	2.670248723
1788	GLIPR1	11	1	-3.148588773	2.383191801
1807	CALN1	11	1	-3.09813654	2.480715728
1821	LAMP5	11	1	-3.146823045	2.632180667
1824	FAM241A	11	1	-2.913671847	2.042204357
1829	PAWR	11	1	-2.825605985	1.102325654
1839	LRRC32	11	1	-2.765854713	1.069902277
1849	CCBE1	11	1	-3.123731729	2.648459888
1857	COL18A1	11	1	-2.445044037	1.035689331
1885	TMEM132A	11	1	-2.494818803	2.158841825
1899	STEAP4	11	1	-2.873963233	1.20116005
1953	CSF1	11	1	-3.192241784	2.13258884
1958	MIR22HG	11	1	-2.242966648	1.795722223
1971	SEMA7A	11	1	-3.262677547	2.013290143
1985	CARD16	11	1	-3.044695254	2.263724065
2014	SLC4A4	11	1	-2.926220056	2.25658176

2055	CTSC	11	1	-3.061730023	1.759916521
2076	RORB	11	1	-3.097901937	2.53310988
2079	AC093908.1	11	1	-2.487522956	1.115823722
2087	AC116366.1	11	1	-2.653341409	2.273291326
2127	ADAMTS1	11	1	-2.536505099	2.055550313
2131	CHCHD10	11	1	-2.996441241	1.89947126
2137	IFI30	11	1	-2.678203221	1.96856854
2161	WDR86	11	1	-2.970529433	1.667477346
2182	PSMB9	11	1	-3.112310287	2.211210466
2236	PLEKHO1	11	1	-2.40139186	1.470408059
2263	IL4R	11	1	-2.304054376	1.038241721
2276	NEFM	11	1	-2.491769191	2.006024337
2293	ERRFI1	11	1	-2.818047877	2.342488265
2296	ISG15	11	1	-3.100017425	2.161126352
2298	MMD	11	1	-2.870641585	2.227287269
2301	PDGFA	11	1	-2.530596849	1.184353328
2321	BID	11	1	-3.190798636	2.856959081
2350	ADGRF5	11	1	-2.695095655	1.272409296
2365	C9orf47	11	1	-2.932321902	2.31175921
2366	IRX5	11	1	-2.449117657	2.251919008
2437	CCL2	11	1	-2.911204215	2.005233264
2469	MSC	11	1	-2.771755572	2.077299095
2485	ICAM4	11	1	-2.632976171	1.897486902
2486	LIFR	11	1	-3.064856645	2.477162338
2509	CXCL8	11	1	-2.559158441	2.060178733
2529	PDK4	11	1	-2.258285876	1.074094034
2532	TMEM37	11	1	-2.930740949	2.276482082
2534	HSPA6	11	1	-2.468688842	2.018049694
2535	PSMB8	11	1	-3.118626948	2.231664157
2536	FBLIM1	11	1	-2.700613376	1.221052385
2596	GJC1	11	1	-2.633978721	1.302653289
2615	NFKBIA	11	1	-2.310807105	1.88224814
2664	ARHGAP28	11	1	-3.129140492	2.287307716
2678	AL034417.3	11	1	-2.55856454	1.954159952
2699	TNFSF11	11	1	-3.099435922	2.459540344
2709	GLIPR2	11	1	-3.14389288	2.239240146
2711	S1PR1	11	1	-3.093459483	2.120620466
2719	SNAI1	11	1	-2.301273819	1.836168266
2721	AC090740.1	11	1	-2.992194768	2.383483863
2734	EBF3	11	1	-3.093189593	1.658793784
2773	EFHD1	11	1	-2.641105052	1.264761186
2814	CXCL1	11	1	-2.873287316	2.228346801
2820	C1orf54	11	1	-3.12195861	1.835231519
2860	RGS5	11	1	-2.693758842	1.244565344
2896	KDM6B	11	1	-2.083978292	1.552296496
2936	CHD4	11	1	-2.189996239	1.169537044
2939	EFNB3	11	1	-3.016872998	2.446130252
2961	SSTR2	11	1	-2.730184432	1.368098951
2970	CD83	11	1	-2.534934398	2.123432136
2993	GNB4	11	1	-2.770066377	0.84512887
18	STMN1	12	2	3.403474931	8.487151361
24	CLSPN	12	2	3.068587188	8.754028535
30	CDC20	12	2	3.751262311	8.562890268
31	KIF2C	12	2	3.641365174	8.564973093
43	DEPDC1	12	2	3.614223603	9.042968965
67	IQGAP3	12	2	3.299972419	9.123677469
73	NUF2	12	2	3.692174558	8.569081521
88	ASPM	12	2	3.714506749	8.616898752

89	KIF14	12	2	3.649994496	8.895631052
90	UBE2T	12	2	3.157400493	8.912328935
99	NEK2	12	2	3.565468434	9.070835329
101	CENPF	12	2	3.677714948	8.754243112
108	RRM2	12	2	2.961574916	8.982947565
113	CENPA	12	2	3.825363759	8.793129182
136	BUB1	12	2	3.69551957	9.197079874
138	CKAP2L	12	2	3.806388501	8.65024302
146	SPC25	12	2	3.327438001	8.865074373
178	SGO1	12	2	3.512039784	8.713175989
184	KIF15	12	2	3.76894391	8.671129442
230	TACC3	12	2	3.581304196	9.003450609
238	NCAPG	12	2	3.23760641	8.976267076
256	CENPE	12	2	3.645845059	9.150509096
266	MND1	12	2	3.001380805	8.782643533
285	CENPK	12	2	3.277207021	8.555779672
287	CCNB1	12	2	3.744881276	8.64231608
314	KIF20A	12	2	3.672846917	8.979392267
315	CDC25C	12	2	3.431398276	8.727650857
325	PTTG1	12	2	3.607154969	8.524603105
326	HMMR	12	2	3.66321433	8.482322908
353	KIFC1	12	2	3.369239215	8.983081079
368	TTK	12	2	3.579837445	8.459645486
382	CENPW	12	2	3.350755099	8.500416017
410	ANLN	12	2	3.460287217	9.006686426
437	PTN	12	2	3.424003247	8.318608499
458	KIF4A	12	2	3.659793977	9.127165056
484	ESCO2	12	2	2.93957961	8.942677713
485	PBK	12	2	3.442429427	8.734367586
521	CKS2	12	2	3.409454946	8.523445344
573	FAM111B	12	2	2.958305243	8.857108331
619	CDK1	12	2	3.547153596	8.522653795
633	CEP55	12	2	3.607273225	9.069931245
646	MKI67	12	2	3.630406026	8.795268274
651	CDCA3	12	2	3.550735597	8.494592882
696	IL26	12	2	3.832064752	8.552350259
721	SKA3	12	2	3.398296241	8.739092088
731	DIAPH3	12	2	3.681448583	9.035937524
743	CDKN3	12	2	3.595445279	8.537924982
745	DLGAP5	12	2	3.843502168	8.628337121
766	BUB1B	12	2	3.524670247	9.197941995
767	KNL1	12	2	3.652652387	8.98748324
768	NUSAP1	12	2	3.531965379	8.617204881
774	CCNB2	12	2	3.596353654	9.067372537
781	PCLAF	12	2	3.202027444	8.570656038
784	KIF23	12	2	3.293537024	9.058755136
809	SHCBP1	12	2	3.602599267	9.08297751
836	AURKB	12	2	3.294551019	9.108410097
841	TOP2A	12	2	3.606953267	8.748839593
855	PRR11	12	2	3.583638791	8.438558794
867	JPT1	12	2	3.407439593	8.280010438
873	TK1	12	2	3.102904681	8.663968301
874	BIRC5	12	2	3.706870202	8.533872819
882	TYMS	12	2	3.112488154	8.789431787
892	SKA1	12	2	3.453592662	9.18105433
908	TPX2	12	2	3.521724824	8.984121538
918	UBE2C	12	2	3.471487168	8.594823099
923	AURKA	12	2	3.646742944	8.705458856

936	UHRF1	12	2	2.883560542	8.92184279
943	SPC24	12	2	3.844262246	8.656840539
992	GTSE1	12	2	3.762656335	8.721543527
1015	CDCA2	12	2	3.777838353	8.659799791
1041	LMNB1	12	2	3.428483132	8.982430673
1095	CENPM	12	2	2.905862693	8.817216135
1098	MELK	12	2	3.333729629	9.118471361
1110	DTL	12	2	2.890904311	8.90582583
1170	UBE2S	12	2	3.356550578	8.419344163
1192	CCNA2	12	2	3.534975413	8.840443826
1207	ECT2	12	2	3.661875848	9.100925661
1211	KPNA2	12	2	3.286791448	8.625941492
1266	PARPBP	12	2	3.462509755	8.920558191
1283	HIST1H1B	12	2	3.347885255	8.764913774
1296	HMGB3	12	2	3.761639241	8.633555627
1299	HJURP	12	2	3.529138211	9.191822267
1359	PRC1	12	2	3.685908917	9.082749582
1426	SAPCD2	12	2	3.608561162	8.485917306
1430	ZWINT	12	2	2.901899938	8.876316286
1437	HIST1H3B	12	2	3.539506558	8.661895967
1446	KIF11	12	2	3.55079997	8.465229249
1479	MAD2L1	12	2	3.33210719	8.736201501
1480	CENPN	12	2	3.436879043	8.87620852
1493	SGO2	12	2	3.593089704	9.082030511
1510	ARHGAP11/	12	2	3.53004754	9.030932641
1538	CKAP2	12	2	3.768263463	8.726842142
1563	PLK1	12	2	3.463514213	9.022279954
1573	TROAP	12	2	3.827673081	8.64322207
1598	CDCA5	12	2	3.319064502	8.751129365
1603	TRIP13	12	2	3.662266377	9.17534659
1637	MCM10	12	2	3.419077758	8.45832274
1663	HIST1H2BB	12	2	3.372221831	8.773305154
1751	CDCA8	12	2	3.551800374	9.177706933
1770	NDC80	12	2	3.789133195	8.676910615
1838	SDC1	12	2	2.930112246	8.824852205
1866	FAM83D	12	2	3.508309726	8.662833429
1873	BICDL1	12	2	3.3538667	9.06823752
1882	SKA2	12	2	3.409661416	8.311764932
2093	FOXM1	12	2	3.568416718	8.768885828
2196	E2F1	12	2	2.962023858	8.919206834
2204	GGH	12	2	3.563804749	8.259521699
2300	HIST1H3G	12	2	3.536480073	8.654273248
2351	DDX39A	12	2	3.196923617	8.754046655
2363	PCNA	12	2	2.935809258	8.867784715
2415	CCDC34	12	2	3.53544772	8.488035417
2448	PIMREG	12	2	3.379508618	9.113792634
2457	H2AFV	12	2	3.703525666	8.497667528
2479	HIST1H3J	12	2	3.150552634	8.837428308
2519	ARSI	12	2	3.352075938	8.962183214
2571	DTYMK	12	2	3.418461207	8.3259022
2614	PLK4	12	2	3.147909764	9.063962198
2644	NCAPH	12	2	3.548485402	9.129957414
2668	SMC4	12	2	3.426675204	9.045560098
2782	GMNN	12	2	3.269877318	8.436579919
2784	ZNF93	12	2	3.623541955	9.11549685
2851	HELLS	12	2	2.949192409	8.803106523
2892	CDC45	12	2	2.869129304	8.895782686
2910	RAD51AP1	12	2	3.059619311	8.826534486

2935	KIF18A	12	2	3.790161256	8.653631425
2945	CDC6	12	2	2.953404311	8.962233758
2952	SMC2	12	2	2.96415389	8.952857233
2954	RACGAP1	12	2	3.405040387	9.087572313
2979	LRR1	12	2	3.05038083	8.752709604
2981	CXCL5	12	2	2.882351283	8.894482828
2982	RTKN2	12	2	3.267578248	8.850549913
2987	H2AFX	12	2	3.368056182	8.48710177
2992	CDCA7	12	2	2.889016274	8.828724122
17	TMEM50A	13	1	-0.47700081	0.486970342
55	ECM1	13	1	-1.357852157	-0.544383966
70	ADAMTS4	13	1	-1.267632481	0.36803881
72	UAP1	13	1	-0.84596556	-0.520486125
77	F5	13	1	-1.212549683	0.122807807
96	CD55	13	1	-0.970819887	-0.530217015
142	TNFAIP6	13	1	-1.390816923	-0.11686846
211	CHST2	13	1	-1.243229803	0.291090942
232	MSX1	13	1	-1.367036756	0.406236089
299	GLRX	13	1	-1.163589772	-0.109007993
346	SOX4	13	1	-1.410610732	0.492923475
381	GJA1	13	1	-1.275675472	0.051459647
439	KDM7A	13	1	-1.239139911	-0.149407514
480	SLC39A14	13	1	-0.756264891	-0.630175465
502	CTHRC1	13	1	-2.147434946	-0.058676564
519	CTSL	13	1	-1.138274725	0.064933604
531	PALM2	13	1	-1.772332188	0.797202445
541	ENG	13	1	-1.172953244	0.307611919
544	PRRX2	13	1	-1.240076836	0.197911954
545	PTGES	13	1	-1.003400084	-0.059552872
552	IFITM2	13	1	-1.61118054	-0.104602792
559	SPON1	13	1	-1.330313262	0.186678803
567	CD44	13	1	-0.948347118	-0.554352873
592	SERPINH1	13	1	-2.036255475	0.836501933
606	CLMP	13	1	-0.585545834	-0.451722355
660	GPRC5A	13	1	-0.85759386	-0.218537704
694	SLC16A7	13	1	-1.334717985	-0.355382039
724	MEDAG	13	1	-1.33298957	0.068246729
739	MMP14	13	1	-1.149084087	-0.012811654
746	HIF1A	13	1	-1.03457826	-0.108021879
776	C2CD4A	13	1	-1.1616829	0.13174758
791	CEMIP	13	1	-1.185969766	0.106679386
798	ANPEP	13	1	-1.057226475	0.102403379
842	IGFBP4	13	1	-1.946054336	0.11886189
856	VMP1	13	1	-1.289581653	0.501545048
884	RAB31	13	1	-1.313625749	0.339923656
921	CEBPB	13	1	-1.029444095	0.378411508
988	A4GALT	13	1	-1.441947635	0.158566124
993	TYMP	13	1	-0.985104915	0.261216319
995	ADAMTS5	13	1	-1.359648105	-0.14500437
1054	PITPNC1	13	1	-1.474125143	0.575662291
1061	PHC2	13	1	-1.049657103	0.210349477
1066	FJX1	13	1	-1.278270777	0.239312685
1121	ITPRIP	13	1	-1.074934836	0.151339001
1145	PDE3A	13	1	-1.509217497	0.178895271
1255	SMIM14	13	1	-0.910225745	-0.542854437
1275	B4GALT1	13	1	-1.093263503	-0.255447553
1322	CD276	13	1	-1.110265251	0.118003345
1324	AKR1C3	13	1	-1.485019918	0.147252655

1389	NAMPT	13	1	-1.178251084	0.417751587
1429	PLIN2	13	1	-1.595682617	0.716614104
1433	STEAP1	13	1	-1.537312385	-0.207911589
1434	ABHD17C	13	1	-1.154138084	0.061485893
1496	ACTN1	13	1	-0.375761103	-0.083352887
1509	ADAMTS6	13	1	-0.706287738	-0.459742257
1529	ANKH	13	1	-1.158624764	0.125102169
1549	NTRK3	13	1	-1.228449758	0.081906504
1562	THBD	13	1	-1.457552966	0.22559098
1574	SOCS2	13	1	-1.335759398	0.466076827
1577	SAMD11	13	1	-1.362804111	0.334609426
1632	SLC7A2	13	1	-1.035272118	-0.412229703
1660	FAT1	13	1	-1.930495378	0.578596867
1665	BCL3	13	1	-1.215023931	0.450711167
1692	FST	13	1	-1.113511857	0.484034098
1831	SMIM3	13	1	-0.956163432	0.242949284
1850	SOD2	13	1	-0.985505041	-0.510317215
1880	OSMR	13	1	-0.882101681	-0.321806228
1933	LHFPL2	13	1	-1.777322289	0.606566763
1934	SIGIRR	13	1	-1.511914965	-0.198649117
1950	ECE1	13	1	-1.9258877	0.295729554
1960	FHAD1	13	1	-1.34156638	0.290098405
1981	SH3PXD2B	13	1	-1.423642453	0.343430853
1992	CMKLR1	13	1	-1.576612946	0.244306779
2118	UNC5B	13	1	-1.414327141	0.361156738
2175	HMOX1	13	1	-0.928463962	-0.046714866
2228	C2CD4B	13	1	-1.200374361	0.229588187
2232	SYNJ2	13	1	-1.175024625	-0.032657051
2234	REEP3	13	1	-0.446247551	0.065518415
2258	CPT1A	13	1	-1.307785269	0.259756363
2287	C1GALT1	13	1	-1.039527114	-0.686739945
2328	FAM126A	13	1	-1.724493381	0.756120778
2349	STMN3	13	1	-1.195589956	0.14356435
2473	MAPKAPK2	13	1	-1.125835475	0.192517555
2511	TMTC1	13	1	-1.460094329	-0.18175722
2554	GEM	13	1	-1.138302501	0.548238314
2570	KDSR	13	1	-1.266451236	-0.311655077
2585	GLIS3	13	1	-1.568276759	0.701255775
2590	ACSL4	13	1	-1.105061348	0.110624916
2616	PLXNA1	13	1	-0.3821509	-0.081584656
2618	CSF3	13	1	-1.198520478	0.128728456
2634	KDM7A-DT	13	1	-1.204533275	-0.028165513
2649	LINC01615	13	1	-1.78447568	0.242407835
2673	STK24	13	1	-0.858264234	-0.492792689
2718	SLC3A2	13	1	-0.761234667	0.200251079
2751	ST3GAL1	13	1	-0.769873287	0.087819016
2775	FGF10	13	1	-1.360321637	-0.069994711
2885	CSGALNAC	13	1	-1.109521862	0.134876019
2906	HRH1	13	1	-1.24916869	-0.476164573
2917	SZRD1	13	1	-1.163537737	0.043221033
2931	CCDC71L	13	1	-1.156235214	-0.256864169
2956	PGM2L1	13	1	-1.371192809	0.676493144
5	RPL22	14	1	0.576508764	3.184490896
128	TMSB10	14	1	-0.119151529	2.685775972
182	RPSA	14	1	0.701571945	3.063237882
335	RACK1	14	1	0.479902033	3.28667543
449	TMSB4X	14	1	-0.182792392	2.83088944
582	MALAT1	14	1	-0.179247763	3.47552917

649	GAPDH	14	1	0.268060271	3.515959716
661	MGP	14	1	0.007267866	3.509022689
690	NACA	14	1	0.429901604	3.514800525
785	RPLP1	14	1	-0.0072028	3.53839562
813	MT2A	14	1	-0.1882095	3.486541009
1073	RPL10A	14	1	0.535500411	3.222472644
1076	RPL27A	14	1	0.411486868	3.334819532
1104	IFITM3	14	1	0.057238761	3.44822476
1106	RPS7	14	1	0.208424691	3.510693765
1125	EEF1A1	14	1	0.012287859	3.553529001
1154	PFDN5	14	1	0.603938106	3.145858264
1171	RPL14	14	1	0.266512934	3.582789875
1173	RPS15A	14	1	0.184343819	3.433697677
1252	RPL5	14	1	0.487006608	3.388265586
1317	RPL7	14	1	0.476427618	3.316873765
1333	RPL29	14	1	0.30053753	3.469304538
1342	RPL35A	14	1	0.209434453	3.552574134
1404	RPL39	14	1	0.090849642	3.653363443
1420	RPL10	14	1	0.080125276	3.565030075
1467	FTL	14	1	0.055506889	3.509540058
1476	UBA52	14	1	0.233078245	3.517117477
1499	RPS5	14	1	0.505776886	3.245394445
1561	HMGB1	14	1	0.633021001	3.045348621
1570	RPS20	14	1	0.367045645	3.402146554
1593	VIM	14	1	0.064211849	3.49210403
1601	RPL41	14	1	0.022098009	3.540869689
1697	FAU	14	1	0.409126584	3.373676038
1700	RPS25	14	1	0.348053996	3.474358774
1767	RPS3	14	1	0.414558295	3.37778399
1778	RPL15	14	1	0.304830316	3.388079143
1780	RPL35	14	1	0.194821004	3.498164392
1797	RPS12	14	1	0.015417192	3.679009653
1843	RPL7A	14	1	0.407182757	3.365180231
1884	RPL23	14	1	0.413961176	3.341756082
1895	RPL6	14	1	0.397852067	3.427725053
1937	RPL11	14	1	0.156228546	3.583801723
1943	RPL26	14	1	0.250135783	3.631840682
1951	RPL34	14	1	0.101439122	3.665031171
1993	B2M	14	1	0.039896313	3.491278148
1994	RPS3A	14	1	0.234218244	3.495836235
1996	RPS29	14	1	0.190693501	3.559223867
2041	H3F3B	14	1	0.346578602	3.439423776
2088	RPL37	14	1	0.151274089	3.598927474
2100	RPL24	14	1	0.304390315	3.458112455
2102	RPL38	14	1	0.311317984	3.456449485
2130	RPS13	14	1	0.276731078	3.458462215
2134	RPS9	14	1	0.303100172	3.410164333
2143	S100A6	14	1	-0.03100222	3.186690307
2151	RPS4X	14	1	0.3033554	3.558568216
2153	RPS15	14	1	0.163907293	3.618176675
2154	HBB	14	1	-0.182582881	2.876548267
2192	RPS28	14	1	0.047375683	3.601581312
2208	RPL36	14	1	0.104344074	3.584059692
2220	RPL9	14	1	0.392537538	3.379714704
2224	HBA1	14	1	-0.219585609	2.789912916
2248	RPS14	14	1	0.085253123	3.6670501
2311	RPS18	14	1	0.116953794	3.602162815
2322	HBA2	14	1	-0.189397421	2.840065456

2324	RPL30	14	1	0.130285624	3.595311857
2331	RPL12	14	1	0.188512091	3.569528556
2339	RPS16	14	1	0.290319685	3.429471946
2357	PTMA	14	1	0.233610634	3.615502334
2377	RPL18	14	1	0.230955903	3.530613876
2388	RPS6	14	1	0.226964179	3.486763454
2421	GNG11	14	1	-0.224498745	2.635043121
2442	RPL13	14	1	0.06758887	3.558050132
2483	RPL32	14	1	0.143833045	3.595838523
2546	RPS2	14	1	0.165571097	3.451988197
2555	FTH1	14	1	0.146189634	3.658355451
2556	RPL23A	14	1	0.172209028	3.572514034
2565	RPS11	14	1	0.402336005	3.33985231
2586	RPS27A	14	1	0.090179626	3.566229082
2636	RPL3	14	1	0.311770085	3.450379587
2647	RPS27	14	1	0.050215725	3.532373643
2679	RPL8	14	1	0.313539211	3.39176128
2716	RPLP0	14	1	0.314266507	3.435644365
2729	RPS24	14	1	0.145156685	3.581520534
2752	RPLP2	14	1	0.150329653	3.519165731
2854	RPS23	14	1	0.146523956	3.579984642
2855	AQP1	14	1	-0.308862563	2.631386257
2868	RPL19	14	1	0.173485104	3.609991765
2886	ACTB	14	1	-0.065000918	2.68294189
2984	RPL18A	14	1	0.036521766	3.469323373
14	CAMK2N1	15	1	-3.485271331	0.456007696
54	TXNIP	15	1	-3.402074452	0.431455112
104	EPHX1	15	1	-3.638953801	0.240349627
141	ZEB2	15	1	-3.644869443	0.183173395
172	COL6A3	15	1	-3.179459687	0.226088739
192	PTPRG	15	1	-3.170678731	0.372955239
200	PHLDB2	15	1	-3.487510558	0.196930564
244	IGFBP7	15	1	-4.010564204	0.798007346
249	ANTXR2	15	1	-3.411494847	0.181703127
263	SPRY1	15	1	-3.472274419	0.607292092
281	C7	15	1	-3.586313602	0.470481074
282	PLCXD3	15	1	-3.64691722	0.593843556
283	SELENOP	15	1	-3.505513307	0.227055943
284	ESM1	15	1	-3.317743894	1.014773703
313	SPOCK1	15	1	-3.373708125	0.146958358
321	PDGFRB	15	1	-3.010810252	0.447317458
443	TMEM176B	15	1	-3.377135154	0.304502404
444	TMEM176A	15	1	-3.335381623	0.440827883
448	ARHGAP6	15	1	-3.460408088	0.330524242
451	SRPX	15	1	-3.718406554	0.066918499
493	PREX2	15	1	-3.659113284	0.669696784
494	SULF1	15	1	-3.249280807	0.106980271
533	SVEP1	15	1	-3.286686536	0.063814557
553	IFITM1	15	1	-3.551653501	0.519496537
614	NRP1	15	1	-3.497439023	0.638612783
616	CXCL12	15	1	-3.268245336	0.244912541
659	CLEC2B	15	1	-3.534863349	0.314527429
663	EPS8	15	1	-3.610379573	0.200319148
668	ABCC9	15	1	-3.190674659	0.072543151
772	FGF7	15	1	-3.509688016	0.443898058
794	ADAMTSL3	15	1	-3.365839597	0.056052512
819	CDH11	15	1	-3.451939698	0.064804829
871	CYGB	15	1	-3.508026	0.585726297

914	MAFB	15	1	-3.393801566	-0.213866548
922	TSHZ2	15	1	-3.420494433	0.238115228
968	APOE	15	1	-3.335764762	1.182721353
969	APOC1	15	1	-3.425331231	0.509070015
978	GGT5	15	1	-3.480774756	0.552195287
1025	SLIT2	15	1	-3.602061149	-0.056821578
1068	HHIP	15	1	-3.64888775	0.641345239
1126	LEPR	15	1	-3.261314746	0.541518605
1146	PDE5A	15	1	-3.524875279	0.801845885
1156	PTGER4	15	1	-3.403035279	0.357456899
1190	AC092651.2	15	1	-3.637753125	0.774378872
1221	ANGPT1	15	1	-3.440309163	0.564967311
1260	ZFP36L2	15	1	-3.56786048	0.138656027
1273	TMEM150C	15	1	-3.402759191	0.439224339
1279	GHR	15	1	-3.335438605	0.09008897
1325	NID1	15	1	-2.969719525	0.163671887
1335	SAMHD1	15	1	-3.401790734	0.08619923
1408	KCNE4	15	1	-3.032790776	0.381384886
1412	ETS1	15	1	-3.212140676	0.271575904
1513	PLEKHA5	15	1	-3.363113996	0.202825165
1516	PTGIR	15	1	-3.373304244	0.607782758
1546	FRMD6	15	1	-3.068199988	0.325435198
1589	DACT1	15	1	-3.617638227	0.699728942
1638	TMEM204	15	1	-3.416009542	0.575840509
1711	TNFSF10	15	1	-3.437945243	0.139615781
1746	FZD4	15	1	-3.247232314	0.006198323
1769	TRIM22	15	1	-3.483527061	0.466258622
1802	SYNPO2	15	1	-3.183954593	0.997590161
1874	KITLG	15	1	-3.440804835	0.152438796
1889	ANGPT2	15	1	-3.577660199	0.776703334
1983	ARHGDIB	15	1	-3.633478757	0.832869149
2006	PGM5-AS1	15	1	-3.499916907	0.687261558
2114	RAI14	15	1	-3.684653159	0.835358954
2124	C16orf89	15	1	-3.467893716	0.192938126
2165	SCN4B	15	1	-3.558559056	0.787502623
2207	CCDC102B	15	1	-3.528544065	0.746630049
2333	AP001528.3	15	1	-3.468482371	-0.018174403
2338	RFTN1	15	1	-3.536741849	0.719668603
2461	STK17B	15	1	-3.535259362	0.901570416
2480	CLSTN2	15	1	-3.336270209	0.355516291
2541	AGT	15	1	-3.420489188	0.708003855
2583	DPYD	15	1	-3.242216464	0.061900235
2625	RCAN2	15	1	-3.600967046	0.711192823
2676	FMO2	15	1	-3.34670293	0.595923639
2732	LRRC17	15	1	-3.550418016	0.43838892
2869	IRF2BP2	15	1	-3.480524417	0.354648626
2904	NAV2	15	1	-3.253241178	0.855311728
2946	APLNR	15	1	-3.337772723	0.892319179
2953	NPNT	15	1	-3.366294261	0.907034374
2962	NRXN2	15	1	-3.325460788	0.56279794
2964	HGF	15	1	-3.502755042	0.435578084
25	RHBDL2	16	1	-3.514295932	3.610917306
66	BGLAP	16	1	-3.535849925	3.727621294
157	SATB2	16	1	-3.42018139	3.472193933
183	ENTPD3	16	1	-3.540523168	3.72701571
206	CHST13	16	1	-3.55460846	3.738204217
226	LRRC15	16	1	-3.406188842	3.490748859
369	PRSS35	16	1	-3.530192014	3.704704023

497	CA3	16	1	-3.444236394	3.684729314
510	COL22A1	16	1	-3.540044185	3.752682424
554	SCT	16	1	-3.534137603	3.71893332
561	INSC	16	1	-3.35618436	3.419440961
565	ANO5	16	1	-3.477416631	3.638561464
621	COL13A1	16	1	-3.503156777	3.657163358
683	SP7	16	1	-3.49695551	3.574583507
710	TMEM119	16	1	-3.2939738	3.277392602
750	SLC8A3	16	1	-3.492571469	3.664796091
820	SMPD3	16	1	-3.532806274	3.678762651
830	CDH15	16	1	-3.519935485	3.625619626
1078	HOMER2	16	1	-3.319049712	3.374820209
1205	CREB3L1	16	1	-3.430140134	3.538380361
1216	CADM1	16	1	-3.465190764	3.531771875
1256	NPTX2	16	1	-3.427505132	3.570238328
1344	GALNT3	16	1	-3.482136127	3.61886499
1360	QPCT	16	1	-3.510838862	3.709789491
1367	CD36	16	1	-3.490944739	3.637363887
1370	LRP4	16	1	-3.284866925	3.296293474
1659	PHOSPHO1	16	1	-3.517681476	3.706505514
1702	SGMS2	16	1	-3.445779677	3.671183801
1730	LINC02515	16	1	-3.352326747	3.386285758
1903	CDH2	16	1	-3.497801896	3.644515729
1913	WIF1	16	1	-3.324487325	3.383673168
1917	GLDC	16	1	-3.326657411	3.315322137
1925	NPW	16	1	-3.262021895	3.154943681
1959	MYO1B	16	1	-3.540010568	3.644917226
2101	UBE2QL1	16	1	-3.52586877	3.73267243
2105	APCDD1	16	1	-3.500464316	3.613395668
2129	NCAM1	16	1	-3.299807187	3.36911819
2133	NCS1	16	1	-3.12872326	3.163464523
2226	CDC42EP3	16	1	-3.294142123	3.339627004
2264	PARD6G	16	1	-3.294071313	3.291843152
2386	NPB	16	1	-3.285649177	2.970948911
2463	MIR181A1H	16	1	-3.432771321	3.516468263
2489	ADAMTS18	16	1	-3.257857915	3.028555608
2522	CLDN11	16	1	-3.289797898	3.319897867
2523	PTH1R	16	1	-3.242498275	3.261299825
2611	TP53I11	16	1	-3.365023728	3.438644147
2630	CD24	16	1	-3.537444707	3.725070215
2733	COL24A1	16	1	-3.486270782	3.689559198
2754	C1QTNF1	16	1	-3.379723426	3.436479307
2757	CSRP2	16	1	-3.248336907	3.162293172
2811	SATB2-AS1	16	1	-3.495213862	3.644941783
2985	MMP11	16	1	-3.402150508	3.486115194
