# Supplementary material for: Spatially defined single-cell transcriptional profiling characterizes diverse chondrocyte subtypes and nucleus pulposus progenitors in human intervertebral discs
Source: Bone Res. 2021 Aug 16;9:37. doi: 10.1038/s41413-021-00163-z (PMC8368097; doi:10.1038/s41413-021-00163-z)
Supplement: Supplementary file 10 — Supplementary Table 9 [file 41413_2021_163_MOESM10_ESM.pdf]

Supplementary Table 9. Pairwise cellular interaction counts among NP subclusters by Cellchat analysis														
	NPPC-1	NPPC-2	NPPC-3	NPPC-4	Fib1	Fib2	Fib3	Chond1	Chond2	Chond3	Osteo	EC	Neu1	Pericyte
NPPC-1	63	52	68	46	116	6	182	57	6	98	149	88	34	97
NPPC-2	74	69	88	62	149	17	206	70	19	126	176	109	55	124
NPPC-3	81	69	81	58	156	10	209	78	9	125	180	108	44	126
NPPC-4	64	51	69	37	135	12	199	41	11	98	159	93	42	107
Fib1	111	104	123	84	172	2	208	108	11	150	192	117	53	147
Fib2	6	3	13	3	20	0	78	2	0	19	41	21	11	29
Fib3	152	145	160	121	195	51	238	143	70	176	220	155	100	179
Chond1	59	60	73	31	110	4	155	66	11	96	145	85	35	86
Chond2	13	12	17	9	27	0	89	19	0	42	50	17	19	29
Chond3	92	79	102	60	134	8	171	84	27	112	162	101	34	118
Osteo	164	161	173	132	224	42	256	171	55	208	237	163	109	203
EC	88	93	95	84	131	26	193	81	27	114	142	117	61	134
Neu1	20	13	23	15	55	6	129	10	6	30	85	39	28	55
Pericyte	88	85	97	61	153	13	203	82	11	128	179	108	50	117

Pairwise cellular interaction counts among cell clusters from NP, CEP and AF by Cellchat analysis														
	NP-NPPC-1	NP-NPPC-2	NP-NPPC-3	NP-NPPC-4	CEP-Chond1	CEP-Chond2	CEP-Chond3	CEP-Pericyte	CEP-Stroma	AF-Chond1	AF-Chond2	AF-Chond3	AF-Pericyte	AF-Stroma
NP-NPPC-1	46	45	45	44	50	6	28	14	14	55	9	47	29	49
NP-NPPC-2	51	52	48	41	51	12	29	20	21	58	16	58	34	47
NP-NPPC-3	47	50	45	42	59	13	36	23	22	62	15	57	30	48
NP-NPPC-4	39	34	38	28	32	7	16	11	8	43	7	39	24	32
CEP-Chond1	63	64	67	45	42	14	42	12	17	47	28	57	34	48
CEP-Chond2	3	8	10	7	5	0	0	0	0	8	0	4	1	0
CEP-Chond3	28	36	34	16	31	0	14	0	1	36	1	38	6	21
CEP-Pericyte	11	21	19	12	1	1	1	4	1	2	1	3	9	4
CEP-Stroma	15	25	24	12	6	6	6	6	6	10	6	7	9	6
AF-Chond1	68	68	68	57	44	19	42	13	22	51	27	60	40	52
AF-Chond2	3	12	13	7	10	0	0	0	0	15	0	9	1	0
AF-Chond3	66	67	68	47	53	9	45	8	12	58	20	63	36	67
AF-Pericyte	34	33	34	28	17	4	5	12	4	25	4	22	27	27
AF-Stroma	66	63	61	50	46	10	28	13	13	50	12	72	36	52
