# Supplementary figures and images for: Spatially defined single-cell transcriptional profiling characterizes diverse chondrocyte subtypes and nucleus pulposus progenitors in human intervertebral discs
Source: Bone Res. 2021 Aug 16;9:37. doi: 10.1038/s41413-021-00163-z (PMC8368097; doi:10.1038/s41413-021-00163-z)

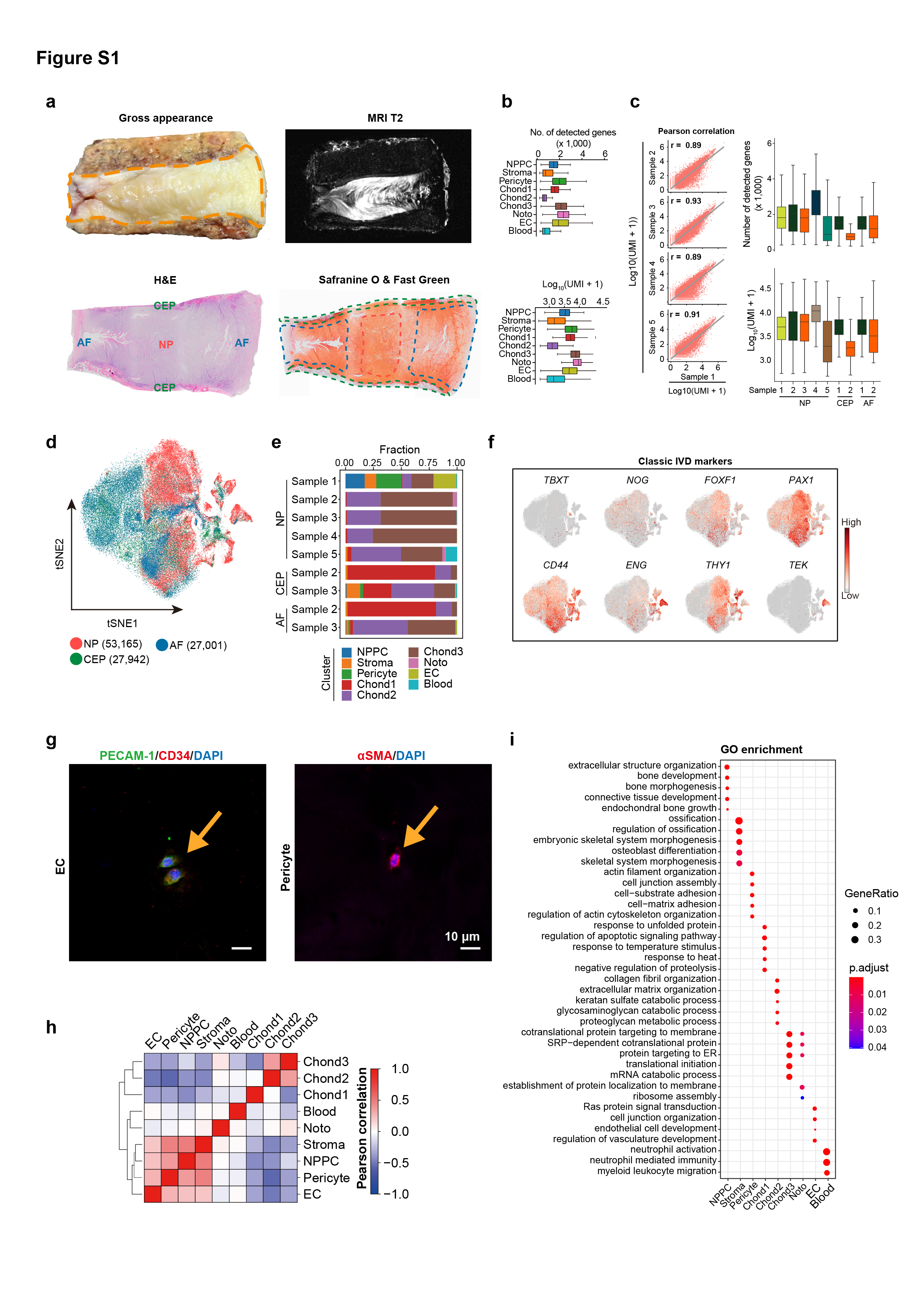

Supplement: Supplementary file 11 — Supplementary Figure 1 [file 41413_2021_163_MOESM11_ESM.jpg]

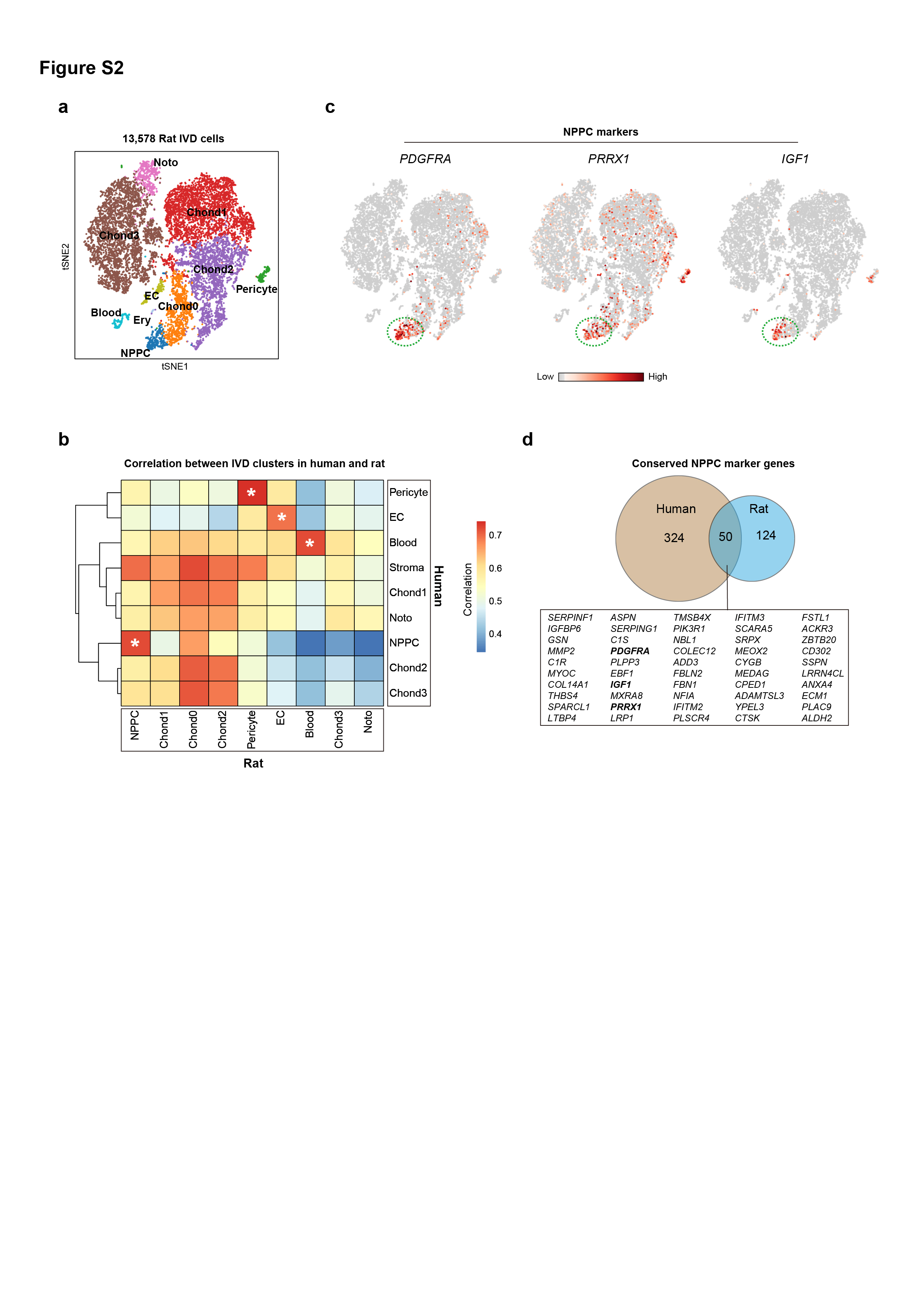

Supplement: Supplementary file 12 — Supplementary Figure 2 [file 41413_2021_163_MOESM12_ESM.jpg]

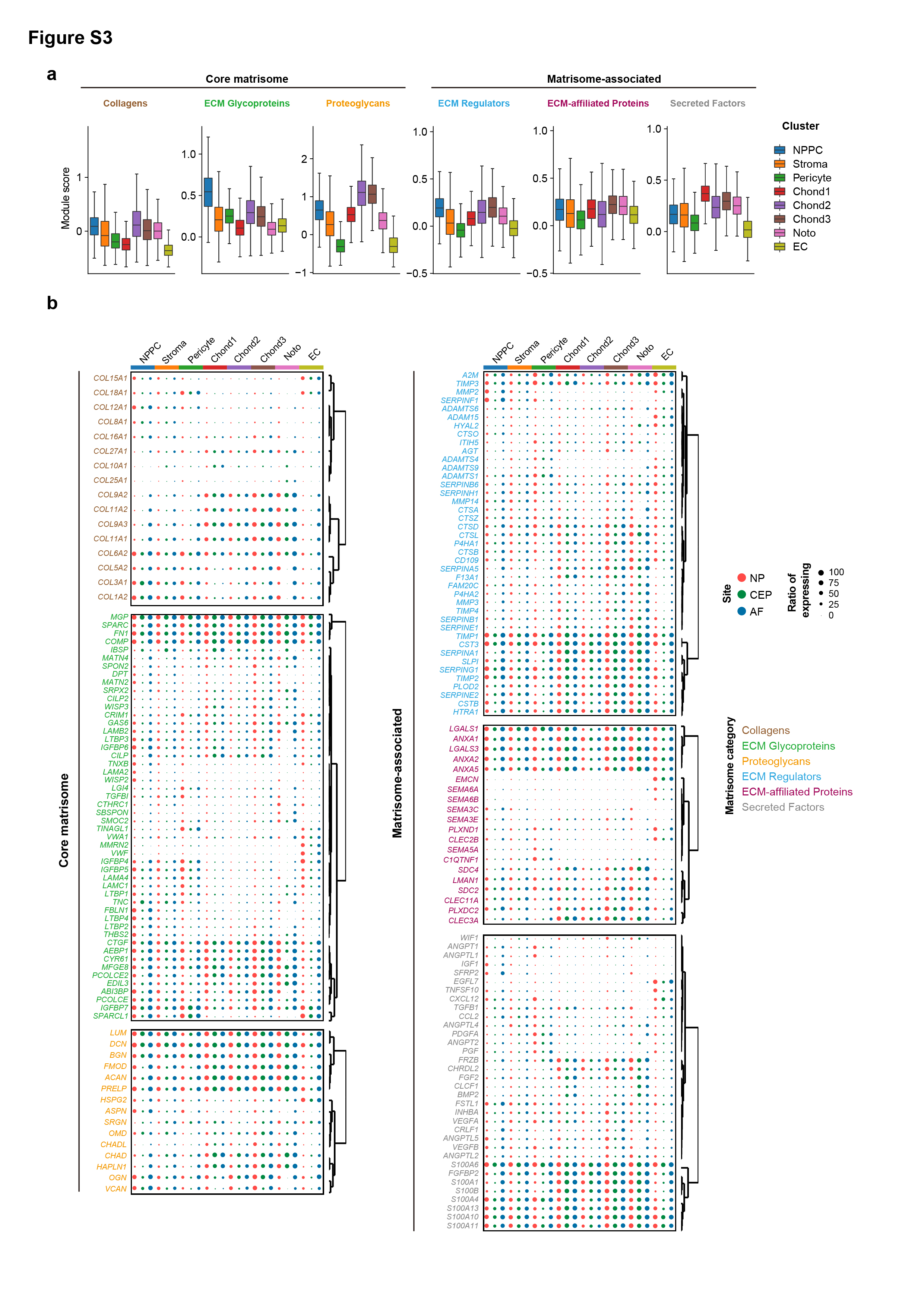

Supplement: Supplementary file 13 — Supplementary Figure 3 [file 41413_2021_163_MOESM13_ESM.jpg]

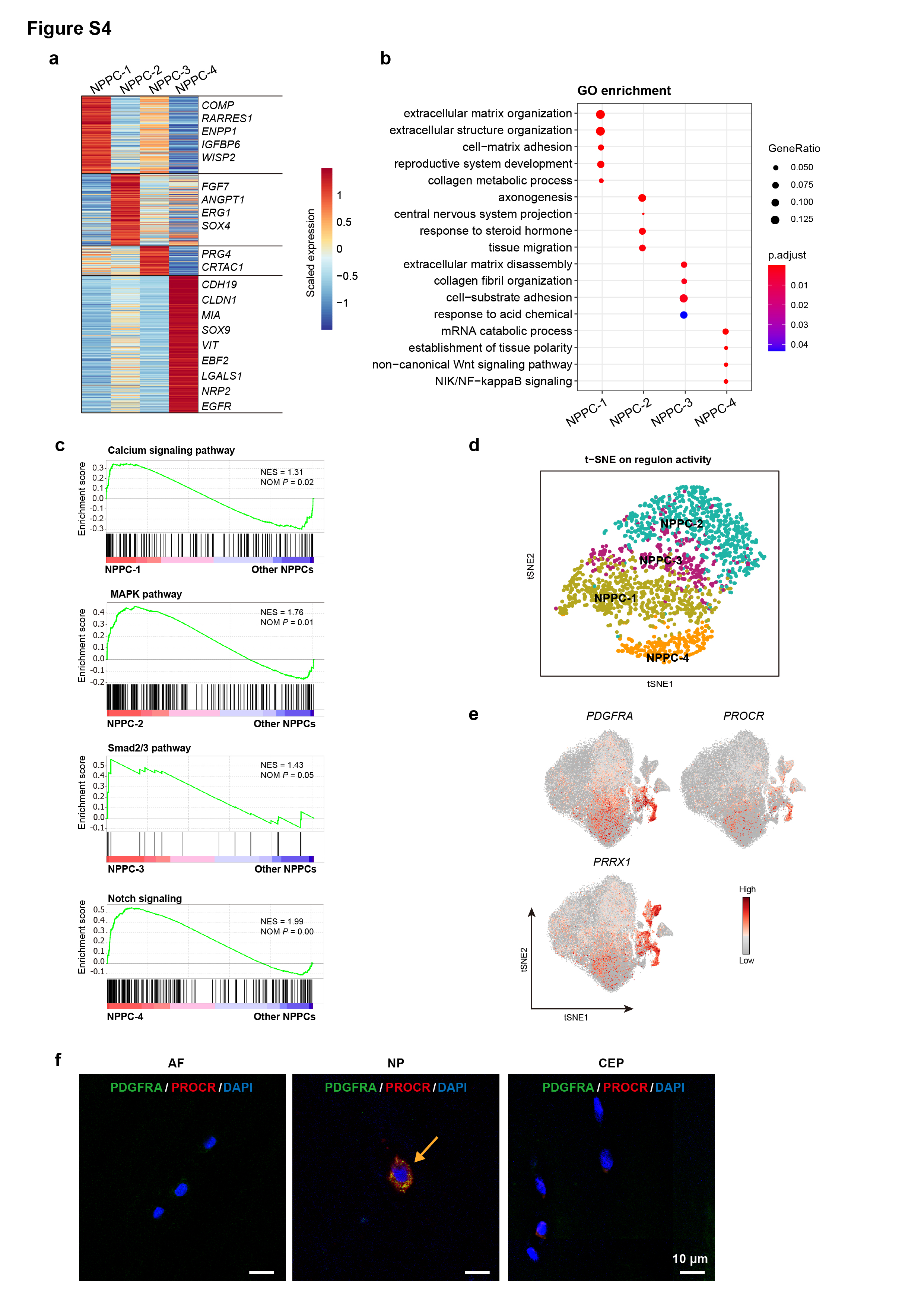

Supplement: Supplementary file 14 — Supplementary Figure 4 [file 41413_2021_163_MOESM14_ESM.jpg]

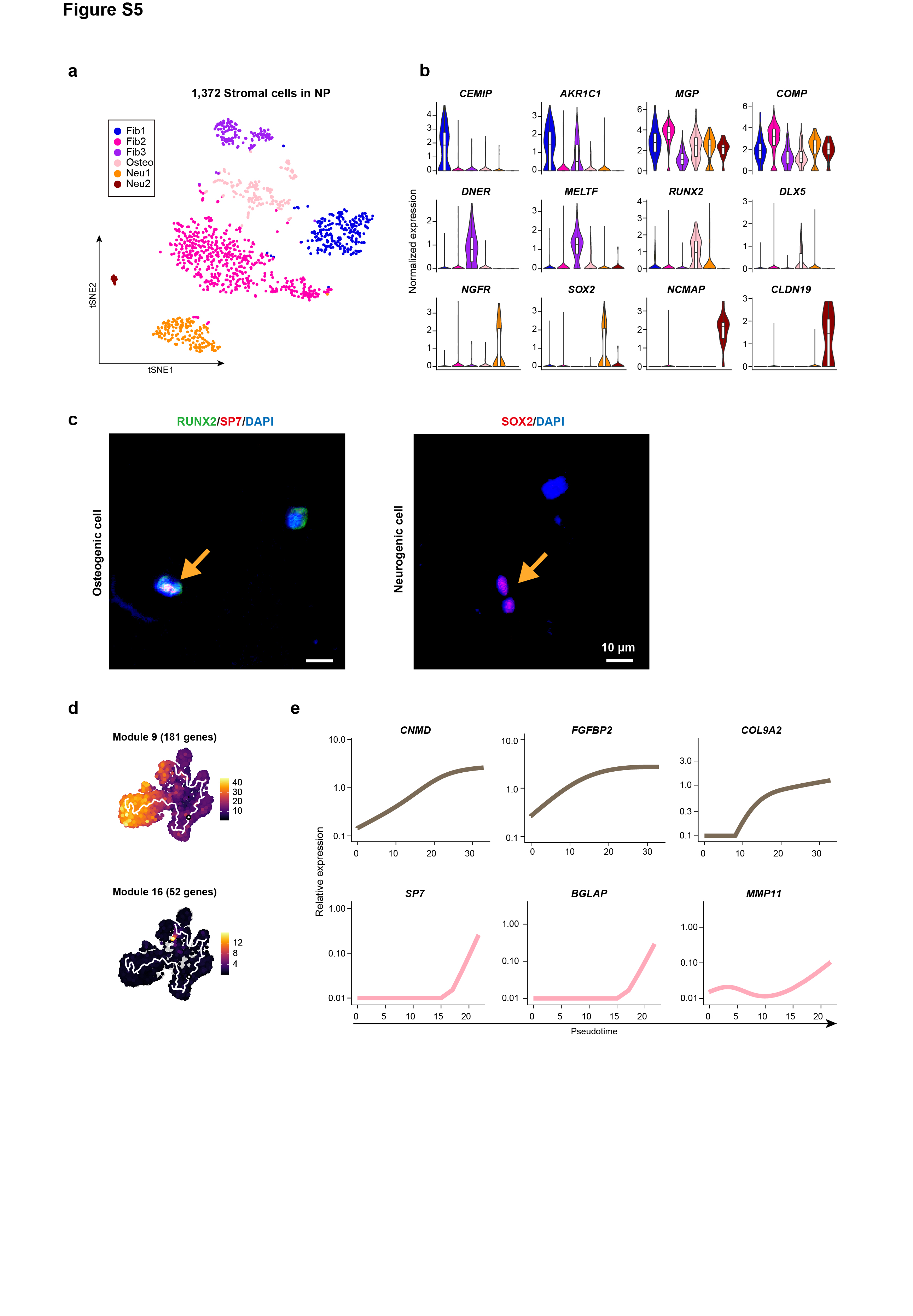

Supplement: Supplementary file 15 — Supplementary Figure 5 [file 41413_2021_163_MOESM15_ESM.jpg]

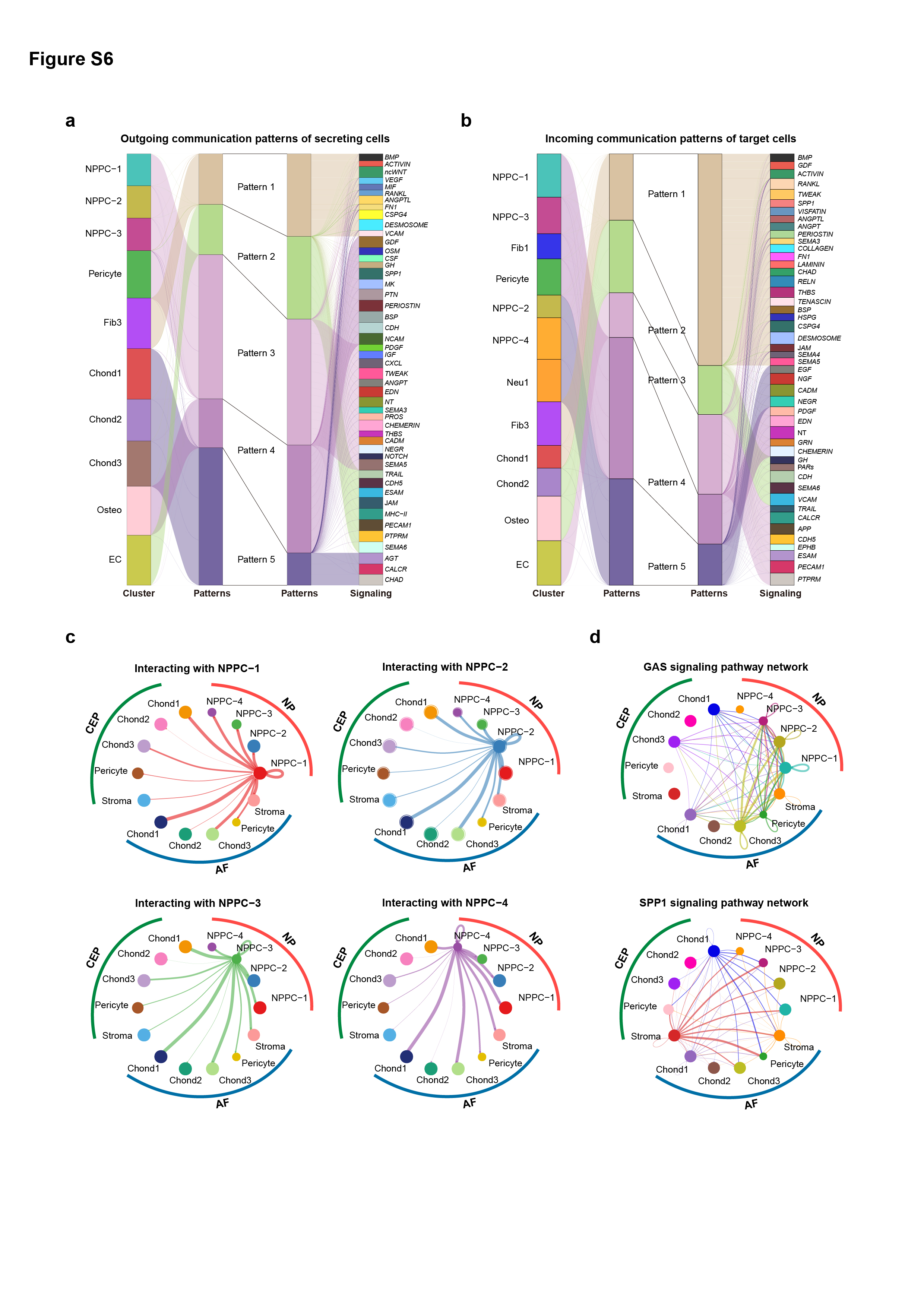

Supplement: Supplementary file 16 — Supplementary Figure 6 [file 41413_2021_163_MOESM16_ESM.jpg]
